# A protein interactions map of multiple organ systems associated with COVID-19 disease

**DOI:** 10.5808/gi.20078

**Published:** 2021-06-30

**Authors:** Dhammapal Bharne

**Affiliations:** Department of Biotechnology and Bioinformatics, University of Hyderabad, Hyderabad 500046, India

**Keywords:** cluster analysis, COVID-19, gene ontology, protein interaction maps, RNA-Seq

## Abstract

Coronavirus disease 2019 (COVID-19) is an on-going pandemic disease infecting millions of people across the globe. Recent reports of reduction in antibody levels and the re-emergence of the disease in recovered patients necessitated the understanding of the pandemic at the core level. The cases of multiple organ failures emphasized the consideration of different organ systems while managing the disease. The present study employed RNA sequencing data to determine the disease associated differentially regulated genes and their related protein interactions in several organ systems. It signified the importance of early diagnosis and treatment of the disease. A map of protein interactions of multiple organ systems was built and uncovered CAV1 and CTNNB1 as the top degree nodes. A core interactions sub-network was analyzed to identify different modules of functional significance. AR, CTNNB1, CAV1, and PIK3R1 proteins were unfolded as bridging nodes interconnecting different modules for the information flow across several pathways. The present study also highlighted some of the druggable targets to analyze in drug re-purposing strategies against the COVID-19 pandemic. Therefore, the protein interactions map and the modular interactions of the differentially regulated genes in the multiple organ systems would incline the scientists and researchers to investigate in novel therapeutics for the COVID-19 pandemic expeditiously.

## Introduction

Coronavirus disease 2019 (COVID-19) is a pandemic disease caused by the novel coronavirus, severe acute respiratory syndrome coronavirus 2 (SARS-CoV-2) (World Health Organization). As on 11 August 2020, it reported to infect more than 78 million and decease over 1.7 million people around the world (https://www.worldometers.info/coronavirus/). The disease cases are further escalating causing human sufferings. Currently, several vaccines are being evaluated at various clinical stages [1,2] and some available drugs are being investigated to re-purpose [3,4] in the treatment of manageable cases of the COVID-19 disease. The SARS-CoV-2 is a highly transmissible virus containing unusually larger RNA as genome and spike like glycoprotein envelope [5]. It is different from other corona viruses in having strong binding affinity with human cell surface receptors [6]. The virus begins the process of infection by binding to human cell receptors such as angiotensin-converting enzyme 2 (ACE2), transmembrane serine protease 2, cyclophilins, CD147, and CD26 [7]. The ACE2 are the functional receptors of SARS-CoV-2 and are distributed in the cells of lung, heart, kidney and intestinal tissues [8]. Therefore, the virus can transmit to several organ systems and evade the host immune response leading to multi-organ failure and death. Hence, infection in the multiple organs should be quickly assessed [9] to manage the individual patients early and reduce the risk of decompensation.

Recent report of re-emergence of the SARS-CoV-2 in a recovered patient [10] necessitated better understanding of the infection, contagion and pathology of the disease. IgG levels and neutralizing antibodies in the recovered patients were decreasing after few months [11]; however, the receptor-binding domain specific antibodies possessed the strong antiviral activity [12]. In addition to these recent findings, perspectives of gene regulations and protein interactions at multi-organ level could play significant role in gaining insights and therapeutic interventions of the disease. The rapid development of sequencing technologies in the past few decades made significant impact on research in molecular biology of viral diagnosis [13] and drug discovery [14]. RNA-sequencing technology provided unprecedented information about the novel and known gene structures and annotations from coding and non-coding transcripts. Analyzing RNA-sequencing data of multiple organ systems associated with COVID-19 could unveil several aspects of the pandemic disease. Therefore, the present study employed RNA-sequencing data of several organ systems from the SARS-CoV-2 infected and deceased individuals to analyze differentially expressed genes and interpret protein interactions that led to identification of several proteins for the therapeutic interventions.

## Methods

### Identification of differentially expressed genes

RNA sequencing data was obtained from autopsy specimens of lung, heart, jejunum, liver, kidney, bowel, marrow, fat, placenta and skin of 24 patients deceased due to COVID-19 infection. The total number of samples in the sequencing data was 88 including five negative control samples. The sequencing data was mapped to HG38 Human reference genome and processed using HTSeq-Count [15] to produce raw read counts of mRNA transcripts. Such transcripts read counts for each organ sample were retrieved from the NCBI Gene Expression Omnibus public repository using the accession number GSE150316. Transcripts with total read counts of only one or lesser were filtered out. The resulting transcripts of each of the organ system and the control samples were analyzed using DESeq2 [16] to identify differentially expressed genes (DEGs). The DEGs with the p ≤ 0.01, and with a log2 fold change of ≥1.5 and ≤‒1.5 was considered statistically significant up and down-regulated genes respectively. Further, the genes significantly regulated only in one organ system or in multiple organ systems were predicted.

### Construction and analysis of protein interactions map

Experimentally verified human protein-protein interactions (PPIs) were retrieved from the Database of Interacting Proteins (DIP) database [17]. From these PPIs, the interactions among the up or down-regulated gene products were extracted and the protein interactions of a specific organ system, multiple organ systems as well as cross-organ protein interactions were recognized. An interaction map of the resulted PPIs was constructed using R igraph [18]. The nodes in the interactions map were colored differently to distinguish the organ-specific, cross-organ, and the multi-organ protein interactions. Topological properties of the interactions map were analyzed to interpret the biological significance of the interactions map.

### Functional annotations and pathways of modules

The largest component in the protein interactions map was unwrapped as a core interactions sub-network. The core interactions sub-network was processed through edge betweenness clustering algorithm [19] to predict different modules in which the nodes were densely connected among themselves than the nodes of other modules. Each module and the nodes other than that of core interactions sub-network as a whole were employed using the PANTHER’s [20] over-representation analysis with Fisher's exact test and Bonferroni correction for multiple testing algorithm. Further they were filtered with p ≤ 0.05 and minimum three proteins per function to obtain significant functional annotations and pathways [21].

### Exploration of drug-target interactions

Total drug protein interactions were retrieved from the MATADOR database [22]. The drugs targeting the proteins of the interactions map were extracted from this resource. Further, the type of drug-target protein interactions was interpreted from the results.

## Results and Discussion

### Differentially regulated genes

The distribution of log2 fold change values relative to the mean of DESeq2 normalized counts can be visualized in the Supplementary Fig. 1. It reveals that there were several genes which were significantly expressed in different organ systems. The total number of significant up or down-regulated genes in all the organ systems was found to be 8,326. Of these, 3,111 genes were differentially regulated in more than one organ system. A list of differentially regulated genes of all the organ systems with their log2 fold change values and the significant p-value is shown in the Supplementary Table 1. It was observed from the table that the number of differentially regulated genes was the highest in liver and the least in fat; therefore, the pandemic could be severe in patients associated with liver and fat related diseases. Further, the table revealed several genes that were commonly regulated in multiple organ systems such as *IGF2*, *ITM2C*, MAPT, and *PPP1R1A* genes which were up-regulated in bowel, heart, jejunum, kidney and lung and *ABCA3*, *SFTPA1*, *SFTPA2*, *SFTPB*, and *SLC34A2* genes which were down-regulated in all the organ systems except lung. Some genes were observed to be differently regulated such as *ANK2* and *CLU* both of which were up-regulated in bowel and jejunum but down-regulated in marrow and placenta. A heatmap of organ-wise averaged read counts of genes differentially regulated in more than seven organ systems can be visualized in the Supplementary Fig. 2. It shows that the gene expression counts of lung were the most contradictory to the control samples suggesting that the lungs were severely affected than the other organ systems in the COVID-19 infection. ACE2, the angiotensin I converting enzyme 2, was observed to be up-regulated only in heart. CD147 (BSG), a transmembrane protein of the Ig superfamily, was observed to be up-regulated both in the heart and the marrow while TMPRSS2, a transmembrane protein of serine protease family was significantly down-regulated in bowel, heart, jejunum, marrow, placenta and skin. CD26 (DPP4), a functional receptor on lymphocytes, was observed to be up-regulated in placenta but down-regulated in jejunum. Therefore, differential regulation of the genes and receptors might lead to morbidity and severity of the pandemic disease.

### Protein interactions map

**Fig. 1.**
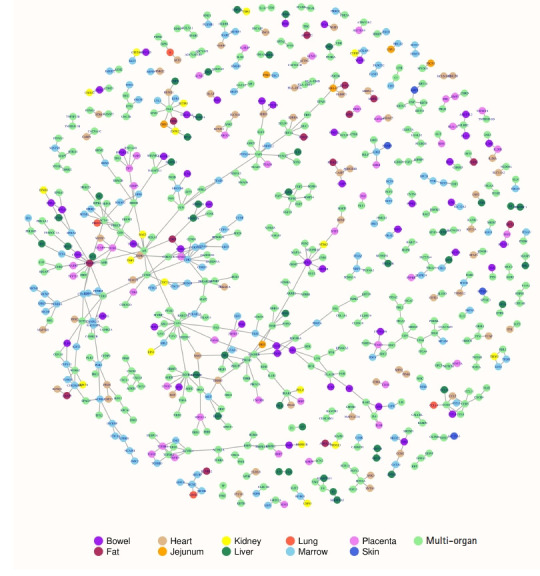
A multi-organ protein interactions map of the coronavirus disease 2019. Circles are nodes representing the proteins and the lines between them are edges representing the interactions. Organ-specific nodes are colored uniquely while the multi-organ proteins are colored light-green. Cross-organ protein interactions are interpreted by the interaction between differently colored nodes.

The DIP database constituted 6,729 experimentally verified human PPIs. The significant up or down-regulated gene products were detected to engage in 608 PPIs. Of these PPIs, two were specifically observed in bowel, four in heart, five in liver, 36 in marrow, and five in placenta. One hundred and ten PPIs were observed as cross-organ protein interactions where a protein of a specific organ system interacts with a protein of another organ system. Four hundred and forty-six PPIs were observed as multi-organ protein interactions where proteins of multiple organ systems interact with proteins of other organ systems. The involvement of large number of multi-organ protein interactions suggests that the COVID-19 pandemic affects several organ systems to reach its severe pathological state; therefore, early diagnosis and treatment of the pandemic could prevent patient decompensation and thus make easy recovery. A protein interaction map can be visualized in the Fig. 1. In the protein interactions map, the organ-specific, cross-organ and the multi-organ protein interactions were easily distinguishable using color representations of the nodes viz., purple, maroon, burly-wood, orange, yellow, sea-green, tomato, sky-blue, violet, royal-blue and light-green corresponding to bowel, fat, heart, jejunum, kidney, liver, lung, marrow, placenta, skin and multi-organ systems respectively. The map constitutes 608 edges or interactions among 672 nodes or proteins. The number of isolated interactions in the protein interactions map was 77 and the number of connected components was 54. Transitivity or clustering coefficient of the entire interactions map was found to be 0.094 revealing good local connections and sparse sub-graphs. Fitting power-law distribution suggested that the map is a discrete graph. Degree representing the number of interactions for a node was the highest for CAV1 with a value of 15 followed by CTNNB1 with a value of 13. The organ-wise highest degree nodes were HTR2A for bowel, CTNNB1 for fat, ERBB3 for heart, EIF4A1 for jejunum, CDC27 for kidney, ESR1 for liver, S and GINS3 for lung, CDK1 for marrow, DDB1 and TGFBR1 for placenta and KRT5 for skin. Removal of these high degree nodes would disrupt the protein interactions map significantly [23]. The degree distributions of the interactions map indicated that the node degree was decreasing with increase in the number of nodes suggesting a scale free interactions network. It can be viewed in the protein interactions map that ACE2 of heart interact with S protein of lung both of which were found to be up-regulating gene products. The S protein also interact with DPP4 suggesting different downstream regulations. The DPP4 in turn interact with PTPRC which was found to be down-regulating gene product in bowel, heart, jejunum, kidney and placenta. Thus, the map of experimentally validated protein interactions brought about several prospects for the researchers and scientists to investigate in the COVID-19 research.

### Functional annotations and pathways

The largest connected component of the protein interactions map was interpreted as the core interactions sub-network and it constituted 306 edges among 265 nodes. The core interactions sub-network can be visualized in the Supplementary Fig. 3. It was observed that the top 5 highest degree nodes contained in the core interactions sub-network implying of high functional significance. The edge betweenness clustering of the core interactions sub-network produced 18 modules or clusters. Modularity of these clusters was 0.84 suggesting good clustering and the significant modular structure [24]. Functional annotation and pathways of each of these modules and of non-core proteins is provided in the Supplementary Table 2. It is perceivable from the table that each of the modules have proteins significantly enriched in similar gene ontology terms such as biological process, molecular functions, cellular components and pathways. The largest modules (cluster 2, 6, and 14) were observed to contain mostly the membrane proteins with various cell binding and signaling activities. Further, the top functional annotations and pathways of each of the cluster can be viewed in Supplementary Fig. 4. The modular structure of the core interactions sub-network is represented in the Supplementary Fig. 5. The figure clearly depicts that 36 proteins bridges different modules with 29 interconnections. AR protein bridges six different modules, CTNNB1 5, CAV1 and PIK3R1 4, CCND1 and CTNNA1 3 and CDH1, CDK1 and DDB1 bridges two different modules while 27 other proteins bridge with at least one different module demonstrating their vitality for the flow of information across several pathways.

### Drug-target interactions

The number of interacting proteins mapped to MATADOR databases was 222. This indicates that the protein interactions map of COVID-19 is enriched with several significant targets with known drug candidates. Therefore, proteins of the interactions map can be further investigated for drug re-purposing strategies. The Supplementary Table 3 lists all these proteins with the sight of significant regulation, core or non-core interaction, degree, drug, MATADOR score and the type of drug-target interaction. To highlight some of the proteins, a list of drug candidates is shown in the following Table 1. CTNNB1, AR, EGFR, HTR2A, ESR1, INSR, JUN, and PDGFRB are the core and high degree nodes which could be investigating for interventions of the COVID-19 disease. Further, the experimental studies [25] showed that the interacting proteins of this study were targeted by the SARS-CoV-2 spike and other proteins. Therefore, the present study would facilitate and support the scientists and researchers to empathize the complex molecular mechanisms involving multiple organ systems associated with the COVID-19 pandemic.

## Figures and Tables

**Table 1.** List of high degree nodes and the drug candidates

Target	Organ system	Interaction	Drug
CTNNB1	Fat	Indirect	Sulindac
AR	Multi-organ	Direct	Aclarubicin, adapalene, aripiprazole, bezafibrate, carbamazepine, carteolol, eprosartan, isocarboxazid, losartan, nordihydroguaiaretic acid, pargyline, tazarotene, telmisartan, troglitazone, valsartan, warfarin
EGFR	Multi-organ	Direct	Gefitinib
HTR2A	Bowel	Direct	Aripiprazole, clozapine, metergoline, mianserin, olanzapine, quetiapine, risperidone, sertindole, zotepine
ESR1	Liver	Direct	Fulvestrant, tamoxifen, raloxifene, phenol red, estrogen, diethylstilbestrol, clomiphene citrate
INSR	Multi-organ	Direct	Metformin
JUN	Multi-organ	Direct	Nordihydroguaiaretic acid
PDGFRB	Multi-organ	Direct	Imatinib

## Supplementary Materials

Supplementary Table. 1. List of significant differentially regulated genes of all the organ systems.

Supplementary Table. 2. Functional annotations and pathways of protein modules.

Supplementary Table. 3. List of significant proteins and the drug candidates.

Supplementary Fig. 1. MA-plot of the DESeq2 processed data. The mean of normalized counts is represented on the X-axis while the log fold change values on the y-axis.

Supplementary Fig. 2. A heatmap of organ-wise averaged read counts of genes differentially regulated in more than seven organ systems. Light yellow color represents the maximum value, light yellow the mini-mum and light-green color in between maximum and minimum values.

Supplementary Fig. 3. A map of core interactions sub-network.

Supplementary Fig. 4. A graph of top functional annotations and pathways of genes of different modules.

Supplementary Fig. 5. A map of modules in the core interactions sub-network.
