# Supplementary material for: A protein interactions map of multiple organ systems associated with COVID-19 disease
Source: Genomics Inform. 2021 Jun 30;19(2):e14. doi: 10.5808/gi.20078 (PMC8261268; doi:10.5808/gi.20078)
Supplement: Supplementary Table. 1. — List of significant differentially regulated genes of all the organ systems. [file gi-20078-suppl1.pdf]

Genes	Bowel	Fat	Heart	Jejunum	Kidney	Liver	Lung	Marrow	Placenta	Skin
5_8S_rRNA	0	0	0	0	0	2.79948768116296 (0.00787044621704567)	0	0	0	0
7SK	0	5.84940858059088 (9.36188742177197e-05)	0	0	0	0	0	0	5.46164680638995 (5.11583022639577e-05)	0
A1BG	0	0	0	0	0	6.26387336201017 (6.61343768021066e-07)	0	0	0	0
A1CF	0	0	0	0	0	9.51208724750444 (3.12763371822287e-12)	0	0	0	0
A2M	-1.85563051372984 (1.36612811269233e-08)	-3.31930771390564 (1.46003988357464e-07)	-1.55748293235697 (0.000174925627768757)	0	0	-2.00629778680339 (0.00301732035573054)	0	-6.76108166792497 (8.670103135888e-25)	-2.25234186898749 (7.99947771575984e-09)	-2.24168522282029 (4.81250738745832e-05)
A2ML1	0	0	0	0	0	0	0	0	7.12557473183964 (2.29085518738272e-05)	9.54500464551145 (1.04509683134262e-12)
A4GALT	0	0	1.99509615065628 (0.000297995855720685)	0	0	0	0	0	0	0
AADAC	0	0	0	0	0	7.99756860467392 (8.85057063065081e-08)	0	0	0	0
AAED1	0	0	0	0	0	0	0	-5.81321865128799 (0.00965899149887187)	0	0
AARD	5.41134842547872 (3.50540107103988e-05)	0	0	0	0	0	0	0	0	0
AATK	0	0	0	0	0	0	0	0	-2.16937826152985 (0.00510559230171386)	0
ABAT	0	0	0	0	0	5.17306971730826 (3.2878356198297e-09)	0	0	0	0
ABBA01017803.1	0	0	0	0	0	4.11988714629635 (0.00420616732975775)	0	0	0	0
ABCA1	0	-6.08325282388224 (0.00544831852718987)	-2.43958749441384 (3.63547884585765e-06)	0	-2.10168159960644 (0.00283639412606696)	0	0	-2.82579558522349 (0.00105732833305445)	0	0
ABCA10	0	0	2.44796775179574 (2.87610960207541e-05)	0	0	0	0	0	0	0
ABCA12	0	0	0	0	0	0	0	0	2.85457630632056 (0.00369337184828844)	6.92107487358604 (3.80371809164092e-11)
ABCA13	-4.04200111455009 (2.82107397788894e-05)	0	0	0	0	0	0	5.13432939069355 (3.42741216321039e-08)	-5.32108774304178 (6.86578585292052e-08)	0
ABCA17P	0	0	0	0	0	0	5.30024812225292 (0.00259020923066966)	0	0	0
ABCA3	-3.51716567388322 (1.20819773336959e-15)	-5.66531133173315 (0.0087497335274507)	-3.42757939611464 (1.78407935661864e-11)	-3.62777148791234 (3.38853367295676e-06)	-2.87979205830059 (9.29465809404703e-06)	-2.95782093591694 (0.000218717604742604)	0	-5.65826253120301 (5.57732163398202e-06)	-5.56705646531032 (1.59798109846057e-37)	-4.82615774732285 (1.3457864765268e-08)
ABCA4	0	0	0	0	5.8833159939322 (0.000346002584637765)	0	0	0	0	0
ABCA6	0	0	0	0	0	0	0	0	-2.39470233534062 (4.69242898661951e-06)	0
ABCA7	-2.46524547276151 (0.000900901069458429)	0	0	0	0	0	0	3.09353145685276 (0.00124819243126714)	-1.78031434512434 (0.00859210131328342)	0
ABCA8	0	0	1.95431764132702 (0.00308353463940344)	0	0	0	0	0	0	0
ABCA9	1.51471571695231 (0.00252374400990806)	0	1.82627546025021 (0.00307790896412217)	0	0	0	0	-5.96629380400429 (0.00868758301128721)	-2.95211781193033 (6.15401466742008e-05)	2.26451583422987 (0.00967181434513373)
ABCB1	0	0	0	0	0	4.19972398529619 (1.55602709312758e-05)	1.89209490660107 (0.00638217895264329)	0	2.33577604013785 (0.00734026863489142)	0
ABCB10	0	0	0	0	0	0	0	3.33950822539712 (6.17071050089725e-05)	0	0
ABCB11	0	0	0	0	0	7.81427132926229 (2.94559765256729e-05)	0	0	0	0
ABCB4	0	0	5.20393187635589 (0.000113135020871401)	0	0	8.96183925060238 (4.30488647805218e-10)	5.50481997017102 (0.00126038001615961)	0	0	0
ABCC11	0	0	0	0	0	6.13308424872926 (0.000205072725404856)	0	0	0	0
ABCC12	0	0	0	0	0	8.04162219239548 (0.0012892446034553)	0	0	0	0
ABCC2	0	0	0	0	0	6.53673599028625 (6.61793762355684e-13)	0	0	0	0
ABCC3	-2.70580222785514 (6.73543567432476e-05)	0	-3.4528377943732 (7.43293920902473e-08)	-8.37204799994332 (0.000127230621894206)	0	1.86613354028819 (0.000838689230435816)	0	0	0	0
ABCC4	0	0	0	0	0	-2.49615796662738 (0.00139089836007313)	0	0	-2.78179899032643 (1.02133036858472e-10)	0
ABCC6	-3.4515330277223 (0.000412851911894807)	0	0	0	0	0	0	0	-5.48935757795376 (4.07877972190391e-07)	-6.39774009256637 (0.00747935237810106)
ABCC8	0	0	0	0	0	0	0	0	-4.08222520515326 (0.000761016260259416)	0
ABCC9	2.71440512000238 (1.59230888699834e-16)	0	1.74858406283336 (9.27504020229783e-05)	2.56490659490259 (7.79638070368323e-05)	0	0	0	0	0	1.74844767805714 (0.00887349495722286)
ABCG1	-1.77792630114686 (0.000124244904782296)	0	0	0	0	0	0	-3.58793351458482 (0.0060378570308391)	0	0
ABCG2	0	0	0	0	0	3.15159791593321 (0.00197604447651009)	0	0	4.07145441927126 (4.15012910444875e-08)	0
ABCG4	0	0	0	0	5.0602426192642 (0.00646359408462247)	6.37550090157665 (4.21535228305766e-05)	0	0	0	0
ABCG5	0	0	0	0	0	6.80361138704753 (5.91593926435536e-05)	0	0	0	0
ABCG8	0	0	0	0	0	6.57513391897989 (1.7344946878661e-05)	0	0	0	0
ABHD12B	0	0	0	0	0	0	0	0	0	5.33488079865238 (0.00480459195090949)
ABHD17A	0	0	0	0	0	0	0	0	-1.58674727127791 (0.000481902484447438)	0
ABHD17C	-2.00363952143944 (0.000648388276463492)	0	-2.92865150385175 (7.54998177750488e-05)	-6.47386129831695 (0.00366809860217044)	0	0	0	0	0	0
ABHD2	0	0	0	0	0	0	0	0	-1.897999820065 (2.79132711333046e-10)	0
ABHD5	0	0	0	0	0	0	0	2.40455456694977 (0.00249680677866365)	0	0
ABI3BP	0	0	0	0	-1.94437070537366 (0.00111666486276798)	-2.47225512365217 (6.24695470193193e-07)	0	-6.6694189859059 (3.11460943744407e-05)	0	0
ABL1	1.5067501826487 (8.58119212763657e-05)	0	0	2.1886957826388 (0.00235036834368558)	0	0	0	0	0	0
ABL2	0	0	0	0	0	0	0	-2.26594043446862 (0.00565538128430257)	0	0
ABLIM1	0	0	2.66633286207481 (1.41918088014739e-13)	0	0	0	0	-2.35952030633176 (0.000717751072385531)	0	1.80670249239584 (0.00136466134812987)
ABLIM2	0	0	0	0	2.8000677962191 (0.00277132869136759)	0	0	0	0	0
ABLIM3	0	0	1.89919168545242 (5.62949488737214e-07)	0	0	0	0	0	0	0
ABO	0	0	0	0	0	0	0	0	-2.62300674729824 (5.84947063784223e-06)	0
ABRA	0	0	7.9699833443861 (1.33231546626303e-08)	0	0	0	0	0	0	0
ABRACL	-2.46022517043031 (0.00182846444865427)	0	0	0	0	0	0	0	0	0
ABTB1	0	0	1.50905472226743 (0.00323150097496018)	0	0	0	0	0	0	0
ABTB2	0	4.16006352218452 (0.00292256296013667)	2.68072825413001 (6.66087130091133e-05)	0	0	2.79291050539066 (0.00406858531565775)	0	0	1.82678800280274 (0.0042581173614827)	0
AC003958.2	0	0	0	0	0	6.35429202637639 (0.00819894180732007)	0	0	0	0
AC003984.1	0	0	0	0	0	5.92383765599611 (0.00893443873901692)	0	0	4.03710230652508 (0.00971613101156483)	0
AC004019.13	0	0	0	0	0	0	3.40164121914514 (0.00863357939867156)	0	0	0
AC004156.3	0	0	0	0	6.33185381649403 (0.000865491730202107)	6.02166962498863 (0.00924834391122143)	0	0	0	0
AC004158.3	0	0	0	0	0	6.76659816327141 (0.000214866601419842)	0	0	0	0
AC004540.5	0	0	0	0	3.89649446313133 (0.00808286857364205)	0	0	0	0	0
AC004603.4	0	0	0	0	0	0	0	0	4.56227415099541 (0.00530854654535494)	0
AC004893.11	0	0	0	0	0	2.67257160355299 (0.00679909404193703)	0	0	0	0
AC005082.12	0	0	0	0	5.49828842844653 (0.00841201059266549)	0	0	0	0	0
AC005105.2	0	0	4.63419500047703 (0.00115882308976882)	0	0	0	0	0	0	0
AC005235.1	0	0	0	0	0	7.33249069172871 (0.00477557364721531)	0	0	0	0
AC005264.2	-3.99019707045519 (0.00412254251099493)	0	0	0	0	0	0	0	-3.19130809444691 (0.00330682129778002)	0
AC005281.2	0	0	0	0	0	0	0	0	4.60012430167337 (0.000810097076726662)	0
AC005330.2	0	0	0	0	6.65447770053204 (0.00254565647500638)	0	0	0	0	0
AC005481.5	0	0	0	0	0	5.64681297834411 (0.0020684447686034)	0	0	0	0
AC005532.5	0	0	0	0	0	0	0	0	4.39409974843157 (0.00025388428330316)	0
AC006019.3	0	0	0	6.09359286470464 (0.00640596319862823)	0	0	0	0	0	0
AC006019.4	6.043879191613 (2.09548857848092e-06)	0	0	6.55623430861536 (0.00721277329296838)	0	0	0	0	0	0

AC006486.10	0	0	0	0	4.53409685034146 (0.00957854386889111)	0	0	0	0	0
AC006548.28	0	0	0	0	7.76445988971595 (1.36744446103144e-07)	9.23198736098005 (3.531995317747e-10)	6.1686090447672 (0.000488126904604376)	0	0	0
AC006978.6	0	0	0	0	0	0	0	0	1.97080569412933 (0.000663810602857885)	0
AC007040.11	0	0	0	0	4.46986578765956 (0.0068592631663985)	4.92835763315765 (0.00715319093661951)	0	0	0	0
AC007099.1	0	0	0	0	0	0	0	0	4.1286119975179 (0.000334052426163914)	0
AC007256.5	0	0	0	0	0	0	0	3.96203578483356 (0.00503004395508514)	0	0
AC007278.2	-4.28825330080045 (0.000398528811162577)	0	-3.64643514016262 (0.00271322061169872)	0	0	0	0	0	-3.73889234997596 (4.43362639086561e-05)	0
AC007278.3	-3.23076787948752 (0.000121263275216621)	0	-5.01009611812952 (1.38055117008664e-07)	-5.50353650136261 (0.0013639753981034)	-4.05708395818395 (0.00236231372429314)	0	0	0	-4.38523365656287 (5.83745054894315e-09)	-4.78232965072469 (0.000538940422424284)
AC007292.3	0	0	0	0	0	4.98366661660587 (0.00405521418456342)	0	0	0	0
AC007319.1	0	0	-3.15612479149173 (0.000969175013949857)	0	0	0	0	0	0	0
AC007326.10	0	0	0	0	7.08014610211939 (0.000681244886600786)	7.32624266357827 (0.00538433295435045)	0	0	0	0
AC007386.2	0	0	0	0	5.59641445460015 (0.00941447804126436)	0	0	0	0	0
AC007743.1	0	0	0	0	0	0	0	0	3.2348151225516 (0.000166848397805198)	0
AC007750.5	0	0	0	0	0	6.03255896650603 (0.00959365730383889)	0	0	0	0
AC007952.5	0	0	0	0	5.94307778723946 (4.58727572825803e-06)	0	0	0	0	0
AC008079.10	0	0	0	0	4.0206353684853 (0.000396397700682116)	4.53403513293566 (0.000366001070707774)	0	0	0	0
AC009133.12	-3.15577658665698 (0.000135024278824134)	0	-3.46728738558569 (0.000219542333825617)	0	0	0	0	0	-3.3980967883149 (3.0303393592893e-06)	0
AC009166.7	0	0	0	0	0	6.5704967386687 (0.00392614356087615)	0	0	0	0
AC009264.1	5.71304071341163 (2.9889465313784e-09)	0	0	0	0	0	0	0	0	0
AC009501.4	0	0	0	0	0	4.77514899367404 (0.000638472869108957)	0	0	0	0
AC009948.5	0	0	0	0	0	0	0	0	1.60253141509813 (0.00158856182588225)	0
AC009950.2	0	0	0	0	3.88968849695565 (0.00897576481926317)	0	0	0	0	0
AC010969.1	0	0	0	0	0	6.17825649751032 (0.00501893615801156)	0	0	0	0
AC010970.2	0	0	0	-3.39847029440113 (0.00977085884138285)	0	4.04716270418697 (1.06422252337761e-05)	0	0	-2.14361221150295 (0.00255000238986897)	0
AC010980.2	3.5255731352629 (0.00902992581303371)	0	0	0	0	0	0	0	0	0
AC011043.1	3.55549054013354 (0.00679609629742315)	0	0	0	0	0	0	0	3.34147241799171 (0.00301648091671578)	0
AC011343.1	0	0	0	0	0	6.16243294315138 (0.000369481619814904)	0	0	0	0
AC011738.4	0	0	0	0	0	6.38968800314438 (0.00475066945748178)	0	0	0	0
AC011899.9	0	0	0	0	0	3.98758561541224 (0.00126125149867358)	0	0	0	0
AC012314.8	0	0	0	0	0	3.31132472279832 (0.00670974799366896)	0	0	0	0
AC012360.6	0	0	3.21114034133978 (0.000248824223954407)	0	0	3.30493410563457 (0.00738295073405087)	0	0	2.87938575190413 (9.04773602448445e-05)	0
AC012531.25	4.96393238647135 (0.0015286503104213)	0	0	0	0	0	0	0	4.61896348189287 (0.000220143730823918)	0
AC013275.2	0	0	0	0	5.24659825842358 (0.000400070681663069)	0	0	0	0	0
AC013472.3	0	0	0	0	0	4.84978355240047 (0.00988519653149729)	0	0	0	0
AC015849.15	0	0	0	0	0	7.41661066314602 (0.000755620027344852)	5.13110118498003 (0.00401385630927465)	0	0	0
AC015849.16	0	0	0	0	0	5.81397077063224 (0.000338135472491085)	0	0	8.01720411924336 (2.16780146948268e-07)	0
AC016735.2	0	0	0	0	0	5.92383765599611 (0.00893443873901692)	0	0	4.52403283358523 (0.0043838854528167)	0
AC016738.4	0	0	0	0	0	5.17340908343992 (0.000585445088391916)	0	0	0	0
AC016907.3	0	0	0	0	0	6.10213009384402 (0.00755053791667713)	0	0	0	0
AC018442.1	0	0	0	0	0	4.34671248158743 (0.00164095136899591)	0	0	0	0
AC018464.3	0	0	4.45341516953971 (0.00454253807808971)	0	0	0	0	0	0	0
AC018804.7	0	0	0	0	0	6.15860567358818 (0.000577977338783464)	0	0	0	0
AC018890.6	0	0	0	0	0	0	0	0	3.18083385824668 (8.85343103939996e-05)	0
AC020571.3	0	0	0	0	0	0	0	0	5.18961594554025 (0.0033526588912815)	0
AC022007.5	0	0	0	0	3.76636481347507 (0.0095667952739386)	0	0	0	0	0
AC022819.3	0	0	0	0	0	3.69140632927635 (0.00419443471879637)	0	0	0	0
AC023115.2	0	0	0	0	0	6.41941017940674 (0.00484782295056037)	0	0	0	0
AC025165.8	0	0	0	0	3.70935316431112 (0.00794774719953086)	4.11830713739968 (0.00541947338302083)	0	0	0	0
AC053503.12	4.12391201986888 (0.000634475834543841)	0	5.43837791222658 (2.51888428281332e-05)	5.78187607635437 (0.00101315303686263)	0	0	0	0	0	0
AC053503.6	0	0	5.12189108233917 (0.000231821594040208)	0	0	0	0	0	0	0
AC063976.7	0	0	0	0	0	3.62473292718674 (0.00704625869307006)	0	0	0	0
AC068580.6	0	0	-3.22806974489374 (8.47322715455085e-05)	-5.24265398791486 (0.00398738747857889)	0	0	-2.19443213879936 (0.000179500747701609)	0	-5.78696113101351 (4.76593133886633e-11)	0
AC068831.10	0	0	0	0	0	6.89844137680823 (0.00146210936952516)	0	0	0	0
AC069213.1	0	0	0	0	0	5.19422886916845 (0.00895787691661891)	0	0	0	0
AC073046.1	4.71653936066979 (1.38246824185868e-07)	0	0	0	0	0	0	0	0	0
AC073109.2	0	0	0	0	0	0	0	0	3.90477303269959 (0.000132359413880944)	0
AC079466.1	0	0	0	0	0	7.37291534030357 (0.00497391000732472)	0	0	0	0
AC079612.1	0	0	0	0	0	7.75537006631976 (0.000269423952665357)	0	0	0	0
AC079630.4	-4.61624910641463 (0.00133580036698466)	0	0	0	0	0	0	0	-5.39953842829821 (3.27125433060698e-05)	0
AC079779.6	0	0	0	0	0	6.10213009384402 (0.00755053791667713)	0	0	0	0
AC079807.4	0	0	0	0	0	6.9080154569599 (0.000102118549581005)	0	0	0	0
AC079949.1	0	0	0	0	3.44504878001817 (0.00883361285858704)	3.59469543639797 (0.00618299362051013)	0	0	-3.65145503570881 (0.00531510548462867)	0
AC083843.1	0	0	0	0	0	0	0	0	1.90648745584183 (0.00231169765578565)	0
AC083884.8	0	0	0	0	0	0	0	0	2.57141083626527 (5.83968902426999e-07)	0
AC090505.6	0	0	0	0	0	5.92320077315633 (0.00187738139348567)	0	0	0	0
AC091492.2	-4.44959125885762 (0.00492091091577824)	0	0	0	0	0	0	0	0	0
AC092066.1	-2.59026122414344 (0.00217429141466159)	0	0	0	0	0	0	0	-2.0633849648378 (0.00308767498985428)	0
AC092071.1	-5.6633552711319 (2.83070517514289e-05)	0	-3.94247928849767 (0.00180866317904069)	0	0	0	0	0	-5.71194913212902 (7.81400764622247e-07)	0
AC092162.1	0	0	0	0	0	7.0371540031009 (0.00213789794773112)	0	0	0	0
AC092171.4	0	0	0	0	0	0	0	0	-2.84828559118353 (0.00550343983132869)	0
AC092535.3	0	0	0	0	5.96639391414183 (0.000530373312695535)	0	0	0	0	0
AC092667.2	0	0	0	0	0	6.35947680596301 (0.00421653283516686)	0	0	0	0
AC092687.5	0	7.66282511003579 (0.000226746650776502)	0	0	0	0	0	0	0	0
AC092835.2	0	0	0	0	0	3.66637246696162 (0.00263297564141027)	0	0	0	0
AC092881.1	0	0	-2.92829536193395 (0.000307677531762083)	0	0	0	0	0	0	0
AC093616.4	-3.76456766343378	0	0	0	0	0	0	0	0	0

	(0.0095139752599924)									
AC093642.3	0	0	0	0	0	0	0	0	3.21234202284148 (0.000303611314335595)	0
AC093802.1	0	0	0	0	0	7.29646960103843 (0.000960664264781237)	0	0	0	0
AC096574.5	0	0	0	0	0	6.37208004457718 (0.00601138540544141)	0	0	0	0
AC097468.4	0	0	0	0	7.30573886964219 (0.000208366323090074)	0	0	0	0	0
AC100802.3	0	0	0	0	0	0	0	0	4.36889916833974 (0.00750477632694174)	0
AC104389.1	0	0	0	0	0	0	0	0	3.95537414589328 (0.00985800800507607)	0
AC104777.2	0	0	0	0	0	6.38968800314438 (0.00475066945748178)	0	0	0	0
AC104809.3	0	0	0	0	0	0	0	0	-4.76972177441784 (0.000964171306843884)	0
AC105342.1	0	0	0	0	0	7.96748171657473 (0.000167552957910135)	0	0	0	0
AC108142.1	0	0	0	0	0	0	0	0	3.41336037348849 (0.00170932702364509)	0
AC108938.5	0	0	0	0	0	0	0	0	3.88860872572307 (0.000358726104555834)	0
AC109642.1	0	0	0	0	0	0	0	0	-5.35046141422163 (0.000160042959853869)	0
AC113618.1	0	0	0	0	0	6.98175140929643 (0.00827365846225426)	0	0	0	0
AC114776.3	0	0	0	0	0	7.5571521687225 (3.60773252236403e-05)	0	0	0	0
AC136352.6	0	0	0	0	5.58716762543879 (0.00208114252067917)	0	0	0	0	0
AC139099.7	0	0	0	0	6.01984552865545 (0.0021925020044347)	0	0	0	0	0
AC139712.2	0	0	0	0	0	7.05645578158446 (0.00152284467383944)	0	0	0	0
AC226118.1	0	0	0	0	7.13500379894683 (3.0976464632767e-05)	7.97454113664986 (0.00889443746813182)	0	0	0	0
AC233755.1	0	0	0	0	0	0	3.41450198065545 (0.00372731579019539)	0	0	0
AC240274.1	0	0	0	0	0	0	0	0	1.564311908947 (0.00324538094712862)	0
AC253572.1	0	0	0	0	0	0	0	0	-3.05866929805155 (0.00971075454135433)	0
ACAA1	0	0	0	0	0	2.12557530924961 (9.09146564029045e-05)	0	0	0	0
ACACB	1.90310064563164 (0.000248932417699154)	0	3.29436153331875 (1.04401482960294e-14)	0	2.15084682966102 (0.0010703580018115)	2.53809562333041 (4.09005119775259e-05)	0	0	0	3.41172998135212 (9.6723045655072e-05)
ACADL	-5.82255477859301 (5.43847469560641e-06)	0	-2.74559223219866 (0.00491522972610097)	0	0	0	0	0	0	0
ACADM	0	0	1.9469595555431 (8.6742051331186e-05)	0	0	0	0	0	0	0
ACADS	0	0	2.72111463091803 (1.33213281556891e-06)	0	0	3.29956979753777 (5.32086796613162e-08)	0	0	0	0
ACADSB	0	0	0	0	0	2.11413209259554 (0.00225581992046072)	0	0	-1.74908848914561 (0.000203751809181344)	0
ACADVL	0	0	1.67512579727762 (3.60914410710384e-08)	0	0	0	0	0	0	0
ACAP1	-2.3333257088984 (0.00130840357611538)	0	0	0	0	0	0	2.6100628601391 (0.00806462124175586)	0	0
ACAP2	0	0	-1.65790959035719 (0.000124046069339396)	0	0	-1.55381267763372 (0.00179707187244767)	0	0	0	0
ACAT1	0	0	1.85696952151402 (7.05171789319496e-05)	0	0	3.57617694498727 (1.64665233092602e-05)	0	0	0	0
ACBD4	0	0	0	0	0	2.28139832267214 (0.00169077552332336)	0	0	0	0
ACE	-2.8705453532647 (0.000552317393237122)	0	0	0	0	0	0	0	0	0
ACE2	0	0	3.28423389464978 (0.000298199000248087)	0	0	0	0	0	0	0
ACER2	0	0	0	0	0	0	0	0	3.16403513941221 (8.05971978416116e-08)	0
ACER3	0	0	-1.93909383465206 (0.000301423863196204)	0	0	0	0	0	0	0
ACHE	0	0	0	0	0	0	0	3.2914356812571 (0.00811225108905968)	0	0
ACKR1	3.45135253620508 (4.32620904782983e-05)	0	0	0	0	0	2.7974687060675 (0.000341059248423406)	0	0	0
ACKR2	0	0	0	0	0	3.48769779232894 (0.000313015388918077)	0	0	5.45281056948501 (7.3619029223424e-24)	0
ACKR3	0	4.4489005747022 (3.60316604715064e-05)	0	0	0	3.50873342569854 (6.005984777561e-05)	0	0	2.18263892347901 (1.51440381105161e-06)	3.64501101711585 (8.30641156948296e-05)
ACMSD	0	0	0	0	0	6.45572491686146 (0.000333756181001224)	0	0	0	0
ACO2	0	0	3.36661821796431 (4.63447086612332e-15)	0	0	0	0	0	0	0
ACOT1	0	0	0	0	0	5.12982750344221 (0.000806878531091988)	0	0	0	0
ACOT11	2.56563596763207 (5.11096788824605e-06)	0	2.40403903616781 (0.000449787641479043)	0	0	0	0	0	0	0
ACOT12	0	0	0	0	0	6.2919247941663 (0.00429897876173186)	0	0	0	0
ACOX1	0	0	0	0	0	1.61291719835552 (0.000818855070306047)	0	0	0	0
ACOX2	0	0	0	0	0	2.5028914787338 (0.00426919208911981)	0	0	0	0
ACOXL	-4.70259714154642 (0.000589634903311017)	0	0	0	0	0	0	0	0	0
ACP5	-3.24247801424677 (7.55779214193651e-06)	0	-5.12303261892466 (9.53292683529632e-08)	-4.32012372377049 (0.00320761162566113)	0	0	0	0	-2.78799913551291 (0.000302034771366571)	-4.01699236045477 (0.00276085302722612)
ACSBG1	0	0	0	0	0	0	0	5.92911697185018 (0.000105225579780822)	0	0
ACSL1	-1.57742839501447 (0.00212094677494257)	0	0	0	0	0	0	0	-2.37099606145306 (1.47895498861817e-08)	0
ACSL3	0	0	0	0	0	0	0	-2.91563952537999 (0.0010130744658166)	0	0
ACSL4	0	0	0	0	0	0	0	-2.26177317623189 (0.0083522620309486)	0	0
ACSL5	0	0	0	0	0	0	0	0	-1.55807829774271 (0.000421316026077025)	0
ACSL6	0	0	0	0	0	5.14209370045885 (0.00103880537408706)	3.69459980177444 (0.00384227868695959)	0	0	0
ACSM2A	0	0	0	0	7.46633282986189 (3.23750915187574e-06)	9.79182586261365 (2.12639294646279e-15)	6.69622699285988 (0.00872924748536941)	0	0	0
ACSM2B	0	0	0	0	6.91800884378814 (0.00837990899630342)	9.31031263764188 (1.29485520614081e-13)	0	0	0	0
ACSM3	1.91909079474928 (0.00136581645851087)	0	0	0	0	2.83936551978392 (0.000402119181941985)	0	3.43570207138918 (0.00159230797878227)	0	0
ACSM5	0	0	0	0	0	4.26102544308279 (0.000822743049844379)	0	0	0	0
ACSS1	0	0	0	0	0	0	0	0	2.32844706009242 (0.00128005063542543)	0
ACSS3	0	0	0	-4.68104503959844 (0.00484629278047281)	0	0	0	0	-2.0387417177788 (0.000602744607783382)	0
ACTA1	0	0	12.5876636986995 (3.71063101714194e-18)	0	0	0	0	0	3.75207632483431 (0.00399511576608659)	0
ACTA2	0	0	0	0	0	0	0	-9.20425874681352 (2.13660873517956e-05)	0	0
ACTA2-AS1	0	0	0	0	0	0	0	0	2.15106660864496 (8.84574900838398e-05)	0
ACTC1	0	0	12.9539049309705 (5.81784255655545e-33)	0	5.92020834064043 (0.000340987647979674)	5.97962335626233 (0.00808979935369055)	0	0	7.48843719794494 (9.08772459592608e-10)	0
ACTG1P1	0	0	0	0	0	5.62552789589499 (0.00459723176233694)	0	0	0	0
ACTG2	5.52024466370897 (9.64943055099789e-34)	0	0	5.77577297160417 (5.0499654374325e-15)	0	0	0	-7.44502518676573 (0.000728626911494626)	2.43121523724685 (3.30582469640317e-06)	0
ACTN1	1.54919609760632 (1.47459770413962e-07)	0	0	1.86696998096169 (0.00216200596556487)	0	0	0	0	0	0
ACTN2	0	0	8.97120356819432 (1.94363127794272e-44)	0	0	0	0	0	0	0
ACTR3	0	0	-1.54696331660443 (9.95901718990826e-05)	0	0	0	0	0	0	0
ACVR2A	1.784957346217 (0.0015197745270809)	0	0	0	0	0	0	0	1.72340373365897 (0.000100312152511483)	0
ACVR2B	0	0	0	0	0	0	0	0	1.6372829709976 (0.000137992751877076)	0
ACVRL1	-2.05146225915829 (3.56429002368651e-10)	0	0	-1.84665034268851 (0.00391728421546725)	0	-3.55859335338162 (2.44920374747518e-08)	0	-5.91694389255313 (0.000159631106041161)	0	0
ACY1	0	0	0	0	0	4.19662999267238 (0.00024011533316058)	0	0	0	0
ACYP2	0	0	2.37786695732908 (0.000954801591482947)	0	0	3.19717418551038 (0.000694315606270378)	0	0	0	0

ADAM11	0	0	5.16083393235891 (0.00195939754541249)	0	0	0	0	0	0	0
ADAM12	-2.24480826881336 (0.00660612507930193)	0	-2.40863491791733 (0.00218088342625606)	0	0	0	0	0	6.64823010214186 (2.29563372828042e-14)	0
ADAM15	0	0	0	-2.8454382313013 (0.00352973268761395)	0	0	0	0	0	0
ADAM17	0	0	0	0	-2.55825449752007 (0.000401466007477263)	-2.36746988560115 (4.38295843472365e-05)	0	0	0	0
ADAM21P1	0	0	0	0	0	6.61365949608464 (0.0032560484510769)	0	0	0	0
ADAM22	0	0	0	0	3.60652774020548 (0.000397946169592738)	0	0	0	0	0
ADAM23	1.83463841304714 (0.00958547385259447)	0	5.01198028768256 (5.83729078575022e-13)	0	0	0	0	0	0	0
ADAM28	0	0	0	0	0	0	1.7788184988634 (0.00788831251480525)	0	2.82533129446209 (0.000478103051126929)	0
ADAM29	0	0	0	0	6.82403350424883 (0.000166287887147743)	6.38968800314438 (0.00475066945748178)	0	0	0	0
ADAM33	2.81838087647816 (3.47711443548573e-08)	0	0	3.20167008959934 (0.000892073731409515)	0	0	0	0	0	0
ADAM8	-2.94996507768461 (0.000308495897814488)	0	-3.93687539957781 (0.000361227175822911)	0	0	0	0	0	0	0
ADAM9	0	0	-1.66244253834066 (4.48432856534648e-07)	0	-2.09381262786994 (4.72239528088693e-05)	-3.321819062124 (1.39289966701524e-09)	0	0	0	0
ADAMDEC1	6.69228719675493 (4.3568476667877e-07)	0	0	0	0	0	0	0	3.26183507512769 (0.00417824589741422)	0
ADAMTS1	0	0	0	0	0	0	0	-5.6163996504238 (8.38142340289164e-08)	0	0
ADAMTS12	-2.41765478643892 (0.000110927714707518)	0	0	0	0	0	0	-6.57340871614812 (0.00350104306528588)	0	0
ADAMTS13	0	0	0	0	0	3.36826862044917 (0.00142035780874152)	0	0	0	0
ADAMTS18	0	0	0	0	0	0	0	0	7.21002884741759 (0.00012067860031041)	0
ADAMTS19	0	0	0	0	0	0	0	0	4.46504946557031 (8.25652856828687e-06)	0
ADAMTS2	-2.46569926144279 (8.64773597250776e-08)	0	-2.03942023976294 (6.45278797349207e-05)	-2.7190599853366 (0.000577746357457365)	0	0	0	0	0	0
ADAMTS4	0	0	0	0	0	0	0	0	-3.12920279046376 (4.20165924719862e-06)	0
ADAMTS5	2.98439918215384 (8.41270249889887e-10)	0	0	3.00209314803103 (0.00284081594333008)	0	0	0	0	5.45997156840333 (9.76715139340543e-15)	4.37108611797404 (5.51802626703084e-07)
ADAMTS6	0	0	0	0	0	0	1.86810691854175 (0.00437960559129517)	0	5.00872577771318 (6.08479002069079e-14)	0
ADAMTS8	0	0	-3.83799717600808 (0.000103118819053073)	0	0	0	0	-6.48476932907396 (0.00590261870300589)	-4.70244764521167 (8.05774940504924e-09)	-5.38099959174318 (0.0034051046788368)
ADAMTSL1	2.44003034010761 (0.00132100243909475)	0	0	0	0	4.21186678165771 (0.00694214282238573)	3.33266051144997 (0.0025419068778749)	0	0	0
ADAMTSL2	-4.7268597723062 (5.2985306524497e-06)	0	0	0	0	0	0	0	-3.89430768648706 (8.28363705936539e-07)	-6.87214084231727 (0.00283074274341664)
ADAMTSL3	0	0	0	0	0	0	0	0	0	-4.24521039679272 (0.00141893407122296)
ADAMTSL4	0	0	0	0	0	0	0	-4.90758534795138 (0.00241803597635972)	1.52906800838184 (5.3688966561281e-05)	0
ADAMTSL5	0	0	4.0799304297573 (3.92009786553358e-06)	0	0	0	0	0	3.07487575019739 (0.00134281449718056)	0
ADAP1	0	0	-3.21267692260571 (0.00249387556300644)	0	0	0	0	0	0	0
ADCK1	0	0	0	0	0	0	0	3.3954366745429 (0.00783155915843337)	0	0
ADCK3	2.26122732966081 (1.67506386836258e-09)	0	3.90672283248224 (2.81970861326161e-20)	0	0	2.44981519875261 (1.84345252321839e-08)	0	0	0	0
ADCY1	0	0	3.07044565756775 (1.51623170839154e-05)	0	0	4.9120986141962 (1.78998490000346e-08)	0	0	0	0
ADCY2	3.31634654260653 (0.000126391230579797)	0	0	0	3.93570503818194 (0.00601462173846834)	4.60805245562565 (0.0029989377751963)	0	0	0	0
ADCY5	3.13264048473281 (1.58154160199102e-09)	0	4.62627737624016 (1.39405164631936e-17)	3.10509084318867 (0.00118156228481026)	2.89186833782028 (0.000558164882088395)	0	0	0	0	0
ADCY6	0	0	2.08906702975579 (1.28182308840797e-07)	0	0	0	0	0	0	0
ADCY8	0	0	0	0	0	4.87612464140995 (0.00664302333912846)	0	0	0	0
ADCY9	0	0	0	0	0	0	0	-3.86889949770121 (0.000683790418505527)	0	0
ADCYAP1	4.67773297696274 (0.00014476130061842)	0	0	0	0	5.70928192008291 (0.0048758615594079)	0	0	0	0
ADCYAP1R1	5.34821219122376 (2.3662525639846e-07)	0	4.24501642829699 (0.00010751090985126)	0	0	0	0	0	0	0
ADD2	0	0	0	0	0	0	0	5.76111580178672 (2.0783395695932e-10)	-2.78896993960904 (0.00639123181041811)	0
ADH1A	0	0	0	0	0	5.73489703260076 (0.000296979005954773)	0	0	0	0
ADH1B	0	0	0	0	0	0	0	-5.28303448697872 (2.82119721372285e-07)	-6.93428155629443 (2.29000055362652e-25)	0
ADH1C	0	0	0	0	0	4.77927387745109 (5.72737457698996e-05)	0	0	0	0
ADH4	0	0	0	0	0	8.84039269108319 (8.02494781237287e-08)	0	0	0	0
ADH5	1.68630440498316 (9.8788952913528e-07)	0	0	0	0	0	0	0	0	0
ADH6	0	0	0	0	0	5.32213814938168 (4.4989814832603e-06)	0	0	0	0
ADIPOQ	0	0	6.26874898605652 (2.07210496504339e-05)	0	0	0	0	0	0	8.89445205850559 (9.02556888134249e-10)
ADIRF-AS1	0	0	0	0	0	0	0	0	2.08856556954724 (0.00392189893857707)	0
ADM	0	0	-2.13838552489615 (0.000219501360983939)	0	0	0	0	0	0	1.65399098397485 (0.00729905192071514)
ADM2	0	6.0765561857187 (5.98643312421363e-05)	0	0	0	0	0	0	0	0
ADORA1	0	0	3.38604024665989 (0.00459926159250235)	0	4.78764093645835 (0.000637157047421033)	4.77179477222036 (0.00773238291032226)	3.10819030044462 (0.00744521837238696)	0	3.46314226923585 (0.000624785352230423)	0
ADORA3	0	0	-2.56541520654814 (0.00371818491343137)	0	0	0	0	0	0	0
ADPGK	-1.86969503969597 (1.6442133245812e-07)	0	-1.72371193769403 (3.87305058615339e-05)	-2.41421659932014 (0.00112010649920999)	0	0	0	0	0	0
ADPRHL1	0	0	10.0253819401262 (5.21898425969687e-21)	0	0	0	0	0	0	0
ADRA1A	0	0	0	0	0	3.1943658732836 (0.000210557466940909)	0	0	-4.15319930324991 (0.000329386965909008)	0
ADRA1B	0	0	0	0	0	5.22837146997531 (0.00021389122890298)	0	0	0	0
ADRA2A	0	5.07936692381954 (0.00103708704292736)	0	0	0	0	0	0	0	0
ADRA2B	0	0	4.61393146764757 (6.65562174600637e-05)	0	0	4.78644229496029 (0.00575637971430947)	0	0	0	0
ADRA2C	0	0	0	0	6.39429523353052 (4.54698805954294e-07)	0	0	0	4.91052930048168 (5.47410715890082e-05)	0
ADRB1	-4.11067871109099 (1.36788968852409e-05)	0	0	-6.80018730563642 (0.00369367993385117)	0	0	0	0	0	-7.07883394403569 (0.00248985184432637)
ADRBK1	0	2.16750852826289 (0.00678145167573839)	0	0	0	0	0	0	0	0
ADRBK2	0	0	-2.72856877094653 (1.34791296803721e-05)	0	0	0	0	0	0	0
ADSSL1	0	0	3.57086713084414 (3.83889765329842e-10)	0	0	3.84277409982311 (3.71628606945833e-05)	0	0	0	0
AE000661.37	0	0	0	0	0	5.95524930379388 (0.0028490376563024)	0	0	0	0
AEBP1	0	0	0	0	0	0	0	-3.88587743906168 (1.43965145449206e-06)	0	0
AEN	0	0	0	0	0	0	0	0	-1.79595618569921 (0.00134468647210224)	-3.3332564952881 (0.00966943827634705)
AF001548.3	3.62152215467603 (5.15826087434621e-07)	0	0	0	0	0	0	0	0	0
AF001548.4	4.10256306892012 (1.49125057087247e-06)	0	0	0	0	0	0	0	0	0
AF001548.5	3.60792679927969 (8.48971415235146e-08)	0	0	4.13475909642009 (0.00285790338626268)	0	0	0	0	0	0
AF001548.6	4.3379279900225 (5.91424274886208e-05)	0	0	0	0	0	0	0	0	0
AF064858.6	0	0	0	0	0	6.40144766710873 (1.16319165272767e-05)	0	0	0	0
AF121898.3	0	0	0	0	0	7.04063115215185 (0.00840552745288383)	0	0	0	0
AF127577.11	0	0	0	0	0	0	0	0	3.028992584933 (0.004127901363201)	0
AF127936.5	0	0	0	0	0	0	0	0	4.17802139101048 (0.000736786816263086)	0
AF127936.7	0	0	0	0	0	0	0	0	7.08140019036016 (8.98705667173480e-11)	0
AF127936.9	0	0	0	0	3.84273157299639	3.26413521141761	0	0	3.62121780772734	0

					(0.00312085601601986)	(0.00678526890026251)			(1.53912418312998e-06)	
AF131215.3	0	0	0	0	0	0	0	0	-2.14459588772428 (0.00460476175052829)	0
AF131217.1	0	0	0	0	0	0	0	0	2.94353883834534 (2.69295377867891e-08)	0
AFAP1L2	0	0	0	0	0	0	0	0	1.84276000448061 (0.000436046813693387)	0
AFF2	-2.88654496725047 (3.09113169397852e-05)	0	-2.48373674385059 (0.00153409598632278)	0	0	0	0	0	2.645591754578 (3.58812734334851e-06)	0
AFF3	0	0	0	0	0	0	0	0	0	-3.54007476921513 (0.00191266996724788)
AFF4	0	0	0	0	0	0	0	-1.87011233195218 (0.00259010262047267)	0	0
AFG3L2	0	0	1.51499541225379 (0.000603294780810848)	0	0	0	0	0	0	0
AFM	0	0	0	0	0	8.70617380222133 (4.66120658281187e-11)	0	0	0	0
AFMID	0	0	0	0	0	2.04360587377498 (0.0031270419653399)	0	0	0	0
AGAP2	0	0	0	0	0	0	0	3.12137391678383 (0.00231957847903745)	0	0
AGAP6	0	0	0	0	0	0	0	0	-1.87359960286766 (0.00235732876623371)	0
AGBL4	0	0	0	0	4.70579727478189 (0.00340605675612058)	4.91205205408623 (0.00289521356485572)	0	0	0	0
AGBL5	0	0	0	0	0	2.34174372504259 (0.00457770745052823)	0	0	0	0
AGER	-5.28720752665653 (1.08338582000826e-05)	0	-4.58512862527646 (7.29346278022317e-09)	-4.53499781104537 (0.000389174692794738)	-6.04000699319152 (5.70944213671036e-05)	-3.34387478291819 (0.00472401562431343)	0	0	-4.00674871293849 (6.57843428867544e-09)	-4.8130770617907 (0.000166839488866648)
AGFG1	0	0	0	0	-1.60976069221851 (0.00618736411103088)	0	0	0	0	0
AGFG2	0	0	0	0	2.99003222191823 (1.1330160582409e-07)	2.71669555707628 (6.21406741990407e-06)	0	0	0	0
AGMAT	0	0	0	0	5.9004153537719 (0.000625208191165675)	6.76470892149973 (2.02357202054032e-06)	0	0	0	0
AGMO	0	0	0	0	0	5.53943056468491 (0.00560033605482282)	0	0	0	0
AGPAT2	0	0	0	0	0	0	0	0	-2.84538859411813 (2.33646239972684e-06)	0
AGPAT9	0	0	3.36605958650376 (8.16953427482028e-07)	0	0	0	0	0	0	0
AGPS	0	0	0	0	-2.03952920888571 (0.00664563238919822)	0	0	0	0	0
AGR2	0	0	-4.19394595417968 (6.50963182449475e-05)	-7.35627850610121 (0.00158048555986933)	0	0	0	-6.29958253408852 (0.00706391625233)	-3.60165046071095 (4.57754111085281e-06)	-3.60810988461994 (0.0091593935505203)
AGT	0	0	5.01060802158443 (3.64056564226891e-11)	0	3.78021969721071 (0.000374287512873399)	7.29605949615931 (2.41319213041112e-16)	0	0	0	0
AGTPBP1	0	0	0	0	0	0	0	1.9309067566124 (0.00723886708401978)	0	0
AGTR1	0	0	0	0	0	0	0	0	3.55006169086506 (0.00368222082884782)	0
AGXT	0	0	0	0	0	10.8289806231362 (7.8133325162796e-21)	0	0	0	0
AGXT2	0	0	0	0	0	7.52103228123198 (1.2474023792114e-07)	0	0	0	0
AHCTF1	0	0	0	0	-1.78038777684294 (0.000260073985757304)	0	0	0	0	0
AHCYL2	0	0	-2.4103136613102 (3.08465558390825e-05)	0	0	0	0	0	0	-3.36002663148443 (0.000774643475151168)
AHDC1	0	0	0	0	0	0	0	-3.48326176956124 (0.00824962097052193)	0	0
AHNAK	0	0	0	1.76896195088865 (0.00414669102767818)	0	-1.98686882795988 (0.000595572297731014)	0	-2.98804882639612 (3.19906446922142e-06)	0	0
AHNAK2	2.88413489660342 (8.00869497480878e-09)	0	0	3.75909849642329 (9.91452005408193e-06)	0	0	0	-4.75165593001728 (0.0057290969989385)	0	3.87520485010033 (5.39181512943321e-06)
AHR	0	0	-1.50048614528164 (5.81514963310926e-05)	0	0	0	0	-3.46729943921792 (1.34798459114285e-05)	0	0
AHSG	0	0	0	0	0	11.0651208266171 (2.9841709988612e-19)	0	0	0	0
AIF1L	0	0	2.75625377243264 (4.73141705345284e-09)	0	4.07309789666696 (1.74684823695358e-09)	0	0	0	2.09402955741306 (2.32294148632157e-07)	0
AIFM2	0	0	0	0	0	0	0	0	-2.08014118693365 (4.13912597746995e-05)	0
AIG1	0	0	0	0	0	1.81428490529386 (0.00274560689344355)	0	0	0	0
AIM1	-1.85105027300136 (1.65591281292091e-05)	0	-2.0299171934076 (5.7500502583306e-05)	0	0	0	0	0	0	0
AIM1L	0	0	0	0	0	0	0	0	4.62062393331994 (8.29309258595871e-10)	0
AJAP1	0	0	0	0	0	5.77633301895591 (0.000371815851671778)	3.34494212683374 (0.00460712587155139)	0	0	0
AK1	0	3.72981627465258 (0.0018774413970025)	2.72740307574234 (3.15322474826408e-06)	0	0	0	0	0	0	0
AK4	1.86746052106954 (3.15402893287527e-08)	0	1.55835517403472 (5.67165602998646e-05)	0	0	2.041409290855 (3.16255929354103e-06)	0	0	0	2.31541465011616 (0.0014844870776449)
AK7	0	0	0	0	0	0	0	0	-4.1778040666498 (0.000146588977550336)	0
AKAP1	1.87728632502787 (1.52264464207746e-05)	0	1.80196832764247 (2.13064923422847e-05)	0	0	0	0	0	0	0
AKAP12	0	0	-1.52923642326512 (0.00546596669565367)	0	0	0	0	-3.56482768784893 (8.88761073444899e-06)	0	0
AKAP13	0	-2.97186404863589 (0.000767010388828055)	0	0	-1.79064011227597 (0.000250451939767936)	0	0	0	0	0
AKAP2	0	0	3.27471209012444 (5.09728203430914e-05)	0	0	0	1.94621563956728 (0.000205437861378842)	0	0	0
AKAP6	2.42917844835107 (4.48640342575309e-06)	0	3.73914722426458 (3.26881541236907e-11)	2.73077174263406 (0.00522371535347867)	0	0	0	0	0	0
AKAP9	0	-6.12016806695029 (0.00387693672786045)	0	0	0	0	0	0	0	0
AKIRIN1	0	0	0	0	0	0	0	-6.03047437803031 (0.00637025405159721)	0	0
AKNA	0	0	-2.33591652879609 (1.07576356437709e-05)	0	0	0	0	0	-1.63755797164638 (0.000595126850908274)	0
AKNAD1	0	0	0	0	0	0	0	0	3.8547494070985 (0.00402321915613103)	0
AKR1B10	0	0	0	0	6.91800884378814 (0.00837990899630342)	7.70409380374078 (5.48070124021529e-07)	0	0	0	6.79009821217895 (0.0026785245388961)
AKR1C1	0	0	0	0	2.29011173792428 (0.00080139165124174)	3.64650799991908 (6.02607118267944e-09)	0	0	-3.62549754894267 (1.40778481336819e-08)	0
AKR1C2	0	0	0	0	2.18683100912199 (0.00557735528648293)	3.72831911288124 (2.13093936407654e-13)	0	0	-4.99459019730865 (4.61347940167735e-07)	0
AKR1C3	0	0	0	0	0	0	0	0	-5.68114502248309 (5.21306922196148e-10)	0
AKR1C4	0	0	0	0	0	7.56550848466482 (2.19958182724076e-08)	0	0	0	0
AKR1D1	0	0	0	0	0	6.57229454377607 (1.00325162722874e-05)	0	0	0	0
AKR7A3	0	0	0	0	0	7.28140465910827 (3.12372577576866e-06)	0	0	0	0
AKR7L	0	0	0	0	0	4.98864367735528 (0.00204490293233807)	0	0	0	0
AKT3	0	0	0	0	0	0	0	-4.44074447526397 (7.78028127457822e-05)	0	0
AL022344.7	0	0	0	0	5.83005063945246 (0.00374446131635038)	0	0	0	0	0
AL035610.1	4.0176790431449 (0.00925858674371086)	0	0	0	0	0	0	0	5.25961127641861 (2.99857029383946e-06)	0
AL035610.2	0	0	0	0	0	0	0	0	5.39170360798719 (1.43620679045935e-06)	0
AL121932.1	0	0	6.46049632313754 (1.30676225673486e-05)	0	0	0	0	0	0	0
AL132709.8	0	0	0	0	0	0	0	0	5.63037327531451 (1.02951243822027e-09)	0
AL133493.2	0	0	0	0	5.55680815125306 (0.000919703341719475)	0	0	0	0	0
AL139099.2	2.18029576151625 (0.000125650653021328)	0	2.13107354856173 (0.00460971607996813)	0	0	2.17584391656604 (0.00410015961544293)	1.54901936558667 (0.000231602716217315)	0	2.04057247514057 (0.000134844127037325)	0
AL161626.1	0	0	0	0	0	5.95762280571853 (0.00174139201547661)	0	0	0	0
AL353644.10	3.05616358763427 (0.00408022649350079)	0	4.11447386695093 (7.16219725424407e-05)	0	4.38824362576709 (0.000125905946688189)	0	2.84315962638913 (0.00203422738012801)	0	0	0
AL353644.3	0	0	0	0	0	8.22763867757318 (0.000103144277947859)	0	0	0	0
AL353644.4	0	0	0	0	0	4.03619491814538 (0.000161331521361969)	0	0	0	0
AL353644.6	0	0	0	0	6.00101807038484 (3.477054808068e-06)	0	0	0	0	0
AL355075.1	0	0	0	0	0	0	1.58644814300456 (0.000191785348702923)	0	0	0
AL356289.1	0	0	0	0	0	6.01924806985817 (0.00191239931550822)	0	0	0	0

AL591893.1	0	0	0	0	0	6.20814193002162 (0.00647527445295343)	0	0	0	0
AL627171.2	0	0	0	0	0	0	3.51115443800228 (0.00261186867333775)	0	0	0
ALAD	0	0	0	0	0	0	0	3.32820592459093 (2.45927210747952e-06)	0	0
ALAS1	0	0	0	0	0	1.82090151360905 (0.00118200735987911)	0	0	0	0
ALAS2	-4.8792920327873 (2.85202583016073e-05)	0	0	0	0	0	0	4.94158914345989 (4.34313984188487e-07)	0	0
ALB	3.60957612437392 (0.00145856927435548)	0	0	0	0	12.2997143984548 (1.27545701602248e-56)	0	0	0	0
ALCAM	-1.87213367375158 (3.78524444191999e-05)	0	-4.16201454211823 (4.09666723061678e-08)	-1.98478483063048 (0.00943066849306618)	-3.68416533440172 (1.52951153800867e-05)	-1.81417648154301 (0.00592282485422131)	0	-3.2488831517231 (0.000415210188986612)	0	-1.96868456222478 (0.00948583121899978)
ALDH1A1	1.87092331653426 (3.36150430397859e-05)	0	0	0	0	0	0	0	0	0
ALDH1A3	1.80436270796325 (0.00222674048992525)	0	-2.11569608250338 (0.00883363580158654)	0	0	0	0	0	0	0
ALDH1B1	2.60071069436726 (3.64405633346615e-09)	0	0	2.30498893197703 (0.00636617624528821)	0	1.9505071553289 (0.00133778906888704)	0	0	0	0
ALDH1L1	0	0	3.20418462301652 (8.35182378001178e-08)	0	3.11674408753597 (0.000363882019171945)	5.94581750986126 (4.15411626341398e-15)	0	0	-3.32955424982104 (0.00657630582317896)	0
ALDH2	0	0	0	0	0	0	0	0	-3.50260805737814 (3.37568949644506e-17)	0
ALDH3A2	0	0	0	0	0	0	0	0	-1.75664991983903 (1.29005245773249e-05)	0
ALDH3B1	-1.82029972438493 (0.00323623396102313)	0	-2.20885909713823 (0.00073253449829952)	0	0	0	0	0	0	0
ALDH3B2	0	0	0	0	0	7.15930295843649 (0.00706173409822335)	0	0	5.07888364169156 (0.000143220919986297)	8.84322655167778 (1.49004358813255e-09)
ALDH4A1	0	0	0	0	0	5.16782860383479 (1.13460328788444e-06)	0	0	0	0
ALDH5A1	0	0	0	0	0	2.5053382723844 (0.00229230352135695)	0	0	0	0
ALDH6A1	0	0	0	0	0	2.56166891399502 (8.7480971244025e-05)	0	0	0	0
ALDH7A1P1	0	0	0	0	0	0	0	0	2.9519261499499 (0.00898665285794041)	0
ALDH8A1	0	0	0	0	0	7.92656936348947 (1.01024089165408e-09)	3.95512224062722 (0.0053281610841655)	0	0	0
ALDOA	0	0	1.75706356941366 (1.7282509364893e-07)	0	0	0	0	0	0	0
ALDOB	8.13091925357615 (2.68139220534701e-07)	0	0	8.68703755325713 (1.9048178055399e-10)	9.15495094919431 (6.11193779604815e-05)	12.1101356793574 (6.94782239927887e-25)	0	0	0	0
ALDOC	0	0	3.11415601029045 (2.00272015632662e-07)	0	0	0	0	0	0	0
ALG10	0	0	0	0	0	0	2.12822059971834 (0.00470393014118994)	0	2.0673368200362 (0.00869394586883329)	0
ALG10B	0	0	0	0	0	0	0	0	1.53589362570809 (0.000796897929356954)	0
ALG14	0	0	0	0	2.827850153386 (0.00792445428659529)	0	0	0	0	0
ALG1L9P	0	0	0	0	0	5.12959572081933 (0.00372615838714881)	0	0	0	0
ALK	3.62972595105616 (0.00766614865506304)	0	0	0	0	0	0	0	0	0
ALKBH5	0	0	1.51535831268336 (9.14123305353156e-06)	0	0	0	0	0	0	0
ALKBH7	0	0	1.99494849046274 (0.000496062082449419)	0	2.60061989124808 (0.00376519138126528)	0	0	0	0	0
ALOX12B	0	0	0	0	0	0	0	0	0	7.11177382293463 (6.06217722319067e-06)
ALOX15B	-5.82525778538 (4.46098963216643e-08)	0	-5.24537204195139 (3.81966042922052e-07)	-3.48181520866449 (0.0080662237753594)	-4.62953595580365 (0.0021061167508446)	-4.12987239286363 (0.00107427074806745)	0	-6.45323284769663 (0.00492368613176407)	-6.10140587830708 (4.83354644196296e-11)	0
ALOX5	-1.50892488285103 (0.000592099149239416)	0	-2.67482259764144 (0.00073417346901211)	0	0	0	0	0	0	0
ALOX5AP	0	0	-2.67730984535807 (0.000362386797481508)	0	0	0	0	0	-2.04159508932156 (0.00230881169946267)	0
ALPK1	0	0	-2.11659192551272 (3.55177294778687e-05)	-2.97040414754189 (0.00113661912323384)	0	0	0	0	0	0
ALPK2	0	0	8.62024341369958 (2.30248684592412e-21)	0	0	5.99859472959451 (1.98508620446358e-06)	0	0	5.19770103676996 (2.17669474103652e-07)	0
ALPK3	0	0	3.2048273809403 (3.72730758489258e-11)	0	0	0	0	0	0	0
ALPL	-5.11005350509094 (4.14893170734177e-13)	0	0	-6.46260126624198 (0.000130039788779583)	-3.33549842892217 (0.00105485766363135)	0	0	0	-6.7718544495212 (3.42622378912481e-19)	-4.42123628066802 (4.9643366147046e-05)
ALPP	0	0	0	0	0	0	0	0	7.23750668208989 (3.65509169737511e-07)	0
ALS2CL	-1.88454488073987 (0.000459825969767181)	0	0	0	0	0	0	0	0	0
ALS2CR12	0	0	0	0	0	0	0	0	-3.14040027739278 (6.52682694338036e-05)	0
ALX4	0	0	0	0	0	6.02900320501296 (0.00103795813026124)	0	0	0	0
AMBP	0	0	0	0	0	12.7678638927527 (7.54987842364982e-29)	0	0	0	0
AMDHD1	0	0	0	0	0	7.09380578255547 (1.36619427803807e-06)	0	0	0	0
AMICA1	0	0	-3.11297891996708 (0.00165879012338913)	0	0	0	0	0	0	0
AMIGO2	1.8650251626123 (0.0016094466488429)	0	0	3.26433775581783 (0.000259584130095259)	0	0	0	0	0	0
AMMECR1	-1.82703583601696 (0.00975138004885673)	0	0	0	0	0	0	0	0	0
AMN	0	0	0	0	0	4.61188527065562 (0.000378445135518497)	0	0	0	0
AMOT	2.10049453043499 (0.000571583036465029)	0	0	0	0	3.58096735088835 (0.000558835901953084)	1.75431227861417 (0.0051690623365619)	0	4.82863637047782 (4.85281889367336e-36)	0
AMOTL2	0	0	0	0	0	0	0	-7.04566584594105 (0.00181749951386941)	0	0
AMPD3	0	0	-2.80990033115008 (1.08042375095193e-05)	0	0	0	0	0	0	0
AMPH	0	3.77388262096256 (0.0055365395936427)	0	0	0	0	0	0	0	3.0482357846351 (0.00653251843524815)
AMZ1	0	0	0	0	0	0	0	0	1.90990607541225 (0.00417864624544714)	0
ANG	3.68354371048813 (0.00702025524511916)	0	0	0	0	6.90717044231516 (1.72919790336296e-08)	2.89534493530106 (0.00332651105302629)	0	0	0
ANGPT1	-1.80691513379498 (0.0022666245594927)	0	-3.07929708168631 (1.39513980713702e-06)	-3.3506458077401 (0.000672499152578279)	0	-2.39334873786702 (0.00136252681140512)	0	-3.29466357368728 (0.00456785642179428)	0	-3.40940022723502 (0.00038592733591775)
ANGPT2	0	4.15973109138646 (4.25366231732857e-05)	0	0	0	0	0	0	0	0
ANGPT4	0	0	0	0	0	7.26793601984222 (0.000733571029014321)	0	0	0	0
ANGPTL1	3.66958999985378 (8.4713467872941e-12)	0	0	0	0	0	0	0	2.95844643754497 (1.42776464207151e-12)	0
ANGPTL2	1.87443489167092 (2.75966051249433e-05)	0	0	0	0	0	0	0	0	0
ANGPTL3	0	0	0	0	0	6.89062270295349 (2.60080253106208e-12)	0	0	0	0
ANGPTL4	0	2.49441513564306 (0.00951634790224857)	0	0	0	1.94074514858788 (0.00317924870227596)	0	0	0	2.28369011125027 (0.00437272761263842)
ANGPTL6	0	0	0	0	0	7.29624285392293 (2.9448720259491e-07)	0	0	0	0
ANK1	0	0	3.31881724645079 (1.53625606257675e-07)	0	2.51279169975742 (0.00431936859118299)	0	0	7.31093245426476 (2.15432631685196e-22)	0	0
ANK2	3.09881622063684 (4.64946753304134e-09)	0	2.82249880647936 (1.69918371576072e-07)	3.26124634719084 (0.000277869254647304)	0	0	0	-6.26507149296356 (0.00606315780823294)	-1.77686276822735 (0.00438270898723852)	2.99438062415095 (0.000945628076834495)
ANK3	0	0	2.24469584693094 (2.40033829135305e-08)	0	0	0	0	0	0	0
ANKFN1	0	0	0	0	0	0	0	0	2.37636094914869 (0.00851569050019995)	0
ANKH	0	0	1.91992878368505 (6.9110883478078e-07)	0	0	0	0	0	0	0
ANKLE1	0	0	0	0	0	0	0	4.5104500914372 (0.00459324031072538)	0	0
ANKRD1	-9.84438993151146 (7.24573648711277e-10)	0	3.85794105672927 (3.81343366375432e-05)	-6.93633510602505 (0.00155109580466054)	-5.06570159466393 (0.000105914956259369)	-6.1144976318792 (4.50535097165402e-07)	0	0	-3.84683554840909 (3.04248429842265e-05)	-5.63561089048366 (0.00544498166921023)
ANKRD13A	0	0	-1.50633035959653 (1.42725775431307e-05)	0	0	0	0	0	0	0
ANKRD18CP	0	0	0	0	0	5.05975715855891 (0.00396435802052786)	0	0	0	0
ANKRD2	0	0	9.50074373998421 (1.84358897727535e-11)	0	0	0	0	0	0	0
ANKRD20A5P	0	0	0	0	0	0	0	0	3.12089740188673 (0.00155254179180012)	0
ANKRD29	0	0	0	0	0	0	0	0	-4.16308556064138 (8.81554512457484e-06)	0
ANKRD32	0	0	0	0	0	0	0	2.36326237565085 (0.0086915413432731)	0	0
ANKRD33	0	0	0	0	0	7.28925638830003	0	0	5.14124902521734	0

						(0.00568970025826563)			(0.000493648734436189)	
ANKRD34A	0	0	0	0	0	4.16458006621691 (0.00251602838261876)	0	0	0	0
ANKRD35	0	0	0	0	0	0	0	0	0	3.20014081207543 (0.00999877973251405)
ANKRD36BP2	0	0	0	0	0	0	2.78477610273622 (5.47262423818046e-06)	3.59101217972039 (0.00563625906720687)	0	0
ANKRD40	0	0	0	0	0	0	0	-2.56950007472087 (0.00248280146032339)	0	0
ANKRD44	0	0	-1.5302273219665 (0.00389394829720081)	0	0	0	0	0	0	0
ANKRD53	0	0	0	0	0	5.61094368372754 (0.00511611814747)	0	0	0	0
ANKRD6	0	3.25215643593306 (0.00429433077010663)	0	0	0	0	0	0	0	0
ANKRD9	0	0	2.56499955990273 (3.84763948893095e-07)	0	0	0	0	0	0	0
ANKS1B	2.52347745246958 (0.00102799311567609)	0	0	0	0	0	0	0	0	0
ANKS4B	0	0	0	0	0	8.11233848418261 (3.08578729167049e-11)	0	0	0	0
ANKS6	0	0	0	0	0	0	0	0	-2.01363563610843 (0.00495874957452054)	0
ANLN	0	0	0	0	0	0	0	3.43578933677975 (0.000951503939741539)	0	0
ANO1	1.85036980311606 (0.00852343952250921)	0	0	0	0	0	0	0	0	0
ANO3	0	0	0	0	0	4.71451872941972 (0.0016170536491495)	0	0	0	0
ANO5	2.12721399951369 (0.000722922659211)	0	3.2185827061557 (7.85805769112462e-08)	0	0	0	0	0	-3.40234609386279 (0.000692687430705888)	0
ANO9	0	0	-2.93142103844563 (0.00918876907025999)	0	0	0	0	0	0	0
ANP32A	0	0	0	0	0	0	0	1.73324383548287 (0.00412067247282049)	0	0
ANP32B	0	0	0	0	0	0	0	2.10822046701287 (0.00445397728412093)	0	0
ANP32E	0	0	0	0	0	0	0	1.87307213917696 (0.00769563743676918)	0	0
ANTXR1	0	0	0	0	0	0	0	-6.77401038100585 (2.14475445546525e-05)	0	0
ANTXR2	2.19208288316675 (5.69750728949641e-11)	0	0	2.10289301521667 (0.000828812289451179)	0	0	0	0	0	0
ANTXRL	0	0	0	0	6.798559966251 (0.0016583499845375)	0	0	0	0	0
ANXA1	0	0	-1.60213788293591 (5.17547304175935e-05)	0	-1.6667271850241 (0.000333835744453538)	-2.3342478406901 (1.00413919766675e-05)	0	0	1.5434494971763 (2.35729283271338e-05)	0
ANXA13	0	0	0	0	0	6.95412718041661 (0.00071330515515318)	0	0	0	0
ANXA2	0	0	0	-1.60514653697974 (0.0087797011038679)	0	0	0	-3.1887504998226 (4.08301752559828e-06)	0	0
ANXA2P2	-2.29454568577414 (0.00447169940388993)	0	0	0	0	0	0	0	0	0
ANXA3	0	0	0	0	0	0	0	2.0156052803839 (0.00916414757875848)	0	0
ANXA4	0	0	-1.58396592045242 (0.000346149638282957)	0	0	0	0	0	0	0
ANXA8	0	0	0	0	0	0	0	0	4.87022119588786 (1.01773057452764e-09)	0
ANXA8L1	0	0	0	0	0	0	0	0	2.81799398674061 (0.000113002730640461)	0
AOAH	-2.32093074100608 (0.00286216112390266)	0	-2.74112963454316 (0.00245094482972611)	0	0	0	0	0	0	0
AOC1	0	0	0	0	7.56974435845542 (5.18175562584263e-06)	6.54465898329173 (0.00355480838821528)	0	0	11.5920150980401 (2.46518416359751e-29)	0
AOC3	0	0	-2.21868668046361 (9.51797233875745e-07)	0	-1.60145036591919 (0.00527998724461741)	-1.8901591262563 (0.000307955325817153)	0	-6.46768109350419 (4.66857458278201e-05)	0	0
AOC4P	0	0	0	0	0	0	0	0	-3.33872824071525 (0.00838506793901178)	0
AOX1	0	0	0	0	0	4.21403947597609 (5.09670504464232e-08)	0	-6.66717622476232 (0.00307078732294703)	0	0
AP000230.1	0	0	4.84754379455407 (0.00575413896735819)	0	0	0	0	0	0	0
AP000708.1	0	0	0	0	0	5.6960474793485 (0.000525964875139289)	0	0	0	0
AP000997.2	0	0	0	0	0	8.19250618406407 (0.00109421045875971)	6.40843255592276 (0.00828411141417714)	0	0	0
AP000997.3	0	0	0	0	0	7.30538415343277 (0.00475228636090536)	0	0	0	0
AP001046.5	0	0	0	0	3.89155104067777 (0.00519992690206733)	3.48838606240453 (0.00671283186783273)	0	0	0	0
AP001187.9	0	0	0	0	0	6.10755852975053 (1.04367598762015e-05)	0	0	0	0
AP001626.1	0	0	0	0	0	5.15092075995531 (0.00349326389045762)	0	0	0	0
AP001626.2	0	0	5.14671025593822 (0.000815208784861423)	0	6.30120091593639 (0.00140082310262185)	7.59845717568359 (2.15634697908795e-07)	4.74786706632273 (0.00174605918373999)	0	0	0
AP001627.1	0	0	0	0	4.79428893199474 (0.00295825242970303)	0	0	0	0	0
AP003025.2	0	0	0	0	0	0	3.8825609584551 (0.00901196165663799)	0	0	0
AP003774.4	0	0	0	0	6.65447770053204 (0.00254565647500638)	0	0	0	0	0
AP006285.6	0	0	0	0	0	0	0	0	4.08513178827357 (0.00630950662572699)	0
AP1M2	-3.51654540156044 (0.00102307195974122)	0	-4.03300636495585 (0.00105596432826384)	0	0	0	0	0	0	0
AP1S3	-3.48408199076048 (0.00486801785830076)	0	0	0	0	0	0	0	0	0
AP3S1	0	2.59586178527621 (0.00479539392750024)	0	0	0	0	0	0	0	0
AP4B1	0	0	0	0	0	0	1.82978027174849 (0.00592419330747014)	3.01104250980341 (0.00716680657749987)	0	0
AP4E1	0	0	-1.65581388470142 (0.00185708021457949)	0	0	0	0	0	0	0
AP5B1	-2.02157640653198 (0.000309992886183146)	0	0	-3.60295374828885 (0.00813235550695461)	0	0	0	0	0	0
APBA1	1.80441849391505 (0.00392868916430706)	0	0	0	2.46672977718347 (0.00565777749507766)	3.30598102150665 (0.000428410555330201)	0	0	2.02934486493225 (0.00159143063611316)	0
APBB1	0	0	2.16432899582901 (5.58937409037696e-05)	0	0	0	0	0	0	0
APBB1IP	0	0	0	0	0	0	0	2.33704165027128 (0.00836893380000892)	0	0
APBB2	0	0	0	0	0	0	0	-2.93358132825534 (0.00273145293089487)	0	0
APC2	0	0	0	0	0	4.31194188287486 (0.00120722958204974)	0	0	0	0
APCDD1	0	0	0	0	0	0	0	0	2.45022596208532 (1.7877028751353e-05)	0
APCDD1L-AS1	0	0	0	0	0	5.02955115771474 (0.00426146523919458)	0	0	0	0
APCS	0	0	0	0	0	11.2438965932339 (1.11043042772585e-17)	0	0	0	0
APLN	-2.57092628621718 (0.00766208671525657)	0	0	-7.01273784388174 (0.00426774735084707)	0	0	0	0	0	0
APLNR	0	0	6.59494457018222 (4.78918307665553e-10)	0	5.84776782581898 (0.000693158964850091)	5.32254291670715 (0.000199691819971159)	4.75314229180407 (9.72925760439315e-05)	0	7.15429231862712 (5.11061014989523e-06)	0
APLP1	0	0	0	0	4.92024845942125 (0.00799730271179513)	4.87231031413018 (0.00663810175274752)	0	0	0	0
APMAP	0	0	0	0	0	0	0	2.30194874859931 (0.000240353512794424)	0	0
APOA1	0	0	7.30944930162493 (0.000124946864021617)	0	0	11.4631463267624 (1.20988252919874e-20)	0	0	0	0
APOA1BP	0	0	2.14308949628687 (8.54054994719316e-05)	0	0	0	0	0	0	0
APOA2	0	0	0	0	0	8.93844505320132 (2.57757855875914e-17)	0	0	0	0
APOA4	0	0	0	0	0	10.9295846551809 (4.16118899654737e-14)	0	0	0	0
APOA5	0	0	0	0	0	8.76712133275591 (1.56959497462564e-11)	0	0	0	0
APOB	3.56227647523578 (5.00760077143222e-06)	0	3.14223318560111 (3.16120175221917e-05)	0	0	10.487239402403 (4.15983484744373e-29)	0	0	0	0
APOBEC2	0	0	8.73498135501749 (7.27140353889833e-15)	0	0	0	0	0	0	0
APOBEC3A	-5.97024921111853 (1.5342224850819e-05)	0	-3.47200923662134 (0.00634550915185627)	0	0	0	0	0	0	0
APOBEC3D	0	0	0	0	0	0	1.73210641326237 (0.00386287955526359)	0	0	0
APOBR	-2.57335514099011 (3.00968628190287e-05)	0	-2.5212505851144 (0.000358262897409487)	-2.58814735578193 (0.00527908885368094)	-2.61757729883737 (0.00553545305214281)	0	0	0	-1.66169302284804 (0.00479831240571681)	0

APOC1	-3.32805411767347 (0.000466158556889234)	0	0	0	0	3.18752751557487 (4.50847753605153e-05)	0	0	0	0
APOC1P1	0	0	0	0	0	5.75843995666774 (0.00323365941088685)	0	0	0	0
APOC2	0	0	0	0	0	7.77832517701357 (1.79578141629665e-10)	0	0	0	0
APOC3	0	0	0	0	0	12.0123867983102 (1.84721058794979e-30)	0	0	0	0
APOD	0	0	3.67919788817887 (4.19370853440337e-08)	0	0	0	0	0	0	3.75543757197551 (1.87173415770967e-06)
APOE	-3.28703355596298 (2.85229794278744e-07)	0	0	0	0	2.62061336326804 (0.00159941798966576)	0	0	-1.88830293214484 (0.000242392683403557)	0
APOF	0	0	0	0	0	8.15047998134234 (1.48870783129304e-10)	0	0	0	0
APOH	0	0	0	0	0	11.2420890365027 (2.14449306318246e-19)	0	0	0	0
APOL1	0	0	0	0	0	0	0	0	0	-2.31942669041961 (0.00204226941402268)
APOL4	0	4.90888581063447 (0.000181676444718545)	0	0	3.14638432985848 (0.00201481123973561)	0	2.79457773489727 (0.0001261681036178)	0	1.74011746241767 (0.00857350519762707)	0
APOM	0	0	0	0	0	4.72897753645381 (0.00183254657331598)	0	0	0	0
APRT	0	3.9614528308115 (6.30319075703007e-05)	0	0	0	0	0	0	0	0
AQP1	-2.01118989283271 (2.26395655162905e-05)	0	0	0	-2.39837118989848 (0.00201313672148026)	-2.82899981435538 (0.00021700024723265)	0	-2.98509981080004 (0.000808084139248276)	-3.69711184495711 (1.38383047268914e-16)	0
AQP2	0	0	0	0	10.0313619647612 (4.73754333463478e-16)	0	0	0	0	0
AQP3	-3.50410941457865 (5.23357380117055e-09)	0	0	-3.42106244482601 (0.00226677601538055)	0	0	0	0	-3.01118905700981 (1.19576846800967e-07)	0
AQP4	-8.58292581378751 (2.0545745028898e-12)	0	-4.35095754310089 (7.91903122006458e-08)	-6.69327147363988 (0.000160606571000777)	-4.21012660538371 (0.000454107760861284)	-4.45148377775062 (4.66558966483435e-05)	0	-8.07896124358282 (0.000437699516305722)	-6.68577567072512 (2.08268932340249e-08)	-6.97316062993466 (8.46901253241776e-05)
AQP6	0	0	0	0	6.52373871996049 (1.29040452798938e-05)	6.3104054400639 (0.00647666131582755)	0	0	0	0
AQP7	0	0	7.12323826209313 (4.58481327436104e-18)	0	5.23456598209858 (0.000110698213772502)	5.54298642075439 (5.30576598602834e-06)	0	0	0	7.05619399028573 (3.34077404352775e-09)
AQP8	0	9.24943000594362 (4.79443712316101e-08)	0	0	0	0	0	0	0	0
AQP9	-3.79980248485368 (0.00351398966098742)	0	-2.87999664710735 (0.0019983669125114)	-3.99663692146997 (0.00122993420440279)	-3.7328174330718 (0.00294479072956172)	0	0	0	-2.55318193372811 (0.000815033828671676)	0
AQPEP	3.88331278066656 (1.59845219216352e-05)	0	0	0	0	0	0	0	7.52615114611478 (2.73090412444471e-22)	0
AR	0	0	2.07975915372167 (2.4352214523471e-05)	0	0	3.28308056710233 (1.10956494878605e-08)	0	0	0	0
ARAP1-AS2	-5.5076692552011 (2.34997997602568e-06)	0	0	0	0	0	0	0	-1.99316396356917 (0.00044840059851627)	0
ARAP2	0	0	-2.07056145259231 (0.0015828894959164)	0	0	0	0	0	0	0
ARAP3	-1.75930689494493 (0.00295186314480494)	0	0	0	0	0	0	0	0	0
ARCN1	0	-5.77908680307327 (0.00679073018235211)	0	0	0	0	0	0	0	0
AREG	-3.17963493797409 (4.82382527912659e-06)	0	-3.16262288979963 (1.4346722445848e-05)	0	-3.90947740640853 (0.00346608396242511)	-3.88573591157893 (0.000905318028896116)	0	0	0	0
ARG1	0	0	0	0	0	4.69351878916478 (1.53128500640563e-07)	0	5.20494182736475 (1.60917703411815e-07)	0	0
ARHGAP10	0	0	0	2.01997283763429 (0.00182272540764553)	0	0	0	0	0	0
ARHGAP11A	0	0	0	-6.07425354618224 (0.00801210861825254)	0	0	0	3.69745058880719 (7.74997541537059e-06)	0	0
ARHGAP11B	0	0	0	0	0	0	0	4.67691662808795 (7.75247697195015e-06)	0	0
ARHGAP15	0	0	0	0	0	0	0	2.53447346550309 (0.00379855100277602)	0	0
ARHGAP18	-1.55497820020902 (0.0006236516912447)	0	-2.34758027662909 (5.51963079158475e-06)	-2.00119221086108 (0.00898001894521395)	0	-3.17241444563735 (3.06926151463217e-06)	0	0	0	0
ARHGAP19	-1.91300391642936 (0.00158138476176771)	0	-2.43610232811247 (0.00396108735813588)	0	0	0	0	0	0	0
ARHGAP19-SLIT1	0	0	0	0	0	5.25024380134881 (0.00895461229631386)	0	0	0	0
ARHGAP20	0	0	-4.02603649557497 (6.609153802726e-05)	0	0	0	0	0	0	0
ARHGAP25	-1.94154664525303 (0.000455998738683724)	0	-1.54637453592073 (0.0083002163849375)	-2.65305764188246 (0.00395322463978954)	0	0	0	0	-1.57355289841195 (0.000468015504901565)	0
ARHGAP27	-2.21813110770059 (4.17884729383773e-05)	0	0	0	0	0	0	0	0	0
ARHGAP29	0	0	0	-2.43299811118222 (0.00323620867555588)	0	0	0	-3.26549839176144 (0.000676430612556526)	0	0
ARHGAP30	-2.84696001670508 (1.25085159134055e-05)	0	-3.42751481812501 (5.7810343125837e-08)	0	0	0	0	0	-1.99071781187759 (8.61272416444873e-07)	0
ARHGAP31	0	0	0	0	0	0	0	-2.78552658440578 (0.000781690804165419)	0	0
ARHGAP42	0	0	0	0	-2.84834662431432 (0.00977715461893627)	0	0	-6.29771414114097 (0.00406521660590622)	0	0
ARHGAP9	-2.63103648400463 (0.00123799348154291)	0	-2.20805993075187 (0.00992294132096088)	0	0	0	0	0	0	0
ARHGDIB	0	0	-1.5119399656045 (0.00338918977208969)	0	0	0	0	0	0	0
ARHGEF10	0	0	0	0	0	-1.63350331779057 (0.00969277588538999)	0	-3.59491762134628 (0.00084328882338083)	-1.72252075353884 (8.97078377515506e-05)	0
ARHGEF10L	0	0	1.54649218945142 (0.00315722883239189)	0	0	1.92707093649891 (0.00769913712332919)	0	-6.081032316715 (0.00785377100940712)	0	0
ARHGEF11	0	0	0	0	0	0	0	-2.58056685095943 (0.0028316188258548)	0	0
ARHGEF16	0	0	0	0	0	3.62851182457169 (0.000998721883360563)	0	0	2.20171026387877 (0.00110994050941423)	0
ARHGEF25	2.48764332807256 (4.31081873534008e-06)	0	0	0	0	0	0	0	0	0
ARHGEF26	1.76788501695483 (3.43938281512532e-05)	0	-1.92875745534998 (0.00675772029093829)	0	0	0	0	-6.39756059708449 (0.00401664728089028)	0	0
ARHGEF26-AS1	0	0	0	0	0	5.34548912647045 (0.00360919455256918)	0	0	0	0
ARHGEF28	0	0	0	0	2.6941021211888 (8.72091402515391e-05)	0	0	0	0	0
ARHGEF37	0	0	1.62593465485471 (0.000180418235697116)	0	0	0	0	0	0	0
ARHGEF38	-4.19869110833551 (9.6718237345372e-05)	0	-3.79615375033489 (0.000251892842189486)	0	0	0	0	0	-5.18447568293211 (5.02569689116387e-08)	0
ARHGEF4	0	0	0	0	0	0	0	0	0	4.77426699251611 (0.000142598780102122)
ARHGEF5	0	0	0	0	0	2.56274703681329 (0.00993154241004339)	0	0	0	0
ARID3A	0	0	0	0	0	0	0	0	2.24594963325163 (2.0793795002399e-18)	0
ARID3B	-1.72195656979575 (0.00136347544315973)	0	0	0	0	0	0	0	0	0
ARID5B	0	0	0	0	0	0	0	-2.06519648573519 (0.00207762889605333)	0	0
ARL1	0	0	0	0	0	0	0	-2.87823640420317 (0.00510901080396613)	0	0
ARL2	1.55426553915741 (0.0077903170846964)	0	0	0	0	0	0	0	0	0
ARL4A	0	0	0	0	0	0	0	0	0	2.53372451685927 (0.00467317223761033)
ARL4C	0	0	-3.33920448004887 (5.55451252607347e-06)	0	0	0	0	0	0	0
ARMC3	0	0	0	0	0	0	0	0	-4.7047638204043 (0.00146344087236368)	0
ARMC9	1.89152290731122 (5.02656526578086e-06)	0	-1.88148726640072 (0.00123429168515572)	2.14130848264502 (0.00448831711009787)	0	0	0	0	0	0
ARMCX2	1.56668880831611 (0.0048718303046759)	0	0	0	0	0	0	0	0	0
ARNT2	0	0	0	0	0	0	1.60156475178017 (0.00720857610885444)	0	0	0
ARNTL2	0	0	-2.40055780372593 (0.000102269384899835)	0	-4.07193321373521 (0.00238937403270441)	0	0	0	0	0
ARPC3	0	0	-1.60444599959391 (0.00245771571771175)	0	0	0	0	0	0	0
ARPIN	0	0	0	0	0	0	0	-6.9581705629549 (0.00148956164687866)	0	0
ARPP21	0	0	0	0	0	6.74786481380398 (8.80437728601864e-06)	0	0	0	0
ARRB2	0	0	-1.62089202086013 (0.00117540020190989)	0	0	0	0	0	-1.88961584924744 (3.72592860140761e-07)	0
ARRDC2	-1.85381249978641 (0.00383026577507194)	0	-1.67669237127112 (0.00581109063092678)	0	-2.71029358060755 (0.00840091122030536)	0	0	0	-2.05726472155254 (0.00184147823858324)	0
ARRDC3	0	0	-2.84769477583386 (7.30646689087837e-12)	0	0	0	0	0	0	0
ARRDC4	0	0	0	0	0	-2.55954048815684 (0.00588070664742047)	0	0	0	0
ARSE	0	0	0	0	0	5.44419727377073	0	0	0	0

						(0.00418268940178505)				
ARSJ	-1.98664281397842 (0.00517528881745678)	0	0	0	0	0	0	0	0	0
ART3	0	0	7.01680802029288 (1.48425283001255e-08)	0	0	0	0	0	0	0
ART4	0	0	0	0	0	3.40986613112171 (0.00289687491028235)	0	0	4.57622227201702 (6.85229830077005e-08)	0
ARVCF	-2.26751255322984 (0.00568211996025939)	0	0	0	0	0	0	0	-2.47750760760275 (0.000146926012659573)	0
ASAH1	-1.68115534162665 (0.00309342189722523)	0	0	0	0	-1.93398389494738 (0.00703138430090098)	0	0	0	0
ASAP3	0	0	1.65289488214765 (0.000157877324652771)	0	0	0	0	0	0	0
ASB1	0	0	1.61739377103855 (0.00196320587596221)	0	0	0	0	0	0	0
ASB10	0	0	4.86844905634285 (0.00130411905133341)	0	0	0	0	0	0	0
ASB11	0	0	8.12057647148566 (6.19433242722946e-12)	0	0	0	0	0	0	0
ASB15	0	0	5.43353342190398 (9.82743979358828e-06)	0	0	0	0	0	0	0
ASB18	0	0	4.7602890881983 (0.00113723014344896)	0	0	6.16980119559886 (0.00649144516590301)	0	0	0	0
ASB2	4.87647590231046 (4.10272391736986e-12)	0	5.61429373776639 (4.94959828158646e-15)	5.75405381635466 (6.67286116514576e-07)	0	3.93607729995842 (0.00987304752144421)	0	0	0	0
ASB4	0	0	0	0	5.2591357430818 (0.00365962439263801)	0	0	0	0	0
ASCL2	0	0	0	0	0	0	0	0	4.73682448771695 (1.41343201573978e-07)	0
ASF1B	0	0	0	0	0	0	0	4.544846333776 (0.000678084693964115)	0	0
ASGR1	0	0	0	0	0	6.73955002869236 (1.36111055886779e-16)	0	0	-4.65778397372044 (0.000473944468124856)	0
ASGR2	0	0	0	0	0	6.32788414914783 (3.05754257752026e-11)	0	0	0	0
ASIC1	2.84792548350987 (0.00249930719690266)	0	0	0	0	0	0	0	0	0
ASL	0	0	0	0	0	2.5138257339084 (1.31720340280185e-05)	0	0	0	0
ASMT	0	0	0	0	0	5.65769172927683 (0.00339014467838121)	0	0	0	0
ASPG	0	0	3.22276484763877 (0.00291528540065867)	0	0	6.0978909593417 (1.10970132505303e-10)	0	0	0	0
ASPH	0	0	0	0	-1.58188031299907 (0.00540295147338511)	0	0	0	0	0
ASPHD1	-3.73604250640707 (0.00735739160105282)	0	0	0	0	0	0	0	0	0
ASPHD2	0	0	0	0	0	0	4.18357360332493 (0.00263767426616529)	0	3.90679433248204 (0.00221502607910259)	0
ASPM	0	0	0	0	0	0	0	4.23444464543379 (4.04940712563073e-05)	0	0
ASPN	2.854116652947 (3.86466719436952e-07)	0	0	0	0	0	0	0	0	0
ASPRV1	0	0	4.12687500408946 (0.00459649802056131)	0	0	0	0	0	0	7.8839170712239 (1.88371778996008e-07)
ASRGL1	0	0	0	0	0	0	0	0	-3.29446154510472 (0.000211989743040938)	0
ASS1	0	0	1.92395971093474 (5.54899798098757e-05)	0	1.72762385812008 (0.00964390628030431)	3.98185500348206 (8.7454771862626e-08)	0	0	0	0
ASTE1	0	0	0	0	0	2.46894524434779 (0.00938078559958202)	0	0	0	0
ASTN1	0	0	0	0	6.32495481118369 (0.00525243568177162)	7.30218884254118 (1.64232961666336e-05)	0	0	0	0
ASTN2	0	0	0	0	0	3.30414174172403 (0.00430189276802407)	0	0	0	0
ATAD2	0	0	0	0	0	0	0	3.44133569704565 (1.04352186995388e-06)	0	0
ATAD5	0	0	0	0	0	0	0	5.24954348761837 (8.57361374890083e-11)	0	0
ATCAY	4.00525044313184 (0.00284426488232401)	0	0	0	0	0	0	0	0	0
ATF3	-3.27253520837414 (2.05189786407901e-07)	0	0	0	-2.03468785697786 (0.00746188397949673)	0	0	-4.74537540291484 (4.61288909132752e-05)	0	0
ATF5	0	0	2.27646026660804 (0.00752608761221936)	0	0	5.94636426802031 (2.13711567359164e-18)	0	0	0	0
ATG16L2	-2.53676404683481 (4.89270491530821e-06)	0	-1.76861312274337 (0.000647035167145994)	-2.84896798221256 (0.00213865305819784)	0	0	0	0	-1.68793312940145 (9.88098259371328e-05)	0
ATG4D	0	0	2.62021107189896 (0.000741168790408093)	0	0	0	0	0	0	0
ATG9B	0	0	0	0	0	5.17664911313689 (0.00567216653954686)	0	0	6.88789209988485 (5.2703345301581e-19)	5.14678817556971 (0.00101219066876255)
ATHL1	-1.7923906442458 (0.00709084044245457)	0	-1.61688694847972 (0.00828034819540568)	-2.8027609999137 (0.00929704768824818)	0	0	0	0	-2.79088466337834 (6.52245294362097e-07)	0
ATL2	2.42720433746693 (2.5106488019107e-13)	0	0	1.87006538625558 (0.00610346551685962)	0	0	0	0	0	0
ATL3	0	0	0	0	0	0	0	-1.85031195475742 (0.0062923431725337)	0	0
ATP10A	-2.01755500775318 (0.00680255858235587)	0	0	0	0	0	0	0	0	0
ATP10B	0	0	0	0	5.97858440900229 (2.1317759109534e-05)	7.05067399147698 (9.47001098819897e-08)	0	0	0	5.44772011902913 (0.00409515473607798)
ATP10D	0	0	0	0	0	0	0	-2.40464483665071 (0.00910630726052521)	0	0
ATP11A	-1.66843248222963 (2.06119901706109e-06)	0	0	-1.77147462629984 (0.00937148336744778)	-1.95772391347582 (0.000414125919818064)	0	0	0	0	0
ATP12A	0	0	0	0	0	0	0	0	0	6.10046195518675 (1.14541693739355e-05)
ATP13A3	-1.89052961971591 (6.07192038030075e-07)	0	0	-1.87475113625044 (0.00528355905593375)	-1.96976730103206 (0.00151440862927638)	0	0	-1.86737459446742 (0.00803113667291225)	0	0
ATP13A4	-6.00052092973888 (3.08223245104686e-08)	0	-5.66084083443567 (3.05167915338799e-09)	-7.24958486591506 (0.00113077007559637)	0	0	0	-6.1910471113978 (0.00564244666712194)	0	0
ATP1A1	0	-3.18747470577414 (0.00664375458141878)	0	0	0	-1.55895354240771 (0.00302230544975032)	0	-2.01129353103094 (0.00149208490359534)	0	0
ATP1A1-AS1	0	0	0	0	0	0	0	0	-1.72298038678909 (0.00926048263506121)	0
ATP1A2	2.70939998396031 (1.20512354032415e-05)	0	4.376312005972 (1.50121033896924e-12)	3.62853873200372 (0.000269144912527072)	0	0	0	0	0	0
ATP1A3	4.86589319920497 (9.93933496246134e-05)	0	9.38340340979039 (6.3683868643937e-20)	0	5.2928439506446 (0.00908567205209642)	0	0	0	0	0
ATP1B1	0	0	0	0	0	0	0	-3.10040671897845 (0.00154202522762576)	-1.7689556363981 (3.33148339128189e-05)	0
ATP1B2	2.6459121622371 (0.000109141122786728)	0	0	3.05986832258434 (0.0018714683238879)	0	0	0	0	0	0
ATP2A2	0	0	2.68545499003952 (7.21622388194873e-13)	0	0	0	0	0	0	0
ATP2A3	0	0	0	0	0	0	0	2.11742220304872 (0.00631423535044773)	-2.88188877742637 (4.6579880393309e-09)	0
ATP2B2	0	0	0	0	5.74662776654196 (6.02131428011409e-05)	8.03495949385967 (3.58815837579814e-14)	0	0	0	0
ATP2B3	0	0	0	0	5.46967821176292 (0.0025456407800973)	6.79692181232265 (0.00011013746103806)	0	0	3.72777835703397 (0.00580929028663476)	0
ATP2B4	1.79511530819747 (1.56499375852744e-08)	0	0	1.71393475248252 (0.00555737301209204)	-1.69039412979612 (0.000594561596479111)	0	0	0	0	0
ATP2C1	0	0	0	0	-1.70808606814773 (0.00512242640890202)	-1.74025367778941 (0.000809507088822353)	0	0	0	0
ATP2C2	-3.68456780706648 (0.000267107896856461)	0	0	0	0	0	0	0	0	0
ATP4A	0	0	0	0	6.24434293510046 (0.0075784156376657)	0	0	0	0	0
ATP5A1	0	0	1.73555810037247 (1.02606461812982e-05)	0	0	0	0	0	0	0
ATP5A1P3	0	0	0	0	0	5.57020872414248 (0.000203519530244698)	0	0	0	0
ATP5B	0	0	1.9468209803723 (3.12315719648454e-08)	0	0	0	0	0	0	0
ATP5D	0	0	1.86076780478879 (0.000104034203334427)	0	0	0	0	0	-1.68961793234591 (0.00235387619449424)	0
ATP5G1	0	0	2.72187872101293 (2.49747552827103e-05)	0	0	0	0	0	0	0
ATP5G1P3	0	0	0	0	0	7.26986837985775 (0.00668205493536917)	0	0	0	0
ATP5G3	0	0	1.91315184581834 (0.00120974287249091)	0	0	0	0	0	0	0
ATP5I	0	0	1.70615634793073 (0.00242441011427669)	0	0	0	0	0	0	0
ATP5O	0	0	1.7109111372459 (0.000900173905250731)	0	0	0	0	0	0	0
ATP5SL	0	0	0	0	0	0	0	0	0	-2.54705238823168 (0.00806225596055883)
ATP6V0A4	0	0	0	0	5.9842069297552 (0.00169453865488575)	0	0	0	0	0

ATP6V0B	0	2.72672263477231 (0.00151691923727653)	0	0	0	0	0	0	0	0
ATP6V0E2	0	0	3.57116958757823 (2.17701609054492e-07)	0	3.60296950226961 (0.00189435570321252)	0	0	0	0	0
ATP6V1A	0	0	-2.29442356885462 (2.96061831146572e-05)	0	0	0	0	0	0	0
ATP6V1B1	0	0	0	0	0	0	0	0	3.68905641273584 (0.000258687571735264)	0
ATP6V1B1-AS1	0	0	0	0	7.20697900913001 (0.00515462419084721)	0	0	0	0	0
ATP6V1B2	-1.77271574166948 (9.58934512052231e-05)	0	-2.75981215294576 (4.07696529478024e-09)	0	-2.15239273147574 (0.00188401750648581)	0	0	0	-1.56368372207154 (1.35330861480882e-05)	0
ATP6V1C1	0	0	-1.60556214473396 (0.00313819969808143)	0	0	0	0	0	0	0
ATP6V1C2	0	0	0	0	0	0	0	0	4.71248163543877 (1.62912801254079e-08)	0
ATP7B	0	0	0	0	0	3.42444967266067 (0.000277854535386486)	0	0	2.20618988010029 (0.00015171198227862)	0
ATP8A2	0	0	0	0	0	0	0	0	3.22695644079089 (0.0012176187585204)	0
ATP8B1	0	0	0	0	0	0	0	-6.77367239807072 (0.00206823096227238)	0	0
ATP8B3	0	0	0	0	0	0	0	4.20131290588776 (0.00200644883153507)	0	0
ATP8B4	0	0	0	0	0	0	0	4.78765150785036 (1.09410562118095e-09)	1.92271222947942 (5.98209615124339e-05)	0
ATP8B5P	0	0	0	0	0	5.53969084502242 (0.000586617208788932)	0	0	0	0
ATP9A	1.70392323304595 (1.24847369110507e-06)	0	0	2.02635133123247 (0.00302256450916304)	0	0	0	0	0	0
ATPIF1	0	0	1.81991634344331 (4.93802810203002e-07)	0	0	0	0	1.88786733172192 (0.0071275892471251)	0	0
ATRAID	0	0	1.77220870921828 (0.000248228568467372)	0	0	0	0	0	0	0
ATRNL1	4.67160763715906 (9.55287857523143e-06)	0	0	0	6.5082264427913 (2.39224717314233e-08)	5.28938087855038 (0.00232079414723273)	0	0	3.53154987311175 (0.000442994904334112)	0
AURKA	0	0	0	0	0	0	0	3.5576296017202 (0.00990772458933711)	0	0
AURKAIP1	0	0	1.77219356761833 (0.000183702847379717)	0	0	0	0	0	0	0
AURKB	0	0	0	0	0	0	0	4.57321293381977 (0.000519157923494439)	0	0
AURKC	0	0	0	0	0	6.43665455615025 (0.000230294299976291)	0	0	0	0
AVEN	0	0	1.50083123010503 (0.00497467488954612)	0	0	0	0	0	0	0
AVPR1A	0	0	0	0	0	6.81472817483429 (1.95229769743434e-08)	0	0	0	5.15502494729745 (0.00013808646756339)
AXDND1	0	0	0	0	0	6.36960894606871 (0.00685580200505035)	0	0	0	0
AXL	0	0	0	2.11232254275253 (0.00696757488356823)	0	0	0	0	0	0
AZGP1	0	0	3.85104557246597 (3.71563834660435e-07)	0	0	5.81817508877448 (7.67619970504589e-15)	0	0	-4.61018797115303 (0.000125517540482449)	4.0389278523498 (0.00013873536830094)
AZU1	-4.36814903220317 (0.00823602129899109)	0	0	0	0	0	0	7.1891553916099 (1.90843046313876e-13)	-4.4121795574214 (0.00352119804146031)	0
B3GALNT2P1	0	0	0	0	7.23806043843821 (0.000719882457903311)	7.98359928389732 (0.00139368001622027)	0	0	0	0
B3GALT4	0	0	0	0	0	0	2.71646553388488 (0.00166807839099041)	0	0	0
B3GALT5	3.76932859334378 (0.000275921809611133)	0	0	0	5.12146444656822 (0.000215842670470889)	6.31805222117513 (0.000168594832926964)	0	0	3.01284838771239 (0.00731922945888477)	0
B3GALT6	0	0	0	0	0	3.09641034098339 (0.00444575582532053)	0	0	0	0
B3GALTL	0	0	1.66128872746868 (0.00215336712545954)	0	0	0	0	0	0	0
B3GAT1	0	0	0	0	0	5.45975319965859 (1.14303236882801e-06)	0	0	0	0
B3GNT3	0	0	0	0	0	0	5.18195710775462 (0.000834138479284857)	0	5.90135981417186 (1.7678836996795e-06)	0
B3GNT5	-2.62051484069444 (2.38629147877063e-05)	0	-1.88393088599733 (0.00861259231141142)	0	0	0	0	0	0	0
B3GNT7	-2.06503789084442 (0.00237424930820804)	0	0	0	0	0	0	0	0	0
B3GNT8	0	0	0	0	0	0	0	0	-2.43175984468749 (0.00814368444196945)	0
B4GALNT1	0	0	0	0	0	3.64669899680021 (0.00754590063202984)	0	0	0	0
B4GALNT3	-2.96868295465334 (0.00182318584516654)	0	2.39749661141218 (4.71104607911906e-05)	0	0	0	0	0	-2.48506269613012 (0.00244834881006318)	0
B4GAT1	1.84783671057163 (7.41188694921968e-05)	0	0	0	0	0	0	0	0	0
BAAT	0	0	0	0	0	10.1621517481966 (4.11424503393242e-17)	0	0	0	0
BACH1	0	0	-1.60730903643376 (0.000104214974273467)	0	0	0	0	0	0	0
BACH2	0	0	0	0	0	2.66468280452471 (0.000879944895778104)	0	0	0	0
BAG1	0	0	0	0	1.73608884864466 (0.00669854884841511)	0	0	0	0	0
BAG2	2.87997836963484 (2.35908635991523e-15)	0	0	3.73384946911351 (1.31674164050498e-07)	0	0	0	0	0	0
BAG3	0	0	2.29975226844845 (0.000218195729573753)	0	0	0	0	-4.14029206513941 (0.000243383259381257)	0	0
BAHCC1	0	0	0	0	0	0	0	0	-2.06175664801925 (0.000334838433074317)	0
BAI3	0	0	0	0	0	0	0	0	-4.28005255245847 (6.62371842526869e-05)	0
BAIAP2	0	0	0	0	0	0	0	-6.12457859399307 (0.00780947781269863)	0	0
BAIAP2-AS1	-2.04345937840345 (0.000265077927589185)	0	0	0	0	0	0	0	0	0
BAIAP2L1	-2.33799340179614 (0.000239990554681261)	0	0	-3.38558059624869 (0.00510363658586879)	0	0	0	0	0	0
BAMBI	0	0	0	0	0	0	0	0	1.94139559769345 (0.00228766085220437)	0
BANK1	0	0	0	0	0	0	1.70902995055916 (0.000346479182328145)	0	-2.7187998798869 (0.00217442070305005)	0
BASP1	0	2.951114680533 (0.000132140898972174)	-3.50648030571737 (2.71038841647612e-08)	0	0	0	0	0	0	0
BAX	0	0	0	0	0	0	0	0	-1.57786175398572 (0.000544261498197607)	0
BAZ1A	0	0	0	0	0	0	0	1.6685273832671 (0.0087312822997137)	0	0
BBC3	-2.08238484738537 (0.00297623494957338)	0	0	0	0	0	0	0	-3.07543079938167 (2.3706139855516e-05)	0
BBIP1	0	0	0	0	0	0	0	0	1.52667288096337 (5.74798617727297e-05)	0
BBOX1	0	0	0	0	5.49650402739087 (0.0092703561870476)	0	0	0	0	0
BCAM	0	0	0	0	2.63180602445352 (1.92506729671303e-06)	0	0	-7.05928763793562 (0.00105425821734234)	0	0
BCAN	4.5628611480291 (5.87820855700096e-05)	0	0	0	0	0	0	0	0	0
BCAR4	0	0	0	0	0	0	0	0	7.53751038183067 (1.45312616142375e-10)	0
BCAS1	0	0	0	0	6.07156542351806 (1.35058937920049e-07)	5.12912605880227 (0.00292452092547689)	4.07199417635803 (0.00203437830375266)	0	0	0
BCAS3	0	0	0	0	0	1.56989938950915 (0.00121598139659092)	0	0	0	0
BCAT1	0	0	-3.6763910491009 (6.75158326001545e-08)	0	-2.63736893853757 (0.00365116565009239)	0	0	-3.05708758214608 (0.00370343327730696)	0	0
BCAT2	0	0	0	0	0	2.66524219969902 (0.000826503672184506)	0	0	0	0
BCHE	2.78561447372972 (0.000417254585608736)	0	0	0	0	0	0	0	0	0
BCL11A	2.82944157958871 (0.000104543176399528)	0	0	0	0	0	0	4.77765136211711 (3.06526211909179e-05)	0	0
BCL2A1	-3.14102010291169 (0.00995246145271073)	0	-3.62954042103861 (2.87487409032566e-05)	-8.15554019910899 (0.000306904428836174)	-4.28510014962374 (0.00159695510494293)	0	0	0	-2.76682630289964 (7.43519577225958e-06)	-3.6703943188271 (0.00151169470121959)
BCL2L1	-2.63969079295711 (2.83303937431141e-09)	0	0	0	0	0	0	0	0	0
BCL2L13	0	0	1.53001740351119 (0.000161421886690616)	0	0	0	0	0	0	0
BCL3	0	0	0	0	0	0	0	0	-2.24908993791783 (5.80585586917187e-06)	0
BCL6	0	0	0	0	0	-1.66459663454893 (0.00813995131713766)	0	0	-2.21715460551297 (3.02016815121815e-11)	0
BCL7A	0	0	0	0	2.39421486521854 (0.000581387446389985)	0	0	0	-1.89219481083576 (0.00260582871578314)	0
BCL9L	0	0	0	0	0	0	0	-2.81899051176653	0	0

								(0.00114021907805602)		
BCL9P1	0	0	0	0	0	6.03337679369525 (0.00804466620636718)	0	0	0	0
BCO2	0	0	3.70483853148551 (1.59760759150411e-09)	0	0	0	0	0	0	0
BCYRN1	1.77271469816623 (0.000278672838423171)	0	0	2.86609479163982 (0.000741497643744576)	0	0	0	0	0	2.59803499607989 (0.00260232095296459)
BDH1	0	0	1.94819907453614 (0.00506672305630251)	0	0	3.5784399349103 (2.48388790041207e-05)	0	3.04410278147951 (0.00812020338841533)	-2.38177082236807 (0.00460131211825413)	0
BDKRB1	0	0	0	0	0	6.62972050443079 (0.00300730370188095)	0	0	0	0
BDKRB2	2.51977080532373 (6.19195378491207e-06)	0	0	0	0	0	0	0	0	3.70704857567432 (6.08269511670374e-05)
BDNF	0	0	3.31159142719439 (0.000797366458781437)	0	0	0	0	0	0	0
BDP1P	0	0	0	0	0	6.26733469182352 (0.00672450885589941)	0	0	0	0
BEND3P1	0	0	0	0	4.98683386605517 (0.00341591730924816)	5.82875593939785 (0.00112550903254448)	0	0	0	0
BEND4	0	0	0	0	0	5.17676177347296 (0.000446289803310407)	3.656035634477 (0.0011136700670233)	0	0	0
BEND5	2.17106594435973 (0.004797715064939)	0	0	0	0	0	0	0	0	0
BEST1	-1.91044371593898 (0.000316278801963142)	0	-2.73675232878503 (2.8226895284822e-05)	0	0	0	0	0	-1.57807891174254 (0.00807297871272872)	0
BEST3	0	0	0	0	0	7.98359928389732 (0.00139368001622027)	0	0	0	0
BEST4	0	0	0	0	0	4.05441918629263 (0.00582957282591556)	0	0	0	0
BET1	0	0	0	0	0	0	0	0	2.39273936056181 (2.16676398910994e-06)	0
BEX1	3.54365463283369 (0.00682992595675982)	0	0	0	0	0	0	0	0	0
BGN	-2.68485995170006 (2.31972117310755e-16)	-6.37071407744745 (0.00279062927940986)	0	-2.3374886498767 (0.000234491991007688)	0	0	0	-3.56031930887231 (1.34927229308152e-06)	0	-1.79850375624879 (0.00410165206015945)
BHLHB9	0	3.69125186169821 (0.00157298512494928)	0	0	0	0	0	0	0	0
BHLHE22	0	0	0	0	0	0	0	0	-4.47563457909014 (0.000482513755541162)	0
BHLHE40	-1.90688305877558 (1.43694539830586e-05)	0	-1.65149980844329 (0.000312663379907622)	0	0	0	0	0	0	0
BHMT	0	0	0	0	0	9.39378299831446 (7.33504372459922e-18)	0	0	0	0
BHMT2	3.25036871881801 (1.8004430185097e-08)	0	0	3.51409779821917 (0.000904578722570582)	3.14691073757128 (0.00101646990042893)	4.9075132013503 (2.77473638123483e-14)	0	0	0	0
BIN2	-2.54673110041612 (2.8231068583291e-05)	0	-2.01842464264911 (0.000727256701639585)	0	0	0	0	2.5718315343596 (0.00107583637296607)	0	0
BIRC3	0	0	-2.4651714418866 (2.40221114482193e-05)	0	0	0	0	0	-2.25754038609383 (2.02966105930998e-05)	0
BIRC7	0	0	0	0	0	0	0	0	5.83036420897114 (5.51029725108466e-07)	0
BLNK	0	0	0	0	0	3.42511129917942 (7.67284493317491e-06)	0	0	1.66780053087313 (0.00926814379631917)	0
BLVRA	0	0	0	0	0	0	0	0	-1.57309834664257 (0.000318457951413012)	0
BLVRB	0	0	0	0	0	0	0	2.96903364657298 (0.000230290691923475)	-1.80341218578464 (0.000733474690682333)	0
BLZF1	0	2.67670926994497 (0.00718097577214902)	0	0	0	0	0	0	0	0
BMF	0	0	-2.35677961928053 (0.00890606067014589)	0	0	0	0	0	0	0
BMP1	0	0	0	0	0	0	0	0	3.56297506846066 (4.98457356424698e-10)	0
BMP10	0	0	0	0	0	7.98023099168391 (6.71142805229064e-09)	0	0	0	0
BMP2	-2.6255568386866 (1.09647210499129e-05)	0	-3.36675846232805 (3.50328533937194e-05)	0	0	0	0	0	0	0
BMP2K	0	0	0	0	0	0	0	2.52516584427349 (0.000209091471133101)	0	0
BMP3	6.27159970805219 (3.55232415816039e-14)	0	0	0	0	5.39880248274019 (0.000294314420658247)	0	0	0	0
BMP4	2.73518139418562 (6.83595536650755e-06)	0	0	0	0	0	0	0	0	0
BMP5	-2.98230221295416 (7.8414464566278e-05)	0	-2.68586190469822 (0.00205281740327713)	0	0	0	0	0	0	0
BMP6	-3.23625019852328 (6.92314079127919e-05)	0	-2.16401833262569 (0.00421068658242579)	0	0	0	0	0	0	0
BMP7	0	0	0	0	0	0	0	0	5.25374797188651 (1.0070240990631e-06)	0
BMPR1A	2.23484015033491 (1.12719355276002e-11)	0	0	2.3723650367339 (0.00086399854692256)	0	0	0	-5.88660805582484 (0.00752541224696151)	0	0
BMPR1B	0	0	0	0	3.44838508762889 (0.00710009651973098)	0	0	0	0	0
BMPR2	0	0	0	0	-1.55511490672177 (0.0085877582263201)	-1.73274111102968 (0.000138297182651116)	0	-3.1460172880835 (4.84121049718643e-05)	0	0
BNC1	0	0	0	0	0	0	0	0	0	5.63781727312976 (0.00172646000645547)
BNC2	2.66820863456632 (2.68948882002546e-15)	0	0	3.20701793142657 (3.02079612047004e-06)	0	0	0	-6.21502917387238 (0.00470376686239581)	0	0
BNIP3	0	0	0	0	0	1.51690771739483 (0.00842621564198753)	0	0	0	0
BNIPL	0	0	0	0	0	0	0	0	0	4.22055743351498 (0.00545537586407361)
BOC	4.00190172490538 (9.10072717709312e-14)	0	0	3.0551599731148 (0.0013850855523249)	2.24669653833109 (0.00471257517435121)	0	0	0	0	2.67147403382544 (0.00673819197980877)
BOK	0	0	0	0	0	3.05670401242509 (0.00135848049872011)	0	0	0	0
BPGM	0	0	0	0	0	0	0	2.47049367439898 (0.0047299588237975)	2.59590686987222 (7.74817035560081e-06)	0
BPHL	0	0	0	0	0	2.52910536860923 (0.00134811680143651)	0	0	0	0
BPI	0	0	0	0	0	0	0	6.3333868045011 (1.62353138972853e-11)	-3.66545552363494 (0.00492958018196804)	0
BPIFB1	0	0	0	0	0	0	3.96349558870131 (0.000258068030301896)	0	0	0
BRCA2	0	0	0	0	0	0	0	2.68850440847727 (0.00792884847832185)	0	0
BRD7P4	0	0	-2.93182189993112 (0.00854114078339695)	0	0	0	0	0	0	0
BRI3	-1.60512984899911 (6.50953208081501e-05)	0	0	0	0	0	0	0	0	0
BRIP1	0	0	0	0	0	0	0	3.77052157611313 (0.00727578538729606)	0	0
BRSK2	2.91444348463681 (0.00392299730579938)	0	0	0	4.7974869835292 (1.50704432376824e-05)	4.88089026138494 (0.000156578358209909)	0	0	0	0
BSCL2	0	0	0	0	0	0	2.54129624694404 (0.0015982725412384)	0	0	0
BSDC1	0	0	0	0	0	0	0	1.74820476342411 (0.00741554541668538)	0	0
BSG	0	0	2.05034325045838 (6.85128128699833e-11)	0	0	0	0	1.95717989497417 (0.00271192265226629)	0	0
BSN	0	0	0	0	3.7207230039001 (0.00327806612372983)	4.88769800856701 (2.40398785014005e-05)	0	0	0	0
BSND	0	0	0	0	7.20972347782504 (8.33192697222776e-07)	6.19953425757015 (0.000171657730975112)	0	0	0	0
BST1	0	0	0	0	0	0	0	3.48868966653748 (0.00214488894252439)	0	0
BTBD19	-1.56425662786463 (0.00825144111219452)	0	0	0	0	0	0	0	0	0
BTBD3	0	0	0	0	0	0	0	0	2.73226189798994 (1.00906612038561e-14)	0
BTBD9	0	0	0	-2.42482533970534 (0.00550532835174944)	0	0	0	0	0	0
BTC	2.74711991234719 (0.00135505732767584)	0	0	0	0	0	0	0	0	0
BTG2	0	0	0	0	0	-1.56090801166534 (0.0019088397442748)	0	0	0	0
BTG3	-2.14615767011939 (0.00048629395587718)	0	0	0	0	0	0	0	0	0
BTK	0	0	-3.0957950944776 (0.000323466781433201)	0	0	0	0	2.53063883362733 (0.00385876938570989)	0	0
BTLA	0	0	0	0	0	7.17531063868961 (0.0016444463099887)	3.91058615958045 (0.00334129545067239)	0	0	0
BTN2A2	0	0	0	0	0	0	0	0	-1.9378633853377 (0.00287375217925545)	0
BTN3A2	0	0	-1.5143207534807 (0.00836309702828604)	0	0	0	0	0	0	0
BTNL9	0	0	0	0	0	0	0	0	-4.17954665588247 (0.000158415847437886)	0
BUB1	-4.08989551597285 (0.00467036172854362)	0	0	0	0	0	1.55661690401002 (0.00822467738145251)	4.18491948388939 (0.000235952198332927)	0	0

BUB1B	0	0	0	0	0	0	0	4.63779088680566 (4.21528292177475e-05)	0	0
BVES	3.05720461048097 (2.98777621578802e-08)	0	3.40147738268363 (4.5532772028218e-08)	3.44928967839533 (0.000957025510342368)	0	0	0	0	0	0
BZW2	0	0	2.33128303040132 (1.77401604689555e-09)	0	0	0	0	0	2.68918852410361 (2.44461927011174e-13)	0
C10orf10	0	0	0	0	0	0	0	-2.97272102792956 (0.00243669008116271)	0	0
C10orf11	0	3.81838378142234 (0.00464456310771372)	0	0	0	0	0	0	0	0
C10orf53	0	0	0	0	0	8.08791186690628 (0.00117930857036654)	0	0	0	0
C10orf71	0	0	9.20147678553052 (6.16629340482862e-17)	0	5.6403872762919 (0.00641340890155407)	7.69214768702483 (1.03539151705468e-05)	0	0	0	0
C10orf82	0	0	0	0	0	6.93037023825997 (0.00993412460458525)	0	0	0	0
C10orf90	0	0	0	0	0	6.93037023825997 (0.00993412460458525)	0	0	0	0
C11orf21	0	0	0	0	0	0	0	3.64759762167621 (0.00231941901782632)	0	0
C11orf39	0	0	0	0	0	6.1035404149236 (0.00986674034872968)	0	0	0	0
C11orf63	0	0	0	0	0	0	1.5003566196177 (0.00518140397381202)	0	0	0
C11orf84	0	0	0	0	0	0	1.70318900511523 (0.00200118594087814)	0	0	0
C11orf87	0	0	0	0	5.74834683433224 (0.00104342290984836)	0	0	0	0	0
C11orf96	0	0	0	0	0	0	0	-5.42319335605969 (0.00149074865189181)	-1.73170186483718 (0.00424884147638536)	0
C12orf49	-2.28923078432942 (1.68687543491434e-07)	0	-1.5899180724817 (0.0014039814864226)	-4.05383450748138 (0.000237081856396663)	0	0	0	0	-1.78529502895449 (5.46535605965301e-07)	0
C12orf75	0	0	0	0	4.62310259050612 (2.15268990591047e-07)	0	2.13677346957639 (0.000646798393612072)	0	4.37618274028569 (1.3563190938672e-08)	0
C12orf77	0	0	0	0	6.26356497464546 (0.00580250986142461)	0	0	0	0	0
C14orf105	0	0	0	0	7.62491429632298 (4.52063054310171e-07)	7.12012884737093 (5.14607517255161e-06)	0	0	0	0
C14orf132	0	0	-2.4609770140361 (0.000409899600907259)	2.37116430114868 (0.0082199905390101)	0	0	0	-6.02394883301136 (0.00818107441754189)	0	0
C14orf180	0	0	7.67436781903853 (9.06173309779291e-13)	0	0	0	0	0	0	0
C14orf37	0	0	2.10621892794919 (0.0016579724288579)	0	0	0	0	0	0	0
C14orf80	0	0	0	0	0	3.5181911503458 (0.006472213729338)	0	0	0	0
C15orf41	0	0	3.69365828971442 (3.434322604365e-10)	0	0	0	0	0	0	0
C15orf52	1.81015621535729 (0.000395319235185154)	0	2.3246171367087 (4.12705980271616e-05)	0	0	0	0	0	0	0
C15orf53	0	0	0	0	0	5.42126514981206 (0.00710912307001227)	0	0	0	0
C15orf57	0	0	0	0	1.83096737942217 (0.0072817349450861)	0	0	0	0	0
C15orf59	0	0	5.8899098338766 (1.46562693909613e-13)	0	0	0	0	0	0	0
C15orf61	0	5.17084777683631 (0.000760323902957513)	0	0	0	0	0	0	0	0
C15orf62	0	0	0	0	3.4387732843984 (0.00531490347625091)	0	0	0	0	0
C16orf45	1.88005500991898 (9.07985006201924e-05)	0	1.52326089630011 (0.00549914233324827)	2.62311195595581 (0.00496261188962231)	0	0	0	0	-2.84418578207786 (0.000994922012559223)	0
C16orf74	0	0	0	0	0	0	0	0	2.94333294167333 (0.00496199224572369)	0
C16orf89	0	0	-3.80885808337841 (0.000534064483467016)	0	0	0	0	0	-4.25207587331377 (1.85585928324253e-05)	0
C17orf102	0	0	0	0	0	6.94851805125464 (0.00137262988708502)	0	0	0	0
C17orf104	0	0	0	0	0	5.5370398122934 (0.000490994435106585)	0	0	0	0
C17orf105	0	0	0	0	0	4.784914070237 (0.00818226887490094)	0	0	0	0
C17orf50	0	0	0	0	6.47559966567641 (0.0042482603748159)	0	0	0	0	0
C17orf53	0	0	3.57855457774582 (0.00482165196959481)	0	0	5.50762354648228 (0.0010748350867576)	0	0	0	0
C17orf58	0	0	0	0	0	0	0	0	2.69984913087272 (2.04412697478572e-05)	0
C18orf54	0	0	-2.93742618529034 (0.00195508942544909)	0	0	0	0	0	0	0
C19orf12	0	0	2.14248197749798 (6.79202436366105e-05)	0	0	2.90388326135395 (0.000374898556014616)	0	0	0	0
C19orf26	0	0	4.34969574328794 (0.00360880700371375)	0	0	0	0	0	0	0
C19orf33	-3.87643732394737 (0.00490852958559789)	0	0	0	0	0	0	0	0	0
C19orf38	0	0	0	0	0	0	0	3.63074106210941 (0.00391562005192703)	0	0
C19orf47	0	0	3.15157329652542 (6.95056880866291e-08)	0	0	0	0	0	0	0
C19orf66	0	0	0	0	0	1.62187507563238 (0.00495293042989824)	0	0	0	0
C19orf68	0	0	0	0	0	0	0	0	-1.97619437225878 (0.000896624846923321)	0
C19orf80	0	0	0	0	0	6.69062257341677 (1.60386606000125e-07)	0	0	0	0
C1GALT1	0	0	-1.81544687488419 (0.00064412558376749)	0	0	0	0	0	0	0
C1QL1	0	0	0	0	6.53007003761723 (0.00410227708867015)	0	0	0	0	0
C1QTNF1	0	0	3.14884774463546 (2.70189987908451e-09)	0	0	0	0	0	1.76273048258076 (9.52442904517977e-05)	0
C1QTNF2	0	0	0	0	0	5.48645675180696 (0.00685374295230385)	0	0	0	0
C1QTNF3	4.28045368658426 (9.24771775056574e-06)	0	0	3.97376065906573 (0.00850851732576829)	3.68024074547123 (0.00441218755864632)	3.54885130679763 (0.00300020175347473)	0	0	0	0
C1QTNF6	0	0	0	0	0	0	0	0	3.27701022910927 (6.17636086110288e-08)	0
C1QTNF7	2.12433470177183 (0.000168060750054767)	0	0	0	0	0	0	0	1.73556214441985 (0.00462325424037624)	0
C1R	0	0	0	0	0	0	0	-3.76745877752328 (2.30268233939515e-07)	-1.70034001945292 (0.000985442181415364)	0
C1RL	0	0	-1.56833873744563 (0.00104193178883533)	0	0	0	0	0	-2.07767395752276 (0.000588784957152361)	0
C1S	0	0	0	0	0	0	0	-4.41053770533834 (4.91756832734092e-09)	-1.5896327088222 (0.00579924310789388)	0
C1orf111	0	0	0	0	4.41320387610493 (0.005024260097617)	0	0	0	0	0
C1orf115	0	0	0	0	0	0	0	-6.65772718432411 (0.00242117380870634)	2.89040279947134 (1.71958713593382e-16)	0
C1orf116	-5.48883714676839 (2.20514982475854e-14)	0	-6.36255562444605 (3.14253010364258e-14)	-3.85834705167932 (0.000190440113366974)	0	0	0	-5.12382180849768 (0.002065113753761)	-4.50690272652864 (2.04303360753555e-09)	0
C1orf147	0	0	0	0	0	4.36773599856587 (0.000196813107658614)	0	0	0	0
C1orf159	0	4.38936539018344 (0.0022397317966181)	0	0	3.33068695644865 (0.00372284435467092)	0	0	0	0	0
C1orf162	0	0	-3.61264004600123 (2.97391618195192e-05)	0	0	0	0	0	-2.07483379865607 (0.00174322357592355)	0
C1orf186	0	0	0	0	5.70120859955342 (0.00934464482525645)	0	4.21357629098772 (0.0043402037715534)	0	3.65842110606534 (0.00516423204844135)	0
C1orf21	0	0	0	1.73903369609804 (0.00559670976025167)	0	0	0	-3.9857281875243 (0.00108821558642791)	0	0
C1orf226	3.10972681295738 (0.00177301374635012)	0	0	0	4.161028966803 (0.00666192444174461)	6.1522535624198 (3.84346803720906e-08)	3.7530670790724 (0.000234390606269673)	0	3.10255584152534 (0.000842776119198322)	0
C1orf61	0	0	0	0	7.4022293925345 (0.000231644832239065)	8.42113661635808 (3.72907155317198e-07)	6.34193920663452 (0.00599435978464417)	0	0	0
C1orf68	0	0	0	0	0	0	0	0	0	8.94392090478443 (5.53422200385399e-10)
C1orf95	5.14564939116202 (5.08394466995994e-11)	0	0	0	4.52561759831542 (0.000487987903137221)	4.5003665841952 (0.00131564350693705)	0	0	0	0
C2	0	0	0	0	-4.22259617417993 (0.00473118281290882)	2.15516141248091 (0.000876419716487968)	0	-6.02084918877386 (0.00841631697168559)	0	0
C20orf166-AS1	0	0	0	0	5.6403872762919 (0.00641340890155407)	0	0	0	0	0
C20orf85	0	0	0	0	0	0	3.65098291709442 (0.00366872746765611)	0	0	0
C21orf58	-3.52741567187206 (0.00777788953731526)	0	0	0	0	0	0	3.67829209041041 (0.000405530728850281)	0	0
C21orf62	0	0	0	0	6.01984881389889 (0.00219286599955368)	6.15285542182167 (0.00195058435897731)	0	0	0	0
C21orf91	0	0	0	0	0	0	0	0	1.59599933442333	0

									(0.00757232301407204)	
C22orf34	-1.72924798516003 (0.00771352549913351)	0	0	0	0	0	0	0	-1.76107408865142 (0.00214111894333735)	0
C22orf39	0	0	0	2.42699367153966 (0.00676993560346622)	0	0	0	0	0	0
C2CD4A	0	0	0	0	0	6.75596666636941 (3.28530428656383e-06)	0	0	0	0
C2orf15	-4.24417208024991 (0.00873606019441646)	0	0	0	0	0	0	0	0	0
C2orf16	0	0	0	0	0	5.46153984730492 (0.000118902113820578)	0	0	0	0
C2orf40	4.14134066784858 (2.61233474649648e-05)	0	0	0	0	0	0	0	0	0
C2orf50	0	0	0	0	0	4.80574838762096 (0.00748051134682206)	0	0	0	0
C2orf70	0	0	0	0	0	6.8747981313601 (0.00366426386871548)	0	0	0	0
C2orf71	0	0	0	0	4.31392809972519 (0.00899687883097422)	5.32286883637825 (0.00138314258301079)	0	0	0	0
C2orf72	0	0	0	0	0	6.45881346598304 (6.511080759087e-05)	0	0	5.72402889933731 (4.48989219396842e-11)	0
C2orf76	0	0	0	0	0	0	2.17605705676091 (0.00784929069901156)	0	0	0
C2orf88	2.87796525972566 (5.86816431733306e-06)	0	0	0	0	0	0	0	0	0
C2orf91	0	0	0	0	0	7.46102324576142 (0.000708109083945895)	5.33052624256537 (0.00937150101283959)	0	0	0
C3	0	-7.06548409332372 (0.000991059153060849)	0	0	0	3.16611319805487 (2.45535090003549e-05)	0	-3.81540566456146 (4.76090977533342e-07)	0	0
C3AR1	0	0	-3.00963486218975 (0.000330250335524663)	0	0	0	0	0	0	0
C3P1	0	0	0	0	0	9.41555705759708 (8.22772834563655e-13)	0	0	0	0
C3orf18	0	0	3.02654814090117 (0.000153432179771774)	0	0	0	0	0	0	0
C3orf36	0	0	0	0	0	4.02242486493943 (0.00552487581392904)	0	0	0	0
C3orf52	0	3.18661406050145 (0.00656277168243863)	0	0	0	0	0	0	0	0
C3orf70	4.3585137468512 (1.04709099229309e-12)	0	0	4.14467358657197 (8.1753400659729e-05)	0	0	0	0	0	0
C4A	1.92674047514939 (0.00212243899322598)	0	0	0	2.6286312862332 (0.0029253848975395)	4.76700563251134 (3.06900710831679e-07)	0	0	0	0
C4B	0	0	0	0	0	4.45105141059737 (4.15550673478086e-05)	0	0	0	0
C4BPA	-6.1357706522794 (0.00389429661912004)	0	0	0	0	4.25953241296239 (0.000522147360110384)	0	0	-6.09189713087963 (0.000391693878833426)	0
C4BPB	0	0	0	0	0	6.32470459799504 (1.02599995476974e-08)	0	0	2.98039025763677 (0.00343448662793526)	0
C4orf26	0	0	0	0	0	0	0	0	4.03537491690226 (0.00120103550397024)	0
C4orf36	0	0	0	0	0	0	0	0	2.55390680253988 (0.0001097841392585)	0
C5	0	0	0	0	0	4.70836309379931 (2.6880418589659e-09)	0	0	0	0
C5AR1	-2.48580481113637 (0.000144512499792164)	0	-3.68755454826062 (3.64582657340697e-08)	0	-2.91295312209996 (0.00209872900897243)	0	0	0	-2.8648250236533 (1.507087427488e-08)	0
C5AR2	-2.5794318611074 (0.00666142003384991)	0	-2.75820475428453 (0.00495344460664537)	0	0	0	0	0	0	0
C5orf15	0	0	0	-3.09931963640157 (0.0039400455400457)	0	0	0	0	0	0
C5orf17	0	0	0	0	0	0	0	0	3.98724538173648 (0.00282155753469964)	0
C5orf38	-4.87388812154752 (0.000805497918808252)	0	0	0	0	0	0	0	0	0
C5orf46	0	0	0	0	0	0	0	0	5.42716776081478 (0.000121308102649408)	0
C5orf58	-4.48871232124663 (0.000882184274203121)	0	0	0	0	0	0	0	-3.96308933436055 (0.000140546707543907)	0
C5orf60	0	0	0	0	0	6.80030666102153 (0.00242138477854474)	0	0	0	0
C5orf66-AS2	0	0	0	0	0	5.92383765599611 (0.00893443873901692)	0	0	0	0
C6	0	0	3.61569614702838 (0.000863434482190831)	0	0	6.75392891430631 (6.37982838224271e-14)	0	0	0	0
C6orf132	-3.5234916664535 (4.56711056489679e-05)	0	0	0	0	0	0	0	0	0
C6orf15	0	0	0	0	0	6.10213009384402 (0.00755053791667713)	0	0	0	0
C6orf183	0	0	0	0	0	0	0	0	5.28375968521666 (3.88381901236529e-05)	0
C6orf223	0	0	0	0	0	5.12170686695412 (0.0054834618697054)	0	0	0	0
C6orf57	0	0	2.48175002760777 (0.00742848074602927)	0	0	0	0	0	0	0
C7	0	-4.97666215163257 (0.00170598468923655)	0	0	0	0	0	-3.68047694417868 (1.01449613648094e-06)	0	-1.9861645306244 (0.00422838131133738)
C7orf26	0	0	0	0	0	0	0	0	-1.66453351620468 (0.00654821027119972)	0
C7orf50	0	0	0	0	0	0	0	0	-1.64179813151745 (0.000139291507526258)	0
C7orf55	0	0	4.66266976751686 (0.000516770903829417)	0	0	0	0	0	0	0
C7orf65	0	0	0	0	7.20697900913001 (0.00515462419084721)	0	0	0	0	0
C8A	0	0	0	0	0	7.58420853429391 (5.5159266281815e-08)	0	0	0	0
C8B	0	0	0	0	0	8.75208831251754 (1.03766166533933e-13)	0	0	0	0
C8G	0	0	0	0	0	8.05505493090693 (1.70357659255407e-09)	0	0	0	0
C8orf4	0	0	0	0	0	0	0	-6.58162683040524 (7.44367760684955e-05)	0	-3.06368361723833 (0.000330025748115928)
C8orf48	0	0	0	0	0	5.31003535832784 (0.00948142022058294)	0	0	0	0
C8orf58	0	0	0	0	0	0	0	0	1.59135920951386 (0.00398642554119873)	0
C8orf74	0	0	0	0	0	7.13036380369488 (0.00128033179179201)	0	0	0	0
C8orf82	0	0	2.19593457295871 (0.00270443337509004)	0	0	0	0	0	0	0
C8orf88	2.52341723949391 (0.000106740190355005)	0	0	0	0	0	0	0	0	0
C9	0	0	0	0	0	11.0850030316945 (1.76994245257755e-16)	0	0	0	0
C9orf156	0	0	-2.77544744414819 (0.00182720497265254)	0	0	0	0	0	0	0
C9orf172	0	0	2.09877424921857 (0.00215301085941336)	0	0	2.94181714684993 (0.00119297865344793)	0	0	0	0
C9orf50	0	8.83438846949353 (1.89611992709485e-06)	0	0	0	0	0	0	0	0
C9orf72	0	0	-2.59659867559622 (1.56263631711804e-05)	0	-2.61248844970108 (0.00258251271869917)	0	0	0	0	0
C9orf89	0	0	0	0	0	0	0	0	-1.50369084054968 (0.00754230624552459)	0
C9orf91	0	0	-2.38043884836944 (0.00122753252182324)	0	0	0	0	0	0	0
CA1	-5.64042819666872 (4.3324401952739e-06)	0	0	-6.84206724565689 (0.00396307683366781)	0	0	0	6.10532858904059 (1.07238372637419e-09)	-3.07423244575084 (0.000234036619413445)	0
CA10	0	0	0	0	0	0	0	0	4.63201589297273 (0.00251167626566456)	0
CA11	3.43600455918208 (3.227949451693e-06)	0	2.48427109095734 (0.00963243739758263)	4.45853946368855 (0.000368743281536983)	0	0	1.83244432976469 (0.00350262458874245)	0	0	0
CA12	0	0	-4.44594766664839 (1.48933424252847e-05)	0	0	0	0	0	0	3.88348957591857 (0.000299082592799337)
CA13	0	0	0	0	0	0	0	0	-2.35417534453006 (0.00830387244505325)	0
CA14	4.33919739631934 (0.00100703322484616)	0	0	0	5.88438457059004 (0.00152361356212711)	0	0	0	0	0
CA2	-2.25618055199556 (0.00157921776630959)	0	0	0	0	1.93162448968302 (0.006041267670391)	0	5.27829121063729 (6.93456217178054e-11)	-1.66299515659899 (0.00517499143757363)	0
CA3	-5.61624019280405 (0.000224708982178621)	0	-4.78058666660851 (1.77356053761404e-05)	0	0	-3.77102796979531 (0.00512064572638345)	0	0	-4.78466824013984 (5.58257895720139e-07)	-4.41982065331833 (0.00496208546586766)
CA4	0	0	0	0	0	0	0	0	-3.70019779086331 (0.000354667940504715)	0
CA5A	0	0	0	0	0	7.78984161434381 (9.01910931953511e-07)	0	0	0	0
CA8	0	5.80729834473724 (0.000377992282689075)	3.17468338871154 (0.00113632545594015)	0	5.04194520073331 (1.40610942647774e-05)	0	0	0	2.86309674596281 (0.000793822697545871)	0
CAB39L	2.15182442987986 (0.000175958102914103)	0	0	3.20291382378796 (0.000574787018415623)	0	0	0	0	2.09523592507355 (0.00392685418691842)	0

CABP1	0	0	4.04824849085052 (0.0044875596363606)	0	0	0	0	0	0	0
CACNA1B	3.88682807271686 (0.00730241125432677)	0	0	0	0	5.56682818678313 (0.00717130421196283)	0	0	0	0
CACNA1C	1.77775909041588 (0.000485302057409772)	0	1.71747241520163 (0.00159347530064843)	0	0	0	0	-7.25670400685786 (0.00123215384214513)	0	0
CACNA1C-AS4	2.15413506532598 (0.00104839945297573)	0	0	0	0	0	0	0	0	0
CACNA1C-IT2	0	0	4.68512360132851 (0.00702098963599369)	0	0	0	0	0	0	0
CACNA1D	-1.914249724853 (0.00535250515536561)	0	-4.37975126930278 (2.19071075635857e-06)	0	0	0	0	-6.01349034786325 (0.00859521187724418)	-4.38686486143537 (5.89251467850477e-09)	-7.35112144128535 (0.00124205811191183)
CACNA1E	0	0	0	0	0	5.8443758987475 (0.00201421599780541)	0	0	0	0
CACNA1F	0	0	0	0	0	0	4.05493894506703 (0.00670350783742512)	0	0	0
CACNA1H	2.73096224588075 (7.65579100320737e-08)	0	0	3.713315496566 (2.26779346715476e-05)	0	0	0	0	0	0
CACNA1I	0	0	0	0	0	5.48080033973263 (0.00238334700473465)	0	0	0	0
CACNA2D1	2.33252064718698 (9.28685840636565e-10)	0	0	0	0	0	0	0	0	0
CACNA2D2	-2.64919257329049 (0.000244028738157024)	0	-2.33699023870685 (0.00178779398361642)	0	0	0	0	-6.79397280783828 (0.00280539412043941)	-4.89928931113217 (1.70510805434758e-09)	-4.68652934500012 (0.000882082471340551)
CACNA2D3	0	0	2.78303888630074 (0.00949991560010525)	0	0	0	0	0	0	0
CACNB1	0	0	0	0	0	0	0	0	-2.13313764811307 (0.000264621184539479)	0
CACNB2	2.07606624741644 (9.55673359077307e-05)	0	0	0	0	0	0	0	0	0
CACNG8	0	0	0	0	7.40844190398104 (0.00013825306335216)	8.573559012602 (0.00498034638872196)	6.6102308371872 (0.000648321127740906)	0	0	0
CACYBP	0	2.1621234146282 (0.00954163366999798)	0	0	0	0	2.07941243924484 (0.00304062422090048)	0	0	0
CADM3	3.39886882113283 (1.94513994346765e-05)	0	0	3.69498707767379 (0.00503277693318072)	0	0	0	0	2.711666462945 (0.00133335059629985)	0
CADM3-AS1	2.7187618493858 (0.00530634109227239)	0	0	0	0	5.00566077696873 (0.00146497299574833)	2.84626974904551 (0.008346657496153)	0	0	0
CADM4	3.71734694722453 (4.08095660885407e-07)	0	2.29594297179883 (0.00568026951553482)	0	0	0	0	0	0	0
CADPS	3.91066755655793 (1.09572440780645e-05)	0	3.35438740051893 (0.00138036340523508)	0	5.17615307580474 (0.000439370560092024)	4.92804339280428 (0.00236732648298508)	3.69857856028948 (0.00834423741338009)	0	0	0
CADPS2	0	2.33960902381377 (0.00189028336435265)	0	0	-2.80165052402508 (0.000992121190741911)	0	0	-7.13238785529755 (0.000923660100463029)	-1.556428629043 (2.41238437848799e-05)	-2.44307532539356 (0.00171259919728064)
CALB2	5.75777658830602 (2.58865611809072e-06)	0	0	0	0	0	0	0	0	0
CALCA	0	0	0	0	0	5.45865528084486 (0.000990346006206913)	0	0	0	0
CALCRL	-2.21120474625257 (1.16529997763221e-05)	0	0	0	-3.66698699390367 (7.35170625193598e-05)	-2.7205019275396 (0.00019436348894235)	0	-8.55017592802464 (0.00011686620876771)	-2.45814480656832 (2.38046514455572e-07)	0
CALD1	2.63422938651583 (2.17195505239379e-13)	0	0	3.87298035639909 (1.24203671283386e-09)	0	0	0	-2.62358215404421 (9.85064154229098e-05)	0	0
CALML5	0	0	0	0	0	0	0	0	0	7.86049920286229 (6.54818088766905e-06)
CALN1	0	0	0	0	6.43563438225718 (0.0061813268135828)	8.22209589362692 (9.9346026177268e-08)	0	0	0	0
CALU	0	0	0	0	-1.58822655239962 (0.00487244208882233)	-1.95778111583646 (0.000313326079262376)	0	-2.5788532939077 (0.000160589255560068)	0	0
CAMK2A	0	0	4.42355371008121 (6.26703919181057e-05)	0	0	4.74564791043412 (0.000685150983834021)	0	0	0	0
CAMK2B	0	0	8.14939438387484 (6.00907853854638e-18)	0	0	5.49209699396875 (0.000513590938622266)	0	0	0	0
CAMK2D	0	0	0	0	0	0	0	-2.7351213506233 (0.00124343747779557)	0	0
CAMK2G	1.867497520813 (2.55011034484406e-06)	0	0	0	0	0	0	0	0	0
CAMK2N1	0	0	0	0	0	0	0	-4.56474371058998 (0.000885356747444867)	0	0
CAMP	0	0	0	0	0	0	0	6.37877109565716 (0.000329367881492896)	0	0
CAMSAP2	0	0	0	0	0	-1.56489105936918 (0.00275942979098283)	0	-3.80650647380845 (0.000119551089869124)	0	0
CAMSAP3	0	0	0	0	0	2.8308506579416 (0.00313967844934735)	0	0	0	0
CAND2	0	0	2.60869268127696 (7.71740573192606e-05)	0	0	0	0	0	-1.97968894573919 (0.00629188927716776)	0
CAP1P2	0	0	0	0	0	5.2744471697276 (0.00785895513196325)	0	0	0	0
CAP2	3.8148239053996 (6.31318396233248e-12)	0	4.01407381341639 (5.08564436077591e-09)	5.68626083303371 (1.4836460395391e-11)	0	0	0	0	2.67654033170335 (7.78374815283931e-06)	0
CAPG	-2.31105961753059 (4.52049902625415e-05)	0	-3.27746816630435 (1.74129445668041e-05)	0	0	-2.52995305130081 (0.00562438848986049)	0	0	0	0
CAPN10-AS1	0	0	0	0	0	4.24711554919008 (0.0013217879829281)	0	0	0	0
CAPN12	0	0	0	0	4.68333823447069 (0.000757663618669535)	4.81751198192888 (1.00046561161042e-05)	0	0	0	0
CAPN13	0	0	0	0	0	0	1.9453919439005 (0.00120105906039647)	0	-3.872477591387 (0.00530102991629875)	0
CAPN2	0	0	0	0	0	-1.73583678676345 (7.54503056489156e-06)	0	-2.80225990052111 (2.43291006108887e-05)	0	0
CAPN5	0	3.31314928375774 (0.00928990738359385)	0	0	0	2.4087440593302 (0.000349945870317484)	0	0	0	0
CAPN6	3.63056507453607 (0.00888244642077792)	0	0	0	5.5283249375643 (0.00856437994247807)	0	0	0	9.44361496148407 (1.00572521146115e-22)	0
CAPN8	-3.13138613679616 (0.000281739053461716)	0	-4.8374807014792 (2.55239949404872e-06)	-6.4435436826841 (0.00464685479220134)	0	0	0	0	-5.43036745508112 (2.68769542493577e-09)	0
CAPNS2	0	0	0	0	0	0	0	0	0	6.7365139721226 (0.00034639586846839)
CAPRIN2	0	0	0	0	0	0	0	4.18839719224735 (1.51205132923858e-10)	0	0
CAPZA1	0	0	-1.85522714249045 (0.00204245487553537)	0	-2.015292444136 (0.00727277273726657)	0	0	0	0	0
CARD10	0	0	0	0	0	1.98060002436546 (0.0057764143688714)	0	0	0	0
CARD14	0	0	0	0	4.21193109362117 (0.00242144123227252)	0	0	0	0	0
CARD6	0	0	0	0	-2.17862421676202 (0.00531879436780626)	0	0	0	0	0
CARKD	0	0	0	0	0	1.77853642922089 (0.00235660700338327)	0	0	0	0
CARTPT	5.63944170840178 (0.000155361093654806)	0	0	0	0	0	0	0	0	0
CASC10	0	0	0	0	0	0	0	0	2.58310469493331 (4.39841633938193e-06)	0
CASC11	0	0	0	0	0	6.12759210531209 (0.00218637561515686)	0	0	0	0
CASC15	0	0	0	0	0	0	0	0	-2.66804976855539 (0.000736071299789588)	0
CASC18	0	0	0	0	0	6.9351085228489 (0.00179051293467684)	0	0	0	0
CASC5	-2.98200814579201 (0.00110293494878362)	0	0	0	0	0	0	4.11950652885361 (1.18570819217579e-05)	0	0
CASK	0	0	0	0	0	0	0	-4.55843260099434 (0.00468808957676198)	0	0
CASKIN1	0	0	5.0932585378084 (0.000126697880370822)	0	7.03264347848523 (0.0003623415780549)	6.44506403171399 (0.00125092256111326)	0	0	0	0
CASKIN2	0	0	0	0	0	0	0	0	-1.78248504651519 (0.00398684465843513)	0
CASP10	-1.62250665635306 (0.00327620145323539)	0	0	0	0	0	0	0	0	0
CASP14	0	0	0	0	7.63604282932785 (1.78266356051587e-07)	0	0	0	0	11.6610610370643 (2.2796412897292e-24)
CASP16	0	0	0	0	0	5.67919576975931 (0.000348255364375039)	0	0	0	0
CASP6	1.98444233896481 (0.00235986406465053)	0	0	0	0	0	0	0	0	0
CASP7	0	0	-1.73196522560155 (0.00544912020634893)	0	0	0	0	0	0	0
CASQ1	0	0	6.98045056581031 (0.000325998854333003)	0	0	0	0	0	0	0
CASQ2	3.41813365454525 (2.60941090213464e-06)	0	6.47973732258907 (4.96064240397877e-21)	3.6603044616549 (0.00343382118835457)	0	0	0	0	0	0
CASR	0	0	0	0	7.5070014335697 (1.12886607701164e-06)	7.63012014102351 (0.000519530046316305)	0	0	0	0
CASS4	-3.36678787301825 (0.000277117332843985)	0	-4.41726028215062 (2.23196379647305e-05)	0	0	0	0	0	0	0
CASZ1	0	0	0	0	0	0	0	0	-1.91693414591957 (0.000177480611870499)	0
CAT	0	0	0	0	0	0	0	2.68035837843853 (0.000370760744719556)	0	0
CATSPER1	0	0	0	0	0	6.12982525419746	0	0	0	0

						(0.0018788647512987)				
CATSPER2	0	0	0	0	0	2.55416862967489 (0.00766470398869618)	0	0	0	0
CATSPERB	0	0	0	0	0	0	0	0	3.53693681359567 (0.00233872979043354)	0
CATSPERG	0	0	0	0	4.03902941905698 (0.00147811727719988)	4.46996552488114 (0.000864499805728921)	0	0	0	0
CAV1	0	0	0	0	0	-2.3046009629825 (2.9879283026348e-05)	0	-5.61117344019679 (0.000397995272102857)	0	0
CAV2	0	0	0	0	-2.2056707377064 (0.00023992412613249)	-2.07789919310437 (8.73566773389033e-05)	0	-8.19557263559136 (0.00012566660813139)	0	0
CAV3	0	0	5.49782483298658 (0.000107202557380193)	0	0	0	0	0	0	0
CBFA2T3	-1.62756405114445 (0.0027138399477391)	0	0	0	0	0	0	0	0	0
CBFB	0	0	0	0	0	-2.01692205365661 (0.00591465043604635)	0	0	0	0
CBLN2	4.64609988101081 (0.00350973280199088)	0	0	0	0	6.8747978295475 (0.00948482808621846)	0	0	0	0
CBWD1	0	0	0	0	0	0	0	2.25577190146958 (0.00572750290187174)	0	0
CBX2	0	0	0	0	0	4.4534160635539 (0.00216367025349838)	0	0	0	0
CBX7	1.83188060130887 (3.42687403733667e-05)	0	1.72868187584076 (0.000453239336560929)	2.70222732587316 (0.00137487991366359)	0	0	0	0	0	0
CBX8	-3.81227904789071 (0.00670797905995725)	0	0	0	0	0	0	0	0	0
CCBE1	0	0	-4.15060884242443 (3.62146769287642e-08)	-2.9374141175989 (0.00464353373152338)	0	0	0	0	-6.10031662056218 (3.7530658087762e-15)	0
CCDC102B	-4.61337888313608 (5.01998818530851e-17)	0	-3.46026967141822 (4.18167362653835e-08)	-2.8712587414354 (0.000424697942574646)	-2.62189635476226 (0.000340810737797125)	-2.41650338732676 (7.18177743440039e-05)	0	-6.06369381996396 (0.000203851453390104)	0	-3.49430967860798 (2.86667088274353e-05)
CCDC113	-3.38334851466971 (0.000475279969159711)	0	0	0	0	0	0	0	0	0
CCDC125	0	0	0	0	0	2.16053129160215 (0.000524466468967424)	0	0	0	0
CCDC129	0	0	0	0	0	4.74646053065831 (0.00430221433418681)	0	0	0	0
CCDC136	3.3208242148704 (1.60209194009605e-08)	0	2.16205580069489 (0.00507790164073663)	5.71181560448915 (4.52490831127575e-10)	0	0	0	0	0	0
CCDC14	0	0	-2.10434252417861 (5.97099522279606e-06)	0	0	0	0	0	0	0
CCDC141	0	0	2.31063047600767 (0.000283615677549202)	0	0	0	0	0	-2.24842647119888 (7.11049238378121e-05)	0
CCDC144CP	0	0	0	0	0	4.36212330736295 (0.000663265284458218)	0	0	4.0769368255099 (5.34931449727414e-09)	0
CCDC144NL-AS1	0	0	0	0	0	0	0	0	4.2496270824688 (2.10834885398584e-06)	0
CCDC150	0	0	0	0	0	3.48789925470033 (0.0079381229334918)	0	0	0	0
CCDC150P1	0	0	0	0	0	0	0	0	2.0844313980413 (0.000969242228945366)	0
CCDC157	0	0	0	0	0	4.93684706002021 (0.00282287101562378)	0	0	0	0
CCDC158	0	0	0	0	0	3.82734505138837 (0.00959843617814646)	0	0	0	0
CCDC162P	0	0	0	0	0	0	0	0	4.41117447090474 (6.35783812326359e-05)	0
CCDC166	0	0	0	0	0	6.469711239845 (0.00392260125449649)	0	0	0	0
CCDC167	0	0	0	0	0	0	1.56475904392223 (0.00837932453208868)	0	0	0
CCDC168	0	0	0	0	4.71421186076072 (0.00123576624214566)	5.66623608813769 (2.07832087744449e-05)	4.46204650235198 (0.00207727178980109)	0	0	0
CCDC169	0	0	0	0	0	4.98496017861094 (0.00104712601933286)	0	0	0	0
CCDC170	-4.62796351519618 (0.000606158993139864)	0	-3.51203874052695 (0.00339655876836602)	0	0	0	0	0	0	0
CCDC171	0	0	0	0	0	0	0	0	1.69018089628775 (0.00756416983516071)	0
CCDC178	0	0	0	0	6.65548282265748 (0.000250159305027331)	6.10213009384402 (0.00755053791667713)	0	0	0	0
CCDC180	0	0	0	0	0	4.37061354837789 (0.000134139431124327)	0	0	0	0
CCDC183-AS1	0	0	0	0	0	3.79435274518241 (0.00810576690126631)	0	0	0	0
CCDC186	0	0	0	0	0	-1.51281753437617 (0.00718098398836213)	0	0	0	0
CCDC34	0	0	0	0	0	0	0	3.81500127297592 (0.000333376596013507)	0	0
CCDC38	0	0	0	0	0	6.37117209463019 (0.00442564728836765)	0	0	0	0
CCDC40	0	0	0	0	0	0	0	0	-1.74347594086767 (0.00422395752326805)	0
CCDC50	0	0	0	0	0	0	0	-2.39251499972334 (0.00217512117951961)	0	0
CCDC64	0	0	0	0	2.86743095353087 (0.000416631742864805)	0	0	0	0	0
CCDC64B	-4.02072833341718 (0.000553164499870989)	0	-2.86895716605834 (0.00560535725875229)	0	0	0	0	0	0	0
CCDC68	0	0	0	0	0	0	0	0	0	-3.32190881560117 (0.00687408692664079)
CCDC69	1.72883044702906 (3.03001073551992e-07)	0	0	1.89498792651725 (0.00391361747938945)	0	0	0	0	0	0
CCDC71L	0	0	-2.5368661997241 (6.83686546115301e-05)	0	0	0	0	0	-2.29751884540042 (0.000531853590674868)	0
CCDC73	0	0	0	0	0	0	5.26850043511447 (0.00784570046835955)	0	0	0
CCDC80	0	0	0	0	0	0	0	-4.37397539336011 (3.25091370087373e-06)	0	0
CCDC84	0	0	-1.5787950343417 (0.0021635382978862)	-2.34713479332862 (0.00843069256474727)	0	0	0	0	0	0
CCDC85A	0	0	0	0	0	0	1.89272251626116 (0.00333039561317262)	0	0	0
CCDC85B	0	0	1.69578326106274 (0.00602798829491662)	0	0	0	0	0	-1.52180462859859 (0.000818038301687929)	0
CCDC88B	-2.65927790580319 (5.91520574135351e-05)	0	-3.29913096566888 (5.23045081928965e-05)	0	0	0	0	0	-3.45636189660225 (7.67130856860031e-08)	0
CCDC88C	-2.38681551251886 (2.90189443215962e-05)	0	0	0	0	0	0	0	-2.73622329556004 (8.99218382806538e-09)	0
CCER1	0	0	0	0	0	6.7718725898029 (0.00239250939652811)	0	0	0	0
CCKAR	0	0	0	0	6.47559966567641 (0.0042482603748159)	0	0	0	0	0
CCL11	3.63157547739299 (0.00375511832818684)	0	5.67309327104521 (1.12808224794482e-08)	0	0	0	0	0	0	0
CCL14	0	0	0	0	0	0	4.24675109307443 (0.00767090414314997)	0	0	0
CCL16	0	0	0	0	0	7.07489970956963 (2.41244106650322e-07)	0	0	0	0
CCL18	0	0	-5.28462770215139 (0.00126120418360129)	0	-6.22641514604911 (0.0050216461115657)	0	0	0	0	-9.35102670525629 (0.00535170160977833)
CCL19	0	0	0	0	0	4.26029867769811 (1.64956349087111e-05)	2.5199134684349 (0.00777162885836817)	0	-3.98553218411996 (0.00311665432061614)	0
CCL2	0	0	0	0	0	0	0	-5.24379217899201 (0.00471167669934049)	0	0
CCL20	-4.40326651808973 (0.000112497434526144)	0	-4.44091126837972 (0.000123298280966512)	-6.45778576717171 (0.00755364528615492)	0	0	0	0	-2.75605713462903 (0.00737846385420053)	-6.73984782971107 (0.00527116127128725)
CCL21	2.05477241976078 (5.93777166115364e-05)	0	0	0	0	0	0	-6.45627547988953 (0.00428019360069957)	0	0
CCL22	0	0	0	0	0	5.19359891629961 (0.00860401631260728)	4.14143687074989 (0.00618477036950451)	0	0	0
CCL28	0	0	0	0	3.08270820864743 (0.00793103313426186)	3.01858315062118 (0.00729315233655088)	0	0	0	0
CCL4	-5.84667541037232 (2.04138577248776e-05)	0	0	0	0	0	0	0	0	0
CCL5	0	0	-4.0240001503978 (0.000116166253724762)	0	0	0	0	0	0	0
CCL8	0	0	0	0	0	0	3.18249617524213 (0.000939041258595378)	0	3.43683383628467 (0.000127741028484373)	0
CCM2L	0	0	2.20327349108814 (0.00113445054646915)	0	0	0	0	0	0	0
CCNA2	0	0	0	0	0	0	0	3.79435043759907 (0.000895432549769615)	0	0
CCNB1	0	0	0	0	0	0	2.13420912798034 (0.000913727050408715)	4.88290401493322 (1.20793260411645e-06)	0	0
CCNB2	0	0	0	0	0	0	0	4.42541665184631 (0.000101887120740002)	0	0
CCNC	0	0	-1.5603732711317 (0.00454475281052744)	0	0	0	0	0	0	0
CCND1	-1.67955891016193 (6.52641961563193e-05)	0	0	0	-1.87826048595502 (0.0010144229061358)	0	0	-4.4976279138376 (1.34450595750808e-06)	0	0

CCND2	0	-5.58204447913648 (0.00826454425000057)	0	0	0	-2.3705363448664 (7.80926699689248e-07)	0	0	0	0
CCNE1	0	0	0	0	0	0	0	0	3.40541091520378 (0.000121075622331171)	0
CCNF	0	0	0	0	0	0	0	3.70918047837578 (0.000294669366806579)	0	0
CCNJL	-4.33951905479313 (0.00145679914770265)	0	0	0	0	0	0	0	-4.19531835505994 (0.000171087492981697)	0
CCNK	-1.54007672017134 (0.00134505432401626)	0	-1.65345822220887 (0.00232537557846123)	0	0	0	0	0	0	0
CCNL1	0	0	-1.62123698225231 (2.33148981151849e-05)	0	0	0	0	0	0	0
CCNL2	0	0	0	-2.11364704973972 (0.00577985224480799)	0	0	0	0	0	0
CCNYL1	0	2.72935388656072 (0.00770179096069666)	0	0	0	0	0	0	0	0
CCP110	0	0	0	0	0	0	0	1.78166455081839 (0.00935245910517312)	0	0
CCPG1	0	0	0	0	-2.70871790609782 (0.00947563160696406)	0	0	0	0	0
CCR1	0	0	-3.05427285532741 (3.5758545261757e-05)	0	0	-2.20278301221989 (0.00638943036142064)	0	0	0	0
CCR5	-3.27257263137001 (0.00282375507278357)	0	-3.05336739453226 (0.00820070526654722)	0	0	0	0	0	0	0
CCRL2	0	0	0	0	0	0	0	0	-3.67096517870824 (3.01868022395013e-06)	0
CCSAP	0	0	0	0	0	0	0	0	3.55954309278019 (4.65453456277324e-11)	0
CCSER1	0	0	0	0	0	0	1.92595933900155 (0.00665063031021538)	0	0	0
CCT3	0	0	0	1.731994642762 (0.00755002645790293)	0	0	0	0	0	0
CCT6A	0	0	0	0	-1.73764341637895 (0.00509876003257248)	0	0	0	0	0
CD109	0	0	-1.61714910597035 (0.00540935515172396)	0	-3.04499315891527 (0.00295414706992671)	-2.69854221464988 (0.00140702310744309)	0	-7.91930541223356 (0.000437849661231562)	0	0
CD151	0	0	1.66137873735655 (7.50593858861077e-08)	0	0	0	0	0	0	0
CD163	0	-4.37736189668627 (0.000258011282293667)	-2.79886227461326 (0.000140159959704124)	0	-2.84104099784813 (0.000926885161666461)	-2.78220665846852 (2.65729416727853e-05)	0	-3.25291519626651 (0.000366641833876482)	-2.077749553802 (1.13327011088664e-05)	0
CD163L1	0	0	0	0	0	0	0	0	2.22690627674997 (0.000851790126166261)	0
CD19	0	0	0	0	0	5.52429764269143 (0.00543262516207356)	0	0	0	0
CD200	0	0	0	0	0	0	0	0	3.69358327119772 (6.15122345867038e-06)	0
CD209	2.53692755862168 (0.000403484409860341)	0	0	0	0	0	0	0	2.19348350465205 (0.000506682692990595)	3.62408383157411 (0.00116438252485897)
CD226	0	0	-2.22962422289053 (0.00878296583055823)	0	0	0	0	0	0	0
CD24	0	0	-2.5503833925535 (0.00512339894332165)	-4.10555718941283 (0.00606289647776905)	3.72548428506809 (0.000346809383413402)	0	0	3.29457265384045 (0.00140532531337602)	1.50262075114318 (0.0093427255860888)	0
CD248	0	0	0	0	0	0	0	-7.56136457391266 (0.000991823288596821)	0	0
CD27-AS1	0	0	0	0	2.27067461655591 (0.0051558062641482)	0	0	0	0	0
CD274	-4.20677794009074 (6.94014977116568e-12)	0	-1.84106149413293 (5.4317392361739e-05)	-5.44022638351592 (0.0007787006762673)	0	-2.97667631306035 (0.000415785864402266)	0	-3.3823325501249 (0.00767395900151478)	0	-2.26158389862288 (0.00776381812989041)
CD276	0	0	0	0	0	0	0	-6.78173830302955 (0.00211553913962498)	0	0
CD28	0	0	0	0	0	0	0	0	3.73924920290006 (1.75151556281553e-07)	0
CD300A	-2.78931395392229 (1.06745985445368e-05)	0	-4.20872410638674 (6.57823934291417e-09)	-3.19780319678149 (0.00215801344006428)	0	0	0	0	-2.86992616520278 (9.0335023993798e-07)	0
CD300E	-4.93193631580284 (0.000284488464338356)	-5.92481615046272 (0.00758195372505168)	-5.07754093712581 (4.25365103338404e-13)	-4.2867096045862 (7.45729765752008e-06)	-4.28441419566124 (2.39913420239857e-05)	-2.64911180750195 (0.00113333402026279)	0	-5.92637186912121 (1.11694294742485e-05)	0	-2.74173448004611 (0.00218532433755122)
CD300LB	-4.12577242769516 (0.00614498203251414)	0	0	0	0	0	0	0	0	0
CD300LF	-3.74597379625072 (0.00731794984602235)	0	0	0	0	0	0	0	-3.6455604214433 (0.00149314061800617)	0
CD300LG	0	0	2.41048387522114 (0.00842729382019156)	0	0	0	0	0	0	0
CD302	0	0	-2.16250871746067 (0.000736804049708504)	0	0	0	0	0	0	0
CD320	0	0	1.94549783465031 (0.00764153993624366)	0	0	0	0	0	0	0
CD34	0	0	1.83005707711517 (0.00547159071051909)	0	0	0	0	0	2.16647800224438 (0.00135933512030528)	0
CD36	0	2.73337129477718 (0.00226311404505135)	2.81369016419529 (8.36779733362064e-08)	0	0	0	0	2.81486496207736 (0.00130156885949162)	0	3.02047493286646 (0.000430152516274016)
CD37	0	0	-1.90923340031256 (0.00119273812363387)	0	0	0	0	0	0	0
CD38	-2.59804662532149 (0.00263082664872395)	0	0	0	0	0	0	0	-2.00150480113143 (0.00692454466053906)	0
CD3D	0	0	0	0	0	0	3.74252998919048 (0.00141674295799289)	0	0	0
CD4	0	0	-3.22698640959055 (2.83720870344257e-09)	0	0	0	0	-2.89789008743893 (0.00487817019272304)	0	0
CD44	0	0	-2.92445418306932 (9.46918693159924e-08)	0	-1.71889806085194 (0.00770343065990051)	-1.95846947324494 (0.000121155560706591)	0	0	0	0
CD46	0	-6.3142460765826 (0.00307495637175137)	0	0	0	0	0	0	0	0
CD47	0	0	-1.71797624899316 (8.62070947150437e-05)	0	0	0	0	0	0	0
CD48	-2.59344661139022 (0.00943052854064494)	0	0	0	0	0	0	0	0	0
CD52	0	0	-3.05491339798874 (0.00389035518555939)	0	0	0	0	0	0	0
CD53	-1.88636142479004 (0.00288571421714509)	0	-2.81808061954826 (0.000279595294070605)	0	0	0	0	0	0	0
CD55	0	0	0	0	-2.17469397227998 (0.000406635473688668)	-1.66540571813951 (0.000879628580685999)	0	0	0	0
CD59	0	0	0	0	0	0	0	-2.00070983231393 (0.00165184117330596)	0	0
CD7	0	0	0	0	0	3.67740981440841 (0.00273002349276384)	0	0	0	0
CD79B	0	0	5.96335438027579 (6.9044481103229e-05)	0	5.7800642702998 (0.00591384444719865)	0	4.38076743507486 (0.000223871081621254)	0	0	0
CD81	0	-5.67978478972158 (0.00758643049931565)	0	0	0	0	0	-2.42559814387103 (0.000433816017400463)	0	0
CD82	0	0	0	-2.76864240898995 (0.000404403101457063)	0	0	0	0	0	0
CD83	-2.91970426433282 (9.43952627525597e-05)	0	0	0	0	0	0	0	0	0
CD84	-1.7081715084072 (0.00784857615761404)	0	-2.99243585202369 (0.000126078301768398)	0	0	0	0	0	0	0
CD86	0	0	-2.56455370241603 (0.00648310180466035)	0	0	0	0	0	0	0
CD9	0	0	0	0	0	-2.18839430362479 (0.00104028188157085)	0	-3.0877530760043 (0.0011962286270385)	0	0
CD93	-1.91726292541121 (2.65769736059809e-05)	0	0	0	0	0	0	-2.90983443243051 (0.000212642740499532)	0	0
CD99L2	0	0	1.87667161291989 (4.93194444788607e-06)	0	0	0	0	0	0	0
CDA	0	0	0	0	0	0	0	5.29085414791476 (0.000117436024948702)	0	0
CDC20	0	0	0	0	0	0	0	5.30971771618062 (0.00317148591043771)	0	0
CDC25B	0	0	-2.67470311417351 (2.42101918817222e-10)	-1.73643710477592 (0.00991238463664)	0	0	0	0	0	0
CDC26	0	0	0	3.14855163815844 (0.00364817798559645)	0	0	0	0	0	0
CDC27	0	0	0	0	-2.02464998999544 (0.00140764548671494)	0	0	0	0	0
CDC42BPG	-4.28634228140446 (6.45863345267962e-07)	0	-4.48142639176824 (2.04820718810565e-06)	-6.9296668293288 (0.0020361412015421)	0	0	0	-5.87162068760631 (0.00929056444507047)	0	0
CDC42EP1	-1.68221272320763 (0.000144103427215299)	0	0	0	0	0	0	-7.46080173996939 (0.000672003192957818)	0	0
CDC42EP2	0	3.04531903665913 (0.00186952600533452)	0	0	0	0	0	0	0	0
CDC42SE2	0	0	-1.72459583350417 (0.00102941782921337)	0	0	0	0	0	0	0
CDCA2	0	0	0	0	0	0	0	4.18964767537535 (0.00049820254415189)	0	0
CDCA3	0	0	0	0	0	0	0	4.01569358985252 (0.000800849592464833)	0	0
CDCA7	0	0	0	0	0	0	0	0	2.00047972941964 (0.00622615883786213)	0
CDCA8	0	0	0	0	0	0	0	4.5583604369197 (0.000462688619764596)	0	0
CDCP1	0	0	-3.74475707280004	0	0	0	0	-6.08303215243868	0	0

			(3.83175720187307e-05)					(0.00705874954111471)		
CDH1	-3.53849310305644 (3.65170114316884e-10)	0	-6.70344096129711 (3.1416533681636e-15)	-4.1953827952764 (5.23192896716008e-05)	0	0	0	0	0	0
CDH11	-1.58152598966415 (0.000153414977424261)	-5.7866836149202 (0.00718999903169846)	-3.41435773957488 (1.7056913258279e-12)	-2.25444574426054 (0.00144457826987289)	-1.89885807863956 (0.000483671061304986)	-2.85859200986644 (1.82117492744996e-06)	0	-9.22618224990501 (1.88948997058824e-05)	0	0
CDH16	0	0	0	0	9.10085441122133 (2.1944597233877e-12)	0	0	0	0	0
CDH17	4.186231563754 (0.00298898522286478)	0	0	0	0	0	0	0	0	0
CDH18	0	0	0	0	0	6.8747978295475 (0.00948482808621846)	0	0	0	0
CDH19	5.71691987090218 (2.28511390459027e-17)	0	5.33349180685942 (3.19619294024333e-11)	4.90303668715853 (3.22629996674431e-05)	0	0	0	0	0	0
CDH2	3.18523864203934 (1.04226129770635e-05)	0	7.91111650278029 (2.08889622822504e-30)	4.16085342528255 (0.00256182498646107)	0	4.66507012810253 (2.09414306068436e-06)	0	0	2.63844078797591 (0.00761168644323125)	0
CDH22	0	0	0	0	6.64816874282679 (0.00347040281013931)	8.04770168613798 (0.000161147798135545)	0	0	0	0
CDH23	-2.28693507741375 (0.00034094763778169)	0	0	0	0	0	0	0	-3.19974150470348 (1.46901132288303e-06)	0
CDH5	-2.47009112544505 (6.96378417249496e-08)	0	0	0	0	0	0	-4.57458137695079 (1.32149783836037e-05)	0	0
CDH6	0	0	-1.63449575113562 (0.00782440237193413)	0	0	0	0	-6.22529341667485 (0.00542736118510463)	0	0
CDH7	0	0	0	0	0	7.49821284982717 (3.28818572099154e-05)	0	0	0	0
CDH8	0	0	0	0	0	6.63672301841438 (0.00079575909094898)	0	0	0	0
CDHR1	0	0	0	0	0	4.48097448302828 (0.00581421447496596)	0	0	0	6.67413977318989 (1.01182033066289e-07)
CDHR2	0	0	0	0	0	7.00816509239574 (1.28269548327898e-07)	0	0	0	0
CDHR3	0	0	-2.88745680904763 (0.00638907506420173)	0	0	0	0	0	-3.50484713027573 (0.000308570737835307)	0
CDHR5	0	0	0	0	3.61834360468289 (2.18250026648513e-05)	4.47691077643506 (2.99582827988734e-07)	0	0	0	0
CDIP1	0	0	1.62955617990629 (0.00215116206783373)	0	0	1.96964887112112 (0.00609265283752655)	0	0	0	0
CDK1	0	0	0	0	0	0	0	3.12854975180275 (0.00557277252121878)	0	0
CDK15	0	0	0	0	0	5.88022810156311 (0.000757830218339401)	4.75905118045848 (0.00536701497546091)	0	0	0
CDK17	0	0	0	0	-2.16149315598272 (0.00227626994922568)	-1.57614461965378 (0.00381898760136129)	0	0	0	0
CDK18	0	0	3.64705081306359 (3.20576173180844e-10)	0	0	0	0	0	0	0
CDK20	0	0	0	0	0	3.56929184203382 (0.00136346696203362)	0	0	0	0
CDK2AP2	0	0	1.85107903036666 (0.00122406820393353)	0	0	0	0	0	0	0
CDK5RAP2	0	0	0	0	0	0	0	1.99467231151484 (0.00336004795984175)	0	0
CDK6	0	0	0	0	0	0	0	0	2.08650257471688 (3.02597511341488e-08)	0
CDK7	0	4.0802760323331 (0.000733421054385079)	0	0	0	0	0	0	0	0
CDK8	1.82816534913384 (6.06689342148957e-06)	0	0	0	0	0	0	0	0	0
CDKL2	0	0	0	0	0	0	0	0	-4.7673094933244 (3.54014295990249e-05)	0
CDKN1A	-2.07999767298562 (4.4866068199495e-06)	0	0	0	0	0	0	-5.53642006044785 (9.13755551689261e-06)	-2.12225323553098 (1.12987954896614e-09)	0
CDKN1B	0	0	0	0	0	0	0	1.97785486860172 (0.00283420894433013)	0	0
CDKN1C	0	0	0	0	2.41476403270286 (0.00391932544198206)	0	0	0	4.57447372129177 (4.94609299518117e-31)	0
CDKN2A-AS1	0	0	0	0	0	7.26986837985775 (0.00668205493536917)	0	0	0	0
CDKN2AIPNL	0	0	1.93132104806287 (0.00647731437083155)	0	0	0	0	0	0	0
CDKN2B	0	0	-2.58974002969529 (0.000286565510072564)	0	0	0	0	0	0	0
CDKN2D	0	0	0	0	0	0	0	2.52288811328594 (0.00953717750399526)	-2.1892919972942 (0.00382820817361952)	0
CDKN3	0	0	0	0	0	0	0	3.72383143494829 (0.00372276060672441)	0	0
CDO1	0	0	0	0	0	3.9127754244136 (7.74505314731997e-05)	0	0	2.71087717033122 (0.00296674938963501)	0
CDON	5.6112432859695 (8.46578206150915e-25)	0	0	5.11463013893071 (4.96866755241285e-07)	0	3.18719924152605 (0.00135182586238384)	2.60561732846457 (0.000669046926780656)	0	2.72382663126351 (0.000350458639114982)	6.00661124074599 (5.30255592111636e-11)
CDR2L	0	0	0	0	0	0	0	-6.38278923645517 (0.00424968293532195)	0	0
CDS1	-3.46156046791607 (0.000263195510208374)	0	-3.42907452250565 (0.000530699124243353)	0	0	0	0	0	0	0
CDT1	0	0	0	0	0	4.36750347783658 (0.00261828325909283)	0	6.09940675699101 (8.57423581204988e-07)	0	0
CDV3	0	-5.75620519540377 (0.00724429132434359)	0	0	0	0	0	0	0	0
CDX2	0	0	0	0	7.33141360835363 (0.000333976211774335)	0	0	0	0	0
CDYL	0	0	0	0	0	0	0	0	2.03495531047424 (2.24704714636742e-12)	0
CDYL2	0	0	0	0	0	0	0	0	2.0082311944096 (0.000970716270710586)	0
CEACAM1	0	0	0	0	0	2.27803633894209 (0.00143342272437243)	0	3.69412263635634 (4.67664608386491e-05)	1.72320286534091 (0.000806051253235846)	0
CEACAM3	0	0	0	0	0	0	0	5.33848428070632 (3.66970328403977e-06)	0	0
CEACAM5	0	0	0	0	0	0	2.34980241571128 (0.00872896916262096)	0	-3.82035743323275 (0.0047472044591247)	0
CEACAM6	-6.87776629128103 (6.09971510381485e-09)	-5.97924103362766 (0.00592436992720921)	-5.56804720436412 (1.33554649654411e-13)	-8.03701740518826 (7.3814022719065e-07)	-5.47267488034619 (2.71333620939668e-08)	-4.96181185646638 (8.92328418750275e-10)	0	0	-6.78534193386641 (1.72695518626304e-23)	-5.14848723091584 (6.6237877622145e-09)
CEACAM8	0	0	0	0	0	0	0	6.09060779202246 (1.13227334260888e-11)	0	0
CEACAMP3	0	0	0	0	0	5.44188658199151 (0.00411635845121231)	0	0	0	0
CEBPA	0	0	0	0	0	3.26571999885389 (0.000129761499009115)	0	0	0	0
CEBPB	0	0	-1.64953059651474 (0.00501791343267869)	0	-1.83984907191849 (0.00619837618657751)	0	0	-2.31084135224881 (0.00574522773131366)	0	0
CEBPE	0	0	0	0	0	0	0	7.219721828534 (5.09604310802016e-05)	0	0
CECR1	0	0	0	0	0	0	0	0	-1.94255257554522 (0.00620764235400334)	0
CECR2	-1.99832281262494 (0.00217728947667706)	0	0	0	0	2.25104777271187 (0.000241180212662349)	0	0	0	0
CECR5	0	0	0	0	0	2.19598722717037 (0.00121162066787628)	0	0	-1.82481544707219 (0.00222502865283543)	0
CECR7	4.54393273853082 (0.00230530525036229)	0	0	0	0	6.17403808203239 (0.00788213607523971)	4.60140741491584 (0.00333481142704326)	0	0	0
CELF2	0	0	0	0	-2.50411709623862 (0.000615190861227492)	-2.33911022374215 (0.000313880825194973)	0	0	0	0
CELF2-AS1	0	0	-2.7838561843281 (0.000716346016497883)	0	-3.83647364312871 (0.00683374852294262)	0	0	0	-1.81014535862802 (0.000536695230876577)	0
CELF3	0	0	0	0	0	5.80500968092082 (0.00375571273448078)	0	0	0	0
CELSR1	-3.04614431124106 (4.76100538931284e-08)	0	0	0	0	0	0	0	0	0
CELSR2	0	0	0	0	3.03888057469911 (1.83315949475046e-07)	2.68395238441323 (0.00944377121756888)	0	0	0	0
CELSR3	0	0	0	0	0	4.75704872523136 (0.000199206362211078)	0	0	0	0
CEMIP	0	0	-5.73002041761805 (9.91381761253042e-15)	-10.2884211976208 (7.41857847662553e-06)	-5.67496909961708 (7.05914013103037e-06)	-2.68860390039314 (0.00142987184213665)	0	-6.79209627595632 (0.000149016421642729)	-4.98154669786245 (6.88275783674843e-14)	0
CENPBD1P1	0	0	0	0	0	0	0	0	-1.625109037309 (0.00287636143170715)	0
CENPF	-2.87013284289741 (2.22324501488722e-06)	0	0	-3.18989137038123 (0.005586199047599)	0	0	0	3.82704433455535 (3.85999844193476e-06)	0	0
CENPK	0	0	0	0	0	0	0	3.57893407207582 (0.000104124808380363)	0	0
CENPU	-2.78673800414601 (0.0094768003256131)	0	0	0	0	0	0	3.63666124158697 (0.00262246716744246)	0	0
CEP112	0	0	0	0	0	0	0	-5.87581207430011 (0.00860928487435547)	0	0
CEP120	0	0	0	0	-2.80858127594471 (0.00304651830591341)	0	0	0	0	0
CEP128	0	0	0	0	0	0	0	2.501959044607 (0.00408639065105472)	0	0
CEP135	-1.72039209019653 (0.000253761644929252)	0	-1.63739018221307 (0.00164063316022334)	0	0	0	0	0	0	0
CEP152	0	0	0	0	0	0	0	3.58683194361769 (1.00898114382574e-06)	0	0

CEP170	0	0	0	0	-2.25740068724713 (3.61617283302159e-05)	0	0	0	0	0
CEP295	0	0	0	0	0	0	0	2.22195004841853 (0.00157408033744008)	0	0
CEP70	0	0	0	0	0	0	0	0	-2.46294980936723 (6.35950255786132e-10)	0
CEP83-AS1	0	8.67909521208046 (1.78462402834257e-07)	0	0	0	0	0	0	0	0
CEP85	0	0	1.91762840208725 (0.000848982885833162)	0	0	0	0	0	0	0
CERK	0	0	2.15824154192553 (9.78255476481414e-07)	0	0	0	0	0	0	0
CERS3	0	0	0	0	0	7.21553662328963 (0.000319841674255 65)	0	0	0	7.52635328144787 (2.83641090771008e-06)
CES1	0	0	0	0	-2.5399835293697 (0.00917942931190581)	3.36171838464997 (2.13366438627177e-05)	0	-3.71238432001124 (0.00588642949298197)	-3.74767990942289 (3.24096688258485e-10)	-2.4663362867322 (0.00988543160073506)
CES2	0	0	2.09254114228888 (8.88835591256863e-06)	0	0	2.66844783176263 (7.92480142547009e-06)	0	0	0	0
CES3	0	0	0	0	4.96158518367274 (0.000796866091629289)	6.10603442916458 (3.1747573384016e-10)	0	0	0	0
CES5A	0	0	0	0	6.47559966567641 (0.0042482603748159)	0	0	0	0	0
CETP	0	0	0	0	0	6.07570097093894 (0.0020417580326662)	0	0	4.50615201043499 (0.00131351136066309)	0
CFAP221	-5.56463637042395 (5.95165456906389e-05)	0	0	0	0	0	0	0	-3.06222530279319 (0.000763122003007542)	0
CFAP43	-3.63915206943725 (0.0011278997457997)	0	-4.48343748214571 (0.000629611530924975)	0	0	0	0	0	-3.21067971409782 (0.000271727169494205)	-6.41393941165793 (0.00996304695425558)
CFAP45	-2.66661099611709 (0.00419722283725558)	0	-2.92800035028612 (0.00527745179167126)	0	0	0	0	0	-2.53676767536672 (0.00145255983434959)	0
CFAP46	-3.49027498411141 (0.00769661180300915)	0	0	0	0	0	0	0	-5.20778602890436 (6.84755683445115e-05)	0
CFAP57	-4.5681531600141 (0.00413595933167659)	0	0	0	0	0	0	0	0	0
CFAP69	0	0	0	0	0	0	0	0	-3.39261725018855 (5.86114149316729e-05)	0
CFAP74	0	0	0	0	0	0	0	0	-3.15576549938164 (0.00519777155184439)	0
CFB	0	0	0	0	0	4.87428075488028 (7.5305842670559e-09)	2.0126631371678 (0.000841018920286233)	0	0	0
CFD	2.20020599387913 (5.14801411194418e-06)	0	0	0	0	0	0	0	0	0
CFH	0	0	0	0	0	0	0	-3.22911863083227 (3.88111629560943e-05)	0	0
CFHR1	0	0	0	0	0	9.2703373022438 (4.61986473818212e-09)	0	0	0	0
CFHR2	0	0	0	0	0	8.1573661351625 (1.07151893172501e-08)	0	0	0	0
CFHR4	0	0	0	0	0	7.91998468973186 (3.28280856803572e-07)	0	0	0	0
CFHR5	0	0	0	0	0	7.4775811809241 (0.000103295594872913)	0	0	0	0
CFI	0	0	0	0	0	2.82568722040746 (0.000625646105700119)	0	0	0	0
CFL2	1.94433474953687 (7.360245136751e-06)	0	1.92177237580381 (0.000112580229737355)	2.16184212564056 (0.00347974986583037)	0	0	0	0	0	0
CFLAR-AS1	-1.99178329662294 (0.000923509610257128)	0	0	0	0	0	0	0	0	0
CFP	-3.09406416275448 (0.00386337805993876)	0	0	0	0	0	0	0	-3.78528416112332 (0.000249042663786056)	0
CFTR	0	0	0	0	0	2.77226550678989 (0.00116691422560267)	0	0	0	0
CGA	0	0	0	0	0	0	0	0	10.98433803685 (6.33556010280657e-09)	0
CGN	-3.50328901044169 (9.71064419582965e-09)	0	-5.77483467824572 (3.54277750649478e-12)	0	0	0	0	-7.32471928007231 (0.000929405782805061)	-3.30115452887776 (2.94066216588486e-09)	0
CGNL1	0	0	0	0	2.26734093835145 (0.000504738454524757)	1.84991091019791 (0.000171451656021542)	0	-4.10117539829115 (0.00116435004453367)	0	0
CGREF1	0	0	0	0	0	5.51198620791553 (2.43090438184276e-06)	3.66727514090967 (0.0083963581883222)	0	0	0
CH17-118O6.2	0	0	4.39658028246791 (0.00542877909777682)	0	0	0	0	0	0	0
CH17-264L24.1	0	0	0	0	0	4.71920449795229 (2.75825392770822e-05)	2.86515174553552 (0.00889961605262428)	0	0	0
CH17-351M24.1	5.1007160635256 (0.000301977835745762)	0	0	0	0	6.44955136903916 (0.000306568870938611)	0	0	0	0
CH17-360D5.2	0	0	0	0	0	0	0	0	3.34014755106408 (0.000757904644014515)	0
CH17-373J23.1	0	0	0	0	0	0	0	0	2.38337974046206 (0.00818125901908861)	0
CH17-472G23.1	0	0	4.09197805630911 (0.00825177392264773)	0	0	0	0	0	0	0
CH17-472G23.4	0	0	2.01761078399617 (0.000980711822499038)	0	0	0	0	0	0	0
CH17-76K2.6	0	0	0	0	0	5.96635319306394 (0.000369489340011038)	3.97237301324281 (0.00931365747791301)	0	0	0
CH25H	0	0	0	0	0	0	0	0	-2.73452407316935 (0.00166039720544932)	0
CH507-513H4.3	0	0	0	0	0	6.4585474004008 (0.00321594440552497)	0	0	0	0
CH507-513H4.5	0	0	0	0	0	4.04283326639029 (1.80018280696229e-06)	0	0	0	0
CH507-513H4.6	0	0	0	0	0	4.78772044458367 (0.000484774304668875)	0	0	0	0
CHAC2	0	0	0	0	0	0	0	0	-3.98533179449657 (0.0009852880667797)	0
CHAF1A	0	0	0	0	0	0	0	3.16606740286411 (0.000432373363264205)	0	0
CHCHD10	0	0	3.80866388643921 (1.13068187429845e-15)	0	0	2.018528906316 (0.00416235134534115)	0	0	0	0
CHCHD5	0	0	0	0	0	3.70456625317111 (0.00797440874127983)	0	0	0	0
CHD3	0	0	0	2.22620788899155 (0.000902171577894768)	0	0	0	0	0	0
CHD4	0	-5.9842426651144 (0.0046816352393485)	0	0	0	0	0	0	0	0
CHD5	2.75995988057635 (0.00476391801057158)	0	0	0	0	0	0	0	0	0
CHDH	0	0	0	0	0	3.73987411773356 (8.9199713267701e-05)	0	0	0	0
CHGA	7.32388684679029 (1.28908121631359e-09)	0	0	7.14120550460416 (0.00060914181167483)	0	0	0	0	0	0
CHGB	5.23949116107016 (8.6786338185509e-06)	0	4.04547993028636 (0.000938110367686265)	5.62902252990689 (0.00494251244767752)	0	6.28852192723841 (0.00067907491101651)	0	0	0	0
CHI3L1	0	0	0	-7.98972061948897 (0.000249441143580958)	0	1.94577181783288 (0.00223143668443905)	0	0	0	0
CHI3L2	-2.12730162699554 (0.00419566626398768)	0	-3.25766948217471 (0.000583726656880961)	-6.99623895565255 (0.00218120657105552)	0	0	0	0	0	0
CHIC1	1.77701024086592 (6.12069325077798e-06)	0	0	0	2.32740124861703 (0.000804841529395608)	0	0	0	0	0
CHIT1	0	0	0	0	0	0	0	6.22085800047784 (6.52128101428244e-08)	0	0
CHL1	2.55816253889259 (9.10395888727164e-07)	0	0	2.60291783551336 (0.0053453301956206)	0	0	0	0	-4.9766957161699 (2.07253093389666e-07)	0
CHML	0	0	0	0	0	0	0	0	1.94988636566793 (4.03487737032183e-09)	0
CHMP1B2P	3.02313512374024 (0.00720112847815911)	0	0	0	0	0	0	0	0	0
CHMP3	0	0	0	0	0	-1.85475407969742 (0.00812715644958542)	0	-2.80580957675215 (0.00755144883663061)	0	0
CHMP4C	0	0	0	0	0	0	1.84367972845006 (0.0070850661110636)	0	2.74309278068544 (0.000298331120437109)	0
CHN2	0	0	0	0	0	2.89122736894681 (0.00184453779501429)	0	0	2.37762250501196 (6.19930038323162e-06)	0
CHODL	0	0	0	0	0	0	0	0	5.66172561890175 (4.72327367132044e-05)	0
CHORDC1	0	0	0	0	-2.18754285045889 (0.00569496727553863)	0	0	0	0	0
CHP2	4.7374103043041 (0.00396284657272186)	0	0	0	0	0	0	0	0	0
CHPF	0	0	2.12706638505324 (3.41900707001857e-05)	0	0	0	0	0	0	0
CHRD	0	0	0	0	3.41008047324006 (0.000838810703078276)	3.42646415423036 (2.57657055074726e-05)	0	0	0	0
CHRDL1	0	0	-3.64565148781518 (2.63108608377726e-07)	0	-1.63403514844105 (0.00813004153653314)	0	0	-3.18522206693635 (0.000508610055024 38)	0	0
CHRDL2	6.75867582694726 (1.57721186613711e-07)	0	5.39398621413346 (1.20982244138005e-06)	0	0	4.28229705196906 (0.000229924573281221)	0	0	0	0
CHRM2	6.72958365707306 (1.19921447053998e-25)	0	3.82118207891276 (5.81900411140044e-06)	5.6403603791557 (1.03798417419064e-07)	0	0	0	0	0	0
CHRM3	6.07921352595007	0	0	4.65830645016757	0	4.52347475932854	3.40066325771465	0	0	0

	(5.78315052118948e-18)			(0.00347968787534119)		(0.00241862081433658)	(0.00352311999170146)			
CHRM3-AS2	5.04842468124608 (8.46920545236612e-06)	0	0	0	0	0	0	0	0	0
CHRNA3	7.46436937037529 (4.04731459557605e-11)	0	0	0	0	0	0	0	0	0
CHRNA4	0	0	0	0	6.81201551833002 (0.00158736055535963)	7.49545375316033 (6.41287690791013e-07)	0	0	0	0
CHRNA5	0	0	0	0	0	6.17213850306494 (9.32467874268007e-05)	0	0	0	0
CHRNA6	0	0	0	0	0	6.54401974831726 (0.00335150846722125)	0	0	0	0
CHRNB2	0	0	0	0	0	7.14261019565052 (0.00718963385642891)	5.68620931956978 (0.000424072387500166)	0	0	0
CHRNB4	0	0	0	0	0	6.83616064796014 (0.00210048989075515)	0	0	0	0
CHST1	0	0	0	0	0	3.98474233902109 (0.00780241025220017)	0	0	0	0
CHST11	-1.9919244174481 (0.00014102606706953)	0	-2.90154027960869 (6.00443927733001e-07)	0	0	0	0	0	0	0
CHST12	0	0	0	0	0	2.27523188480638 (0.00176349641306896)	0	0	0	0
CHST15	0	0	-2.17903934291623 (4.77338942766065e-09)	0	0	0	0	0	-2.21512654531164 (6.19881668970285e-15)	0
CHST4	0	0	0	0	0	4.34262824828762 (0.00326803367566667)	0	0	0	0
CHST7	0	0	3.43078190551857 (4.29869043367302e-05)	0	0	0	0	0	2.31739297451497 (0.0056398762387495)	0
CHST9	0	0	0	0	0	0	0	0	-4.01046489257978 (0.00360498563890592)	0
CHSY1	-1.5073912402835 (8.68319832376452e-05)	0	0	0	0	0	0	0	0	0
CHSY3	0	0	0	0	0	0	0	0	2.05742124552404 (0.00181647859287585)	0
CIB2	0	0	4.50943523335846 (0.000651409728459383)	0	0	0	0	0	0	0
CIDEC	0	0	4.58622379808592 (0.00720636596709807)	0	0	0	0	0	0	7.22306328554423 (0.000349646628979572)
CIITA	-1.54415379647267 (0.00262362754898275)	0	0	0	0	0	0	0	0	0
CILP	4.52359556271672 (1.04650157794504e-09)	0	3.70431901036499 (2.23745534120134e-07)	3.73079820637807 (0.00262879324966679)	0	0	0	0	0	0
CISD3	0	0	0	0	0	0	0	0	-1.97050340014643 (0.00791247005595519)	0
CISH	-2.59361543152855 (6.65715581210668e-05)	0	0	-3.8100336709957 (0.00258664830817094)	0	0	0	0	0	0
CIT	0	0	0	0	0	0	0	4.72124030934895 (2.25246999281306e-08)	0	0
CITED4	0	0	0	0	4.37713873770853 (0.0013067459697701)	4.69643032361815 (0.00279627848427506)	0	0	0	0
CITF22-92A6.2	0	0	0	0	0	7.55954562460132 (0.000597761216750395)	0	0	0	0
CKAP2	0	0	0	0	0	0	0	2.90165822608285 (0.000707740443845308)	0	0
CKAP2L	0	0	0	0	0	0	0	4.40294390500037 (2.36187106008947e-05)	0	0
CKB	3.44053671287929 (4.3971039943909e-08)	0	2.49714856352088 (1.24489378353704e-05)	3.76685293403104 (0.000199524950379566)	0	0	0	0	0	0
CKM	0	0	12.9184340342369 (8.53262350993173e-34)	0	0	0	0	0	0	0
CKMT2	0	0	7.70112925704705 (1.39260800259875e-34)	0	0	0	0	0	0	0
CKS2	0	0	0	0	0	0	1.6956620032116 (0.00209222265592887)	3.66146543265261 (0.00107972155964887)	0	0
CLASP2	0	0	0	0	-1.60466756194654 (0.00770625939867272)	0	0	0	0	0
CLC	0	0	0	0	0	0	0	6.80720155574357 (0.00424504638285965)	0	0
CLCA1	4.80233907215993 (0.00173502423321374)	0	0	0	0	0	0	0	0	0
CLCA2	0	0	0	0	0	0	3.88588383653381 (0.0024719801087294)	0	0	8.38155099389662 (7.09961562837388e-13)
CLCN4	0	0	2.29126580135195 (0.000665516198196441)	0	0	0	0	0	-2.22291905956681 (0.00894042153777274)	0
CLCN5	-1.76753075713291 (0.00365672517652304)	0	-1.65634953719822 (0.00510498711947599)	0	0	0	0	0	0	0
CLCNKA	0	0	0	0	6.74110619902462 (1.87378956568204e-05)	0	0	0	0	0
CLDN1	0	0	-2.97955543879773 (0.00252273502158213)	0	0	3.47576088040855 (1.33688357658929e-05)	0	0	0	3.79472970163978 (4.80762111555241e-06)
CLDN18	-4.22521354962086 (5.00506694007255e-06)	0	-2.96952302360781 (0.00138924229694767)	-7.05980020768141 (0.0025682589335078)	0	0	0	0	-3.23579299696068 (0.000255273551742302)	-7.33749550584355 (0.00171342327184163)
CLDN19	0	0	0	0	0	6.57967538783077 (0.00353011144244189)	0	0	5.83643010407592 (9.7577453405892e-08)	0
CLDN2	0	0	0	0	0	6.71916000540733 (7.96891833786774e-07)	0	0	0	0
CLDN4	-2.33829709489979 (0.00659748292054986)	0	-4.18818159148304 (7.62593738749046e-05)	0	0	0	0	0	0	0
CLDN5	-1.72150163078342 (2.66089116214438e-05)	0	0	-2.39673902357379 (0.00166400088601543)	0	0	0	-5.03768921953094 (0.00154076639928574)	-4.16420702772952 (5.87503040485145e-23)	0
CLDN7	-3.86536599441769 (0.000160920464065516)	0	-4.08720396525071 (0.000195648832404769)	0	0	0	0	0	0	0
CLDN9	0	0	0	0	0	6.04204651602242 (0.000535198174596217)	0	0	0	0
CLEC12A	0	0	0	0	0	0	0	3.63476229525627 (0.00034689933012194)	0	0
CLEC14A	0	0	1.52137323313543 (0.00054135582831013)	0	0	0	0	0	0	0
CLEC18A	0	0	0	0	5.52563000148059 (0.00185864785767518)	0	0	0	0	0
CLEC18C	0	0	0	0	0	6.10213009384402 (0.00755053791667713)	0	0	0	0
CLEC1B	0	0	0	0	0	3.99854020487627 (0.000740422315247566)	3.03864742221819 (0.00217854463319748)	0	0	0
CLEC3B	0	0	2.46506966097298 (1.9542510080204e-05)	0	0	0	2.24925444012456 (4.97999150531917e-06)	0	2.50255431272792 (2.95665628864014e-07)	0
CLEC4A	-3.67037598599855 (0.00974193034154862)	0	0	0	0	0	0	0	-2.99567305302586 (0.00529174067081772)	0
CLEC4E	-3.30382631774647 (0.000686115698931359)	0	-3.66999726838836 (0.000242006748304659)	0	0	0	0	0	0	0
CLEC4G	0	0	0	0	0	3.36827800385952 (0.00666224625054957)	0	0	0	0
CLEC4M	0	0	0	0	0	4.55034985126417 (0.000228753014060302)	0	0	0	0
CLEC7A	-2.1848325448991 (0.0012923603865507)	0	-3.76494233044202 (4.24431969232482e-06)	-2.8539688368263 (0.00733918815361903)	-3.47651761578153 (0.00226935858199588)	0	0	-3.37930670157205 (0.00808420507639459)	0	0
CLECL1	0	0	0	0	0	0	4.72288961586393 (0.0028130965145341)	0	0	0
CLGN	0	0	3.56182869664357 (0.00376335508608148)	0	0	0	0	0	0	0
CLIC2	-1.59991310049338 (0.00221706320461767)	0	0	0	-3.80332370275283 (0.000832217665818903)	-2.32639053855523 (0.00173668458760065)	0	0	-2.50355578970011 (3.95274251369102e-06)	0
CLIC3	0	0	0	0	0	0	0	0	4.36760430547831 (2.0925159956265e-10)	0
CLIC4	0	0	0	0	-1.57698093476261 (0.00525809524111716)	-2.06835941353953 (4.95990075806622e-05)	0	-3.15030251658175 (6.52874462532784e-06)	0	0
CLIC5	-2.56784103450849 (0.000127093270060196)	0	2.75651303895987 (5.32927172642771e-08)	0	0	0	0	-3.72123274602967 (0.0071576672785478)	0	-3.2527558727054 (0.00227102574099429)
CLIC6	0	0	0	-6.33623845328913 (0.00752853993974552)	0	0	0	0	-3.36231855666797 (0.000112858617769841)	0
CLIP3	2.80652784295043 (2.00494898415658e-13)	0	0	2.53140247987339 (0.000536485486487798)	0	0	0	-6.70002972678143 (0.00229401492986766)	0	0
CLK4	0	2.51626120235235 (0.00289519816415773)	0	0	0	0	0	2.27992926322203 (0.000972687686348268)	0	0
CLLU1	0	0	0	0	0	6.469711239845 (0.00392260125449649)	0	0	0	0
CLMP	2.56418587063922 (3.49719796846362e-14)	0	0	1.84049300040889 (0.0065207076988828)	0	-1.98272058482843 (0.00285697185099556)	0	0	0	0
CLN3	0	4.06401616314678 (0.000116648243722462)	0	0	0	0	0	0	0	0
CLN6	0	0	0	0	0	0	0	0	0	-6.00474927197301 (0.00991129226162064)
CLSPN	0	0	0	0	0	0	0	4.31098381000293 (2.05829214920529e-05)	0	0
CLSTN2	2.07867266725905 (0.00593984102640287)	0	2.04308034951791 (0.00264215357805908)	3.13296682055015 (0.00830928688425922)	0	3.59079635671216 (0.000772650151733296)	0	0	0	3.81104243992064 (0.000503456974842612)
CLSTN2-AS1	0	0	0	0	0	6.20727943273514 (0.00531588844419913)	0	0	0	0
CLSTN3	0	0	0	0	0	1.60233475023505 (0.000519560338915446)	0	0	0	0
CLTB	0	0	2.12909128026884 (7.21724207182405e-07)	0	0	0	0	0	0	0

CLTCL1	0	0	0	0	0	0	0	3.6608701585832 (0.000472175761265394)	0	0
CLU	1.83532224844933 (1.28274807432952e-07)	0	0	2.06909373339013 (0.000303501484489657)	1.82159050279933 (2.88851224273889e-05)	2.15286593797668 (5.46100128589064e-05)	0	-2.47046724029294 (0.000178891285319234)	-1.6668938922916 (0.000562373389452655)	0
CLUH	0	0	2.17852654293078 (6.78134828832554e-06)	0	0	2.40716761900814 (0.00116266946043706)	0	0	-1.68516501056855 (0.00180463007632174)	0
CLVS2	3.92420354456554 (0.00779172295336619)	0	0	0	0	6.463777956067 (0.00517393553494358)	0	0	0	0
CMAS	0	0	0	0	0	1.84282972910945 (0.00155365777571539)	0	0	0	0
CMIP	0	2.26870617666272 (0.00154278397458918)	0	0	0	0	0	0	0	0
CMTM4	0	0	-1.61213692440857 (0.00110903640732025)	0	0	0	0	0	0	0
CMTM5	0	0	4.33924265797257 (0.00233099228000432)	0	0	0	0	0	0	0
CMTM7	-1.72837634787359 (0.0016576723833752)	0	0	0	0	0	0	0	0	0
CMTM8	-4.60291576739322 (0.000460648540227623)	0	0	0	0	0	0	0	0	0
CMTR2	0	0	-1.52812358217049 (0.00380366073923874)	0	0	0	0	0	0	0
CMYA5	0	0	7.6013228337137 (3.0177727229725e-74)	0	0	0	0	0	2.11683373368504 (0.000145169813906481)	0
CNDP1	0	0	0	0	6.64816874282679 (0.00347040281013931)	0	0	0	0	0
CNFN	0	0	0	0	0	0	0	0	0	8.22307170900328 (3.9949244967974e-07)
CNGB1	0	0	0	0	0	7.10557813963175 (0.00762538710788847)	0	0	0	0
CNKSR1	0	0	0	0	0	0	0	0	1.92129257777439 (0.00259840232926764)	0
CNKSR2	0	4.91121777049456 (0.000116093053682656)	0	0	0	0	0	0	0	0
CNKSR3	0	0	0	2.40447869187995 (0.000352757520942409)	0	2.11074642872242 (1.49168446512722e-06)	0	0	0	0
CNN1	4.44243421497397 (1.56237309204795e-19)	0	2.09142581676143 (0.00259302227993046)	5.8993129036706 (3.91381681593812e-13)	0	0	0	0	0	0
CNN3	0	0	0	0	0	0	0	-4.11626953273173 (3.62649492102873e-07)	0	0
CNNM1	0	0	0	0	4.14306072656175 (0.0034357534368745)	4.24174045771523 (0.00645727344523864)	0	0	0	0
CNNM3	0	0	0	0	2.01155275830211 (0.00723093134085923)	0	0	0	0	0
CNOT8	0	0	0	0	-2.82255677317582 (0.000566255963511171)	-1.80624373858009 (0.00174620385234405)	0	0	0	0
CNR1	1.91074391104847 (0.00322340698393551)	0	-3.98030782627596 (0.00057275683007116)	0	0	0	0	0	0	0
CNTD2	0	0	0	0	6.24434293510046 (0.0075784156376657)	0	0	0	0	0
CNTN1	3.92525372912441 (1.63594624125821e-08)	0	0	4.15083492435901 (6.97258428373576e-05)	0	0	0	0	0	0
CNTN2	3.44911885504723 (0.00195738559440411)	0	0	0	0	6.01599989929181 (5.48512937117245e-05)	0	0	0	0
CNTN5	0	0	0	0	4.99267819516915 (0.00708666810481715)	0	0	0	4.37610546739778 (0.00154519881457027)	0
CNTNAP1	1.54816424891788 (0.00652001576387198)	0	0	0	0	0	0	0	0	0
CNTNAP2	4.00345681072718 (0.0051177057371087)	0	0	0	0	7.19468421070681 (4.3844937964505e-07)	4.64071701550375 (0.00765583464576902)	0	4.36585917672617 (0.000315953442368603)	0
CNTNAP3	3.43780697913178 (5.68777424177209e-06)	0	0	0	0	0	0	0	0	0
CNTNAP3B	2.2255534254547 (0.00608193368575372)	0	0	0	0	2.86685288310104 (0.00788433730811592)	0	0	0	0
CNTNAP4	4.59931616751723 (0.0070921922239886)	0	0	0	0	0	0	0	0	0
CNTNAP5	4.51399552997393 (0.00988528806828787)	0	0	0	0	0	0	0	0	0
CNTRL	0	0	0	0	0	0	0	1.89667021042067 (0.00824020180722668)	0	0
COA5	0	3.30507798114792 (0.00141227532780524)	0	0	0	0	0	0	0	0
COBL	0	0	1.553687620364 (0.00655358727635029)	0	0	0	0	-6.09614044503674 (0.00723057713495327)	0	0
COBLL1	-1.62010543337568 (2.41785134870247e-05)	0	0	-1.89107055480106 (0.00605769868081711)	0	0	0	-2.46982040675411 (0.00228260657550767)	2.66261198130528 (1.90337056563562e-14)	0
COG1	0	0	0	0	-3.6885704012376 (0.00639046973605542)	0	0	0	0	0
COG6	0	3.14251224417677 (0.000451468686531608)	0	0	0	0	0	0	0	0
COL11A1	0	0	0	0	0	0	0	0	6.04633816545125 (2.20690555031792e-16)	0
COL12A1	2.29185225029345 (3.55432338388133e-07)	0	0	2.33251305608006 (0.00290419770475456)	0	0	0	-4.33278433268524 (0.000208187565826826)	0	0
COL14A1	0	0	0	0	0	0	0	-3.19565846063692 (0.00975627780662616)	1.85554340097535 (0.000681885001425479)	0
COL15A1	0	0	0	0	0	-3.18105305939664 (0.000175510291240069)	0	-7.86766622171181 (0.000301994038256341)	2.08387973002691 (8.52073485259094e-05)	0
COL16A1	0	0	-1.96353108996656 (0.00023527643006652)	0	0	0	0	-5.49036856844784 (0.00109881028250651)	0	0
COL17A1	0	0	0	0	0	0	0	0	7.23485902199654 (1.72403340201656e-09)	0
COL18A1	0	0	0	0	1.87801539550536 (0.000111443780213311)	2.0685315735262 (0.00185673470036715)	0	0	0	0
COL19A1	0	0	0	0	0	0	0	0	3.97199358360357 (0.00104453742033131)	0
COL1A1	-1.54978275922515 (0.00969493056242332)	-2.93053560108794 (0.00599566758469871)	0	-3.61064801629694 (0.000165800012173362)	0	-2.68257466642498 (0.000612138770345094)	0	-5.78977604814458 (3.50775481398845e-08)	0	0
COL1A2	0	-2.79983972556895 (0.00333983904271838)	0	0	0	-2.5560037682284 (0.000448784142825764)	0	-6.04843819438876 (2.45734972928941e-10)	0	0
COL20A1	0	0	0	0	0	6.22879715816375 (0.000516171692571809)	0	0	0	0
COL23A1	0	0	3.74599632616812 (2.89188559651809e-05)	0	0	0	0	0	0	0
COL24A1	2.26694768819389 (0.00416301479878746)	4.62918158064014 (0.00168474013900376)	0	0	0	0	0	0	0	0
COL25A1	0	0	0	0	0	3.47915078076202 (0.00479283785537761)	0	0	0	0
COL26A1	0	0	0	0	5.7800642702998 (0.00591384444719865)	0	0	0	0	0
COL28A1	3.58540946467113 (2.69734520387321e-05)	0	0	0	0	0	0	0	2.34981367377549 (0.00655578973507176)	0
COL3A1	-1.8624466388039 (0.00299759096205676)	-5.43619571527674 (0.00403158329891617)	-2.58555805093623 (0.000211623126216843)	-2.86151107982162 (0.00556652283216448)	-2.71933246712756 (0.000554534754800418)	-3.37502613552704 (0.000215642660587059)	0	-7.30308502713788 (1.09580924007824e-09)	0	-3.4719298721057 (0.000811072841099858)
COL4A1	0	0	0	0	0	0	0	-4.48130409348538 (8.53202638593832e-12)	0	0
COL4A2	0	0	0	0	0	0	0	-3.74333206901319 (2.23510590583237e-08)	0	0
COL4A2-AS2	0	0	0	0	0	5.70045615821583 (0.00605986571233858)	0	0	0	0
COL4A3	-2.93695040360899 (9.31392681278877e-05)	0	0	-6.24694116376579 (0.00610812784934322)	2.50573972935388 (0.00150329563553298)	0	0	0	0	0
COL4A4	-2.89126768522508 (9.68229384576156e-06)	0	0	0	1.7750922474202 (0.00568062703858778)	0	0	0	0	0
COL4A5	1.86365645222862 (0.000857670160280093)	0	0	0	0	0	0	0	1.89526214108023 (0.000415913772762311)	0
COL4A6	2.49471978426681 (0.000209563743445652)	0	0	0	0	0	0	0	2.52435466671851 (0.000724076569478669)	0
COL5A1	-1.64309218328463 (0.00113275272280894)	-6.95641934858165 (0.00164683238579246)	0	0	0	0	0	-6.9393849818592 (1.88366602533954e-07)	0	0
COL5A2	0	-6.13465738776866 (0.00536476832245728)	-2.11921570418292 (0.000523455318170499)	0	-3.07722543179676 (2.75931570615925e-05)	-2.47320077381798 (6.17545002295795e-05)	0	-9.5731868455405 (1.46705480972592e-05)	0	0
COL5A3	-2.16555293982714 (0.000377437228972193)	0	0	0	0	0	0	0	0	0
COL6A1	0	0	0	0	0	0	0	-2.97511270995497 (4.56537549570761e-05)	0	0
COL6A2	0	0	0	0	0	0	0	-2.92635684050636 (2.55638646559727e-05)	0	0
COL6A3	0	0	0	0	-2.69884635491415 (1.74640287219758e-05)	-2.69368709306917 (6.01678348471063e-06)	0	-3.79613421554177 (1.63687092860933e-05)	0	0
COL6A5	-7.63700598745116 (8.35595635528845e-06)	0	-5.8660067148922 (1.09130606572419e-06)	-7.44698626213275 (0.00210578751874442)	0	0	0	-6.391155346653 (0.00864244038535859)	-6.48256895969511 (2.10848998418131e-09)	0
COL6A6	-3.56399914339395 (0.00219400576811041)	0	-2.49512431850187 (0.000423458442039586)	-3.82518872613702 (0.00152676296473176)	0	0	0	-7.21138027316138 (0.00149691701184386)	-2.9661733745351 (0.00872912912991669)	-3.78335031774575 (0.00113965903649776)
COL7A1	0	0	0	0	3.39769580029331 (0.00141151568366886)	0	0	0	0	0
COL8A1	-1.59649581250364 (0.000159976042705522)	0	0	0	-2.23053720328532 (0.00135297682727418)	-1.83406987919998 (0.00253132844810825)	0	-5.87351932435248 (0.000203116991470104)	0	-3.30484863086647 (6.22122330041751e-06)
COL8A2	0	0	0	0	0	0	0	0	3.03533452302634 (2.99388775857279e-06)	0
COL9A3	0	0	0	0	0	6.47049639696431	0	6.61455301937216	0	0

						(0.00415965122570728)		(0.00847819931613035)		
COLCA1	0	0	0	0	0	3.59410476922619 (0.00917168197754329)	0	0	0	0
COLEC10	0	0	0	0	0	6.00902087118539 (1.89019674145095e-07)	0	0	0	0
COLEC11	0	0	3.8917514229929 (0.001135127362626)	0	0	7.04365136636056 (2.99174212175912e-08)	0	0	0	0
COLEC12	0	0	0	0	0	0	0	-7.21957275578545 (0.00108945534167861)	0	0
COLGALT1	0	0	0	0	-1.73200436685022 (0.00901929262570062)	0	0	0	0	0
COMP	-3.35643100301097 (0.00399075607352244)	0	0	0	0	0	0	0	0	3.35639105811994 (0.00171302942250555)
COPG1	0	0	0	0	-1.57504708946152 (0.00160748478487039)	0	0	0	0	0
COPRS	1.51584998635227 (0.00770919566765128)	0	0	0	0	0	0	0	0	0
COPS5	0	2.53645042165663 (0.00238794448935893)	0	0	0	0	0	0	0	0
COQ10A	0	0	3.9742598696324 (1.38109871281596e-05)	0	0	0	0	0	0	0
COQ5	0	3.37139835921917 (0.00953572845579773)	0	0	0	0	0	0	0	0
COQ9	0	0	1.71674496176873 (0.00135916479521904)	2.18738941611116 (0.00717882440150795)	0	0	0	0	0	0
CORIN	0	0	4.29774416886591 (2.83913786081901e-06)	0	0	0	0	0	0	0
CORO1A	0	0	0	0	0	0	0	2.61868535811798 (0.000649179678687774)	-1.9261292556621 (0.000212694717419994)	0
CORO2A	-2.73500786308928 (2.13826264431035e-05)	0	-2.96384958061452 (0.000147955775394528)	0	0	0	0	0	0	0
CORO6	0	0	2.05551621035161 (0.00172735404515101)	0	0	0	0	0	3.24420605756128 (3.50871038411358e-12)	0
CORO7	0	0	0	0	0	0	0	0	-1.60030149417455 (0.00453258028306729)	0
COTL1	0	0	-2.12493329809274 (6.29576055108447e-06)	0	0	0	0	0	0	0
COX14	1.81645590684564 (0.00507310711977905)	0	2.62600203853556 (0.000187506876393535)	0	0	0	0	0	0	0
COX16	0	0	0	0	0	0	1.88824430103246 (0.000879946458131381)	0	0	0
COX4I1	0	0	1.93810631957672 (5.29017743387038e-08)	0	0	0	0	0	0	0
COX5A	0	0	2.3964355345092 (4.52391588403454e-07)	0	0	0	0	0	0	0
COX5B	0	0	2.17484203398296 (7.64029613716041e-08)	0	0	0	0	0	0	0
COX6A2	0	0	10.1163119718453 (1.52870145378128e-22)	0	0	0	0	0	0	0
COX6B1	0	0	1.97701386350228 (2.10012100227119e-07)	0	0	0	0	0	0	0
COX7A1	2.97913543611295 (0.000197908033604596)	0	5.62213240360386 (1.62324152958261e-21)	0	0	0	1.65219423597135 (0.00395517848124783)	0	0	0
COX7B	0	0	2.21880004526379 (4.31843771877164e-06)	0	0	0	0	0	0	0
COX8A	0	0	1.95910320708975 (0.00286957705854032)	0	0	0	0	0	0	0
CP	0	0	0	0	0	5.85288946008711 (1.72386205757711e-11)	0	0	0	2.42002454031945 (0.00729204597359285)
CPA1	0	0	0	0	6.47559966567641 (0.0042482603748159)	0	0	0	0	0
CPA4	0	0	0	0	0	0	0	0	3.93360658156385 (0.00175045207589057)	0
CPAMD8	-3.19662606280361 (4.08778695636643e-06)	0	0	0	0	0	0	0	-1.76219802048293 (0.00444021226188545)	0
CPB2	0	0	0	0	0	6.35399590496766 (7.60718384476049e-11)	0	0	0	0
CPB2-AS1	0	0	0	0	0	4.37405206324126 (0.000909779638194211)	0	0	0	0
CPD	-2.02162607059907 (1.20171774118436e-09)	0	-2.19911855487566 (2.09899258623114e-09)	-1.74884111197672 (0.00736613213141198)	-2.18743169886617 (2.65200740583394e-05)	-1.82418234748011 (0.000244140540597574)	0	-2.21996136251318 (0.00129698461886725)	0	-2.04187226778715 (0.00185821535159677)
CPE	2.1771711709807 (3.08096578892768e-05)	0	1.56860322576075 (0.00377778014911307)	0	2.44558506132361 (2.23123015517734e-05)	0	0	0	0	0
CPEB1-AS1	0	0	0	0	0	5.25841418946758 (0.00281396534478509)	0	0	0	0
CPED1	1.83062349256681 (0.000129252641737562)	0	0	2.25145992412583 (0.00213240291312047)	0	0	0	0	0	0
CPLX2	3.78544645839083 (0.00121161827823259)	0	0	0	0	5.73319558900765 (0.00124043561354746)	0	0	0	0
CPM	-2.70420814164493 (1.02323747725479e-05)	0	-3.08764574864622 (1.5533454154294e-07)	0	0	-2.13218639587263 (0.00597816962367273)	0	-4.45188691993422 (8.52913962514758e-05)	0	0
CPN1	0	0	0	0	0	6.67944909718396 (0.000253210351355361)	0	0	0	0
CPN2	0	0	0	0	0	9.43472975527721 (8.46868398494307e-13)	0	0	0	0
CPNE1	0	0	0	0	-4.18582455845441 (0.000381331804366173)	0	0	0	0	0
CPNE4	0	0	5.13142206048836 (0.000468645762842241)	0	0	6.79692181232265 (0.00011013746103806)	0	0	0	0
CPNE5	0	0	4.73138165183684 (1.35610884920003e-07)	0	0	0	0	0	0	0
CPNE6	0	0	0	0	0	5.78534054309996 (0.00131654948931097)	0	0	0	0
CPOX	0	0	0	0	0	0	0	4.28171321371479 (1.47798011759747e-08)	0	0
CPS1	0	0	0	0	0	7.65358778030715 (4.74544911262607e-22)	0	0	0	0
CPT1B	0	0	2.97279567674864 (0.00601424538586273)	0	0	4.19662930323198 (0.00435853131439823)	0	0	0	0
CPTP	0	0	2.97401439454664 (2.50573806071196e-05)	0	0	0	0	0	0	0
CPVL	0	0	0	0	0	0	0	0	1.58257044772218 (0.00294945319972224)	0
CPXM1	0	0	0	0	0	0	0	0	3.30890142312119 (0.00280913253981953)	0
CPXM2	4.2723028321802 (2.00418163831778e-25)	0	0	4.67301292766251 (2.66131893864829e-08)	0	0	0	0	2.70323026112673 (0.000114970468640266)	0
CPZ	0	0	0	0	0	0	0	0	4.73661325725869 (5.35138849315163e-05)	0
CR1	0	0	-2.13577553317834 (0.00455272811631031)	-4.67806324382892 (0.00824641975871498)	0	0	0	0	0	0
CRABP2	0	0	0	0	0	0	0	0	0	4.6901290363658 (9.50572255522457e-05)
CRACR2A	0	0	0	0	0	2.73265530012804 (0.00683026214976869)	0	0	-2.30004156245807 (0.00632424587313839)	0
CRACR2B	0	0	2.08856450752905 (0.00377237864863647)	0	0	0	0	0	0	0
CRAT	0	3.51260735468252 (0.000530267781372755)	1.87440072173261 (7.99299225003234e-05)	0	0	0	0	0	0	0
CRB1	0	0	0	0	0	6.19087655590515 (0.00808608856376238)	0	0	0	0
CREB3L1	-2.15926727384654 (0.00143475759660809)	0	0	0	0	-2.50326861708194 (0.00927513502137622)	0	-4.62684570481248 (0.00836158693234749)	0	0
CREB3L3	4.96480892615188 (0.00121450139175637)	0	0	0	6.48222785639122 (0.00467673319314815)	0	5.4114532238508 (0.00902540930207162)	0	0	0
CREBL2	0	0	-1.89360560924281 (0.000418014914945507)	0	0	0	0	0	0	0
CREG1	0	0	-2.08649923495088 (0.00073278318709148)	0	0	0	0	0	0	0
CRH	0	0	0	0	0	0	0	0	8.98683812617603 (0.00047778048709775)	0
CRHBP	0	0	0	0	0	6.54160106494503 (1.29067865988256e-06)	0	0	4.05343858378495 (0.00532249481087381)	0
CRIM1	0	0	0	0	0	-1.66466735221129 (0.000768140329336247)	0	-5.50359777186257 (7.97303744347963e-07)	1.83352342606873 (6.24005819810728e-07)	0
CRIP1	0	0	0	0	0	0	1.56747182884988 (0.00020602153029758)	0	0	0
CRIP2	0	0	2.68219625853422 (2.39335977806757e-16)	0	0	0	0	0	0	0
CRIP3	0	0	5.63661894362762 (0.000147160375240457)	0	0	5.28579270488507 (0.00765943827372971)	0	0	0	0
CRISP3	0	0	0	0	0	7.26986837985775 (0.00668205493536917)	4.57444693978363 (0.00226428777016815)	9.01667531988768 (1.02801241726329e-09)	0	0
CRISPLD1	0	0	0	0	0	0	3.13559063008796 (0.00112933625919357)	0	0	0
CRISPLD2	0	0	0	0	0	0	0	-2.53606166694632 (6.18487629552484e-05)	0	0
CRLF1	-2.38092780000197 (0.0042328685193509)	0	0	0	0	0	0	0	0	0
CROT	0	0	0	0	0	0	0	0	1.76303203546163 (6.21944703968721e-05)	0

CRP	0	0	0	0	0	13.7531335235569 (6.35267257340575e-25)	0	0	0	0
CRTAM	0	0	0	0	0	0	5.11905627283275 (0.000427770077642742)	0	0	0
CRX	0	0	0	0	0	7.21532832831108 (0.00155691227333103)	0	0	0	0
CRY2	0	0	1.58869721675996 (0.0014023989220336)	0	0	0	0	0	0	0
CRYAB	0	2.21039660381438 (0.0045097780518095)	5.55452145473279 (2.0907945076081e-22)	2.94702323260526 (1.12109288460122e-05)	2.46168088046004 (2.35458510448115e-05)	0	0	-5.38322219026388 (0.000852996392753391)	0	0
CRYBB2P1	0	0	0	0	0	1.91328029799887 (0.00781404826190107)	0	0	0	0
CRYBG3	0	0	0	0	0	0	0	-2.66139045029135 (0.000884034336495228)	0	0
CRYGN	0	0	0	0	0	0	0	0	4.66355030473153 (0.00468007098700411)	0
CRYL1	0	0	0	0	0	2.23464365431481 (0.00104236093915206)	0	0	0	0
CRYM	0	0	5.32226837162294 (1.76720060544897e-07)	0	0	0	0	0	0	0
CRYZ	0	0	0	0	0	3.07153014854633 (1.56382329073729e-08)	0	0	0	0
CSDC2	0	0	6.07761295693049 (7.64776245271216e-21)	0	0	0	0	0	0	0
CSF1R	0	0	0	0	0	0	0	-4.80451804350515 (0.00479428187110996)	1.97131340870558 (5.64005297612606e-07)	0
CSF2RA	-2.75802857917087 (0.000175040187752302)	0	-2.58090582681165 (0.00264606484195872)	0	0	0	0	0	1.75877451910611 (4.75651999466337e-05)	0
CSF2RB	0	0	0	0	0	0	0	0	2.37802690540009 (1.45962070283434e-08)	0
CSF3R	-4.22186729116862 (7.3806653455711e-05)	0	-3.79726667155053 (1.06025888014142e-07)	-4.06843472761518 (2.93238722797126e-05)	-2.67387921855709 (0.0012656687794254)	0	0	0	0	-3.08281707784454 (0.000929610684638307)
CSGALNACT2	-1.56134389243825 (0.00270116717463215)	0	-2.37175229859233 (6.04848048442487e-07)	0	-3.02381625951338 (0.000122506721152291)	0	0	0	0	0
CSH1	0	0	0	0	7.35579798179143 (0.00270092242411477)	0	0	0	12.1737626527184 (2.14176445953498e-10)	0
CSH2	0	0	0	0	0	7.9224813778533 (0.00210535674458453)	0	0	11.9699409791022 (6.19657765165391e-09)	0
CSHL1	0	0	0	0	0	0	0	0	10.0895617607972 (7.08452779962192e-07)	0
CSK	-1.69742755500734 (3.79852163176235e-05)	0	0	0	0	0	0	0	0	0
CSMD1	0	0	0	0	0	5.20842662802661 (0.00103465110373694)	0	0	3.41733098930987 (0.000816245668951166)	0
CSMD2-AS1	0	0	0	0	0	6.96977162684898 (0.00897266232869622)	0	0	0	0
CSMD3	0	0	0	0	0	4.56856459618567 (0.00686539606877928)	0	0	0	0
CSNK1A1P1	0	0	0	0	0	0	0	0	5.69681571943296 (1.83482651183633e-07)	0
CSPG4	0	0	0	2.31979432227957 (0.0083358057949536)	0	0	0	-7.61765059311116 (0.000693344864701653)	0	0
CSRNP1	-1.53703283866035 (0.00845106371603466)	0	0	0	0	0	0	0	0	0
CSRNP3	3.06899177047078 (3.68127033679767e-05)	0	0	0	0	4.03338427979034 (0.00126771398416914)	3.15126818016846 (0.0035680498024909)	0	0	0
CSRP1	1.99012436818217 (4.36180481150834e-07)	0	0	2.15836550269328 (0.00151375421455487)	0	0	0	-3.65555251122745 (1.79800742595373e-05)	0	0
CSRP2BP	0	0	0	0	0	0	0	0	2.30327379596644 (1.77060156117561e-05)	0
CSRP3	0	0	11.4336607262751 (2.77612745630218e-25)	0	0	0	0	0	0	0
CST3	1.53019312254623 (8.75530284164561e-05)	0	0	0	0	0	0	-3.43978445715083 (0.000334674638372267)	0	0
CST6	0	0	0	0	0	0	0	0	3.82443685765167 (0.00608303648840404)	0
CST7	0	0	0	0	0	0	0	3.79191230429932 (0.00299634628189378)	-3.8688423281821 (0.00035659067314694)	0
CSTA	0	0	0	0	0	0	0	0	-2.80839901772312 (0.00318673564591155)	0
CSTB	0	0	-1.80873744925681 (0.00324903354798525)	0	0	0	0	0	-1.87933253469115 (0.000488565575442979)	0
CTA-125H2.2	0	0	5.68909759493159 (0.000118768156887453)	0	0	0	0	0	0	0
CTA-204B4.2	0	0	0	0	0	4.50722580747514 (0.00164511670707034)	0	0	0	0
CTA-212A2.3	0	0	0	0	0	6.91734660669512 (0.000161812225694681)	0	0	2.7881847125726 (0.00801447558418711)	0
CTA-228A9.4	0	0	0	0	3.71315631967604 (0.000274698323626726)	4.32697573259104 (3.50479089716464e-05)	0	0	0	0
CTA-280A3.2	0	0	0	0	0	7.93180391118459 (0.000176338887697007)	6.21767390003098 (0.00883343969857724)	0	0	0
CTA-292E10.9	-2.0515987592745 (0.000107141435200831)	0	-1.54744001026889 (0.00366800988563946)	-4.29183416685671 (0.000935181874087439)	0	0	0	0	0	0
CTA-29F11.1	0	0	0	0	0	0	0	0	-3.80686781032148 (0.000532131422397248)	0
CTA-315H11.2	0	0	0	0	0	6.26595102663239 (1.23181194326802e-05)	0	0	0	0
CTA-342B11.3	0	0	4.78430624803681 (0.0033951626523294)	0	7.73253260519586 (3.49064814021803e-06)	9.19146563716291 (8.17092101612707e-07)	7.6163487887588 (0.000403541237361765)	0	0	0
CTA-360L10.1	0	0	0	0	0	0	0	4.81582981110266 (0.00870861738868877)	0	0
CTA-414D7.1	0	0	0	0	0	0	1.85527240959641 (0.00788807876110247)	0	0	0
CTA-722E9.1	-5.05661647993095 (0.000517170200486879)	0	0	0	0	0	0	0	0	0
CTA-796E4.5	0	0	7.37428451727574 (1.75967172989903e-12)	0	6.54579188954221 (0.000256005979761898)	9.55927276802719 (0.000107353978765978)	7.56333727737441 (0.000115291030913603)	0	0	0
CTA-929C8.6	0	0	0	0	0	6.75767980180103 (0.00196565619494222)	0	0	0	0
CTA-929C8.9	0	0	0	0	0	8.52332137691116 (4.79881137447427e-09)	6.183435313262 (0.00711057479560508)	0	0	0
CTA-992D9.11	0	0	0	0	6.18525407318681 (0.00135010533343173)	0	0	0	0	0
CTB-113D17.1	0	0	0	0	0	3.79407626858377 (0.00936225262292493)	0	0	0	0
CTB-127C13.1	0	0	0	0	5.26801605159851 (0.00200551660799014)	0	0	0	0	0
CTB-129P6.7	0	0	0	0	0	5.02592281161154 (0.00711260313206708)	0	0	0	0
CTB-131B5.2	-3.03928233468292 (0.00513216297239106)	0	-3.27037446968865 (0.00729096301965724)	0	0	0	0	0	-2.35542650000652 (0.00401353320741281)	0
CTB-133G6.1	3.42847058311208 (0.000354410790949665)	0	0	0	0	0	0	0	0	0
CTB-139P11.2	-3.99394496596291 (0.00366621096696544)	0	0	0	0	0	0	0	0	0
CTB-176F20.3	0	0	2.26532309238499 (0.00215217031353242)	0	0	0	0	0	2.39515001646816 (0.0020872936351279)	0
CTB-193M12.3	0	0	0	0	5.91840120918759 (0.00384303247343804)	0	0	0	0	0
CTB-193M12.4	4.8414595455885 (4.10621983755133e-07)	0	0	0	0	4.58133005202647 (0.00785629075295286)	0	0	0	0
CTB-33O18.3	0	0	0	0	0	6.37117209463019 (0.00442564728836765)	0	0	0	0
CTB-50L17.14	0	0	0	0	0	4.36075793438391 (5.09838445021073e-05)	0	0	0	0
CTB-50L17.16	0	0	0	0	0	6.66630397801698 (0.00075033488650414)	0	0	0	0
CTC-242N15.1	0	0	0	0	0	7.71471884077475 (0.000325711714080041)	0	0	10.9327081852819 (5.20208760476863e-14)	0
CTC-296K1.3	0	0	0	0	0	0	0	0	4.57427773085103 (0.00184190340638146)	0
CTC-338M12.9	0	0	0	0	0	0	0	0	4.02644551543931 (0.00715916281872189)	0
CTC-339O9.1	0	0	0	0	0	0	0	0	4.90705845716286 (7.32192334221781e-05)	0
CTC-339O9.2	0	0	0	0	0	0	0	0	6.83622899266976 (3.83916868156831e-09)	0
CTC-360G5.9	0	0	0	0	0	6.35993786149111 (0.00619281386009252)	0	0	0	0
CTC-459M5.2	0	5.83502835963941 (0.00130842982103256)	0	0	0	0	0	0	0	0
CTC-510F12.2	0	0	0	0	0	0	0	0	-3.47428255706786 (0.00473456440699395)	0
CTC-523E23.5	0	0	0	0	0	4.74756977894605 (0.00972786793056627)	0	0	0	0
CTC-529P8.1	0	0	0	0	0	0	0	0	6.87787771245586 (2.22727237675959e-08)	0
CTC-559E9.1	0	0	0	0	0	4.33468186053662 (0.0053290014458996)	3.2739072559706 (0.00624429807688093)	0	0	0
CTCFL	0	0	0	0	0	8.04095071334086	0	0	0	0

						(1.37893542199119e-06)				
CTD-2001J20.1	0	0	0	0	0	7.06157958536341 (0.00823408752216087)	0	0	0	0
CTD-2006M22.2	0	0	4.32110525637482 (0.00267471592714794)	0	0	0	0	0	0	0
CTD-2015G9.2	0	0	0	0	0	5.969716078721 (0.00100669290432877)	0	0	0	0
CTD-2017F17.2	0	0	0	0	4.57754435748346 (9.58227745571594e-06)	0	2.83869697309295 (0.00283468520401998)	0	0	0
CTD-2033D15.2	0	0	-2.23931301796521 (0.00503089458002165)	-4.00175120512057 (0.00157581445769536)	-3.5802426357703 (0.0033990287275393)	0	0	-6.38402310028043 (0.00346876515307017)	0	-4.28174723661089 (0.000714216147771119)
CTD-2034I21.2	0	0	0	0	0	6.39894237371284 (0.0044052338061436)	0	0	0	0
CTD-2047H16.2	-4.06843511942228 (0.00341262514784975)	0	0	0	0	0	0	0	0	0
CTD-2047H16.4	-2.04533968679765 (0.00150927541249145)	0	0	0	0	0	0	0	0	0
CTD-2083E4.4	0	0	0	0	0	6.20814128832204 (0.00647518950128456)	0	0	0	0
CTD-2095E4.4	0	0	0	0	6.53007539839149 (0.00410261578073612)	0	0	0	0	0
CTD-2114J12.1	0	0	0	0	0	4.03727578688834 (0.00535117766292721)	0	0	0	0
CTD-2118P12.1	0	0	0	0	0	0	0	0	3.9215041762507 (0.00655989336852372)	0
CTD-2201G16.1	0	0	6.47541745586147 (5.86542875247209e-07)	0	0	0	0	0	0	0
CTD-2201I18.1	0	0	0	0	0	0	5.07466963978005 (0.00266954029117462)	0	0	0
CTD-2207A17.1	0	0	0	0	0	7.30820580829314 (0.000786837051542909)	0	0	0	0
CTD-2246P4.1	0	0	0	0	0	6.54422691721563 (0.000729388963300479)	0	0	0	0
CTD-2260A17.2	0	0	0	0	5.6403872762919 (0.00641340890155407)	0	0	0	0	0
CTD-2267D19.1	0	0	0	0	0	3.19981433528751 (0.00786649266683482)	0	0	0	0
CTD-2269E23.3	0	0	0	0	0	0	0	0	4.1314494493984 (0.00823430788937652)	0
CTD-2270P14.5	0	0	0	0	5.54275394732722 (0.00433940043765025)	5.65784927899725 (0.00290931667391512)	0	0	0	0
CTD-2281E23.1	0	0	0	0	0	6.68891162470414 (0.00306283178446008)	0	0	0	0
CTD-2311M21.2	0	0	0	0	0	0	0	0	8.38988983212281 (1.33374002440774e-15)	0
CTD-2311M21.3	0	0	0	0	0	6.36378003766588 (0.000676739958453976)	0	0	6.64362532238044 (2.34616102803346e-07)	0
CTD-2313J23.1	0	0	0	0	0	7.5389106497497 (0.00386277983424322)	0	0	4.75871834929368 (0.0054939596215004)	0
CTD-2314G24.2	0	0	0	0	0	6.68707223129894 (0.000742099447937559)	0	0	0	0
CTD-2328D6.1	0	0	2.58511227777732 (0.00816789807811937)	0	0	5.20889796703375 (5.81499916938368e-09)	0	0	-1.66006743897918 (0.00524152681533522)	0
CTD-2336O2.1	0	0	4.56005047636953 (0.0026051375148397)	0	0	0	0	0	0	0
CTD-2342N23.1	0	0	0	0	0	6.03826101173533 (0.00784359047003729)	0	0	0	0
CTD-2368P22.1	0	0	0	0	2.45376897602082 (0.00159673511515931)	0	0	0	0	0
CTD-2376I20.1	0	0	0	0	0	6.78974093956219 (0.000872703340346255)	0	0	0	0
CTD-2378E12.1	0	0	0	0	0	5.41162249698773 (0.00689439649845858)	0	0	0	0
CTD-2384A14.1	0	0	0	0	0	0	0	0	4.7712570113514 (0.000117055643471755)	0
CTD-2516F10.2	0	0	0	0	0	4.44797925382885 (1.62151121106616e-05)	0	0	0	0
CTD-2521M24.4	0	0	0	0	0	0	4.72319700691053 (0.00226028029265486)	0	0	0
CTD-2521M24.5	0	0	0	0	0	5.1350829791335 (0.00246790197963634)	0	0	0	0
CTD-2521M24.9	0	0	0	0	0	3.96295959562871 (0.009517105018855)	0	0	0	0
CTD-2528L19.6	0	0	0	0	0	0	0	0	3.35927014692234 (0.00837763716205844)	0
CTD-2541J13.1	0	0	0	0	0	4.67779575504791 (0.000137137304307242)	0	0	0	0
CTD-2542L18.1	0	0	0	0	0	0	0	0	1.77904760459415 (0.000231387974102174)	0
CTD-2553C6.1	0	0	3.47195978578245 (0.00225413054904981)	0	0	4.01087961176623 (0.00544627493256113)	0	0	0	0
CTD-2553L13.9	3.06160402223018 (0.00841760301738672)	0	0	0	0	4.8908345014709 (0.00106615650980082)	2.82730141762955 (0.00396173600312696)	0	0	0
CTD-2561J22.5	0	0	0	0	0	0	0	3.47822669255202 (0.000828030953815619)	0	0
CTD-2587M2.1	0	0	0	0	0	4.54116506858739 (5.79531611425953e-05)	0	0	0	0
CTD-2619J13.17	0	0	0	0	0	3.80778290055365 (0.00260307848979399)	0	0	0	0
CTD-2619J13.19	0	0	0	0	0	5.6767573241389 (0.000556777374730619)	0	0	0	0
CTD-2619J13.8	0	0	0	0	4.06605675009559 (0.000504934156959219)	0	0	0	0	0
CTD-2620I22.3	0	0	0	0	0	0	0	0	6.64186967292096 (8.00756402245302e-10)	0
CTD-2626G11.2	0	0	0	0	5.33288946594913 (1.78238033290204e-05)	0	0	0	0	0
CTD-3006G17.2	0	0	0	0	0	0	0	0	4.38032234234092 (0.000623917419377638)	0
CTD-3051D23.1	0	0	0	0	0	7.45693321175246 (0.00354873928772628)	0	0	0	0
CTD-3064M3.7	0	0	2.81546191204062 (0.000609164046977029)	0	0	0	0	0	0	0
CTD-3088G3.8	-3.20948350808125 (0.000528583696489638)	0	0	0	0	0	0	0	-2.2041329348794 (0.00211940336055845)	0
CTD-3092A11.1	0	0	0	0	0	0	0	3.23188133523916 (0.00809287800275716)	0	0
CTD-3099C6.9	0	0	0	0	0	0	0	0	2.22351509150883 (3.81062422670676e-09)	0
CTD-3128G10.7	0	0	0	0	0	6.80220476362649 (8.56606296674622e-11)	0	0	0	0
CTD-3193O13.12	0	0	0	0	0	7.21261123709105 (0.00552449200967179)	0	0	0	0
CTD-3214H19.6	0	8.25013738959257 (5.25926584225771e-06)	0	0	0	0	0	0	0	0
CTD-3222D19.12	0	0	0	0	0	6.00495494762326 (0.00322385120207526)	0	0	0	0
CTD-3247H4.2	0	0	0	0	0	0	0	0	1.70597558792455 (0.00756530336168495)	0
CTD-3252C9.4	-2.28107350558339 (0.000404186156178771)	0	0	0	0	0	0	0	1.98251971084184 (4.56154184985829e-06)	0
CTDSPL	0	0	0	0	0	0	0	-6.32584583378616 (0.00361948420301256)	0	0
CTF1	2.78769766361986 (0.000453792181238563)	0	0	0	0	0	0	0	0	0
CTGF	0	0	0	0	0	0	0	-8.39720394518413 (0.000115900222179695)	0	0
CTH	0	0	0	0	0	5.21525225231161 (1.41954218284506e-07)	0	0	0	0
CTIF	0	0	1.70097910373321 (0.000970205986799962)	0	0	0	0	0	0	0
CTNNA1	0	0	0	0	0	0	0	-1.84566540840439 (0.00182351510765151)	0	0
CTNNA2	5.20400247730056 (0.000564755488152989)	0	0	0	0	0	0	0	0	0
CTNNA3	6.58764599640229 (1.37807879379375e-09)	0	7.40907700501466 (2.23263208465434e-15)	7.14299137317378 (3.74015402991366e-06)	0	5.80144470700252 (0.000535950651275037)	0	0	3.36225161420697 (0.00929156184345186)	0
CTNNB1	0	-5.55921546262717 (0.00903453039999587)	0	0	0	0	0	0	0	0
CTNNBIP1	0	0	1.95133083301172 (0.00146216654077567)	0	0	0	0	0	0	3.05109472000711 (0.000941103923614767)
CTNND1	0	0	0	0	0	0	0	-2.00220957123899 (0.00174587342315985)	0	0
CTNND2	0	0	5.46659823309758 (1.13595759831746e-06)	0	0	0	0	0	0	0
CTSB	0	0	-1.65718786668609 (0.000549878758887509)	0	0	0	0	-2.43406860191806 (0.00842630284705743)	0	0
CTSC	-1.77077865733985 (0.000109034609183239)	0	-2.3633782214414 (8.93283355215708e-05)	-2.19373674576669 (0.00694099734037028)	0	-2.12774906780296 (0.000389490745409566)	0	-2.55082681359335 (0.00367100267512116)	0	0
CTSD	-1.7690632277479 (0.000224847734700358)	0	0	0	0	0	0	0	-2.11384652316137 (1.66927242490215e-06)	0

CTSE	-7.72004742344013 (8.06659699215221e-10)	0	-5.98958058609819 (2.36038983021777e-08)	-7.54489596939046 (0.00112314432364764)	0	-3.04761555128625 (0.00923284533381469)	0	-6.48898576084234 (0.00527864319049637)	-7.7551335636753 (1.28486908276275e-13)	-7.82655395547284 (0.000725718959888226)
CTSF	2.06113301066355 (0.00244065479426847)	0	2.70297326420246 (6.89914683880743e-05)	0	0	0	0	0	0	0
CTSG	0	0	0	0	0	0	0	8.71167432246382 (7.01431624598083e-05)	0	0
CTSH	-1.85438504297888 (9.59168470951953e-06)	0	-1.64236439114099 (0.000486704912136566)	-2.09984649864383 (0.00676201790009657)	-1.67248020123544 (0.00776507716649502)	0	0	0	-1.73966128145797 (1.24100948737093e-05)	0
CTSL	0	0	-2.28833933024397 (1.07753908408292e-05)	0	-2.26558240933414 (0.00554074628582944)	0	0	0	0	0
CTSO	0	0	-1.52342120196873 (0.00869595278871421)	0	0	0	0	0	0	0
CTSS	-2.7266498436236 (0.00532829941310 98)	0	-2.8320935420981 (0.00510124405466499)	0	-2.80471044970445 (0.00764704114512857)	0	0	0	0	0
CTSV	0	0	0	0	0	0	0	0	4.79877741158361 (3.57557722621157e-06)	7.81133053615548 (8.59997093776575e-09)
CTSZ	0	0	0	0	-2.36070583972203 (0.000496992266692328)	0	0	-2.16899505655045 (0.00917074182472794)	0	0
CTTNBP2NL	0	0	0	0	0	0	0	-4.56334090753735 (0.000173603965640588)	0	0
CTU1	0	0	0	0	0	4.27876401395349 (0.000661815240277032)	0	0	0	0
CUBN	0	0	0	0	0	3.84058880528397 (0.000687581040064315)	2.36786985867965 (0.00771183164818883)	0	0	0
CUX2	0	0	0	0	0	7.36154552020064 (3.09388396442678e-06)	0	0	0	0
CX3CL1	-2.16435011336209 (4.70215880177905e-05)	0	0	0	0	0	0	-4.4403808238678 (0.00828424702819284)	-3.41818329444743 (7.39760988755575e-09)	0
CXADR	0	0	0	0	0	0	0	0	-1.89287741577144 (0.00474902755071117)	3.68836474039018 (7.39332046873059e-06)
CXCL1	0	0	-3.37013114732339 (0.00145741218070434)	0	0	0	0	0	0	-5.13640833091316 (0.0084075753855456)
CXCL12	0	0	0	0	0	0	0	0	-3.15511450857696 (1.22044765601734e-09)	0
CXCL14	0	0	0	0	3.07860793491458 (0.00621493106068984)	0	0	0	3.35860623275029 (1.48653449021724e-06)	3.56632058917757 (0.00726535852755643)
CXCL16	-3.27679070901541 (9.47417070734541e-09)	0	-4.12347789895121 (1.29212741995001e-09)	0	-2.29707656168626 (0.00660442992474101)	0	0	0	-1.97223831978233 (1.85320159183402e-05)	0
CXCL17	-5.3619882900846 (6.41067291405661e-07)	0	-4.83729927978829 (1.5997224701869e-06)	-7.13625396584917 (0.00210961870412883)	-4.28190274668136 (0.00617211873268624)	0	0	-6.07869811689972 (0.00913381126555306)	-6.93487103119001 (1.97045535411719e-11)	-4.97214568008516 (0.00599610657248527)
CXCL2	-3.67298325376934 (6.97278069364286e-08)	0	0	-4.06923553722582 (0.000522935597927338)	0	0	0	-7.45622667589487 (0.000928041426153053)	-2.79220577915313 (0.000151355578467734)	0
CXCL3	0	0	-4.72200774502164 (5.15961357234034e-05)	-6.67263280028517 (0.00598614363256744)	0	0	0	0	-4.05836440547537 (2.09607795016035e-05)	0
CXCL5	-3.65594907410108 (0.00687140422811049)	0	-5.12643531586912 (1.26211929894409e-05)	-7.39326799211145 (0.00237095692220868)	-4.53100133735154 (0.00917975937649203)	0	0	-6.33725606086911 (0.00954647710674879)	-4.68770670941441 (0.00261018381691329)	-5.23389733883815 (0.00716736334910502)
CXCL8	0	0	-6.10468917028949 (4.38753573548558e-06)	-9.84293564483276 (0.00249095124781017)	-3.63106844672288 (0.00632233782428415)	0	0	-8.78015910989334 (0.00694101398688936)	-3.77320840496617 (0.000785517185925181)	0
CXCL9	0	-17.7090379621776 (0.00103605878217785)	0	0	0	0	0	-21.0282660863417 (9.80284439710084e-05)	0	0
CXCR1	-3.91457312239981 (0.00257164713293479)	0	0	0	0	0	0	0	-2.50594957339406 (0.00429055219333379)	0
CXCR4	0	0	-3.55568230703096 (2.04690151035189e-05)	0	-2.76090017818346 (0.00872208217517576)	0	0	0	-2.01771416156491 (0.000737661433259038)	0
CXXC4	2.58369243150769 (0.00599385548119751)	0	0	0	0	3.60111484144119 (0.00259119545086238)	0	0	0	0
CXXC5	0	0	0	0	2.29972754618063 (0.00100019338315639)	2.98140210244154 (6.33204345285727e-05)	0	0	0	0
CXorf36	0	0	2.95106077601167 (9.03784229275823e-06)	0	0	0	0	0	2.60021806861263 (6.80462821100044e-05)	0
CYB561	-1.70302876212332 (0.00501776354173286)	0	0	0	0	0	0	0	0	0
CYB5A	0	0	0	0	0	2.41132236955032 (0.000174614181726358)	0	0	0	0
CYB5R1	0	0	2.44195356845034 (1.39265616360153e-06)	0	0	0	0	0	1.9714426607247 (2.72149096419527e-07)	0
CYB5R2	0	0	0	0	2.80330292883197 (0.00666256189234994)	0	0	0	0	0
CYB5RL	0	0	0	0	0	4.02500010458005 (0.00666970635495315)	0	0	0	0
CYBB	0	0	-3.2387217499711 (3.02001624577105e-05)	0	0	-2.90993585757074 (0.00109454103486263)	0	0	0	0
CYC1	0	0	2.80322507225635 (3.39893038814734e-09)	0	0	0	0	0	0	0
CYFIP1	0	0	0	0	-1.53314519845491 (0.00163834931643246)	0	0	0	0	0
CYGB	0	0	0	0	0	0	0	0	-2.43687662426697 (0.000146955624218294)	0
CYP11A1	0	0	0	0	0	0	0	0	9.0332154539 (1.79669641770115e-17)	0
CYP11B1	0	0	0	0	6.47559966567641 (0.0042482603748159)	0	0	0	0	0
CYP19A1	0	0	0	0	0	0	0	0	7.24947458685888 (7.76097682437068e-07)	3.46682133605352 (0.00733033113828739)
CYP1B1	0	0	0	0	0	-3.70029388068252 (4.58624152142153e-06)	0	0	-3.02689664040775 (0.00133499084310176)	0
CYP21A2	0	0	0	0	0	5.13974376280386 (0.000159417214691082)	0	0	0	0
CYP24A1	0	0	0	0	4.45807875494497 (0.000163618241434086)	0	0	0	0	0
CYP26B1	0	0	0	0	5.55259826680753 (2.11687424909428e-12)	0	0	0	0	3.60643502702277 (0.0025744200753134)
CYP27A1	0	0	0	0	0	0	0	-6.19930781860723 (0.00697544483217312)	-3.49638776455381 (6.46869775060967e-08)	0
CYP27C1	3.80669296254036 (0.0040183345090193)	0	0	0	5.66542619910608 (0.00370318669078859)	0	0	0	0	0
CYP2A6	0	0	0	0	0	6.17009259496243 (1.01229679610071e-05)	0	0	0	0
CYP2B6	0	0	0	0	0	9.50232816328957 (1.11085390694634e-12)	0	0	0	0
CYP2B7P	-6.55625840038145 (1.25711516418595e-14)	0	-6.36354725539201 (1.76453545526972e-12)	-6.64534342807347 (8.59966524177096e-05)	-4.44066290442739 (0.000178988140509121)	0	0	-8.03034816746706 (0.000322502288114376)	-7.39379893043688 (6.4768247775277e-18)	-9.36928895832593 (2.58327857710611e-05)
CYP2C18	0	0	0	0	0	7.80084277407914 (3.45160877806298e-11)	0	0	0	0
CYP2C8	0	0	0	0	0	9.45247751764146 (1.22692966985095e-13)	0	0	0	0
CYP2C9	0	0	0	0	0	10.7613449397664 (5.55489218542795e-18)	0	0	0	0
CYP2D6	0	0	0	0	0	5.60323571457948 (9.32396850311275e-10)	0	0	0	0
CYP2D7	0	0	0	0	5.6403872762919 (0.00641340890155407)	6.56217436161025 (0.000231557683919443)	0	0	0	0
CYP2E1	0	0	0	0	0	10.5173385682567 (8.78984554110552e-28)	0	0	0	0
CYP2J2	0	0	7.26944926999474 (5.93066472719111e-12)	0	0	7.18721403474435 (2.32653473131294e-08)	0	0	6.14910166841274 (1.88170899736328e-09)	0
CYP3A4	0	0	0	0	0	8.38355332313473 (5.68945574403385e-07)	0	0	0	0
CYP3A5	0	0	0	0	0	4.62891319944868 (3.90815227089035e-08)	0	0	-1.836112091076 (0.00753588440280921)	0
CYP3A7	0	0	0	0	0	5.39175169261323 (0.000155722851148132)	0	0	0	0
CYP46A1	0	0	0	0	0	3.28592206591959 (0.00718698395291224)	0	0	0	0
CYP4A11	0	0	0	0	0	4.77627209306231 (1.13524948533287e-05)	0	0	0	0
CYP4A22	0	0	0	0	0	5.6730646532033 (1.36750700037323e-08)	0	0	0	0
CYP4B1	-2.27349916097045 (0.000376080269198332)	0	0	0	-3.32861309752103 (0.00224123633514133)	-4.47366301704973 (1.5685026856556e-05)	0	-6.7950161669295 (0.00189659532570797)	0	0
CYP4F11	0	0	0	0	0	6.15221554438384 (3.41284948423986e-08)	0	0	0	0
CYP4F12	0	0	0	0	0	4.67998753160546 (0.000257556575780538)	0	0	0	0
CYP4F2	0	0	0	0	0	7.7463413895073 (2.33261374230226e-07)	0	0	0	0
CYP4F22	0	0	0	0	0	7.56047870546787 (0.000621964074702878)	0	0	0	7.05313623554546 (0.000826100766648662)
CYP4F3	0	0	0	0	0	5.99448520516523 (4.88858431913711e-15)	0	5.6831907419214 (3.544063019276e-08)	0	0
CYP4F8	0	0	0	0	0	7.06157958536341 (0.00823408752216087)	0	0	0	7.67763367213968 (2.31960125059796e-05)
CYP4X1	0	0	0	0	0	0	0	0	-4.14868182450515 (0.0024136071797639)	0
CYP51A1	0	0	0	0	0	0	1.86494656101597 (0.00301920826217562)	0	0	0
CYP7B1	-1.85112756365525 (0.000932280011802211)	0	-2.8637859980902 (0.000290149468067137)	0	0	0	0	-6.13235917415194 (0.00633936103652438)	-3.36645281910538 (3.8285569100953e-07)	0
CYP8B1	0	0	0	0	0	8.19261568913372 (6.04201711952904e-14)	0	0	5.45454126705742 (7.47199590677126e-11)	0
CYR61	-2.88226815352136	0	0	0	-1.73034776551393	0	0	-6.85448261081182	0	0

	(2.96392256303687e-07)				(0.00506763588310426)			(2.23328458578919e-07)		
CYS1	4.52580689657648 (0.00038774858796429)	0	0	5.68795056596227 (0.00714721231451014)	8.26354863377034 (3.8421060004301e-13)	0	0	0	0	0
CYSLTR1	0	0	0	0	0	0	2.94746199681099 (0.000876216979838082)	0	0	0
CYSLTR2	0	0	3.01286668847173 (0.00327795302174924)	0	0	0	0	0	4.38717302758698 (2.96836545949389e-06)	0
CYTH4	0	0	0	-3.45239522648175 (0.00881599204228736)	0	0	0	0	0	0
CYTIP	-2.3786388855586 (0.00127193636051362)	0	-4.42092657359924 (1.77762718981107e-08)	-3.49437971577287 (0.000904641675694266)	-3.15631674675062 (0.00191320613882704)	0	0	0	-2.14198492472081 (0.00118458860948055)	0
CYTL1	0	0	0	0	0	0	0	0	5.14834620369006 (0.00126072596491348)	0
CYYR1	0	0	1.75476466670473 (0.00333058522865199)	0	2.25397588944028 (0.00180700074634025)	0	0	0	0	0
D4S234E	4.80730683257041 (0.00119901177794191)	0	0	0	0	0	0	0	6.23501716415575 (9.98521673330467e-08)	0
DAAM2	1.62008524650408 (3.45349640963711e-06)	0	0	2.06963698718412 (0.00219692430493037)	0	0	0	0	0	0
DAB2	0	0	-1.79641846085367 (0.000329708692100291)	0	0	-2.05813302818513 (0.00109108487394189)	0	-3.19005029768035 (8.95657488424538e-05)	0	0
DACT2	0	0	0	0	0	0	0	0	5.86685341642836 (1.79182434839837e-07)	0
DACT3	2.07866540795777 (0.000153080607641623)	0	0	0	0	0	0	0	0	0
DAGLA	0	0	0	0	0	0	0	0	-3.66707964688853 (0.000599401544065775)	0
DAND5	0	0	4.17020247349951 (0.00823434815348422)	0	0	0	0	0	0	0
DAO	0	0	0	0	0	6.44300749539742 (0.00303884703552569)	0	0	0	0
DAP	0	0	-1.66846953714798 (4.10773583265397e-05)	0	0	0	0	0	0	0
DAPK1	0	0	-1.799092353574 (0.00498776464521158)	0	0	0	0	0	3.02476618218184 (7.80368363950354e-12)	0
DAPK1-IT1	0	0	0	0	0	0	0	0	2.92343540374898 (0.00996342465524526)	0
DAPK2	0	0	0	0	0	0	0	0	-3.97798869220941 (1.36434781707598e-08)	0
DAPP1	-2.13305235409549 (0.00879824118452463)	0	-2.77032533906738 (0.00538039451816755)	0	0	0	0	0	0	0
DARS	0	0	0	0	0	0	0	0	0	1.69661385802148 (0.00480447643877161)
DAZAP2	0	0	-1.5166827677471 (0.00111023782045874)	0	0	0	0	0	0	0
DBH	0	0	0	0	0	6.06876134193105 (5.08273427626955e-05)	0	0	0	0
DBNDD2	0	0	2.75636090471843 (4.04903547703929e-06)	0	0	0	0	0	0	0
DBNL	0	2.17363637074765 (0.00306528737049864)	0	0	0	0	0	0	0	0
DBP	0	0	0	0	3.56713190265809 (0.00592000778370293)	0	0	0	0	0
DBT	0	0	0	0	0	0	0	-6.50816139753058 (0.00320149637573992)	0	0
DCAF11	0	0	0	0	0	1.58442923729876 (0.00245103164727466)	0	2.39904707758349 (0.00124175179456135)	0	0
DCAF4	0	0	0	0	0	0	0	0	1.90864928320374 (0.000618343847780962)	0
DCBLD1	-1.92625720214131 (2.83962811565851e-05)	0	0	-2.49255444275608 (0.00588274475676783)	0	0	0	-4.24283912219158 (0.00900096523705103)	0	0
DCBLD2	0	0	0	0	0	0	0	-5.08118718300315 (0.00216659772412663)	0	0
DCC	0	0	0	0	0	6.48942371179734 (0.00413062351583803)	4.70725122490079 (0.00114562324431499)	0	0	0
DCD	0	0	0	0	0	0	0	0	0	9.2755433685213 (1.8603338168893e-11)
DCDC1	0	0	0	0	0	3.22038287472517 (0.00119348685564947)	2.32130032917497 (0.00995139365106996)	0	0	0
DCDC2	-4.74030558206387 (0.000571480987315069)	0	0	0	2.6027269595185 (0.00155942256453515)	2.67942094430698 (0.00372960484591645)	0	0	0	0
DCHS2	3.93381693713909 (3.62449747036559e-05)	0	0	0	0	5.55521265055074 (0.000591916363651526)	0	0	0	0
DCK	0	0	0	0	0	0	0	2.09238491447625 (0.00951678331528386)	0	0
DCLK1	1.73722113683483 (0.00187982185173208)	0	0	0	0	0	0	0	0	0
DCN	0	-4.43165924154881 (0.00516377937054593)	0	0	0	0	0	-5.56696193582201 (4.10283868671957e-09)	0	0
DCT	0	0	0	0	0	0	0	0	0	6.79922653539252 (3.33438470885593e-05)
DCTN1	0	0	1.64099346962414 (0.000261292550257269)	0	0	0	0	0	0	0
DCTN3	0	0	0	0	0	0	0	2.95346838817708 (0.00223475585642317)	0	0
DCUN1D2	0	0	1.96376154371824 (0.000148625122871686)	0	0	0	0	0	0	0
DCXR	0	0	0	0	0	2.44422620412849 (0.000675327016335781)	0	0	-1.8129876325236 (0.00235718075988095)	0
DDAH1	0	0	0	0	0	0	0	-5.45042794242856 (0.000653970810397664)	0	-2.03334545209425 (0.00506199983237319)
DDB1	0	0	0	0	0	0	0	0	1.82094237828739 (4.07226530373953e-11)	0
DDB2	0	0	-2.10350473321577 (0.000444854582639851)	0	0	0	0	0	0	0
DDI2	0	0	0	0	0	0	0	1.68024680049268 (0.00741836762168454)	0	0
DDIT4	0	0	-1.6395188030313 (0.00548394193007457)	0	0	0	0	0	0	0
DDIT4L	3.72196538970761 (0.00384879263279891)	0	0	0	0	0	0	0	0	0
DDN	0	0	0	0	0	6.56299978493851 (0.00127968775587249)	0	7.21946469051153 (6.06192366319578e-05)	0	0
DDO	0	0	3.15734867724824 (0.00163748671801547)	0	0	0	0	0	0	0
DDR1	0	0	0	0	2.25806894277243 (6.93111727924683e-05)	0	0	-6.43842423820166 (0.00325203272876866)	0	0
DDR2	2.03837429195628 (2.44465725328386e-09)	0	0	0	0	-2.24827274294968 (0.00216306703207127)	0	-4.60689872015797 (2.08460869100312e-05)	0	0
DDTL	0	0	0	0	0	4.95797299466458 (5.02048855837306e-07)	0	0	0	0
DDX10P2	0	0	0	0	0	6.75581055286089 (0.0024413439442343)	0	0	0	0
DDX25	0	0	0	0	0	7.35195875023175 (0.000887111044120985)	0	0	0	0
DDX28	0	0	0	0	0	4.70087992476287 (0.000499024955626675)	0	0	0	0
DDX3X	0	0	0	0	-1.57359793078803 (0.00182832080871178)	0	0	0	0	0
DDX43	0	0	0	0	0	0	0	0	2.70509678340677 (0.00859280475993058)	0
DDX43P1	0	0	0	0	0	0	0	0	5.92750445906849 (3.00499096047903e-08)	0
DDX58	0	0	0	0	0	0	1.54484454742073 (0.00228309521799598)	0	2.57251965497034 (3.10514244399205e-05)	0
DDX60	0	0	0	0	0	0	0	0	1.7666886926171 (3.46618706022564e-05)	0
DDX60L	0	0	0	0	0	0	0	0	1.99849877159745 (3.18479022623314e-05)	-2.30675759504545 (0.000960497774894946)
DECR2	0	0	0	0	0	3.86618403184383 (6.2788310438447e-05)	0	0	0	0
DEF6	0	0	0	0	0	0	0	2.59369186737211 (0.00244423790886482)	0	0
DEFA4	0	0	0	0	0	0	0	8.40729647637597 (9.142183504626e-13)	0	0
DEFA5	8.6776575453038 (4.51680833535991e-14)	0	0	10.867845313084 (3.42188783950101e-19)	0	0	0	0	0	0
DEFA6	7.1941230748484 (1.25618496465396e-09)	0	0	10.295798399801 (2.58806360344028e-16)	0	0	0	0	0	0
DEFB1	0	0	0	0	7.53224017122658 (4.2765675303985e-07)	7.04676266071476 (2.26549958990642e-05)	0	0	0	6.33554699450578 (0.00214320689733768)
DEGS2	0	0	0	0	0	4.87820308089498 (0.00592510708253076)	0	0	0	0
DEK	0	0	0	0	0	-1.79456144536051 (0.00584905356260311)	0	0	0	0
DENND1C	0	0	-2.8599516438756 (0.000364599845016937)	0	0	0	0	0	-1.7545915307088 (0.00151175858251117)	0
DENND2C	-2.77380612378167 (0.0028813945116858)	0	0	0	0	0	0	0	0	0
DENND2D	-1.827861875224 (0.000379014004326322)	0	-2.75073759418678 (4.03477804918449e-05)	-3.89411253052605 (0.0029331320597473)	0	0	0	0	0	0

DENND3	-1.81088480640421 (2.41722440199042e-05)	0	0	0	0	0	0	0	0	0
DENND5B	0	0	2.24616097362902 (4.61208272721067e-08)	0	0	0	0	0	0	0
DEPDC1B	0	0	0	0	0	0	0	0	6.77478027836939 (0.000959887051179029)	0
DEPDC7	5.04400123902348 (2.10016650324269e-05)	0	0	0	0	6.69868944684856 (5.59409368510833e-08)	3.67980145886736 (0.00574079672389861)	0	3.83896376682481 (0.00843101458599131)	0
DEPTOR	2.91286280517384 (1.48240049199658e-06)	0	0	0	0	0	0	0	2.43830415569327 (0.00282238695805528)	0
DERL3	0	0	0	0	0	0	3.33158772963835 (0.000808058616476919)	0	0	0
DES	4.20797002732313 (1.02639620427966e-14)	0	6.25396595573094 (1.48172582366267e-32)	5.39571766979603 (6.10161276524834e-10)	0	-3.07118784532596 (0.00149304493932431)	0	0	0	0
DESI1	0	0	0	0	0	0	0	-3.37332108233943 (0.00927240757343752)	0	0
DET1	0	0	0	0	3.78254685087675 (0.00134845182387815)	0	0	0	0	0
DEXI	0	0	2.38826074876712 (0.00740615452332623)	0	0	0	0	0	0	0
DFNB31	0	0	0	2.75026717515847 (0.00678588588780353)	0	0	0	0	0	0
DGAT2	0	0	0	0	0	3.32709076011469 (0.00114975232933582)	0	0	0	0
DGCR5	0	0	0	0	0	5.13125556059154 (0.00436535224234345)	0	0	0	0
DGKA	0	0	-1.98631020117234 (0.00083733229243287)	-2.44098216747077 (0.00354960713934104)	0	0	0	0	0	0
DGKB	5.39396216806313 (6.91866384826592e-09)	0	0	0	0	0	0	0	0	0
DGKE	0	0	0	0	0	0	0	0	-1.52991780655991 (0.000713546581710679)	0
DGKH	0	0	0	0	0	-2.29996946656803 (1.71045328124645e-05)	0	-2.57551661260676 (0.00184645369020649)	0	0
DGKI	0	0	0	0	0	2.83727612571818 (0.00867304930894186)	0	0	0	0
DGKQ	0	0	0	0	0	0	0	0	-2.28359607981843 (0.00274170693206449)	0
DHCR24	-2.96115716980747 (2.7315930365773e-09)	0	-2.3527689790723 (2.03055198819595e-05)	-3.03143386532822 (9.56386071099403e-05)	-1.80846678700599 (0.00591348465869989)	0	0	-3.47644608780943 (0.000165396638168827)	-2.89792888274336 (5.46747004108832e-08)	0
DHCR7	0	0	0	0	0	0	0	0	-3.17186040988892 (0.000111411387390262)	0
DHODH	0	3.27599376412549 (0.00733071052630955)	0	0	0	3.26816132544446 (7.1961639535236e-06)	0	0	0	0
DHRS12	0	0	0	0	0	2.99995546977957 (0.0081036866470643)	0	0	0	0
DHRS2	0	0	0	0	7.13598023483296 (5.04619800121332e-06)	6.74290008702884 (3.84168808751726e-06)	0	0	5.40900278961726 (1.13905011501147e-06)	0
DHRS3	0	0	0	0	1.97055076978359 (0.00967662935846412)	2.08059881579393 (0.00662916524272842)	0	0	0	0
DHRS7C	0	0	4.91199055205888 (0.00414364499615015)	0	0	0	0	0	0	0
DHTKD1	0	0	0	0	0	2.49973087974016 (2.31642873974898e-06)	0	0	0	0
DIO2	0	0	2.49838872784063 (0.00400540680373638)	0	0	0	0	0	4.66630894054709 (3.96287253766856e-10)	3.57736696420289 (0.0030645340562904)
DIP2C	1.92252161146862 (2.25588548389053e-09)	0	0	2.49073955312959 (0.000285746141936415)	0	0	0	0	0	0
DIRAS1	0	0	7.67472534572636 (4.44487348654624e-20)	0	0	5.04287574629255 (0.00200432581686071)	0	0	0	0
DIRAS2	0	0	0	0	4.97564746498395 (0.00832114323084874)	0	0	0	0	0
DISC1	0	0	0	0	0	0	0	0	2.21795600462433 (4.89618657652654e-05)	0
DISP2	0	0	0	0	4.84792586126141 (0.00196920773101446)	6.66587412820548 (3.08818499499719e-07)	3.82816339682738 (0.00387589708878836)	0	0	0
DIXDC1	2.43509160105595 (5.64816735140289e-15)	0	0	2.13816722803838 (0.00307570285157254)	0	0	0	-5.98738538236165 (0.0065512730984304)	0	0
DKC1	0	2.32037952533544 (0.00848200268135704)	0	0	0	0	0	0	0	0
DKFZP434A062	0	0	0	0	0	7.48886565224318 (0.000101297263810537)	0	0	0	0
DKFZP434H168	4.43349692651011 (0.000688261086203157)	0	0	0	0	0	0	0	0	0
DKFZp434J0226	0	0	0	0	0	6.05015463499433 (0.00300717459722733)	0	0	0	0
DKK1	0	0	0	0	0	0	0	0	5.99178587404085 (1.33432963065846e-05)	0
DKK2	-3.49659643027737 (0.00486723515689795)	0	0	0	0	0	0	0	0	0
DKK3	0	0	0	0	0	-2.41237727269443 (0.00416978189954578)	0	-6.20726430235882 (0.000135061810902948)	-1.66004624236371 (0.00043584048390161)	-2.18830947515602 (0.00436135231624899)
DLAT	0	0	1.51253369089914 (0.00265126933376353)	0	0	0	0	0	0	0
DLC1	0	0	0	0	0	0	0	-3.68688338088153 (3.04147947407987e-06)	0	0
DLEC1	-5.49418200524373 (5.84612616558444e-05)	0	-4.03212993631453 (0.00155020839498595)	0	0	0	0	0	-2.78966078783246 (0.00125696815007502)	0
DLEU1	0	0	-1.68423856146976 (0.00665654566265771)	0	0	0	0	0	0	0
DLG2	0	0	0	0	0	0	0	0	-1.95042063703971 (0.000290202512449714)	0
DLG5	0	0	0	0	0	0	0	0	2.90898020551135 (7.77551257434372e-09)	0
DLGAP1-AS5	0	0	0	0	4.23250708285056 (0.00286800801751978)	0	0	0	0	0
DLGAP2	0	0	0	0	0	4.67934543977697 (0.00491545153400275)	0	0	0	0
DLGAP2-AS1	0	0	0	0	6.24434293510046 (0.0075784156376657)	0	0	0	0	0
DLGAP5	0	0	0	0	0	0	0	4.67683273181809 (9.88358468736462e-06)	0	0
DLK1	0	0	4.65262076708242 (0.00359634279070492)	0	6.69155492453767 (0.000582816664212058)	0	0	0	11.4776152274451 (8.33924957115387e-29)	0
DLST	0	0	0	0	-2.07161886889741 (0.00117942055411792)	0	0	0	0	0
DLX2	0	0	0	0	6.34175424908784 (0.000450997244425966)	6.70984928697538 (5.93918739868533e-06)	0	0	0	0
DLX3	0	0	0	0	0	0	0	0	6.02988925302673 (6.39594773027981e-08)	0
DLX4	0	0	0	0	0	0	0	0	4.93655695311667 (0.000381077349506686)	0
DLX5	0	0	0	0	0	0	0	0	5.55956360619001 (7.30621517881373e-08)	6.33191093972904 (0.000289149340253417)
DLX6	0	0	0	0	0	0	0	0	4.34080411896568 (0.000133248847882283)	0
DLX6-AS1	0	0	0	0	0	6.65032359453391 (0.000392457591696049)	6.38190964876932 (0.00656068084725532)	0	6.43599423853523 (7.61820468021394e-10)	0
DMBT1	0	0	0	-6.44759703520087 (0.000411863565184495)	0	0	0	-7.83277303974362 (0.000814469704732644)	0	-9.16930842923096 (8.40283476693059e-05)
DMD	2.73285913090526 (2.10730569941213e-08)	0	3.02829788699007 (9.96998142247494e-09)	3.32955855168241 (7.91133681144703e-05)	0	0	0	0	0	0
DMGDH	0	0	0	0	0	6.64636088674042 (1.40712477948018e-09)	0	0	0	0
DMKN	-3.42402608638145 (1.91653276124801e-05)	0	-4.39751016833633 (3.17868016392677e-06)	0	0	0	0	0	0	5.3746508547627 (1.91311710810359e-10)
DMP1	0	0	0	0	4.95360518220304 (0.00294025339472209)	6.18378230505633 (0.00193048178578611)	0	0	0	0
DMPK	0	0	3.10225414226035 (7.95574573444386e-06)	0	0	0	0	-6.47718079805402 (0.00601922196396327)	0	0
DMRT2	0	0	0	0	7.16120793480307 (4.38918165112183e-07)	0	0	0	0	0
DMRTA1	0	0	0	0	0	3.88334634441378 (0.0074504251258463)	0	0	0	0
DMTN	0	0	0	0	0	0	0	3.53442574356945 (3.04147590063394e-06)	-2.12267034688664 (1.42260791130058e-05)	0
DMXL2	0	0	-2.41418013561719 (1.83460553619862e-07)	-1.82227769147475 (0.00577010433246514)	0	0	0	0	0	0
DNAAF1	-4.41433229392175 (1.24562124011237e-05)	0	0	0	0	0	0	0	0	-6.91405462794152 (0.00317697298921915)
DNAH1	0	0	0	-4.24506678791089 (0.00355364568671599)	0	0	0	0	0	0
DNAH10	-2.74794622669075 (0.00520118294445425)	0	-3.13560113148215 (0.00407598050075709)	0	0	0	0	0	-6.27175321430377 (2.9227386356667e-07)	0
DNAH11	0	0	-3.45789486423519 (0.00125718142 1513)	0	0	0	0	0	0	0
DNAH12	-6.43061341975233 (2.14074506670309e-06)	0	-4.63004694398475 (0.000167373267733117)	0	0	0	0	0	-5.61504897974181 (3.77477175117277e-07)	0
DNAH14	0	0	-2.20326406607649 (0.00925353409526607)	0	0	0	0	0	0	0
DNAH2	-5.07120648585261	0	0	0	0	0	0	0	0	0

	(0.0003979504796003)									
DNAH3	-5.18729213463549 (0.000611218028075473)	0	0	0	0	0	0	0	-3.50040150607774 (0.00415061404227965)	0
DNAH5	-5.0872374764511 (1.72833211768931e-08)	0	-4.02679199235013 (0.000225612737325759)	-5.40392175260372 (0.00264499457871411)	0	0	0	-6.78274387564131 (0.00343625825601768)	-6.77455740415395 (3.53826386624192e-11)	0
DNAH6	-3.3755505225195 (0.00284814206996433)	0	0	0	0	0	0	0	0	0
DNAH7	0	0	-3.70043077792581 (0.00281067798663783)	0	0	3.25715073036154 (0.00786913096084228)	0	0	0	0
DNAH9	-6.0191318515409 (2.40003438915214e-05)	0	-4.9335144368357 (0.000222828422614716)	-6.5733688255756 (0.00930931421403631)	0	0	0	0	-4.20998571598935 (3.61796461385359e-05)	0
DNAJA1	0	0	0	0	0	0	1.61689896797755 (0.00735385821462938)	0	0	0
DNAJA4	0	0	2.42944577362535 (0.00019599410802656)	0	0	0	0	0	-1.83899499743585 (0.00207192802188433)	0
DNAJB1	0	0	0	0	0	0	0	0	-1.50132179250927 (0.000739502472004171)	0
DNAJB13	0	0	0	0	0	4.18285277762029 (0.00876407517872481)	0	0	0	0
DNAJB14	0	0	-1.64228043060943 (0.000109324304833767)	0	0	0	0	0	0	0
DNAJB2	0	0	1.5614556822829 (0.0013509081598886)	0	0	0	0	0	0	0
DNAJB5	1.88635820766366 (1.21693997399066e-06)	0	2.45027151984163 (3.52503602350695e-05)	3.13714104348776 (2.36183581205204e-05)	0	0	0	0	0	0
DNAJB8	0	0	0	0	0	6.96331268626672 (0.00168454536380207)	0	0	0	0
DNAJC1	0	0	-1.88239598305063 (0.000354003880429613)	0	0	0	0	0	0	0
DNAJC10	0	0	0	0	0	0	0	-1.89739824068443 (0.00786087403994868)	0	0
DNAJC12	4.86495017707588 (0.000531294132588537)	0	5.15003347036927 (0.00706253764249636)	0	0	0	3.48858168181365 (0.0035858703034624)	0	0	0
DNAJC22	0	0	0	0	0	4.39880314492843 (4.61871187244714e-09)	0	0	0	0
DNAJC27-AS1	0	0	0	0	0	4.77700090555691 (0.00833956504093743)	0	0	0	0
DNAJC6	0	0	0	0	0	0	0	0	1.76281307328486 (7.23547232998093e-05)	0
DNASE1L1	-1.5188498255774 (0.00811464775578764)	0	0	0	0	0	0	0	0	0
DNASE1L3	0	0	0	0	4.65912812335062 (0.000157057846134961)	5.13178610047583 (1.3209366928997e-07)	0	0	0	0
DNER	4.17357393588838 (0.000111360396530375)	0	0	0	0	0	0	0	0	0
DNM3	0	0	0	0	0	0	0	0	-1.98744775914571 (0.000440849370435081)	0
DNM3OS	2.15079145665815 (3.9644573206494e-06)	0	0	0	0	0	0	0	1.53393097142779 (0.000320418581759917)	0
DNMT1	0	0	0	0	0	0	0	2.19210971315827 (0.00319124793838581)	0	0
DOC2B	0	0	1.96425030162156 (0.0040125479109934)	0	0	0	0	0	0	0
DOCK1	0	0	0	0	0	0	0	-2.39468188355303 (0.00468148236877238)	0	0
DOCK10	-1.57249061644825 (0.00471443676438217)	0	-2.30059151792409 (8.77767365651631e-05)	0	0	0	0	0	0	0
DOCK11	0	0	-2.26712214434596 (0.000212185184222583)	0	0	-2.64304139582156 (6.05383120578244e-05)	0	0	0	0
DOCK2	-1.6204921531042 (0.000991490670354047)	0	-2.77363446646375 (1.30066586533256e-06)	0	0	0	0	0	0	0
DOCK3	2.38897477623835 (0.000730373282434556)	0	2.68729831028804 (0.00102431543495871)	0	0	0	0	0	0	0
DOCK4	-1.75139186499209 (3.91910705424359e-07)	0	0	0	-1.95448955552342 (0.000330528582353551)	0	0	-3.6833075538287 (2.60374999506952e-05)	0	0
DOCK4-AS1	-2.71748602133578 (0.00081996367825652)	0	0	0	0	0	0	0	0	0
DOCK5	-1.53482111724405 (6.88403496635354e-06)	0	0	0	0	0	0	0	0	0
DOCK6	-1.68151300006977 (0.00376078424505745)	0	0	0	0	0	0	-3.84334572251052 (0.00148544913157775)	0	0
DOCK8	-1.64001326191087 (3.44614325177984e-05)	0	-2.14460121440913 (1.54622276980037e-05)	0	0	0	0	0	0	0
DOCK9	0	0	0	0	0	-2.36026433059891 (8.18469236638855e-07)	0	-4.3694680457991 (7.52930828861108e-07)	0	0
DOK2	-2.70141695009176 (0.00253928603370055)	0	0	0	0	0	0	0	-2.24451072889247 (0.000626634426962255)	0
DOK3	-2.5317116363633 (0.000312659515189935)	0	-3.56827336427473 (1.56955166580172e-05)	0	0	0	0	0	-2.21242299023421 (3.71534199930295e-05)	0
DOK4	0	0	0	0	2.21657786792639 (0.00196395470367044)	0	0	0	0	0
DOK7	0	0	5.4210162756741 (2.54030118320074e-06)	0	0	0	0	0	0	0
DPCR1	-5.16392327575989 (1.18610540240252e-06)	0	-3.65708151180394 (5.01684641247607e-05)	-6.41173152010827 (0.00400158900553416)	0	0	0	0	-5.04923243471949 (5.87823797769893e-10)	-6.69235363819591 (0.00264560721286791)
DPF3	0	0	4.142719529947 (2.89824586607696e-10)	0	0	4.28679298810528 (2.87398964411135e-05)	0	0	0	0
DPH1	0	0	0	0	2.6059905085953 (0.000565933920886337)	2.14376635403327 (0.000550394190125891)	0	0	0	0
DPM1	0	3.60285835270678 (0.000314492718855188)	0	0	0	0	0	0	0	0
DPP10	5.78372517898949 (2.4863056085035e-05)	0	0	0	0	6.11373970644227 (0.00112125932725744)	0	0	0	0
DPP4	0	0	0	-3.58610852512762 (0.00858443446445853)	0	0	0	0	2.7598333655577 (1.51735347419531e-06)	0
DPP6	5.55663441884665 (2.16433187741212e-13)	0	0	5.57150737278721 (1.23463890369135e-05)	0	4.79752874730579 (0.000217071866726586)	0	0	0	0
DPT	3.42585726533578 (1.80066860805031e-06)	0	0	4.24782272954463 (3.45647680953263e-06)	0	0	0	0	0	0
DPY19L1	0	0	0	0	-2.69654951402708 (0.00200592970795073)	0	0	0	0	0
DPY19L2	0	0	2.30747334314075 (0.0016619684994041)	0	0	0	0	0	1.96461402602971 (0.00167341463215145)	0
DPY19L2P1	0	0	4.89904688121877 (1.90496537707104e-05)	0	0	0	0	0	0	0
DPYD	0	0	-1.93195644104674 (0.000692139764188171)	0	-1.63194427665581 (0.00171723703559681)	0	0	0	-1.98063524971013 (8.74053442768739e-09)	0
DPYS	0	0	0	0	0	5.57491365452811 (1.32509469582852e-09)	0	0	0	0
DPYSL2	0	0	0	0	0	0	0	-2.88152478956536 (1.31489512208053e-05)	0	0
DPYSL3	2.68183899724048 (3.8696112356043e-18)	0	0	2.26488499725937 (0.000104601396529811)	0	0	0	-7.70085888085801 (0.000314492232336027)	0	0
DPYSL4	0	0	3.08820204590236 (0.00130598657820598)	0	0	0	0	0	-3.90309833480036 (0.00942685923802832)	0
DPYSL5	0	0	0	0	0	7.36072096533358 (0.000228021287266735)	0	0	0	0
DRAM1	-1.58525856571943 (0.00523850484132624)	0	-2.51211197782483 (7.94877670242882e-05)	-3.76882880853338 (0.000403452984417685)	0	0	0	0	0	0
DRC1	-5.05101834299205 (0.000470073996451067)	0	-3.95459973047757 (0.00537909499591376)	0	0	0	0	0	-4.94239106580963 (9.0365673216387e-05)	0
DRGX	0	0	0	0	0	6.61176688061951 (7.9484687668161e-05)	0	0	0	0
DRP2	5.76311099441066 (2.55309462016918e-06)	0	0	0	0	6.92906270832939 (0.00244409444608752)	0	0	0	0
DSC2	0	0	0	0	0	0	0	-6.01035337155048 (0.00752494332033476)	2.67795535372531 (7.53694873001161e-11)	0
DSC3	3.92867111014006 (0.000247917397083264)	0	0	0	0	5.57906726535008 (0.00135347179030184)	3.67530508119777 (0.003011494130343)	0	8.176913811304 (4.55104618628745e-22)	10.5656415276457 (3.42303128653587e-27)
DSCAM	0	0	0	0	0	6.9352116117859 (5.95519301335706e-05)	0	0	0	0
DSCAM-AS1	0	0	0	0	0	6.07209398694257 (0.00761536373541361)	0	0	0	0
DSCAML1	0	0	0	0	5.33003161936686 (1.71778434821951e-05)	4.44914302064161 (0.000535347479225129)	0	0	0	0
DSCR4	0	0	0	0	0	0	0	0	4.8977788891313 (0.000455645369142277)	0
DSE	0	0	-2.20986576559735 (6.13995561575064e-09)	0	0	0	0	-4.05557173774651 (9.45214956313043e-05)	0	0
DSEL	0	0	-1.85159413267477 (0.000261790460284192)	0	0	0	0	-6.04333616860179 (0.00586194418998469)	0	0
DSG1	0	0	0	0	0	0	0	0	5.76666756864785 (3.92865135991655e-05)	11.913204954196 (1.94941006040821e-24)
DSG2	-2.59237508404841 (4.89071884799221e-05)	0	0	0	0	0	0	0	1.74587380392747 (0.000683349209973901)	0
DSG3	0	0	0	0	0	0	5.32529101290654 (0.0077208102837953)	0	5.48040805631027 (0.000338406302656881)	10.1824354640711 (8.63223066005391e-16)
DSP	0	0	3.68842624241631 (3.95430501980701e-16)	0	0	2.59194418591502 (1.31942415187584e-05)	0	-3.185847761466 (0.00413892476758563)	4.14500613500417 (1.04582901278002e-29)	5.98977357442712 (6.63391288112723e-15)
DST	0	0	0	0	-1.50483500196415 (0.0090199629593664)	0	0	-3.0434745149646 (1.46253561394896e-05)	0	0

DSTN	1.53034785447228 (6.60169831352753e-05)	0	0	1.9616309307866 (0.00169483152802566)	0	-2.03969857745114 (0.000108171750258377)	0	-2.44781269309122 (0.000404502426710874)	0	0
DSTNP1	0	0	0	0	4.81806693236607 (5.29874602867279e-05)	0	0	0	0	0
DTD2	0	0	-3.77605145199299 (1.55596053655374e-05)	0	0	0	0	0	0	0
DTHD1	0	0	0	0	0	0	0	0	-2.70905447834061 (0.00834927764944785)	0
DTL	0	0	0	0	0	0	0	4.19908551010967 (0.000795616834178638)	0	0
DTNA	1.90982219299707 (3.06361878409215e-05)	0	1.81849473944415 (0.00100269786000897)	0	0	0	0	0	0	0
DTX1	0	0	3.44752992669205 (0.00170953666868605)	0	4.70148833777145 (0.00105113596424919)	5.87103959807409 (0.000129462953961407)	0	0	0	0
DTX4	-2.42066900400586 (2.81562376788132e-05)	0	-1.8100320968796 (0.00223429341128894)	-3.59845116720004 (0.0027246137798819)	0	0	0	0	0	0
DUOX1	-4.5476151697163 (2.7865825335882e-13)	0	-4.46952410463452 (1.01010087866584e-13)	-5.66177721942536 (6.53859039616839e-06)	-3.3914544812241 (0.000112148430416424)	0	0	-3.60352473995429 (0.00052899846033032)	-3.97857472651891 (1.95403245243609e-14)	0
DUOX2	0	0	0	0	0	2.84793316298776 (0.00839354941078329)	0	0	0	0
DUOXA1	-5.13450198173214 (1.00536939336242e-07)	0	-5.11917499474582 (4.29172555695596e-08)	-7.3869413466228 (0.00102232346601963)	0	0	0	-6.32769359234348 (0.00510462252704875)	-4.89190457464093 (2.27205109653958e-12)	0
DUSP1	-2.64883883427903 (8.94357553042393e-11)	-6.22013921417643 (0.00368753972591341)	-1.74296837773524 (2.87487418983737e-07)	0	0	0	0	0	0	0
DUSP13	0	0	4.46695374486098 (0.000432207954890665)	0	0	0	0	0	0	0
DUSP14	0	0	0	0	0	0	0	0	1.60859855581993 (0.0022610600035163)	0
DUSP2	0	0	0	0	0	0	0	0	-4.18648126031369 (0.00181656539125747)	0
DUSP23	0	0	0	0	0	0	0	0	-2.56328397493596 (0.00167665431069074)	0
DUSP26	3.42010775617682 (0.00649894189705094)	0	6.54929141825654 (1.93068886459525e-09)	0	0	0	0	0	0	0
DUSP27	0	0	10.1810041652903 (9.41156733472667e-26)	0	0	6.33625601895534 (0.000531188616837081)	0	0	3.91286201346219 (0.000315529768721476)	0
DUSP28	0	0	3.02785441260359 (0.00221531333270026)	0	0	0	0	0	0	0
DUSP3	0	0	0	0	0	0	0	-2.70218678148227 (0.000698991197512626)	0	0
DUSP4	-2.75077444967267 (0.000638935508279349)	0	0	0	0	0	0	0	0	0
DUSP5	-3.05248202066428 (8.00066601216491e-05)	0	0	0	0	0	0	0	0	0
DUSP7	-1.94725153543286 (3.01073487205953e-07)	0	0	0	0	0	0	-3.7654382195735 (0.00223250492902944)	0	0
DUSP8	-1.64936299899976 (0.0092803076344697)	0	1.57167960642753 (0.0044058541163544)	0	0	0	0	0	-1.89832754114668 (0.000847065339187067)	0
DUSP9	0	0	0	0	7.72018138541824 (6.7726726057314e-06)	0	0	0	8.53867262182969 (1.63249999077179e-06)	0
DUX4L17	0	0	0	0	6.47559966567641 (0.0042482603748159)	0	0	0	0	0
DUX4L18	0	0	0	0	0	6.83616064796014 (0.00210048989075515)	0	0	0	0
DUX4L19	0	0	0	0	7.19634778037627 (0.000464954399480524)	0	0	0	0	0
DUX4L26	0	7.62038154045556 (9.4993622492865e-08)	0	0	6.88492744950504 (6.26357524857607e-07)	7.61533174098815 (1.88618591506127e-06)	0	0	0	0
DVL1	0	0	1.54970125009655 (0.0024180391354617)	0	0	0	0	0	0	0
DYNC1I1	2.86744635771893 (4.52063302178604e-05)	0	3.434809775656 (3.62917291293001e-09)	0	0	0	0	0	0	0
DYNC1LI2	0	0	0	0	0	-1.71418140104269 (6.17127117578912e-06)	0	0	0	0
DYNC2H1	0	0	0	0	0	0	0	-3.36310322632691 (0.00701279329905616)	0	0
DYRK1B	0	0	2.1787479746257 (0.00917848395902627)	0	0	0	0	0	0	0
DYRK3	0	0	0	0	0	0	0	0	-2.04210650006243 (0.00177046080521046)	0
DYSF	0	0	0	0	0	0	0	0	0	-2.64172368291817 (7.74212667787121e-05)
DZIP1L	0	0	0	0	0	0	0	0	-2.03756082308508 (0.00531936679419367)	0
E	0	0	0	0	0	0	23.0319811707045 (2.09042671528484e-22)	0	0	0
E2F2	0	0	0	0	0	0	0	4.45536535995928 (0.00029378399382908)	0	0
E2F8	0	0	0	0	0	0	0	5.2476513685161 (0.000140922816728853)	0	0
EAF1	-1.67614185602112 (0.00390789988712038)	0	-2.22127959020917 (0.000261338904415673)	0	0	0	0	0	0	0
EBF1	1.71937883480556 (1.20910067486925e-06)	0	1.60996366360669 (0.000707653744368149)	0	0	0	0	0	2.51837885386639 (2.60231045238772e-10)	2.94317446407133 (3.63120324130505e-05)
EBF2	0	0	2.83054151960082 (8.06190847166405e-06)	0	0	0	0	0	0	0
EBF3	4.49915741832563 (0.00166021639123207)	0	7.05031141365851 (2.55190856713457e-11)	0	0	0	3.65477185328932 (0.00849092654433561)	0	3.74668280026837 (0.00540362582584622)	0
EBI3	0	0	0	0	0	0	0	0	7.9452964036826 (2.09834592310647e-13)	0
EBP	0	0	0	0	0	2.0649625709593 (0.00502619700847682)	0	0	0	0
ECE1	0	0	0	0	0	0	0	-2.26476521369775 (0.002351660787103)	0	0
ECE2	0	0	0	0	0	4.48708651268299 (0.00495221306725377)	0	0	0	0
ECH1	0	0	1.90091048482619 (1.0315452573402e-06)	0	0	0	0	0	0	0
ECHDC1	0	0	-2.18210532066481 (3.05525633936143e-06)	0	0	0	0	0	0	0
ECHDC3	0	0	1.93930375578036 (0.00569446332522514)	0	0	2.4794110811245 (0.00890632862212233)	0	0	-2.75671754240678 (0.000524960477994534)	0
ECHS1	0	0	1.72131036518256 (6.53270335586429e-05)	0	0	3.15392208265283 (8.94418003355072e-06)	0	0	0	0
ECI1	0	0	2.56604801204263 (6.76651565068406e-05)	0	0	2.81953546747896 (0.000661559115816488)	0	0	0	0
ECI2	0	0	1.84430248313812 (5.69383436879507e-05)	0	0	1.87056354097194 (0.00240978297064843)	0	0	0	0
ECSIT	0	0	2.95044518355936 (1.30243993467802e-06)	0	0	0	0	0	0	0
ECT2	-1.57033346012439 (0.00270520551046215)	0	0	-3.59411043829948 (0.00706961228756519)	0	0	0	0	0	0
EDA	0	0	0	0	0	3.25837532376926 (0.00316042770370409)	0	0	0	0
EDA2R	0	0	0	0	0	0	0	0	-3.07100169155526 (0.000180917847703259)	0
EDAR	0	8.83438846949353 (1.89611992709485e-06)	0	0	0	0	0	0	0	0
EDARADD	0	0	0	0	0	0	0	0	3.92417149634884 (0.00344205956605936)	0
EDEM1	-1.75524222491197 (5.87797996550386e-06)	0	-2.1140435578296 (5.5389803141464e-07)	-1.72572807808585 (0.00873569615752653)	-1.72719470491192 (0.00324732872990467)	0	0	0	0	0
EDIL3	0	0	0	0	0	0	0	-6.23298530841616 (0.00539990548531383)	0	0
EDN1	-3.84791891274277 (0.000396244785982194)	0	-3.61763773523879 (1.77242518306292e-05)	0	0	-4.28213636791427 (0.000299847492256237)	0	-7.78934392638088 (0.000919350007166498)	-2.27125380460286 (0.000381810182975908)	0
EDN3	6.63988431851572 (2.50644920228944e-07)	0	0	0	0	0	0	0	0	0
EDNRA	-1.6149666408393 (0.000500040092893438)	0	0	0	0	0	0	-6.19816907863282 (0.00472103527141904)	0	0
EDNRB	0	0	0	0	0	0	0	-4.32965352218464 (0.000224008076564861)	0	0
EEF1A1P21	0	0	0	0	0	6.93572967077739 (0.00168787030662363)	0	0	0	0
EEF1A1P3	0	0	0	0	0	3.35355232839914 (0.00625007184278014)	0	0	0	0
EEF1A2	6.20811104104655 (5.59520479528291e-06)	0	12.4867336454888 (1.0423355913235e-33)	0	6.4677859897343 (0.00467623025217429)	0	0	0	0	0
EEF1DP3	0	5.83061951258306 (0.000166476584427283)	3.27386158306897 (7.70246006713474e-05)	0	0	0	0	0	0	0
EEFSEC	0	3.89021027579732 (0.00223584307856477)	0	0	0	0	0	0	0	0
EEPD1	0	0	0	0	0	2.19341425688436 (0.000174027014174768)	0	0	0	0
EFCAB1	0	0	0	0	0	0	0	0	-3.30995827687383 (0.0072415632405068)	0
EFCAB2	0	0	0	0	0	3.00797778081125 (0.0005694790129014)	0	0	1.81443027392102 (9.64613219211452e-06)	0
EFCAB5	0	0	0	0	0	0	3.98258958219093 (0.00832713620067127)	0	0	0
EFEMP1	0	-5.65411525034019	0	0	0	0	0	-6.65231914524505	0	0

		(0.0087746673605205)						(3.50784191272192e-05)		
EFHD1	0	0	2.19226820786311 (0.00120717788674638)	0	2.86592533878699 (0.00181383786558684)	0	0	0	3.70021013581214 (2.50250691115258e-12)	0
EFNA1	0	0	0	0	0	2.94237667578128 (0.000119374972888471)	0	0	0	0
EFNA5	2.04373853878086 (9.94545774446002e-05)	0	0	0	0	0	0	0	0	0
EFNB1	0	0	0	0	0	0	0	-3.39078497046168 (0.00762949225846703)	0	0
EFNB3	0	0	0	0	4.12836643402077 (0.00275378993088252)	0	0	0	0	0
EFR3A	0	0	0	0	0	0	0	-3.28273470435805 (0.000970835869811968)	0	0
EFR3B	0	0	3.00449138833268 (7.01790109172979e-06)	0	0	0	2.50048379320242 (0.00749568274791867)	0	0	0
EGF	0	0	2.68720450556572 (0.000657966502724129)	0	0	0	0	0	0	0
EGFL6	-6.21159655591714 (2.5040878746751e-08)	0	-5.94597571974122 (1.25951433029184e-09)	-7.48560604200599 (0.000839620297165527)	0	-3.61329393365796 (0.00121992210066635)	0	-6.42879054380605 (0.00428632278941609)	3.03428185133901 (0.000784705380601011)	-5.32237532401455 (0.00185303036875021)
EGFL7	-1.63039019774859 (0.00416696473894133)	0	0	0	0	0	0	0	0	0
EGFLAM	3.53378931802742 (0.00332243567966181)	0	5.39869955841008 (1.63774064852906e-07)	0	0	4.92454509478166 (0.00209151902288582)	3.88359010354436 (0.00610013564968224)	0	5.80204212151301 (1.52332543199995e-07)	0
EGFLAM-AS4	0	0	0	0	0	7.26986837985775 (0.00668205493536917)	0	0	0	0
EGFR	0	0	0	0	0	0	0	-4.97706027000903 (3.40329342776964e-05)	3.269700294684 (5.73049104091137e-34)	1.85168114163626 (0.0021460567739588)
EGFR-AS1	0	0	0	0	0	0	0	0	3.85685566725569 (6.1691797554278e-08)	0
EGLN1	0	1.98438542531459 (0.00944776497144531)	0	0	0	0	0	0	0	1.82367997740532 (0.00269035023194358)
EGLN3	0	0	2.75591081443125 (9.36994827273937e-06)	0	0	0	0	0	3.33954262414047 (3.66747598046391e-07)	3.11020302270044 (9.59863925080233e-05)
EGR1	0	0	0	0	0	0	0	-4.29785545772982 (3.72767235890276e-07)	0	0
EGR3	-3.03467114866513 (0.000427652681978266)	0	0	0	0	0	0	0	0	0
EHBP1	1.96091711526345 (2.11449452328338e-09)	0	0	2.02772409459263 (0.00362413882244694)	0	0	0	0	0	0
EHBP1L1	0	0	0	0	0	-1.78527717083477 (0.000286965809489774)	0	0	-1.68694279925865 (2.63828035122459e-07)	0
EHD2	0	0	0	0	0	0	0	-2.85491508653827 (0.000211736301812179)	0	0
EHD3	0	0	2.08318790189203 (0.000618329061034616)	0	0	0	0	0	0	0
EHF	-4.40156759302186 (2.39359878903358e-08)	0	-4.57099815027744 (1.50968620874184e-07)	0	0	0	0	-6.26615255390353 (0.00532615427949937)	-3.04882403134583 (0.000466971692088181)	0
EHHADH	0	0	0	0	0	3.34269906184011 (0.00125472017728176)	0	0	0	0
EIF1B	0	0	1.70011736174124 (0.000535838122096569)	0	0	0	0	0	0	0
EIF2AK1	0	0	0	0	0	0	0	2.39889952020137 (0.000210119378178662)	0	0
EIF3K	0	0	1.71946494101575 (1.63932184389299e-05)	0	0	0	0	0	0	0
EIF3LP1	0	0	0	0	0	6.11373970644227 (0.00112125932725744)	0	0	0	0
EIF4A1	0	0	0	-1.82014021966259 (0.0080642010704087)	0	0	0	0	0	0
EIF4E3	0	2.76080932491377 (0.00112467331461302)	0	0	0	0	0	0	0	0
EIF4EP1	-5.34476063565657 (0.000247205378296304)	0	0	0	0	0	0	0	-3.60392821081494 (0.00264936861116515)	0
EIF4G1	0	-2.74149199625434 (0.00760794152415122)	0	0	0	0	0	0	0	0
ELANE	0	0	0	0	0	0	0	7.65708022627153 (2.72643008879803e-16)	-4.52592604735276 (0.00109612728486234)	0
ELAVL4	6.64686834142949 (6.69979350840528e-09)	0	0	0	0	0	0	0	0	0
ELF3	-3.55323689174377 (6.36625369353151e-08)	0	-2.3419408814643 (0.00979831119824292)	-2.85217433769614 (0.00351107299527542)	0	0	0	-4.37824803647953 (0.00797116796421834)	0	-3.39666286845168 (0.000798342120088286)
ELF4	-1.79079821846223 (1.17792955863189e-05)	0	0	-2.30421555939791 (0.00676884146726355)	-2.65099242022555 (0.00439959260882754)	0	0	0	0	0
ELFN1	3.93702355523588 (0.00740522526459329)	0	4.82783164649864 (0.000169479356863085)	0	0	5.69152842778103 (0.00102998284641343)	3.9842110851991 (0.00519369998805048)	0	0	0
ELK3	0	0	0	0	0	0	0	-2.44734396137862 (0.00499170526535931)	0	0
ELL2	-1.84884679720377 (1.33627641923489e-05)	0	0	0	0	0	0	0	0	0
ELMOD1	0	0	0	0	6.47559966567641 (0.0042482603748159)	0	0	0	0	0
ELN	-1.66994101028934 (0.0013255948692813)	0	0	-1.90171041453741 (0.00751397059271611)	0	-2.30853051594575 (0.000323542361610495)	0	-5.65025277471011 (0.000392464118568619)	0	-2.28868761423535 (0.00140381977755809)
ELOF1	0	0	0	0	0	2.46195102458687 (0.00477290735495363)	0	0	0	0
ELOVL2	0	0	3.82074549517079 (0.00549195001173366)	0	0	5.46324109713805 (0.00841542142053734)	0	0	7.28943789362092 (4.80837829109892e-13)	0
ELOVL4	0	0	0	0	0	0	0	0	0	5.03728978074987 (0.000228948314932875)
ELOVL7	0	0	0	0	0	0	0	0	0	2.87777775716497 (0.00848898404682887)
ELP5	0	0	2.39314070202955 (4.12455046836847e-05)	0	0	0	0	0	0	0
EMB	0	0	-1.75254028696873 (0.00239415850254442)	0	0	0	0	2.31358752506413 (0.00867508423516673)	0	0
EMC10	0	0	2.17788904700053 (0.00228324844028087)	0	0	0	0	0	0	0
EMC4	0	2.44037437536178 (0.0085680476339667)	0	0	0	0	0	0	0	0
EMCN	0	0	0	0	0	0	0	-6.91544795544365 (0.00361056687300187)	-3.5753978211669 (0.00674153950395563)	0
EMILIN1	0	0	0	0	0	0	0	-2.97712362045931 (0.000317583603770838)	-1.95993732545785 (8.63701724077014e-08)	0
EMILIN2	-1.55639040198484 (0.00171908304892856)	0	0	0	0	0	0	0	0	0
EMILIN3	4.07628834384891 (0.00463222999212342)	0	0	0	0	0	0	0	0	0
EML1	1.77805972356826 (3.37144264390819e-05)	0	0	0	0	0	0	0	0	0
EML3	-1.89515429202055 (0.000371505407059133)	0	0	0	0	0	0	0	-1.88250350808963 (0.000111594909865392)	0
EML4	0	0	-1.62240701524979 (0.00037811542389034)	0	0	0	0	0	0	0
EML6	0	0	0	0	3.29652630756478 (0.000307647710372854)	4.38689422112734 (5.82125756885637e-05)	2.25347430875994 (0.0088132888450622)	0	0	0
EMP1	0	0	0	0	0	-2.39916318702945 (2.16897789400859e-07)	0	-7.23620572959434 (5.07816521193758e-06)	0	0
EMP2	0	0	0	0	-2.20331943350622 (0.000752173287110616)	-1.70388066914665 (0.000433350157163302)	0	-4.78019788209865 (1.51616075569959e-05)	-1.77124997501414 (0.000763693300557532)	0
EMR2	-3.26231651776072 (4.47716385912062e-05)	0	-3.82415091924863 (2.7964027795434e-06)	-5.21642161850469 (0.0030078921736094)	0	0	0	0	-2.64116286561819 (0.000315846519067557)	0
EMX2	0	0	0	0	9.5627863969876 (6.03158641505002e-13)	0	0	0	6.46987425171706 (0.000231850026021726)	6.79009821217895 (0.0026785245388961)
EMX2OS	0	0	0	0	8.98405647648738 (1.10355156978432e-13)	7.56701121292355 (0.00367244102585355)	6.37026415755622 (0.00465424494598992)	0	8.16644269303695 (4.17395364671523e-06)	8.1120397180827 (9.78983117200707e-07)
ENAH	0	0	0	0	0	0	0	-5.95154990308717 (9.24472561764117e-07)	0	0
ENAM	0	0	0	0	5.25178314552086 (0.00681977618508599)	0	4.1221967830803 (0.0024982814534203)	0	0	0
ENC1	-2.45202851252501 (0.000152452865726115)	0	0	0	0	0	0	0	0	0
ENDOG	0	0	3.47399257710637 (0.00443425822845997)	0	0	0	0	0	0	0
ENDOU	0	0	0	0	7.20697900913001 (0.00515462419084721)	0	0	0	4.93145499270914 (0.000390480788276757)	0
ENG	0	0	0	0	0	0	0	-1.99588836785434 (0.00915045105270396)	0	0
ENO3	0	0	6.25570458437185 (1.28737898610785e-21)	0	0	4.7850640004553 (2.46671860739036e-06)	0	0	0	0
ENOX1	2.78064764448718 (0.00131023100390535)	0	0	0	0	0	0	0	2.12504236204167 (0.00532762010242342)	0
ENPEP	0	0	0	0	0	0	0	0	1.56345435707023 (0.000626706750353145)	0
ENPP1	0	0	0	0	3.29705156713874 (0.00180855080473046)	4.00857572827936 (0.000117011752729458)	0	0	6.47174135782532 (7.94830938659561e-35)	0
ENPP2	0	0	-1.85744546776317 (0.0094706119508332)	0	0	0	0	0	2.41744348632506 (7.14784031052233e-08)	0
ENPP4	0	0	0	0	-2.55007649132185 (0.00770329796088211)	0	0	0	-2.60522767882421 (2.04042216687921e-08)	0
ENPP7	0	0	0	0	0	7.38349686985317 (4.10625165331924e-05)	0	0	0	0

ENTPD1-AS1	0	0	-2.15430186426633 (0.00520350450448554)	0	0	0	0	0	0	0
ENTPD5	0	0	0	0	0	2.97565247742137 (7.35672999072465e-07)	0	0	0	0
ENTPD7	-1.68146348894154 (0.000406802532265368)	0	-1.65182410192598 (0.00758532118021039)	0	0	0	0	0	0	0
EOGT	0	0	0	0	0	-3.27074621636906 (0.000482333878828865)	0	0	0	0
EPAS1	-2.36933671718694 (2.02165788884035e-08)	-2.17822203002541 (0.00950884398140551)	0	0	0	-1.84539044593655 (0.000324362761884871)	0	-4.33512661156643 (6.60424068821733e-08)	0	0
EPB41	0	0	0	0	0	0	0	2.61227166441033 (1.34011251622927e-05)	0	0
EPB41L2	0	-6.19829490917965 (0.0035047705118833)	0	0	0	0	0	-1.77188710798415 (0.00575927515344292)	0	0
EPB41L3	0	0	-1.8294553620268 (0.000199677511931906)	0	0	0	0	-3.02933906914623 (0.00145545395961305)	0	0
EPB41L4A	0	3.82443697365791 (7.18681614587226e-05)	0	0	0	0	0	0	0	0
EPB41L4B	0	0	0	0	0	3.89738751011678 (1.64736717283257e-07)	0	0	0	0
EPB41L5	0	0	-1.69697043528949 (0.00125568398026865)	0	0	0	0	-6.62037145071864 (0.00251443078679491)	0	0
EPB42	0	0	0	0	0	0	0	8.28417179577257 (8.75374912360955e-15)	0	0
EPCAM	-2.32185955995296 (4.30565992154572e-05)	0	-3.40311497842353 (3.44929610362162e-05)	0	0	0	0	0	-3.72945200417206 (7.22931552770165e-11)	-3.38745278050415 (0.00136796999853502)
EPDR1	-1.82667595648291 (0.00300765070175467)	0	0	-3.81946740545833 (0.00113758522011619)	0	0	0	0	0	-3.36469242550494 (0.0013375913073051)
EPHA1	0	0	0	0	0	2.92649426503071 (0.00946391622890051)	0	0	0	0
EPHA2	0	0	0	0	0	0	0	-6.17347341307594 (0.00786753009483371)	0	0
EPHA3	2.5875872081153 (0.000113369006473675)	0	2.19688771123994 (0.00306960681382438)	0	0	0	0	0	3.44027781847907 (0.00319411843142966)	0
EPHA4	2.71174465813379 (2.62595563725385e-08)	0	0	0	0	0	0	0	0	0
EPHA6	6.33097878226628 (4.13460218846744e-07)	0	0	6.74888257264414 (0.00352112695408781)	0	0	0	0	0	0
EPHA7	7.84909877986564 (3.64056356088699e-20)	0	0	8.49988973183006 (2.90083470459917e-17)	4.66345179612552 (0.000758898612565785)	0	0	0	0	0
EPHA8	0	0	0	0	0	6.51813181020835 (0.000996809447046881)	0	0	0	0
EPHB2	0	0	0	0	0	2.62919336165747 (0.00354730609610417)	0	0	0	0
EPHB6	0	0	0	0	4.13828804698413 (0.00137357866572131)	0	0	0	0	0
EPHX1	0	0	0	0	0	2.92389189715065 (3.40787588588363e-05)	0	0	-1.95156175312798 (1.2297715870297e-06)	0
EPHX2	2.18818625506856 (0.00242451223515305)	0	0	0	3.19127962661423 (0.00155410566595002)	3.69377879450562 (2.24876255900655e-06)	0	0	0	0
EPM2A	3.1114056143199 (2.47215662269059e-05)	5.00012788478104 (0.00035712872430955)	2.30101321009583 (0.00342615396665632)	3.56094549950733 (0.00769245728121975)	0	0	0	0	0	0
EPN1	0	0	1.87913889183085 (0.00010972839321639)	0	0	0	0	0	0	0
EPN3	0	0	0	0	0	5.67333246115441 (0.000351074131241554)	0	0	0	0
EPOR	0	0	0	0	0	0	0	3.30672179960391 (0.00326912232662992)	0	0
EPPIN	0	0	0	0	0	5.58530760092159 (0.00579178639951945)	0	0	0	0
EPPIN-WFDC6	0	0	0	0	0	6.96977162684898 (0.00897266232869622)	0	0	0	0
EPPK1	0	0	-4.95540776476591 (9.47310450902614e-08)	0	0	0	0	-6.64304444855463 (0.00377608255880693)	0	0
EPS8	0	0	0	0	0	0	0	-5.43098275160361 (0.000651407289518264)	0	0
EPS8L1	-2.28837505183812 (0.00457525504394921)	0	-2.99212552857163 (0.00148390034112177)	0	0	0	0	0	3.54825355420676 (2.59639801473487e-06)	0
EPS8L2	-2.39737138617981 (0.000212168483300582)	0	-3.11698146257622 (0.00032896435652315)	0	0	0	0	-6.6175040374626 (0.00387309124222074)	1.81537354328354 (0.000456931311371289)	0
EPSTI1	0	0	0	0	0	0	0	0	1.64670320263801 (0.004071793172834)	0
EPX	0	0	0	0	0	0	0	7.03130079722076 (9.91594863264378e-06)	0	0
ERAP1	0	0	0	0	0	-1.59182501467161 (0.00646689611440267)	0	0	0	0
ERAP2	0	0	-2.9916752071964 (8.07699106659025e-06)	-4.65338843875927 (0.000556567790958613)	0	0	0	0	0	-3.34891577428846 (0.0015609883135184)
ERBB3	0	0	-3.83113534971804 (2.77627942334585e-05)	0	0	0	0	0	0	0
ERBB4	0	0	0	0	0	0	0	0	-2.22700186511214 (0.00980696378219729)	0
ERI1	0	0	-2.15959558134569 (0.000362907707631437)	0	0	0	0	0	0	0
ERICH3	0	0	-4.37247523961024 (0.000316143103571469)	0	0	0	0	0	-4.98697968398884 (1.988252499354e-06)	-7.25429509359619 (0.00345084050355869)
ERICH3-AS1	0	0	0	0	0	6.46475271615364 (0.00375024932752695)	0	0	0	0
ERLIN1	0	0	0	0	0	0	0	1.85034367104686 (0.00914109432471395)	0	0
ERMAP	-1.81609931272398 (0.00723239237537747)	0	0	0	0	0	0	3.0600127805077 (0.000523695030247612)	0	0
ERMARD	0	3.85615033622566 (0.000171965779350102)	0	0	0	0	0	0	0	0
ERMP1	0	0	0	0	3.295357769104 (5.61750071708587e-06)	0	0	0	0	0
ERN1	-1.74424127656517 (0.00102742179260943)	0	-1.97879603058078 (2.60949654530058e-06)	0	0	0	0	0	0	0
ERN2	0	0	0	0	0	5.91958287813631 (0.00104574956850468)	0	0	0	0
ERO1L	0	0	-2.01424893004931 (6.24085845243738e-06)	0	0	0	0	0	0	0
ERRFI1	-1.57921901715225 (0.00099014006457398)	0	-3.43443590172018 (8.86187253573892e-11)	0	0	0	0	0	0	0
ERV3-1	0	0	0	0	0	0	0	0	1.74239903282124 (0.0047550996238475)	0
ERVFRD-1	0	0	0	0	0	0	0	0	4.21916683634123 (1.09611283094093e-06)	0
ERVH48-1	0	0	0	0	0	3.67053720320766 (0.00546278092650376)	0	0	6.80016363129451 (1.68574936283242e-22)	0
ERVMER34-1	0	0	0	0	0	0	0	0	3.48682425892565 (0.000193438313649773)	0
ERVV-1	0	0	0	0	0	0	0	0	5.61582898751383 (5.06548034009564e-06)	0
ERVV-2	0	0	0	0	0	0	0	0	6.30066204250094 (2.04611220493817e-06)	0
ERVW-1	0	0	0	0	0	0	0	0	6.18292218196993 (1.59927793077941e-17)	0
ESAM	-1.80458829833042 (3.33319944323913e-06)	0	0	0	0	-2.31410628592882 (0.006968329229875)	0	-5.048531381188 (0.00152575118019552)	0	-1.86535341341909 (0.00954646129562885)
ESF1	0	0	0	0	-1.64494836839208 (0.00656182248614438)	0	0	0	0	0
ESM1	-5.96085678781757 (3.15949351208802e-07)	0	-5.47416712836799 (1.302136677087e-07)	-7.08927475807836 (0.00193698526727478)	0	-3.14522386183907 (0.00864845654984431)	0	0	-6.87507065835152 (5.76326163556922e-12)	-7.37002321460468 (0.00126480150361247)
ESPL1	0	0	0	0	0	0	0	5.08513846065045 (7.57227450843428e-05)	0	0
ESPN	0	0	0	0	0	5.53475579128948 (4.13167323636672e-05)	0	0	0	0
ESR1	0	0	0	0	0	2.50735744338304 (0.00460318385050637)	0	0	0	0
ESRG	0	0	0	0	0	6.23575170660495 (0.00732756258299777)	0	0	0	0
ESRP1	-3.3952687676323 (3.19523898244654e-05)	0	-4.57706666480502 (3.38690606369746e-06)	0	0	0	0	0	0	0
ESRP2	-3.62444350001813 (8.17898171876417e-06)	0	-2.77541085406364 (0.000799582815871942)	-6.39229915883125 (0.00479068075988943)	0	1.86781494192495 (0.00358794888394797)	0	0	0	0
ESRRA	0	0	2.3087061569068 (4.20853400486781e-05)	0	0	0	0	0	0	0
ESRRG	3.73536491863603 (0.000448182321527824)	0	4.72679025785964 (2.00056648448672e-07)	0	4.68886162047033 (0.00361342284067454)	5.34941120652389 (0.000737390659168879)	0	0	6.14487094500062 (3.31129174183444e-07)	0
ETFA	0	0	0	0	0	1.62392360888725 (0.00136108860281836)	0	0	0	0
ETFB	1.57829080566716 (0.00157949726778333)	0	2.82248204775992 (8.71044652185514e-09)	0	0	2.7891875894579 (2.1679120939611e-06)	0	0	0	0
ETFDH	0	0	2.62340229745316 (6.80812729072029e-08)	0	0	2.80209964211769 (0.000870754377141019)	0	2.41703403607081 (0.0082915146802826)	0	0
ETNK2	0	0	0	0	0	3.66061968657164 (0.000296269153054377)	0	0	0	0
ETS1	-1.84517866203579 (3.96737415015797e-06)	0	0	0	0	-1.72981834573643 (0.00843019603730982)	0	0	0	0
ETS2	0	-6.1335464830518	0	0	0	0	0	-3.68299752388691	0	0

		(0.00394168829444415)						(1.51612321450745e-06)		
ETV1	0	0	0	0	0	0	0	-4.4890605853024 (0.00923843968098808)	-3.31413028656708 (3.76985635632028e-08)	-5.82617049789133 (0.000678165657673273)
ETV7	0	0	0	0	0	0	2.38799438056828 (0.0044649432621078)	0	0	0
EVA1A	0	0	0	0	0	2.32996470965195 (0.00778467808126902)	0	0	1.88925177066787 (0.00046558478927205)	0
EVC	0	0	0	0	2.01581715647287 (0.00187071186974142)	0	0	0	0	0
EVI2A	0	0	-3.23008557759949 (0.000419319369921676)	0	0	0	0	0	0	0
EVI2B	-3.0807173684646 (5.56834794910761e-05)	0	-2.80585384334186 (6.65727020969798e-05)	0	0	0	0	0	-2.20198740838851 (0.000224708665752829)	0
EVI5L	0	0	1.63667249001417 (0.0025094300370654)	0	0	0	0	0	0	0
EVPL	0	0	-5.49573285000642 (3.26589484298867e-10)	0	0	0	0	0	0	2.18670471126832 (0.00363686264226308)
EXO1	0	0	0	0	0	0	0	4.82923170944352 (5.76195494199677e-05)	0	0
EXOC3	0	0	0	0	0	1.85933794720761 (0.00781017857993392)	0	0	0	0
EXOC3L2	0	0	0	0	4.90747901698481 (0.00341777352472239)	0	0	0	0	0
EXOC3L4	0	0	0	0	0	3.5065076390026 (0.000279005175268451)	0	0	0	0
EXOC6	0	0	0	0	0	0	0	0	1.82546741262558 (0.00066955275872101)	0
EXOC6B	0	0	2.0997527772944 (4.8508434236634e-10)	0	0	0	0	0	2.20211521609579 (1.53233867812279e-12)	0
EXPH5	-3.42695987450504 (1.03270387719446e-10)	0	-4.16781907228096 (1.25090836181212e-09)	-7.70167608607177 (0.000364837582646483)	0	0	0	0	2.78785572278322 (2.28121416957399e-05)	0
EXTL1	0	0	0	0	0	5.40003992969234 (0.00950890912101097)	0	0	0	0
EXTL3	0	0	1.58152390496954 (2.6466756617903e-06)	0	0	0	0	0	0	0
EYA4	0	0	1.92296823728939 (0.00727270166031708)	0	0	0	0	0	0	0
EZH2	-3.23863204327464 (0.00114617981550597)	0	0	0	0	0	0	3.75830406798319 (2.79224849297782e-05)	0	0
EZR	-1.62390282464278 (4.85831263574859e-07)	0	0	0	0	0	0	0	0	0
F10	0	0	0	0	0	3.90601507175026 (2.88095158738601e-05)	0	0	0	0
F11	0	0	0	0	0	6.60128674202477 (8.44299619266408e-06)	0	0	0	0
F11-AS1	0	0	0	0	0	6.20619434883014 (0.00115833211091622)	0	0	0	0
F11R	-2.62192107630889 (1.32813224106685e-06)	0	0	-3.89832055875191 (4.37342267375719e-05)	0	0	0	0	0	0
F12	0	0	0	0	0	7.86944488349399 (4.88842583284396e-10)	0	0	0	0
F13A1	0	0	0	0	0	-2.08546138578378 (0.00584453156220995)	0	0	1.99516503601921 (0.00105056972972202)	0
F13B	0	0	0	0	0	6.25348140923245 (0.000442414708190049)	0	0	0	0
F2	0	0	0	0	0	11.0833764153581 (4.18234656252839e-22)	0	0	0	0
F2RL3	-3.58236927296291 (2.12574416893535e-07)	0	0	0	0	0	0	-3.05415253795837 (0.00652349889589429)	-5.86951519114958 (2.83520964718447e-15)	0
F3	-1.99651173779072 (9.83171314556379e-06)	0	-1.95768786812475 (7.69652910944576e-05)	0	-4.32552514832957 (1.0278963784766e-05)	-2.60984242196653 (0.000744719719540076)	0	-8.28102187735598 (0.000141479182100546)	0	0
F5	0	0	0	0	0	3.78869673697538 (2.513957253293e-07)	0	0	4.22202170571694 (2.93092687016288e-08)	0
F7	0	0	0	0	0	8.18808183000257 (2.69385336887149e-06)	0	0	0	0
F8	0	0	1.86479412904857 (0.00151169612075058)	0	0	0	0	-3.49113793871099 (0.00214158899352697)	-2.77315771388936 (4.20802526008133e-08)	0
F9	0	0	0	0	0	10.2205798033014 (5.19699848806861e-14)	0	0	0	0
FABP1	7.77112576526443 (1.00926021092001e-11)	0	0	8.07082493173434 (1.62201609851366e-06)	7.53239484060353 (0.00164711516668102)	12.3744712574101 (2.18185864810697e-20)	0	0	0	0
FABP3	0	0	7.47353165942898 (1.01414779535585e-50)	0	0	0	0	0	0	0
FABP4	2.63606561926645 (0.00014208871972065)	6.42237773397303 (3.86216755865923e-10)	4.47569978104781 (3.86776884951927e-12)	3.84783997153831 (0.000793193190044237)	4.22843966333123 (0.000103660980847942)	0	0	0	0	6.66649316754238 (1.37094679939468e-11)
FABP5	0	3.10088794868212 (0.00658084832245437)	0	0	0	0	0	0	0	3.12922211122255 (0.000872412796430942)
FABP6	5.96394279419918 (0.000106711426693973)	0	0	5.13434656951875 (0.00741755722281349)	0	0	0	0	0	0
FADS1	0	0	-1.68989828691428 (0.000120009789030304)	0	0	0	0	0	0	0
FADS6	0	0	0	0	0	5.97928386444677 (0.0018041296097416)	0	0	0	0
FAH	0	0	0	0	0	1.89799889644526 (0.00455480195042038)	0	0	-2.52158214838713 (1.46076479626951e-06)	0
FAHD2A	0	0	0	0	0	1.9244294673341 (0.00253606979863288)	0	0	0	0
FAIM2	3.99373093079014 (9.08892394026584e-08)	0	3.57515496730254 (0.000561152599715029)	4.69085313552243 (0.00086438201827885)	0	4.40533612180643 (0.00193060984004423)	0	0	0	0
FAIM3	0	0	0	0	0	0	0	4.41848990718668 (0.00584692511033528)	0	0
FAM101B	0	3.09824955881349 (0.00206605583267777)	0	0	0	0	0	2.66340314170157 (0.00234385366697215)	0	0
FAM102B	0	0	-1.60527350383259 (0.00197777783384286)	0	0	0	0	0	0	0
FAM105A	-1.77680092098774 (0.000494906232439329)	0	-3.71215585671286 (3.77120808036228e-05)	0	0	0	0	0	0	0
FAM107A	0	0	1.6725287930877 (9.53252478369303e-05)	0	0	-2.78864040045693 (0.00134742769561319)	0	-7.19335315017051 (0.00100012541274375)	0	0
FAM107B	0	0	-2.00099322255149 (0.00391196232271117)	0	0	0	0	0	0	0
FAM110A	-4.26886337251396 (0.00769977662463985)	0	0	0	0	0	0	0	0	0
FAM110B	2.40417712048144 (0.00444040619428821)	0	2.8954422943541 (0.00102829484234082)	0	0	0	0	0	0	0
FAM110C	-2.30273221516102 (0.00258716137211855)	0	-2.55750728379043 (0.00544924389827966)	0	0	0	0	0	0	0
FAM110D	0	0	3.24700560112684 (0.00209392405344221)	0	0	0	0	0	0	0
FAM114A1	0	0	0	0	0	0	0	-2.41961014805731 (0.00344056501512362)	0	0
FAM117A	0	0	0	0	0	0	0	3.47547742979376 (2.12844439981781e-05)	0	0
FAM120A	0	-5.68051986231687 (0.00749898815574005)	0	0	0	0	0	0	0	0
FAM124A	2.97472170500575 (4.95113701408307e-05)	0	0	0	0	0	0	0	0	0
FAM124B	0	0	0	0	5.18178620803368 (0.000106081427302151)	0	0	0	0	0
FAM126A	0	0	0	0	-1.65456347639972 (0.00319496662948974)	0	0	-2.55249334232432 (0.00109454735683717)	0	0
FAM127A	0	0	1.54280647825592 (0.000142593220675414)	1.6299094112047 (0.00795187283239537)	0	0	0	0	0	0
FAM129A	1.76130634581016 (1.50411957772987e-05)	0	1.66946645361715 (9.14786022862276e-05)	0	0	0	0	0	0	0
FAM129B	-2.12732474970682 (2.80537880413235e-06)	0	0	0	0	-1.7234176853158 (0.00580006594718477)	0	-2.51890672821218 (0.00261457143365777)	0	0
FAM129C	0	0	0	0	4.82195123744511 (0.0026114399527988)	4.72333145217506 (0.00804033063057753)	3.89547255295112 (0.00735226260556564)	0	0	0
FAM131B	0	0	0	0	0	0	4.53622728035296 (0.00474087070961835)	0	0	0
FAM132B	0	0	0	0	0	4.54223204298541 (0.00452699492355372)	0	6.74783964295905 (6.02741403852413e-09)	0	0
FAM134B	0	0	2.20449029848244 (0.000198879028915883)	0	0	0	0	0	0	0
FAM135B	0	0	0	0	5.89016098644507 (0.0034468858259432)	6.71352841298921 (0.00069434683621508)	0	0	0	0
FAM13A	0	0	0	0	0	0	0	0	2.16877286480797 (5.31888224104019e-06)	0
FAM13C	2.25605596978393 (9.56307158516861e-05)	0	0	0	0	0	0	0	0	0
FAM154A	0	0	0	0	0	7.44279116366548 (0.00404563973443074)	0	0	0	0
FAM155B	0	0	5.61235072404791 (1.12418964807482e-06)	0	0	0	0	0	0	0
FAM162A	0	0	0	0	0	1.79653522388221 (0.000585503400426854)	0	0	0	0
FAM162B	0	0	0	0	0	0	0	0	3.17609514637389 (0.00250880796058261)	0
FAM163B	0	0	0	0	0	5.6481706121049 (0.00204037755378022)	0	0	0	0

FAM169A	0	0	0	0	0	2.96706617514495 (0.00935009351468745)	0	0	0	0
FAM169B	0	0	0	0	0	5.98240123705944 (0.00244308965013444)	0	0	0	0
FAM171B	0	0	0	0	0	0	0	0	2.19002222492719 (0.00558147653704388)	0
FAM177B	0	0	0	0	0	0	0	0	4.48374611025047 (0.00562708703585619)	0
FAM179A	0	0	0	0	0	4.24811632937352 (0.000542080103761626)	0	0	-3.77881371236652 (0.00159819696674265)	0
FAM180A	0	0	0	0	0	0	0	0	-3.27620686044913 (0.00404865379734083)	0
FAM183CP	0	0	0	0	6.24434293510046 (0.0075784156376657)	0	0	0	0	0
FAM184A	0	0	0	0	0	0	0	0	1.92083703274289 (0.00827637622083258)	0
FAM189A2	0	0	1.9150942346216 (0.00431182038233905)	0	0	0	0	0	0	0
FAM195A	0	0	2.26902010632198 (0.000693586068270207)	0	0	0	0	0	0	0
FAM195B	0	0	2.17685319183494 (0.000364806367260132)	0	0	0	0	0	0	0
FAM196B	-3.83032308242298 (0.00270623661964716)	0	0	0	0	0	0	0	0	0
FAM19A2	0	0	0	0	0	4.0141245195689 (0.00681684840946746)	0	0	0	0
FAM205CP	0	0	0	0	0	5.56734392333371 (0.00742251880135109)	0	0	0	0
FAM20A	0	0	-1.90930642529825 (0.00236429631956506)	-4.72739594517596 (0.000395923522061689)	0	0	0	-3.67034102724064 (0.00644155152925002)	-1.8940215810326 (9.00930957666178e-05)	0
FAM20C	0	0	0	0	0	0	0	-3.19826310788386 (0.00441249878187253)	-2.55979919572488 (1.6779637940238e-08)	0
FAM212B	0	0	1.63708299386914 (0.00382527114924201)	0	0	0	0	0	0	0
FAM213A	1.52821866316547 (0.0027978942467982)	0	1.5769685611139 (0.00158262220822251)	0	1.92355083564037 (0.00870497089898005)	1.70540337098795 (0.00463180033921469)	0	0	0	0
FAM213B	0	0	0	0	0	0	0	0	-1.62869643842836 (0.002560421578758)	0
FAM216B	0	0	-4.4727436832955 (0.000381796474819542)	0	0	0	0	0	-5.11326053353045 (1.04627235757083e-05)	0
FAM221B	0	0	0	0	0	6.5263999083892 (0.00590764642395917)	0	0	0	0
FAM222A	0	0	4.90051068692195 (8.42586154870023e-09)	0	0	4.16882916819376 (0.00328885535951378)	0	0	0	0
FAM229B	2.52105227669084 (0.0028465767269654)	0	0	0	0	0	0	0	0	0
FAM26F	0	0	0	0	0	4.59229896645823 (0.00258614622996577)	3.46363683675598 (0.000543902335722554)	0	2.97740072697614 (0.00727352314540328)	0
FAM3A	0	0	1.65773909780732 (0.00787199809557134)	0	0	0	0	0	0	0
FAM3C2	0	0	0	0	0	0	0	0	3.23158093046883 (0.00227549103445168)	0
FAM43A	0	0	0	0	0	0	0	0	1.84159603809707 (0.00299263144869105)	0
FAM45A	0	0	0	0	0	1.95246954247407 (0.00601186248228429)	0	0	0	0
FAM46A	0	0	0	0	0	0	0	0	2.47581717143362 (1.51986735073984e-07)	0
FAM46C	-2.2417054983067 (0.000279806361538406)	0	0	0	0	0	0	3.00974051623064 (0.000133047404129967)	0	-3.72832239661716 (0.000721152648269983)
FAM47E	0	0	0	0	0	4.49730428116197 (0.000845056956777653)	0	0	0	0
FAM47E-STBD1	0	0	0	0	0	1.78361668414086 (0.000213982617341542)	0	0	0	0
FAM49A	0	0	-2.59084481251717 (0.000262257177621233)	0	0	0	0	0	0	0
FAM49B	-1.68739773764835 (0.00025772409466599)	0	-1.76413327771549 (0.000359668937362393)	-3.90872705730822 (9.63409903081135e-05)	0	0	0	0	0	0
FAM50B	0	0	3.40694380266375 (0.000450642369986023)	0	0	0	0	0	0	0
FAM63A	0	0	2.67460214598881 (6.01603366480058e-07)	0	0	0	0	0	0	0
FAM65A	0	0	0	0	0	-1.86604359778998 (8.7725496864212e-05)	0	0	0	0
FAM65B	-2.86428937047616 (1.80378495228702e-07)	0	-2.63438899733279 (1.0208739041688e-06)	-2.59597670263853 (0.00359826373674031)	0	0	0	0	0	-3.57498334465831 (0.000158355593137549)
FAM65C	0	0	0	0	2.60962648769654 (0.00539996464877543)	0	0	2.96770770296312 (0.00311534801981999)	0	0
FAM66C	0	0	0	0	0	0	0	0	2.94446871259369 (0.00205311533018963)	0
FAM69A	2.82915672089012 (2.8054416461069e-06)	0	0	0	0	0	0	0	2.43739063355679 (2.19627090394438e-06)	0
FAM71F2	0	0	0	0	0	4.90086965185041 (0.00214890287319707)	3.67955796002478 (0.00415391855735602)	0	0	0
FAM76B	0	0	0	0	0	0	0	2.24326273191864 (0.00950169637216466)	0	0
FAM78A	0	0	2.03770878151896 (0.00461594673299037)	0	0	0	0	0	0	0
FAM81B	0	0	0	0	0	0	0	0	-4.23877777301004 (0.00784826524278098)	0
FAM83B	0	0	0	0	0	0	0	0	4.75526337089711 (2.36999132042775e-11)	6.13058865101483 (1.50140994928955e-07)
FAM83D	5.05365708147601 (8.86723221228531e-16)	0	0	6.39485996723408 (1.53790842164003e-13)	0	0	0	0	0	0
FAM83F	0	0	0	0	5.41318855190045 (1.80751406096331e-06)	4.44512683006808 (9.96339088737133e-05)	0	0	0	0
FAM83G	-3.10795223138819 (0.00101613079431705)	0	0	0	0	0	0	0	0	0
FAM83H	-3.96096774552313 (4.7153031673627e-05)	0	-3.70340716313111 (0.000354667415158487)	-6.78597245212605 (0.00444065469699365)	0	0	0	0	0	0
FAM86B3P	0	0	0	0	0	0	0	0	-3.15448269697511 (0.00398142048955533)	0
FAM89A	-2.29242839134958 (0.00319897638903499)	0	0	0	0	0	0	0	2.75429604074008 (4.79780059324333e-09)	0
FAM91A1	0	0	-1.80313895644098 (0.00194311396160279)	0	0	0	0	0	0	0
FAM98C	0	0	0	0	4.93125148067571 (2.19610414154552e-05)	3.35689719354636 (0.000712754764369298)	2.23190291314889 (0.00505397172899737)	0	0	0
FAM99A	0	0	0	0	0	7.08235576184144 (0.000441293498865441)	0	0	0	0
FAM9B	0	0	0	0	0	7.68498254587203 (0.000326875263062371)	0	0	0	0
FAM9C	0	0	0	0	0	6.47744949093614 (9.37797799656859e-05)	0	0	0	0
FANCA	0	0	0	0	0	0	0	4.18903815509487 (4.1020751009738e-05)	0	0
FANCD2	0	0	0	0	0	0	0	3.99435588630233 (2.51684251047427e-05)	0	0
FANCI	0	0	0	0	0	0	0	3.93608269693666 (1.19088353909617e-06)	0	0
FANCL	0	4.25530251104334 (0.000528559295890528)	0	0	0	0	0	0	0	0
FAP	0	0	-2.47760425767558 (0.00182489648331723)	0	0	0	0	-6.13266095538977 (0.00740591478164685)	0	0
FAR1	0	0	0	0	0	-1.71621887240465 (0.0017659937853641)	0	0	0	0
FAR2	0	0	0	0	0	0	0	3.28847094348188 (0.000213045770008064)	0	0
FAR2P1	0	0	0	0	0	6.83616064796014 (0.00210048989075515)	0	0	5.69802230181345 (1.44442162137637e-05)	0
FAR2P4	0	0	0	0	0	0	0	0	3.7380951589977 (0.00810043758714868)	0
FARP1	0	0	0	0	0	0	0	-2.73065823579294 (0.00268415727265273)	0	0
FAS	-1.69551993455366 (9.89348644783232e-06)	0	-2.33401377427684 (5.68822592566231e-06)	-3.11141952348999 (0.000207853160701433)	0	-2.36120546279463 (0.000318750549465368)	0	-2.27643192982305 (0.00983202377092647)	0	0
FASN	-2.16213405050584 (5.95423719153819e-08)	0	0	0	0	0	0	0	-1.6690631621937 (1.36205088265271e-06)	0
FASTK	0	0	1.76739249234749 (0.0022560035673402)	0	0	0	0	0	0	0
FASTKD2	0	2.89448413729362 (0.00475260231356514)	0	0	0	0	0	0	0	0
FAT1	0	0	0	1.50453386510675 (0.00985977934590245)	0	0	0	-4.84555986410709 (2.75896286343799e-07)	0	0
FAT2	0	0	0	0	0	0	0	0	2.1225081430174 (0.000827712362499574)	4.97125310246231 (5.22247933054111e-07)
FAT3	-2.99108363404953 (0.00239439295395372)	0	0	0	0	0	0	0	0	0
FAT4	0	0	0	0	0	0	0	-4.47276410728298 (8.36491940935322e-05)	0	0
FAXC	5.15403196300271	0	0	0	0	5.66613112869483	4.09419867678232	0	0	0

	(1.80147743500933e-10)					(0.00169937316277783)	(0.00178660341752519)			
FBF1	0	0	0	0	2.62084627955673 (0.0096034674068381)	0	0	0	0	0
FBL	0	0	0	0	0	0	0	2.29843631649485 (0.000689392422236796)	0	0
FBLN1	0	-5.7560777165854 (0.00706807538418602)	0	0	0	-2.43896647262051 (8.15549410647808e-05)	0	-2.51631919664495 (0.000617505889580273)	0	0
FBLN2	0	0	0	0	0	-2.17205221772761 (0.00569447442800406)	0	-5.01213586875907 (0.00297500183505705)	0	0
FBLN5	0	0	0	0	0	0	0	-5.88400810741697 (0.000292378485107739)	0	0
FBN1	0	0	0	0	0	0	0	-3.5095530932651 (3.59936841256932e-05)	2.33019491345771 (7.39000489302527e-10)	0
FBN2	0	0	1.99169982891495 (0.00882929431708053)	0	0	0	0	0	7.24165639898286 (2.90641274924133e-41)	0
FBP1	-2.51273803041741 (0.00529658498990736)	0	0	0	0	0	0	0	-2.92174175467462 (0.00303410561653423)	0
FBXL16	0	0	0	0	3.82270845852218 (0.00455109450777988)	0	0	0	0	0
FBXL22	5.02135128198552 (3.08250727943315e-05)	0	0	0	0	5.83813468070524 (0.000864449721664743)	0	0	0	0
FBXL6	0	0	0	0	0	3.07901949311327 (0.0033157547620493)	0	0	0	0
FBXO2	0	0	0	0	3.79272912319274 (0.000544468660117983)	0	0	0	0	0
FBXO27	0	0	0	0	0	4.05803332261114 (0.00882746192510256)	0	0	0	0
FBXO30	1.5526368437867 (4.32666986018293e-06)	0	0	0	0	0	0	0	0	0
FBXO31	0	0	1.61394923522913 (0.000831457740187206)	0	0	0	0	0	0	0
FBXO32	4.65771148593195 (9.82624496091568e-21)	0	2.22366394060212 (6.30753759694721e-05)	3.35699954671119 (0.000116470001044632)	0	0	0	0	2.16495696047072 (0.000137298625132882)	0
FBXO33	0	0	0	0	0	1.96470777746306 (0.00888840298293159)	0	0	0	0
FBXO40	0	0	9.98836411387152 (8.61878708269375e-19)	0	0	6.62397853114318 (0.00357156159371329)	0	0	0	0
FBXO41	0	0	0	0	3.38606834267646 (0.0091656633547224)	3.41823819325447 (0.00595798687607894)	0	0	0	0
FBXO44	0	0	0	0	0	0	0	0	-3.16258645204571 (0.000859061682720239)	0
FBXO7	0	0	0	0	0	0	0	2.11108981502761 (0.00153148388278217)	0	0
FBXO8	0	0	0	0	0	1.81742009360574 (0.00407007986625491)	0	0	1.79616089914342 (9.67329895614992e-07)	0
FBXW5	0	0	2.18733897110515 (8.81856199278745e-05)	0	0	1.80696686315131 (0.00527560216927943)	0	0	0	0
FCAMR	0	0	0	0	6.51680923550324 (0.00027447236007066)	0	0	0	0	0
FCAR	-3.13840579341532 (0.00158526560886397)	0	-2.7557989862777 (0.0069235308926295)	0	0	0	0	3.42710985920167 (0.00154837242662843)	-3.39386473083145 (1.6634280799233e-05)	0
FCER1G	0	0	-2.86142153937098 (0.000797771031285029)	0	0	0	0	0	0	0
FCGBP	0	0	0	0	3.40924827160675 (1.85259334267084e-06)	1.89345194941872 (0.00966671676932154)	0	0	2.55259363180817 (7.18077535734195e-06)	3.73119179709237 (2.31314799886815e-05)
FCGR2A	0	0	-2.44486154493884 (0.000767414000204652)	0	0	-2.02794617427242 (0.00819021307696959)	0	0	0	0
FCGR2B	0	0	0	0	0	2.69068874291646 (0.00580207703113181)	0	0	3.87706036838573 (1.17436853289119e-07)	0
FCGR2C	0	0	0	0	0	0	0	0	2.93788791987493 (2.03127209877731e-05)	0
FCGR3B	-3.85408759877124 (3.27948809529934e-05)	0	0	0	0	0	0	0	0	0
FCHO2	0	0	-1.90455674517084 (0.000477847705733247)	0	0	-1.79675362697226 (0.00406151378565121)	0	0	0	0
FCN1	-3.5028793539767 (2.18959628825067e-05)	0	0	0	0	0	0	0	0	-3.98194936141741 (0.00423260384951026)
FCN2	0	0	0	0	0	7.34490710555804 (2.85675639003488e-05)	0	0	0	0
FCN3	-5.53403503362276 (2.44345690221795e-11)	0	0	-8.33294790231235 (0.000201219073430046)	-5.38823191452784 (0.00013801717259808)	0	0	-7.27063094973345 (0.00122019347141192)	-4.92387767691269 (1.53410541908675e-12)	-8.6107030347615 (0.000121882507897442)
FCRL2	0	0	0	0	0	4.24815598138949 (0.0022272894682295)	0	0	0	0
FCRL4	0	0	0	0	0	6.54401974831726 (0.00335150846722125)	0	0	0	0
FDX1	0	0	0	0	0	0	0	0	2.23489705597746 (6.8505143572497e-05)	0
FDXR	-2.72117643887586 (0.00378827243893)	0	0	0	0	0	0	0	-2.42183128133552 (0.000731552044561673)	0
FECH	0	0	0	0	0	0	0	3.51680180843042 (1.13913211658514e-05)	0	0
FEM1A	0	0	2.57246317574569 (3.16462530907022e-06)	0	0	0	0	0	0	0
FEM1AP4	0	0	0	0	0	6.16856019215855 (0.00609985852466005)	0	0	0	0
FEN1	0	0	0	0	0	0	0	3.40154569618859 (0.00171319559972467)	0	0
FENDRR	2.33044844134441 (6.62523541846322e-05)	0	-5.17029543946288 (1.95248657817741e-06)	0	0	0	0	-6.18638541788607 (0.00842733775386136)	-2.07373801902465 (0.00576601274627309)	-7.5270758753666 (0.00127043197242817)
FER1L4	0	0	0	0	0	0	0	0	2.72566685536463 (0.000864764806427462)	0
FERMT1	0	0	0	0	0	0	0	0	2.60941897734371 (8.57157710460806e-05)	0
FERMT2	0	0	0	0	0	0	0	-4.02617731088247 (2.61590384846411e-05)	0	0
FERMT3	0	0	-1.62607276623206 (0.00594937343951453)	0	0	0	0	1.97454566500943 (0.00811269410883732)	0	0
FEZ1	0	0	0	0	0	0	0	0	-1.50959980423305 (0.000179601239968411)	-2.56430994686948 (0.00683502367803608)
FEZF2	0	0	0	0	0	7.03692977040407 (0.00194198839502691)	0	0	0	0
FGA	0	0	0	0	0	9.85886899073464 (1.99419122694754e-36)	0	0	0	0
FGB	4.79157998968078 (0.00247057316566285)	0	0	0	6.76050429187818 (0.0016501357384824)	14.4609515758269 (7.81918172255509e-31)	0	0	0	0
FGD2	-1.75699057092624 (0.00285626883319195)	0	-2.43171731757904 (0.00411575615638567)	0	0	0	0	0	0	0
FGD3	0	0	-2.16910419014452 (0.0013495668952568)	0	0	0	0	0	0	0
FGD5	0	0	0	0	0	0	0	-3.44670252595545 (0.00385157041482965)	0	0
FGD5P1	0	0	0	0	5.98001874788069 (3.21684608644412e-05)	0	0	0	0	0
FGD6	0	0	0	0	0	0	0	-4.34196095976849 (0.00667234574598593)	0	0
FGF10	3.31284228092369 (6.40194385663626e-06)	0	0	0	0	0	2.71331708893363 (0.000678299427384159)	0	2.95816975715895 (7.70273979536117e-06)	0
FGF12	0	0	5.16476269836186 (2.84446534740427e-10)	0	3.59360724897537 (0.00720841139149761)	3.97819124100888 (0.00295137054456514)	0	0	0	0
FGF13	2.78482706261115 (0.00104425664557563)	0	0	0	0	0	0	0	1.98402240180692 (0.00726267426442499)	0
FGF14	0	0	-2.09845588450517 (0.00809500874752397)	0	0	0	0	0	-1.99245715079922 (0.000954904697543483)	0
FGF2	1.62483532871913 (2.38767617012816e-06)	0	0	0	0	0	0	-6.24507730651042 (0.00443840935922828)	0	0
FGF21	0	0	0	0	0	6.12149777905616 (0.00694088403532365)	0	0	0	0
FGF23	0	0	0	0	0	8.38973109783814 (3.41221818813022e-06)	0	0	0	0
FGF5	5.9908114699339 (4.92077897889994e-08)	0	0	6.61749316217728 (0.000698358067673283)	0	0	0	0	0	0
FGF7	0	0	-2.24381396413629 (1.09213364480631e-05)	0	0	-1.65057609332004 (0.0091723731083658)	0	-8.93699909690833 (2.7693752911997e-05)	-1.57591518800481 (0.000180001733545847)	0
FGFBP1	0	0	0	0	0	0	4.72883433367681 (0.00136909916172326)	0	0	7.32615792143239 (0.000199345983629159)
FGFR1OP	0	0	0	0	0	2.93735383077058 (0.000700855183434003)	0	0	0	0
FGFR2	2.53086364077529 (1.521715384071e-07)	0	0	0	0	0	0	0	1.68125306131529 (0.000912257635489324)	0
FGFR3	0	0	0	0	0	2.80883942372643 (0.00893669175861205)	0	0	0	0
FGFR4	0	0	0	-6.29519031632307 (0.00831889993555471)	0	0	0	0	-4.42062854473295 (8.60838037671158e-07)	-6.57588199807927 (0.00578869315106057)
FGG	0	0	-4.47592174827994 (3.89566181131762e-06)	0	0	6.69908072853618 (5.17949541815914e-14)	0	-6.1254077173265 (0.00809026311569765)	0	-7.46318365448306 (0.00118736877234899)
FGGY	0	0	0	0	0	2.27273476676782 (0.00163761474161194)	0	0	0	0
FGL1	0	0	0	0	0	12.937373916142 (1.24367399941087e-25)	5.0155157159698 (0.00760117596103878)	0	4.1083572518162 (0.0066489006488521)	0
FGR	-3.26770364601663 (6.86469307126803e-07)	0	-2.64487232302631 (1.55476284352622e-05)	-3.02304220047555 (0.000707395910129349)	0	0	0	0	-3.33733910289938 (8.17214576334039e-09)	-2.71697597754834 (0.00142881593060852)

FHDC1	-3.51036407059857 (2.66440349468172e-05)	0	-2.30124778228304 (0.00566056357673373)	0	0	0	0	3.0875579627928 (0.000267852088715294)	2.7368166450536 (8.81265265883377e-05)	0
FHIT	0	0	0	0	2.43725395315378 (0.00684235586986362)	3.11407038278474 (8.87972450763813e-05)	0	0	0	0
FHL1	0	0	2.84518454418237 (2.9555566588831e-07)	2.09872785105173 (0.00310613182904199)	0	0	0	-2.61254612234688 (0.00376197350434706)	0	0
FHL2	2.08756032058267 (7.55273108316752e-06)	0	4.90722594788218 (5.44278007747264e-24)	0	2.60669821476358 (4.19605828037336e-05)	2.60051761187025 (0.000117496783383998)	0	0	3.20307904503273 (1.03411158380455e-13)	2.4959939042508 (0.00619268462580505)
FHOD1	-2.08979355640593 (0.00125435317680523)	0	0	0	0	0	0	0	-1.73569292365037 (0.00472430102059346)	0
FHOD3	3.36256307458004 (1.26118624459706e-09)	0	6.60837067182614 (2.50111796121021e-26)	4.34755500339154 (2.61688169812928e-05)	0	0	0	0	1.98901918556212 (0.00542646440494276)	0
FIGF	0	0	0	0	0	0	0	0	-5.23191781344608 (2.26464978992706e-06)	0
FIGNL2	0	0	0	0	6.49876283514328 (0.000473218678350271)	0	0	0	0	0
FILIP1	1.95168756899681 (4.91768676211678e-06)	0	2.82830166246297 (8.07034439433765e-10)	2.77227154604224 (0.000285153882001496)	0	0	0	0	0	0
FILIP1L	0	0	0	1.9008476446461 (0.00302985526813744)	0	0	0	-4.46753627464576 (2.98529559455987e-05)	0	0
FIRRE	0	0	0	0	0	0	0	5.05025559739464 (0.00109778743257145)	3.31433891620021 (0.00015947131651202)	0
FIS1	0	0	1.9742033226738 (1.96960170653695e-05)	0	0	0	0	0	0	0
FITM1	0	0	6.85392693390754 (7.10571376556208e-09)	0	0	0	0	0	0	0
FITM2	0	0	4.90795972455805 (3.08291088289564e-26)	0	0	0	0	0	-1.87629845497487 (0.00119631416911541)	0
FIZ1	0	0	1.75500765155728 (0.00737085781335909)	0	0	0	0	0	0	0
FKBP10	0	0	0	0	0	0	0	-7.39867173204215 (0.00080021535201245)	0	0
FKBP1A	-1.57531779917403 (0.000143231087469915)	-5.869773601716 (0.00667702970607474)	0	0	0	-2.23857809678021 (0.000435928280075169)	0	0	0	0
FKBP5	0	-2.86537006181461 (0.00270386864867051)	-1.55035032303945 (0.000303500514881745)	0	-2.20228016500895 (4.47457179765829e-05)	-2.02196632730927 (0.000448167299788125)	0	0	-2.16471577748922 (5.14372338461888e-06)	0
FLG	0	8.8208196373312 (2.777702885468e-05)	0	0	4.55965603087115 (0.00191196978665476)	4.58617288056011 (0.00458932429392392)	3.96302459845986 (0.00282071201477932)	0	0	11.1687550952607 (6.4266947003827e-08)
FLG2	0	0	0	0	5.43006642306184 (0.00314428760656518)	6.39271613983532 (0.000520071595895467)	5.10266677474972 (0.00664771799702915)	0	0	12.6149737326238 (1.13787568132945e-32)
FLJ16779	0	0	0	0	5.07235331632832 (0.00207204291455999)	6.49576226788457 (2.19462270758901e-05)	0	0	5.20364127171995 (2.81781445292595e-06)	0
FLJ21408	0	0	0	0	0	6.17516042145537 (0.0098694721805306)	0	0	0	0
FLJ35934	0	0	0	0	5.5781349099938 (0.00165490226837572)	0	0	0	0	0
FLJ38122	0	0	0	0	0	7.37338300472095 (5.35054720157315e-06)	0	0	0	0
FLJ41941	0	0	0	0	7.19458648309057 (0.000324888048259777)	0	0	0	0	0
FLJ42393	0	0	0	0	0	0	0	-6.18435149513645 (0.00491720391132454)	0	0
FLNB-AS1	0	0	0	0	0	2.71691166167798 (0.00551542732564012)	0	0	0	0
FLNC	3.43844708270987 (1.89078996782407e-10)	0	7.18843564129958 (7.12959779014157e-23)	5.24022240032271 (4.16225117698742e-09)	0	0	0	0	0	0
FLRT2	1.82257200022111 (0.000954136399961618)	0	0	0	2.47591400615552 (0.00294195994145241)	2.4852306866403 (0.00752157933628371)	0	0	0	0
FLRT3	-2.91912080807642 (0.000221854559747445)	0	-3.81639896137441 (0.000145590104883123)	0	0	0	0	0	-1.58888987412704 (0.00936486996287017)	-6.66247512652363 (0.00393329333755417)
FLT1	0	0	0	0	0	0	0	-2.19008224406346 (0.00427151307539594)	3.27552325527151 (1.0645087728462e-14)	0
FLT3	-3.39466850797834 (0.00405942799318791)	0	-3.6669433841558 (0.00427833339096442)	0	0	0	0	0	-4.24725651018339 (5.66074832789383e-05)	0
FLVCR2	-2.79353773276528 (0.00291477346135102)	0	-2.72943720580825 (0.00532352940688919)	0	0	0	0	0	0	0
FMN1	0	0	-1.78569301409416 (0.000157512226655955)	0	0	0	0	-3.28360704320104 (0.000122684582737215)	0	0
FMN2	6.69133710817724 (1.82173106856299e-09)	0	0	7.8363579463085 (9.23624078594653e-06)	0	7.35195875023175 (0.000887111044120985)	5.93979768774383 (0.00405801773300753)	0	0	0
FMNL1	-2.22270321172461 (5.70673369441633e-05)	0	-2.48752587979909 (0.00030829019104561)	0	-2.16407155701669 (0.0090397088975586)	0	0	0	0	0
FMNL2	0	0	0	0	0	0	0	-3.48710407279454 (0.000121669656442569)	0	0
FMO2	0	0	0	0	0	0	0	0	-8.59433927884311 (9.19213104396637e-18)	0
FMO3	0	0	0	0	0	2.52321141598101 (0.00129258304848849)	0	0	-4.82239193858888 (2.56692422080634e-07)	0
FMO5	0	0	0	0	0	3.70637461108806 (4.68000019994253e-12)	0	0	-1.85027405802714 (0.00533817993767238)	0
FMOD	0	0	-1.71270243902547 (0.000891838557399661)	0	0	0	0	-4.84030145388939 (0.000131651446444034)	0	0
FN1	0	0	-1.71884333846475 (0.000696158452395601)	0	-2.84930239423383 (4.44434793031899e-07)	-2.16650521191501 (0.000385481429999546)	0	-5.40538080296423 (2.44748043865462e-15)	2.76117187826955 (9.31001065216451e-08)	-3.09073398218187 (1.68575516605953e-06)
FN3K	0	0	2.32776321042562 (1.31533439742573e-05)	0	0	1.87929186140036 (0.000471358406994089)	0	2.91967191967418 (0.000663159641658354)	0	0
FNDC1	6.65812370714606 (6.66905840390479e-11)	0	0	0	4.44250291586274 (0.00605989548001883)	5.11067667816387 (0.000109602990609888)	3.47641477759008 (0.00292365579522425)	0	0	0
FNDC4	0	0	0	0	0	4.3376218460269 (4.20727840507415e-07)	0	0	0	0
FNDC5	0	0	4.27772317028044 (2.32931996100782e-07)	0	0	0	0	0	0	0
FNDC9	0	0	0	0	0	4.79793865022276 (0.0018171585692814)	0	0	0	0
FNIP1	0	0	-1.60877572031487 (2.17500661198606e-05)	0	-1.713781061105 (0.00509037554309527)	0	0	0	0	0
FOLR1	-5.36438227635153 (4.02871495623019e-05)	0	-3.61422149706577 (0.00337220972108455)	0	0	0	0	0	0	0
FOLR2	3.73043957687315 (6.01607094619626e-05)	0	0	0	0	0	2.74831254757252 (0.00633548556288557)	0	4.15156072247477 (2.18507902543184e-08)	0
FOS	-2.62540232318226 (1.56147900054567e-10)	0	-2.36187992541605 (8.80636644587425e-08)	0	-2.01389885535393 (0.000624341079226662)	0	0	-2.07947955678756 (0.00281405138517823)	2.02741288819597 (6.45606495564636e-07)	0
FOSB	-3.279376197117 (0.000276070965432001)	0	0	2.48536026666718 (0.00321367287474107)	0	0	0	0	4.67405084564086 (7.74480956619644e-19)	0
FOSL1	0	0	0	0	0	0	0	-6.01349505562967 (0.0068538243258356)	-2.157888292149 (0.00148555103266976)	0
FOXA1	-3.57612656487426 (0.00748373992110845)	4.12444387921774 (0.00209788859375711)	0	0	0	0	0	0	-4.71405616949679 (4.91663217948504e-05)	0
FOXA2	-4.77101290207501 (0.000197175819157511)	0	-3.75938525727312 (0.00225576533146269)	0	0	0	0	0	-5.54387910159909 (5.98918853966134e-07)	0
FOXA3	0	0	0	0	0	7.42959334005833 (2.14436321618591e-07)	0	0	0	0
FOXD1	0	0	0	0	7.33770294221884 (2.28200310979363e-06)	0	0	0	0	0
FOXE3	0	0	0	0	7.30573886964219 (0.000208366323090074)	0	0	0	0	0
FOXF1	1.88940248252955 (2.18891535561023e-08)	0	-5.45723428667294 (1.47488442733411e-09)	0	0	0	0	-5.95245604088212 (0.00683552937561601)	0	-4.84886985149634 (0.00318793889857695)
FOXF2	0	0	-3.06222398099971 (0.00446679388312864)	0	0	0	0	0	0	0
FOXG1	0	0	0	0	6.47559966567641 (0.0042482603748159)	6.0645547329706 (0.00760120533172173)	0	0	0	0
FOXI3	0	0	0	0	0	0	0	0	4.70924452791411 (0.00169693616138626)	0
FOXJ1	-4.78307634503035 (0.000400954685543839)	0	-4.50295741630269 (0.00105470934506857)	0	0	0	0	0	-5.89847223448988 (2.09205230260919e-06)	0
FOXK2	0	2.35491905261815 (0.00992326104526223)	0	0	0	0	0	0	0	0
FOXL2NB	0	0	0	0	0	0	0	0	4.16016450045466 (0.00702810957237294)	0
FOXM1	-3.00148557408902 (0.00202605083446813)	0	0	0	0	0	0	3.57393479541644 (0.000281296605250453)	0	0
FOXN2	0	0	0	0	-2.48110729036551 (0.0059569966811205)	0	0	0	0	0
FOXN3	0	0	0	1.80435046145348 (0.00606036347742833)	0	0	0	0	0	0
FOXN4	0	0	0	0	0	6.28293043341668 (0.00114601734092013)	0	0	0	0
FOXO1	0	0	-1.69965156209223 (1.99671531015574e-05)	0	0	0	0	-2.29856014231027 (0.00271185081039507)	0	0
FOXO3	0	-5.53037055136821 (0.00895452007893814)	0	0	0	0	0	0	0	0
FOXO4	0	0	0	0	0	0	0	0	3.38306872619728 (1.06788814782398e-13)	0
FOXP2	3.50409444672316 (2.6833202231409e-15)	0	0	3.94057371983462 (1.51504286415259e-05)	0	2.05086002210087 (0.00749844647875478)	1.60734029350473 (0.00672442145800891)	0	0	0
FP236383.10	0	0	0	0	6.74495549713948 (0.00185141427727328)	0	0	0	0	0
FP236383.9	0	0	5.04802591041132 (0.00364626777725019)	0	0	10.6440946988066 (4.72566479049138e-08)	0	0	0	0
FP325317.1	4.63602762458699	7.66282183688538	5.34132302554368	6.47913075072643	0	0	0	0	0	6.04416178468517

	(0.00126046862039067)	(0.000227147361998072)	(3.8231105982688e-05)	(0.00130342743347331)						(0.00634271091530826)
FP671120.6	0	0	0	0	0	7.41141436170492 (0.00557641536668993)	0	0	0	0
FPGS	0	0	0	-4.87531502539752 (0.00416469598286976)	0	0	0	0	-1.70797758650151 (0.00196209801030018)	0
FPR1	0	0	-2.02572857617204 (0.00887609154697146)	0	0	0	0	0	-1.61831840858461 (0.00570927728855003)	0
FPR2	-2.77480561055052 (0.000106596230239444)	0	-4.1998050832557 (3.04464921453544e-06)	0	0	0	0	0	0	-3.76328997081603 (0.00793602444002299)
FPR3	0	0	-4.78558529966342 (2.86740676316022e-06)	0	0	0	0	0	0	0
FRAS1	0	0	-3.02871371313827 (2.14953525282222e-05)	0	0	0	0	-6.78223226704076 (0.0028267275661888)	0	0
FRAT2	0	0	0	0	0	0	0	0	-1.70031586200673 (0.00309571205760691)	0
FREM2	1.77055800829353 (0.00489673602934705)	0	-3.05009355183148 (0.00240180516895886)	0	0	0	0	0	0	-6.19320920001902 (0.00786369649856033)
FREM3	0	0	0	0	0	6.994111889428 (5.01898010976961e-05)	0	0	0	0
FRMD3	1.94920654652007 (0.000313716702852566)	0	3.15101708278299 (9.09727484602968e-13)	0	0	0	0	0	2.55965464408448 (1.54620331556002e-06)	0
FRMD4B	-1.93073863771945 (3.34987172883692e-05)	0	0	-4.3758700726508 (0.000123166544218564)	0	0	0	0	0	0
FRMD6	0	3.7417290870629 (1.31668076096476e-05)	0	0	0	0	0	0	0	0
FRMPD3	0	0	0	0	6.04928751320611 (0.000548725923399553)	6.00136482948708 (0.00334271537953511)	0	0	0	0
FRRS1	0	0	0	0	0	0	1.95970362227344 (0.00330417312484403)	0	2.72854108462678 (5.60454912671616e-06)	0
FRRS1L	5.17492660725413 (1.09236945545685e-06)	0	3.56487388789639 (0.00801459679107463)	5.54683205063282 (0.00160220645017016)	0	5.73277784954169 (0.00204700152890196)	0	0	0	0
FRY	0	0	1.72393654596459 (0.000135808042711508)	0	0	0	0	0	0	0
FRZB	0	0	0	0	3.57193730579948 (0.00217669338838885)	0	0	0	3.11718834568062 (0.000308952167196149)	0
FSCN1	0	0	0	-3.80043609168821 (0.00307253654748825)	0	0	0	0	0	0
FSCN2	0	0	0	0	0	5.3095234620446 (0.00912990257818871)	0	0	0	0
FSCN3	0	0	0	0	0	7.32624266357827 (0.00538433295435045)	0	0	0	0
FSD2	0	0	5.54648583982044 (2.92331053597191e-07)	0	0	0	0	0	0	0
FSIP2	0	0	0	0	0	3.5466456947873 (0.00158263305361467)	0	0	2.81547348113964 (1.13729932289875e-06)	0
FST	0	4.29406013749569 (0.000473102175074698)	0	0	0	2.11715478188152 (0.00996325703194824)	0	0	2.48087081340784 (5.86257550991973e-08)	0
FSTL1	0	-6.12241184472502 (0.00420446555326066)	0	0	0	-1.60206263267911 (0.001706214233694)	0	-3.53198739723262 (4.81130362276264e-06)	0	0
FSTL3	0	0	0	0	0	0	0	0	2.74193229590002 (2.35234594392597e-08)	0
FSTL4	0	0	0	0	0	6.46918328010546 (3.18517804086497e-05)	0	0	0	0
FTCD	0	0	0	0	0	8.8945043084372 (2.38696052110746e-13)	0	0	0	0
FTH1P11	0	5.3173536375155 (7.90471237887881e-06)	0	0	0	0	0	0	0	0
FTH1P4	-4.42774763213378 (0.00343916839800663)	0	0	0	0	0	0	0	0	0
FTH1P7	0	0	0	0	0	0	0	0	-3.10837934475655 (0.00748576768978482)	0
FTH1P8	0	4.53529660461145 (0.000292008457930511)	0	0	0	0	0	0	0	0
FTL	0	0	0	0	0	0	0	0	-1.6738268174926 (0.000211075214388022)	0
FTLP3	0	0	0	0	0	0	0	0	-2.39337820543123 (0.000627473173678099)	0
FUCA1	0	0	-3.23199978065032 (0.0006644305065657)	0	0	0	-1.64581765306582 (0.00221768222405059)	0	0	0
FUNDC2	0	0	1.6607920284612 (0.000143355318917284)	0	0	0	0	0	0	0
FURIN	-1.65983713808111 (0.000208186367568666)	0	0	0	0	0	0	0	0	0
FUT1	-2.59528297556275 (0.00312599411142193)	0	0	0	0	0	0	0	-2.81409157652427 (0.000245377090476299)	0
FUT2	0	0	0	0	3.6756260853383 (0.00844844099672066)	0	0	0	0	0
FUT4	0	0	0	0	0	0	0	3.55603497564374 (0.00016830997582609)	0	0
FUT6	0	0	0	0	0	6.25148533883376 (7.43222711171709e-05)	0	0	0	0
FUT8	0	0	0	0	0	0	0	0	2.09672716506285 (7.60502423505533e-10)	0
FUT9	0	0	0	0	6.0235020645757 (0.00102437077642912)	6.08022013478627 (0.00982730814293662)	0	0	4.02652027065442 (0.00129624181363916)	0
FXR2	0	0	2.33930530959047 (5.69169648086903e-06)	0	0	0	0	0	0	0
FXYD1	3.9138386065805 (0.000274601120875316)	0	4.11705883663616 (3.43896449304669e-05)	0	0	3.74033381375349 (0.00857908888234497)	0	0	0	0
FXYD2	0	0	0	0	7.62549393061528 (5.64089909291248e-14)	5.44953870938191 (9.12253968838557e-06)	0	0	0	0
FXYD3	0	0	-3.85068953888005 (0.00199676374840519)	0	0	0	0	0	0	0
FXYD6	3.30861997759053 (8.66456081471552e-11)	0	0	3.91326357322689 (1.24209255103564e-06)	0	0	0	0	0	0
FYB	-1.92950470948118 (0.000394759982017134)	0	-2.92049963800373 (2.52875068022037e-06)	-2.54928291999583 (0.0036460132872432)	0	0	0	0	0	0
FYCO1	0	0	1.90358089900638 (0.000233589798843741)	0	0	0	0	0	0	0
FYTTD1P1	0	0	0	0	0	8.5962897837694 (1.04811417830601e-05)	0	0	0	0
FZD10-AS1	0	0	0	0	4.41827504548026 (0.000505241888860792)	4.84322191511132 (0.000554041155037714)	0	0	0	0
FZD2	0	0	0	0	0	5.40205618139091 (0.000782466383899239)	0	0	0	0
FZD3	2.54544558352474 (3.6392793140283e-07)	0	0	0	0	0	0	0	0	0
FZD4	0	0	0	0	0	0	0	-4.34046998340116 (0.0017124868044113)	-1.74207724109697 (0.00356575858604876)	0
FZD5	0	0	-2.38418297024959 (0.000263994831785669)	0	0	0	0	0	0	0
FZD6	0	0	0	0	0	-2.99886011877386 (0.0030441195033738)	0	0	0	0
FZD7	2.75036823333476 (1.09479085686715e-07)	0	0	2.45662356295385 (0.00625833299757455)	0	0	0	0	0	0
G0S2	0	0	0	-2.07737408256414 (0.00491016868355828)	0	0	0	-2.79957201126416 (0.00056198797030725)	-4.00269590090499 (2.04041656675089e-21)	0
G3BP1	0	0	0	0	-1.62837836866272 (0.00148176303881993)	0	0	0	0	0
G6PC	0	0	0	0	0	8.22203531811589 (3.06439029892931e-13)	0	0	0	0
G6PD	-1.85029944615314 (0.00041177058431805)	0	0	0	0	0	0	0	-2.17680787440581 (0.000145593479313927)	0
GAB2	0	0	0	0	0	-1.50707126716279 (0.00504117058696879)	0	0	0	0
GABARAPL3	0	0	0	0	0	7.10473021854048 (0.000174531145058712)	0	0	0	0
GABBR2	-5.06974610938087 (0.000555040941343912)	0	0	0	0	0	0	0	-3.54849873315202 (0.00105809177240524)	0
GABRA2	0	0	0	0	7.66922601221081 (3.61983527916219e-08)	6.37929666870103 (0.000110225779487594)	5.11427764599259 (0.00456261883039521)	0	0	0
GABRA3	0	0	0	0	0	0	0	0	3.72152392088988 (0.00787485917339572)	0
GABRA4	0	0	6.64912692554275 (4.52238726937584e-07)	0	6.91800884378814 (0.00837990899630342)	8.19107707685944 (0.000998425729837212)	0	0	4.74811835917648 (0.00109872802926405)	0
GABRB3	0	0	0	0	0	3.48622593256496 (0.00888548434691583)	0	0	0	0
GABRE	0	0	0	0	0	0	0	0	4.37041462809692 (4.85453541141676e-11)	0
GABRG2	0	0	0	0	0	7.20898483477395 (0.00651838541340656)	0	0	0	0
GABRG3	0	0	0	0	6.24434293510046 (0.0075784156376657)	7.11254271341322 (0.00756733940646282)	0	0	0	0
GABRP	0	0	0	0	0	0	0	0	4.91600569958799 (0.00149382183532502)	0
GADD45A	-2.82932672403732 (1.16480603058299e-06)	0	0	0	0	0	-1.95379056383417 (1.2912495419067e-05)	0	-2.10730587784425 (0.000139656045478061)	0
GADD45G	0	0	2.5252486098747 (0.000886408322870341)	0	0	0	0	0	2.77125361673545 (0.000319749280735339)	0
GADL1	0	0	0	0	0	6.06308937190089 (0.00228721263907649)	0	0	0	0

GALNT10	0	0	0	0	0	0	0	0	-1.69897526048171 (6.82765989909603e-06)	0
GALNT13	0	0	0	0	0	4.2743963990727 (0.00626161187053872)	0	0	0	0
GALNT15	1.95639265281659 (0.00123180750432835)	0	0	2.78241219199854 (0.00362104404631781)	0	0	0	0	1.65831995513405 (0.00660914075816287)	3.60039700836628 (0.000113384282923186)
GALNT16	1.66172279989994 (0.00494709449179009)	0	3.86826988556248 (8.52250574697018e-13)	3.10028637946596 (0.00642652080766468)	0	3.30552492627517 (0.0030074786573332)	0	0	0	0
GALNT18	-2.01716448752898 (0.00225023973388934)	0	0	0	0	0	0	0	0	0
GALNT3	0	0	-3.99793043027539 (0.000533477775950991)	0	0	0	0	0	0	0
GALNT5	-4.71086273760442 (1.38684102121971e-05)	0	-5.12767495314898 (3.03193710178598e-06)	-6.67890881176445 (0.00411126662060755)	0	0	0	0	0	0
GALNT6	0	0	0	0	0	0	0	2.87501223631235 (0.00528819730044514)	2.92420475249673 (5.4628548429724e-08)	0
GALNT8	0	0	0	0	5.7800642702998 (0.00591384444719865)	7.0048238026879 (0.00773068985945733)	5.24904568531012 (0.00288258757055086)	0	0	0
GALNT9	0	0	0	0	5.79712044871431 (0.000960887504490318)	0	0	0	0	0
GALR1	0	0	0	6.06145843556709 (0.00632704649183289)	0	7.24648676636329 (6.79124032548276e-05)	0	0	0	0
GALR2	6.26465858730613 (1.20250436514697e-06)	0	0	0	0	0	0	0	0	0
GALT	0	0	0	0	0	2.29612034638648 (0.00585799666025345)	0	0	0	0
GAMT	0	0	4.27443824525581 (1.32311716114767e-05)	0	0	4.81588549048366 (0.00045389009951584)	0	0	0	0
GAP43	6.98575275892689 (9.72547390237217e-09)	0	0	6.65577167061567 (0.00503524753410479)	0	0	0	0	0	0
GAPDHP1	0	7.03517784057268 (2.7711688808048e-07)	0	0	0	0	0	0	0	0
GAPDHP33	0	0	0	0	0	0	0	0	3.92384345501154 (0.00883233488569827)	0
GAPDHP59	-3.54121984619126 (0.00845395971032747)	0	0	0	0	0	0	0	-4.22448813535379 (0.000404531389730331)	0
GAS1	0	0	0	0	0	0	0	0	4.24784805373702 (1.20405669279222e-07)	0
GAS7	0	0	0	0	-1.64203450427891 (0.00400581669475617)	0	0	0	0	0
GATA1	0	0	0	0	0	0	0	5.67862869449164 (0.000734680026579306)	0	0
GATA2	-2.28018642703022 (0.000405598014167222)	0	0	0	0	0	0	0	1.58333356079527 (0.00116494473638208)	0
GATA3	0	0	0	0	0	0	0	0	5.40617968107024 (7.65444466770524e-15)	0
GATA4	0	0	9.1380135892512 (5.59535761349118e-20)	0	0	7.21698339867309 (9.34294876354154e-07)	0	0	5.43260137047733 (0.000103035980113174)	0
GATA6	-2.19485576863155 (0.00930647050149408)	0	2.29635657087578 (0.000655329710463066)	0	0	0	0	0	0	0
GATB	0	0	2.86331064594609 (3.69204788304177e-06)	0	0	3.40203432957285 (0.000178744290370445)	0	0	0	0
GATM	2.99181979896979 (0.00039272449056844)	0	2.9523162718094 (0.00117662476741247)	0	0	6.18171294822595 (1.99711897072416e-10)	0	0	4.22647108453138 (5.97041333439534e-11)	0
GATSL2	0	0	3.45831359919032 (0.000139561204024492)	0	0	0	0	0	0	0
GBA3	0	0	0	0	0	6.08553211647466 (0.00672465291330808)	0	0	0	0
GBP1	0	0	0	0	0	0	0	-2.66343365694934 (0.00905744128476486)	0	0
GBP1P1	0	0	0	0	0	0	2.78651101847777 (0.00299423168991983)	0	0	0
GBP3	0	0	0	0	0	-1.99849885738026 (0.00427499145043965)	0	-7.5339006728866 (0.000588903830275428)	0	0
GBP4	0	0	0	0	0	0	0	-7.71636645428504 (0.000688789170552731)	0	-2.91043392366997 (0.00581073263946857)
GBP5	-2.67002151100842 (0.0019239279828747)	0	0	-4.13547597623337 (0.00610911156266007)	0	0	0	0	0	0
GBP7	0	0	0	0	0	4.47928307430153 (0.00516797761707182)	0	0	0	0
GBX1	0	0	0	0	7.19458648309057 (0.000324888048259777)	7.24070370783021 (0.00594801261388227)	0	0	0	0
GC	0	0	0	0	0	12.4486024628826 (1.43736584181745e-21)	0	0	0	0
GCA	0	0	0	0	0	0	0	2.80628625605139 (0.000779214798780129)	0	0
GCDH	0	0	0	0	0	3.27469225832532 (1.49306266825242e-06)	0	0	0	0
GCGR	0	0	0	0	0	6.27482296174374 (0.004209680465281)	0	0	0	0
GCH1	-2.73086348438175 (0.000577926250282108)	0	-3.70735531396483 (8.5092334292198e-05)	0	0	0	0	0	0	0
GCK	0	0	0	0	0	5.67923335443026 (0.00460943781333014)	0	0	0	0
GCKR	0	0	0	0	0	7.59126893563462 (7.53535656047427e-10)	0	0	0	0
GCM1	0	0	0	0	0	0	0	0	8.08502777259157 (9.56020604953081e-23)	0
GCNT3	0	0	0	0	0	4.03457633732405 (0.00376586168355883)	0	0	0	0
GCNT4	0	0	0	0	0	0	0	0	2.34294942360636 (0.000346354395053455)	0
GDA	0	0	0	0	0	6.91535618189957 (3.6818509377515e-10)	0	0	5.24738533260986 (5.88482502864556e-07)	0
GDAP2	0	0	0	0	-1.86571357496632 (0.00676970294473828)	0	0	0	0	0
GDF10	0	0	0	0	0	0	0	0	-4.07241085715075 (0.00125873409009671)	0
GDF15	-4.27148211512676 (3.21638999345856e-08)	0	-4.39635657626286 (2.53025393469205e-06)	-4.05303606463815 (0.000481081330789891)	0	0	-2.43445370901044 (0.000363618780943595)	0	0	-3.48812546860162 (0.00123782325991262)
GDF6	0	0	0	0	4.59609050529168 (0.00880095582292758)	6.18029103669083 (0.00894791953101118)	0	0	4.6915900497755 (2.84153407135234e-05)	0
GDF7	0	0	0	0	4.38290830398885 (0.000469022298618657)	5.0926708467457 (0.000295618996620876)	0	0	0	0
GDNF	5.04069393271629 (4.86433130378628e-05)	0	0	0	0	5.20522850772392 (0.00974597258667192)	0	0	4.19805779346179 (0.000417373006802554)	0
GDNF-AS1	0	0	0	0	0	7.76345302701345 (3.48803208586904e-05)	0	0	4.09413751193915 (0.00205688453968633)	0
GDPD5	0	0	0	0	0	0	0	0	2.91603957858923 (0.00536653472528028)	0
GDPGP1	0	0	0	0	0	3.96660212408777 (0.00104711236349797)	0	0	0	0
GEM	1.81540107224322 (3.42784720281991e-06)	0	0	1.91252093363133 (0.00747128047666881)	0	0	0	0	-1.6995577647979 (0.0025132482191789)	0
GEMIN6	1.69251331711539 (0.00218589602653157)	0	0	0	3.15498704672825 (4.54236779942654e-05)	0	0	0	0	0
GFI1	0	0	0	0	0	0	0	5.16909730569978 (0.000127470183281545)	0	0
GFI1B	0	0	0	0	0	0	0	5.65241709824842 (7.89539746868551e-05)	0	0
GFOD1	0	0	2.30622983177865 (7.46107805800756e-10)	0	0	0	0	0	0	0
GFOD2	0	0	0	0	0	2.44656567795509 (0.000312407745779869)	0	0	0	0
GFPT1	0	-5.66518641187412 (0.00778776478531148)	0	0	-1.80179616413961 (0.00171125709106714)	0	0	0	0	0
GFPT2	0	0	0	0	0	0	0	-6.07444617523737 (0.00732339805810031)	0	0
GFRA1	4.83171903611446 (3.00565862168248e-14)	0	0	4.9945476852641 (1.71929800058317e-06)	0	2.7414318235207 (0.00880004983997644)	0	0	-3.74854854078878 (0.000923964095565305)	0
GFRA2	0	0	0	0	0	0	0	0	2.60495147110655 (0.0072283368689988)	0
GGH	0	0	0	0	0	3.45771986015525 (0.00573373480900835)	0	0	0	0
GGT6	0	0	0	0	0	4.88358497443108 (0.00838878529525244)	3.61473108633682 (0.00526648075930378)	0	0	4.98019948660435 (0.00985438653562942)
GGTA1P	0	0	0	0	0	0	0	0	2.520486955639 (0.000503179208279599)	0
GH2	0	0	0	0	0	0	0	0	8.9729531814549 (2.91211659537107e-06)	0
GHR	2.15482089861405 (0.000145664450775611)	0	0	0	0	3.22359342681248 (0.0013818928304649)	0	0	0	2.72277411286344 (0.00861920054961243)
GID4	0	0	0	2.40392109755928 (0.0056666513747019)	0	0	0	0	0	0
GIMAP5	-2.01155735741879 (0.000254697748966047)	0	0	0	0	0	0	0	-1.56129608458614 (0.00227986227113784)	0
GIMAP8	0	0	0	0	0	-2.04007272214917 (0.00493512354594021)	0	0	0	0
GINM1	0	0	0	0	0	0	0	-2.88799006665719 (0.00901898406895403)	0	0
GINS1	0	0	0	0	0	0	0	5.15594867337146	0	0

								(0.00175504619077815)		
GINS3	0	0	0	0	0	0	3.24244392350697 (0.00871844282336705)	0	0	0
GIPC2	0	0	0	0	0	2.86587313263194 (0.00193245742341147)	0	0	0	0
GIPC3	0	3.63293504769293 (0.00628770719884589)	0	3.25237811940176 (0.0023527336290474)	0	0	0	0	0	0
GJA1	0	0	0	0	0	-2.07606756174351 (0.00155931476788379)	0	-4.63073512993468 (0.000195868795019804)	2.31590233241638 (2.35470370337744e-11)	1.82237626447244 (0.0072982221055451)
GJA3	0	0	5.7470556387684 (2.28506833700447e-05)	0	0	0	0	0	4.55306771069369 (0.00314826332686638)	0
GJA4	0	0	1.72369431879582 (0.00211673702576978)	0	0	0	0	0	0	0
GJA5	0	0	0	0	0	0	0	-6.18862716528598 (0.00659008669314628)	1.69328242632778 (5.66071224004328e-05)	-4.08424214883831 (0.00380794061337867)
GJB1	0	0	0	0	0	8.40876673754004 (1.30600060139223e-08)	0	0	0	0
GJB2	0	0	0	0	0	0	0	0	0	7.0160023925954 (0.00512922053037508)
GJB4	0	0	0	0	0	5.56597899983282 (0.00717847242133961)	0	0	0	0
GJB6	0	0	0	0	0	0	0	0	0	6.57804517678195 (0.000746627810432485)
GJB7	0	0	0	0	6.11833150746452 (0.00167768307910175)	0	0	0	4.08431536165085 (0.000781742761761153)	0
GK	-2.48553608952502 (6.98012470203341e-06)	0	-2.98762131151182 (8.08021172719084e-06)	-5.50683602536622 (0.00087853128634622)	-3.43621607690448 (0.00221287063189378)	0	0	0	-3.05810497052333 (1.62238987609193e-08)	-3.20163032509491 (0.00137183627619629)
GK-AS1	-4.48862585245113 (0.0019575778425544)	0	0	0	0	0	0	0	-3.60828530576866 (0.00302342354877226)	0
GKAP1	0	3.59262829009173 (0.00688358581191683)	0	0	0	0	0	0	0	0
GKN1	0	0	0	0	0	0	0	0	5.76628858286057 (0.00373902424708277)	0
GLB1L2	0	0	0	0	4.40581994063596 (6.68041304340084e-05)	0	0	0	0	0
GLCCI1	0	0	0	0	0	0	0	0	1.68553519816776 (2.46082489741696e-05)	0
GLCE	0	2.86236733119938 (0.00618573526941427)	0	0	0	0	0	0	0	0
GLDC	0	0	0	0	4.28988053159382 (0.00789521997476939)	6.03982659456388 (4.13557090729405e-10)	0	0	6.11330414692253 (1.22197374429997e-17)	0
GLDCP1	0	0	0	0	0	7.35517093416065 (0.000647654880123448)	0	0	0	0
GLDN	-5.2577019637032 (2.29568852472043e-12)	0	-3.78578809759243 (1.05163401740353e-08)	-5.50999720445348 (6.59757328502774e-05)	-2.56700312019313 (0.00937123434523983)	-3.63073950338816 (0.000183652858732279)	0	0	0	0
GLE1	0	3.30080025078126 (8.34342498130274e-05)	0	0	0	0	0	0	0	0
GLI1	0	0	0	0	0	0	3.98045316817581 (0.00332681871854443)	0	0	0
GLI3	1.53348770815212 (0.000126782754443425)	0	0	0	0	0	0	0	1.69404669634035 (2.32117991307986e-05)	0
GLIDR	0	0	0	0	5.38072472064522 (0.000788055274500848)	0	0	0	0	0
GLIPR1	0	0	-2.67803821606666 (0.000278324440360196)	0	0	0	0	0	0	0
GLIPR2	0	0	0	0	0	0	0	0	-1.66307281586821 (0.00147107348190977)	0
GLIS2	-1.69145775615056 (0.0026829322352573)	0	0	0	0	0	0	0	0	0
GLIS3	0	0	-1.67056465609115 (0.00572090507405731)	0	0	0	0	0	0	0
GLP1R	0	0	0	0	0	5.59858946563144 (0.0081844550952342)	0	0	0	0
GLP2R	6.60661737140018 (5.08465456058392e-08)	0	0	0	0	6.68434365713143 (0.00367258187758186)	5.47109521540374 (0.00107553781700549)	0	0	0
GLRA3	0	0	0	0	0	5.79583692437278 (0.00343387599602771)	0	0	0	0
GLRB	4.64098373090518 (9.91474994438215e-05)	0	4.12610175671102 (0.000848141824102696)	0	0	0	0	0	0	0
GLRX	-1.61989501659423 (0.0021141763368599)	0	-1.77223106799877 (0.000118083747030306)	-2.30590634487818 (0.00323743873647851)	0	0	0	0	0	0
GLRX5	0	0	1.66910672303075 (0.00131473269045639)	0	0	0	0	2.39765544573316 (0.00847834767354018)	0	0
GLS	0	0	0	0	0	-1.59977141613777 (0.000626431316445407)	0	0	0	0
GLS2	0	0	0	0	0	5.28614826739001 (0.000331063871304447)	0	0	0	0
GLT1D1	-4.63102685855634 (0.000448167421947733)	0	0	0	0	2.72286494036014 (0.000373483868482581)	0	0	-2.86182377919322 (0.00349872758088799)	0
GLUD1	0	0	0	0	0	0	0	-2.46098917601825 (0.0041501006470688)	0	0
GLUD2	0	0	0	0	0	3.59427892055119 (0.00349494520479529)	0	0	0	0
GLUL	0	-3.34469175371442 (0.000166940665291431)	-2.33416263979573 (1.52293165336054e-10)	0	-2.43573442961285 (0.000469066874182967)	0	0	0	0	0
GLYAT	0	0	0	0	0	5.61603037104059 (0.00505121393350551)	0	0	0	0
GLYATL1	0	0	0	0	0	7.98966052036661 (7.70901119168241e-09)	0	0	0	0
GLYCTK	0	0	0	0	0	3.93378943624454 (2.85049081450387e-07)	0	0	0	0
GM2A	0	0	-1.56947875079887 (0.00475503836202084)	0	0	0	0	0	0	0
GMDS-AS1	0	0	0	0	0	2.1788813028463 (0.0045246622986405)	0	0	0	0
GMFB	0	0	0	0	0	0	0	-3.66684958534343 (0.00634596697760304)	0	0
GMIP	-2.12713822155107 (0.000138848640734393)	0	-2.71857882749041 (6.18396588889761e-05)	0	0	0	0	0	0	0
GMNC	0	0	0	0	0	5.8320435271638 (0.00212439276894611)	0	0	0	0
GMPR	0	0	2.95851322430078 (5.1239317503297e-07)	0	0	0	0	3.66464604001732 (0.000408843349010278)	0	0
GNA13	0	0	-1.67995073310225 (0.000205963143075281)	0	0	0	0	0	0	0
GNA14	0	0	0	-6.02741664473902 (0.00881802252919697)	0	0	0	0	0	0
GNAL	2.20671432907443 (0.000503036267426335)	0	0	0	0	2.51478272960194 (0.00754818599009889)	0	0	0	0
GNAO1	5.47413895273667 (5.0605728708881e-20)	0	3.2020927461926 (5.23013503381089e-05)	4.98966790601454 (7.97489809933829e-06)	4.11820625193607 (3.51028162695469e-05)	4.53404752749663 (5.36482738894534e-06)	0	0	0	0
GNAZ	4.03989141737011 (1.8791460301448e-09)	0	0	0	0	0	0	0	0	0
GNB5	0	0	0	0	0	2.65060984972017 (0.000117399381338247)	0	0	0	0
GNE	0	0	0	0	0	1.96737118478098 (3.96225767178995e-05)	0	0	0	0
GNG12	0	0	0	0	0	0	0	-4.8754477934791 (7.43392367696336e-05)	0	0
GNG2	2.06021477545152 (2.00318335788031e-05)	0	0	0	0	0	0	0	0	0
GNG4	3.88365271175494 (0.00211417214094175)	0	0	0	0	5.14921859606818 (0.00182215910994476)	0	0	0	0
GNG7	0	0	4.78295990130918 (9.82820238101151e-12)	0	0	3.43305230088693 (0.000995671050165625)	0	0	0	0
GNGT1	0	0	0	0	0	7.18609492789082 (0.00581362640846632)	0	0	0	0
GNLY	-3.76614900441319 (6.56339759076734e-05)	0	-3.43154582430303 (0.00133855430450038)	0	0	0	0	0	0	0
GNPATP	0	0	0	0	0	7.28410256386346 (0.00123813729563598)	0	0	0	0
GNPDA1	0	0	-1.82091088871798 (0.00366810539517103)	0	0	0	0	0	0	0
GOLGA2P5	0	0	-3.16647110516615 (0.00624596703890042)	0	0	0	0	0	0	0
GOLGA4	0	-2.42146853589165 (0.00828005052672247)	0	0	0	0	0	0	0	0
GOLGA6L2	0	0	0	0	8.97552714929857 (0.00967184207860819)	7.76451638699445 (0.00268536568872558)	0	0	0	0
GOLGA6L4	0	5.20171394852305 (0.0025443278926232)	0	0	0	0	0	0	0	0
GOLGA6L7P	4.85064435237176 (0.002121425668271)	0	0	0	0	0	0	0	0	0
GOLGA8A	0	0	-1.85815412045274 (0.00340270629594757)	0	0	0	0	0	0	0
GOLGB1	0	-3.33354423050194 (0.00397506046083095)	0	0	0	0	0	0	0	0
GOT1	0	0	3.69915485271464 (1.92673603295467e-16)	0	0	4.02601213695447 (1.81178486315266e-06)	0	0	0	0
GOT2	0	0	2.33649181080843 (1.02478484084164e-08)	0	0	0	0	0	0	0

GP2	0	0	0	0	7.35579798179143 (0.00270092242411477)	0	0	0	0	0
GP6	-4.64082468727667 (0.00236894700643551)	0	0	0	0	0	0	0	0	0
GPAT2	0	0	3.17927155662108 (0.000948097044754552)	0	0	0	0	0	0	0
GPATCH4	0	0	0	0	0	0	0	0	-1.70592753540526 (2.87762466343918e-06)	0
GPBAR1	3.58570092987147 (0.00886662297384162)	0	0	0	0	0	0	0	0	0
GPC1	0	0	2.65716508644692 (1.88416364720967e-05)	0	0	0	0	0	0	0
GPC2	0	0	0	0	0	5.09755009143568 (0.00504480398928871)	0	0	0	0
GPC3	-1.50600496580981 (0.00191421576283754)	0	-2.33950020641698 (1.19126261741499e-05)	-2.10299568326217 (0.00579084480516944)	0	0	0	-7.18692949581912 (0.000829028111106813)	2.44455881989364 (2.77250924738277e-06)	0
GPC4	0	0	-2.18024370028412 (0.000422862125550516)	0	0	0	0	-6.95111675894885 (0.00150988417640071)	0	0
GPC6	0	0	-2.11136469009808 (0.00174222274553482)	0	0	0	0	0	0	0
GPCPD1	-1.75396246725259 (0.00148600728971612)	0	-2.53403220858455 (5.07845445274469e-07)	0	-2.09131901866623 (0.00584288031977765)	0	0	0	0	0
GPD1	0	0	2.79858611717892 (0.00308462629493879)	0	0	4.27957483837085 (4.41629929225329e-05)	0	0	0	5.54851668143867 (8.71593267322699e-07)
GPD1L	0	0	2.20884985951032 (2.54130927695395e-06)	0	0	0	0	0	0	0
GPD2	0	0	0	0	0	-1.70915386643507 (0.00705496468212565)	0	0	0	0
GPI	0	0	0	0	0	0	0	1.94238772616795 (0.0016393811205958)	0	0
GPLD1	0	0	0	0	0	4.71877385066567 (3.62467778805597e-05)	0	0	0	0
GPM6A	3.7524491849788 (6.11389059616025e-06)	0	0	0	5.15902990245087 (0.000109890362174287)	3.74882920545505 (0.00118164178033002)	3.2146709761345 (0.000478204110360805)	0	0	0
GPM6B	0	0	-2.86817495017747 (0.000621809952356943)	0	0	0	0	0	-1.55007575671973 (0.00232717049650863)	0
GPNMB	0	0	0	0	0	-2.81280979762078 (0.0028276958944491)	0	0	0	0
GPR1	0	0	0	0	0	0	0	0	4.83335890317821 (5.92230461415491e-05)	0
GPR1-AS	0	0	0	0	0	0	0	0	6.46450315502726 (2.17234532457196e-07)	0
GPR110	-6.49382812731616 (1.27325613408306e-06)	0	-4.76263326020876 (8.98793663695542e-05)	-6.3184197785938 (0.00885439450049537)	0	0	0	0	-2.36988545001164 (0.00388177868435351)	-6.59844824470858 (0.00621823076741424)
GPR111	0	0	0	0	0	6.61489342504846 (0.000500265812804008)	0	0	0	0
GPR112	0	0	0	0	0	0	5.39860545720225 (0.00333986749172449)	0	0	0
GPR115	0	0	0	0	0	0	0	0	6.09240968334656 (1.23709986614387e-08)	0
GPR116	-2.91441247840099 (1.05138631390719e-17)	-4.37637385160271 (0.00497591733854564)	0	-3.33103064191664 (2.23174511735538e-07)	-2.30694742939015 (1.47944616425937e-05)	-3.49882668559651 (2.32947198087208e-08)	0	-3.43421198073834 (8.30771869345009e-07)	-1.83888987672114 (0.00281131291771882)	-2.89930721140098 (4.42818193927313e-06)
GPR124	0	0	0	0	0	0	0	0	1.60069436114721 (6.21034694133906e-05)	0
GPR125	1.83922237892303 (0.000759373022431739)	0	0	0	0	0	0	0	0	0
GPR126	-1.65060634354848 (0.000200345578838288)	0	-3.04963392512444 (4.49328142628572e-05)	0	-2.60456895662368 (0.00200011170352569)	0	0	-7.52591042335413 (0.000471219758138877)	1.60667086119608 (0.00335442128778138)	0
GPR133	0	0	0	0	0	0	0	-4.75422091085129 (0.0041323431253842)	0	0
GPR143	0	0	0	0	0	0	0	0	3.91497496215376 (0.00533223724219677)	0
GPR144	0	0	0	0	6.80050039693727 (0.00176612853259011)	7.87361530700542 (0.00172085486164274)	0	0	0	0
GPR155	2.39195868281571 (7.2929415773545e-05)	0	0	0	0	0	0	0	0	0
GPR156	0	0	0	0	0	6.11700665307663 (0.000163030641283685)	0	0	0	0
GPR158	0	0	0	0	0	0	0	0	4.59000695930712 (4.39835071914175e-05)	0
GPR161	1.69147634660427 (0.00377792870048546)	0	0	0	0	0	0	0	0	0
GPR162	0	0	0	0	0	0	0	0	-2.51017131147094 (0.00787608859922065)	0
GPR176	0	0	-2.30221029555237 (0.00444927049139141)	0	0	0	0	0	0	0
GPR179	0	0	0	0	0	4.73223881280355 (0.00857717563136131)	0	0	0	0
GPR183	0	0	-3.26924062665031 (0.000475146895406338)	0	0	0	0	0	0	0
GPR26	0	0	0	0	0	6.31228457415244 (0.00146023147499294)	0	0	0	0
GPR27	0	0	0	0	0	0	4.1527767767686 (0.00457996131726045)	0	0	0
GPR34	2.15690807168558 (0.00349123745437447)	0	0	0	0	0	0	0	3.04799945965406 (6.7987492054927e-10)	0
GPR37	0	0	0	0	0	4.6118406230637 (0.00533741285863963)	0	0	3.70087992005989 (0.00108653084035348)	0
GPR37L1	0	0	0	0	0	5.87259701218333 (0.00109858814981401)	0	0	0	0
GPR39	0	0	0	0	0	0	2.55795200573799 (0.00858439884634582)	0	0	0
GPR4	0	2.33949551423106 (0.00207587825519684)	0	0	0	0	0	-7.25701294635322 (0.000781352571582191)	-3.47746316898758 (6.59952395090995e-14)	0
GPR55	0	0	0	0	0	0	4.78653440031918 (0.0086205333615241)	0	0	0
GPR56	-1.86253277956899 (0.000309120078754252)	0	0	0	0	0	0	-3.17500979067089 (0.00437181443323287)	0	0
GPR61	0	0	0	0	0	5.58064766943314 (0.00177567597634684)	0	0	0	0
GPR65	0	0	-3.24884720742755 (0.00712080863683494)	0	0	0	0	0	0	0
GPR75	0	0	0	0	0	5.09719736251247 (0.000820366869842759)	0	0	0	0
GPR78	0	0	0	0	0	5.84849491006459 (0.00248878374318437)	0	0	7.29834526074947 (1.44358756866691e-14)	0
GPR83	0	0	0	0	0	6.61769545560163 (0.00266817113552489)	0	0	0	0
GPR85	0	0	0	0	0	5.88627607926316 (0.000292702606054476)	0	0	0	0
GPR87	0	0	0	0	0	0	0	0	5.92372494475111 (8.13317429613355e-08)	0
GPR97	-2.39430348876735 (0.00199584735287671)	0	-2.63058903688214 (0.00749394513669001)	0	0	0	0	0	-2.8387695725581 (3.98318496396815e-05)	0
GPR98	-3.58511647760445 (5.19133868121114e-07)	0	-4.65157657935953 (4.00379255789049e-07)	-4.62924432692725 (0.00621708111595624)	0	0	0	-6.01239902020223 (0.00721995918621132)	-3.63558250388593 (4.23068229441891e-09)	-4.9112923546393 (0.00368776990321339)
GPRASP1	2.50110800172575 (6.25405524699148e-05)	0	0	3.04995315119202 (0.00604473375448786)	0	0	0	0	0	0
GPRC5A	-4.04114866548746 (1.69155676809505e-11)	0	-4.64964708823198 (1.53154831868424e-12)	0	-2.10225285616876 (0.00122637904934842)	-3.03924447713186 (0.000908630749044941)	0	-4.87787029330872 (8.63065628538046e-07)	0	-2.78373370739774 (0.000274607334130912)
GPRC5B	0	0	2.88164868391021 (2.34141968219286e-05)	0	0	0	0	0	0	0
GPRC5C	0	0	0	0	0	2.94919085637022 (3.91179927213252e-05)	0	0	-2.4958106973985 (0.00199560343224047)	0
GPRIN1	0	0	0	0	0	4.51575134842339 (0.00997682721787544)	0	0	0	0
GPRIN3	-1.7107863552262 (0.00446239865533446)	0	0	0	0	0	0	0	0	0
GPSM1	0	0	0	0	0	0	0	0	-2.54335118884913 (7.32947340462733e-05)	0
GPSM2	0	0	-2.63239448197083 (1.12960330430644e-05)	0	0	0	0	0	0	0
GPSM3	-1.95746876902224 (0.00771625547717005)	0	0	0	0	0	0	0	0	0
GPT	0	0	3.77684448192933 (0.000203290368485497)	0	0	6.05131440961285 (3.04644041436854e-07)	0	0	0	0
GPT2	0	0	0	0	0	3.46412448120687 (3.83772156973137e-07)	0	0	0	2.83544140626859 (0.00283701538341778)
GPX2	0	0	0	0	0	4.77377185795284 (4.5040931608873e-12)	0	0	0	0
GPX3	0	-2.24287646112671 (0.00130343513381384)	0	0	0	0	0	-4.59641946976385 (1.14608096646359e-11)	-1.85003332518858 (6.45132353523656e-12)	0
GPX5	0	0	0	0	0	6.8747978295475 (0.00948482808621846)	0	0	0	0
GPX8	0	0	0	0	0	0	0	0	1.69638089627667 (0.00042023478614484)	0
GRAMD1B	0	0	2.66957651177054 (8.67923566332444e-06)	0	0	2.85408958218635 (0.00399211239152959)	0	3.02412143780143 (0.00590336962909579)	2.23687837777623 (0.000142132073678505)	0
GRAMD1C	-3.40008054207783 (0.00788995017671394)	0	0	0	0	0	0	0	0	0
GRAMD2	0	0	0	0	0	0	0	0	3.10353884295161	0

									(6.14848875904192e-08)	
GRAMD3	0	0	0	0	0	0	0	0	2.25313086273114 (4.70606223098187e-08)	0
GRAMD4	0	0	1.74594594122082 (4.13256515203037e-05)	0	0	3.07821511591908 (2.63414085862476e-06)	0	0	0	0
GRASP	-1.93263031415898 (0.000114098487396745)	0	0	0	0	0	0	0	-1.78709491563582 (4.49949777405961e-05)	0
GRB14	0	0	0	0	0	5.41674558962202 (1.39278447399819e-05)	0	0	3.58716650358844 (0.00447452323991949)	0
GRB7	0	0	0	0	5.01663851638278 (0.000137987998892885)	0	0	0	2.8990932254581 (0.000506147533212706)	0
GREB1	0	0	0	0	0	0	0	0	7.26058444852066 (1.16962088533153e-06)	0
GREB1L	0	0	2.3847681035872 (0.00874900687961047)	0	0	0	0	0	4.69123385341392 (4.33757273901411e-09)	0
GREM1	3.94102652744021 (0.000661074034283789)	0	0	0	0	0	0	0	-4.31305345743817 (5.87799092376577e-05)	0
GREM2	6.60714980568593 (7.83192470363312e-20)	0	0	6.09799664035091 (6.61103971482218e-07)	0	5.67320988174429 (1.3930779141745e-09)	0	0	3.09318967844908 (0.00431126196411167)	0
GRHL1	0	0	0	0	0	2.37184603315664 (0.00943832333279863)	0	0	4.42805607197144 (2.01468468763529e-21)	4.53343120159325 (1.48673985498835e-06)
GRHL2	-3.52841054302274 (5.81127486639392e-05)	0	-4.04433079169458 (4.84116941192416e-05)	-5.98532796103726 (0.00881968685923596)	0	0	0	0	2.91260293535685 (1.33266605537809e-14)	0
GRHL3	0	0	0	0	0	0	0	0	0	7.42237423892421 (0.000114736283664314)
GRHPR	0	0	0	0	0	1.57660711038717 (0.00167593102705609)	0	0	0	0
GRIA1	0	0	-4.13686836455078 (0.00314667796602476)	0	0	0	0	0	-4.69872573383751 (8.77107465725877e-05)	0
GRIA2	0	0	0	0	0	7.47343968741419 (0.00359481789269584)	0	0	0	0
GRIA3	0	0	0	0	0	6.13608393772006 (3.30000399953933e-05)	0	0	4.07368778712223 (0.00218030917150897)	0
GRID2	0	0	0	0	6.48222785639122 (0.00467673319314815)	0	0	0	0	0
GRIK2	0	0	0	0	0	0	0	0	5.31957454284036 (6.18729053107348e-08)	0
GRIK3	6.14286747998125 (6.64159189847632e-08)	0	0	7.39169182368079 (9.34507568680672e-06)	6.12735103573817 (0.00192526047013706)	6.56100085425968 (0.000513550764059771)	0	0	0	0
GRIK5	4.53828174309072 (2.25668182532867e-07)	0	0	5.28126066344561 (0.00083450527568284)	4.62028790299998 (0.000486595362936097)	4.055552490685 (0.0098153095740153)	0	0	0	0
GRIN2A	0	0	5.50495874980882 (0.000232583121583323)	0	5.96114275387634 (0.00319587739806226)	7.53271253665406 (0.00092526171127467)	6.29638350938734 (0.00273370970305203)	0	0	0
GRIN2B	0	0	0	0	0	6.20726515531401 (4.56687701354577e-07)	3.47959119546182 (0.00979370863191116)	0	0	0
GRIN2C	0	0	0	0	0	5.08885912234777 (0.00719191162900765)	0	0	0	0
GRIN3A	0	0	0	0	5.525281118786 (0.00125858935590946)	6.75002002762544 (1.25892358285979e-05)	4.59663972167988 (0.00215090537568625)	0	0	0
GRIP1	6.11871340386806 (1.68978820838611e-08)	0	0	6.61749316217728 (0.000698358067673283)	0	0	4.13912876542682 (0.00571708890710274)	0	7.05360779567602 (4.15581774898503e-10)	0
GRIP2	0	0	4.17991753268629 (9.10722740449069e-07)	0	0	0	0	0	0	0
GRK5	0	0	0	0	0	0	0	-3.10329653798613 (0.00666518308339644)	0	0
GRK6	0	0	0	0	0	0	0	0	-1.75451259840008 (0.00110322049020417)	0
GRM1	0	0	5.17879808159605 (0.000654340962652158)	0	6.24434293510046 (0.0075784156376657)	7.32624266357827 (0.00538433295435045)	0	0	0	0
GRM2	0	0	0	0	6.54901766634297 (0.000306706057878746)	5.52082589059189 (0.00705584955831895)	0	0	0	0
GRM4	0	0	0	0	0	5.5771120418126 (0.00465039293754586)	0	0	0	0
GRM5	0	0	0	0	0	5.92383765599611 (0.00893443873901692)	0	0	0	0
GRM8	0	0	0	0	0	6.72043210127567 (0.00212158947910585)	0	0	0	0
GRTP1	2.64376605635909 (0.00313848859323682)	0	0	0	0	0	0	0	0	0
GS1-358P8.4	2.53694251224019 (3.87394250224325e-07)	0	0	0	0	0	0	0	0	0
GSAP	0	0	-2.0083271599968 (0.0031136611048877)	0	0	0	0	0	0	0
GSDMC	0	0	0	0	0	0	0	0	0	5.6114572159425 (5.73074053055254e-06)
GSE1	0	0	0	0	0	0	0	0	1.79801785664306 (1.21758929281739e-08)	0
GSG1L	0	0	4.0679966420005 (0.006886707312059)	0	0	0	0	0	0	0
GSN	1.57717170080413 (5.24201884464376e-07)	0	2.80941613695819 (4.4260061773603e-11)	2.0417212425786 (0.000437091245260138)	0	0	0	0	0	0
GSTA3	0	0	0	0	0	0	0	0	4.43834627167621 (0.0025160207224549)	0
GSTM2	1.99471509365244 (0.00127298497623616)	0	0	0	0	0	0	0	0	0
GSTM3	2.79081299565979 (2.12416305475696e-06)	0	1.75614741154354 (0.00844560207948667)	3.0006707878298 (0.00429572214692054)	0	0	0	0	0	0
GSTM5	2.22576602026675 (0.00377414069511359)	0	0	0	0	0	0	0	0	0
GSTP1	0	0	0	0	0	-1.98428984498649 (0.00404832897730545)	0	0	0	0
GSTZ1	0	0	0	0	3.62058128965764 (0.00202455748325156)	4.91802427787867 (7.63021790699391e-07)	2.86566041130599 (0.00534753394334003)	0	0	0
GTF2H1	0	0	0	0	-2.31696666922871 (0.00502976319715659)	0	0	0	0	0
GTF2H5	1.88248487470591 (0.00389635999827513)	0	0	0	0	0	0	0	0	0
GTF2IRD2	0	0	0	0	3.99365024773926 (0.00136176479490761)	0	0	0	0	0
GTSE1	0	0	0	0	0	0	0	3.80303115702784 (0.00556412453067166)	0	0
GTSF1	0	0	0	0	0	0	0	0	2.95512095806006 (0.00176491308480879)	0
GUCD1	0	0	1.61395801648333 (1.08032661408619e-05)	0	0	0	0	1.94663941357991 (0.0076321885646983)	0	0
GUCY1A2	-2.10570760603677 (0.000183896643051856)	0	-1.57383696729263 (0.00612903522939493)	0	0	0	0	-4.59233554576844 (0.000316603951149217)	1.56767065435405 (0.00391785398200048)	-2.34654789921955 (0.00351532064858123)
GUCY1A3	0	0	0	0	0	0	0	-3.02858388393673 (0.00398648923260948)	0	0
GULP1	0	0	-2.30760209955828 (0.0007282719281345)	0	0	0	0	-6.10320488248368 (0.00570213416707378)	1.78937210314182 (0.000188726251885142)	0
GVINP1	0	0	0	0	0	0	0	0	0	-3.54965924421341 (0.00592970951467628)
GXYLT2	0	0	1.82936342403308 (0.00945627453557905)	0	0	0	0	0	0	0
GYLTL1B	0	0	0	0	0	0	0	0	3.73982377225615 (0.00162369579754913)	0
GYPA	0	0	0	0	0	0	0	6.92945532228935 (2.14263903634519e-09)	0	0
GYPC	0	0	0	0	0	0	0	2.22975896564594 (0.00380353941209512)	0	0
GYPE	0	0	0	0	0	0	0	6.22437810889325 (0.000110838392444284)	0	0
GYS1	0	0	2.37878528211967 (1.98192913847033e-05)	0	0	0	0	0	0	0
GYS2	0	0	0	0	0	7.53280282504311 (6.92424705311833e-08)	0	0	0	0
GZMA	0	0	0	0	0	0	2.78202916916592 (0.00033749087532186)	0	0	0
GZMB	0	0	-3.28855574092003 (0.00968973150351674)	0	0	0	0	0	0	0
H19	2.25218383667936 (3.59895687269743e-06)	0	4.29177155494805 (6.03281711155104e-16)	2.48008495677019 (0.0087996673511012)	4.53803869011117 (4.97079846609498e-10)	4.8289894811744 (5.69366393584787e-07)	0	0	7.63060941370264 (5.20940262036207e-93)	0
H2AFX	0	0	0	0	0	0	0	2.7735053267913 (0.00661172828608722)	0	0
H2AFY	-1.53571276468372 (1.23856914176712e-06)	-5.86627056390145 (0.00570195426668745)	-1.59159736225793 (6.43604009677722e-07)	-1.82300193070217 (0.00304607422280162)	-1.83401984951946 (0.000110916882727034)	0	0	0	0	0
H3F3A	0	0	-1.5732821143604 (0.00135579255147511)	0	0	0	0	0	0	0
H6PD	0	0	0	0	0	1.93808205913102 (0.000216486870960123)	0	0	0	0
HAAO	0	0	0	0	0	3.08931885631533 (0.000118532341210693)	0	0	0	0
HABP2	0	0	0	0	0	8.65965496849168 (4.33634913173317e-13)	0	0	0	0
HACE1	0	0	0	0	0	0	0	0	2.39396965509814 (1.58301988631205e-06)	0
HADH	0	0	3.0030385298611 (3.1402071784618e-09)	0	0	2.58043572744307 (0.00265921312062633)	0	0	0	0
HADHB	0	0	1.87064954328313 (6.12965060047935e-05)	0	0	0	0	0	0	0

HAGH	0	0	2.49425162085047 (7.16255020583438e-07)	0	0	1.75502819178681 (0.00711588474478884)	0	0	0	0
HAL	0	0	0	0	0	5.5735963162655 (3.47942928071552e-08)	0	0	0	0
HAMP	0	0	0	0	0	8.5270615864022 (6.38774954426112e-07)	0	0	0	0
HAND1	8.78241473708801 (5.43910165900767e-17)	0	6.64689554405235 (1.26260641973554e-07)	7.20832051987778 (0.000432609154539534)	0	0	0	0	0	0
HAND2	8.36049052902326 (1.67602492661668e-24)	0	7.34436736523642 (3.04916204492984e-15)	7.94833127921481 (1.06458146476711e-10)	0	5.4975049751746 (0.000666903468277912)	0	0	8.39339270193438 (4.09873713573279e-17)	0
HAND2-AS1	8.98787723168593 (8.76369619845691e-18)	0	7.23891920043112 (2.3940657367063e-10)	8.65867822113817 (2.30106178889904e-10)	5.83004520490158 (0.00374383828643843)	7.51482088130397 (7.44180232902481e-09)	0	0	8.48350139583148 (9.83764837610236e-14)	0
HAO1	0	0	0	0	0	9.44919420062332 (2.12783352066406e-15)	0	0	0	0
HAO2	0	0	0	0	0	6.95289693149051 (0.000127671742096855)	0	0	0	0
HAP1	0	0	0	0	6.37559784609272 (3.43108861799962e-06)	5.86676694827523 (0.00311580484739102)	0	0	0	0
HAPLN1	4.07405569695185 (0.00938035326024492)	0	0	0	0	0	0	0	6.74924717421772 (3.55343975883818e-05)	0
HAPLN2	0	0	0	0	0	6.735308362192 (0.00147218812141493)	0	0	0	0
HAPLN3	0	0	0	0	0	0	2.16026211787239 (0.00250951233330022)	0	0	0
HAVCR2	-2.29820109375861 (0.000744592507969169)	0	-2.78671964340139 (0.00164925316255879)	-4.87700259733474 (0.00519932267206632)	0	0	0	0	0	0
HAX1	0	0	2.11356903498546 (4.31799293939358e-06)	0	0	0	0	0	0	0
HBA1	-5.04167775213313 (2.43223837474299e-08)	0	0	-4.0887034800871 (0.00258877954939722)	0	0	0	3.77204156243072 (4.25726476746753e-06)	0	-7.81083799059839 (0.000463690803996314)
HBA2	-5.67654825056075 (6.44283660169943e-16)	0	0	0	0	0	0	2.94894491314613 (3.27499444043165e-05)	0	-5.30500313934776 (1.48051343230804e-06)
HBB	-6.64773243804846 (4.37414747900861e-24)	-6.73440108387287 (0.00201272769712061)	0	-3.46657801396433 (1.52709674991284e-05)	0	0	0	3.47161149207074 (7.32392272185562e-06)	0	-3.234323732419 (4.4884264985639e-05)
HBD	-4.15687398447886 (0.00805942165550977)	0	0	0	0	0	0	6.2227286120918 (4.58562027923778e-11)	0	0
HBEGF	-2.12287731067894 (8.8519407824177e-05)	0	0	0	0	0	0	-6.47520581108539 (0.00353138022651705)	-2.15953521042487 (4.02301547156928e-05)	0
HBG1	0	0	0	0	0	0	0	0	4.0698055565494 (0.000700043801170917)	0
HBG2	0	0	0	0	0	0	0	0	5.84663655563266 (0.000154900808203124)	0
HBM	0	0	0	0	0	0	0	7.39217250514775 (0.00029122728349668)	0	0
HCAR1	0	0	0	0	0	0	4.82088625435418 (0.00226278867687568)	0	5.377475377783 (7.57979990312645e-06)	0
HCK	-2.45024180959007 (7.23827039220885e-05)	0	-2.97897987595239 (0.0017483229072451)	-2.907150732297 (0.00653074897934924)	0	0	0	0	0	0
HCLS1	0	0	-2.31241855277048 (6.85574826605554e-06)	0	0	0	0	0	0	0
HCN1	0	0	0	0	0	7.2773212878179 (0.000111805621911897)	0	0	0	0
HCN2	0	0	4.46756002067042 (0.000131760109343164)	0	0	0	0	0	0	0
HCN3	0	0	0	0	4.73334539799482 (0.000273787045443291)	0	0	0	0	0
HCN4	0	0	7.4038962046369 (2.43095706235379e-10)	0	5.57856130299113 (0.00693597022972821)	6.72950614583378 (0.00266451951182455)	4.98293490361525 (0.00867341658092446)	0	0	0
HCP5	0	0	0	0	0	0	0	0	-2.31939272667017 (0.000126016103283372)	0
HDAC11	0	0	2.15043131721269 (0.00746598732429034)	0	0	0	0	0	0	0
HDAC4	1.76920147375287 (4.78698314379907e-05)	0	0	2.91943839842745 (0.000322169834376989)	0	0	0	0	0	0
HDAC5	0	0	1.68010491151357 (0.000117205518518005)	0	0	0	0	0	0	0
HDAC9	2.89563242017249 (1.12498739999825e-07)	0	2.45642861325644 (2.25235678672729e-06)	3.03312737608003 (0.00283488355953686)	0	2.24387425657741 (0.00664230107673297)	1.73804778021815 (0.0088255837006677)	0	2.11706893494304 (5.15615453288976e-06)	0
HDGF	0	0	0	0	0	0	0	1.5817750986397 (0.00752913844782134)	0	0
HDHD3	0	0	0	0	0	2.80591351652038 (0.00740926551122512)	0	0	0	0
HDX	0	4.79631410520824 (2.95273046901662e-05)	0	0	0	0	0	0	0	0
HEATR1	0	2.19438005440256 (0.00789682005063991)	0	0	0	0	0	0	0	0
HECTD4	0	0	0	0	0	0	0	1.97374404546998 (0.00966033812789291)	0	0
HECW1	0	0	0	0	0	5.48857163618035 (0.00254107863988861)	0	0	0	0
HECW2	-4.25752194026572 (7.59163995195622e-12)	0	0	-3.31221294781598 (0.00104503561653004)	0	0	0	-7.86323733895447 (0.00043415057879339)	0	-3.59032775577931 (0.000379079755448826)
HEG1	0	0	0	0	0	-1.6421834967934 (9.75335404456648e-05)	0	-5.82395684682192 (1.12907890216223e-06)	0	0
HELLPAR	0	0	0	0	5.19144702097533 (1.69420482610796e-09)	6.12011315134474 (1.0745244923418e-09)	4.20839858434046 (0.000127241471706217)	0	4.19933308502366 (2.31216694732017e-08)	0
HELLS	0	0	-2.41428849718603 (0.00340018217068786)	0	0	0	0	0	0	0
HEMGN	0	0	0	0	0	0	0	6.1258368176815 (3.86103747984588e-16)	0	0
HEPH	1.84615262368284 (0.00110530499519325)	0	0	0	0	0	0	0	0	0
HERC2P3	0	0	0	0	0	0	1.80879187245338 (0.00593832128133484)	0	2.02607075834442 (0.00104349360091695)	0
HERC6	0	0	0	0	0	0	0	0	1.88706508829365 (0.00755891515507284)	0
HES1	0	0	0	0	0	0	0	-4.7431278147671 (0.00989559339052734)	0	0
HES2	0	0	0	0	0	6.62972050443079 (0.00300730370188095)	5.05465448956228 (0.0010784384245881)	0	7.60692515905396 (4.24086406965194e-11)	0
HES6	0	0	0	0	0	0	0	6.92550165083614 (7.61848637271506e-07)	0	0
HES7	0	0	2.87481240978325 (0.00799496936116348)	0	0	4.08310764399734 (0.001622797408565)	0	0	0	0
HEXB	0	0	0	0	0	0	0	0	1.81740632980439 (0.000125063223331086)	0
HEY1	0	3.92626262656802 (0.00244252824895826)	0	0	0	0	0	0	1.98073277915934 (0.000567303740912898)	0
HEY2	0	0	2.17143346950615 (0.000154796822276059)	0	0	0	0	0	0	0
HEYL	-1.62802188716864 (0.00174704447751941)	0	0	0	0	0	0	0	0	0
HFE2	0	0	5.87559600704714 (8.77345083306932e-06)	0	0	8.04622399849171 (3.7090826145788e-09)	0	0	0	0
HGD	0	0	0	0	0	7.13082516939352 (1.61749518913103e-09)	0	0	0	0
HGF	-1.84916269096392 (0.000488159838942135)	0	-2.29111453262652 (0.0011778755962284)	0	0	0	0	0	2.62277745890627 (0.000250219291918226)	-4.04003139326648 (6.09302533300129e-05)
HGFAC	0	0	0	0	0	7.88372462058608 (2.36569509437294e-10)	0	0	0	0
HGSNAT	0	0	0	0	0	0	0	-2.39113419457108 (0.00800435859103417)	0	0
HHATL	0	0	9.21021359443615 (1.2629383262026e-18)	0	0	0	0	0	0	0
HHEX	0	0	0	0	0	2.89172947785433 (0.000527720960428047)	0	0	0	0
HHIP	0	0	-4.9053577229128 (8.27609099013654e-08)	-3.30514433959646 (0.00708277832071666)	0	0	0	-7.01339965495037 (0.00235774764989602)	-5.45681950858908 (5.73776540749635e-12)	-5.9061758881602 (0.000917318490304168)
HHIPL1	0	0	0	0	0	3.8238909433939 (0.000804492235821444)	0	0	0	0
HHLA1	0	0	0	0	0	7.18609492789082 (0.00581362640846632)	0	0	0	0
HIBCH	0	0	0	0	0	2.12065998589256 (0.00213181029349769)	0	0	0	0
HIC2	0	0	0	0	0	0	0	0	2.39126109580499 (1.45905681535073e-11)	0
HIF1A	-1.80839178209613 (2.19968255399237e-05)	0	-2.65650641234673 (3.16624029732673e-07)	0	0	-2.0961001750437 (0.00465128200798303)	0	-2.27434640011112 (0.000708073152823142)	-1.51232726246702 (1.09433332476896e-07)	0
HIF1A-AS2	0	0	-2.56997004254348 (0.00250856165379312)	-5.98849553768372 (0.00849088356054434)	0	0	0	0	-2.09319188016218 (0.000395069109327006)	0
HIF3A	0	0	0	0	0	0	0	-7.4858567415271 (0.000817276621611938)	0	0
HIGD2A	0	0	2.04079780497771 (0.00965719746462767)	0	0	0	0	0	0	0
HILPDA	0	0	-3.19078431387031 (1.08479356836338e-05)	0	0	0	0	0	0	0
HINFP	0	0	0	0	0	2.53061222003567 (0.000325402228017234)	0	0	0	0
HIP1R	-1.82839543866174 (0.000369071231834688)	0	0	0	0	0	0	0	-1.60454967012074 (0.000299569880212845)	0
HIPK3	0	-5.98403972127828	0	0	0	0	0	0	0	0

		(0.00519597738866727)								
HIST1H1B	0	0	0	0	0	0	1.59949658280551 (0.00831015690508552)	5.59285926344725 (6.23868317352964e-08)	0	0
HIST1H1C	0	0	0	0	0	0	0	2.7730557337874 (0.000106756596476883)	0	0
HIST1H1D	0	0	0	0	0	0	0	3.65907639328547 (2.64885189662321e-05)	0	0
HIST1H1E	0	0	0	0	1.57158897147332 (0.00905787656199044)	0	0	2.79204229510162 (0.000102251435029684)	0	0
HIST1H2AC	0	0	0	0	0	0	0	2.46019331690848 (0.00140698667877536)	0	0
HIST1H2AD	0	0	0	0	0	0	0	4.56698620145573 (8.51339163794251e-07)	0	0
HIST1H2AE	0	0	0	0	0	0	0	5.23756541269741 (2.85433968597801e-09)	0	0
HIST1H2AG	0	0	0	0	0	0	1.50369903748978 (0.00463843773623037)	5.14409279353533 (1.51367191969355e-06)	0	0
HIST1H2AH	0	0	0	0	0	0	0	6.66157676726956 (1.17109197736291e-08)	0	0
HIST1H2AI	0	0	0	0	0	0	0	6.10147791557019 (5.41856858992345e-08)	0	0
HIST1H2AL	0	0	0	0	0	0	0	5.66303801754013 (3.44573997406147e-08)	0	0
HIST1H2AM	0	0	0	0	0	0	0	6.80720155574357 (0.00424504638285965)	0	0
HIST1H2BB	0	0	0	0	0	0	0	5.61572142170163 (2.37724908119722e-06)	0	0
HIST1H2BC	0	0	0	0	0	0	0	3.57546598588669 (2.0685317453868e-07)	0	0
HIST1H2BD	0	0	0	0	0	0	0	3.63535018887862 (7.86887051104311e-08)	0	0
HIST1H2BE	0	0	0	0	0	0	0	4.99886593290533 (0.000186744238898733)	0	0
HIST1H2BF	0	0	0	0	0	0	2.47952679025271 (9.74758850625313e-05)	5.4442978305779 (8.9697622699735e-07)	0	0
HIST1H2BG	0	0	0	0	0	0	0	4.359638070485 (1.17300529956066e-06)	0	0
HIST1H2BH	0	0	0	0	0	0	0	5.46979256686816 (8.48784751365251e-07)	0	0
HIST1H2BI	0	0	0	0	0	0	0	4.68903391085806 (0.000413400387531356)	0	0
HIST1H2BJ	0	0	0	0	0	0	0	4.20240777413217 (4.87377155601601e-05)	0	0
HIST1H2BK	0	0	0	0	0	0	0	4.25513062638057 (5.37807790039586e-08)	0	0
HIST1H2BL	0	0	0	0	0	0	0	7.04722599036505 (2.64601741867437e-10)	0	0
HIST1H2BM	0	0	0	0	0	0	0	4.71857015209228 (6.1866463136846e-05)	-3.78853042954236 (0.00429431520713906)	0
HIST1H2BN	0	0	0	0	0	0	1.93765896036437 (0.000261884642367202)	5.08747156426477 (1.36425760070675e-09)	0	0
HIST1H2BO	0	0	0	0	0	0	2.80016020225287 (0.000420729486782827)	6.52131328089817 (1.07860337350539e-09)	0	0
HIST1H3B	0	0	0	0	0	0	0	5.38312910096522 (6.44194186783493e-07)	0	0
HIST1H3C	0	0	0	0	0	0	2.65154982896591 (0.00165664204020086)	6.20081190786717 (7.84849415416208e-08)	0	0
HIST1H3D	0	0	0	0	0	0	0	4.88596278984522 (6.55608374989124e-06)	0	0
HIST1H3F	0	0	0	0	0	0	0	5.75842989999352 (1.12665661904027e-06)	0	0
HIST1H3G	0	0	0	0	0	0	2.7433091738811 (0.000627806669470028)	5.48724751036196 (3.11527559253534e-05)	0	0
HIST1H3H	0	0	0	0	0	0	1.66444400873658 (0.00982507893511922)	4.68552672033178 (0.000596146327817938)	0	0
HIST1H3I	0	0	0	0	0	0	0	5.05454102865378 (1.63896820926669e-06)	0	0
HIST1H3J	0	0	0	0	0	0	0	6.67610028479458 (0.000947138400539343)	0	0
HIST1H4A	0	0	0	0	0	0	0	6.80250431028387 (0.000486899312846794)	0	0
HIST1H4B	0	0	0	0	0	0	0	3.80647992128716 (0.00142936834624417)	0	0
HIST1H4C	0	0	0	0	0	0	0	3.2048299926219 (0.000298470226410176)	0	0
HIST1H4D	0	0	0	0	0	0	0	5.17384529482502 (0.000128594984299444)	0	0
HIST1H4E	0	0	0	0	0	0	0	4.08776894617465 (7.83603337496997e-05)	0	0
HIST1H4H	0	0	0	0	0	0	0	3.65669819532857 (9.33247336763707e-05)	0	0
HIST1H4I	0	5.49438462062331 (8.34792342243088e-05)	0	0	0	0	1.89892795022904 (0.00272479336446519)	4.26775735873277 (0.00195654902032126)	0	0
HIST2H2AB	0	0	0	0	0	0	0	4.44898146902006 (5.03509670181491e-07)	0	0
HIST2H2BE	0	0	0	0	0	0	0	2.43288717090785 (0.00592180076979399)	0	0
HIST2H2BF	0	0	0	0	0	0	0	3.29244485237707 (0.000119818928599138)	0	0
HJURP	0	0	0	0	0	0	0	5.41030721249649 (2.67673606128587e-07)	0	0
HK1	0	0	0	0	0	0	0	1.57433529385977 (0.00888410010225996)	0	0
HK2	-2.1173182355438 (0.00457035952478808)	0	0	-3.00788200795215 (0.000424896466898861)	0	0	0	0	0	0
HK3	-2.76448093618601 (0.000148039344968746)	0	0	-4.02027962777973 (0.00300038561868871)	0	0	0	2.71960502063203 (0.00119351516811201)	-2.02143528092076 (0.00446849733252841)	0
HKDC1	0	0	0	0	0	0	0	0	-4.88740878703256 (4.46644462758774e-05)	0
HLA-A	0	-3.6661406441614 (0.00165950035772021)	0	0	0	0	0	0	0	0
HLA-B	-1.5901191230527 (5.04465121226223e-05)	0	0	-2.1017813551293 (0.00195760450430748)	0	0	0	0	0	0
HLA-C	-1.51967972580711 (4.7398621596168e-06)	0	0	0	0	0	0	0	0	0
HLA-DMA	-1.74957546818459 (0.00983622938868572)	0	-1.87841998051249 (0.00587899500637094)	0	0	0	0	0	0	0
HLA-DMB	-2.94493686260513 (2.71751154352203e-05)	0	-3.48023868313038 (1.99371243796221e-05)	0	0	0	0	0	0	0
HLA-DOA	0	0	-3.1845602766992 (0.00513646098813287)	0	0	0	0	0	0	0
HLA-DQA1	0	0	-3.86058758854087 (1.23336405193663e-05)	0	0	0	0	0	0	0
HLA-DQB1	0	0	-2.74937902547013 (3.04954611148194e-05)	0	0	0	0	0	0	0
HLA-DRA	0	0	0	0	0	0	0	-3.0587969181858 (0.00777154279213516)	0	0
HLA-DRB5	0	0	0	-3.64890879484102 (0.00621622362366643)	0	0	0	0	0	0
HLA-F	0	0	0	-2.74250913048082 (0.00299063370738448)	0	0	0	0	0	0
HLA-G	0	0	0	0	0	0	0	0	3.01177209957187 (2.962339100881e-07)	0
HLA-L	0	0	0	0	0	0	0	0	-1.82221985478954 (0.00333876615593305)	0
HLF	3.24243320772496 (6.19633679014122e-13)	0	0	0	0	2.43078947919452 (0.000562174624097339)	0	0	0	0
HMBS	0	0	0	0	0	0	0	3.74898217691283 (0.000264858617808859)	0	0
HMCN1	-1.52466947131291 (7.17940345731075e-05)	0	-1.55973580549491 (0.00348729932400724)	-2.02769751895136 (0.00480997748142245)	-2.24523609108749 (0.000232732071729729)	-1.67964618219078 (0.0019293079882088)	0	-8.93796564871982 (3.51858304645712e-05)	-2.23633008016662 (3.25863613556384e-08)	-2.9748974425757 (5.89189415697559e-05)
HMCN2	3.68296515438562 (4.63300375158523e-11)	0	0	4.11728341581434 (2.23565751659318e-05)	2.84291125951198 (0.00304585289383791)	0	0	0	0	0
HMGA1	-2.22003170530629 (0.000111062692416506)	0	0	0	0	-2.27391025373073 (0.00122313002108655)	0	0	-1.57204816325296 (0.000437981109584961)	0
HMGA2	0	0	0	0	4.00511536604401 (0.00110456645072669)	0	0	0	0	0
HMGB1	0	0	0	0	0	0	0	1.85312949640876 (0.00445172147828178)	0	0
HMGB1P26	0	0	0	0	0	7.18609492789082 (0.00581362640846632)	0	0	0	0
HMGB2	0	0	0	0	0	0	0	3.51039940648499 (6.66420736290558e-08)	0	0
HMGB3	0	0	0	0	0	0	0	0	3.21118368012694 (1.35428477228266e-09)	0
HMGCR	0	0	-1.94273710693414 (0.00320181541566055)	0	0	0	0	0	0	0
HMGCS2	4.8749455291705 (0.00418462895597888)	0	7.60664092611212 (2.23840211279177e-09)	0	0	11.6435689048137 (5.78151217985267e-15)	0	0	0	0
HMGN3-AS1	0	0	0	0	0	0	0	0	3.04222516009089 (9.63072330766402e-06)	0

HMGN5	0	0	0	0	0	0	0	3.02773871862654 (0.000378531157212045)	-1.5932148454075 (0.00341778554689515)	0
HMHA1	-2.62004828776891 (5.31934529017332e-05)	0	-2.39177093042307 (0.00326479319566102)	0	0	0	0	0	0	0
HMOX1	0	0	-4.33509420506565 (1.26504796301947e-08)	-6.24663047781624 (6.19122101020374e-07)	0	0	0	-4.45220045851232 (0.000106635866089495)	-2.65525333029527 (1.04612692458918e-06)	0
HMOX2	0	0	1.73246492645045 (3.16738188926414e-06)	0	0	0	0	0	0	0
HMP19	6.06042753721022 (1.52504446061016e-07)	0	0	6.7437870533237 (0.000378030430674534)	0	0	0	0	0	0
HN1	0	0	0	0	0	0	0	0	1.81193392266974 (0.000174288635532163)	0
HNF1A	0	0	0	0	0	7.18483158284125 (5.77471840788796e-09)	0	0	0	0
HNF1A-AS1	0	0	0	0	0	7.47886563817148 (1.5470901890477e-07)	0	0	0	0
HNF1B	-3.43415447336873 (0.00938959149464048)	0	0	0	3.8092753596042 (7.89475285723503e-08)	0	0	0	-3.89287429155454 (0.000463638744500341)	0
HNF4A	0	0	0	0	7.059094244196 (0.000754370310964906)	9.62556332945228 (2.51271568403293e-12)	0	0	0	0
HNF4A-AS1	0	0	0	0	0	6.57068735078676 (0.00149599561241224)	0	0	0	0
HNRNPA1P22	0	0	0	0	0	0	0	0	4.38885346289345 (5.93431585446769e-07)	0
HNRNPA1P54	0	0	0	0	0	5.94390296887754 (0.00357921403500104)	0	0	0	0
HNRNPA2B1	0	-1.89485055074696 (0.00546682434009699)	0	0	0	0	0	0	0	0
HNRNPA3P6	0	6.08470337112793 (3.80110868689693e-09)	0	0	0	0	0	0	0	0
HNRNPCP2	0	0	0	0	0	0	0	0	-2.32720795538492 (0.000507648504772894)	0
HNRNPCP7	0	0	0	0	0	4.58103611802094 (0.00910334259842879)	0	0	0	0
HNRNPH3	0	0	0	1.74250315041844 (0.00309667117818573)	0	0	0	0	0	0
HOGA1	0	0	5.26093211479684 (1.01162160144054e-05)	0	0	5.76787340169745 (1.06439599530133e-05)	0	0	0	0
HOMER1	0	0	2.99099840806609 (6.6671692388468e-09)	3.97362894727401 (1.58548353750915e-05)	0	0	0	0	2.41624660571807 (1.08095786673749e-06)	0
HOMER2	0	0	1.87721829815854 (0.000767856642759915)	0	0	2.88897882188583 (7.81812942514185e-05)	0	0	0	0
HOOK2	0	0	1.6659652530373 (0.00879262587323026)	0	0	0	0	0	0	0
HOPX	-3.34802443875979 (2.79878506050483e-09)	0	-3.4705443899279 (3.22270458010117e-09)	-2.86716680435245 (0.00136936967057694)	-2.99485723395109 (0.00051975744939941)	0	0	-8.25115227299159 (0.000185723598336486)	0	0
HOTAIRM1	0	0	0	0	0	0	0	0	-2.39047051586116 (0.00580891522567681)	0
HOTTIP	0	0	0	0	6.47559966567641 (0.0042482603748159)	0	0	0	0	0
HOXA-AS2	0	0	0	0	3.0998836543946 (0.0038920376358916)	0	0	0	0	0
HOXA-AS3	0	0	0	0	4.87522671166986 (0.000425831301356354)	0	0	0	0	0
HOXA10	0	0	0	0	0	0	0	0	4.97943266784478 (1.64896959275598e-05)	0
HOXA11	0	0	0	0	0	0	0	0	5.4766923673642 (1.30042509837309e-05)	0
HOXA13	0	0	0	0	0	0	0	0	7.78055960929567 (3.95849212174947e-12)	0
HOXA5	0	0	-3.49557677289365 (0.00408815371818691)	0	0	0	0	0	0	0
HOXB-AS3	3.56911003429119 (0.00170290961319767)	0	0	0	0	0	0	0	0	0
HOXB13	0	0	0	0	6.81201551833002 (0.00158736055535963)	0	0	0	0	0
HOXB2	1.53895166763953 (0.00376427995028397)	0	0	0	0	0	0	0	0	0
HOXB3	0	0	0	0	2.00221797656119 (0.00168207018768408)	0	0	0	0	0
HOXB4	2.37442323346261 (0.000671437829768929)	0	0	0	0	0	0	0	0	0
HOXB5	2.32831014665444 (0.00545485276342075)	0	0	0	3.03059969971975 (0.0069385670507066)	0	0	0	-3.19335233870604 (0.00953461260152151)	0
HOXB6	1.89977629688811 (0.00184403614948729)	0	0	0	0	0	0	0	0	0
HOXB7	3.03225894958942 (9.12559937559096e-08)	0	0	3.55530811190222 (0.0019201175698403)	0	0	0	0	0	0
HOXB8	6.24447119269322 (3.38310017526609e-07)	0	0	0	6.97428225467614 (1.59442216630047e-05)	0	0	0	0	0
HOXB9	0	0	0	0	5.96622682455296 (4.74351757477649e-05)	6.17700295717285 (0.00190166610691764)	0	0	0	0
HOXC10	0	0	0	0	7.93775499089576 (9.34164216959003e-09)	0	0	0	0	7.11203065854564 (0.000618894886710335)
HOXC12	0	0	0	0	0	7.19462663457433 (0.00558633867875564)	0	0	0	0
HOXC13	0	0	0	0	0	6.59090583247402 (0.00279358708149304)	0	0	0	0
HOXC4	5.87554295714449 (1.97736361135847e-06)	0	0	0	0	0	0	0	4.33617939065659 (0.000444520770834878)	0
HOXC6	5.99126992295545 (8.41232409845948e-12)	0	0	0	0	0	0	0	2.85939707854828 (0.00349188757607358)	0
HOXC8	8.32532310758572 (5.84061591919695e-16)	0	0	7.27245160959529 (0.000308198984895893)	0	0	0	0	5.19900562226224 (5.98138058129503e-05)	0
HOXC9	5.17966492178617 (0.000149481905224866)	0	0	0	0	0	0	0	0	0
HOXD10	0	0	0	0	6.21789778565674 (0.000952420421888696)	0	0	0	4.73886808955167 (0.00112153048801157)	0
HOXD13	0	0	0	0	0	7.533084448249 (0.000131501781312465)	0	0	0	0
HOXD8	5.69718697317056 (1.33717681530842e-06)	0	0	0	7.85649803109226 (3.17696602840281e-10)	0	0	0	4.14555818420028 (0.00581342274118408)	0
HOXD9	0	0	0	0	6.00750308254399 (0.000124462820186156)	0	0	0	0	0
HP	0	0	0	0	0	9.58096692059778 (6.40257377743241e-31)	2.32722161249924 (0.00663547747344165)	3.44638774785388 (0.0051299067823235)	0	0
HP09025	0	0	0	0	0	6.49941868802561 (0.00485344882487319)	0	0	0	0
HP1BP3	0	-5.58711478894244 (0.00814497656085729)	0	0	0	0	0	0	0	0
HPCAL1	-1.63101011782108 (0.00381744091046756)	0	0	-2.70706196526014 (0.00767734348158849)	0	0	0	0	0	0
HPCAL4	0	0	0	0	0	6.49716084993858 (0.00184085995008428)	0	0	0	0
HPD	0	0	0	0	0	8.80731577440823 (1.00384637632025e-20)	0	0	0	0
HPGD	0	0	-5.0134108449508 (1.47831193088334e-05)	0	0	0	0	0	3.6260431228334 (1.08549189451667e-08)	0
HPGDS	0	0	0	0	0	0	0	0	3.32208901516094 (0.000422007472044293)	0
HPN	-3.17178557791332 (0.00868822778018784)	0	0	0	0	4.7131190895088 (9.18328015383201e-09)	0	0	0	0
HPN-AS1	0	0	0	0	0	5.71066006635506 (0.00078760475861801)	0	0	0	0
HPR	0	0	4.57683137566763 (4.62749393261195e-05)	0	0	7.83775732514832 (2.26581529338998e-13)	0	0	0	0
HPS3	0	0	-1.87148921494742 (0.000198947611725783)	0	0	0	0	0	0	0
HPS4	0	0	0	0	0	0	0	2.25047789310727 (0.00194085209419446)	0	0
HPS5	0	0	-1.72503002276692 (0.000110678974679728)	0	0	0	0	0	0	0
HPSE2	2.10945646150754 (0.00761079286747202)	0	0	0	0	0	0	0	0	0
HPX	0	0	0	0	4.71038568389688 (0.00223346267704189)	9.51882576816266 (5.78743526536428e-20)	0	0	0	0
HR	0	0	0	0	3.9749810861325 (0.00774611804604646)	5.04202518246581 (0.00225339271958353)	0	0	0	0
HRASLS2	0	0	0	0	0	0	3.65352631804146 (0.00623603868919637)	0	0	0
HRC	0	0	8.50743178775271 (1.79251388414455e-49)	0	0	0	0	0	0	0
HRG	0	0	0	0	0	10.3532785564348 (7.9406962720861e-19)	0	0	0	0
HRH2	0	0	0	0	0	0	0	3.5093017988578 (0.00743415329647198)	0	0
HRK	0	0	0	0	5.7800642702998 (0.00591384444719865)	7.94054229941438 (0.000297010697156367)	0	0	0	0
HRSP12	0	0	0	0	0	3.12895712605942 (0.000983363164264697)	0	0	0	0
HS1BP3	0	0	0	0	1.82019025174246	0	0	0	0	0

					(0.00109367322499455)					
HS2ST1	0	0	-2.25172756969633 (7.60130211337605e-05)	0	0	0	0	0	0	0
HS3ST2	-5.46662121910611 (0.000336435878702696)	0	0	0	0	0	0	0	-3.42792049566247 (0.00706019243689958)	0
HS3ST3B1	-3.82826904885856 (0.00615385053628427)	3.95267726032153 (0.00352277462091265)	0	0	0	3.37160555051342 (8.05344612774315e-06)	0	0	0	0
HS3ST4	0	0	0	0	6.64816874282679 (0.00347040281013931)	6.61748565105267 (0.00329571580944009)	0	0	0	0
HS6ST3	0	0	0	0	4.32758640679131 (0.00243705455099881)	5.50326486373175 (0.00100840359495833)	0	0	0	0
HSD11B1	0	0	0	0	0	2.22213402961158 (0.00253380500189452)	0	0	2.56615065508197 (0.00848365106867821)	0
HSD17B1	0	0	0	0	0	0	0	0	3.19671711103952 (0.0085177699337469)	0
HSD17B13	0	0	0	0	0	4.14338668978774 (0.00034550093289981)	0	0	0	0
HSD17B2	0	0	0	0	0	5.25755803567665 (2.10825868860138e-05)	0	0	6.809259188447 (3.36771204847415e-08)	0
HSD17B6	0	0	0	0	0	5.93302036636354 (2.1790411353949e-10)	0	0	0	0
HSD3B1	0	0	0	0	0	0	0	0	10.596156951714 (5.76590583609949e-21)	0
HSD3B7	0	0	0	0	0	4.3513128313105 (0.00907382459194566)	0	0	0	0
HSD3BP5	0	0	0	0	3.67389068172242 (0.00708956054818854)	0	0	0	0	0
HSF4	-2.50985789655306 (0.00465871421143765)	0	0	0	0	0	0	0	0	0
HSH2D	0	0	0	0	0	0	2.1448618484125 (0.000224686015571757)	0	0	0
HSP90AA2P	0	4.54819625288497 (0.000543423868176195)	2.97682215814675 (0.001431859404008)	0	0	0	3.5565605104252 (0.000114404543497273)	0	0	0
HSP90B2P	0	0	0	0	0	4.23487672988468 (0.00134161922188341)	2.76670097652932 (0.00959751659355121)	0	0	0
HSPA12A	2.3948862112528 (2.36408065672661e-08)	0	1.88194274767229 (0.00114377905967462)	0	2.21567518038722 (0.00871695390707163)	0	0	0	0	0
HSPA1A	0	0	0	0	0	0	0	0	-3.02858694663764 (2.73000374648128e-09)	0
HSPA1B	0	0	0	0	0	0	0	0	-2.68886729077057 (1.54393440373135e-06)	0
HSPA1L	3.69151910426839 (0.00527391600332658)	0	0	0	0	4.91756523036799 (0.0052991349199106)	5.21228521842557 (1.85972840773017e-05)	0	0	0
HSPA2	3.58142719082132 (3.36716062389673e-12)	0	0	4.56694948643228 (2.70819602363926e-07)	0	0	0	0	3.15313197123925 (3.87927928182503e-13)	0
HSPA4L	0	0	0	0	0	0	0	0	-2.19844719406747 (0.00124748121062789)	0
HSPA6	0	0	-2.91077902760245 (0.000432526621740318)	0	0	0	0	0	-3.69076548792881 (6.31115381532452e-07)	0
HSPA7	0	0	0	0	0	3.17414221093194 (0.0062566427882066)	2.19590920091159 (0.0052943703998477)	0	2.62296880015781 (0.00118722156596683)	0
HSPA8P8	0	0	0	0	0	7.26986837985775 (0.00668205493536917)	0	0	0	0
HSPA9P1	0	0	0	0	0	6.19233246862476 (0.00176002804533406)	4.44881312869176 (0.00991338558833262)	0	0	0
HSPB1	0	0	2.99958084618332 (2.10255381314357e-09)	2.20787404310386 (0.00179578303570988)	0	0	0	0	0	0
HSPB1P1	0	7.36344265042506 (8.25950777977784e-06)	4.42496009945566 (0.000456174941514145)	0	0	0	0	0	0	0
HSPB3	0	0	8.16241717627926 (1.83497094279348e-14)	0	0	0	0	0	0	0
HSPB6	2.69988912435239 (3.46125818380032e-10)	0	2.53106920651336 (5.23335732448481e-07)	3.64652922309117 (1.85798184541162e-06)	0	0	0	0	0	0
HSPB7	0	0	6.70289437839259 (3.74769294729956e-30)	2.76108258401477 (0.0023563520460001)	0	0	0	0	-2.5165375666064 (1.65568529268386e-05)	0
HSPB8	0	0	3.14765354438867 (2.43361292571356e-10)	0	0	-2.02200643203398 (0.00695293679818035)	0	-3.67573850089001 (0.00416584498990702)	0	0
HSPBAP1	0	0	-1.72102675662658 (0.00469067651065726)	0	0	0	0	0	0	0
HSPE1	0	0	0	0	0	0	0	0	-1.62123878385067 (0.000371602194879231)	0
HSPG2	0	0	1.8286072057042 (1.67811128666383e-08)	1.6732766645594 (0.0097340250779206)	0	0	0	-4.16907541401146 (7.30833367423454e-07)	0	0
HSPH1	0	0	0	0	-2.87460578267136 (0.000276738741758711)	0	0	0	0	-2.81504220851119 (0.00151307740290766)
HTR1D	0	0	0	0	0	6.31040539604999 (0.00647657119037574)	0	0	0	0
HTR2A	3.72005147324643 (0.0038703400899211)	0	0	0	0	0	0	0	0	0
HTR2B	2.02819269660463 (0.00861170924599468)	0	0	0	0	0	0	0	0	0
HTR3B	0	0	0	0	5.83005063945246 (0.00374446131635038)	0	0	0	0	0
HTR4	0	0	4.81631920359131 (0.00681523073252896)	0	0	7.327051986738 (0.00538566466583367)	5.95283319372962 (0.0078140374935987)	0	0	0
HTR7P1	0	0	0	0	3.29147596534756 (0.00450447301319531)	0	0	0	0	0
HTRA1	0	0	0	0	0	0	0	-6.23235970496666 (0.00595377057708778)	2.7319613235538 (2.22160406403389e-09)	0
HTRA3	0	0	1.79967428438708 (0.00130117112306779)	0	0	0	0	0	0	2.41065513923854 (0.00278663504173737)
HTRA4	0	0	0	0	0	0	0	0	8.11688924339313 (7.1729150438328e-13)	0
HUNK	0	0	0	0	4.35132466489283 (1.09877823486553e-06)	5.35942725081265 (1.69812656818885e-05)	2.95391492832396 (0.0097873039100846)	0	2.64937315148569 (0.000646899786431916)	0
HYAL1	0	0	1.61383744761352 (0.00515698496412205)	0	0	0	0	0	-3.15071227273382 (6.97578129627531e-06)	0
HYAL2	-2.4859142051373 (8.97950184855349e-07)	0	0	0	0	0	0	0	0	0
HYAL4	0	0	0	0	0	0	0	0	4.98584062422163 (5.83091935044474e-06)	0
HYDIN	-4.0955135708413 (0.0010646841717074)	0	-4.31844513380991 (0.00120024652546278)	0	0	0	0	0	-4.26300809902063 (0.000231096386433368)	0
HYKK	0	0	0	0	0	2.90808243874729 (0.00469080171106)	0	0	0	0
HYMAI	0	0	0	0	0	0	0	0	3.9445867902486 (4.46480521352275e-09)	0
ICA1	-1.65468553462123 (0.00322031606517558)	0	0	0	0	0	0	0	0	0
ICA1L	1.80937496511499 (0.000962226612587703)	3.97372462360061 (0.00220215161057057)	0	0	0	0	0	0	1.67751535486805 (0.00126201392472854)	0
ICAM1	-2.57630146272951 (6.72496152967308e-06)	0	-3.08390738057821 (1.46212599999211e-09)	-2.63408819482885 (0.000175670479533636)	-1.64102163866155 (0.00252415588968143)	0	0	-3.99152964113448 (7.77301677462569e-06)	-2.6358661747489 (2.27452276876293e-10)	0
ICAM2	0	0	2.09395961928475 (0.00376468375811997)	0	0	0	2.04714027738793 (5.88540665561021e-05)	0	0	0
ICAM5	-4.31782246521859 (0.00092914698820108)	0	0	0	0	0	0	0	-2.67247938933175 (0.00239344183353583)	0
ICK	0	0	0	0	0	0	0	0	1.92242155927833 (3.4151445115661e-06)	0
ID1	0	0	0	0	0	0	0	-4.25167534620633 (0.00915750649517784)	0	0
ID2	0	0	0	0	0	2.50339530524195 (0.00288109928125039)	0	0	0	0
ID4	0	0	-2.96420913249654 (2.17983990919649e-05)	0	0	0	0	-7.08050573775956 (0.00165806447496843)	0	0
IDH1	0	0	-2.20319560226456 (0.00204197563413577)	-3.74168393629584 (0.00210100453290339)	0	0	0	0	0	0
IDH2	0	0	3.45997034708152 (1.7072626258728e-10)	0	0	0	0	0	0	0
IDH3A	0	0	2.2168231405317 (1.49215298479028e-12)	1.94390190768416 (0.0049427622166884)	0	0	0	0	0	0
IDI1	-1.60317351044034 (0.00381394520946321)	0	-2.63655051818176 (1.62496064831581e-05)	0	0	0	0	0	0	0
IDO1	0	0	0	0	0	0	2.56972219921703 (0.00599327975727412)	0	2.35836133650117 (0.0076992805302474)	0
IDO2	0	0	0	0	0	6.45077925459454 (0.00401811255958677)	0	0	7.76638555213091 (7.8552389526522e-05)	0
IDS	0	0	0	0	0	0	0	-2.84749769578409 (0.000322117908802118)	0	0
IDUA	0	0	0	0	0	0	0	0	-2.55679192428138 (0.00180190400240011)	0
IER2	-1.66838159427322 (0.00140048487772136)	0	0	0	0	0	0	0	0	0
IER3	0	0	0	0	0	0	0	-4.29083395793193 (0.000270357951503996)	0	0
IER5	-1.84059854170165 (1.28403083001224e-05)	0	0	0	0	0	0	0	0	0
IER5L	0	0	0	0	0	2.5139329274197 (0.000315794929806136)	0	0	0	0
IFI27	0	0	0	-3.8883986601539 (0.000795720038706582)	0	0	0	0	0	0

IFI27L2	1.81245834729481 (0.000802358710343987)	0	0	0	0	0	1.61787602868325 (5.19503708580799e-05)	0	0	0
IFI30	-4.62682191074615 (1.25795578434647e-05)	0	-4.94046919597072 (4.1124077308048e-06)	-6.5686900450193 (0.00435183068655666)	0	0	0	0	-2.88157346302252 (3.71077432541479e-05)	0
IFI44L	0	0	0	-3.84054420202049 (0.000945181205289088)	0	0	0	0	0	0
IFI6	0	0	0	0	0	0	0	0	2.13888003056653 (0.00056816042952177)	0
IFIH1	0	0	0	0	0	0	0	0	1.79167468370731 (0.00189442853669727)	0
IFIT1	0	0	0	0	0	0	0	0	2.75404742903537 (0.00527841255511332)	0
IFIT2	0	0	0	-3.94984700437294 (0.00762715894740256)	0	0	0	0	0	0
IFIT3	0	0	0	-3.76990489093577 (0.000545943890951705)	0	0	0	-5.0325882105466 (0.00305204278168776)	1.85840064967365 (0.00585591638311018)	0
IFITM1	1.52530641228191 (0.00810353817274263)	0	0	0	0	0	1.7102453265262 (0.00137942983518516)	0	0	0
IFITM10	-3.95303872506651 (0.00639670132555408)	0	0	0	0	0	0	0	0	0
IFITM2	0	0	0	0	0	0	0	0	-1.88175365261638 (5.05669643818054e-07)	0
IFNGR1	0	0	0	0	-2.06358157889296 (0.00192585784945095)	0	0	-2.22282440913625 (0.00600151016182852)	0	0
IFNLR1	0	0	0	0	0	3.41752043949373 (0.00219696334855545)	0	0	0	0
IFT43	0	4.30658802109087 (0.000880643499598584)	1.84844538345797 (0.00860689908666426)	0	0	2.87645977024547 (0.00708573025314894)	0	0	0	0
IFT57	0	0	-1.98663178270817 (4.14873004822111e-05)	0	-2.01368130635399 (0.00968952772398721)	-2.21642034066276 (0.000805821135290278)	0	0	-2.27225772163274 (2.27461752300559e-07)	0
IGBP1P4	0	0	0	0	0	7.26970403665886 (0.00105567204388479)	0	0	0	0
IGDCC3	0	0	0	0	0	0	0	0	4.34401763594825 (0.0017082046581172)	0
IGDCC4	0	0	0	0	0	0	0	0	3.11623345042309 (3.57232513635662e-05)	0
IGF2	4.24525827925181 (3.21882367489988e-13)	3.3801471275822 (3.72983789878511e-05)	5.64707754367409 (3.02691441361622e-19)	4.12222529720175 (5.15530968700942e-08)	6.01872575568109 (3.29672984662387e-27)	6.23632770935829 (8.10312785184443e-15)	2.26568725753867 (0.00330357190378045)	0	9.34661761799176 (4.52217185573573e-93)	2.07410580370214 (0.00858355459825511)
IGF2-AS	0	0	0	0	0	5.89663347622359 (0.00299891021656507)	0	0	0	0
IGF2BP1	0	0	0	0	6.81201551833002 (0.00158736055535963)	0	5.41680930764355 (0.00606545936103408)	0	6.94388928730353 (2.30143806961379e-10)	0
IGF2BP2	0	0	0	-3.1838610661755 (0.000437571157643332)	0	0	0	1.82918205538936 (0.00748601918978426)	0	0
IGF2BP3	0	0	0	0	0	0	0	0	5.50129560386186 (2.15109817424199e-22)	0
IGFBP1	0	0	0	0	0	10.7000579365998 (1.87630805645192e-13)	0	0	13.0425524520527 (4.60326950799551e-11)	0
IGFBP2	0	0	0	0	0	2.30808881984241 (0.00389382170882527)	0	0	0	0
IGFBP3	0	0	0	0	0	2.32201322988334 (0.00328691723639272)	0	0	5.81810946310557 (3.06912598480698e-30)	0
IGFBP4	1.54500935189565 (6.38130452584404e-06)	0	0	0	1.50446638916866 (0.00116616838566264)	0	0	-1.97034504490944 (0.00284976259738117)	0	0
IGFBP5	1.70998993501786 (9.03366638066787e-07)	0	0	0	0	0	0	0	2.44657392979339 (1.12102886444314e-09)	0
IGFBP6	2.80985952046697 (1.4051577066793e-06)	0	2.65647629025924 (0.00132056540187846)	3.42634913607075 (0.000543055016535289)	0	0	0	0	0	0
IGFBP7	0	0	0	0	3.03976402303506 (1.73710470244922e-05)	0	0	-2.21708178025738 (0.0026072097228413)	0	0
IGFN1	-4.02469614690576 (0.000128478854250974)	0	-5.20574696915857 (1.11585484608523e-05)	-7.27234714149064 (0.00302368642516036)	0	0	0	0	-6.24996602620859 (1.60360861035971e-08)	0
IGHA1	0	0	0	0	0	0	2.68115611865656 (9.89634386172154e-05)	0	0	0
IGHA2	0	0	0	0	0	0	2.38120021344127 (0.00249861657301039)	0	0	0
IGHD	0	0	0	0	0	0	3.91039244402178 (0.000187619779847679)	0	0	0
IGHG1	0	0	0	0	0	0	2.52442564380694 (0.000853330054060303)	0	-4.72678668618463 (0.00358284181607087)	0
IGHG2	0	0	0	0	0	0	2.43645166170058 (0.000789748865754684)	0	-4.0097995964746 (0.000825348874722419)	0
IGHG3	0	0	0	0	0	0	3.55842228146159 (1.90714056878334e-05)	0	-6.46270057106466 (0.00373879018833835)	0
IGHG4	0	0	0	0	0	0	3.52267218989338 (8.89993537621847e-06)	0	0	0
IGHGP	0	0	0	0	0	0	3.00031642121892 (0.00219372853140774)	0	0	0
IGHM	0	0	0	0	0	0	3.59695540903104 (1.52745895433799e-05)	0	-3.39575964949278 (6.11855078730997e-05)	0
IGHV1-18	0	0	0	0	0	0	3.33402358315651 (3.89632770986702e-05)	0	0	0
IGHV1-2	0	0	0	0	0	0	3.58035075833799 (0.0029308691012026)	0	0	0
IGHV1-46	0	0	0	0	0	4.19607727265849 (0.00395124149298874)	4.49033213400101 (7.63487155434141e-05)	0	0	0
IGHV1-69-2	0	0	0	0	0	0	4.29961452731196 (0.000342421669214398)	0	0	0
IGHV2-26	0	0	0	0	0	0	3.98698160699552 (0.00217312963045767)	0	0	0
IGHV2-5	0	0	0	0	0	0	3.50108923615615 (0.00547076668996494)	0	0	0
IGHV2-70	0	0	0	0	0	0	4.35517820554309 (0.00558675061722343)	0	0	0
IGHV3-15	0	0	0	0	0	0	2.40845430308854 (0.00351483705790848)	0	0	0
IGHV3-21	0	0	0	0	0	0	3.13851037753034 (0.00111321805009038)	0	0	0
IGHV3-23	0	0	0	0	0	0	2.6768466371022 (0.00141681482736862)	0	-4.56085920455204 (0.00160457208867371)	0
IGHV3-30	0	0	0	0	0	0	2.9745724573634 (0.00125670777758514)	0	0	0
IGHV3-33	0	0	0	0	0	0	3.0531795127887 (0.00213013914190159)	0	-4.56941713219429 (0.00897027335348576)	0
IGHV3-49	0	0	0	0	0	0	2.65225710867104 (0.00908697393329197)	0	0	0
IGHV3-64	0	0	0	0	0	0	4.46266699650351 (0.0057429598061414)	8.50765927096693 (1.14328942807408e-07)	0	0
IGHV3-66	0	0	0	0	0	0	3.66061437950266 (0.00345490324225234)	0	0	0
IGHV3-7	0	0	0	0	0	0	2.4299050685782 (0.00876845870853537)	0	0	0
IGHV3-73	0	0	0	0	0	0	4.71529482724295 (0.000622193228779389)	0	0	0
IGHV3-74	0	0	0	0	0	0	3.75360330641559 (0.000168875667472134)	0	0	0
IGHV4-34	0	0	0	0	0	0	4.6477237502688 (2.28870871251624e-05)	0	0	0
IGHV4-39	0	0	0	0	0	0	2.87101138609082 (0.000996960202326313)	0	0	0
IGHV4-59	0	0	0	0	0	4.88480561116317 (0.00183819493402753)	5.05503807216674 (5.72762327604703e-07)	6.03040587356649 (0.000250559920361432)	0	0
IGHV5-51	0	0	0	0	0	0	3.34981047741281 (0.000130071623510695)	0	0	0
IGJ	0	0	0	0	0	0	2.61792976864303 (0.000322558530499142)	0	-5.9752515030481 (0.000967113643039563)	0
IGKC	0	0	0	0	0	0	2.47994998818062 (0.00059376464344754)	0	-5.78709030355232 (0.00562990909186804)	0
IGKV1-12	0	0	0	0	0	0	3.80977790742718 (0.000633028337939767)	0	0	0
IGKV1-16	0	0	0	0	0	0	3.50980812859768 (0.000858676380957509)	0	0	0
IGKV1-27	0	0	0	0	0	0	2.54407035463653 (0.00750674210124221)	0	0	0
IGKV1-39	0	0	0	0	0	0	3.47399796904708 (0.000995862103536436)	0	0	0
IGKV1-5	0	0	0	0	0	0	2.72626766778448 (0.000293332575659522)	0	-5.40165579729665 (0.00442179052498887)	0
IGKV1-6	0	0	0	0	0	0	2.71999185234532 (0.00269058602889593)	0	0	0
IGKV1-9	0	0	0	0	0	0	2.82073068104817 (0.00465875589948452)	0	0	0
IGKV1D-13	0	0	0	0	0	0	4.13358049254507 (0.00566214531574021)	0	0	0
IGKV1D-17	0	0	0	0	0	0	0	7.39217250514775 (0.00029122728349668)	0	0
IGKV2-24	0	0	0	0	0	0	4.37545115005598 (0.000775733598504988)	0	0	0
IGKV3-11	0	0	0	0	0	0	2.46619505889142 (0.00520055497640542)	0	0	0
IGKV3-15	0	0	0	0	0	0	2.2893738749754	0	0	0

							(0.00535339335657306)			
IGKV3-20	0	0	0	0	0	0	0	0	-5.07378495698096 (0.0089075717731292)	0
IGKV3D-20	0	0	0	0	0	0	4.69052079587594 (0.00329936739430737)	0	0	0
IGKV4-1	0	0	0	0	0	0	3.02239515090445 (4.56739150284582e-05)	0	0	0
IGLC2	0	0	0	0	0	0	2.9330039072191 (0.000213417231098486)	0	-6.32063204051368 (0.00977467569950884)	0
IGLC3	0	0	0	0	0	0	3.19605457918634 (0.000194970257669612)	0	0	0
IGLL5	0	0	0	0	0	0	3.3386112087247 (2.85676660615759e-05)	0	0	0
IGLON5	0	0	0	0	0	5.38831679237665 (0.00206432922914151)	0	0	0	0
IGLV1-40	0	0	0	0	0	0	3.38689849992537 (3.26898236712072e-05)	0	0	0
IGLV1-44	0	0	0	0	0	0	2.81206838340905 (0.000256473044369296)	0	0	0
IGLV1-47	0	0	0	0	0	0	3.92743892046926 (1.09413835660024e-05)	0	0	0
IGLV1-51	0	0	0	0	0	0	3.25496546300257 (0.000306897395118073)	0	0	0
IGLV2-11	0	0	0	0	0	0	2.82057331889296 (0.00381385683787948)	0	0	0
IGLV2-14	0	0	0	0	0	0	2.57227235862993 (0.00070207962426543)	0	0	0
IGLV2-23	0	0	0	0	0	0	3.12894514046579 (0.00166631474496271)	0	0	0
IGLV2-8	0	0	0	0	0	0	3.2129846130299 (0.00601259967891993)	0	0	0
IGLV3-1	0	0	0	0	0	0	3.82014185701557 (0.000464724164821329)	0	0	0
IGLV3-10	0	0	0	0	0	0	4.04149466620721 (0.000710260631703064)	0	0	0
IGLV3-19	0	0	0	0	0	0	3.45499226641782 (0.000790413256510315)	0	0	0
IGLV3-21	0	0	0	0	0	0	4.51550770789801 (1.85028128935722e-05)	4.6013837063366 (0.00215151264205263)	0	0
IGLV3-25	0	0	0	0	0	0	4.18213986368939 (4.26792827527176e-06)	0	0	0
IGLV3-27	0	0	0	0	0	0	3.78904917885875 (0.00939809585893364)	0	0	0
IGLV4-69	0	0	0	0	0	0	5.45701929616968 (1.67232063410427e-05)	0	0	0
IGLV5-45	0	0	0	0	0	0	4.30307771636349 (0.00279434594107308)	0	0	0
IGLV8-61	0	0	0	0	0	0	3.2540046976612 (0.00340424874583493)	0	0	0
IGLV9-49	0	0	0	0	0	0	4.67273435226802 (0.0006379710157742)	0	0	0
IGSF1	0	0	0	0	0	6.03974816035092 (0.00833856951076069)	0	0	4.85420658184196 (0.000455520528207952)	0
IGSF10	0	0	-3.22295120438755 (5.01980611146732e-09)	-3.55737525409708 (2.50852582123661e-05)	-2.47594657580914 (0.000743209110591985)	-2.12876810896778 (0.00808624968363674)	0	-5.67086019695951 (0.000426573990861982)	0	0
IGSF3	2.30791313900658 (1.56173843457638e-05)	0	2.02203606537963 (0.000160302793405888)	0	0	0	0	0	3.07162109230279 (1.10099678894379e-11)	0
IGSF5	0	0	0	0	0	0	0	0	6.20766728697855 (4.04140111134673e-06)	0
IGSF6	-2.85865801598867 (0.000246079104403695)	0	-2.45028583073792 (0.00143731411961004)	0	0	0	0	0	0	0
IGSF9	0	0	0	0	0	5.19888005820875 (0.000526728827215602)	0	0	0	0
IGSF9B	3.07185468692501 (6.61973971671781e-06)	0	3.55258588916699 (4.73535674196954e-06)	4.12219137828759 (0.000362047330391572)	0	0	0	0	0	0
IK	0	0	0	1.57948760934448 (0.00680223881384774)	0	0	0	0	0	0
IKZF1	-1.84079227034062 (0.00448502158760051)	0	-2.76921172324145 (6.56750142271878e-05)	0	0	0	0	0	0	0
IKZF2	0	0	-1.93141813179114 (0.00793408359697042)	0	0	0	0	0	0	0
IKZF3	0	0	-3.27359396282678 (0.000247343093200588)	0	0	0	0	0	0	0
IL10RA	-1.64771274888651 (0.00290017293468021)	0	-2.88559857765845 (1.84273279848928e-07)	0	0	0	0	0	0	0
IL10RB	0	0	-1.93254060709215 (0.00149912009412953)	0	0	0	0	0	0	0
IL12RB2	0	0	0	0	0	0	0	0	2.9326180335184 (0.000664558691068805)	0
IL13RA1	0	0	-1.90042544495246 (0.000342558878287559)	0	-2.09620615658314 (0.00932784543847135)	0	0	-2.53937294956216 (0.00941782313777767)	0	0
IL15RA	0	0	0	0	0	0	0	0	-1.58951274810748 (0.00948322038407914)	0
IL16	0	0	-1.5858784811605 (0.00833975200906164)	0	0	0	0	0	-1.61389711714939 (0.000177662749597018)	0
IL17RB	0	0	0	0	0	2.65590943605136 (0.00217624091048041)	0	0	0	0
IL17RC	0	0	0	0	0	2.17652592042624 (0.00097158106700021)	0	0	0	0
IL17RD	3.98220248211604 (3.12987562227402e-08)	0	3.12112987929615 (0.00272904798593878)	0	3.53935218337158 (0.00502260638739892)	3.92384172043941 (0.00582022289685787)	0	0	0	0
IL18	-2.11453084588053 (0.00498362429513943)	0	-3.18824959779718 (0.000178819462001187)	0	0	0	0	0	0	0
IL18R1	-2.69926875521085 (1.56808518314877e-05)	0	-2.77503106110225 (1.03993319910559e-05)	-4.92105550308543 (1.19158418525225e-05)	-2.83184603594703 (0.000646290712923416)	0	0	0	-1.87295795196352 (0.000100656844537671)	0
IL18RAP	0	0	-3.11244552986979 (0.00422318436976286)	-6.14886716413677 (0.00923927837220252)	0	0	0	0	-3.46264068542842 (0.000500930839881286)	0
IL1B	-4.08482397913589 (0.00174111719433803)	0	-3.62353107972552 (0.00910480466206813)	0	0	0	0	0	0	0
IL1R1	0	0	-2.23320551694148 (0.000107385765941308)	-2.01865654106333 (0.0028309894781257)	-1.57275769357514 (0.00503751491199533)	-1.77041341629965 (0.00239772873718415)	0	-3.1343099091488 (1.38521731367409e-05)	0	0
IL1R2	-4.09380568292546 (1.36031308433152e-08)	0	-5.10715705874997 (3.18005571564687e-12)	-2.87800822971512 (0.000646953058655519)	-3.11464842857149 (0.000254309837289629)	-4.2775835877516 (6.30577928589922e-06)	0	0	0	-2.68934117843269 (0.00100860777820311)
IL1RAP	0	0	0	0	0	0	0	0	1.94208969206544 (2.67452733012601e-09)	0
IL1RL1	-3.52219785066165 (0.000219133904614744)	0	-3.61289612033044 (1.09719814673102e-06)	-4.79474870582389 (2.29081239744258e-10)	-2.71797553536049 (6.60031446790159e-06)	0	0	-7.12940595660982 (8.67982317800539e-09)	0	-2.5266974205253 (0.000326684617310208)
IL1RN	-3.07705256050736 (0.000493652833918104)	0	0	0	0	3.580159954669 (0.000311716071909174)	0	0	0	0
IL20RB	0	0	0	0	0	0	0	0	0	4.17230774428358 (0.00191909686259187)
IL22RA1	0	0	0	0	0	4.4200206686276 (0.00242293996031542)	0	0	0	5.35555837973099 (0.00040298280702834)
IL2RA	0	0	-3.7658947817552 (0.00444868582803652)	0	0	0	0	0	0	0
IL2RB	0	0	0	0	0	0	0	0	3.39649272928739 (6.32640403887788e-06)	0
IL2RG	-2.43859228230415 (0.00259872153993292)	0	0	0	0	0	0	0	0	0
IL32	-2.2336975968753 (0.000443515604403773)	0	0	0	0	2.84059303449251 (2.77068822305988e-08)	0	0	-2.3687089863659 (0.000399938522641324)	0
IL36RN	0	0	0	0	0	0	0	0	5.15783516593025 (7.46302230813136e-06)	8.05314564677364 (1.55286088811466e-06)
IL3RA	0	0	0	0	0	0	1.56614425731078 (0.00157391464865077)	0	-2.7920502396724 (0.000121095390937008)	0
IL4R	0	0	-1.78988538772528 (0.000828563007585506)	0	0	0	0	0	-1.52956258535011 (0.000758139289465252)	0
IL6	0	0	0	0	0	0	0	-5.84444225874071 (0.00879120938162337)	-1.83834289683528 (0.00206025624599525)	2.40043151521717 (0.00189058316662238)
IL6R	0	0	0	0	0	0	0	0	-2.04493867288078 (3.84329809008048e-09)	0
IL6ST	0	-2.07215677557326 (0.00573921676280903)	0	0	0	-1.61974774776118 (0.00219772501209676)	0	-3.26479815193129 (5.12511359697302e-07)	0	0
IL7R	0	-5.81784935273351 (0.00913611600619783)	-4.85820599511704 (1.15439020811599e-11)	0	-2.57838656545229 (0.00286200707508871)	0	0	0	-4.12938026654091 (3.74519710717888e-08)	0
ILDR2	2.33189408113083 (0.00957253798656721)	0	0	0	0	5.58098801397781 (6.26180216361215e-06)	0	0	0	0
ILF2P2	0	0	0	0	0	6.16856019215855 (0.00609985852466005)	0	0	0	0
ILVBL	0	3.79218242584275 (0.000905847380838104)	2.00845492555284 (0.00207449187529914)	0	0	0	0	0	0	0
IMMT	0	2.14095888449772 (0.00510686513207656)	0	0	0	0	0	0	0	0
IMPDH1	0	0	0	-2.43081842133507 (0.008772768517506)	0	0	0	0	-1.6323387163844 (0.000492163672084902)	0
IMPG2	0	0	0	0	0	0	5.24205428997391 (0.00100394829434488)	0	0	0
INA	3.49816960917964 (0.00284985375674518)	0	0	0	0	0	0	0	0	0
INCENP	0	0	0	0	0	0	0	3.26490060819392 (9.52162467046469e-05)	0	0

INHA	0	0	0	0	0	0	0	0	5.71433366569116 (1.65628566194516e-07)	0
INHBA	0	0	-1.84223184097902 (0.00655623060195039)	0	0	0	0	0	3.45956053664587 (4.96961243091409e-08)	0
INHBB	0	0	2.51658484397583 (0.00623587907191079)	0	0	2.4943974127545 (0.00768911432956248)	0	0	0	4.90190103379705 (2.78320546877164e-07)
INHBC	0	0	0	0	0	7.13190182665234 (1.5964309966292e-06)	0	0	0	0
INHBE	0	0	0	0	0	8.64368509817383 (1.2499031531288e-11)	0	0	0	0
INMT	-2.6051848056643 (5.27470401681491e-07)	0	0	-2.92418685559303 (0.000929253712793503)	-2.67580516906242 (0.000452896705724508)	0	0	-4.36685996215996 (0.000198752671138483)	-2.77104613904951 (9.77930295730253e-09)	-6.29048109605066 (1.41146331116353e-06)
INPP5A	0	0	1.5830710886838 (0.00143616852048419)	0	0	0	0	0	0	0
INPP5F	0	2.96141174902074 (0.000526931239354418)	0	0	0	0	0	0	0	0
INPP5J	0	0	4.28226821374342 (0.000105867806095467)	0	4.53309742519177 (0.0054894637878445)	0	0	0	0	0
INPP5K	0	3.31713163166652 (0.00251750265743884)	0	0	0	0	0	0	0	0
INS-IGF2	0	0	0	0	0	6.40698742999093 (0.00288035660563019)	0	0	0	0
INSIG1	-1.92399012408845 (0.00201477657100217)	0	-1.77556916657933 (0.0046109693688272)	0	0	0	0	0	0	0
INSL4	0	0	0	0	0	0	0	0	5.71158549285706 (0.00431230102251264)	0
INSR	1.66381935554985 (5.14408501664702e-07)	0	2.32129275802315 (2.61265688799215e-11)	0	0	2.01639170299361 (0.000991547927231148)	0	0	2.03999603939227 (3.35487250867984e-06)	0
IP6K3	0	0	2.43495144454374 (0.000637223397200234)	0	0	0	0	0	0	0
IPO13	0	0	1.82610209710455 (0.000381793243506502)	0	0	0	0	0	0	0
IQCJ-SCHIP1	0	0	0	3.47210571114951 (0.00756045096278528)	0	0	0	0	0	0
IQGAP1	0	0	-1.67842345023073 (0.000277007100355848)	0	0	-2.09363921543711 (9.55207773155189e-05)	0	0	0	0
IQGAP2	0	0	-1.85329118059971 (0.00799083684832242)	0	0	0	0	0	0	0
IQGAP3	0	0	0	0	0	0	0	3.95222301706356 (0.000542388933670403)	0	0
IRAK1	0	0	0	0	-2.07123279799686 (0.00144887922634597)	0	0	0	-1.53267387311249 (0.000388142103712861)	0
IRAK1BP1	2.205105161534 (0.00621658743544529)	0	0	0	0	0	0	0	0	0
IRAK2	-2.65992503524927 (0.000508296789245059)	0	0	0	0	0	0	0	-2.30819081039609 (0.0026361000003861)	0
IRAK3	-2.27989903382419 (9.59697979245138e-07)	0	-2.16317613430465 (8.77253917020054e-08)	-2.03967125834388 (0.00557260900708668)	-2.35844292853865 (0.000398073931531701)	-1.85399244208611 (0.00152983815251141)	0	0	-1.83914056462687 (2.1352801743988e-06)	0
IRF1	0	0	0	0	0	0	0	0	0	-2.09246781214587 (0.0043430603573914)
IRF4	0	0	0	0	0	0	1.84624700791877 (0.00304300119776951)	0	0	0
IRF5	0	0	-3.01947339754209 (0.0028326309967907)	0	0	0	0	0	0	0
IRF6	-2.3567390442764 (0.0096396902014616)	0	0	0	0	0	0	0	1.69846074484516 (6.54720362777598e-05)	2.80129586544421 (0.00307164433905967)
IRF8	0	0	-2.75931325219136 (1.36331741634983e-05)	0	0	0	0	0	0	0
IRS1	0	0	0	2.14020184534006 (0.00950607714825775)	0	0	0	0	0	0
IRX1	0	0	0	0	3.74119877282868 (0.00842910386654907)	0	0	0	0	0
IRX2	-5.36894769129118 (1.1003938477976e-06)	0	-2.64524625434442 (0.000813425122155593)	-6.63966673363382 (0.00319682411366651)	0	0	0	0	0	0
IRX3	-4.46378932598889 (0.000186067960474058)	0	3.07739605107847 (3.15087405279706e-07)	0	0	0	0	0	-3.66159170411944 (8.30952094909108e-06)	2.64296236539274 (0.00611745984357779)
IRX4	0	0	5.22743536434185 (0.00019225638867184)	0	0	0	0	0	0	0
IRX5	0	0	2.86804703403479 (0.00192896810142954)	0	0	0	0	0	0	0
IRX6	0	0	4.7779817694291 (3.59319678099195e-05)	0	0	0	0	0	0	0
ISG15	0	0	0	0	0	0	2.16850506845723 (0.00303493487912483)	0	2.75211781789036 (0.000209402975673594)	0
ISG20	-2.29369008052119 (0.000871105999270804)	0	0	0	0	0	0	0	0	0
ISL1	0	0	0	0	0	0	0	0	4.4027968339702 (0.000286138934317634)	0
ISLR	1.64499099461362 (0.00124164388876501)	0	0	0	1.80127238183876 (0.00980833280931263)	0	0	0	0	0
ISM2	0	0	0	0	7.5070014335697 (1.12886607701164e-06)	0	0	0	11.6302326165399 (1.71118590040655e-30)	0
ISOC2	0	0	0	0	0	0	0	0	-2.35592627274986 (0.00368177974243017)	0
ISPD	0	0	0	0	0	0	0	0	1.57175709949226 (0.00296699302112617)	0
ISX	0	0	0	0	6.24434293510046 (0.0075784156376657)	6.83616064796014 (0.00210048989075515)	0	0	0	0
ISYNA1	0	0	0	0	0	0	0	0	1.82238425369407 (0.00671040473014055)	0
ITFG3	0	0	1.59710560150769 (0.00175491834116804)	0	0	0	0	0	0	0
ITGA1	0	0	0	0	0	0	0	-4.25689299356931 (7.3285188666879e-07)	0	0
ITGA10	0	0	0	0	0	0	0	0	-2.08839812833842 (0.00119881669200269)	0
ITGA11	0	0	2.56766999029626 (4.68213937477358e-05)	0	2.50485575801337 (0.00444188699057421)	2.82671790558414 (0.00645409445287942)	0	0	2.41038943308792 (2.48318674716509e-05)	0
ITGA2	-2.91620212059933 (1.80307359383194e-07)	0	-4.43124462108557 (9.30146858692819e-16)	-4.00281498170204 (3.52712888509766e-06)	-3.51056950514521 (1.18345105259011e-05)	-3.18126965725099 (2.76759663339648e-06)	0	-5.40316158504472 (2.17661176921409e-05)	-2.19193895048881 (2.35575512032469e-07)	0
ITGA2B	-3.82953124577498 (0.00342982035311845)	0	0	0	0	0	0	5.26720194176741 (1.18633361872184e-08)	0	0
ITGA3	-2.37642584241609 (9.63266455744872e-10)	0	-2.54638596910746 (5.84965804459868e-10)	-2.75032565658811 (0.000206575427783224)	0	-2.55099381867931 (7.69033492149187e-06)	0	-5.5004892234635 (9.25537436477479e-06)	-1.63708014252748 (1.42278648198267e-07)	-1.86162214727812 (0.00917532878004978)
ITGA4	-1.60190557162973 (0.00640575450597546)	0	-2.51590835172725 (0.00111712897127025)	0	0	0	0	3.17500354739786 (0.000187406826814492)	0	0
ITGA5	0	0	0	0	0	0	0	-3.23524372775758 (1.334175558747e-05)	0	0
ITGA6	0	0	0	0	0	0	0	-2.69459042564817 (0.00376514226664366)	0	0
ITGA7	1.90766591405853 (5.31249071260191e-07)	0	3.78168511270792 (7.49655155119067e-21)	2.44117411874526 (0.000786320032398011)	0	0	0	0	0	0
ITGA8	-1.76427941976881 (0.000583843311981772)	0	-2.5480908517337 (6.97322668395148e-06)	-3.5126479278877 (0.000345056340344848)	0	-2.65138854698037 (0.000420911787083514)	0	-7.48181131136918 (0.000654704286670301)	-8.33028509080307 (1.95168052957851e-20)	0
ITGA9	0	0	2.08385585224886 (6.68590343628769e-09)	0	0	0	0	0	0	0
ITGA9-AS1	0	0	0	0	0	3.01632847172989 (0.00732180620734473)	0	0	0	0
ITGAD	0	0	0	0	0	4.84255075836932 (0.00952928152699508)	0	0	0	0
ITGAL	-2.1703077407483 (0.00568827190505974)	0	0	-3.93246940218647 (0.00824059421839214)	0	0	0	0	0	0
ITGAM	0	0	-1.78998116194742 (0.00133981019791081)	0	-2.76063316793316 (0.00109386883079017)	0	0	2.07987246993568 (0.00125645153741856)	0	0
ITGAV	0	0	0	0	0	0	0	-3.42152552540277 (1.85663469952659e-05)	0	0
ITGAX	-3.97272793374059 (6.51103740573299e-10)	0	-4.67681257214126 (1.84351434846513e-16)	-4.08062551507032 (1.89849414562338e-06)	-2.94566505080208 (0.000313380671943006)	0	0	0	-3.1228060445421 (4.96171792867484e-08)	-3.73179445885385 (4.94019293744965e-06)
ITGB1BP2	0	0	4.15033276832951 (1.1541285677214e-06)	0	0	0	0	0	0	0
ITGB2	-1.70258683872405 (0.00960680551575257)	0	-2.11865776242854 (0.00077245272462214)	0	0	0	0	0	0	0
ITGB3BP	0	0	0	0	0	0	0	2.89497778785007 (0.00927744189301322)	0	0
ITGB4	1.85224504153302 (0.000133511055567359)	0	0	0	0	0	0	0	3.27584761696293 (6.7740750142385e-18)	3.82546258429299 (9.36626607721458e-06)
ITGB6	-6.05374680303948 (1.71830081617615e-07)	0	-4.64859091684137 (3.88684616862203e-07)	-3.74959003409439 (0.0089633201131086)	0	0	0	-6.13426258940371 (0.00743953684339609)	0	-7.47427914381993 (0.00105214831977633)
ITGB8	0	0	-2.17417674058163 (0.00177450005784125)	0	0	0	0	-6.39120865222506 (0.0044220350621805)	3.36506289700818 (0.00082982101572655)	0
ITGBL1	-7.53387569440111 (4.79168942726843e-10)	0	0	-4.9156918365947 (0.00489503024797749)	0	0	0	0	0	0
ITIH1	0	0	0	0	0	10.9089042210145 (9.46828944438743e-19)	0	0	0	0
ITIH2	0	0	0	0	0	10.2877118155335 (1.28196226762349e-18)	0	0	0	0
ITIH3	0	0	0	0	0	8.47377318712415 (2.45471212954192e-27)	0	0	0	0
ITIH4	0	0	0	0	0	8.56628899763752 (2.02690422324629e-09)	0	0	0	0
ITIH5	2.65121573963925	0	0	2.80994075610704	0	0	0	0	3.10782734018805	2.52027483247539

	(3.86803562623045e-10)			(0.000383287646704292)					(1.07569542411866e-14)	(0.00167087646696605)
ITK	0	0	-3.86589891030002 (0.000564974780817402)	0	0	0	0	0	0	0
ITLN1	4.67171359718608 (0.00224615074638839)	0	0	0	0	0	0	0	0	0
ITM2A	0	0	1.9277151716011 (0.00770865870950915)	0	0	0	0	0	0	0
ITM2B	0	0	0	0	0	0	0	-2.24457049263027 (0.00591433190067423)	1.51304233278446 (0.000116496406731605)	0
ITM2C	3.60128616125792 (2.09072508956828e-15)	0	1.96212964392441 (6.4277240982755e-05)	3.93599457076948 (1.07802251508995e-06)	2.94510191390632 (2.14475517116072e-06)	0	1.8170320052775 (2.65098790920622e-05)	2.91673647174458 (0.00212663547148612)	1.85016001626352 (0.00366962565639281)	0
ITPK1	0	0	1.63580075895139 (0.000328909865470933)	0	0	0	0	0	0	0
ITPK1-AS1	0	0	0	0	0	3.27305955085486 (0.00172409325673914)	0	0	0	0
ITPKB	0	0	0	2.10665171298883 (0.00125989231610316)	0	0	0	0	0	0
ITPKC	0	0	0	0	0	0	0	-4.36205313730439 (0.00704094837746127)	0	0
ITPR3	-2.01822844275557 (0.000149698277764458)	0	0	0	0	0	0	0	-1.95012279218106 (7.91223562323729e-06)	0
ITPRIP	-2.18979763616976 (4.19640268845815e-07)	0	0	0	0	0	0	0	0	0
ITPRIPL2	0	0	0	0	0	0	0	-2.62148529987451 (0.00064275522307253)	0	0
IYD	0	0	0	0	4.91124663070252 (0.00165790752599683)	5.62361424594009 (5.1801186090781e-06)	3.7195235717463 (0.00732728224514759)	0	0	0
JADE3	0	0	0	0	0	0	0	0	2.48673866877474 (8.45927182038710e-10)	0
JAG1	0	0	0	0	0	0	0	-5.67708654288116 (0.000452716249017066)	0	0
JAG2	0	0	2.51914807059959 (4.73194299353411e-05)	0	0	0	0	0	0	0
JAGN1	1.56168507374709 (0.00508262968975144)	0	0	0	0	0	0	0	0	0
JAK3	-1.8352574116564 (0.000764350366792511)	0	0	-4.48347017650701 (0.000729200323378746)	0	0	0	0	0	0
JAM2	0	0	0	0	0	0	0	-5.89324403760286 (0.00951745196378089)	2.71371448490997 (5.17134347107875e-10)	0
JARID2	0	0	0	0	0	0	0	2.86793888411425 (5.0788378498544e-06)	0	0
JHDM1D-AS1	0	0	0	0	0	0	0	0	0	3.56221469781046 (0.000409529808382412)
JMY	1.51794742508616 (7.37464106864903e-05)	0	0	0	0	0	0	0	0	0
JOSD1	0	0	0	0	0	0	0	-2.39924361913668 (0.0064111033502048)	0	0
JPH1	0	0	2.46282789801544 (2.09264277745277e-05)	0	0	0	0	0	0	0
JPH2	3.13060076418234 (9.06039590276167e-08)	0	5.18210814775588 (7.15402571856869e-14)	3.12956420723271 (0.00214797432332666)	0	0	0	0	0	0
JPH3	4.30616990783635 (0.00639704929502358)	0	0	0	0	7.5462341041073 (7.34654051358129e-05)	0	0	0	0
JUN	-1.79043913464936 (0.000152133377056475)	0	0	0	0	0	0	-2.59504502311756 (0.00149166872588127)	0	0
JUNB	-1.65857572720654 (0.000133613614636858)	0	0	0	0	0	0	0	0	0
JUP	0	0	1.57192103849352 (0.000134462190942136)	0	0	0	0	-4.46860325224907 (0.0061171500443223)	0	2.65596033472311 (0.000103952048661094)
KAL1	-4.08897949621519 (0.00013069907935558)	0	0	-6.01447407835871 (0.00982580232307443)	0	0	1.63380643345725 (0.00542590143643528)	0	0	0
KANK1	0	0	1.7188132395015 (1.44132141721023e-05)	0	0	2.83389361423907 (3.07701794331418e-06)	0	-6.51461817824892 (0.00372862517520173)	1.56322354497967 (1.28198648207959e-05)	0
KANK2	1.66716706947983 (1.46214698687684e-05)	-5.57460393735243 (0.00949607768896242)	0	0	0	0	0	0	0	0
KANK3	0	4.13946702113015 (0.000179697741162152)	0	0	0	0	0	0	0	0
KANK4	0	0	0	0	0	0	0	0	3.79486101114664 (7.86107468674699e-15)	0
KAT6B	0	0	0	2.01657677759124 (0.00223645458894553)	1.71422808133464 (0.00134267900082349)	0	0	0	0	0
KAZALD1	0	0	0	0	0	5.28228783448225 (0.00020798087765903)	0	0	0	0
KAZN	0	0	0	0	2.55762869301752 (6.3707426790119e-05)	0	0	0	0	0
KB-1125A3.12	0	0	0	0	0	0	0	0	3.53378586005065 (0.00166735078558703)	0
KB-1517D11.4	0	0	0	0	4.40070582583984 (0.00779995259640371)	4.58372615682385 (0.00921342732942754)	0	0	0	0
KB-226F1.2	0	0	0	0	0	5.14900437368558 (0.0026611416215786)	0	0	0	0
KBTBD11	0	0	0	0	0	0	0	0	-3.19929803845673 (5.74356500077463e-06)	0
KBTBD12	0	0	4.52823252145414 (6.62897511875929e-07)	0	0	0	0	0	0	0
KC6	0	0	0	0	0	0	0	0	4.1327907741918 (0.00754974269416504)	0
KCNA2	4.2541727854795 (0.002733879710716)	0	0	0	6.56631018310623 (0.000751194117790309)	6.29777500001923 (0.0063330606845031)	0	0	0	0
KCNAB2	0	0	0	0	0	0	0	0	-1.79763086285037 (0.000140321403197477)	0
KCNAB3	0	0	0	0	0	4.3164933600502 (0.00747280555627311)	0	0	0	0
KCNB1	6.11814219617715 (6.54251321022298e-22)	0	2.5545577859845 (0.003967647839644)	4.71724171766163 (0.000430770188520066)	0	5.99022696440348 (4.10927272206472e-11)	0	0	0	0
KCNB2	0	0	0	0	6.48222785639122 (0.00467673319314815)	6.0645547329706 (0.00760120533172173)	0	0	0	0
KCNC1	0	0	0	0	0	6.65802911812241 (3.75059186672413e-07)	0	0	0	0
KCNC3	-2.75380415611372 (0.00897453146675213)	0	0	0	0	0	0	0	0	0
KCNC4	0	0	0	0	1.71097049483636 (0.0035969533013665)	2.51902054098642 (0.00939185843756529)	0	0	0	0
KCND2	0	0	0	0	6.30139135490905 (0.0080350818762734)	0	4.51314515425968 (0.00801447015637531)	0	0	0
KCND3	1.55930923835192 (0.000508187580049346)	0	0	0	0	0	0	-5.88757910731325 (0.00805471107395793)	-2.25440018859569 (0.000200481943324755)	0
KCNE1	-3.59974688898402 (4.1312409054247e-06)	0	-2.58719066993225 (0.00244392986254245)	-4.46752800403488 (0.00925445978126114)	0	0	0	0	-3.35997792053046 (8.51495093270093e-06)	-4.74908375897396 (0.00563831764857101)
KCNE4	0	0	0	0	0	2.27748583402165 (0.00440726971069869)	0	0	0	0
KCNG1	0	0	0	0	0	0	0	0	3.65020851392248 (0.000635502864404822)	0
KCNG2	0	0	0	0	0	4.35232228178456 (0.000768963947958457)	0	0	0	0
KCNH1	0	0	0	0	0	7.06157958536341 (0.00823408752216087)	0	0	4.90917604478048 (0.00152728626743929)	0
KCNH2	5.94269691982818 (1.4864129681403e-15)	0	6.70885523086818 (2.60675626412788e-15)	5.39866231242548 (5.23412545503517e-05)	0	0	0	7.8165721866403 (5.92694148312757e-15)	0	0
KCNH5	0	0	0	0	0	7.0048238026879 (0.00773068985945733)	0	0	0	0
KCNIP2	0	0	5.35815547453492 (2.95510700025292e-17)	0	0	0	0	0	0	4.34985800918646 (0.000561940131679984)
KCNIP4	5.45242869244288 (1.22676642160578e-06)	0	0	0	0	0	0	0	0	0
KCNJ1	0	0	0	0	5.79476439755603 (1.39584894791066e-05)	0	0	0	0	0
KCNJ10	0	0	0	0	6.54807333901858 (0.00474950207339113)	6.92921135976098 (0.000748124745322171)	0	0	0	0
KCNJ11	0	0	3.90172179429685 (0.000852139312795806)	0	0	0	0	0	0	0
KCNJ12	0	0	2.31290356963993 (0.00745920394398979)	0	0	0	0	0	0	0
KCNJ15	-4.1510961039366 (3.89521839721763e-08)	0	-3.64944450290961 (1.54844275409517e-06)	-5.08681169370144 (0.00256264620294449)	0	0	0	0	0	0
KCNJ16	0	0	0	0	7.44734540035683 (3.71598990269029e-09)	0	0	0	0	0
KCNJ2	0	4.01306963702648 (0.000564881180871822)	1.60201192548009 (0.00685885030723046)	0	0	0	0	0	2.56752606900667 (5.54231704179511e-09)	0
KCNJ2-AS1	0	0	0	0	0	5.46653271294761 (0.0091926260484846)	0	0	0	0
KCNJ4	0	0	7.36554142035215 (0.00336171306630739)	0	0	0	0	0	0	0
KCNJ5	0	0	0	0	0	3.70957192559622 (0.00302427571059568)	0	0	2.08861615578075 (0.00204770085764876)	0
KCNJ6	0	0	0	0	6.15201733371194 (0.000169773255324781)	8.03365608991556 (4.94275070047723e-08)	0	0	0	0
KCNJ8	1.70323951584737 (0.00117616992623685)	0	2.33659410732206 (1.19260046313124e-05)	0	0	1.92995815222092 (0.00776996386107714)	0	0	0	0
KCNJ9	0	0	0	0	5.83004520490158 (0.00374383828643843)	5.99196961915596 (0.00253559498386935)	0	0	0	0

KCNK2	5.96376928642652 (7.58542824001867e-07)	0	0	0	0	0	0	0	0	0
KCNK3	0	0	-2.5883525275491 (0.00552181927157376)	0	0	0	0	-6.46492164292146 (0.00436128770617642)	0	0
KCNK5	0	0	0	0	0	3.09630775576939 (0.00464235809850452)	0	0	0	0
KCNK9	0	0	0	0	6.24434293510046 (0.0075784156376657)	0	0	0	0	0
KCNMA1	3.82019652487l2 (6.35914759994279e-22)	0	0	4.23565749590852 (1.08010597341348e-09)	0	0	0	-6.39433941879958 (0.00376017672751901)	0	0
KCNMA1-AS1	4.57531553798537 (5.42256815092798e-05)	0	0	0	0	6.22797544093731 (0.000100233051002379)	0	0	0	0
KCNMB1	4.1040888838697 (6.58745024361198e-15)	0	0	4.41206211764591 (8.58771485682221e-06)	0	0	0	0	0	0
KCNMB3	0	0	0	0	0	3.61825068515053 (0.00368893916301617)	0	0	0	0
KCNN2	0	0	0	0	0	0	0	0	-4.13128463198986 (0.00926306322742542)	0
KCNN3	4.44052349423761 (2.14273423108647e-13)	0	0	3.94607394595132 (1.58547090088139e-05)	2.6614195592794 (0.00166340496617465)	3.00321955024643 (0.00155239919915066)	1.80546279027346 (0.00155745136676628)	0	0	0
KCNN4	0	0	0	0	0	0	0	0	2.98005190389715 (9.99516898587231e-06)	0
KCNQ1	0	0	2.10701979261025 (1.60396247395941e-05)	0	0	0	0	0	0	0
KCNQ2	0	0	0	0	0	7.5361175899131 (0.00308368344718617)	0	0	0	0
KCNQ3	0	0	-3.88082600541776 (4.73044044158915e-05)	0	0	0	0	0	0	0
KCNQ4	4.42048821967368 (4.50010451535465e-08)	0	0	0	0	0	0	0	0	0
KCNQ5	4.11071952311428 (0.00354645147703374)	0	0	0	0	6.40350891249126 (0.00167409385104541)	0	0	0	0
KCNS1	0	0	0	0	0	7.32254860298049 (0.000683937380109816)	5.3052103275105 (0.00342368366096602)	0	0	0
KCNS3	-2.28284300442355 (0.000107396876634278)	0	-3.88936779293628 (1.5491587121541e-05)	0	0	0	0	0	0	0
KCNT1	0	0	0	0	0	5.41355192839855 (0.0067294207125871)	0	0	0	0
KCNT2	0	0	0	0	0	2.33790909154868 (0.000992366632794081)	0	0	0	0
KCNV1	0	0	0	0	0	6.51183716833542 (7.0725257399106e-05)	0	0	0	0
KCP	0	0	0	0	5.88169450857704 (3.59283348353708e-06)	0	0	0	0	0
KCTD1	0	0	0	0	0	0	0	0	2.06349923115514 (0.000161916560433833)	0
KCTD10	0	0	0	0	0	0	0	-4.46025760639871 (0.000221872646713612)	0	0
KCTD11	0	0	0	0	2.03866544237859 (0.00981155595739646)	0	0	0	0	0
KCTD12	0	-6.22022376528866 (0.0043457423844187)	0	0	0	-1.8099113237073 (0.00109482111190263)	0	-3.65109096214797 (4.75529609146907e-05)	0	0
KCTD16	0	0	0	0	3.53016716833344 (0.00397730395265782)	4.60029745184505 (0.000665498851657399)	0	0	0	0
KCTD17	0	4.22161183736084 (0.00135287058776855)	1.6955649392755 (0.00789824808333323)	0	0	0	0	0	0	0
KCTD2	0	0	1.62474064613656 (0.00407427142311454)	0	0	0	0	0	0	0
KCTD21-AS1	0	0	0	0	0	4.46390642579692 (0.00192929284781629)	0	0	0	0
KCTD8	0	0	0	0	0	6.15551698202792 (0.00281460418719922)	0	0	0	0
KDM4A	0	0	0	-2.95954037779442 (0.00220975580550701)	0	0	0	0	0	0
KDR	0	0	0	0	0	-1.91229873745814 (0.00334387199604727)	0	-3.45798594435881 (0.00102382917789099)	1.62317390279707 (3.0417856456814e-05)	0
KEL	0	0	0	0	0	5.94991402519041 (0.00919656742815934)	0	9.00022212042411 (5.01888756908888e-13)	0	0
KHK	2.66883138449137 (0.00756283140538527)	0	0	0	0	5.94638144545396 (6.76832618900977e-08)	0	0	0	0
KIAA0125	0	0	0	0	0	4.54374034876193 (0.000924853178822655)	2.73964291430358 (0.00292968922163064)	4.63044804500938 (0.00138591163266653)	0	0
KIAA0408	3.52374061948874 (0.00580482991368084)	0	0	0	0	0	0	0	0	0
KIAA0895	1.83932420656116 (0.00474332271084827)	0	0	0	0	2.71694768362675 (0.00506301710645558)	0	0	0	0
KIAA1024	0	0	0	0	0	3.89539781977776 (0.00839085814126075)	0	0	0	0
KIAA1045	0	0	0	0	0	6.95251848045635 (7.45747934214881e-05)	0	0	0	0
KIAA1161	0	0	1.87297940829149 (0.00444625108631839)	0	0	2.90560151282028 (0.000173063939672525)	0	0	2.69568673006881 (0.000456022583569967)	0
KIAA1211	0	0	0	0	0	0	0	3.46306494696318 (0.00106384000763562)	0	0
KIAA1217	0	0	0	0	0	0	0	0	1.84676298134011 (1.71714774092834e-15)	0
KIAA1244	0	0	0	0	0	0	0	-6.88578918259734 (0.00138801537877686)	0	0
KIAA1324	0	0	0	0	0	0	2.67943595495045 (0.00153922788199148)	0	0	0
KIAA1456	0	0	0	0	0	1.76813050204662 (0.00306902824049389)	0	0	0	0
KIAA1462	0	0	0	0	0	-3.17241457175278 (2.20040256038722e-05)	0	-6.98076376929197 (4.78264250353431e-05)	0	0
KIAA1549	0	3.7430999315608 (0.00110388279450457)	0	0	0	0	0	0	0	0
KIAA1549L	0	0	0	0	0	5.06734646728529 (0.00188708340492833)	0	0	0	0
KIAA1598	0	0	-2.19621767777332 (0.000133460928996786)	0	0	0	0	0	0	0
KIAA1644	5.99554142181803 (1.06760272115197e-11)	0	0	6.87827899912635 (2.00701645023644e-08)	0	5.60728455786375 (0.000338316274093744)	0	0	4.21454201027758 (0.000906609610706669)	0
KIAA1671	0	0	0	0	0	0	0	-7.58147411808906 (0.000440490957761474)	0	0
KIAA1755	0	0	0	0	3.62330351231912 (0.0051042520579589)	0	0	0	0	0
KIAA1919	0	0	0	0	0	0	0	0	1.63319046870768 (0.000288945107218962)	0
KIAA2013	0	0	0	0	0	0	0	0	0	-2.8828944208866 (0.00705088138250479)
KIAA2022	4.66681640005193 (3.24509911858204e-08)	0	0	4.83547787182676 (0.00294790661000665)	0	4.45044044993509 (0.00749410306058306)	0	0	0	0
KIDINS220	0	-5.72304054053372 (0.00696473563130871)	0	0	0	-1.5431025365202 (7.12875644815954e-05)	0	0	0	0
KIF11	-2.95776187988412 (0.00633731535434893)	0	0	0	0	0	0	4.60597550921067 (6.64678584979364e-07)	0	0
KIF12	0	0	0	0	6.92238648867573 (2.45701241237244e-06)	5.44675917898481 (0.00797621869122314)	0	0	0	0
KIF14	0	0	0	0	0	0	0	3.82782863405866 (0.00132601960517779)	0	0
KIF15	0	0	0	0	0	0	0	5.0205249740296 (2.7944087274086e-06)	0	0
KIF18A	0	0	0	0	0	0	0	4.05690850808961 (0.00100144336336031)	0	0
KIF18B	0	0	0	0	0	0	0	4.48935803360846 (0.000184762403089891)	0	0
KIF1A	6.97365362979474 (1.95339566896708e-09)	0	0	7.68716032593834 (1.02397032143482e-06)	6.64453193007419 (7.21524132296146e-06)	6.95113879261675 (0.000459352149893314)	5.4204333133361 (0.00533860524345387)	0	0	0
KIF1C	0	0	2.66666713660231 (1.54148919334222e-09)	0	0	0	0	-2.61698678035014 (0.00214244050128552)	-1.69445718018505 (9.15024130448844e-06)	0
KIF20A	-3.62023948143322 (0.00615153632679734)	0	0	0	0	0	0	0	0	0
KIF20B	-1.90034341437159 (0.000775868428045724)	0	0	0	0	0	0	2.94643882024541 (0.000196630928011839)	0	0
KIF21A	0	0	1.84925340516871 (5.82711818774885e-08)	0	0	0	0	0	0	0
KIF21B	-2.27432153752824 (0.00299874721917091)	0	0	0	0	0	0	0	-1.82699441014838 (0.00627655095776142)	0
KIF22	0	0	0	0	0	0	0	4.18893129931609 (1.19125322274763e-05)	0	0
KIF23	0	0	0	0	0	0	0	4.20254076145494 (8.52920734571964e-05)	0	0
KIF26A	0	0	3.03422944258468 (2.47244448193646e-05)	0	0	0	0	0	0	0
KIF26B	-1.87661369056582 (0.00371011948702539)	0	-1.85975217097351 (0.00885769274640847)	-7.48927832033666 (0.00101819197338311)	0	0	0	-6.43315247801123 (0.00496033926358674)	0	-4.32994220174253 (0.0024954238124712)
KIF2C	0	0	0	0	0	0	0	6.60620838897329 (3.18565361811847e-09)	0	0
KIF3C	1.58443805872841 (0.00350209535044693)	0	0	0	0	0	0	0	0	0
KIF4A	0	0	0	0	0	0	0	4.80684259483565	0	0

								(0.000969663312236986)		
KIF4B	0	0	0	0	0	6.58574536051647 (0.000388780117580966)	0	0	0	0
KIF5A	4.48243227534657 (3.0256997633774e-09)	0	0	5.24019856017638 (2.2163814304379e-05)	0	0	0	0	0	0
KIF5B	0	-6.51162035326897 (0.00213014853639555)	0	0	0	-1.66568625950329 (0.00161957338433852)	0	0	0	0
KIF5C	5.24669997162847 (5.84745132564432e-12)	0	0	5.49066796604731 (1.65748836482405e-05)	3.79562158364259 (0.00470318201818172)	5.38963008368851 (0.000222629542944014)	3.46539047546383 (0.00194671629172466)	0	0	0
KIF6	0	0	0	0	0	4.4214665685654 (0.00274733011563444)	0	0	0	0
KIF9-AS1	0	0	0	0	0	5.12762737204346 (0.00117811689915288)	0	0	0	0
KIFAP3	0	0	1.68168157068618 (0.000250329131664111)	0	0	0	0	0	0	0
KIFC1	0	0	0	0	0	0	0	4.90258921358557 (4.66755028067453e-06)	0	0
KIFC3	-1.95500740849307 (0.00102648189869981)	0	0	0	0	0	0	0	0	0
KIR2DL3	0	0	0	0	0	6.35947680596301 (0.00421653283516686)	0	0	0	0
KIR3DL3	0	0	0	0	0	7.28272757431076 (0.0048656497259573)	0	0	0	0
KIR3DX1	0	0	0	0	0	6.77306146470912 (0.00313945115989864)	0	0	0	0
KIRREL	0	0	-1.5662971485217 (8.26245399538631e-05)	0	0	0	0	-5.51133624531933 (0.000586222853823444)	0	0
KISS1	0	0	0	0	0	0	0	0	9.01928973847689 (0.000569043465196943)	0
KIT	3.25951030335941 (1.88280307629364e-07)	0	0	3.28543949494821 (0.00370142572000516)	0	0	0	0	0	0
KITLG	0	0	-2.01800748389275 (0.00201938506535084)	0	-2.25826403399093 (0.000731880964320043)	-3.7838172553933 (7.09067270074935e-08)	0	-5.0464962658317 (5.32295283207501e-05)	-2.16974424094197 (4.38449978843441e-06)	-2.21465812051459 (0.00290003838367476)
KL	0	0	0	0	0	0	1.8211856441392 (0.00310229760002527)	0	3.88294884251054 (6.85969208315298e-06)	0
KLB	0	0	0	0	0	5.9361051563442 (1.5870755856771e-06)	3.46700811083738 (0.00440056774461105)	0	0	0
KLC3	0	0	0	0	0	0	0	0	0	5.55031668356036 (0.0014591159477201)
KLC4	0	0	0	0	0	2.40776491174493 (0.000282692583663319)	0	0	0	0
KLF1	0	0	0	0	0	0	0	7.9116688694142 (2.81246070048406e-08)	0	0
KLF12	1.78738190399685 (0.00140780388773197)	0	0	0	0	0	0	0	0	0
KLF13	0	0	0	0	0	0	0	0	-1.50425947235007 (1.67885845188072e-08)	0
KLF15	0	0	2.59320659368186 (3.61758409293229e-05)	0	0	2.10101466070117 (0.00178827696355576)	0	0	0	0
KLF17	0	0	0	0	0	5.6293855698785 (0.00705473799497917)	0	0	0	0
KLF2	-1.88806603266048 (0.000473362632538315)	0	0	-2.71371186911221 (0.00695805145016996)	0	0	0	-3.46036025935352 (0.00961498746958656)	0	0
KLF3-AS1	0	0	0	0	0	4.98479727162879 (0.000784161353260495)	0	0	0	0
KLF4	0	0	0	0	-2.16399762680459 (0.0085852082059259)	0	0	0	0	1.9936155283673 (0.00471906309524398)
KLF4P1	0	0	0	0	3.88229856133829 (0.00723390377285611)	0	0	0	0	0
KLF5	0	0	-1.85709316836478 (0.00332551997349768)	0	0	0	0	0	1.62014612452834 (2.09223476681132e-05)	3.0064605832919 (7.12611878247933e-06)
KLF6	-2.06572661286943 (1.6639529174223e-08)	0	-1.71438622342362 (1.68302965813211e-09)	0	-1.87470129929439 (0.000162374985180285)	-1.61648333828261 (0.00019494435706556)	0	0	0	0
KLF9	0	0	-1.54684280999512 (0.000571579752543465)	0	0	-2.02565796307027 (9.52351956865302e-06)	0	-3.37657858389065 (2.8342320198834e-05)	-1.68871656193848 (0.00162380359535429)	0
KLHDC3	0	0	2.19758091425099 (0.000692310384445278)	0	0	0	0	0	0	0
KLHDC7A	0	0	0	0	0	4.28268007158785 (0.00859477923394908)	0	0	0	0
KLHDC8A	0	0	0	0	0	0	0	7.71410411188126 (4.41825250843527e-05)	0	0
KLHDC8B	0	0	3.2624464106866 (4.08610216679976e-09)	0	0	0	0	0	0	0
KLHL13	4.63937986315363 (3.50547632631456e-09)	0	0	0	0	0	0	0	0	0
KLHL14	0	0	0	0	5.26807456457017 (0.000132167493620556)	5.64697368799224 (0.000176746958828445)	0	0	0	0
KLHL2	0	0	-1.72391477910181 (0.000793576038950661)	0	0	0	0	0	-1.60151358948653 (0.0002524844921039)	0
KLHL23	2.16270994556998 (3.23163521865217e-05)	0	0	0	0	0	0	0	0	0
KLHL25	0	0	0	0	0	2.41011581745892 (0.0078402219625129)	0	0	-2.18005891094359 (0.00906890595586145)	0
KLHL29	0	0	0	0	0	2.85347779872391 (0.00345442765014709)	0	0	0	0
KLHL3	0	0	3.13000550244157 (7.34205443422149e-08)	0	2.50279663297422 (0.00138100306160801)	3.18971851653126 (0.00126531431787073)	0	0	0	0
KLHL31	0	0	8.69218479731612 (8.91701791959225e-18)	0	0	0	0	0	2.87750241631765 (0.00705068113988356)	0
KLHL38	0	0	6.55172153022825 (3.08959245838985e-06)	0	0	6.14036515488973 (0.00894254957457382)	0	0	0	0
KLHL41	0	0	6.39188063416789 (1.18046187699227e-14)	0	0	0	0	0	0	0
KLHL42	1.80650389042723 (1.26415948410574e-05)	0	0	0	0	0	0	0	0	0
KLHL6	-2.83147983387674 (0.000316770529735437)	0	-2.3577425812776 (0.00012408973567242)	0	0	0	0	0	-1.82239571334126 (4.73771191164812e-05)	-2.81778951490842 (0.00457570807560458)
KLHL7	0	0	1.79673748235407 (2.00460022703818e-05)	0	0	0	0	0	0	0
KLK1	0	0	0	0	0	0	0	0	0	6.12267968953439 (0.00482117969439326)
KLK10	0	0	0	0	0	0	2.81872806670808 (0.00536126148802005)	0	0	4.80603559365272 (0.00175424107616853)
KLK11	0	0	0	0	0	0	2.54106871388003 (0.0074634898296678)	0	0	0
KLK15	0	0	0	0	0	7.30538415343277 (0.00475228636090536)	0	0	0	0
KLK2	0	0	0	0	0	7.05013890158448 (0.00719954120809021)	0	0	0	0
KLK7	0	0	0	0	0	0	0	0	0	8.69700524349499 (6.04631852863325e-09)
KLRD1	-2.2625804552726 (0.00814981186310835)	0	-2.92143854616571 (0.00235996541440914)	0	0	0	0	0	0	0
KMO	0	0	0	0	0	0	0	0	2.3900033815364 (0.0051669625811633)	0
KNCN	0	0	0	0	6.24434293510046 (0.0075784156376657)	0	0	0	0	0
KNG1	0	0	0	0	0	9.62294706111075 (1.44885002054525e-13)	0	0	0	0
KNTC1	0	0	0	0	0	0	0	3.10374650091211 (1.85582009308835e-05)	0	0
KPRP	0	0	0	0	0	0	0	0	0	10.0380454067332 (4.42108121824089e-15)
KRBA1	0	0	1.76125846022787 (0.00355918022018939)	0	0	0	0	0	0	0
KRBOX1	0	0	0	0	0	0	0	0	4.99846987593786 (3.44382073528658e-05)	0
KRBOX1-AS1	0	0	0	0	0	0	0	0	4.74252416103598 (0.000245995066902702)	0
KRBOX4	0	0	0	0	0	2.70653639842635 (3.65836636391649e-05)	0	0	0	0
KRT1	0	0	0	0	6.81201551833002 (0.00158736055535963)	6.40597670733315 (0.000486175206329604)	0	0	0	13.6666337606511 (2.74551157128672e-33)
KRT10	0	0	0	0	0	0	0	0	0	8.59421288843824 (1.37407255691643e-40)
KRT14	0	0	0	0	0	6.26929187881211 (0.000243842318332585)	0	0	8.11731563913744 (2.06203226173896e-07)	11.9721740969415 (3.82346206899977e-10)
KRT15	0	0	0	0	0	0	0	0	0	4.50682210329753 (1.04281749174206e-06)
KRT16	0	0	0	0	0	0	0	0	0	8.1432991246113 (1.98041948749069e-13)
KRT16P2	-4.60222265233292 (0.00224131990637553)	0	0	0	0	0	0	0	-4.21832115948094 (0.000864114992161909)	0
KRT16P6	0	0	0	0	0	0	0	0	6.47902048830229 (0.000118086878345614)	0
KRT17	-3.11762702438693 (0.000634283208279305)	0	-4.84575678153496 (7.74586008103114e-06)	-6.45187941818567 (0.00521924916969436)	0	0	0	0	0	4.37101544126913 (2.73157179605687e-07)
KRT18	-2.34717351538073 (3.50265235091653e-05)	0	-3.80902925955617 (5.58875878887544e-07)	-2.79807980326387 (0.00127190154167078)	0	0	0	-7.88474065905537 (0.000318321534387724)	1.86192042636046 (9.92872486289185e-07)	-2.38783780829437 (0.00418373279023962)
KRT18P15	0	0	0	0	0	0	0	0	-2.86919657720672 (0.00911583206901577)	0

KRT18P3	0	0	0	0	0	7.8466496301183 (0.00175359276277559)	0	0	0	0
KRT18P34	0	0	0	0	0	5.39896877647428 (0.00770494567797495)	0	0	0	0
KRT18P39	0	0	0	0	0	6.28556277714431 (0.00564354943434322)	0	0	0	0
KRT18P60	0	0	0	0	0	4.71238482952729 (0.008391456789343)	0	0	0	0
KRT19	-3.41263289769755 (2.82832994089421e-11)	0	-6.14223052041719 (9.18741380027057e-17)	-2.77746000801237 (0.00134053664912135)	0	0	0	-8.48544504450897 (0.000116318946046)	0	-2.29409556465557 (0.0065038899111892)
KRT2	0	0	0	0	0	7.79997854066755 (0.00217410732729415)	0	0	0	12.0550298213483 (3.79804173385226e-25)
KRT20	6.03973623933099 (1.19770656631686e-06)	0	0	0	0	0	0	0	0	0
KRT223P	0	0	0	0	0	0	0	0	4.61777409380775 (0.00653173002165194)	0
KRT23	0	0	0	0	0	0	0	0	6.51033455034064 (1.62789760145455e-16)	5.51857555703586 (1.2490329549958e-05)
KRT24	0	0	0	0	0	0	0	0	8.27854627764614 (0.00105041592200832)	0
KRT4	0	0	0	0	0	0	3.2851224722514 (0.00708344577967488)	0	0	0
KRT42P	0	0	0	0	0	6.69515730326131 (0.00276995024884354)	0	0	0	0
KRT5	0	0	0	0	0	0	0	0	0	7.8299415881559 (0.00616388033138397)
KRT6A	-7.2006237901644 (0.000101496467419759)	0	-6.10356720033672 (0.00059740345340682)	0	0	0	0	0	0	0
KRT6B	0	0	0	0	0	0	0	0	0	7.05016578133428 (6.26995839021505e-05)
KRT6C	0	0	0	0	0	0	0	0	0	6.86048856106341 (0.00199118553636301)
KRT7	-6.76841225870763 (8.84963215992335e-23)	0	-7.95002632857938 (5.4018411603812e-28)	-6.65415437688084 (1.16057233436552e-09)	-2.57447779976394 (1.01562034359521e-05)	-3.31472721890819 (6.29418211564752e-06)	0	-9.62534391773293 (8.89980484541417e-06)	0	-4.43384594656374 (2.23946483571317e-08)
KRT77	0	0	0	0	0	0	0	0	0	8.51257985747863 (3.30161999159824e-08)
KRT78	0	0	0	0	0	0	0	0	0	6.46816489845318 (0.00889878919560942)
KRT8	-1.75338959537153 (0.000310177203633753)	-5.86728294355515 (0.00777632246461226)	-2.16799664152242 (6.01367746685452e-06)	0	0	0	0	-9.28516901146824 (2.52183395370017e-05)	0	-5.01143983506863 (1.17286202981602e-07)
KRT80	-6.68994825774625 (0.00031413675137255)	0	0	0	0	0	0	0	0	0
KRT82	0	0	0	0	0	7.74022813058379 (0.0021920031485895)	0	0	0	0
KRT8P3	0	0	0	0	0	0	0	0	3.26672247980053 (0.000306016645088808)	0
KRT8P36	0	0	0	0	0	4.18318201056206 (0.00362445641977669)	0	0	0	0
KRT8P48	0	0	0	0	0	5.65624332174295 (0.0042047326169929)	0	0	0	0
KRT9	0	0	0	0	0	7.20898483477395 (0.00651838541340656)	0	0	0	0
KRTAP13-4	0	0	0	0	6.64816874282679 (0.00347040281013931)	0	0	0	0	0
KRTAP21-4P	0	0	0	0	6.24434293510046 (0.0075784156376657)	8.14611295305517 (0.00144837432832693)	0	0	0	0
KRTAP5-3	0	0	0	0	0	6.96977162684898 (0.00897266232869622)	0	0	0	0
KRTDAP	0	0	0	0	0	0	0	0	0	9.57671356631602 (7.403755058462e-13)
KSR2	0	0	0	0	6.1441767214923 (1.85065849156115e-12)	5.50602997427881 (0.000187429640551028)	3.73622977856944 (0.00507210220280526)	0	0	0
KTN1-AS1	0	0	0	0	0	5.23238364490746 (0.00326106397145069)	0	0	0	0
KY	0	0	0	0	0	4.04762637196641 (0.00200100054087787)	0	0	0	0
KYNU	-2.650616347059 (3.63139687808871e-05)	0	-2.44803370489804 (0.00220815957596253)	0	0	0	0	-6.21497876762563 (0.00592692760320304)	0	0
L1CAM	7.32237883710608 (2.55186975066098e-13)	0	3.48797023429195 (0.00741180984594307)	7.26032107337108 (8.43510017108167e-07)	0	0	0	0	0	0
L1TD1	-3.13186815420897 (0.00247974243026752)	0	-3.44614065632334 (0.00215597070658822)	0	0	0	0	0	2.87460060338826 (4.8772635928562e-09)	0
LA16c-306A4.2	0	0	0	0	0	4.71456283204302 (0.00858315001604037)	0	0	0	0
LA16c-380H5.1	0	0	0	0	0	7.05013890158448 (0.00719954120809021)	0	0	0	0
LA16c-390E6.4	-4.57606618974909 (0.0038997168538071)	0	0	0	0	0	0	0	0	0
LACC1	0	0	-2.83404566542716 (0.000397329333343167)	0	0	0	0	0	0	0
LAD1	0	0	3.23773504899594 (6.07940432855318e-09)	0	0	0	0	0	0	2.96011737653429 (0.00394860364224528)
LAG3	2.94106156849627 (0.00873607155410929)	0	0	0	0	0	3.55058622971814 (3.49339771949005e-05)	0	0	0
LAIR1	-2.50071150745886 (1.10154435193268e-07)	0	-3.51391880097909 (1.9509733814717e-08)	0	0	0	0	0	0	0
LAMA2	0	-6.74895590731685 (0.00152805162765139)	0	0	-2.83151829674617 (1.13077098082464e-08)	-1.60537098902144 (0.00746000515986028)	0	-5.43186849398874 (2.02241694846664e-09)	0	0
LAMA3	-2.14080762468368 (5.63330305478278e-07)	0	-2.11922403701033 (1.26839174227241e-05)	0	0	-1.52855766058584 (0.0098339497415745)	0	-7.44242060170677 (0.000621921056643234)	3.21739954916063 (1.52607314603439e-07)	0
LAMA4	0	0	0	0	0	0	0	-3.7846960677333 (2.35944322910511e-05)	0	0
LAMA5	0	0	0	0	0	-1.59563013873287 (0.00538886961141041)	0	-3.3136065714951 (0.000309346380188202)	0	0
LAMB1	0	-2.53569256162815 (0.00502300486866957)	0	0	-1.57053999400378 (8.22042232240907e-07)	-1.9221388807782 (0.00110393843383239)	0	-3.72197669689156 (9.41367752071803e-09)	0	0
LAMB2	0	0	0	0	0	0	0	-2.95882626629131 (0.00060703924197336)	0	0
LAMB3	-3.52978642540606 (4.19597880035333e-05)	0	-5.56238703597221 (1.50131907213248e-08)	-5.51806585826919 (0.00173007547598929)	0	0	0	0	0	0
LAMB4	0	0	0	0	0	0	0	0	5.09910260644319 (6.16475696775953e-09)	5.14826195936343 (0.00111944392414539)
LAMC1	0	0	0	0	0	-2.80807563825603 (1.45416040208667e-06)	0	-5.67581706512591 (4.63422362281308e-11)	0	0
LAMC2	-2.05807998694927 (0.0054966952898219)	0	-3.56634572616774 (3.35101048215901e-07)	-3.38926526345207 (0.00175925752140347)	0	0	0	-6.77332921455148 (0.00214157791036143)	0	0
LAMC3	-6.96152777252728 (5.25772760781743e-10)	0	-3.79375314142696 (3.20308363538686e-07)	-7.5048564367892 (0.000632488132156199)	1.57754440889453 (0.00473825522768037)	0	0	-6.44397708444776 (0.0034521579540114)	1.51920373198683 (0.00158458757953555)	0
LAMP1	0	0	0	0	0	0	0	-2.19508019140638 (0.00808843064440528)	0	0
LAMP3	-8.02846156413625 (1.20029764693679e-16)	0	-8.22731516444991 (2.51329570221111e-18)	-10.12535427229 (7.4504359762753e-06)	-6.29550637828713 (3.31962779014848e-06)	-3.92374513482313 (0.000512652016274378)	0	-5.03963183316904 (8.81419974268597e-05)	-5.61226823830192 (1.23186912931568e-08)	-5.64083017212029 (1.1913535702079e-06)
LAMTOR5-AS1	0	0	0	0	0	4.46895403062909 (0.000150807345976648)	0	0	0	0
LAPTM4A	0	0	0	0	0	0	0	-2.23781055333255 (0.00949508577163203)	0	0
LAPTM4B	0	0	2.11449287939829 (1.61123923495982e-06)	0	0	0	0	0	0	0
LAPTM5	-1.84528006213214 (0.00392896300784984)	0	-3.55674377912493 (9.9761722207918e-07)	0	-2.67923576684286 (0.00136490428858099)	0	0	0	-1.72434937856443 (0.000655690103213779)	0
LARGE	0	0	3.65138961324808 (3.50584133106478e-10)	0	0	0	0	0	0	0
LARP6	0	0	2.59151405065223 (1.09075851085856e-06)	0	0	0	0	0	0	0
LAT2	0	0	-2.10475700901579 (0.00638694650018713)	0	0	0	0	0	0	0
LAX1	0	0	0	0	0	0	2.05206857028305 (0.00572481494409552)	0	0	0
LBH	-2.13622257974228 (0.000160984817549128)	0	0	0	-2.16957839027335 (0.00689116053540707)	0	0	0	0	-2.53270656840411 (0.00863774674833514)
LBP	0	0	0	0	0	8.9088661580217 (1.45019996111943e-16)	0	0	0	0
LBX2-AS1	0	0	0	0	0	2.85572605595608 (0.00426315101829241)	0	0	0	0
LCA5	0	0	-1.84850631573799 (0.00239616756088102)	0	0	0	0	0	0	0
LCE1A	0	0	0	0	0	0	0	0	0	6.71609651231352 (0.00360897601821362)
LCE1C	0	0	0	0	0	0	0	0	0	6.63809277000255 (0.00486975444226376)
LCE2B	0	0	0	0	0	0	0	0	0	7.71610814515995 (1.79240991507195e-05)
LCE6A	0	0	0	0	0	0	0	0	0	6.99173489038131 (0.00110635528067473)
LCMT1-AS2	0	0	0	0	0	0	0	0	4.66436549020309 (8.26929427443733e-07)	0
LCN10	0	0	5.30438556592978 (2.53414956697647e-07)	0	0	0	0	0	0	0
LCN2	-3.42602450404926 (4.04527138849435e-05)	0	0	0	0	0	0	5.01961857842472 (2.56715942301807e-13)	-2.78774203329344 (0.00263979600573997)	0
LCN6	0	0	4.51733173704018	0	0	0	0	0	0	0

			(0.000610765563961013)							
LCP1	-1.78786828754665 (0.00600994422586735)	0	-2.67706254474711 (0.000227810394314727)	0	0	0	0	0	0	0
LCP2	-1.73954986122326 (0.00114185737048828)	0	-2.62355680930203 (1.72893981357643e-07)	0	0	0	0	0	0	0
LCT	0	0	0	0	0	7.38428400772611 (0.000798460619048036)	0	0	0	0
LDB1	0	0	0	0	0	0	0	2.39958427509429 (0.00107908926753578)	0	0
LDB2	0	0	0	0	0	0	0	-3.08893214731367 (0.00446702777199448)	0	0
LDB3	3.19746501843259 (6.56925934597257e-14)	0	7.27076160530066 (5.8003582913197e-43)	2.7922387925158 (0.00220491372519014)	2.62867327886096 (0.000629942873795405)	0	0	0	0	0
LDHB	0	0	1.84871142858594 (5.43372501008417e-06)	0	0	-1.7960099923495 (0.00212321710063649)	0	0	0	0
LDHD	0	0	4.57422215791104 (3.84387077206233e-07)	0	0	4.24778440039342 (0.000661620708171093)	0	0	0	0
LDLRAD1	0	0	0	0	0	0	0	0	-4.67121715240749 (0.00696438067300128)	0
LDLRAD3	0	3.59661678280779 (0.000129578245839774)	0	0	0	0	0	0	0	0
LDLRAD4	0	0	0	0	2.79426351457992 (0.000131038518134922)	0	0	0	1.56164850209685 (0.000130218069427474)	0
LDOC1	0	0	0	0	0	0	0	0	-2.33001336096979 (0.00497176849524966)	0
LEAP2	0	0	0	0	0	6.78656408953264 (9.26144782576463e-13)	0	0	0	0
LECT2	0	0	0	0	0	6.37378740160168 (0.00574882784125654)	0	0	0	0
LEFTY2	0	0	0	0	0	0	0	0	4.79802646618277 (0.000248748023438191)	0
LEP	0	0	0	0	0	0	0	0	4.88340537313965 (0.00262835899373822)	4.81048036571479 (0.00327807880439541)
LGALS14	0	0	0	0	0	0	0	0	8.07830926318868 (6.22453466851124e-07)	0
LGALS17A	0	0	0	0	0	0	3.71970205183734 (0.00496271000373839)	0	0	0
LGALS3	0	0	0	0	-2.28679832268013 (0.00268515307619516)	0	0	-3.22617581998128 (0.00112129799370311)	0	0
LGALS3BP	0	0	0	0	0	0	0	-2.84443510469971 (0.00341027645009845)	0	0
LGALS4	5.50565974723148 (3.27539506201689e-06)	0	0	0	5.83005063945246 (0.00374446131635038)	8.08407474169256 (7.29230270801346e-11)	0	0	0	0
LGALS8	0	2.00139197192426 (0.00420701994452319)	0	0	0	0	0	0	0	0
LGI2	3.34576527708524 (9.22210692694371e-09)	0	0	0	0	0	0	0	0	0
LGI4	3.09171198834148 (1.40179708583989e-08)	0	0	2.9775633898007 (0.00300150872817905)	0	0	0	0	0	2.93570544149197 (0.00337739637438035)
LGMNP1	0	0	0	0	0	5.54826588246871 (0.00680606565398487)	0	0	0	0
LGR4	0	0	0	0	2.53943567870097 (0.00792882239690145)	2.74047942269685 (0.00303496905962372)	0	0	4.33449681374204 (4.29366468954599e-19)	0
LGR5	0	0	0	0	0	0	0	0	5.54548137504845 (1.01204357602688e-07)	0
LGSN	4.79441391975154 (0.00334546280477916)	0	0	0	6.47559966567641 (0.0042482603748159)	0	5.22266638707585 (0.00635148578093914)	0	0	0
LHFPL2	0	0	-2.4899145299543 (1.13343765851555e-05)	0	-3.13622984369915 (0.000692723981240758)	0	0	0	0	0
LHFPL3-AS1	0	0	0	0	6.47559966567641 (0.0042482603748159)	0	0	0	0	0
LHFPL4	0	0	0	0	0	6.35963933890164 (0.00612113747267631)	0	0	0	0
LHX2	0	0	0	0	0	6.24442330405376 (0.00439973633388583)	0	0	0	0
LHX4	0	0	0	0	0	0	0	0	2.37601377873938 (0.000956472518746702)	0
LHX9	0	0	0	0	4.70920664603196 (0.0050090705163517)	4.98310574920786 (0.00269310413620279)	0	0	0	0
LIF	0	0	-2.74914481102827 (0.00115126318899867)	0	0	0	0	-6.22979610044375 (0.00567497905984887)	0	0
LIFR	0	0	0	0	0	0	0	-3.32132643886183 (0.000171581711458243)	0	0
LIG1	0	0	-1.70339923999628 (0.009563070838914)	0	0	0	0	3.48955932390812 (1.42639699016486e-05)	0	0
LIG3	0	0	0	0	0	2.03219678631737 (0.00262470144344948)	0	0	0	0
LILRA6	0	0	0	0	0	0	0	0	-1.92777431954454 (0.00325112791850225)	0
LILRB1	-2.5785890151009 (0.00236432879869872)	0	-4.60535978194327 (3.29253790125993e-05)	0	0	0	0	0	0	0
LILRB2	-2.78558057976745 (1.08518598450528e-05)	0	-3.294350959587 (2.48831856245259e-05)	-2.74096496887614 (0.0083682955216614)	0	0	0	0	0	0
LILRB3	-3.25097361334746 (2.92263610214402e-06)	0	-2.83689365260147 (0.0001938072724033)	0	0	0	0	0	-1.99023086375989 (0.00125448247970779)	0
LILRB4	-2.74496581679946 (0.00109849134829094)	0	-4.64382284463358 (1.70560541960866e-05)	0	0	0	0	0	0	-5.51268688767072 (0.003922363825466)
LIMA1	0	0	0	0	0	0	0	-3.90461192509623 (1.21757724042494e-05)	0	0
LIMCH1	-2.57175840128097 (3.4068771615174e-09)	-5.98946419272981 (0.00511512709678182)	0	0	0	0	0	-5.39891076336743 (3.28537580958153e-07)	0	0
LIMD2	0	0	0	0	0	2.53653406080848 (0.00549644514488261)	0	0	0	0
LIMK2	-1.79103847153073 (0.00013211269060907)	0	0	-2.27500264581745 (0.0051856772986913)	0	0	0	0	0	0
LIMS1	0	0	0	0	0	-1.63472589357436 (0.00883811982422173)	0	0	0	0
LIMS2	1.73304173631094 (0.00119361376086056)	0	1.61499113787756 (0.000177929321484418)	0	0	0	0	-6.8131193893338 (0.002579123787089)	0	0
LIN28B	0	0	0	0	6.81201551833002 (0.00158736055535963)	0	0	0	9.93170653122907 (1.04714988044603e-18)	0
LINC-PINT	0	0	0	0	0	-1.60923308816231 (0.00501398986635138)	0	0	0	0
LINC00116	0	0	4.0955147788409 (5.87539473867476e-09)	0	0	0	0	0	0	0
LINC00152	-2.44925625969784 (0.00882441918424509)	0	0	0	0	0	0	0	0	0
LINC00176	0	0	0	0	4.63481931426922 (0.002497051687134)	3.98420625817499 (0.00936323812648291)	0	0	0	0
LINC00200	0	0	0	0	0	6.28446816679906 (0.00531418671143485)	0	0	0	0
LINC00202-1	0	0	0	0	0	4.18788527930877 (0.00327123773493724)	0	0	0	0
LINC00261	0	0	0	0	0	6.39983305216503 (2.99264762414353e-11)	0	0	0	0
LINC00265	0	0	0	0	0	0	0	0	1.7589245552466 (0.00928691766609555)	0
LINC00299	0	0	0	0	0	6.74647277501199 (2.87680016893142e-06)	0	0	0	0
LINC00304	0	0	0	0	5.90054662841542 (0.0004546866965978)	0	0	0	0	0
LINC00342	0	0	0	0	0	0	0	0	-1.56086640234159 (0.00656012562081375)	0
LINC00346	0	0	0	0	0	0	0	0	2.25355246850383 (0.00246024061988445)	0
LINC00384	0	0	0	0	5.60383939011682 (0.00679500667743972)	7.26880303304766 (7.86594323287741e-05)	0	0	0	0
LINC00456	0	0	0	0	0	0	0	0	4.7777599582143 (0.000675821858730241)	0
LINC00461	0	0	0	0	0	6.09432585898859 (0.00893315405230805)	0	0	0	0
LINC00470	0	0	0	0	0	0	0	0	4.63123037126276 (0.000122484233022027)	0
LINC00473	4.80267520576982 (0.00336133062987534)	0	0	7.27245160959529 (0.000308198984895893)	0	0	0	0	0	0
LINC00476	0	0	0	0	0	3.3666721151064 (0.00284920475359293)	0	0	0	0
LINC00485	0	0	0	0	0	5.61656197410922 (0.00216343463811686)	0	0	0	0
LINC00491	0	0	0	0	7.23806043843821 (0.000719882457903311)	0	0	0	0	0
LINC00493	0	0	0	0	0	0	1.7672507423578 (0.00083170673970707)	0	0	0
LINC00517	0	0	0	0	0	6.150705797245 (0.00750265804279433)	0	0	0	0
LINC00528	0	0	0	0	0	6.32644749635907 (0.00137620335790151)	0	0	0	0
LINC00551	0	0	0	0	0	0	3.8865578554985 (0.00948862427973391)	0	0	0
LINC00574	0	0	0	0	0	7.70752620555927 (0.00311614432761171)	0	0	0	0

LINC00578	4.46840300711596 (2.64084900269147e-06)	0	0	5.08834357999445 (0.0041295876601621)	0	0	0	0	0	0
LINC00598	0	0	0	0	0	5.47615616674083 (0.00805058994943075)	0	0	0	0
LINC00606	0	0	0	0	6.65447770053204 (0.00254565647500638)	8.14082377804611 (0.0010498836238954)	0	0	0	0
LINC00608	0	0	0	0	0	5.79757837852406 (0.000415002001727793)	0	0	0	0
LINC00635	0	0	0	0	0	6.03826101173533 (0.00784359047003729)	0	0	0	0
LINC00639	0	0	6.74312867368406 (3.71458948293635e-09)	0	5.91841390709533 (0.0038434302861903)	0	0	0	0	0
LINC00643	0	0	0	0	0	0	0	0	4.88334370110028 (0.00017127626361365)	0
LINC00645	0	0	0	0	0	0	0	0	6.8369569636562 (9.93902810903083e-08)	0
LINC00648	0	0	0	0	0	0	0	0	4.94671094257128 (0.000567024245273455)	0
LINC00649	0	0	0	0	0	2.97104627922468 (0.00383599479407115)	0	0	2.37700001032907 (3.0384050685466e-05)	0
LINC00661	0	0	0	0	5.7800642702998 (0.00591384444719865)	0	0	0	0	0
LINC00664	0	0	0	0	0	4.96251991043601 (0.00185349862037752)	0	0	0	0
LINC00668	0	0	0	0	0	6.12847066122739 (0.00911786229955422)	0	0	0	0
LINC00671	0	0	0	0	0	5.38446274875018 (0.000585160003903618)	0	0	0	0
LINC00675	0	0	0	0	0	7.01138320954799 (0.000575301585252019)	0	0	0	0
LINC00689	0	0	0	0	5.05553949784534 (0.00518076580360529)	6.25216677859922 (0.000141633178335457)	0	0	3.55178656983657 (0.000609204041573581)	0
LINC00693	4.71957890848299 (0.00493062623352652)	0	0	0	0	0	0	0	0	0
LINC00700	0	0	0	0	0	7.36854323903852 (0.000706119494411524)	0	0	0	0
LINC00837	0	0	0	0	0	6.8968980632387 (0.00193447755663905)	0	0	0	0
LINC00865	4.85115731980025 (0.00276412884912671)	0	0	0	0	0	0	0	3.51624947316458 (0.00382086570838459)	0
LINC00877	0	0	0	0	0	0	3.43009143989277 (0.0086679003812013)	0	0	0
LINC00881	0	0	5.80499517751375 (5.80354242990863e-05)	0	0	0	0	0	0	0
LINC00882	0	0	0	0	0	0	0	0	5.14586224074758 (1.55414033503722e-06)	0
LINC00883	0	0	0	0	0	0	0	0	2.29369561033979 (5.41223920475507e-06)	0
LINC00885	0	0	0	0	0	0	0	0	4.43038738470397 (0.00339305084186128)	0
LINC00886	0	0	0	0	0	3.99158537049277 (0.00758584347416936)	0	0	0	0
LINC00888	0	0	0	0	0	0	0	0	2.29626145745072 (0.00048736612076953)	0
LINC00890	0	0	0	0	0	6.27578664977402 (0.000420706478749188)	0	0	0	0
LINC00898	0	0	0	0	0	7.97896976436846 (0.00174461789794878)	0	0	0	0
LINC00899	0	0	0	0	0	4.08438752646177 (0.00525160716635468)	0	0	0	0
LINC00907	0	0	0	0	0	6.01676452054938 (0.000521154441331288)	0	0	0	0
LINC00910	0	0	0	0	0	4.12828450593799 (0.00831792584345335)	0	0	0	0
LINC00924	0	0	0	0	6.15314616467714 (0.000349246824048648)	0	0	0	0	0
LINC00925	0	0	0	0	0	7.17600624974043 (0.000918535933007756)	5.31676336758893 (0.00650442378534648)	0	0	0
LINC00933	0	0	0	0	0	4.83367223322578 (0.00811363917968091)	0	0	0	0
LINC00939	0	0	0	0	0	6.45134930668839 (3.21514352198901e-05)	0	0	0	0
LINC00948	0	0	0	0	0	7.21261123709105 (0.00552449200967179)	0	0	0	0
LINC00957	0	0	3.50348937697804 (0.000322478906699233)	0	4.11501695406052 (0.00106633860928169)	0	0	0	0	0
LINC00963	0	0	0	0	0	1.83191584611497 (0.00752793470118511)	0	0	0	0
LINC00967	0	0	0	0	0	0	0	0	7.14517241387251 (1.9188543109867e-05)	0
LINC00982	0	0	2.72101416515565 (0.00894816594704269)	0	0	0	0	0	0	0
LINC00989	0	0	0	0	0	5.71409214225606 (0.00498432177775096)	0	0	0	0
LINC01011	0	6.22630368607237 (1.31599189003038e-05)	0	0	0	0	0	0	0	0
LINC01013	0	0	0	0	6.24434293510046 (0.0075784156376657)	0	0	0	0	0
LINC01016	0	0	0	0	0	7.00977001425344 (2.54720010354104e-05)	0	0	0	0
LINC01018	0	0	0	0	0	7.24015183656656 (5.09845846426962e-07)	0	0	0	0
LINC01019	0	0	0	0	6.61820674621378 (0.00367430017474216)	0	0	0	0	0
LINC01089	0	0	0	0	0	0	0	4.65552513732854 (0.002557234417582)	0	0
LINC01094	0	0	0	0	0	0	0	0	1.79453441645687 (0.0026208500742597)	0
LINC01105	0	0	0	0	6.07866553133097 (0.00201106327764286)	0	0	0	0	0
LINC01119	0	0	0	0	0	5.12162256302208 (0.00131384529735468)	0	0	0	0
LINC01121	0	0	0	0	0	6.01004497528137 (0.00262659388663862)	0	0	0	0
LINC01125	0	0	0	0	3.15487002776474 (0.00172388277714791)	3.11816418469797 (0.00809609414895266)	0	0	0	0
LINC01128	0	0	2.30500573730478 (6.63706046395414e-05)	0	0	0	0	0	0	0
LINC01132	0	0	0	0	5.46967821176292 (0.0025456407800973)	0	0	0	0	0
LINC01138	0	0	0	0	4.11757944729539 (0.00114949583205901)	0	0	0	0	0
LINC01144	0	0	0	0	0	5.52246573843628 (0.00522921098428029)	0	0	0	0
LINC01146	0	0	0	0	0	5.11394428331163 (0.000327425829385089)	0	0	0	0
LINC01159	0	0	0	0	0	7.2101153127025 (0.000966908883386606)	0	0	0	0
LINC01164	0	0	0	0	6.47559966567641 (0.0042482603748159)	7.40324802854046 (0.000782677106135631)	0	0	0	0
LINC01197	0	0	0	0	3.61654633819228 (0.00508736880644989)	3.36659942484906 (0.00576174177783357)	0	0	0	0
LINC01204	0	0	0	0	0	7.11254271341322 (0.00756733940646282)	0	0	0	0
LINC01206	0	0	0	0	6.61685523282843 (0.00161243595440384)	7.22763870305629 (0.0010744475832471)	0	0	0	0
LINC01230	0	0	0	0	5.3010712834665 (0.00567214239710959)	0	0	0	0	0
LINC01232	0	0	0	0	0	0	4.09982157552288 (0.00151566191276743)	0	0	0
LINC01272	-2.93194410107283 (0.00386105533710941)	0	-3.87169912257925 (0.000408611147552074)	-6.20319076423728 (0.00800529785295795)	0	0	0	0	-2.09814642438517 (0.00130687314974777)	0
LINC01287	0	0	0	0	0	7.17442331454011 (0.00115960044943826)	0	0	0	0
LINC01289	0	0	0	0	0	6.96977162684898 (0.00897266232869622)	0	0	0	0
LINC01305	0	0	0	0	6.47559966567641 (0.0042482603748159)	0	0	0	0	0
LINC01310	0	0	0	0	0	7.65116276570296 (0.000339733960470625)	0	0	0	0
LINC01314	0	0	0	0	0	3.95479677057166 (0.000730101701581652)	0	0	0	0
LINC01320	0	0	0	0	7.57032718079248 (1.65869245214268e-07)	6.70241193570316 (0.00159400523605601)	0	0	0	0
LINC01322	0	0	0	0	0	7.93694530962604 (0.000312383561653094)	0	0	0	0
LINC01341	0	0	0	0	0	0	4.00193108464533 (0.00855034310211227)	0	3.95540584653083 (0.00157412516055188)	0
LINC01343	0	0	0	0	0	6.54401974831726 (0.00335150846722125)	0	0	0	0
LINC01348	0	0	0	0	0	7.71828771850487	0	0	0	0

						(1.4152136561699e-10)				
LINC01354	0	0	0	0	0	0	5.3563246596374 (0.00396581834271006)	0	0	0
LINC01355	1.86122333998002 (0.000196318424692296)	0	0	0	0	0	0	0	0	0
LINC01376	0	0	0	0	0	0	0	0	4.09508959819097 (0.00123120883549517)	0
LINC01415	0	0	0	0	0	0	0	0	4.51001414783744 (2.57749407612453e-06)	0
LINC01426	0	0	0	0	5.89833031987037 (0.00197278958832419)	0	0	0	0	0
LINC01443	0	0	0	0	0	7.08201150050981 (0.00710054012903161)	0	0	0	0
LINC01446	0	0	0	0	0	0	0	0	4.17872717014914 (0.00209807882830295)	0
LINC01451	0	0	0	0	0	6.85997879166201 (0.00216189124228452)	0	0	0	0
LINC01471	0	0	0	0	0	7.00881329009256 (0.00886510847483269)	0	0	0	0
LINC01483	0	0	0	0	0	0	0	0	4.40852742328015 (0.00156640337492122)	0
LINC01485	0	0	0	0	0	5.88235566435141 (0.00114016017891997)	0	0	0	0
LINC01524	0	0	0	0	0	0	0	0	4.2589005673731 (0.000752900238915635)	0
LINC01527	0	0	0	0	0	7.44279116366548 (0.00404563973443074)	0	0	0	0
LINC01537	0	0	0	0	0	5.33140572831567 (9.05703066885425e-05)	0	0	0	0
LINC01550	0	0	0	0	0	0	3.54773775344364 (0.00233190069834032)	0	0	0
LINC01553	0	0	0	0	0	7.07340643839405 (0.000349631971204926)	0	0	0	0
LINC01554	0	0	0	0	0	5.86161967138455 (3.66560895054499e-07)	0	0	0	0
LINC01578	0	0	-2.28347816784893 (2.32646817421908e-06)	0	0	-1.7435003668095 (0.00869583489645789)	0	0	0	0
LINC01588	0	0	0	0	0	5.48595371074904 (1.97359008886444e-05)	4.13630833588018 (0.00347652056834736)	0	0	0
LINGO1	0	0	0	0	0	0	0	0	2.94462861276184 (0.00646613998775713)	0
LIPA	0	0	-2.02867852728233 (0.00555301115965679)	0	0	0	0	0	0	0
LIPC	0	0	0	0	0	5.26084505218626 (3.62624309928813e-05)	0	0	0	0
LIPE	0	0	4.5735043395196 (1.94150388307529e-09)	0	3.7918476285861 (0.00166223360703613)	3.51601290111741 (0.00597404163090842)	0	0	0	5.74449309431773 (2.63312315196613e-07)
LIPE-AS1	0	0	0	0	0	5.25114728585027 (0.00306451991826531)	0	0	0	0
LIPG	0	0	-2.43073897107376 (0.00596548430422889)	0	0	0	0	0	0	0
LIPH	-4.61561484183225 (6.53507619632114e-06)	0	-3.88653249045802 (2.54556729480085e-05)	-6.40899936103727 (0.00508650866603145)	0	0	0	0	-4.74732187775747 (2.39180810071975e-08)	0
LITAF	-1.63215473602488 (0.000240852946181818)	0	-1.96883265017647 (7.90092836624057e-06)	0	0	0	0	0	0	0
LL22NC03-22D1.1	0	0	0	0	7.37105784090328 (6.49911637178226e-05)	7.79287098853117 (1.48769725942702e-06)	6.55525556432245 (0.0012588099583356)	0	0	0
LL22NC03-27C5.1	0	0	0	0	0	5.401882563661 (0.00672774209339887)	0	0	0	0
LL22NC03-86G7.1	0	0	0	0	1.97970721036552 (0.00673634085514879)	3.8211418045472 (0.000164943073982884)	0	0	0	0
LLGL2	-3.00093754427421 (8.14046993133394e-05)	0	-2.94438718694024 (3.87208747717911e-05)	0	0	0	0	0	-1.83039867644482 (0.000107766088035495)	0
LMAN2L	0	0	1.52645129263378 (0.00712993425376047)	0	0	0	0	0	0	0
LMCD1	0	0	0	0	0	0	0	-3.4729240724822 (0.000251154431729005)	0	0
LMNB1	-1.68524872744749 (0.000951188330193629)	0	0	0	0	0	0	2.21419193019392 (0.00552120643474475)	0	0
LMO3	0	0	-4.50876227629751 (3.71544027703676e-07)	0	-3.04288621437087 (0.00828032761605739)	0	0	-8.04286797027347 (0.000523557097826997)	-2.94209461093064 (0.00677722598575783)	0
LMO7	-4.09046872959184 (3.36011023675495e-32)	0	0	-3.42009868695246 (1.69870636651844e-07)	0	0	0	-4.94783130421715 (1.39763915315328e-07)	0	-1.8943595198114 (0.00181955601134186)
LMOD1	3.70067255032236 (4.76342717146812e-24)	0	0	4.12510568794177 (5.77505618348734e-10)	0	-1.81016895469171 (0.00232807520838196)	0	-3.54671576043522 (0.000514182723279507)	0	2.03589430982576 (0.0030457518634509)
LMOD2	0	0	12.7781335266643 (4.39823673776909e-29)	0	0	0	0	0	0	0
LMOD3	0	0	7.769610989346 (1.89174594281435e-19)	0	0	0	0	0	0	0
LMTK3	0	0	0	0	2.8409265649179 (0.00419971160139154)	0	0	0	0	0
LMX1A	0	0	0	0	0	6.80030666102153 (0.00242138477854474)	0	0	0	0
LMX1B	0	0	0	0	5.94644326484529 (7.89967204915506e-05)	7.30076644191075 (0.000224699941771515)	0	0	0	0
LNX1	0	0	3.79375354351327 (0.00109037274618554)	0	0	4.2705656676009 (0.00725689578334049)	2.64616703866079 (0.00929041444054252)	0	4.75656502670849 (1.53520983759426e-09)	4.96220821997177 (0.00225502016543652)
LONRF2	2.23379170870641 (2.54758069050347e-07)	0	2.30991716674816 (1.89232948983952e-07)	2.56445281857881 (0.00491436432416271)	0	2.58913635284956 (0.00668984843231897)	0	0	0	0
LONRF3	0	0	-2.06984326377951 (0.000814846607246194)	0	0	0	0	0	-5.09435505278026 (2.28382239330921e-11)	0
LOR	0	8.83438846949353 (1.89611992709485e-06)	0	0	0	0	0	0	0	9.06810071310903 (1.58836437432225e-10)
LOX	0	0	-2.43999733513405 (0.000230893648981788)	-2.64937160061665 (0.000627356269577099)	-2.80615340136585 (0.000811912939097317)	0	0	-5.17539075301425 (0.00114852094576723)	0	0
LOXHD1	0	0	0	0	3.67095801728281 (0.00175254207829262)	0	0	0	0	0
LOXL2	1.90522873756877 (0.00165035336914652)	0	0	0	0	0	0	0	2.55122049049032 (4.53634355018862e-08)	0
LPA	0	0	0	0	0	7.23068620363946 (0.000292733390031939)	0	0	0	0
LPAL2	0	0	0	0	0	5.254861387678 (0.00195007669793525)	0	0	0	0
LPAR1	0	0	0	0	0	-2.3968890844877 (0.00794655827824264)	0	-4.57252316891647 (0.0052121497266668)	0	0
LPAR2	-4.46115686395003 (0.0016375433786025)	0	0	0	0	0	0	0	0	0
LPAR3	0	0	3.48649910703135 (0.000300214016408794)	0	0	0	0	0	0	0
LPCAT1	-2.74145762260461 (2.5613162741567e-15)	0	-2.76596514996038 (7.2024855762582e-12)	-2.60137415636022 (0.000112287104011839)	-2.93872060866664 (4.96508368631665e-07)	-2.18130069651821 (4.89674691258977e-05)	0	-1.9589507658576 (0.00528556176988794)	0	-2.4377294147806 (0.000243263736065747)
LPHN2	0	0	-1.54924545255478 (0.000149181244736556)	0	-1.83475330906086 (0.00549113906285498)	0	0	-4.86703039816475 (9.29645187595215e-06)	0	0
LPHN3	0	0	0	0	0	0	0	0	1.77671433800962 (0.00661426142259002)	0
LPL	-2.02607363080223 (0.000193009493696902)	0	3.9393781618899 (4.70870612397377e-09)	0	0	0	0	0	0	0
LPO	0	0	0	0	0	6.22626181674637 (0.000527411130460376)	0	5.59448193895326 (0.00781785893269641)	0	0
LPP	1.71521525408669 (1.28843196993331e-08)	0	0	1.63092212558994 (0.00307175687001196)	0	-1.76883064788582 (6.77241609204088e-06)	0	-5.12974662809757 (7.32554382412886e-13)	0	0
LPP-AS2	0	0	2.71179406479857 (0.00433098547568603)	0	0	0	0	0	0	0
LPPR2	0	0	0	0	0	0	0	0	-1.5756293682193 (0.00256680065414391)	0
LPXN	0	0	-2.0746055760186 (0.00762169160678726)	0	0	0	0	0	0	0
LRCH2	0	0	0	0	0	0	0	-5.83864550463274 (0.00801806288600581)	0	0
LRCH4	-1.65245082709868 (0.00162300615189801)	0	0	0	0	0	0	0	0	-2.61949889204491 (0.00727592277977959)
LRCOL1	0	0	0	0	0	4.34101225711739 (0.00590287439479212)	0	0	0	0
LRG1	0	0	0	0	0	5.17733433807577 (9.02363763442362e-14)	0	0	-3.79537687007357 (0.000286068879271409)	0
LRIG1	2.65458769683099 (3.72876177858916e-12)	0	2.10934321164963 (3.86909028703431e-06)	2.14991786425371 (0.00415868696294033)	0	0	0	0	0	0
LRIG3	1.5075511766682 (0.00354009542943277)	0	0	0	0	0	0	0	0	0
LRIT3	0	0	0	0	0	5.61047017166328 (0.00980572520588432)	0	0	0	0
LRP1	0	0	0	0	0	0	0	-2.53860339834367 (3.92794510935603e-05)	0	0
LRP11	0	0	-1.87353601642159 (0.000161013398569781)	0	0	0	0	0	0	0
LRP1B	3.62479112632509 (0.000997327469861346)	0	0	0	0	5.18623952755492 (0.000223489170795799)	0	0	0	0
LRP2	-4.70145325139498 (2.35072592703647e-09)	0	-4.90680453371471 (1.99876403291083e-09)	-7.74710920384072 (0.000418067948173071)	3.04820295590282 (0.00200814273313697)	0	0	-6.69052734811487 (0.00239654050913811)	0	-4.58405059821246 (0.000412767213912835)
LRP3	0	0	2.38493423417374 (0.00222969482991484)	0	0	2.67669985259337 (0.00733169872442988)	0	0	0	0

LRP4	0	0	2.31457696526321 (0.00327562498886028)	0	0	0	0	0	0	0
LRP5L	0	0	0	0	0	0	0	0	-2.84894719410031 (0.000205826376534213)	0
LRP8	0	0	0	0	2.06812998264901 (0.00272007226576993)	0	0	0	0	0
LRRC1	0	0	0	0	0	0	0	0	2.11393387639459 (8.36249334303748e-06)	0
LRRC10	0	0	6.15865153654484 (6.15962095576247e-05)	0	0	0	0	0	0	0
LRRC14B	0	0	5.7097788681923 (0.000511337700648219)	0	0	0	0	0	0	0
LRRC15	0	0	0	0	0	0	0	0	5.10396963680827 (0.00366266293016152)	0
LRRC19	0	0	0	0	0	4.04275368250794 (0.000769543942011422)	0	0	0	0
LRRC2	0	0	6.18582671129844 (4.0039268126712e-15)	0	0	0	0	0	0	0
LRRC20	0	0	2.41505054091729 (0.00618932035885622)	0	0	0	0	0	0	0
LRRC28	1.98746613094807 (0.00341163378872599)	0	0	0	0	0	0	0	0	0
LRRC3	1.79372658993217 (0.00731423899528753)	0	0	0	0	3.07486967690202 (0.00182016749637974)	0	0	0	0
LRRC32	0	0	0	0	0	0	0	-3.38072172566542 (0.000591787712816273)	0	0
LRRC36	0	0	0	0	0	0	0	0	3.97986969785866 (0.000110586175904258)	0
LRRC37A11P	0	0	0	0	0	6.1282743832105 (0.00972726320301328)	0	0	0	0
LRRC37A7P	0	0	0	0	0	6.13797261868197 (0.00191367728110359)	0	0	0	0
LRRC39	0	0	5.97629003758837 (1.72151596979908e-07)	0	0	0	0	0	0	0
LRRC4	-2.06818432030711 (0.00327494800335972)	0	0	0	0	0	0	0	0	0
LRRC47	0	0	2.05487870594337 (5.9599856966753e-05)	0	0	0	0	0	0	0
LRRC4C	2.80604438551955 (0.00817191958410527)	0	0	0	4.04150915565423 (0.00226982816762174)	0	0	0	0	0
LRRC55	0	0	0	0	0	7.638057616878 (1.74385286523155e-06)	0	0	0	0
LRRC59	0	0	0	0	-1.51644374345678 (0.00393048029494566)	0	0	0	0	0
LRRC61	0	0	0	0	0	2.57624707860951 (0.00912346213736525)	0	0	0	0
LRRC74B	0	0	0	0	4.55976189418693 (0.00354859994922483)	4.10730915145547 (0.0023256363245714)	0	0	0	0
LRRC75A-AS1	0	0	-1.64160001589591 (8.99892161366386e-05)	0	0	0	0	0	0	0
LRRC75B	0	0	0	0	0	2.9937208591954 (0.00960671223836519)	0	0	0	0
LRRC8B	0	2.75139163876796 (0.00266171074336472)	0	0	0	0	0	0	0	0
LRRFIP2	0	0	0	0	-2.15553522734417 (0.0011851505814652)	0	0	0	0	0
LRRIQ1	-3.70801167347123 (0.000274814608137962)	0	-4.31714612946655 (0.000185971885855593)	-6.36138059674486 (0.0078813507992777)	0	0	0	0	-6.15875466561743 (2.06599930867938e-08)	0
LRRK2	-3.35270681374184 (1.03486879376077e-13)	0	-4.34637295395179 (1.60211952033734e-14)	-2.83510044469927 (0.000361884046965921)	-2.9958159044092 (5.89769812636735e-05)	-3.71616023109446 (6.23446216402641e-09)	0	-2.18173536440506 (0.00755061672958146)	-4.08011687620271 (1.26449868642017e-15)	-3.68622390722867 (5.35554403722257e-06)
LRRN4	0	0	0	0	0	0	0	0	-4.63942697405645 (0.000666477256695941)	0
LRRTM2	0	0	-1.73185915488756 (0.000476558798504416)	0	0	0	0	0	0	0
LRWD1	0	0	0	0	0	0	0	0	-1.71283824271181 (0.00685301713508197)	0
LSAMP	0	0	-3.05829491329052 (1.96488144143895e-06)	0	0	0	0	-6.70045148474419 (0.00227585979976901)	-1.98468714698664 (0.00928209287251983)	-3.5971459467945 (0.000709447994647145)
LSINCT5	0	0	0	0	0	6.10213009384402 (0.00755053791667713)	0	0	0	0
LSP1	0	0	0	0	0	0	0	3.02656132327884 (0.000947925938088469)	0	0
LSR	-1.81875256268632 (0.00306206469359035)	0	0	0	0	0	0	0	0	0
LTA4H	-1.54942648878068 (0.000161611067935899)	0	-1.54025565224177 (0.000248901715909586)	0	0	-1.52577483127342 (0.0038587041758404)	0	0	-2.17726449651815 (3.15707673823318e-08)	0
LTBP1	0	0	0	0	0	0	0	0	1.63689816376551 (1.08440467290876e-05)	0
LTBP2	-3.47235951074609 (1.27286631448619e-14)	-6.26051581636772 (0.00382488299725301)	0	-2.36183376309476 (0.00146778469650059)	-3.17184616128851 (2.54214540805329e-07)	0	0	-4.25932754172672 (1.981205534012e-06)	0	0
LTBR	0	0	-1.78268246180003 (8.35240923652032e-08)	0	0	0	0	-3.13493769591541 (7.12566692107194e-05)	0	0
LTF	-3.4448928729785 (0.000131136668930813)	0	0	0	2.71151440459526 (0.0047770581795014)	0	3.41310605818692 (0.00101381991758298)	6.60684324116045 (6.15673948211595e-17)	-3.57879429311889 (7.9520416157956e-06)	0
LUC7L3	0	0	0	0	2.00397786250035 (0.000115202495171122)	0	0	0	0	0
LUCAT1	-2.60251655704422 (0.00161829044667373)	0	-2.68909138762918 (0.00219558653676402)	0	0	0	0	0	-2.52414566043674 (0.000403284137371853)	0
LUM	-1.7846818362531 (0.00362094301392478)	-6.9459732875246 (0.0015515397489349)	-2.72910033199335 (5.86507087070879e-06)	-3.02939998658177 (0.000348599201524196)	-2.04399262871462 (0.00754488843251263)	-3.18197094529932 (9.90092678331587e-06)	0	-5.65034963670558 (1.01563164225255e-07)	0	-2.62556395957967 (0.00184722417404304)
LURAP1L	0	0	0	0	0	0	0	-5.97433888984096 (0.00743356690641071)	0	0
LUZP1	0	0	0	0	0	0	0	-3.34575467697564 (0.000432461781169504)	0	0
LY6D	0	0	0	0	0	0	0	0	0	8.32616578511863 (1.68617003556712e-07)
LY6E	0	0	0	-2.37418589871657 (0.00425106777131568)	0	0	0	0	0	0
LY6G5C	0	0	0	0	0	6.51880613963719 (0.00087057577064043)	4.96549063866603 (0.00720128847539768)	0	0	0
LY6G6C	0	0	0	0	0	0	0	0	4.29029643291832 (0.00476103965393585)	7.18719121025702 (3.0257478438356e-05)
LY75	-4.15147396861984 (0.00485905904504622)	0	0	0	0	0	0	0	0	0
LY86-AS1	0	0	0	0	0	4.16741100615756 (0.00499126294057057)	0	0	0	0
LYAR	0	0	0	0	0	0	0	0	-1.7326561550382 (1.24940396202976e-05)	0
LYL1	0	0	0	0	0	0	0	2.99612236974993 (0.00217537856724147)	0	0
LYN	-2.16454081599635 (0.000565050885423455)	0	-2.49957862913532 (2.96935496535473e-07)	-3.75729180945315 (3.43797012261384e-05)	-2.48952688345806 (0.00253776212124112)	0	0	0	0	-2.11913336515392 (0.00865187299341223)
LYNX1	1.75103744399028 (0.0079363109275401)	0	4.11709414932041 (7.02776277313409e-10)	2.83437996189677 (0.00932671447155594)	0	0	0	0	0	0
LYPD3	0	0	0	0	0	0	0	0	4.05615782306264 (9.46867461504792e-05)	7.12209324231412 (2.01657676204153e-07)
LYPD6	0	0	0	0	0	6.0807486412856 (0.0023280758588174)	0	0	6.71331121579816 (8.65399573166244e-10)	0
LYRM4	0	0	0	0	0	3.25459967934432 (0.00649325990231715)	0	0	0	0
LYRM9	0	0	0	0	0	3.26632049031811 (0.00668648663615243)	0	0	0	0
LYST	0	0	0	0	0	0	0	2.12632866912628 (0.00225654824232548)	0	0
LYVE1	2.31217217605955 (0.000249038501748293)	0	0	0	0	0	0	0	0	0
LYZ	-2.43647159467708 (0.000532768212030143)	0	0	0	0	0	0	2.9437048923491 (0.00399647293783023)	-1.75018887092419 (0.00585184312420856)	0
LZTS1	-2.97138182749767 (0.00137314737326022)	0	0	0	0	0	0	0	0	0
M	0	0	0	0	0	0	23.9088216222878 (1.43705948472522e-23)	0	0	0
MAB21L1	4.57902683570747 (0.00013804466093435)	0	0	0	0	4.95445022330229 (0.00604124699607782)	0	0	0	0
MAB21L2	6.90783313709867 (9.33814831547995e-45)	0	0	7.43739862945766 (1.54490654614943e-23)	0	0	0	0	0	0
MAB21L3	0	0	0	0	0	5.71439955606503 (0.00381313825837107)	0	0	0	0
MACC1	-3.59544201371641 (4.54471642111816e-09)	0	-5.59853632529985 (5.51287652642668e-11)	-3.47266908761542 (0.00205954159479757)	0	0	0	-6.44213362626182 (0.00323944331379583)	0	0
MACF1	0	-3.2425128929435 (5.66946912883974e-05)	0	0	0	-1.50043766550317 (0.00459675282596434)	0	0	0	0
MACROD1	0	0	3.21681590682911 (1.97815872985466e-06)	0	0	0	0	0	0	0
MACROD2	-1.9413847357492 (0.00295114394847936)	0	-4.0352370213849 (3.32417352090249e-06)	0	0	0	0	-5.90447691707575 (0.00944398997524968)	-2.49736055860802 (9.66803647205093e-05)	0
MAEL	0	0	0	0	0	0	0	0	4.69979771947845 (0.000230514675271504)	0
MAF	0	0	-1.52962297663768 (0.00156504217050331)	0	0	0	0	-3.12768274757181 (0.000717727050125771)	0	0
MAF1	0	0	2.23070100848669	0	0	0	0	0	0	0

			(4.07803029088511e-07)							
MAFA	0	0	0	0	5.7800642702998 (0.00591384444719865)	0	0	0	0	0
MAFB	-1.65568058371243 (0.00657777186471775)	0	-2.15767212842514 (0.000271494210468042)	0	0	0	0	0	-1.53518364059366 (0.00109772221287683)	0
MAFF	0	0	0	0	0	0	0	-4.09137174415144 (0.000370282119396381)	0	0
MAGEA10	0	0	0	0	0	0	0	0	6.85584724139081 (4.14114536592822e-08)	0
MAGEA11	0	0	0	0	0	6.34328022616236 (0.00633998907170477)	0	0	4.27572813262956 (0.0011357742164526)	0
MAGEA8	0	0	0	0	0	0	0	0	5.35099362482618 (2.89934343772069e-06)	0
MAGEC1	0	0	0	0	0	7.5017322568852 (0.00343332532783815)	0	0	0	0
MAGI1	0	0	0	0	0	0	0	-4.79708024136032 (0.00413419601216315)	0	0
MAGI2-AS1	0	0	0	0	0	7.07088534283251 (0.009201924440806)	0	0	0	0
MAGI2-AS3	0	0	0	0	0	0	0	-3.02921846640346 (0.00236222267966321)	0	0
MAGI3	0	0	0	0	0	0	0	-3.07933788095545 (0.00479827728257625)	0	0
MAGIX	0	0	0	0	0	4.50491042240694 (0.00992603005342768)	0	0	0	0
MAL	0	0	0	0	5.54664897416149 (8.45118956211153e-09)	0	0	0	0	0
MAL2	-4.30841326328704 (1.26591242735417e-08)	0	-6.01184275463742 (1.80111594369265e-11)	-5.50269736175179 (0.00090639100621783)	0	-2.68741903533294 (0.0026120565281548)	0	-6.88875231963353 (0.00181001049597213)	0	0
MALL	0	0	0	0	0	0	0	0	-2.71427939341221 (0.00233009646882992)	0
MAMDC2	1.51433305418528 (0.000958178974632532)	0	0	2.02920687493669 (0.00882440760305843)	0	0	0	0	-1.73150841180824 (0.00177489461678283)	0
MAML2	0	0	0	0	0	-1.76233753279731 (0.000918378016907215)	0	0	0	0
MAMLD1	-1.65958010967643 (0.00945647508098945)	0	0	0	0	0	0	0	0	0
MAN1A2	0	0	0	0	0	0	0	0	1.87174628565063 (4.03298951402392e-05)	0
MAN1C1	0	0	0	0	0	0	0	0	2.01447235166684 (0.00921543278758595)	0
MAN2A2	0	0	0	0	0	0	0	2.09059818288463 (0.00356021619374247)	0	0
MAN2B1	-1.58652399255478 (0.002940954853166)	0	0	0	0	0	0	0	0	0
MAN2B2	0	0	0	0	0	0	0	-3.51296137574085 (0.00526324215296138)	0	0
MANBA	0	0	-1.72123958101427 (0.000562454003207689)	0	0	0	0	0	0	0
MAOA	0	0	0	0	0	0	0	-6.3064731719774 (6.37282731581981e-05)	0	0
MAOB	2.24065498150533 (4.48551648399457e-06)	0	0	2.94893295143594 (0.000258514657404755)	0	0	0	0	0	0
MAP1A	1.86914038165037 (1.59238792839478e-05)	0	2.2263720346683 (3.44858397907283e-07)	2.42619181100679 (0.00197197876696957)	0	0	0	0	0	0
MAP1B	0	0	0	1.98946876240248 (0.00242600982802758)	0	0	0	-3.23073364506335 (1.18822085266159e-05)	0	0
MAP1LC3A	0	0	2.33901011416143 (0.00193213245897844)	0	0	0	0	0	0	0
MAP1LC3B2	0	5.59359995012975 (5.23342508527029e-05)	0	0	0	0	0	0	0	0
MAP2	-1.76528037521401 (0.000819275377383306)	0	0	0	0	-1.68964897630689 (0.00628768572643202)	0	-8.08792247338263 (0.000191622080054327)	-3.25943666487436 (3.27120654848022e-08)	0
MAP2K3	-1.50961237497063 (0.000360676681495268)	0	0	0	0	0	0	0	0	0
MAP2K7	0	4.03012988741139 (1.01616621200969e-06)	0	0	0	0	0	0	0	0
MAP3K19	-5.06293820310392 (0.000804249224964573)	0	0	0	0	0	0	0	-3.16907427252392 (0.00569626944635545)	0
MAP3K2	0	0	-2.08654563381191 (2.00576164457097e-06)	0	-1.83262614224336 (0.00234184818310954)	0	0	0	0	0
MAP3K6	0	0	0	0	0	0	0	0	-1.88698762953004 (0.00309023800524903)	0
MAP3K8	0	0	-2.00389710176832 (0.000115863804373859)	0	0	0	0	0	0	0
MAP4	0	0	2.00495916758545 (1.7950161796327e-08)	0	0	0	0	-1.77233390615037 (0.00587259263081101)	0	0
MAP6	1.85859240805648 (0.00470480978344706)	0	0	0	0	0	0	0	0	0
MAP7D1	0	0	1.9390373263052 (2.11898926898094e-07)	0	0	0	0	0	0	0
MAP7D2	0	0	0	0	0	0	0	0	4.18798558048984 (2.08684726266409e-05)	0
MAPK13	-1.97566946697429 (0.000248399970723796)	0	-3.31773824364769 (8.18795441410778e-07)	0	0	0	0	0	0	0
MAPK14	0	0	0	0	0	0	0	2.09794923026299 (0.00111641609125213)	0	0
MAPK15	-5.384165148797 (0.000295580208409397)	0	0	0	0	0	0	0	-3.79713638566587 (0.000759396027362707)	0
MAPK4	2.98142171794195 (4.06684829119734e-06)	0	4.94517198532023 (9.37510883665329e-10)	3.82165200468952 (0.00117393370007657)	0	0	0	0	0	0
MAPK6	0	0	-2.23164994499788 (1.70950027848216e-06)	0	0	0	0	0	0	0
MAPK8IP1	0	0	0	0	3.65887258119752 (0.00817892125770589)	0	0	0	0	0
MAPK8IP2	0	0	0	0	4.9245506129995 (0.00403121012080967)	6.0142375964801 (5.14655383230525e-05)	0	0	0	0
MAPKAPK2	0	0	1.96807565158013 (1.24488715622723e-09)	0	0	0	0	0	0	0
MAPKAPK3	0	0	2.69090689038177 (3.35055101117819e-06)	0	0	0	0	0	0	0
MAPRE3	0	0	1.66079500106084 (0.000432863918348837)	0	0	1.85827482470112 (0.000708026140164 68)	0	0	0	0
MAPT	3.48469016754394 (0.000206011106856457)	0	5.44671317341515 (7.02641681356785e-11)	5.71377290182026 (2.17809990850187e-05)	4.21219251611985 (0.0070042745046158)	5.39116490714997 (0.00108577420368861)	3.65474576946971 (0.0054312039809367)	0	3.40749116623413 (0.000517985868690682)	0
MARCH11	0	0	0	0	0	6.03796131297404 (0.0083486374499914)	0	0	0	0
MARCH2	0	0	1.71045396962848 (0.00175703561151767)	0	0	0	0	0	0	0
MARCH7	0	0	0	0	-1.65066387713055 (0.00177841565983549)	0	0	0	0	0
MARCH8	0	0	0	0	0	0	0	2.72547002194467 (3.63827731809281e-05)	0	0
MARCKS	0	0	-1.57670933377408 (0.0055189030425872)	0	0	0	0	0	0	0
MARCO	0	0	0	-3.88534115533507 (0.000778693347627825)	-4.02316846009316 (0.000914041873786732)	0	0	-3.41190156902494 (0.00468286262305454)	-5.03333446845404 (3.99524579775604e-11)	0
MARK2P4	0	0	0	0	6.47559966567641 (0.0042482603748159)	0	0	0	0	0
MARS	0	0	0	0	0	0	0	0	0	-1.8861011538873 (0.00628575704573472)
MARVELD1	0	0	-1.62966858758426 (0.00283498570276905)	0	0	0	0	0	0	0
MARVELD2	-4.95415856587116 (8.45351382122651e-06)	0	-3.15818435563549 (0.000517220035891647)	0	0	1.89203454206146 (0.00702520605357832)	0	0	0	-6.43480958051219 (0.00478816714861936)
MAS1	0	0	0	0	5.29296156662868 (0.000179917339305617)	6.61820748917386 (2.98694908475818e-06)	4.72395905945489 (0.00658335779297519)	0	0	0
MASP1	4.30670881023484 (7.1973235313017e-09)	0	4.08155543892 (9.86492606126561e-15)	0	0	5.68509769042447 (1.21484582308015e-14)	0	0	0	0
MASP2	0	0	0	0	0	4.89853913428198 (2.84998443440768e-11)	0	0	0	0
MAST1	0	0	0	0	0	7.04063115215185 (0.00840552745288383)	0	0	0	0
MAST2	0	0	1.78868894643851 (6.71670679395855e-05)	0	0	0	0	0	0	0
MAST3	0	0	0	0	0	0	0	0	-1.63982809222717 (0.000681451865341541)	0
MAT1A	0	0	0	0	0	9.74180291063101 (4.3791233541779e-20)	0	0	0	0
MATK	0	0	0	0	0	0	0	0	-3.38278207904073 (0.00416478425679104)	0
MATN1-AS1	0	0	0	0	0	4.0797088691644 (0.00360817814886744)	0	0	0	0
MATN2	2.5202395179866 (4.74742775476146e-07)	0	1.65398596859931 (0.000360263712376313)	3.27082103450325 (4.67942081813325e-05)	2.68993813042605 (8.27464163676796e-06)	0	0	0	4.5581586359823 (9.12937821783931e-42)	0
MB	0	0	13.1875475814267 (9.21006787718544e-40)	0	0	6.38968800314438 (0.00475066945748178)	0	7.80721440389322 (2.41763555166414e-05)	0	0
MB21D2	0	0	0	0	0	0	0	0	3.3344424234135 (1.36087450356555e-06)	0
MBD3	0	0	1.69298908634468 (0.00253414607062029)	0	0	0	0	0	0	0

MBIP	0	0	-1.58179507006706 (0.00732719096523495)	0	0	0	0	0	0	0
MBL2	0	0	0	0	0	9.71420682448042 (4.14039853002906e-11)	0	0	0	0
MBNL1-AS1	3.55424876281237 (2.24685172540362e-11)	0	0	0	0	0	0	3.07584752048011 (0.00598386204832757)	2.33286902372258 (0.000577733451078086)	0
MBNL3	-1.93201092934273 (0.000335207009863487)	0	0	0	0	1.54240870544301 (0.00761402180265227)	0	0	4.17602717894818 (1.36449590358398e-13)	0
MBOAT2	0	0	0	0	0	0	0	2.80841439059804 (0.000194264335911222)	0	0
MBP	0	0	0	0	0	0	0	0	-1.83592754503854 (5.58043934444194e-05)	0
MCAM	0	0	0	0	0	0	0	-2.82572083207412 (0.000136492112126436)	0	0
MCCD1	0	0	0	0	0	7.2084256376468 (0.000776470673879134)	0	0	0	0
MCEMP1	-3.97588496697901 (0.000443009416802921)	0	-2.75741583260004 (0.00679041334191895)	0	0	0	0	0	-2.77107572022584 (0.00827523975224325)	0
MCF2L	0	0	2.18123748738569 (4.00794275596263e-06)	0	0	0	0	-3.51379439610459 (0.00954412936744295)	-1.74986920560948 (0.00108813861144009)	0
MCF2L2	2.35447950620069 (0.00508617389246595)	0	0	0	0	5.62662468399611 (5.29369610813896e-05)	0	0	0	0
MCL1	0	-3.70747752442512 (0.00191629623803795)	0	0	0	0	0	0	0	0
MCM10	0	0	0	0	0	0	0	4.65266232377923 (2.83115975472515e-05)	0	0
MCM2	0	0	0	0	0	0	0	3.73317276549277 (0.00082260092319479)	0	0
MCM4	0	0	0	0	0	0	0	3.03991901690665 (0.00100491915592997)	0	0
MCM5	0	0	0	0	0	0	0	3.21779369415551 (0.000649783582035001)	0	0
MCM6	0	0	0	0	0	0	0	1.97624419469123 (0.00941523317548123)	0	0
MCM7	0	0	0	0	0	0	0	2.62259341142115 (0.00199394605863922)	0	0
MCM8	0	4.58152538529043 (3.52106916024182e-06)	0	0	0	0	0	3.24690062774977 (0.000673776860318023)	0	0
MCM9	0	0	-1.65827838057439 (0.00739105659359537)	0	0	0	0	0	0	0
MCOLN2	-4.59769577103601 (0.00135893650791094)	0	0	0	0	0	0	0	0	0
MCPH1	0	3.55064427107545 (7.60124094402894e-05)	0	0	0	0	0	0	0	0
MCRS1	0	0	1.79732709175493 (0.000727268483449583)	0	0	0	0	0	0	0
MCTP2	-2.3415439802633 (3.42173127989732e-06)	0	-2.36808980190589 (0.000127917508411734)	-2.82434393294198 (0.000329221620541053)	-1.61083075912953 (0.00999033016723451)	0	0	0	0	0
MDFI	-3.39400928853054 (0.00271723478978482)	0	0	0	0	0	0	0	0	0
MDGA1	0	0	0	0	0	3.7282641464752 (0.00202234968125694)	0	0	0	0
MDH1	0	0	2.96580990278468 (4.23812702788e-11)	0	0	0	0	0	0	0
MDH2	0	0	2.00449624061424 (1.85904575643454e-07)	0	0	0	0	0	0	0
MDM1	1.75882243701604 (0.000191480702200874)	0	0	0	0	0	0	0	0	0
MDM2	-2.14105152271656 (1.57537760947614e-06)	0	-2.05688412600371 (2.21259364880999e-07)	0	-1.74535816894449 (0.00289830432254993)	0	0	0	0	0
ME1	0	0	0	0	0	0	0	0	-2.13975950063876 (0.000368582399228645)	0
MECOM	0	0	0	0	0	0	0	-2.32355752453583 (0.00504372871572401)	0	0
MED12L	0	0	0	0	3.70225303744067 (0.00974251622096827)	0	0	5.43776364647501 (2.2331765218334e-05)	4.24114402555079 (9.32386882376347e-06)	0
MED25	0	0	0	0	0	0	0	2.10384122706747 (0.0061167710833568)	0	0
MED8	0	2.90758434933881 (0.00322783092599449)	0	0	0	0	0	0	0	0
MED9	0	0	1.72557654983824 (0.00322359170652839)	0	0	3.01540501350566 (0.000493259828146013)	0	0	0	0
MEDAG	0	0	0	2.85997150341207 (9.86416691121242e-05)	0	-2.78589266684175 (0.00861688232665709)	0	0	0	2.81283161937096 (0.0001283391294629)
MEF2A	0	0	0	0	0	-1.7850850847599 (0.00071349320399735)	0	0	0	0
MEFV	-3.08307255394207 (0.000146804521929977)	0	-2.03453742183364 (0.00920094032684314)	0	0	0	0	0	-2.12163268094406 (0.00377959049163502)	0
MEG3	0	0	0	0	0	0	0	0	4.91301650999015 (6.40943343573711e-35)	0
MEG8	0	0	0	0	0	0	0	0	5.30336638871775 (3.58298551215693e-15)	0
MEG9	0	0	0	0	0	0	0	0	4.67706124521751 (0.000387021409667476)	0
MEGF10	5.39674576903012 (0.00057603452003724)	0	0	0	0	0	0	0	0	0
MEGF6	-3.34826314480841 (5.42059695453691e-06)	0	-2.22895623352433 (0.00179758808306043)	0	0	0	0	0	-2.86980021548891 (1.31742750770137e-06)	0
MEGF8	0	0	1.61876792221739 (0.000225047035565802)	0	1.83332819103119 (0.0044057371093412)	0	0	0	0	0
MEGF9	-1.86593114035578 (8.26193116817092e-05)	0	-1.81867691639049 (0.00628583442318004)	-2.48929419259686 (0.00326153181196528)	-1.99149992056898 (0.00757029589064789)	0	0	0	-3.11732870306268 (2.1919301684594e-11)	0
MEIS1	0	0	-1.77299861024053 (3.74934328216789e-05)	0	0	0	0	-3.37097133926923 (0.000332043368269945)	0	-2.86201278949822 (0.000129754012768768)
MEIS2	2.91572905843517 (9.35660496010395e-14)	0	0	2.46022296442932 (0.00330720575010473)	1.86057859278361 (0.00347702340439031)	2.05715293251087 (0.00232348067415402)	0	0	0	0
MEIS3	2.5410551673509 (7.46563433459737e-06)	0	0	0	0	3.0065497003165 (0.00198237684985776)	0	0	0	0
MEOX1	0	0	6.3406902988603 (1.10024384557677e-10)	0	0	4.37676549349558 (0.00583185885624123)	0	0	0	0
MEOX2	-1.80614732836993 (0.00298952912421306)	0	0	0	0	-4.36436560039588 (0.000172540311611952)	0	-6.73532347419005 (0.00277594236784103)	0	0
MERTK	0	0	-1.97753762141484 (0.00269561532135131)	0	0	0	0	0	0	0
MESDC1	-1.62372753467086 (0.00123890748807726)	0	0	0	0	0	0	0	0	0
MEST	-2.25034305841741 (3.51112481538982e-06)	0	0	0	0	0	0	0	3.29404083236964 (4.14181705872648e-08)	-2.73657709258247 (0.00459816307918178)
MET	0	0	0	0	0	0	0	-4.70177438558606 (0.00862315250721886)	0	0
METAP2	0	0	0	0	0	0	0	1.95689114714405 (0.00157082130338857)	0	0
METTL1	0	3.98370795007934 (0.00649580021492226)	0	0	0	0	0	0	0	0
METTL12	0	0	0	0	0	0	1.57190131659699 (0.00145346791304143)	0	1.62238598583187 (0.000622575561945701)	0
METTL17	0	0	0	0	0	0	0	2.3254199030039 (0.00482686290453848)	0	0
METTL24	6.51447049294715 (9.86286111068001e-09)	0	0	7.27245160959529 (0.000308198984895893)	0	0	4.17579300905613 (0.000208479735780496)	0	0	0
METTL7A	0	0	0	0	0	0	0	0	-1.70686498636391 (4.24528166372207e-05)	0
METTL7B	0	0	0	0	0	3.7978254416351 (0.000237250406643199)	0	0	0	0
MEX3B	0	0	0	0	3.09855207644346 (0.00761720663577483)	0	0	0	0	0
MEX3D	0	0	0	0	0	0	0	0	1.81023641168812 (0.00847238124811385)	0
MFAP2	0	0	0	0	0	0	0	0	1.6328629624878 (0.00124402159844604)	0
MFAP3	0	0	0	0	-3.30702537212092 (0.00827202577832234)	0	0	0	0	0
MFAP3L	0	0	0	0	0	4.20930013722972 (6.23638856904725e-06)	0	0	2.49584892716823 (8.48719660172548e-05)	0
MFAP4	0	0	0	0	0	0	0	-8.3734254277014 (0.000125801180709363)	0	0
MFAP5	3.49177307351822 (0.000383323350745682)	0	3.54861343684155 (2.67950244712008e-05)	4.18311145491448 (0.00165863474406471)	0	0	0	0	6.36319451386904 (1.90934518911407e-24)	5.01378377393741 (1.39894418875405e-05)
MFGE8	0	0	1.78878145262581 (9.00849350696e-08)	0	0	0	0	-2.54343953398731 (0.00187898050046638)	0	0
MFI2	0	0	0	0	3.70781516142291 (4.81416229193156e-05)	3.37966127469168 (0.000163265713305109)	0	0	0	0
MFN2	0	0	2.77705412549112 (2.93474587212399e-10)	0	0	0	0	0	0	0
MFSD1	0	0	-2.20769246470655 (0.000587133859827206)	0	-2.94388534217201 (0.00473853275949072)	-2.21695073683498 (0.00851909950588112)	0	0	0	0
MFSD2A	-3.37857128310896 (3.64604096725599e-05)	0	-5.10804267324269 (4.69610285993108e-08)	0	0	0	0	0	0	0
MFSD2B	0	0	0	0	0	4.58345994147837 (0.00997690284200504)	0	0	0	0
MFSD3	0	0	3.7264773129455	0	0	0	0	0	0	0

			(0.00668010979534492)							
MFSD6	0	0	0	0	-2.00751092744628 (0.00533796742132365)	-2.82919366138816 (0.000160452318410599)	0	0	0	0
MGAM	-2.7288423094217 (0.000734915574100666)	0	0	0	0	0	0	3.22303667241739 (0.00167154061792709)	-2.23544517068145 (0.00196756698389573)	0
MGAT3	0	0	0	0	0	3.41731857373415 (0.00562862113171792)	2.21948650351841 (0.000757869614720908)	0	2.77801793504854 (4.78318594099952e-07)	0
MGAT4B	0	0	0	0	0	1.7314534641478 (0.00209533744095567)	0	0	0	0
MGAT4C	0	0	0	0	0	6.34162684082331 (0.000104302569957244)	0	0	0	0
MGC27382	0	0	0	0	0	0	0	0	-4.40419531547529 (0.000478925594291347)	0
MGP	2.05785967535629 (1.02555979522589e-07)	0	0	1.68169267507649 (0.00443391374285902)	2.02540891417103 (4.3303693433887e-05)	0	0	-2.80826365146699 (5.41865064683478e-05)	0	0
MGST2	0	0	2.19325040502273 (0.00033978798668741)	0	0	2.50696000116922 (0.00449369071109878)	1.50861368974101 (0.00285545283192957)	0	2.18942853809556 (0.00101704651331652)	0
MIAT	-2.4270678673141 (0.000470945335189411)	0	-2.85732554204151 (0.000983181365481082)	0	0	0	0	0	0	0
MICA	0	0	0	0	0	0	0	0	-1.69113920929534 (0.000397957476729203)	0
MICAL1	-1.89167002323542 (3.19057912412988e-06)	0	-1.63141282346126 (0.000827654463364764)	-2.6999984521139 (0.00126278577053656)	-2.42546202744942 (0.00148513287821379)	0	0	0	0	0
MICAL3	0	0	1.61796579779213 (1.35872746158282e-05)	2.05378874693126 (0.00835210595928947)	0	1.63325416142107 (0.00208314928216341)	0	0	0	0
MICALCL	-3.14507729693387 (0.00347114496421048)	0	0	0	0	0	0	0	0	0
MICALL2	0	0	0	0	0	0	0	0	-2.48227535809208 (0.000965478987392356)	0
MICB	-3.29426590203825 (7.61444873068761e-05)	0	-2.30881539494069 (0.00561793455679343)	0	0	0	0	0	-2.45285566472883 (5.74040525305685e-05)	0
MICU3	0	0	0	0	0	0	0	0	2.58273801311414 (0.000169634689960789)	0
MID1IP1	0	0	0	0	0	0	0	0	-2.14757325787285 (4.29892439658937e-09)	0
MIEF2	0	0	1.92802347002941 (0.00856702751540103)	0	0	0	0	0	0	0
MINPP1	0	0	0	0	0	0	0	3.3856994640188 (4.34157773432039e-05)	0	0
MIR1-1HG	0	0	5.91241799737715 (1.46554164887358e-06)	0	0	0	0	0	0	0
MIR100HG	3.14984939464085 (0.00259236063711336)	0	0	0	0	0	2.34460866043615 (0.00612997575967532)	0	3.81093466244173 (4.62383820859267e-05)	0
MIR143HG	2.51066540996168 (1.46888291287517e-06)	0	0	0	0	0	0	-3.73990126675944 (0.00018883197559129)	0	0
MIR181A1HG	-4.6466808339748 (5.9038259170716e-05)	0	0	0	0	0	0	0	0	0
MIR205HG	0	0	0	0	0	5.94479879595656 (0.000835627152238052)	5.13921903331473 (2.48974810884947e-05)	0	6.02294430334138 (6.33031130805974e-12)	7.94092644055257 (1.57038973899536e-12)
MIR222HG	-2.22496226457125 (0.00149228381069031)	0	-2.61732750394186 (0.000162384687692665)	0	0	0	0	0	0	0
MIR3180-4	0	0	0	0	6.64816874282679 (0.00347040281013931)	0	0	0	0	0
MIR4435-1HG	-2.10710012135926 (0.000105949477227307)	0	0	0	0	0	0	0	0	0
MIR4449	0	0	4.26843772139914 (0.00585288694610003)	0	0	0	0	0	0	0
MIR4697HG	2.62485103511919 (3.80615428862824e-05)	0	0	3.59229176450665 (0.00158757099848594)	0	0	0	0	0	0
MIR497HG	0	0	0	0	0	4.04358264741629 (0.00302599212631188)	0	0	0	0
MIR503HG	0	0	0	0	0	0	0	0	5.73064006208305 (4.34647179692186e-08)	0
MIR600HG	0	0	0	0	0	3.50030759503373 (0.00246798846210361)	0	0	0	0
MIR663AHG	0	0	4.01278867278198 (0.0041521469352587)	0	5.71595982092029 (8.82827335914506e-05)	4.83121425245851 (0.000525556868624972)	0	0	0	0
MIR7515HG	0	0	0	0	6.62597406094585 (0.000531252611214814)	0	0	0	0	0
MIR7641-1	0	0	0	0	0	3.46476185363674 (0.00262325183017638)	0	0	0	0
MIS18BP1	0	0	0	0	0	0	0	2.50075832714757 (0.000257661135383838)	0	0
MKI67	-2.2436751160063 (0.000842776486711447)	0	0	0	0	0	0	4.87047091312794 (2.22639960813098e-07)	0	0
MKNK2	0	0	0	0	0	0	0	0	-1.82314139819268 (5.4264880727859e-06)	0
MKRN1	0	0	0	0	0	0	0	1.71304526470454 (0.00879362600844305)	0	0
MKRN4P	0	0	0	0	0	0	0	0	6.42991703675528 (2.19846503667393e-08)	0
MLC1	0	0	0	0	0	0	0	5.54715311521855 (7.40677896842841e-05)	0	0
MLEC	0	0	1.54615263532152 (9.37997421958507e-07)	0	0	0	0	0	0	0
MLF1	0	0	2.37372377502927 (0.000265362176535123)	0	0	0	0	0	0	0
MLIP	0	0	10.0431957945061 (1.13714958708567e-23)	0	0	7.33094840939382 (0.000245332543087037)	0	0	0	0
MLIP-AS1	0	0	5.7533083376952 (3.79568668066278e-05)	0	0	0	0	0	0	0
MLK4	0	0	-3.21646611789938 (0.00766403436874655)	0	0	0	0	0	-2.62464402139291 (0.00136084350359688)	0
MLLT11	0	0	1.73054793341525 (0.00160142652873068)	0	0	0	0	0	0	0
MLLT4-AS1	0	0	0	0	4.59793061760977 (0.00878071645461982)	4.78322903936679 (0.00594734208578031)	0	0	0	0
MLPH	-4.90402925168472 (7.46462356180484e-11)	0	0	-3.67367008715119 (0.00148162507372651)	0	0	0	0	0	0
MLXIPL	0	0	0	0	0	6.50166226598939 (1.64364957966169e-16)	0	0	0	0
MMAB	0	0	0	0	0	2.34267587075717 (0.00636833378845049)	0	0	0	0
MME	-3.87339491939283 (0.00194947807232126)	0	-2.57044679124885 (0.00152432938208404)	0	0	0	0	0	2.24820083013167 (0.000512378615912789)	0
MME-AS1	0	0	0	0	0	0	0	0	3.76643540642723 (0.00489686356709262)	0
MMP10	0	0	0	0	0	0	4.62395712181849 (0.00235032517766631)	0	0	0
MMP11	0	0	0	0	6.24434293510046 (0.0075784156376657)	0	0	0	8.57050347767403 (2.34222056243989e-13)	0
MMP12	0	0	0	0	0	0	0	0	4.27178597930829 (0.00521945863083008)	0
MMP14	0	0	-2.3271303741418 (4.75802710159129e-07)	-1.94604823345978 (0.00859071970358865)	-2.10395450059301 (0.000321115340370772)	-2.52162893483172 (1.34339607874859e-06)	0	-3.44358120395842 (2.94872342962978e-05)	0	0
MMP15	0	3.95128770968508 (0.00118861823971311)	3.10161450578613 (1.85632754079664e-08)	0	0	2.48976761851338 (0.00442798719890069)	0	0	2.75213535014618 (2.38052608879166e-05)	0
MMP16	2.04658029386063 (0.00780461063555548)	0	0	0	0	0	0	0	2.20435498066102 (0.000116708858650662)	0
MMP19	-2.9612312085011 (2.0536897673504e-07)	0	-3.62577899379306 (4.34706768715791e-07)	-2.75977090849575 (0.00168112896173838)	-2.272754741269 (0.00301010937201066)	0	0	0	0	0
MMP2	0	-4.90090703169448 (0.00305639703657918)	-1.81971685502823 (0.000191120907703383)	0	-1.83475100178609 (0.0076065968333316)	-3.16610415767444 (9.0728309633892e-05)	0	-5.05123608385785 (1.72964518630061e-07)	0	0
MMP24	0	0	0	0	0	0	0	0	-2.54482744664049 (0.0013800376796688)	0
MMP24-AS1	0	0	0	0	0	0	0	0	-1.66427269677945 (5.05657618051621e-05)	0
MMP25	-4.21038315747007 (0.000128023472560475)	0	0	0	0	0	0	0	-4.17175055327171 (1.04000723717607e-06)	-6.08807438417929 (0.00857207641178079)
MMP25-AS1	0	0	0	0	0	0	0	2.48518581363728 (0.00758521214701506)	0	0
MMP8	-3.80569716245559 (0.000931697074079024)	0	0	0	0	0	0	5.9391201564814 (0.0039251873928251)	-5.35230774283393 (4.08865892259681e-06)	0
MMP9	0	0	0	0	0	0	0	0	-3.08317247826594 (0.00345555042546609)	0
MMRN2	0	0	2.58409731097533 (2.62859231829241e-09)	0	2.04955594729718 (0.000800135703827465)	0	0	0	0	0
MMS22L	0	0	0	0	0	0	0	2.87279641911201 (0.00103585234007846)	0	0
MN1	0	0	0	2.8986987559572 (0.00064857324144509)	0	0	0	0	0	0
MND1	0	0	0	0	0	0	0	5.690638575243 (0.000141521370170246)	0	0
MNDA	-2.6676120324316 (4.63578626504206e-06)	0	0	-2.6301433737075 (0.00980093778123823)	0	0	0	2.6890609831306 (0.00164233204262079)	0	0
MOAP1	1.53056023142495 (0.000511924701824051)	0	0	0	0	0	0	0	0	0
MOB2	1.61740389320784 (0.00335745823000198)	0	0	0	0	0	0	0	0	0
MOBP	0	0	0	0	0	7.79735332433261 (5.09981959120149e-06)	0	0	0	0

MOCOS	0	0	0	0	0	3.10355671626099 (7.56769994351382e-06)	0	0	0	0
MOCS1	0	0	0	0	0	0	0	-6.69291251310009 (0.00331530677818532)	0	0
MOCS3	2.92915859892162 (0.00757175232811919)	0	0	0	0	4.49002321981394 (0.00176034758661768)	0	0	2.70987883243768 (0.00307435344359256)	0
MOGAT2	0	0	0	0	0	7.92879085336449 (9.76366103903795e-12)	0	0	0	0
MOGAT3	0	0	0	0	0	6.07777327913293 (0.00540910550509002)	0	0	0	0
MON1B	0	2.52561113371 (0.00774073796891231)	0	0	0	0	0	0	0	0
MORC4	0	0	0	0	0	0	0	-6.00060553462978 (0.00656295284479736)	2.68797845462389 (7.90490701715691e-08)	0
MOV10L1	0	0	0	0	0	5.11250836846822 (0.00118327620648364)	0	0	3.56516887390385 (0.00289972600184789)	0
MOXD1	0	0	-3.39472154884383 (4.84216031998415e-05)	0	0	0	0	-6.69486859532343 (0.00278631187452944)	-3.6674588475062 (1.23690857337134e-08)	-3.00454870435022 (0.00484992842069474)
MPC1	0	0	2.17060318543036 (8.89056653329462e-06)	0	0	2.29489664989544 (0.00401941355434518)	0	0	0	0
MPDZ	0	0	0	0	0	0	0	-3.95711252480116 (0.000263708597986024)	0	0
MPEG1	0	0	-3.79452054121021 (1.60846264327945e-05)	0	0	0	0	0	0	0
MPHOSPH9	0	0	0	0	0	0	0	2.1409850125727 (0.00554676560708124)	0	0
MPL	0	0	0	0	0	5.26259698727279 (0.00243624477040353)	0	0	0	0
MPLKIP	0	0	0	0	0	2.37105532337132 (0.00382043864299273)	0	0	0	0
MPO	-4.2323825652007 (0.00589980694840008)	0	0	-6.98923445516886 (0.00375045959878677)	0	0	0	6.9562508725256 (0.000122968870389165)	-5.47908023037483 (3.17922937753963e-07)	0
MPP1	0	0	-2.2584968808989 (8.69772336753006e-06)	0	0	0	0	2.19140223866978 (0.00241013991730452)	0	0
MPP2	2.79811765176681 (0.000315472076224651)	0	2.90784179716165 (0.00100689362717849)	4.0596372937187 (0.00505147198712596)	3.59739088828464 (0.00132583124040848)	0	0	0	0	0
MPP3	0	0	0	0	0	0	0	0	-2.77266252995745 (0.000240925553694165)	0
MPPED1	0	0	0	0	6.24434293510046 (0.0075784156376657)	7.15853591877615 (2.87351278105053e-06)	0	0	0	0
MPPED2	3.56836705497174 (9.24261854615804e-05)	0	0	0	0	4.85423392670703 (0.000551328659053901)	0	0	0	0
MPST	0	0	0	0	0	2.19826451965809 (8.72406126739749e-06)	0	0	0	0
MPV17L	2.7801396681754 (0.00146549316850242)	0	2.49188147437887 (0.00439535133450215)	0	3.9940303977947 (0.000634289218314471)	3.90931206131502 (0.00137502849380146)	0	0	0	0
MPZL2	-2.18573627877418 (8.87103663051575e-05)	0	-1.95068632857291 (0.000193065223927376)	0	0	0	0	-7.58430559482495 (0.000597666615052172)	0	0
MPZL3	-2.95762873237961 (0.000222616753097901)	0	-2.46150660966827 (0.00399369684293423)	0	0	0	0	0	0	0
MR1	0	0	-2.25690586228028 (0.000172954631940001)	0	0	0	0	0	0	0
MRAS	0	0	2.15052005745768 (1.39757262655293e-06)	0	0	0	0	0	0	0
MRC1	0	-6.94861199477391 (0.00783888782135077)	-2.74889068303384 (0.00406621952767586)	0	-2.53788174974762 (0.00395344807964931)	-2.65180594901273 (0.00101322363827828)	0	-4.6581897539244 (3.98634498662121e-05)	0	0
MRC2	-1.95797322403774 (9.8043776161949e-08)	0	0	-1.84447106310448 (0.00996804543372055)	0	0	0	-4.6457546535277 (0.000156969328502659)	-2.39395439394925 (1.92425435955009e-06)	0
MREG	-3.62201576829147 (0.00191153146554889)	0	-3.87982350516346 (0.00234859236750468)	0	0	0	0	0	0	0
MRGPRF	2.91309360901147 (3.24300467224534e-06)	4.21333943772874 (0.00208235499802207)	0	3.4356102548723 (0.00291597739042254)	0	0	0	0	0	0
MRO	0	0	0	0	0	3.8856120388098 (0.00288196682055075)	0	0	0	0
MROH1	-1.94238188639088 (6.2340800867535e-05)	0	0	0	0	0	0	0	0	0
MROH7	3.85276867452449 (0.00118180439486492)	0	0	0	0	0	0	0	0	0
MROH8	0	0	3.6211068517673 (0.00140712693640941)	0	0	0	0	0	0	0
MRPL12	0	0	3.17951188932007 (0.00885894098491575)	0	0	0	0	0	0	0
MRPL15	0	0	1.50500293700912 (0.00123525556576776)	0	0	0	0	0	0	0
MRPL2	0	0	0	0	2.32100461279539 (0.00492255803709144)	2.40958603969975 (0.00362367932904232)	0	0	0	0
MRPL4	0	0	0	0	0	0	0	0	-1.802363327626 (0.00677219974945212)	0
MRPL41	0	0	3.21947076167323 (3.51780761871744e-09)	0	0	1.87102826713827 (0.00326385362092672)	0	0	0	0
MRPL54	0	0	0	0	0	2.58685120872656 (0.00333275677372709)	0	0	0	0
MRPS17	1.53293914227204 (0.00420422657146042)	0	0	0	0	0	0	0	0	0
MRPS17P1	0	0	0	0	0	7.14081427305719 (0.000147969425382988)	0	0	0	0
MRPS21	1.52983072967644 (0.00070737963435667)	0	0	0	0	0	0	0	0	0
MRPS23	0	3.33822327731828 (0.000152362225713278)	1.98726579366501 (2.64108279787408e-06)	0	0	0	0	0	0	0
MRPS27	0	2.56592344575208 (0.00439807909527621)	0	0	0	0	0	0	0	0
MRPS34	0	0	0	0	0	0	0	0	-1.62687100611843 (0.00122405140497642)	0
MRPS7	0	0	1.6745340772131 (0.000473599844438554)	0	0	1.64650441550335 (0.00650024406868966)	0	0	0	0
MRVI1	2.85064875344914 (1.4647690383656e-08)	0	0	2.66113046745761 (0.00241375303812484)	0	0	0	0	0	0
MS4A15	0	0	0	0	4.81072558245872 (0.00897719021672)	0	3.41897507325668 (0.000644153221164748)	0	0	0
MS4A3	0	0	0	0	0	0	0	6.65166831709763 (1.99709040009454e-10)	0	0
MS4A4A	0	0	-2.64134611575557 (0.0036502397325952)	0	0	0	0	0	0	0
MS4A7	-1.87615636332238 (0.00983447344916295)	0	-2.88206641108925 (0.00127045795675441)	0	0	0	0	0	0	0
MSANTD4	0	2.44738102982632 (0.00853032966505654)	0	0	0	0	0	0	0	0
MSC	0	0	2.2175274979644 (0.00158547162597712)	0	0	0	0	0	0	0
MSH2	0	0	0	0	0	0	0	2.54096618394106 (0.00764209378104305)	0	0
MSH5-SAPCD1	0	6.98114786041157 (4.99752337791705e-05)	0	0	0	0	0	0	0	0
MSI1	0	0	4.45246886582008 (0.00561761564313214)	0	0	5.49413263303224 (0.00181830173244257)	0	0	0	0
MSLN	-2.8971416650528 (0.000723415631343304)	0	-4.10762619848529 (0.000178811797121846)	0	0	0	0	0	0	-6.01184395951951 (0.00940369457857304)
MSMO1	-2.96956013544895 (0.000167936682630392)	0	-2.76014304047726 (0.00039841379839744)	0	0	0	0	0	-2.57066803758425 (5.1870425487726e-05)	0
MSN	0	0	0	0	0	-1.56462197500195 (0.00352968776770389)	0	0	-1.72810136827917 (3.99238093051186e-13)	0
MSR1	0	0	-3.62834568203111 (0.0011363040737695)	0	0	-2.38039593767303 (0.00953928591316698)	0	-5.37037485762932 (0.000255975763939434)	0	0
MSRA	0	0	1.82071618901452 (0.00246747263755987)	0	0	2.6518432017796 (0.00181862601912154)	0	0	0	0
MSRB2	0	0	1.73771153865149 (0.00235832424915143)	0	0	0	0	0	0	0
MSRB3	1.71048019057029 (3.53209811085658e-05)	0	0	2.12468049903017 (0.00440993015654276)	0	0	0	0	0	0
MSS51	0	0	4.01613489065921 (0.000473585782605581)	0	0	0	0	0	0	0
MST1	0	0	0	0	0	5.49020947657212 (4.24884106563633e-12)	0	0	0	0
MST1P2	0	0	0	0	0	4.6736801191118 (0.000290833145712496)	0	0	0	0
MST1R	-4.57093230272633 (0.00450225309248782)	0	0	0	0	0	0	0	0	0
MSX1	0	0	3.23532123553311 (0.00199345232005256)	0	0	0	0	0	0	0
MSX2	0	0	0	0	0	0	0	0	3.70104797903685 (3.24485625587677e-05)	0
MT-CO1	0	0	0	0	2.33735376577842 (0.00306982412971056)	1.65045853815199 (0.00921494037688706)	0	0	0	0
MT-CO2	0	0	0	0	2.30466577375729 (0.00209635362172835)	1.7656585353896 (0.00611306049904821)	0	0	0	0
MT-CYB	0	0	0	0	2.79720583606717 (0.000505134850538658)	2.41458632545125 (0.0012392203262677)	0	0	0	0
MT-ND1	0	0	0	0	2.19299792238531 (0.00575556876421897)	0	0	0	0	0
MT-ND2	0	0	0	0	2.13136145130904	0	0	0	0	0

					(0.00906025187215933)					
MT-ND3	0	0	1.59334357439902 (0.00824065158139915)	0	2.50122163038202 (0.00190057161897539)	2.49231257721227 (0.00120659792469767)	0	0	0	0
MT-ND4	0	0	0	0	0	1.75093833548956 (0.00980573336017166)	0	0	0	0
MT-ND4L	0	0	0	0	2.53134998555589 (0.000870816499849)	2.48655637677202 (0.000500608278550518)	0	0	0	0
MT-ND5	0	0	0	0	2.82861665703069 (0.000167144647574193)	0	0	0	0	0
MT-ND6	0	0	0	0	2.71641605076745 (0.000883328204732991)	3.07809048065121 (0.000896499792044846)	0	0	0	0
MT-RNR1	0	0	2.10132419716807 (0.000853260298034383)	0	0	0	0	0	0	0
MT-RNR2	0	0	1.93815713967401 (0.00134620815831594)	0	2.25016216399611 (0.00815518662655286)	2.2625073565428 (0.00093506491454984)	0	0	0	0
MT-TA	0	0	4.35201400801465 (0.00225882741697205)	0	0	0	0	0	0	0
MT-TC	0	0	3.87135163585492 (6.59094710668593e-06)	0	5.08329294586537 (3.72417239924489e-07)	4.51363764745024 (6.82391996654845e-06)	0	0	0	0
MT-TD	0	0	3.47846862771742 (0.000400644062416125)	0	4.98092864668408 (1.2317682882864e-05)	0	0	0	0	0
MT-TF	0	0	2.69686067547391 (0.00176592736639048)	-6.86841855293039 (0.00521927870315078)	0	2.72780959524009 (0.00261424348315152)	0	0	0	0
MT-TG	0	0	4.16244419154332 (0.00113841215473364)	0	0	3.5984441328285 (0.00221328128187032)	0	0	0	0
MT-TH	0	0	4.81050216846423 (6.60771243537356e-06)	0	3.28753872501068 (0.00501705555138226)	4.16171294855007 (0.000694789100201408)	0	0	0	0
MT-TI	0	0	4.95573672027144 (1.70117226579182e-06)	0	5.49123100584846 (1.54783057809903e-05)	6.04084704654531 (1.23870366556014e-07)	2.42749766436775 (0.00519494567158114)	0	2.76914250375468 (0.00204234006876579)	0
MT-TL1	0	0	3.0073426385128 (5.60688415359877e-05)	0	2.63046013748441 (0.0060815567199196)	3.40428795685812 (0.000360386208747476)	0	0	0	0
MT-TL2	0	0	4.15952897789978 (0.00371521569271631)	0	0	0	0	0	0	0
MT-TM	0	0	3.74884970780563 (0.000778183481531105)	0	2.96498763441812 (0.00498368167662151)	4.15895998342588 (4.93705785054788e-05)	0	0	0	0
MT-TP	0	0	4.54406608364417 (0.000192758432962502)	0	4.96718579882853 (3.24405978330152e-05)	5.22072996149226 (4.70123054943681e-07)	0	0	0	0
MT-TQ	0	0	4.32166986065615 (0.00267989234798225)	0	0	0	0	0	0	0
MT-TR	0	0	5.00200997925972 (2.73283244177021e-05)	0	0	0	0	0	0	0
MT-TS2	0	0	3.658774822163 (4.42587204869701e-07)	0	4.02478893904899 (1.5740500249416e-05)	4.04420894623042 (1.23100315316459e-06)	0	0	0	0
MT-TT	0	0	3.83063906711459 (0.00174344652728477)	0	0	0	0	0	0	-6.94980503068468 (0.0061586987390931)
MT-TV	0	0	2.62285293909327 (0.000260652882299786)	0	0	3.20965523433695 (0.000597182057230903)	0	0	0	-3.33644255043752 (0.00669656228139692)
MT-TY	0	0	4.57631834980606 (2.18475165976129e-05)	0	6.15547434739355 (4.76970052599301e-05)	4.89926642601744 (0.000111088485251864)	0	0	0	0
MT1E	0	0	0	0	0	2.54677220882432 (0.000475267102426145)	0	-6.28559252171833 (0.00529431859168007)	0	0
MT1F	0	5.1100660773862 (0.000979641966927697)	0	0	3.4735978419796 (0.00501110850779349)	5.69646151755191 (1.7036521074692e-12)	0	0	0	0
MT1G	0	0	5.80802402733033 (0.00725910151022307)	0	4.48106651530506 (0.00687169570089449)	8.47522111292453 (2.36676251037551e-10)	0	0	0	0
MT1H	0	0	0	0	5.43397361388775 (0.00995162242902884)	8.63198722238389 (2.39638905472565e-11)	0	0	0	0
MT1L	0	0	0	0	0	3.15966097746829 (0.000401408843602165)	0	0	0	0
MT1M	0	0	0	0	0	3.24767310777189 (3.70667020807193e-05)	0	0	-2.86197075520138 (0.00943991289216466)	0
MT1X	0	0	0	-3.42903928369569 (0.00072365852711987)	0	0	0	-7.39821621915822 (0.000818262705654604)	-3.5677494717636 (7.25790097545495e-08)	0
MT2A	0	0	0	0	0	1.57696991816049 (0.0060433418155207)	0	-2.45615102112885 (0.00392811948758189)	-1.73287809274412 (0.000777458014045728)	0
MTA3	0	0	0	0	0	1.69764571782332 (0.00872160985720737)	0	0	0	0
MTCL1	2.65798145491783 (5.05784869058175e-06)	0	0	2.93270368361265 (0.00612642159097319)	0	0	0	0	0	0
MTFR1L	0	0	2.91238658198601 (6.15585149484527e-10)	0	0	0	0	0	0	0
MTG1	-3.80532719387272 (0.00325674503525197)	0	0	0	0	0	0	0	0	0
MTHFD1P1	0	0	0	0	4.31723732317998 (0.00503526911963355)	0	0	0	0	0
MTHFR	0	0	0	0	0	0	0	0	-1.61342472173156 (0.000440860022729293)	0
MTL5	0	0	0	0	0	4.99908981314445 (0.0029984442961532)	0	0	0	0
MTMR2	0	0	0	0	0	0	0	-3.50430673986619 (0.00482682150518368)	0	0
MTMR4	0	0	0	0	0	0	0	0	1.73320239854372 (0.00160014670174304)	0
MTND1P23	0	-18.7336194787382 (5.2800471938712e-05)	0	0	0	0	0	0	0	0
MTND2P28	0	0	0	0	2.37171069885828 (0.00321478330058353)	0	0	0	0	0
MTND6P4	0	0	0	0	3.90794817079195 (4.36320112693961e-06)	0	0	0	0	0
MTRF1	0	4.43304777396115 (0.000276168101367448)	0	0	0	0	0	0	0	0
MTRF1L	0	0	0	0	0	0	0	0	-1.68016538831247 (0.00027790296744618)	0
MTRNR2L1	0	0	4.03454569914072 (2.62205809435404e-05)	0	4.27945599828092 (0.000758869849540597)	4.6135746338835 (1.1592598210091e-06)	0	0	0	0
MTRNR2L11	0	0	0	0	7.23691766069691 (7.79355797959052e-05)	0	0	0	0	0
MTRNR2L12	0	0	2.98073375846225 (3.95917860836971e-05)	0	2.9403998931593 (0.000902766932609761)	2.9391285281547 (0.000115873589900339)	0	0	0	0
MTRNR2L3	0	0	3.90321222671675 (0.000210760527429583)	0	0	4.46731498552211 (0.00107029954024829)	0	0	0	0
MTRNR2L8	0	0	4.19833857582422 (0.00015369389815508)	0	0	0	0	0	0	0
MTRR	0	0	0	0	0	0	0	-6.34916501174072 (0.00441338624557262)	0	0
MTSS1	0	0	0	0	0	0	0	-2.29793862660182 (0.00611770169748731)	0	0
MTTP	5.18369588285384 (0.00143836250218705)	0	5.5399034733207 (0.000866791787721395)	0	0	7.86653561119205 (1.39507914035989e-08)	0	0	0	0
MTURN	0	0	0	1.98663427605302 (0.00419666385509389)	0	0	0	0	0	0
MTUS1	0	0	0	0	0	0	0	-3.18819212458164 (0.000167391505142483)	0	0
MTUS2	4.51599855778273 (1.63239141376222e-05)	0	8.02102618909181 (5.72798505451601e-17)	0	0	0	0	0	0	0
MTUS2-AS1	0	0	0	0	6.798559966251 (0.0016583499845375)	0	0	0	0	0
MUC1	-3.81903928761105 (0.000112272105384558)	-6.04311743174055 (0.00734178517257527)	-6.5777671248706 (6.04135642467909e-17)	-7.10192565936487 (3.65989077127521e-07)	0	-4.18785568951291 (4.92702309103581e-06)	0	-3.13753632210255 (0.00247175757215813)	-3.4368922255936 (0.000579863536653487)	-4.13437143056865 (4.28800954030079e-05)
MUC12	0	0	0	0	4.23078589458736 (0.00329583033710312)	6.27414796165798 (2.12350394183817e-05)	0	0	0	0
MUC13	3.84143425711388 (0.000123304706373776)	0	0	0	0	4.14520191543937 (0.00487524814674251)	0	0	0	0
MUC15	-4.59540675608182 (0.00241427456090776)	0	0	0	0	0	0	0	3.73955315149509 (3.22530850177487e-07)	0
MUC16	0	0	-2.94040066536801 (0.00177508404611606)	0	0	0	0	0	4.03204552046644 (0.000524512627526668)	0
MUC17	7.9940545893671 (8.89374747651603e-12)	0	0	9.56908189062433 (9.36951908257645e-13)	6.65548282265748 (0.000250159305027331)	6.82242222759498 (0.00221457039205031)	0	0	0	0
MUC19	0	0	0	0	4.2677223376247 (0.000771732596968332)	4.42540053280231 (0.00755317851750998)	0	0	0	0
MUC2	7.22672189998874 (2.92138553065496e-13)	0	0	7.90293067108717 (2.92664646738018e-12)	0	0	0	0	0	0
MUC20	0	0	0	0	0	2.75005629917045 (0.000559513395312156)	0	0	0	0
MUC21	-4.98479328985596 (0.00142168467137102)	0	0	0	0	0	0	0	-5.35283842857229 (0.000135605279803649)	0
MUC3A	2.63092277909425 (1.1582616744509e-07)	0	4.66747013453206 (1.89821761692237e-05)	4.0570354568212 (5.93673543647845e-06)	0	4.77371257880732 (2.49410952633303e-05)	2.51592459848649 (0.00114701575431732)	0	0	0
MUC4	-5.66260213124997 (7.64531455414024e-09)	0	-7.24199489620643 (1.27916075156786e-15)	-6.3661702659038 (0.00143961201823363)	0	0	0	-9.74915418889207 (0.000646695181094512)	-4.93233344010701 (3.93140147227522e-07)	-5.0592460322059 (0.00795737209871209)
MUC5AC	-4.10847797340391 (0.000572843644787636)	0	-5.03578509198685 (0.000109713537976975)	0	0	0	0	0	-6.42783205796172 (9.43358832776372e-08)	0
MUC5B	-4.33495495778229 (7.64182369448656e-09)	0	0	-4.75545859007963 (0.000734181439303662)	0	0	0	-7.14028209630741 (0.00167124876919668)	-4.74436178868438 (3.38751587778694e-11)	-2.8657064509032 (0.00806667855357836)
MUC6	0	0	0	0	0	5.89876395055047 (3.38713646319502e-07)	0	0	0	0
MUCL1	0	0	0	0	0	0	0	0	0	8.16862359897503 (6.22678265901966e-07)
MUL1	0	0	1.95161419767534 (0.000403415791322739)	0	0	0	0	0	0	0

MUM1L1	0	0	4.55928646446037 (0.00100517603709174)	0	0	0	0	0	5.47443146874558 (9.18466303961066e-05)	0
MURC	0	0	5.56940541928271 (9.6534594800889e-08)	0	0	0	0	0	0	0
MVP	0	0	0	0	0	0	0	-1.79239681163516 (0.00908735071917887)	-1.89009244190087 (5.71541772821502e-08)	0
MXD1	-2.97599542063095 (5.67559237817684e-08)	0	-3.46825403928038 (1.23803448224715e-09)	-2.15830380199342 (0.00955501944058622)	-2.45347423059756 (0.000557131463798855)	0	0	0	0	-2.15554441243925 (0.00934001986946932)
MXD3	0	0	0	0	0	0	0	3.24545044415965 (0.00271293646036162)	0	0
MXRA5	-2.67343111405093 (2.38924908669047e-05)	0	0	-3.22266889193124 (0.00130160855497207)	-2.5605397726768 (0.00971521054949249)	0	0	-4.671161513142 (0.000231643027928234)	-1.62261416529725 (0.000986130263420082)	0
MXRA7	0	0	0	0	0	0	0	-2.68028298265665 (0.00109157573759812)	0	0
MXRA8	0	0	0	0	0	0	0	-3.01540190857677 (0.0094441207700952)	0	0
MYB	0	0	0	0	0	0	0	5.68922334073761 (1.35019661257684e-09)	-3.55570093729746 (0.00132543263924388)	0
MYBBP1A	0	0	0	0	0	0	0	0	-2.40303639259883 (8.82044377131231e-09)	0
MYBL2	0	0	0	0	0	0	0	5.7698043041295 (2.0368805495335e-07)	0	0
MYBPC3	0	0	9.90576612636427 (3.3774506411951e-55)	0	0	0	0	0	0	0
MYCL	0	0	0	0	0	3.93276626643233 (0.00723960161577627)	0	0	2.81909812462738 (0.000452891826341374)	0
MYCN	0	0	0	0	0	5.71984409328395 (0.00138429050983591)	0	0	5.29561064475083 (6.86195430861588e-08)	0
MYCNOS	0	0	0	0	0	0	0	0	5.05554332230258 (4.81318818967096e-05)	0
MYCNUT	0	0	0	0	0	0	0	0	4.42229732437619 (0.000666167218271286)	0
MYCT1	0	2.64988676515259 (0.007306400276884)	0	0	0	0	0	0	0	0
MYEF2	0	0	0	2.28901728025011 (0.00184756995607476)	0	0	0	0	-2.50673906374884 (3.40719338596282e-08)	0
MYEOV	0	0	0	0	6.24434293510046 (0.0075784156376657)	7.14261019565052 (0.00718963385642891)	5.0719463490924 (0.00739670083218404)	0	0	0
MYEOV2	1.88129133971386 (0.00195068937144568)	0	2.36392052611507 (0.00119220378131527)	0	0	0	0	0	0	0
MYH1	0	0	0	0	0	6.03255908330019 (0.00959380943999706)	0	0	0	0
MYH10	0	-6.5571044392114 (0.00265931601811626)	0	0	0	0	0	0	0	0
MYH11	3.91673129782148 (5.98702624809129e-17)	0	0	4.9152140917028 (1.76181724991598e-09)	0	-2.32098831629181 (0.000112063609738849)	0	-5.02700492308273 (1.31718900055852e-07)	0	0
MYH14	-2.61305454187855 (9.1612763049045e-10)	0	1.99330120719584 (2.55540399031673e-06)	0	0	0	0	-7.86454740648384 (0.000275167857198903)	0	0
MYH15	0	0	0	0	0	4.65781559575941 (0.00898406752745877)	0	0	0	0
MYH3	0	0	0	0	0	2.98909911604923 (0.00293094817572201)	0	0	0	0
MYH6	0	0	14.3141941499709 (3.91095611255151e-38)	0	0	7.20258977590752 (0.00113156393925549)	0	10.2666564644186 (1.48160243108429e-15)	0	0
MYH7	0	0	16.0287923238036 (4.44820233297968e-69)	0	0	0	0	8.65232180742689 (6.4172143742874e-13)	0	0
MYH7B	0	0	6.30902734268135 (4.6978254042525e-29)	0	0	0	0	0	0	0
MYL2	0	0	14.3667703028676 (4.13747071049566e-41)	0	0	0	0	0	0	0
MYL3	0	0	8.72884317320976 (1.15906968986392e-74)	0	0	0	0	0	0	0
MYL4	0	0	7.73479627435058 (7.50570745476189e-09)	0	0	0	0	6.80720155574357 (0.00424504638285965)	0	0
MYL6	0	-3.15893156735423 (0.00607021202505752)	0	0	0	0	0	0	0	0
MYL7	0	0	9.66049324798116 (4.57046070267649e-11)	0	0	0	0	0	0	0
MYL9	3.00117313200196 (1.8075712218724e-13)	0	0	3.7365228691648 (2.57723467310352e-07)	0	0	0	-3.47028112968527 (0.000110839865169493)	0	0
MYLIP	0	0	0	0	0	-2.73845805599551 (0.00169869577577681)	0	0	1.62581958609054 (1.38187821067349e-05)	0
MYLK	2.38903082125493 (2.03145905084181e-09)	0	-2.88024027467985 (1.74290150400449e-09)	2.66009763178368 (9.22445179907463e-05)	0	0	0	-4.15087455930415 (1.69861945045989e-07)	0	0
MYLK3	0	0	10.8668330474922 (1.90349650458863e-25)	0	5.89112064521831 (0.000364466677787683)	0	5.12119322167001 (0.000555066008570529)	0	4.20752383595957 (0.00124055688713765)	0
MYO10	-2.46955390126325 (4.00132296114843e-09)	0	-1.79064112642236 (8.15249558867935e-05)	-1.80741347445464 (0.00739224505176174)	0	0	0	-2.65106115809487 (0.000505927216980601)	0	0
MYO15A	0	0	0	0	0	4.50704174871324 (0.00109750512028012)	0	0	0	0
MYO15B	-1.63943777242889 (0.0091184474714206)	0	0	0	0	0	0	0	0	-2.64308184912118 (0.00636480816297966)
MYO18A	0	0	2.02110344530158 (5.77142614195502e-08)	0	0	0	0	0	0	0
MYO18B	0	0	11.0855615817878 (2.94608997475468e-29)	0	0	0	4.34774495669542 (0.00646398437040879)	6.10874135550325 (0.000452738567725003)	0	0
MYO1B	-2.06538985763061 (1.18032101171792e-10)	0	0	-2.05424801313322 (0.000556641475267014)	0	0	0	-4.73318489780809 (2.72402200071683e-06)	0	0
MYO1C	0	0	0	0	0	0	0	-2.67755302351968 (0.000648825987669706)	0	0
MYO1F	-2.38641768248577 (1.12457943313316e-05)	0	-1.61841595948078 (0.00354791776872121)	0	0	0	0	0	0	0
MYO1G	-3.99328307762955 (1.92645041553101e-08)	0	-3.61112996015439 (1.41651397234837e-06)	-3.06458142698898 (0.0040626407767062)	0	0	0	0	-3.03097938781946 (2.34997382626941e-05)	-2.60711494245946 (0.00898505067628844)
MYO5B	-2.00077002445027 (1.58955908374458e-05)	0	-3.57893379465028 (2.30161541543967e-07)	0	0	0	0	0	0	0
MYO5C	-2.02191502023137 (0.0011220156416434)	0	-2.21659925488521 (0.000216614366152778)	0	0	0	0	-6.93490571954645 (0.0019539097267719)	-1.8269244070131 (0.000138816654845382)	0
MYO6	0	0	0	0	0	0	0	-6.13073249224868 (8.55602958772996e-05)	0	0
MYO7A	0	0	0	0	0	2.32901741314392 (0.000911472123846267)	0	0	0	0
MYO7B	0	0	2.81936752980253 (0.000577887081361155)	0	0	0	0	0	2.45820781023486 (0.000192485886658467)	0
MYOC	2.80315140465021 (0.00605267023779923)	0	0	0	0	0	0	0	0	0
MYOCD	3.42904962807927 (7.65528811690036e-14)	0	2.06164712712004 (0.000449329572376958)	3.30579252514826 (0.000128426169164513)	0	0	0	0	0	0
MYOF	0	0	-1.76331011002667 (4.83864550986458e-05)	0	0	0	0	-4.29267229287524 (1.07161628966713e-06)	0	0
MYOM1	3.15214219249089 (9.81015665007275e-08)	0	7.49719937854091 (8.56175784759336e-35)	3.72597317714354 (0.000161674115507615)	0	4.77291934331761 (7.10702546579089e-09)	0	0	0	0
MYOM2	0	0	8.46906718163787 (7.70711965789135e-38)	0	0	0	0	0	0	0
MYOM3	0	0	11.2080939373417 (8.18221890785271e-28)	0	0	7.32664413454337 (0.000235274337955248)	0	0	0	0
MYOT	4.07133690357856 (0.000646576036531628)	0	4.25031979487292 (0.00653510976160197)	0	0	0	0	0	0	0
MYOZ2	0	0	7.44610616170548 (2.34613952331447e-30)	0	0	0	0	0	0	0
MYOZ3	0	0	0	0	0	4.54315236949622 (0.00390670234064269)	0	0	0	0
MYPN	0	0	10.6743103690992 (3.3941818488374e-24)	0	0	0	0	0	0	0
MYRF	-2.96107228005284 (0.000453389218355379)	0	-2.39851861569376 (0.00777080440472924)	0	0	0	0	0	-1.78758356495682 (0.00442746396313331)	0
MYRIP	2.92052430718701 (0.000342619609531574)	0	0	0	0	4.79449611673911 (4.14584498428645e-06)	0	0	0	0
MYT1	0	0	0	0	0	7.12402202659993 (0.00179468134936318)	0	0	0	0
MYT1L	0	0	0	0	0	7.96539364268135 (2.2277519629013e-05)	0	0	0	0
MYZAP	0	0	4.39174843172328 (2.3287594545461e-08)	0	0	0	0	0	0	0
MZB1	0	0	0	0	0	0	3.08406222744885 (0.000222912076137317)	0	0	0
MZT2A	0	0	2.98512829974354 (1.35528249949885e-05)	0	0	2.88592624062733 (0.00363588811376738)	0	0	0	0
MZT2B	0	3.85595555379649 (0.00135393883876844)	2.05215480856805 (0.00134175930702852)	0	0	0	0	0	0	0
Metazoa_SRP	0	0	0	0	0	3.01513746248917 (7.80621041330902e-06)	0	0	0	0
N	0	0	0	0	0	0	25.6174018686478 (4.79297496991026e-31)	0	0	0
N4BP3	-1.75624929523012 (0.00954275834702363)	0	0	0	0	0	0	0	0	0
N6AMT2	0	6.57456842611761 (5.80312740690927e-07)	0	0	0	0	0	0	0	0
NAA11	0	0	0	0	0	0	0	0	6.34170958467506 (4.89390268085544e-08)	0
NAA38	1.68707903389224	0	1.80486805418532	0	0	0	0	0	0	0

	(0.00132420705253848)		(0.00500088734468994)							
NAALAD2	0	0	0	0	0	0	2.55139754657284 (0.00859164987306332)	0	2.53135962247531 (0.000680686315907945)	0
NAALADL1	0	0	0	0	0	3.81942581523486 (0.00867712789315859)	0	0	0	0
NABP1	-2.81929229328041 (4.018744318383e-11)	0	-3.56823805031484 (5.48822652529703e-15)	-3.47011053784677 (6.53764107971732e-08)	-1.50288122361801 (0.00798096284217201)	-1.69630230984753 (0.00214552856009677)	0	0	0	-1.93244832575369 (0.000841390104259708)
NACC2	1.50177953461088 (0.00034576834350604)	0	0	0	0	0	0	0	-1.59870164083135 (0.000107487765526487)	0
NAGPA	0	0	0	0	0	3.40929946492931 (0.000400970421275818)	0	0	0	0
NAGS	0	0	0	0	0	3.45529395684245 (0.000438601917105054)	0	0	0	0
NALCN	0	0	1.91207874818349 (0.00893773218499539)	0	0	4.15366113944614 (0.000537012472716989)	0	0	0	0
NAMPT	0	-2.71700486318427 (0.00228943770002311)	-2.55316321776219 (1.15996849906382e-09)	-2.11845090471252 (0.00255642842776606)	-1.95694542545268 (0.00227535111223149)	0	0	-2.14402691114589 (0.00299412262782888)	-2.46248892555723 (7.60584244111669e-10)	0
NAMPTP1	0	0	-3.6371047419089 (9.01132834067725e-06)	-4.9976623455057 (0.00408302253178614)	0	0	0	0	-2.76152916361948 (2.59793292269613e-06)	0
NANOS1	0	0	4.89576626816236 (0.00122896735446384)	0	0	0	0	0	0	0
NAP1L1	0	-3.92229595932925 (0.000754025873171864)	0	0	0	0	0	0	0	0
NAP1L2	2.91812807154033 (0.000599794287054284)	5.83815485506075 (7.54094818158549e-05)	0	0	3.73057294394263 (0.00528498549068203)	3.74901710814593 (0.00205715903111818)	0	0	0	0
NAP1L4	0	0	0	0	0	0	0	1.75541567490238 (0.00384956797386183)	0	0
NAPSA	-5.70411136921909 (5.02706861446344e-14)	0	-7.36011855697317 (1.49256002615116e-15)	-8.89084891546635 (5.84417553089017e-05)	0	0	0	-5.39077633125746 (0.00135363524637158)	-4.45292902843086 (1.63838485732922e-17)	-9.17212659371259 (3.39576911672178e-05)
NARF	0	0	0	0	0	0	0	3.03035925185379 (0.000236957601781095)	0	0
NAT16	0	0	0	0	6.65447770053204 (0.00254565647500638)	0	0	0	0	0
NAT2	0	0	0	0	0	6.41824022482157 (2.23851891938709e-05)	0	0	0	0
NAT8	0	0	0	0	0	5.16613052955743 (0.00993621958430608)	0	0	0	0
NAT8L	0	0	0	0	0	7.59741219160997 (0.00412697780405706)	0	0	0	0
NAV1	0	0	0	0	0	0	0	-2.5717055282247 (0.00389328339429399)	0	0
NAV2	0	0	0	0	0	0	0	-2.55944683492991 (0.00238201442794408)	0	0
NAV2-AS5	-2.55690140928656 (0.000631166996063563)	0	-3.2189300118187 (0.00059473108702095)	0	0	0	0	0	-1.96789240770639 (0.000930731591787591)	-6.38525479457234 (0.00536207273754849)
NBAS	0	0	0	0	0	0	0	1.82185433155719 (0.00282945195197269)	0	0
NBEA	2.44356869930183 (5.74749975798765e-09)	0	1.50691272515322 (0.000669464845204867)	2.15714177960166 (0.004770215272777)	0	0	0	0	0	0
NBEAL1	0	0	0	0	0	0	0	-2.6852595315852 (0.000603524268121282)	0	0
NBEAL2	-2.73989575145119 (2.98519437561788e-06)	0	0	0	0	0	0	0	-2.24936640685526 (6.33084097195146e-06)	0
NBL1	0	0	0	0	0	0	0	0	-2.96440695948901 (8.93942940568559e-05)	0
NBPF1	0	0	0	0	0	2.64694494950309 (0.00175444105349104)	0	0	0	0
NBPF14	-1.80164875040653 (0.00294105777136193)	0	0	0	0	0	0	0	0	0
NBPF19	0	0	-3.1842167287241 (7.31365447174239e-05)	0	0	0	0	0	0	0
NBPF3	0	0	0	0	0	0	0	0	1.91229494241383 (0.00607533915314031)	0
NCALD	2.18441067502035 (0.00042398979908302)	0	0	3.22339341301514 (0.00164791705953891)	0	0	0	0	0	0
NCAM1	4.43313855341296 (1.7182893411419e-13)	0	5.25125697240994 (2.77754606245248e-20)	4.9371723187886 (2.14394767134046e-07)	0	2.68864251442871 (0.00534606759534156)	0	0	0	0
NCAM2	0	0	-1.84104617309934 (0.00539313068858048)	0	-3.58384949882176 (0.00688819441243245)	-3.09395550148209 (0.00346807632236411)	0	0	-5.00340812357151 (1.53598661751129e-11)	0
NCAN	0	0	0	0	6.64816874282679 (0.00347040281013931)	7.43978491732081 (0.000391249603743542)	0	0	0	0
NCAPD2	0	0	0	0	0	0	0	3.09630934001119 (2.21205075527896e-05)	0	0
NCAPD3	0	0	0	0	0	0	0	3.04786140188908 (0.00371529512587119)	0	0
NCAPG	0	0	0	0	0	0	0	3.94154986997347 (0.000332501897625664)	0	0
NCAPG2	0	0	0	0	0	0	0	4.30117925151155 (5.28123131321405e-08)	0	0
NCAPH	0	0	0	0	0	0	0	4.12348454456796 (0.00191272444824962)	0	0
NCCRP1	0	0	0	0	0	0	0	0	0	8.61793333729518 (1.2642743336919e-08)
NCEH1	-2.64450029747032 (4.38091228303261e-06)	0	0	0	0	0	0	0	0	0
NCF1	0	0	0	0	0	0	0	3.65149621216307 (0.000160870739036402)	0	0
NCF2	-2.74079126689585 (2.3514729753721e-05)	0	-3.26387848246861 (9.69457448831032e-07)	0	-3.54817061433739 (0.000486791462374482)	0	0	0	-2.08232258564287 (4.09351823053192e-05)	0
NCF4	0	0	0	0	0	0	0	2.86482179729714 (0.00733418493806508)	0	0
NCKAP1	0	0	0	0	0	0	0	-3.32466133452006 (8.13485954863194e-06)	0	0
NCKAP5L	0	0	0	0	0	0	0	-6.41489652320857 (0.00437305118863192)	0	0
NCLN	0	0	0	0	0	0	0	0	-1.59593153225527 (0.000178231776437231)	0
NCLP1	2.96348150098902 (0.00967592596707494)	0	0	0	0	0	0	0	0	0
NCMAP	-4.94840397405221 (0.0030752423223068)	0	0	0	0	0	0	0	0	0
NCR3LG1	0	0	0	0	0	0	0	0	-2.19109476339385 (3.73998507125673e-05)	0
NCS1	3.05827913144756 (1.3462715325442e-07)	0	0	3.37276406387794 (0.000492589463116214)	0	0	0	0	0	0
NDE1	1.79139859707889 (1.80442205973033e-06)	0	0	0	0	0	0	0	0	0
NDE1P2	0	0	0	0	0	7.14261019565052 (0.00718963385642891)	0	0	0	0
NDFIP2	0	0	0	0	0	0	0	0	1.84265160222201 (4.20472613952917e-08)	0
NDNF	0	0	-1.93190588415358 (0.000854856954089244)	0	-2.08073320440966 (0.00372413278626388)	-3.52981929978212 (6.97269589714865e-06)	0	-5.61433341990607 (0.000451546147673661)	0	0
NDRG1	0	0	0	0	0	0	0	0	0	2.3604938365988 (8.90413842114571e-05)
NDRG2	0	0	1.80102590492301 (3.47266400131642e-07)	0	0	0	0	0	0	0
NDRG4	0	0	6.09668258967143 (8.85376416994914e-20)	0	0	0	0	0	0	0
NDUFA1	0	0	1.70312561753547 (0.000500053560132667)	0	0	0	0	0	0	0
NDUFA12	0	0	1.77178326516371 (4.21483834168481e-05)	0	0	0	0	0	0	0
NDUFA3	0	0	2.63168811441098 (4.38009725631259e-06)	0	0	2.09499328164988 (0.00240125617786186)	0	0	0	0
NDUFA4	0	0	1.82892544185916 (4.03387809799176e-05)	0	0	0	0	0	0	0
NDUFA4L2	0	0	2.50200169957832 (5.72091167734993e-06)	0	1.99248928438843 (0.0083214221091332)	0	0	0	1.58930534930256 (0.000157727848748014)	3.28088090474451 (2.35099241646609e-05)
NDUFA6	0	0	0	1.95741657776836 (0.00734623805446193)	0	0	0	0	0	0
NDUFA6-AS1	0	0	0	0	0	4.59900776232691 (0.00398814845821089)	0	0	0	0
NDUFA8	1.62489614672341 (0.00105360634175225)	0	2.76741000421466 (9.14767901180353e-09)	0	0	0	0	0	0	0
NDUFA9	1.53168463838383 (0.00855832351646382)	0	1.86613585354399 (0.00129344463322005)	0	0	2.8899986504114 (0.00523898273857522)	2.03860791255325 (0.00354484762756366)	0	0	0
NDUFAB1	0	0	2.09767654798635 (9.45849090629573e-05)	0	0	0	0	0	0	0
NDUFAF1	0	0	0	0	2.54673208976655 (0.0078265605768462)	0	0	0	0	0
NDUFAF2	0	3.14887998494063 (0.00132119557277978)	0	0	0	0	0	0	0	0
NDUFAF3	0	0	2.58376008951048 (0.000683620640146158)	0	0	0	0	0	0	0
NDUFAF4	0	0	1.6472005295407 (0.00423671478290968)	0	0	0	0	0	0	0
NDUFB10	0	0	2.01076269516048 (1.85529199445035e-06)	1.97836318971954 (0.00499796279160873)	0	0	0	0	0	0
NDUFB11	0	0	2.54062227376969 (1.06467926749831e-07)	0	0	0	0	0	0	0

NDUFB2	0	0	2.17284821122229 (1.51261172263667e-05)	0	0	0	0	0	0	0
NDUFB5	0	0	1.84121557080693 (2.1690446665322e-05)	0	0	0	0	0	0	0
NDUFB6	0	0	2.88594117871078 (1.21312663172282e-06)	0	0	0	0	0	0	0
NDUFB7	0	0	2.81148962707623 (4.87570075016196e-10)	0	0	0	0	0	0	0
NDUFB9	0	0	2.11373973227956 (4.83384346358691e-10)	0	0	0	0	0	0	0
NDUFC1	0	0	1.77882322458725 (0.000118289442116175)	0	0	0	0	0	0	0
NDUFS1	0	0	1.55534436398566 (1.6619186000409e-05)	0	0	0	0	0	0	0
NDUFS5	0	0	1.53798071835914 (0.000915384882634853)	0	0	0	0	0	0	0
NDUFS7	0	3.88249561166074 (0.001297245132609)	2.2956283561603 (9.131234594287e-05)	0	0	0	0	0	0	0
NDUFS8	0	0	2.38531608344924 (2.38779971140956e-07)	0	0	0	0	0	0	0
NEAT1	0	0	0	0	0	0	0	0	-1.55605839543232 (0.000122276630440391)	0
NEB	0	0	0	0	0	2.42047099763959 (0.0097769746682987)	0	0	0	0
NEBL	-4.23932371424278 (1.02771536450525e-15)	0	3.19906587807572 (9.65499173845709e-12)	-6.79321670591812 (1.95144615507665e-05)	-1.77469897668022 (0.00364115872628278)	-2.07107966791284 (0.000185744560482387)	0	-3.15142755425107 (0.000497893486658338)	0	0
NECAB1	2.51350207948522 (6.921800709399e-07)	0	2.40962251527232 (0.00119105567768703)	0	0	0	0	0	0	0
NEDD4L	-1.68382223541713 (6.98193004878867e-06)	0	0	0	0	0	0	0	0	-2.24440086025388 (0.00104221809599917)
NEDD9	-3.01062502858598 (5.7637650635513e-10)	0	-1.83950232807172 (6.45597309074481e-05)	0	-1.84180873496526 (0.00274231210071297)	-2.42451498040653 (1.82351686654225e-05)	0	-3.87523763576272 (1.62097311156118e-05)	0	-2.07454814641905 (0.00846701818096238)
NEFHP1	0	0	0	0	6.81201551833002 (0.00158736055535963)	7.6228092993656 (0.00281704145985764)	0	0	0	0
NEFL	6.35492077050992 (7.96495706390792e-07)	0	0	6.85993699047871 (0.000207884174780468)	0	0	0	0	0	0
NEFM	4.98595231134313 (1.26209901026059e-07)	0	0	6.23110049446067 (3.59140393518727e-05)	0	5.66466247242122 (0.00138658865102069)	0	0	0	0
NEGR1	2.37636716030385 (4.53719662369499e-05)	0	0	2.46445184374962 (0.00688104615050517)	0	0	0	0	0	0
NEIL3	-4.2597986781784 (0.00497797452165142)	0	0	0	0	0	0	0	-3.41611940269264 (0.00366282075760446)	0
NEK3	0	3.7022485254323 (0.000286879941139591)	0	0	0	0	0	0	0	0
NEK6	0	0	-1.54338410287378 (2.93878162403791e-05)	0	0	0	0	0	0	0
NEK8	0	0	0	0	0	4.07545464068569 (0.00571667082203808)	0	0	0	0
NES	-1.6167111484246 (0.000926259938328777)	0	1.51918384141505 (0.000470430257619509)	0	0	0	0	-3.9202574147688 (4.04750380791573e-05)	-2.405080359325 (1.76313928209662e-06)	0
NET1	0	0	0	0	0	0	0	0	1.57391931623669 (5.16690455828497e-06)	0
NETO2	-3.11746609271103 (0.00110152223005442)	0	0	0	0	0	0	0	-2.13331136639629 (0.00426008032779938)	0
NEU4	0	0	0	0	0	6.01111496006861 (1.80545840630101e-06)	0	0	0	0
NEURL1	0	0	0	0	0	0	0	0	3.48317787975922 (0.000139711560497939)	0
NEURL1B	0	0	2.07262376697733 (2.10335775974473e-05)	0	1.85764057812563 (0.00203679330065168)	0	0	0	0	0
NEUROD2	0	0	0	0	7.84249870914868 (1.91174859425042e-06)	6.02748257985459 (0.00267585545927866)	0	0	0	0
NEUROG3	0	0	0	0	0	6.0645547329706 (0.00760120533172173)	0	0	0	0
NEXN	0	0	2.42995708402005 (3.86249962083927e-07)	2.70121778963457 (0.00011317538986255)	0	-2.64548164177892 (4.28997104216453e-05)	0	0	0	0
NFAM1	-1.89150362321237 (0.00904478453451762)	0	-2.26860147537797 (0.00347706876373075)	0	0	0	0	0	-2.26407465588112 (0.00014810631678856)	0
NFASC	2.14107331447285 (1.67412295338424e-06)	0	0	2.16597898211151 (0.00242550451048033)	0	0	0	-7.1090948228575 (0.00113834563732008)	0	0
NFATC2	0	0	0	0	0	0	0	0	1.80703407179266 (4.4343996991436e-05)	0
NFE2	-3.54543888098327 (0.00587429318308767)	0	0	0	0	0	0	4.23268512381138 (0.000161360041461476)	0	0
NFE2L1	0	0	1.71772846746772 (1.87392550199428e-07)	0	0	0	0	0	0	0
NFE2L3	0	0	-1.78248687541309 (0.00258142518092386)	0	0	0	0	0	1.99695657882306 (0.000359525082366272)	0
NFIA	1.64588019393864 (9.24452059394881e-06)	0	0	1.70647022188728 (0.00846424828194002)	0	0	0	0	0	0
NFIB	0	0	0	0	0	0	0	-3.36644934813111 (5.72773726303734e-06)	0	0
NFIC	0	0	0	0	0	0	0	-2.07852303545655 (0.00101859849089579)	-2.20112877236184 (3.19619776449394e-17)	0
NFIL3	0	0	-1.84098886185147 (2.38471359354055e-05)	0	0	0	0	0	0	0
NFIX	0	0	0	0	0	0	0	0	-2.11533615687043 (2.1236231656782e-09)	0
NFKBIA	0	0	0	0	0	0	0	-2.02578356958084 (0.00365513709263605)	0	0
NFKBID	0	0	0	0	0	0	0	0	-2.90629803837623 (0.00026646542813602)	0
NFKBIZ	0	0	-1.85239650647903 (5.34954334602426e-05)	0	0	0	0	0	0	0
NFS1	0	0	0	0	0	2.95360040731819 (0.000648769655971688)	0	0	0	0
NFXL1	0	0	0	-3.57706353889866 (0.00877611170327577)	0	0	0	0	0	0
NGEF	0	0	0	0	0	6.5151368907745 (8.75062087056349e-06)	0	0	0	0
NGFR	2.46345826180313 (0.000416444188181257)	0	2.84882331797751 (5.26544287903436e-05)	0	0	3.05610458338899 (0.00133702612444445)	0	0	0	0
NHLH2	0	0	0	0	0	8.13922364025202 (0.000151723437723907)	0	0	0	0
NHS	2.38306221056305 (9.26725757243169e-05)	0	0	0	0	0	0	0	2.12499388749421 (0.000187937734813444)	0
NID1	0	0	0	0	0	0	0	-2.55528483355375 (0.000166504376852159)	0	0
NIPAL1	0	0	0	0	0	0	0	0	3.21299483704965 (0.000127664966572029)	0
NIPAL4	3.48518917605285 (0.00166964931150255)	0	0	0	0	0	0	0	0	5.66732954577947 (0.000290075213814213)
NIPBL	0	-5.98360484080116 (0.00466509740791215)	0	0	0	0	0	0	0	0
NIPSNAP1	0	0	0	0	0	2.23909129343304 (0.00147931597664366)	0	0	0	0
NIPSNAP3B	0	0	3.60796686313392 (6.32359841940719e-05)	0	0	0	0	0	0	0
NIT2	0	0	0	0	0	2.37600413758104 (0.000582814466312268)	0	0	0	0
NKAIN4	0	0	0	0	0	0	0	0	3.82201288994309 (0.00762144304755126)	0
NKD2	-3.73269261276741 (4.35143213762825e-07)	0	-4.00605765847887 (5.71525014927019e-09)	-7.89943424353275 (0.000314378974402149)	-2.83651182339231 (0.00444547874798052)	-2.69400897087081 (0.00143565924192063)	0	-6.84174969726784 (0.00187624908540039)	-3.31273265470838 (6.82340650307556e-06)	-3.1539350721032 (0.0014126947387439)
NKG7	-2.53195777673037 (0.00515744809894859)	0	0	-6.39750257845602 (0.00638140090651957)	0	0	0	3.33498701203534 (0.00100029746111766)	0	0
NKX2-1	-7.11357230340156 (6.30380084243693e-10)	0	-5.27868479018032 (5.17059556757815e-08)	-7.66081470337714 (0.000574973208917356)	-3.80929424019876 (0.00372162153559407)	0	0	-6.60449043531199 (0.00310533782552746)	-7.86522910305932 (5.76401390608792e-17)	-7.94203721384876 (0.000357294761779732)
NKX2-3	6.58372064899773 (6.54509394268202e-10)	0	0	0	0	0	0	0	0	0
NKX2-5	0	0	9.54952434763617 (1.22716951986822e-17)	0	0	0	0	0	0	0
NKX3-2	5.00784072202737 (0.000902921445998391)	0	0	0	0	0	0	0	0	0
NKX6-1	0	0	0	0	0	7.51697971622874 (0.000656978011332421)	0	0	0	0
NLGN1	2.69111865956764 (0.000350059061638397)	0	0	0	3.89912537134639 (0.000185170303658426)	3.62994650159887 (0.00351248244163173)	0	0	0	0
NLGN3	3.51691673269436 (0.00559819286718605)	0	0	0	0	6.2619932533775 (0.000416713363796989)	3.30494910287148 (0.00506982942472774)	0	0	0
NLGN4X	3.49246374587058 (2.93265035271015e-07)	0	0	0	0	0	0	0	2.03499974318156 (0.00528787387026265)	0
NLRC4	-2.99187591808288 (0.00200560097700588)	0	0	0	0	0	0	2.8701475243645 (0.0036610866203257)	0	0
NLRP10	0	0	0	0	0	0	0	0	4.75526301707086 (0.00300890594534692)	0
NLRP14	0	0	0	0	0	5.28441298714575 (0.00731399383739965)	0	0	0	0
NLRP2	0	0	0	0	0	0	0	0	4.03056953409979 (6.19983258834364e-14)	0
NLRP3	-4.04075709604825	0	-3.31130298265342	0	0	0	0	0	0	0

	(3.93031120675774e-05)		(0.000461337148153195)							
NLRP4	0	0	0	0	0	7.78075188122229 (0.000308390707327713)	0	0	0	0
NLRP8	0	0	0	0	6.798559966251 (0.0016583499845375)	0	0	0	0	0
NLRP9P	0	0	0	0	0	8.03380042840165 (0.00149615412257674)	0	0	0	0
NLRX1	0	0	2.39747492336765 (0.000611561971486603)	0	0	0	0	0	0	0
NME1-NME2	0	0	0	0	0	0	0	0	-1.55492997432618 (0.00175455766948737)	0
NME2P1	0	0	0	0	0	0	0	0	3.93277466255296 (0.00945975209010128)	0
NME9	-1.84291140936469 (0.00396737806041969)	0	0	0	0	0	0	0	-1.69441120392461 (0.0030926126643131)	0
NMNAT1	0	0	1.65517056145492 (0.00420261549324749)	0	0	0	0	0	0	0
NMNAT2	0	0	0	0	0	3.5055735897961 (0.00649406091347926)	0	0	1.82162997318627 (0.00522836140326322)	0
NMRK2	0	0	10.393470331669 (8.04133868749369e-24)	0	0	0	0	0	0	0
NNAT	3.07023998246976 (5.06340063721493e-06)	0	0	0	0	0	0	0	3.85136540079258 (3.04751753456645e-07)	0
NNMT	0	0	0	0	0	1.8505731874071 (0.00552585458297873)	0	-5.77063145161403 (0.000234133858992136)	0	0
NNT	0	0	1.93906256924262 (0.000116948810999365)	0	0	0	0	0	0	0
NNT-AS1	0	0	1.55132238372546 (0.00181222442778945)	0	0	0	0	0	0	0
NOD2	-2.81802443774862 (0.00312711894474566)	0	0	0	0	0	0	0	-2.11317859514353 (0.00131617013228523)	0
NOG	0	0	0	0	0	0	0	0	6.80204566681193 (4.93426896511271e-14)	0
NOL10	0	2.42722711636393 (0.00271289306788367)	0	0	0	0	0	0	0	0
NOL3	-1.54856183121361 (0.00905190655544894)	0	0	0	0	0	0	0	0	0
NOLC1	0	0	0	0	-1.86493318626851 (0.000920608009955744)	0	0	0	0	0
NOP2	0	0	0	0	0	0	0	0	-1.50409750337152 (0.000252384616612258)	0
NOS1	0	0	0	0	0	0	0	0	-4.34745324905773 (0.00026559490319668)	0
NOS1AP	0	0	0	0	3.14168107351339 (0.000713728395668472)	3.64456470255409 (7.86272298886337e-05)	0	0	0	0
NOS2	0	0	0	0	0	4.95937920539048 (0.000981706956233297)	0	0	0	0
NOS3	0	0	1.79309980826133 (0.000178743072655921)	0	0	0	0	0	0	0
NOTCH3	0	0	0	0	0	0	0	-2.65403806314206 (0.00045505723110728)	0	0
NOTCH4	-1.87125283777957 (0.00179107668981983)	0	0	0	0	0	0	0	-1.99878701416763 (0.00798491970944057)	0
NOTUM	0	0	0	0	6.17091833115035 (7.25334546316852e-05)	4.91613605756753 (0.00285940984333001)	0	0	7.98867935938874 (2.61650089407653e-23)	0
NOVA1	4.14647311338691 (3.6931821141414e-12)	0	2.48674303890992 (0.00104509476572192)	4.04512936739277 (0.000725997240767556)	0	0	0	0	0	0
NOVA2	0	0	0	0	0	0	0	-5.78657028281056 (0.00984235552058585)	0	0
NOX5	0	0	0	0	0	6.41748830813146 (7.29012460466738e-05)	0	0	3.77656518095104 (0.0099818515541562)	0
NPAP1P4	0	0	0	0	0	6.24545085153665 (0.00609245744822875)	0	0	0	0
NPAS2	0	0	1.88133655086707 (0.00042470530758748)	0	0	0	0	0	0	0
NPC1	-1.54533997766075 (1.31372355486108e-05)	0	-1.58762227751166 (3.12513404071792e-06)	0	-1.56360601488948 (0.00545922444439859)	0	0	-2.02466761238397 (0.00813533560210551)	0	0
NPC1L1	0	0	0	0	0	5.67941142408488 (1.21883517079129e-05)	0	0	0	0
NPC2	-1.72762952359641 (0.00265439507969336)	0	-2.05279872388699 (0.000361083509862149)	0	-3.7814702194878 (0.000396219806100631)	0	0	-3.32156489546865 (0.00315286101967034)	0	0
NPDC1	0	2.82135380278382 (0.00440874287129629)	0	0	0	0	0	0	0	0
NPFFR1	0	0	0	0	6.95283227746436 (0.00102465732257152)	6.65062606838638 (0.000273146199096326)	5.82112936753425 (0.00149047058789963)	0	0	0
NPHP4	0	0	0	0	0	2.61163640592864 (0.00739595864870866)	0	0	0	0
NPHS2	0	0	0	0	7.3517369215321 (2.58965363781595e-06)	0	0	0	0	0
NPIPB11	0	5.66585915259973 (1.47446900496931e-06)	0	0	0	0	0	0	0	0
NPL	-2.98664507784451 (2.64062722007031e-05)	0	-2.98957621538545 (0.000259659541271486)	-4.01182817590338 (0.00123471725420847)	0	0	-1.55372537065784 (0.00113882121588803)	0	-1.95340764688712 (0.00114266348382574)	0
NPM1P27	0	3.50794190398611 (0.0012593665424007)	0	0	0	0	0	0	0	0
NPM1P30	0	0	0	0	0	0	0	0	2.36334294912139 (0.00319097304427053)	0
NPM1P33	0	0	0	0	7.20697900913001 (0.00515462419084721)	0	0	0	0	0
NPM1P51	0	0	0	0	0	7.28272757431076 (0.0048656497259573)	0	0	0	0
NPM1P8	4.17605924069158 (0.000541265188100854)	0	0	0	0	0	0	0	0	0
NPM2	0	0	0	0	0	4.45373396837112 (0.00326417388587356)	0	0	0	0
NPNT	-2.15249788895842 (2.52004790085693e-07)	0	-4.42248113596993 (6.99245318723261e-19)	-2.5026614857213 (0.000337738998817441)	-1.98936390393796 (0.00235428263202786)	-2.56396421666324 (2.92210847841199e-05)	0	-8.74490759346108 (4.61453995882625e-05)	-4.00369280069164 (6.58756833288252e-24)	-2.39240823932809 (0.000492002009665059)
NPPB	0	0	25.1780743309451 (3.0531285336235e-20)	0	0	0	0	0	0	0
NPSR1-AS1	0	0	0	0	0	7.40960786669647 (0.00472974266362044)	0	0	0	0
NPTX1	5.1940771125464 (2.23876352000769e-10)	0	0	5.59570943204852 (2.61227533274931e-07)	0	0	0	0	0	0
NPTX2	0	0	2.8891805883649 (0.00369303901474751)	0	0	0	0	0	0	0
NPTXR	3.42130891783874 (4.34721689094504e-05)	0	5.09085187845706 (2.18756248046629e-10)	4.65302511164178 (0.000487392118490677)	3.92087430048436 (0.00356456813504119)	4.56429412721381 (1.31449920306262e-05)	2.49230914937141 (0.00626216640473816)	0	0	0
NPY2R	4.40777084435341 (0.00919842095943628)	0	0	0	0	0	0	0	0	0
NPY6R	0	0	5.54238074086049 (1.37534830931172e-13)	0	0	0	0	0	0	0
NQO1	-2.05473088368255 (0.00254746720982838)	0	0	0	0	0	0	-7.16908421128998 (0.00202879252150593)	-3.66749237776123 (9.05734762644451e-08)	0
NR1D1	0	0	0	0	0	0	0	-6.62986512227038 (0.00372986766958665)	0	2.60420481958889 (0.00510788231482194)
NR1H3	0	0	-1.80810057766953 (0.0049499865369555)	0	0	0	0	0	0	0
NR1H4	0	0	0	0	0	8.22709892151491 (4.07165860188826e-11)	0	0	0	0
NR1I2	0	0	0	0	5.57857079249512 (0.00693709453128892)	7.13880927950935 (7.41363682189752e-07)	0	0	0	0
NR1I3	0	0	0	0	0	6.45560681991192 (1.44957149045593e-06)	0	0	0	0
NR2E3	0	0	0	0	0	6.04930975984344 (0.00301040787466833)	0	0	0	0
NR2F1	-1.62370983072092 (0.000244952829744077)	0	-5.07781365861129 (1.57041692934222e-15)	-2.05751085711809 (0.00697889287424776)	0	0	0	0	0	-4.73119099131302 (2.4652780903506e-06)
NR2F1-AS1	0	0	-3.19437653354711 (0.000117467109229209)	0	0	0	0	0	0	0
NR2F2	0	0	0	0	0	0	0	-5.65586100758818 (1.76670134570719e-06)	0	0
NR2F2-AS1	0	3.80353583087294 (0.000783536776891701)	0	0	0	0	0	0	0	0
NR2F6	0	0	2.04282325171644 (0.00052129052799435)	0	0	3.38623023599355 (6.10987247727765e-05)	0	0	0	0
NR4A1	-2.77993572636258 (7.01361800541252e-05)	0	0	0	0	0	0	-4.23914601904629 (0.0007060422616418)	-1.69557438798764 (0.00434034995088833)	0
NR4A2	0	0	-1.86451409190443 (0.00270866452775489)	0	0	0	0	0	-1.95151147656415 (0.000102797859485272)	0
NR4A3	0	0	0	4.0234499314277 (1.78407996241463e-10)	0	0	0	-4.70119466806109 (0.00356285114496386)	-2.00822153911795 (2.09807285042361e-05)	0
NR5A2	0	0	0	0	0	4.22309514371082 (1.28090390632779e-09)	0	0	0	0
NR6A1	0	5.25065094229271 (4.19764105701594e-06)	0	0	0	0	0	0	1.82013367606089 (0.000316841576043982)	0
NRAP	0	0	13.0640758272294 (2.55200678627477e-36)	0	0	0	0	0	0	0
NRBP2	0	0	0	0	0	2.04938837747599 (0.0067278850868671)	0	0	0	0
NRCAM	-2.80753893399989 (1.25302370437024e-06)	0	-5.24618633446292 (3.87086677104938e-09)	-5.40125089308708 (0.00126478089954421)	0	-2.78926747241507 (0.00314048989557238)	0	-4.3450625884342 (0.00994996513378367)	0	-3.68372990862456 (0.00095893272564557)

NREP	0	0	0	0	0	0	0	0	1.55515559597048 (0.00140696281648956)	0
NRG1	0	0	0	0	0	2.24897822927133 (0.00206477019985999)	0	0	-3.42051750639828 (0.000146581302917352)	0
NRG2	2.99810052135261 (0.00970081038525634)	0	0	0	0	0	0	0	0	0
NRGN	-2.99050110809034 (4.20675496231118e-06)	0	0	0	0	0	0	0	-2.11590429210703 (0.00314056932815666)	0
NRIP1	0	0	0	0	0	0	0	0	2.18261541582466 (1.73562444647843e-08)	0
NRK	0	0	0	0	0	0	0	0	6.05081912519767 (4.20770342446309e-20)	0
NRN1	0	0	0	0	0	0	0	0	0	2.54798106097646 (0.00484866531117992)
NRP1	0	0	0	0	0	0	0	-2.57356809946534 (0.000145032805539748)	0	0
NRP2	2.21800180888862 (7.8268551824695e-13)	0	-1.66378598554423 (2.04486152873377e-05)	1.972830751731 (0.000580566999778915)	0	0	0	-3.90398508731392 (0.000159875600949102)	0	0
NRTN	0	0	4.38902705484441 (0.00261955709010454)	0	0	0	0	0	0	0
NRXN1	6.93830972183654 (3.16794960700082e-11)	0	4.07209152580005 (0.00688816111117593)	7.81606358463498 (1.54401221492951e-08)	6.14883838591478 (0.000881038748471602)	6.97050358014165 (0.00165022440597226)	5.41192023403415 (0.00661416084790861)	0	0	0
NRXN2	2.35420468319266 (0.00451771499566295)	0	0	0	0	3.75740522767565 (0.000180297970549126)	0	0	0	0
NRXN3	5.18423701227633 (3.28521730077694e-21)	0	0	5.28532281731089 (2.07606673881482e-09)	3.21054476914399 (0.00133197797220953)	4.0413207177343 (0.000483497921292013)	0	0	0	0
NSDHL	0	0	0	0	0	2.38104760268087 (0.0015674916320666)	0	0	0	0
NSUN6	0	0	0	0	0	2.38556970205236 (0.00494266053587202)	0	0	0	0
NT5DC3	0	0	0	2.46786385894943 (0.000830862423893272)	0	0	0	0	0	0
NT5E	2.4952636073798 (5.48396696502175e-07)	0	0	0	0	0	0	0	2.75080727180118 (2.28424654604742e-10)	0
NTF3	0	0	0	0	0	0	0	0	2.56322057379634 (0.00909272188502557)	0
NTM	0	0	0	0	0	0	0	0	-3.21541860538922 (0.000739638436638663)	0
NTN1	3.29831631243047 (3.29076923792055e-11)	0	2.97088452505877 (1.20747903941115e-05)	4.21905377837827 (3.90619565713998e-06)	0	2.64440716542916 (0.00460495287334058)	0	0	0	0
NTN4	0	0	0	0	0	0	0	-6.32320878251732 (0.0047062090827647)	0	0
NTNG2	0	0	0	0	0	0	0	4.60189981086607 (1.17563787539063e-05)	0	0
NTRK3	0	0	0	0	2.51633573789507 (0.0038792616441509)	0	0	0	0	0
NUCKS1	0	-4.23951702072481 (0.00715958354842829)	0	0	0	0	0	0	0	0
NUDT1	0	0	0	0	0	0	0	3.69445909657738 (0.0048940715504195)	0	0
NUDT10	5.04544339822744 (0.000269464083329973)	0	0	0	0	0	0	0	0	0
NUDT16	0	0	0	0	0	-1.68506602414853 (0.00858395358015143)	0	-2.97221740836274 (0.00247569528482923)	-2.58555885226394 (1.92450072382786e-09)	0
NUDT16L1	0	0	0	0	0	4.60320617721533 (2.84867790083418e-05)	0	0	0	0
NUDT19P4	0	0	0	0	0	7.44279116366548 (0.00404563973443074)	0	0	0	0
NUDT4	0	0	2.37238092267239 (1.11728039134871e-12)	0	0	0	0	1.89705698326004 (0.00629941464212069)	0	0
NUDT8	0	0	0	0	0	6.44127965995717 (8.89145398917259e-06)	0	0	0	0
NUF2	0	0	0	0	0	0	0	5.40259714329334 (0.00184308589911845)	0	0
NUGGC	0	0	0	0	0	3.82522946943836 (0.000371939068541272)	0	3.94044497458862 (0.00516051907179133)	0	0
NUP205	0	0	0	0	0	-1.59828125849655 (0.00389205937040696)	0	0	0	0
NUP210	0	0	-2.6177711535152 (0.000104161651223493)	0	0	0	0	2.65603860938892 (0.000738683957633317)	0	0
NUP93	0	0	0	0	0	0	0	2.579982075027 (0.00465480903324037)	0	0
NUPL1	-1.78497072991613 (1.46092255022978e-05)	0	-1.91396786014948 (5.47550320098499e-06)	0	-1.75447453368796 (0.00455715622900916)	-1.54361109238784 (0.00688874082042604)	0	0	0	0
NUPR1	0	0	0	0	0	0	0	-3.56152525331595 (0.000521974569519263)	0	0
NUSAP1	-2.16211655776252 (0.00584369915796709)	0	0	0	0	0	0	3.64068652287188 (0.00026011416211079)	0	0
NUTM2B-AS1	0	0	0	0	-2.34064553405862 (0.00659003170583658)	0	0	0	0	0
NXPE1	0	0	0	0	0	8.15033392202708 (0.000132853038779231)	0	0	0	0
NXPE3	0	2.99793436186817 (0.000205938584253328)	0	0	0	0	0	0	0	0
NXPE4	0	0	0	0	0	7.40176516885225 (0.000670275494300832)	0	0	0	0
NXPH3	2.04660901075901 (0.00618858518257344)	0	0	0	0	0	0	0	0	0
NYAP1	0	0	0	0	5.73439163325034 (0.00547108639398629)	0	0	0	0	0
OAF	1.78825079897298 (0.000170215379513568)	0	1.80536433128559 (0.000268449014836754)	0	2.50740894154658 (0.00084959243324551)	3.86251525994202 (7.73360530010115e-08)	0	0	0	0
OAS1	0	0	0	0	0	0	0	-6.16731465430257 (0.00910426357366361)	0	0
OAS2	0	0	0	0	0	0	0	0	1.98273288323912 (0.00212197263612089)	0
OAS3	0	0	0	-3.61604183935412 (0.000232566906638077)	0	0	0	-3.97224669132463 (0.000881977162349963)	0	-2.50557608179423 (0.00524892073850384)
OAT	1.6808115937926 (2.60715353860606e-05)	0	0	0	0	0	0	0	0	0
OBSCN	0	0	4.53345787283956 (4.65435500195578e-13)	0	0	0	0	0	-1.88285810000885 (0.00144707719298977)	0
OBSL1	0	0	2.3707435942306 (6.49786682310734e-05)	0	1.92482492377798 (0.000949727567713684)	0	0	0	0	0
OCLN	-2.47040183828813 (1.71472586584686e-05)	0	-3.76145778567324 (1.1351170077407e-05)	-6.85416226434102 (0.00189371873745189)	0	0	0	-5.79435575790209 (0.00892606635666689)	0	0
ODF3B	-2.47942919227082 (0.00121446841784117)	0	0	0	0	0	0	0	0	0
ODF3L1	0	0	0	0	0	6.88468650228311 (0.00201165457338688)	0	0	0	0
OGDH	0	0	2.82584763679196 (6.84611461136656e-10)	0	0	0	0	0	0	0
OGDHL	0	0	0	0	7.48041497209413 (5.88049001722268e-07)	8.4336632616745 (3.57320276291509e-10)	0	0	0	0
OGFRL1	0	0	-1.69827624974412 (0.000400187952806504)	0	0	0	0	0	0	0
OGG1	0	0	-1.51283620908877 (0.00846305319058967)	0	0	0	0	0	0	0
OGN	2.32211437141902 (0.00300984452808606)	0	0	0	0	0	0	0	2.22258283941488 (0.00289953313613422)	0
OIT3	0	0	0	0	0	7.14893159481956 (7.73925363181943e-06)	0	0	0	0
OLFM1	0	0	0	0	0	0	0	0	-3.18549294560922 (0.000229611874920369)	0
OLFM3	4.92293578268399 (0.00122390204528977)	0	0	0	0	0	0	0	0	0
OLFM4	3.55559448961608 (3.15615358279335e-08)	0	0	0	0	0	0	7.42335531275769 (8.44682983967463e-27)	-4.19916865382648 (6.76568935437143e-05)	0
OLFML2B	0	0	-3.19639898022356 (4.3132936462055e-06)	0	0	0	0	-4.65700735844601 (0.0059917111394232)	0	0
OLFML3	0	0	0	0	0	0	0	-6.50515391626859 (0.00385384530884257)	0	0
OLIG2	0	0	0	0	0	6.49029837126544 (0.000329900445214933)	0	0	0	0
OLR1	-7.43793603792311 (1.96743570287851e-10)	0	-5.09282509767454 (7.51002690657857e-08)	-3.96384442884275 (0.00135685779426345)	-5.11537667426987 (0.000359965121778402)	-2.82408461724904 (0.00272344311390321)	0	0	0	-3.24327344863973 (0.00283977163649866)
OMG	0	0	-3.0237591754883 (0.00099653163482097)	0	0	0	0	0	0	0
ONECUT1	0	0	0	0	0	7.58985217433136 (7.44803619388283e-09)	0	0	0	0
ONECUT2	0	0	0	0	0	7.25642101524978 (1.58451931165044e-10)	0	0	0	0
ONECUT3	0	0	0	0	0	8.10487236152792 (5.83262033935828e-06)	6.1235979021158 (0.00123173367023663)	0	0	0
OPA3	0	0	1.62904730535427 (0.000693823213373331)	0	0	2.30228652181298 (0.000714845128934605)	0	0	0	0
OPCML	0	0	0	0	0	6.79731418866435 (0.000344092872974137)	0	0	0	0
OPHN1	0	0	0	0	0	0	0	0	1.58357298819714 (0.000390110148092106)	0
OPLAH	-2.12135316094827	0	0	0	0	0	0	0	-3.60456935617177	0

	(0.00770852641361322)								(1.6853137900252e-05)	
OPN3	0	0	0	0	0	0	0	0	1.73030142201109 (0.00389790824084894)	0
OPRD1	0	0	0	0	0	5.96018596867437 (0.000252329349804793)	0	0	0	0
OPRK1	0	0	0	0	5.83005063945246 (0.00374446131635038)	0	0	0	4.51197908582178 (0.00586840108122496)	0
OPRL1	0	0	0	0	0	4.7936459742989 (0.00672782142965949)	0	0	0	0
OPRM1	0	0	0	0	0	7.56543785181961 (4.643332925092e-05)	0	0	0	0
OR13F1	0	0	0	0	0	6.17403800600322 (0.00788201947031849)	0	0	0	0
OR2C3	0	0	0	0	0	6.08313302421906 (0.00861121568334155)	0	0	0	0
OR2J3	0	0	0	0	0	8.03380042840165 (0.00149615412257674)	0	0	0	0
OR2V1	0	0	0	0	0	7.08201150050981 (0.00710054012903161)	0	0	0	0
OR4C4P	0	0	0	0	7.20697900913001 (0.00515462419084721)	0	0	0	0	0
OR4C50P	0	0	0	0	0	6.10213009384402 (0.00755053791667713)	0	0	0	0
OR4M1	0	0	0	0	0	7.92249751581524 (0.00165203987131941)	0	0	0	0
OR4X2	0	0	0	0	0	6.37117209463019 (0.00442564728836765)	0	0	0	0
OR51E1	0	0	0	0	0	7.08201150050981 (0.00710054012903161)	0	0	5.57303403756521 (0.000102140290946866)	0
OR51E2	0	0	0	0	0	0	0	0	4.70674650858907 (0.0010345835355819)	0
OR51T1	0	0	0	0	0	7.14261019565052 (0.00718963385642891)	0	0	0	0
OR55B1P	0	0	0	0	6.95283227746436 (0.00102465732257152)	0	0	0	0	0
OR56A1	0	0	0	0	0	7.35779101142259 (0.00500184185889881)	0	0	0	0
OR5AN2P	0	0	0	0	0	7.59741219160997 (0.00412697780405706)	0	0	0	0
OR5P1P	0	0	0	0	0	0	0	0	4.17443126843248 (0.00744743259295095)	0
OR7D2	0	0	0	0	0	5.46653271294761 (0.0091926260484846)	0	0	0	0
OR7E109P	0	0	0	0	0	7.30820580829314 (0.000786837051542909)	0	0	0	0
OR7E2P	0	0	0	0	0	5.07298489966399 (0.00620310838618998)	0	0	0	0
OR7E47P	-3.39762897070006 (0.0019947018164345)	0	0	0	0	0	0	0	-4.58044173270173 (3.16727041631258e-05)	0
OR8B12	0	0	0	0	0	6.61748565105267 (0.00329571580944009)	0	0	0	0
ORAI2	0	0	0	0	0	0	0	2.54081738066669 (0.00117848553931486)	0	0
ORAOV1	0	0	0	0	0	2.11749663587721 (0.00908135291867167)	0	0	0	0
ORC1	0	0	0	0	0	0	0	5.99180935283915 (8.36288653062565e-06)	0	0
ORF10	0	0	0	0	0	0	8.16851149937837 (0.000678873143361068)	0	0	0
ORF1a	0	0	0	0	0	0	27.4953539590366 (1.26105174889182e-40)	0	0	0
ORF1b	0	0	0	0	0	0	27.7130638330823 (1.30754232007648e-33)	0	0	0
ORF3a	0	0	0	0	0	0	24.1792652332076 (4.00849516114864e-25)	0	0	0
ORF6	0	0	0	0	0	0	23.9407879763357 (9.33940348833733e-26)	0	0	0
ORF7a	0	0	0	0	0	0	24.3866645179073 (3.23593625596623e-24)	0	0	0
ORF7b	0	0	0	0	0	0	8.62512799041888 (0.00109259612982993)	0	0	0
ORF8	0	0	0	0	0	0	21.9713116155993 (7.49195739830568e-22)	0	0	0
ORM1	0	0	0	0	0	8.77292409284767 (4.03301640022372e-25)	0	0	-3.92648998012352 (0.0017790047237425)	0
ORM2	0	0	0	0	0	11.2574185339357 (7.81101146000302e-19)	0	0	0	0
OSBP2	0	0	0	0	0	0	0	5.04331282499881 (1.04022262030393e-07)	0	0
OSBPL10	0	0	0	0	0	0	0	0	2.83261071671973 (4.01453411209262e-15)	0
OSBPL3	-1.54866006168749 (0.00224093405587966)	0	0	0	0	0	0	0	0	0
OSBPL6	0	0	0	0	0	0	0	0	-3.15053758345448 (9.74688127753044e-05)	0
OSBPL7	0	0	0	0	0	0	0	0	-2.30789500022241 (0.000451090962974321)	-6.43207402654742 (0.00624784287343636)
OSBPL8	0	0	-1.89494567456222 (6.34273966198063e-07)	0	-2.21592942238249 (0.000438961779849607)	-2.19058908453049 (9.3139277158101e-06)	0	0	0	0
OSCAR	-2.8976280519741 (0.00219378038397511)	0	0	0	0	0	0	0	-2.61140877970803 (0.00305098475263764)	0
OSGIN1	0	0	0	0	0	0	0	0	-4.72584800721682 (0.00038270933662726)	0
OSGIN2	0	0	-2.77139112863207 (5.62710165172366e-06)	0	0	0	0	0	0	0
OSM	-4.15415187868982 (0.00471400267505065)	0	0	0	0	0	0	0	0	0
OSMR	0	0	0	0	0	-1.71807884220158 (0.000217901151201594)	0	-3.41841761204423 (1.33912390161045e-05)	0	0
OSR1	0	0	-2.0503234767444 (0.00654050903111383)	0	0	0	0	0	-2.36036339130842 (0.000768813793266692)	0
OSR2	0	0	0	0	0	0	0	0	3.53843001620671 (0.00011291632696091)	0
OTC	0	0	0	0	0	7.43291814621598 (0.000153741014535531)	0	0	0	0
OTOA	0	0	0	0	0	5.73619295514306 (0.0012361296822153)	0	0	0	0
OTOG	0	0	0	0	0	7.32624266357827 (0.00538433295435045)	0	0	0	0
OTP	0	0	0	0	0	7.21261123709105 (0.00552449200967179)	0	0	0	0
OTUB2	0	0	0	0	0	5.31707105048395 (5.92179270950912e-05)	0	0	0	0
OTUD1	0	0	0	0	0	-2.70522394500581 (0.00825503172560431)	0	0	0	0
OTUD7A	0	0	0	0	3.21078508682339 (0.00174504254291673)	0	0	0	0	0
OTUD7B	0	0	0	1.69796658666754 (0.00827925140152826)	0	0	0	0	0	0
OVCH2	0	0	0	0	0	0	0	0	3.98198162467327 (0.00179813220860864)	0
OVOL1	0	0	0	0	0	0	0	0	5.10919591553028 (0.000126538134646787)	0
OXCT1	0	0	1.87849906951899 (0.00123104202018832)	0	0	0	0	0	0	0
P2RX4	0	0	-1.85682337876665 (0.00163048509788212)	0	0	0	0	0	0	0
P2RX5-TAX1BP3	0	0	0	0	5.81739974794478 (0.00592266915934036)	0	0	0	0	0
P2RX6	0	0	0	0	5.98514520078338 (9.64667155520581e-05)	0	0	0	0	0
P2RY1	0	0	-2.68508750797241 (7.613396515865e-05)	0	0	0	0	0	0	0
P2RY14	3.40782155365587 (7.23440273059267e-07)	0	0	3.49864002471684 (0.00558492162880537)	0	0	0	0	3.48028946547249 (1.39987180382721e-09)	0
P2RY2	0	0	2.30776064704641 (0.000840295348060047)	0	0	0	0	0	0	0
P2RY6	0	0	0	0	0	0	3.79679226826975 (0.00109818022768608)	0	4.48467716821771 (3.7104641963736e-05)	0
P2RY8	0	0	0	0	0	0	0	3.48960245627914 (0.00734062194164479)	0	0
P3H2	0	0	-2.62779532177842 (0.0068547083244927)	2.64752225460279 (0.00793704039946461)	0	0	0	0	0	0
PABPC1	0	0	-1.75252959510834 (1.42618050539959e-05)	0	0	0	0	0	0	0
PABPC1L	0	0	-2.11895977097512 (0.00856932159420312)	0	0	0	0	0	0	0
PABPC1P4	3.63802863020242 (0.00426884385486103)	0	0	0	0	0	0	0	0	0
PABPC4L	0	0	0	0	0	0	0	0	3.27842006010424 (1.53270012505011e-05)	0

PACRG-AS1	0	0	0	0	0	6.45077925459454 (0.00401811255958677)	0	0	0	0
PACSIN1	0	0	6.05497029660408 (5.89549149353453e-06)	0	0	5.53533543574327 (0.00488620942992971)	0	0	0	0
PACSIN3	0	0	3.7797181793284 (2.01410057491514e-14)	0	0	0	0	0	0	0
PADI1	0	0	0	0	6.798559966251 (0.0016583499845375)	0	0	0	4.95201434750527 (0.00218757917008494)	0
PADI2	0	0	-3.22656963972008 (0.00788205294725549)	0	0	0	0	0	0	0
PADI3	0	0	0	0	6.24434293510046 (0.0075784156376657)	0	0	0	0	0
PADI4	0	0	0	0	0	0	0	5.24310539751059 (3.11156243097989e-07)	0	0
PAEP	0	0	0	0	0	0	0	0	9.30045095656488 (0.00189697562492038)	0
PAG1	-2.44947473128617 (5.72678556638605e-07)	0	-1.74425789492249 (1.91771481741551e-05)	-2.49853050132843 (0.000362879088980955)	-2.50169099637723 (3.87878012270255e-05)	0	0	0	0	-2.23747269360992 (0.00112461394473486)
PAGE4	0	0	0	0	6.46404730620529 (0.00343645514417636)	0	0	0	9.77363091592266 (6.50687252046447e-09)	0
PAH	0	0	0	0	6.47559966567641 (0.0042482603748159)	10.9471265085316 (3.58756809561261e-19)	0	0	0	0
PAIP2B	2.15604656593105 (0.000322292527594245)	0	3.25139988171691 (5.82130467472517e-07)	2.82730302412588 (0.00958168549225101)	0	3.3120309183946 (4.47595555625998e-07)	0	0	0	0
PAK6	-4.24978039122111 (0.00306213114487745)	0	0	0	0	0	0	0	0	0
PALLD	2.16041499991798 (1.4268188858533e-10)	0	1.93513194361422 (4.01716800511374e-08)	2.48458366271767 (6.75418698796751e-05)	0	0	0	-6.56173060175573 (3.42276683484548e-05)	0	0
PALM	0	0	0	0	0	0	0	-6.09338101922112 (0.00557640151135336)	0	0
PALM3	0	0	0	0	0	2.868383384801 (0.00542764111558516)	0	0	0	0
PALMD	0	2.46459537464321 (0.00355207000305388)	2.70212452275096 (2.12871046685856e-12)	0	0	0	0	0	1.88488282218313 (1.44889474634256e-07)	2.42839267438096 (0.000414579517892239)
PAM	0	0	1.81301904538746 (0.000102214877244988)	0	0	-1.71319533288571 (0.000304448134374683)	0	-2.22954062931601 (0.00124779238364375)	0	0
PAMR1	0	0	0	0	0	0	0	0	3.33939483731099 (0.00166342754016584)	0
PANDAR	0	0	0	0	0	6.03826101173533 (0.00784359047003729)	0	0	0	0
PANK1	0	0	0	0	0	2.77463439623862 (0.00108573347409072)	0	0	0	0
PANX2	0	0	0	0	0	3.20653459686172 (0.0010531989579696)	0	0	0	0
PAPLN	-1.66002211955606 (0.0035388650637896)	0	0	0	0	0	0	0	0	0
PAPPA	0	0	-3.23465762797659 (3.6879317040899e-06)	0	0	0	0	-5.0258482865546 (0.00305420339739938)	5.17361512577014 (1.96369314487327e-14)	-2.77969366089631 (0.00409720252687709)
PAPPA-AS2	0	0	0	0	0	0	0	0	5.94152589823959 (0.000236482570029093)	0
PAPPA2	2.51858804674581 (0.00906418291772061)	0	0	0	4.52879560855748 (0.000207167262063098)	4.28965660673167 (0.00482816050286149)	0	0	10.61267720349 (2.8607368916046e-44)	0
PAPSS2	-2.66831980521281 (1.52547031831033e-10)	0	-2.0392698063971 (9.81226421588531e-07)	-2.72285727745711 (0.00012895421421692)	-1.80251493894941 (0.0028023404200253)	0	0	-5.572108906458 (0.000395235876712927)	0	-1.74043016472413 (0.00756330742249088)
PAQR5	-2.62625444243549 (0.00882053945523762)	0	0	0	0	0	0	0	0	0
PAQR7	0	0	0	0	0	0	0	0	2.38743289026707 (2.08891947640285e-05)	0
PAQR8	2.26260109168027 (4.67426486949321e-05)	0	0	0	0	0	0	0	0	0
PAQR9	0	0	4.88932009633989 (0.0045001409840533)	0	0	6.61784331281066 (0.000263537015764799)	0	0	0	0
PAQR9-AS1	0	0	5.14481566512033 (0.000239521427481164)	0	0	0	0	0	0	0
PARD3	0	0	0	1.79352465731391 (0.00290939139652739)	0	0	0	-2.53862905782278 (0.00356767302184682)	0	0
PARD6B	0	0	-2.34021566209627 (0.00238302829634503)	0	0	0	0	0	0	0
PARD6G	0	0	0	0	0	4.5636434537884 (0.000481469145891434)	0	0	0	0
PARD6G-AS1	0	0	0	0	3.54035372246427 (0.00426560465383985)	0	0	0	0	0
PARK2	2.77306291123267 (0.000601970267246702)	0	2.98848921680406 (0.000645868652300712)	0	0	0	0	0	0	0
PARM1	0	0	0	0	0	0	0	-4.47946514610765 (0.00096015032232205)	0	0
PARP14	0	0	0	-1.76489918501979 (0.00157630382668287)	0	0	0	0	0	-1.61636028404663 (0.00365451300811857)
PARP15	0	0	-2.89518312176093 (0.00591644990081509)	0	0	0	0	0	0	0
PARP8	0	0	-1.85584766705418 (0.00204717091579023)	0	0	0	0	0	0	0
PART1	5.50364791297225 (3.25004711422801e-09)	0	0	6.06112652773792 (9.16269876950486e-05)	4.75377557930939 (0.00897380323770182)	4.8342525527518 (0.00809694350344724)	0	0	4.19580200971956 (0.000129300526873056)	0
PARVA	1.91228644106299 (1.48831262182476e-10)	0	0	2.19364128546614 (0.000478250507584521)	0	0	0	-4.03120954693087 (0.00100139005514053)	0	0
PARVB	0	0	2.72722109177993 (2.30129408439458e-07)	0	0	0	0	0	0	0
PARVG	-1.64413631068341 (0.0048112144336613)	0	-1.69410336585917 (0.00756130485463971)	0	0	0	0	0	-1.50355221487399 (0.00938068820166785)	0
PASK	0	0	0	0	0	0	0	0	-1.88570538069351 (0.006586308070023)	0
PATE3	0	0	0	0	0	7.35779101142259 (0.00500184185889881)	0	0	0	0
PATE4	0	0	0	0	0	7.22943862102695 (0.00172832028801412)	0	0	0	0
PAX2	0	0	0	0	9.68705446708221 (4.37736641116845e-16)	7.78235125660529 (0.00028226105212682)	0	0	0	0
PAX4	0	0	0	0	0	6.35993786149111 (0.00619281386009252)	0	0	0	0
PAX5	0	0	0	0	0	5.620943300262 (0.000623379311441764)	4.57781383735212 (0.00469681541981858)	0	0	0
PAX6	3.18981854714742 (0.00355350378588268)	0	0	0	0	5.84799488429522 (0.000429747149271987)	0	0	0	0
PAX7	0	0	0	0	0	6.93940585537634 (9.33601111861849e-05)	0	0	0	0
PAX8	0	0	0	0	6.54637567928953 (2.21968879714681e-17)	0	0	0	0	0
PAX8-AS1	0	0	0	0	3.74836130743383 (8.28927240309873e-05)	0	1.90832159710755 (0.0067187441436173)	0	2.55122408087468 (8.36380323006306e-06)	0
PBLD	0	0	0	0	0	3.94759014207645 (6.12599881172991e-09)	0	0	0	0
PBRM1	0	0	0	0	-1.82572293854933 (0.00109809140502965)	0	0	0	0	0
PBX1	1.82876395490283 (2.91828435750738e-06)	0	0	0	0	0	0	-2.57800835497625 (0.00337822331363062)	0	0
PBXIP1	1.53476634240003 (2.62615187064303e-05)	0	0	2.57612587581346 (0.000171542502985493)	0	0	0	0	0	0
PC	0	0	0	0	0	5.13840928321024 (2.07373034796004e-09)	0	0	0	0
PCA3	3.79926105382725 (6.09924968133784e-11)	0	0	4.64322889835734 (6.00964987449975e-06)	0	0	0	0	0	0
PCAT19	0	0	0	0	0	0	0	-6.14804505779471 (0.0067675760896744)	0	0
PCBD1	0	0	0	2.54180510138169 (0.00797042245892253)	0	2.10456339148995 (0.00188335989744902)	0	0	0	0
PCBP3-OT1	0	0	0	0	0	3.11738491109901 (0.00922553526056282)	0	0	0	0
PCCA	0	0	0	0	0	1.88090236730158 (0.00122053504688481)	0	0	0	0
PCDH1	0	0	0	0	0	-1.71311730266488 (0.00661262343235877)	0	-3.91205634709061 (0.000634353548030607)	0	0
PCDH10	0	0	0	0	0	5.78467852972152 (0.00129603465047504)	4.30702854422693 (0.00349999216404386)	0	5.7601401348178 (7.64198121678507e-11)	0
PCDH11X	0	0	0	0	0	0	0	0	4.83189585485846 (0.00018612450255801)	0
PCDH11Y	0	0	0	0	0	4.95312405167556 (0.00453224589178075)	0	0	0	0
PCDH17	-1.68153310458776 (0.000188918690308281)	0	0	0	0	0	0	0	-2.02743449768671 (0.00204482787003164)	-3.2421534796881 (0.000223064548388648)
PCDH18	0	0	0	0	0	0	0	0	1.56503834288582 (0.00231955290888665)	0
PCDH19	3.96078358932819 (0.00266918252612865)	0	4.55187981690464 (0.000184785665810637)	0	0	6.83883912911116 (3.67704770182064e-05)	4.08838246086717 (0.00848055558161215)	0	0	0
PCDH20	0	0	0	0	0	3.29212382141409 (0.00734953853207998)	0	0	0	0
PCDH7	3.31997628461774 (4.89106455686972e-12)	0	2.87431282810332 (2.72781707472856e-05)	3.35317961373505 (4.88845310553082e-05)	0	0	0	0	0	0
PCDH8P1	0	0	0	0	6.24434293510046 (0.0075784156376657)	0	0	0	0	0
PCDH9	0	0	2.39052220312593	0	0	0	0	0	0	0

			(4.73323528838614e-05)							
PCDHA10	0	0	0	0	0	0	0	0	-3.35843754135547 (0.0035347052243112)	0
PCDHAC1	0	0	0	0	0	4.56860119054774 (0.00040042431967107)	0	0	0	0
PCDHB1	0	0	0	0	0	5.34194100794642 (0.00228493468891166)	0	0	0	0
PCDHB11	2.3200597124693 (0.00377516148203379)	0	0	0	0	2.90203899738364 (0.00987780439280622)	0	0	0	0
PCDHB13	2.49191352461527 (0.000529660836356053)	0	0	0	0	0	0	0	0	0
PCDHB16	0	0	0	0	0	0	2.19074023690426 (0.00468438124986742)	0	0	0
PCDHB7	0	0	0	0	0	3.61014352339934 (0.00481213742593748)	0	0	0	0
PCDHGA2	0	0	2.02043839586084 (0.00268079384999936)	0	0	0	0	0	0	0
PCDHGA3	0	0	3.51401173649891 (0.00354028999118792)	0	0	0	0	0	0	0
PCDHGA4	0	0	3.63710780670475 (0.000245514911883044)	0	0	0	0	0	0	0
PCDHGA5	0	0	2.81840933434829 (0.000284110367305466)	0	0	0	0	0	0	0
PCDHGA6	0	0	2.45573727801727 (0.000570642070905184)	0	0	0	0	0	0	0
PCDHGA7	0	0	3.91274928746482 (4.08937485958532e-07)	0	0	0	0	0	2.40398983571995 (0.00969238243104772)	0
PCDHGA8	0	0	3.31878372039788 (0.000190231530477651)	0	0	0	0	0	0	0
PCDHGA9	0	0	1.86043399807583 (0.00657296843251146)	0	0	0	0	0	0	0
PCDHGB2	0	0	2.33106925201902 (0.00612738222199261)	0	0	0	0	0	0	0
PCDHGB3	0	0	2.71560214556728 (0.000199964718294513)	0	0	0	0	0	0	0
PCDHGB4	0	0	2.665745338178 (0.00379559666498015)	0	0	0	0	0	0	0
PCDHGB6	0	0	1.9826186609937 (0.00462683704012682)	0	0	0	0	0	0	0
PCDHGC3	0	0	0	0	0	0	0	-3.75010420877284 (0.00302825940947721)	0	0
PCDHGC4	0	0	2.54249586662016 (0.00402312688016785)	0	0	0	0	0	0	0
PCK1	5.66814541248877 (1.41307298083123e-06)	0	4.14376307175874 (0.00366316168489106)	6.47478171101286 (0.00012969192725509)	8.17100945812916 (7.72596066208166e-15)	11.7608755921803 (2.32586034671395e-26)	4.97181130817267 (0.00706976609664327)	0	0	0
PCK2	0	0	0	0	0	2.33838674008094 (0.000179591036495919)	0	0	0	0
PCNP	0	0	0	0	0	-1.50126928714421 (0.00238714016659093)	0	0	0	0
PCOLCE	-2.17472608710863 (0.00139292031501764)	0	0	-2.98512319144776 (0.0080862324429893)	0	0	0	0	0	-2.78228609743962 (0.00952095339450312)
PCOLCE2	0	0	3.04676538501185 (3.60631269544015e-06)	0	0	0	0	0	0	0
PCP4	6.01269708008196 (1.77026023818612e-06)	0	0	0	0	0	0	0	0	0
PCP4L1	4.04795374394937 (1.42472803511977e-06)	0	0	5.13130640085766 (0.000358077762373997)	0	0	0	0	0	0
PCSK2	0	0	0	0	0	4.45866595886673 (0.00543135220516186)	0	0	0	0
PCSK6	0	0	2.6149468533913 (2.1453336213465e-05)	0	0	4.83458077289266 (3.20315230105344e-10)	0	0	2.75458174340294 (1.47928751898328e-08)	0
PCSK9	0	0	0	0	0	3.29524405197193 (0.00753021450164039)	0	0	0	0
PCYOX1	1.50429602668971 (0.000503588587717249)	0	0	0	0	0	0	-3.77069345814663 (0.00275075201421103)	0	0
PCYT1A	0	0	0	0	-1.79167343484324 (0.00879071951305633)	0	0	0	0	0
PDCD4	0	0	0	0	0	0	0	0	1.62430719201724 (1.69032306369746e-07)	1.93922724173612 (0.00504527788616519)
PDE10A	0	0	3.00710082711411 (8.28910345385457e-08)	0	2.52250996641192 (0.00791725525086634)	0	0	0	2.89573645569531 (1.13083007967596e-08)	0
PDE11A	0	0	3.41961693985914 (0.00983291814612549)	0	5.36754167567768 (9.77683084146664e-05)	6.43717967841657 (6.65821532304002e-06)	0	0	0	0
PDE1A	2.67988609073146 (7.74751180636204e-09)	0	1.82601741749247 (0.00906787134735134)	2.73213929971721 (0.00853511653823216)	2.61711651503803 (0.00183354269032008)	0	0	0	0	0
PDE1B	2.23937488617254 (0.00011725244723167)	0	0	0	3.03084301676824 (0.00033696554002348)	2.93118570075123 (0.00424631448970207)	0	0	0	0
PDE1C	1.83702275445584 (0.000934407639669448)	0	3.67879556097348 (1.36033890009813e-11)	0	0	0	0	0	0	0
PDE2A	1.65878136351238 (0.000177269096860676)	3.92336225101334 (3.4958979578679e-05)	2.26908312072384 (7.31169466889447e-07)	0	0	1.77773208410471 (0.00505307621700322)	0	0	0	2.69821441273022 (0.00117459617231944)
PDE3A	2.51860499536634 (2.51238938956648e-09)	0	2.89936373691525 (1.48399235053847e-12)	2.60987394113751 (0.000960770393118031)	0	0	0	0	0	0
PDE3B	-1.83982120604675 (0.00097530735576234)	0	-1.96617338147495 (0.000135503250580445)	0	0	0	0	0	0	0
PDE4B	0	0	-1.64688884954787 (0.00726151355414264)	0	0	0	0	0	0	0
PDE4C	0	0	0	0	0	2.90202778729707 (0.00509202218119925)	0	0	0	0
PDE4DIP	0	0	2.97062968231589 (3.99067762564654e-09)	0	0	0	0	-3.0592919112366 (0.00105967487475031)	0	0
PDE5A	0	0	-1.63382450865852 (0.00143437228322374)	0	0	0	0	-2.66381229155093 (0.00752132767626613)	0	0
PDE6A	0	0	0	0	6.92215118568209 (0.000101041528590178)	7.07424808684915 (0.000264572701368491)	0	0	0	0
PDE6G	0	0	0	0	6.55964833306812 (0.00285105583660765)	0	0	0	0	0
PDE8B	0	0	0	0	0	0	0	0	2.11395785316093 (7.55396546978874e-06)	0
PDE9A	3.16897115711393 (3.35621039344069e-09)	0	0	0	2.66113339874476 (0.00468829696060479)	0	0	0	0	0
PDGFA	0	0	0	0	0	0	0	-4.7822583832042 (0.0037427093057664)	0	0
PDGFB	-1.68131069523406 (0.00656667979682598)	0	0	0	0	0	0	0	0	0
PDGFC	1.66229391676558 (0.000602211894020041)	0	0	0	0	0	0	0	0	0
PDGFD	0	0	3.36144794687204 (1.64002195880189e-06)	0	0	0	0	0	2.28143356428767 (0.00167010927615548)	0
PDGFRA	0	0	-1.84483760429685 (7.03663404458691e-10)	0	0	0	0	-3.13347389888428 (9.0942866955551e-06)	0	0
PDGFRB	0	0	0	0	0	-1.54199426962066 (0.00943021495229391)	0	-4.18104689330626 (1.81960902490117e-08)	0	0
PDGFRL	0	0	0	0	0	0	2.62375289991169 (0.00222474980170772)	0	0	0
PDHA1	0	2.73098937003085 (0.000423905639167502)	1.9444822316229 (1.54529860794948e-07)	0	0	0	0	0	0	0
PDK2	2.2743883615946 (0.000340374395080058)	0	3.37254811586372 (8.99437875579756e-08)	0	0	0	0	0	0	0
PDK3	2.09230333182493 (3.18229537189519e-05)	0	0	0	0	0	0	0	0	0
PDK4	0	0	0	0	0	-2.5774281252371 (0.00332839384023656)	0	0	0	0
PDLIM1P4	0	0	0	0	0	0	0	0	3.45351872003994 (0.0057285283972335)	0
PDLIM2	-2.18156452102315 (0.00118751878451667)	0	0	0	0	0	0	0	0	0
PDLIM3	0	0	0	1.70965522688811 (0.00829149336670507)	0	0	0	-8.09495579836022 (0.00016904067656076)	0	0
PDLIM4	3.49901330687768 (2.87022307792232e-07)	0	0	4.32956530914578 (0.00051466391963727)	0	0	0	0	0	0
PDLIM5	0	0	2.04794539256243 (4.40415196460614e-10)	0	0	0	0	-2.26154379501323 (0.00335137307033245)	0	0
PDLIM7	0	0	0	0	0	-1.84876087222676 (0.00731588803260506)	0	0	0	0
PDPN	0	0	0	0	0	0	0	-6.68042132033582 (0.003323568990343)	0	0
PDZD2	0	0	2.29180565640745 (2.04898555486784e-09)	0	0	0	0	-4.41212752024249 (0.0078455833154876)	2.16682044484447 (3.5272579461116e-07)	0
PDZD3	0	0	0	0	6.80050039693727 (0.00176612853259011)	0	0	0	0	0
PDZD7	0	0	0	0	0	6.71387110199867 (1.26785719516669e-05)	0	0	0	0
PDZK1	0	0	0	0	0	5.17429540821372 (9.60742686167068e-05)	0	0	0	0
PDZK1IP1	-5.09170918161556 (0.000189765554505116)	0	0	0	0	0	0	0	0	0
PDZRN3	2.71210663902608 (2.09690940019269e-07)	0	1.7831217764416 (0.000845514216538631)	0	0	0	0	0	0	0
PDZRN4	7.76422009765343 (2.51585145621042e-19)	0	0	8.68381709146177 (9.35473804280724e-16)	0	0	0	0	4.70889825157152 (2.04643275473143e-08)	0

PEA15	0	0	0	0	0	0	0	-4.56665676289562 (7.54619325408331e-08)	0	0
PEAR1	0	0	0	-3.84133118663325 (0.00617782597344194)	0	0	0	0	0	0
PEBP4	-4.35056058526948 (0.000156162608382537)	0	1.77077635375 (0.00295208093227759)	0	0	0	0	0	-4.68471123139509 (9.89583987230663e-07)	0
PECAM1	0	0	0	0	0	-2.73723735025825 (5.24747035061447e-06)	0	-2.22741409981447 (0.00267344357459886)	0	0
PECR	0	0	0	0	0	3.01727923748837 (0.000144640268896473)	0	0	0	0
PEG10	0	0	-5.36052267281414 (4.22893222826044e-12)	0	0	-3.92367755619295 (0.000500469114321787)	0	-8.87033077716165 (0.000103922737255945)	3.09197734731765 (0.000664215675868777)	-4.59637053230621 (3.81462580244423e-05)
PEG3	1.90644236154432 (3.39955715510647e-06)	0	0	2.51471320786165 (0.000704566209424815)	1.82819660518826 (0.00610149374141091)	1.97138868640101 (1.71639718240182e-05)	0	0	5.98249450229011 (1.4707359358219e-46)	0
PELI1	0	0	0	0	0	-2.01190403467396 (0.00200745763171111)	0	0	0	0
PELI2	0	0	-1.9322230526505 (0.000521709863661433)	0	0	0	0	0	0	0
PELI3	0	0	0	0	2.78180496680741 (0.00988635756849589)	0	0	0	0	0
PELP1	0	0	1.62680540947764 (0.00761048403933917)	0	0	0	0	0	0	0
PENK	4.8300447131341 (1.98792404274048e-05)	0	0	0	0	0	0	0	0	0
PER2	0	0	0	2.68459512074329 (0.00598907827989341)	0	0	0	0	0	2.63929864261785 (0.0067668234378051)
PER3	2.50244029149641 (9.15253082094063e-07)	0	0	3.00146784617458 (0.000727531914695991)	0	0	0	0	0	0
PERM1	0	0	6.12918118526316 (8.16587799970454e-15)	0	0	0	0	0	0	0
PERP	0	0	0	0	0	0	0	-7.05938747178876 (0.00134107614358181)	2.24300690821934 (7.00750480054635e-06)	4.43779104187264 (1.06047857647501e-09)
PET100	0	0	2.34414271346163 (0.0037043058427508)	0	0	0	0	0	0	0
PEX14	0	3.50012538039745 (0.000818576599828903)	0	0	0	0	0	0	0	0
PF4	0	0	0	0	0	0	3.51545756910593 (0.00210057771059418)	6.9052044368901 (4.81247477592868e-08)	0	0
PFDN4	1.67908706333412 (0.00583814599389157)	0	0	0	0	0	0	0	0	0
PFKFB1	0	0	0	0	0	6.38374668413308 (0.00293189884976945)	0	0	0	0
PFKFB2	-1.56697868301748 (0.000594156949183217)	0	0	0	0	0	0	0	-1.7940851393987 (0.000149842091461326)	0
PFKFB3	-2.0463611680701 (0.000150950396304929)	0	-2.30525750255911 (4.78989223898299e-14)	0	0	0	0	0	-2.488201191189 (1.95524471516539e-13)	0
PFKFB4	0	0	-2.57848703301208 (0.00226836285584128)	0	0	0	0	0	0	0
PFKM	0	0	2.85381547100994 (8.01774052069031e-09)	0	0	0	0	0	0	0
PFN2	1.71404036417479 (0.000287502016851121)	0	0	0	0	0	0	0	0	0
PGA3	0	0	0	0	6.84195538055943 (0.00207576517305724)	0	0	0	0	0
PGAM5	0	0	0	0	0	2.34969969082227 (0.00307461412929913)	0	0	0	0
PGAP1	0	0	0	0	0	0	0	-6.57462527337881 (0.00252247576107562)	0	0
PGBD2	0	0	0	0	0	0	0	0	1.58968573134132 (0.006825277577096)	0
PGBD5	0	0	3.03655874458928 (0.00613554935636054)	0	0	5.23790769443962 (0.000204023861169767)	0	0	0	0
PGC	-6.83847040873651 (2.72970079651663e-06)	0	-7.23902411025848 (1.17859864324272e-11)	-8.78172467456277 (0.000161902787554748)	0	-3.90167980600453 (0.00200580145489407)	0	-7.72407995794512 (0.00094638921479757)	-8.99713528613998 (1.36598806776231e-11)	-9.06096878294203 (9.95498369907813e-05)
PGD	-1.71179913666685 (0.00498160573613314)	0	-1.50172190575617 (0.00693325266934222)	-2.39143074159285 (0.00631437341435685)	0	0	0	0	-1.68776072407687 (3.74328575032754e-05)	0
PGF	0	0	0	0	0	0	0	0	3.28368363355803 (0.0010091920882294)	0
PGLYRP1	0	0	0	0	0	0	0	6.85503839948126 (5.43353995522359e-06)	0	0
PGLYRP2	0	0	0	0	0	6.79379754205713 (0.000749107744748716)	0	0	0	0
PGM1	0	0	2.7561480551899 (3.96584913142718e-11)	0	0	2.13909465235615 (0.00273923147787163)	0	0	0	0
PGM3	0	0	0	-2.8643838764134 (0.00267499804523905)	0	0	0	0	0	0
PGM5	5.24120375962445 (6.35914254078577e-28)	0	3.03386116969359 (3.31901543695902e-08)	5.39590718126235 (4.31734704437101e-12)	0	0	0	0	0	0
PGM5-AS1	5.86421346648783 (1.33623898783005e-06)	0	0	0	0	0	0	0	0	0
PGR	1.8236687707246 (0.00525442693635404)	0	0	0	0	0	0	0	0	0
PGRMC2	0	0	0	0	0	0	0	0	1.78723341982883 (1.69808484130285e-08)	0
PHACTR1	-2.04349606228376 (4.64234013784237e-06)	0	0	-2.70588451924294 (0.00200668066603197)	0	0	0	0	-2.57982437423088 (3.20453524752731e-08)	0
PHACTR2	0	0	0	0	0	0	0	0	2.38391176638469 (5.14249805176926e-07)	0
PHEX	-2.77877921279348 (0.00764659971545675)	0	-3.36796602985611 (0.00383642850871726)	0	0	0	0	0	-2.47217821961201 (0.00539002305870178)	0
PHF21B	0	0	0	0	0	6.82983796954413 (0.00046085844085267)	0	0	0	0
PHGR1	4.88627846097617 (0.00146763101928722)	0	0	0	0	0	0	0	0	0
PHIP	0	0	0	0	0	-1.56961100895716 (0.0084255564458586)	0	0	0	0
PHKA2	0	0	0	0	0	2.46656198315366 (9.38108053002811e-05)	0	0	0	0
PHLDA1	0	0	-1.66549276548014 (0.000304710689550852)	0	0	0	0	-3.91275036171923 (0.000539037041628501)	0	0
PHLDA2	0	0	0	0	0	0	0	0	2.78959977677564 (1.53759006376441e-05)	0
PHLDB1	0	0	0	0	0	0	0	-2.58693585988433 (0.00595400202185889)	0	0
PHLDB2	0	0	0	0	0	0	0	-2.892968119335 (0.000482734243968061)	2.11154569269162 (2.02653388113942e-09)	0
PHLDB3	0	0	0	0	2.61201502486241 (0.00205001447386545)	2.35496563731268 (0.00384854017526117)	0	0	0	0
PHLPP2	0	0	0	0	0	1.73394212379083 (0.00820254399979163)	0	0	0	0
PHOX2B	5.53566870876277 (5.35158303873562e-05)	0	0	0	0	0	0	0	0	0
PHYHD1	0	0	0	0	0	2.96747326050519 (0.0058933983103423)	0	0	0	0
PHYHIP	3.89371936460721 (2.08194868628252e-05)	0	0	0	0	5.0114647063928 (0.000550365593828656)	0	0	3.89969274236839 (0.000119274142443227)	0
PHYHIPL	0	0	0	0	0	0	0	0	5.89464735264734 (0.000490639318610333)	0
PI15	0	0	0	0	0	0	0	-8.62643798004147 (0.00456691854401149)	-4.19175550254733 (2.0566791299829e-08)	0
PI16	0	0	2.6776400580204 (0.00682373771099662)	0	0	0	0	0	0	0
PI4K2B	0	0	-2.46051834763945 (0.000170834127249733)	0	0	0	0	0	0	0
PI4KAP1	0	0	0	0	0	0	0	0	2.21373734118812 (0.00694760875423849)	0
PI4KAP2	0	0	0	0	0	0	0	0	1.62264954338086 (0.00303187644774669)	0
PIANP	3.70246950999149 (0.00367002826146727)	0	0	0	0	0	0	0	0	0
PID1	1.66679969036975 (6.78255254633994e-07)	0	0	0	0	0	0	-5.78782682784303 (0.00872404826248463)	0	0
PIEZO2	0	0	-6.39140007781931 (2.79410101761817e-13)	0	0	0	0	0	-4.13854669307496 (1.20683296581684e-09)	-3.41982103252756 (0.000988807716794081)
PIGA	0	0	0	-6.13175557463457 (0.00724049653094246)	0	0	0	0	0	0
PIGP	1.79193920167307 (0.00377425224956757)	0	0	0	0	3.14271859625733 (0.00344344832045127)	0	0	0	0
PIGQ	0	0	0	0	0	0	0	3.02393319443681 (0.00249399411410954)	0	0
PIGR	-2.21774543180991 (0.000784639287584322)	-5.87249447049199 (0.0094406914029784)	-4.98241337748574 (0.000800065532373119)	0	0	0	0	-6.89122340617296 (8.37989221950978e-05)	-6.93115034748778 (3.12879276253586e-20)	-10.6730661752905 (2.51073908951986e-06)
PIK3AP1	-2.12188689072691 (0.000971904553211327)	0	0	-4.56636679813228 (0.000422397831969442)	0	0	0	0	0	0
PIK3C2G	0	0	0	0	0	6.56327051358552 (3.39242667512034e-07)	0	0	0	5.72053152823252 (0.00362691024918359)
PIK3CA	0	0	0	0	-2.11796831699008 (0.00527457229729232)	-1.61779741787059 (0.00550144935219438)	0	0	0	0
PIK3CD	0	0	-1.64199373819972 (0.00214299719259123)	0	0	0	0	0	0	0
PIK3CD-AS1	0	0	0	0	0	5.81147881483206	0	0	0	0

						(0.000446112475233785)				
PIK3CG	0	0	0	0	0	0	1.61504457113533 (0.00202937757272469)	0	0	0
PIK3IP1	0	0	-2.09234830582797 (0.000305449892694155)	0	0	0	0	0	-1.51719199132489 (5.22852281811732e-06)	0
PIK3R1	0	-6.0205114135952 (0.00444294096659968)	0	0	0	-1.52189497930743 (9.79936407671341e-05)	0	0	0	0
PIK3R3	0	0	0	0	0	0	0	0	-1.57221727626375 (0.00108234849175562)	0
PIK3R5	-2.74092888835205 (1.69755365681754e-06)	0	-3.50375814180158 (8.72309244090822e-10)	-3.10000652127312 (0.000308089607818575)	-2.09001227624909 (0.00669737431801659)	0	0	0	-2.75022900586364 (1.34701970156098e-08)	0
PILRA	0	0	-3.41464000178405 (0.000590118912430406)	0	0	0	0	0	0	0
PIM2	0	0	-3.5924114726768 (0.000119374909335763)	0	0	0	0	2.97295436739822 (0.00397406281568862)	0	0
PIM3	-2.06328048876246 (2.46513610826654e-07)	0	0	0	0	0	0	-3.24821416760259 (0.00221873683017446)	-1.68340778602209 (4.82896915635356e-06)	0
PIN4	2.22773785712852 (0.00353091612281631)	0	0	0	0	0	0	0	0	0
PINK1	0	0	2.49588951069271 (2.29965840899662e-09)	0	0	0	0	0	0	0
PIP5K1B	0	0	0	0	0	0	0	2.7867541495666 (0.00613542256797057)	0	0
PIPOX	0	0	4.50005546179906 (0.00315774332559296)	0	0	7.69406881606524 (1.29094620864496e-09)	0	0	3.70986013448612 (0.000927194557314927)	0
PIPSL	0	0	0	0	0	7.27455352988058 (0.000272337576168823)	5.05768176283075 (0.00211269287288671)	0	0	0
PIRT	4.91668814194211 (0.00013119784964086)	0	0	0	0	0	0	0	0	0
PISD	0	0	0	0	0	0	0	0	1.56413483148525 (1.91313074769784e-06)	0
PITPNM1	-2.18708305810902 (0.00196657254211266)	0	0	0	0	0	0	0	0	0
PITPNM3	0	0	-2.98594322577525 (0.000994802672631849)	2.68244244155887 (0.000337187791662689)	0	0	0	0	-1.62255639390278 (0.00114804353844482)	0
PITX2	0	0	0	0	0	8.67534440643214 (4.43069159022655e-06)	0	0	7.71806734942669 (1.15070670344146e-14)	0
PIWIL1	0	0	0	0	0	7.43707984036751 (0.000759745408992322)	0	0	0	0
PJA2	0	0	0	0	-1.51914013963452 (0.00398134573824911)	0	0	0	0	0
PKD1L1	0	0	3.01810701705783 (4.36921454508022e-05)	0	0	3.16227347258629 (0.00519793974426849)	0	0	0	0
PKD1L2	0	0	0	0	0	6.87917493948098 (3.41386220108513e-05)	4.83057899256072 (0.0060742015724898)	0	5.5345608528123 (1.87334621712919e-06)	0
PKD1L3	0	0	0	0	0	5.4902011243577 (0.000601182074429007)	0	6.17639469704712 (0.0011481007548893)	0	0
PKDCC	0	0	0	0	0	0	0	0	-1.8036615279954 (0.00750743369711101)	0
PKHD1	0	0	0	0	7.16587675681357 (8.78799410050372e-12)	6.00342088060124 (1.899247386394e-07)	0	0	0	0
PKHD1L1	0	0	-2.63285022143621 (0.000322985450207582)	0	0	0	0	0	0	0
PKIA	0	0	3.05686003148353 (1.8212720039179e-06)	0	0	0	0	0	-2.92104970139076 (6.83687500545176e-05)	0
PKIB	0	0	0	0	0	0	0	0	5.69714234481074 (1.60806520624725e-05)	0
PKIG	0	0	0	1.73479621153721 (0.00455473577856278)	0	0	0	-3.12805318615936 (0.00323028826464101)	0	0
PKLR	0	0	0	0	0	6.35811806389164 (6.65494523376561e-06)	0	7.93922715000964 (3.850936263791e-07)	0	0
PKN2-AS1	0	0	0	0	0	5.34612723395606 (0.0071336073727304)	0	0	0	0
PKN3	0	0	0	0	0	0	0	0	-2.35705220426866 (0.00129742296159586)	0
PKNOX2	-2.28721174988822 (0.00324159429217946)	0	0	0	0	0	0	0	0	-6.76389990571885 (0.00416766214812035)
PKP1	-5.07239353253327 (5.26984798555047e-06)	0	-2.93059445450845 (0.00207908249213601)	0	0	0	0	0	0	3.6524638032031 (0.000182806207411396)
PKP2	3.5147870459391 (9.17837978049551e-07)	0	6.70524478612066 (1.17748768902601e-25)	0	0	3.31927652716202 (0.000859060974676885)	0	0	5.79423454712328 (1.81973913352078e-20)	0
PKP4	0	0	0	0	0	0	0	1.98909669538598 (0.00424354312116631)	0	0
PLA1A	-3.34864030753154 (0.00685648872865101)	0	0	0	0	0	0	0	0	0
PLA2G12B	0	0	0	0	0	6.42625184625691 (0.000248747590754231)	0	0	0	0
PLA2G15	0	0	2.01354622745981 (0.00207806271063718)	0	0	0	0	0	0	0
PLA2G2A	3.64615531630226 (2.63541204506991e-10)	0	4.36820752384969 (8.29607236794875e-05)	3.44470286905122 (0.000596849879326679)	2.728858900936 (0.00107232354357322)	5.02233175379756 (4.6286071693238e-14)	2.41203705869404 (0.0043047083092862)	0	0	0
PLA2G2C	4.34092059151978 (0.00521170623807936)	0	0	0	0	7.52044483565709 (0.000884606455906154)	0	0	0	0
PLA2G2D	0	0	0	0	0	6.32632531097076 (0.00143749478808557)	0	0	0	0
PLA2G2F	0	0	0	0	0	8.40711496779532 (0.0059208620103402)	0	0	0	6.55562925012554 (0.00657939198976023)
PLA2G4A	1.69241017743627 (0.0002712806387208)	0	0	0	0	0	0	0	0	0
PLA2G4B	0	0	0	0	0	0	0	0	-4.20738715663092 (0.00142996720258304)	0
PLA2G4E	0	0	0	0	0	0	4.77521989879238 (0.00675699499902973)	0	0	7.69053262622716 (8.49877981837869e-07)
PLA2G4E-AS1	0	0	0	0	0	0	0	0	0	6.92760369255036 (0.00148283747648552)
PLA2G4F	-5.72226983033458 (2.33633094009309e-07)	0	-3.70187862483281 (4.62742602879965e-05)	-6.92809668963551 (0.00213899790306005)	0	0	0	-5.87088481163568 (0.00960272717902097)	-6.7122024498368 (2.38278916872995e-12)	0
PLA2G5	0	0	5.83385379298332 (2.50925130656326e-12)	0	0	3.69958154551484 (0.0026555390774649)	0	0	4.4043816150641 (4.18893264208572e-06)	0
PLA2G6	0	0	0	0	0	2.53174662647212 (0.00558449229686516)	0	0	0	0
PLA2G7	-4.37483618052536 (0.00415476873801533)	0	-4.99962587144369 (8.66986894027994e-06)	0	-5.01831369395206 (0.00397065261832846)	0	0	0	0	0
PLA2R1	0	0	0	0	0	0	0	0	1.98686795195253 (0.00029462508808349)	0
PLAC1	0	0	0	0	0	0	0	0	6.81695377337731 (9.45002420409729e-05)	0
PLAC4	0	0	0	0	0	0	0	0	5.2932753448182 (1.09035196072192e-06)	0
PLAC8	0	0	0	-6.49726365177546 (0.00362482170316131)	0	0	0	3.24260086003902 (2.89310566763771e-05)	2.58170193490891 (2.72769339247931e-08)	0
PLAGL1	0	0	0	0	0	0	0	0	2.9454602430119 (1.27037766959854e-16)	0
PLAT	0	0	-2.05611476396754 (1.61203965759727e-05)	0	0	0	0	-7.58536765994168 (0.000489120427323806)	0	0
PLAU	-1.81778046968159 (0.00653875312899164)	0	0	0	0	0	0	0	0	0
PLAUR	-2.36519115283428 (0.000188210191358378)	0	-3.41325651199116 (1.21440525639624e-07)	-2.65176795859225 (0.00123049001408204)	-2.02603501842087 (0.00501539216965354)	0	0	-2.43896560189471 (0.00595699735964735)	-2.21383445256698 (5.92027544497906e-06)	0
PLBD1	0	0	0	0	0	0	0	2.83219354902499 (0.000836528041527276)	0	0
PLBD2	0	0	0	0	0	-1.8414056155595 (0.00955361739646603)	0	-3.25223675640014 (0.00233815079269036)	0	0
PLCB1	2.07621617465412 (3.99605497203038e-07)	0	0	2.40201563994022 (0.00209710459283137)	0	0	0	0	1.5369314136697 (0.00984633427769393)	0
PLCB2	-1.95250635205667 (0.000593768978935064)	0	0	0	0	0	0	0	-1.56324998138378 (0.0027764931223307)	0
PLCD1	-1.59394747120674 (0.00297244494767238)	0	-1.6049596824175 (0.00608412490169876)	0	0	0	0	0	0	0
PLCD3	0	0	3.02037355792311 (3.61243063581597e-06)	0	0	0	0	0	0	0
PLCD4	2.43145705832145 (0.000756199340843067)	0	0	0	0	0	0	0	0	0
PLCE1	0	0	0	0	0	0	0	-6.46441710102858 (0.00411692169092907)	0	0
PLCG2	0	0	0	0	0	0	0	2.28072032199421 (0.00657575537492074)	0	0
PLCH1	-4.42818391568449 (0.000123275617770787)	0	-2.71819020830427 (0.00739707012214702)	0	0	0	0	0	-2.6725344625796 (0.000181842455690966)	0
PLCH2	0	0	0	0	0	4.79485158952379 (4.24282804174397e-07)	0	0	0	0
PLCL1	2.50319419996676 (2.95430236008165e-06)	0	3.86677067408991 (1.08775031360372e-15)	0	0	0	0	0	2.59150537322918 (5.80187682273142e-07)	0
PLCL2	-1.78515248904843 (0.00102375125483684)	0	0	0	0	0	0	0	1.9226228142481 (0.000240118978194218)	0
PLCXD1	0	0	0	0	0	2.0183939703926 (0.00129674765564098)	0	0	0	0
PLCXD2	0	0	0	0	0	0	0	0	2.62464981317576 (0.00610706941418128)	0
PLCXD3	0	0	4.44935335067016 (4.90306440881117e-13)	0	3.81275224920569 (6.09508882776688e-06)	2.88740283230456 (0.0087330538143662)	0	0	0	0

PLD1	0	0	0	0	0	0	0	2.09097787171567 (0.00852132169938083)	1.57096458202043 (2.26036028052291e-05)	0
PLD3	0	0	0	0	0	0	0	-2.57537518396315 (0.00375361449166139)	0	0
PLEK	-1.98566305786843 (0.00152248471509072)	0	-2.60138446125476 (0.000199988141001791)	0	0	0	0	0	0	0
PLEKHA1	0	0	0	0	0	0	0	-4.83932027400174 (0.00305099673649419)	0	0
PLEKHA4	0	0	0	0	0	0	0	-2.7103957475315 (0.000975029993214952)	-1.68209226783564 (4.81345271386492e-05)	0
PLEKHA6	0	5.05179680314138 (1.58869145053204e-07)	1.99574499564628 (0.000290718651726737)	0	2.49602789142436 (0.00343322434659744)	2.08161539417833 (0.00307577743399737)	0	0	3.32973747723255 (4.73953070468603e-06)	0
PLEKHA7	0	0	0	0	0	1.64057144395058 (0.00805388186425666)	0	0	0	0
PLEKHB1	0	0	0	0	0	0	0	0	-4.19196525728536 (0.000106391665990663)	0
PLEKHD1	0	0	0	0	5.83004520490158 (0.00374383828643843)	6.6428586881603 (0.000134224949147284)	5.03506069633756 (0.00219230244615493)	0	0	0
PLEKHG1	-1.67039191888053 (8.35487321571373e-05)	0	0	0	0	0	0	-2.71873769094466 (0.00682267221216151)	0	0
PLEKHG4B	0	0	0	0	0	4.6141223187741 (0.00049753237743054)	0	0	3.47329387386974 (7.88795927309073e-05)	0
PLEKHG5	0	0	2.85016214236854 (3.07230239735147e-10)	0	0	0	0	0	1.77273867963653 (0.00118233629823058)	0
PLEKHG6	0	0	0	0	0	0	0	0	5.62980732210522 (6.17937705853376e-08)	0
PLEKHH1	0	0	0	0	0	2.7937510211749 (0.00121257678507999)	0	0	3.31201978236375 (0.000188200449836463)	0
PLEKHH2	0	0	-3.62173708716059 (4.11523489151425e-19)	0	0	-2.21245581138652 (1.47862478537319e-05)	0	-2.81348166330655 (0.000314543776481782)	0	0
PLEKHH3	0	0	0	0	0	0	0	4.72564358225069 (1.9018220185349e-06)	0	0
PLEKHM1P	-1.85160721402498 (0.00319469277097157)	0	0	0	0	0	0	0	0	0
PLEKHO1	1.76601890514666 (8.43622810842875e-06)	0	0	0	0	0	0	0	0	0
PLEKHO2	0	0	0	0	0	-1.89758746901108 (0.00109461751262235)	0	0	0	0
PLEKHS1	0	0	0	0	0	0	0	0	-6.58336001373987 (0.000261010813533115)	0
PLG	0	0	0	0	7.63071255873095 (9.99301542249103e-05)	11.4721435254958 (5.71321556729087e-21)	0	0	0	0
PLIN1	0	0	7.55270744053697 (2.7037992588392e-09)	6.91881011234931 (0.00173281350374006)	7.58892958358567 (4.17191429440613e-05)	7.6353950489166 (1.48348770611978e-07)	5.0691711018079 (0.00232695176057603)	0	0	9.70659407937451 (1.7842291734826e-13)
PLIN2	0	0	0	0	0	0	0	0	-1.59823601986279 (0.000970445477991477)	0
PLIN4	4.78834670357608 (1.0890114746281e-20)	0	6.22237555487668 (1.02539264820734e-33)	5.14193683334201 (6.08262215588394e-09)	3.78075243796121 (0.000127324866137528)	3.77413576995905 (1.85657467197598e-07)	0	0	0	6.31873485100457 (2.51692626100121e-15)
PLIN5	0	0	2.54537490476027 (3.94615521287677e-06)	0	0	3.4567789724317 (6.59713090360597e-07)	0	0	-4.14460507648422 (0.000151156243478748)	0
PLK1	0	0	0	0	0	0	0	3.74057358723077 (0.00258513664198572)	0	0
PLK2	-1.50758483904139 (0.00331639084807255)	0	0	0	0	0	0	-5.36735865262715 (0.00110340646277504)	0	0
PLK3	-2.13038844958714 (0.00030117401538641)	0	0	0	0	0	0	0	0	0
PLK4	-4.2816721686679 (0.00354961310523007)	0	0	0	0	0	0	0	0	0
PLN	4.43887280894444 (3.92782788009546e-15)	0	4.05287407725141 (1.45170448169457e-18)	3.52013099402912 (1.63926342764793e-05)	0	0	0	0	0	0
PLOD2	0	0	0	0	0	0	0	-3.35971301425565 (0.000155669543084925)	0	0
PLP1	7.69067435091345 (7.82387354366025e-13)	0	0	7.48846806504865 (1.43943735324252e-07)	5.06228327902491 (0.00644739972663622)	0	0	0	0	0
PLS1	0	0	-2.83518222510901 (0.00140598852623403)	0	0	0	0	0	-3.21162139581749 (7.07965313386246e-05)	0
PLS3	-1.50139950061546 (0.000248692720701876)	0	-1.7342274404803 (0.000119570335391192)	0	0	0	0	-5.14088688736257 (0.00147029324740502)	0	0
PLVAP	1.9697316109273 (0.000125680139884116)	3.79765861336463 (4.02003278117565e-05)	0	2.70743101780089 (0.00132454220098608)	3.37118225097658 (9.63201766941067e-06)	0	1.71751441308445 (0.00439677087541442)	0	0	3.19176999075036 (0.000102089712020608)
PLXDC1	2.32238294471388 (0.000668904689056453)	0	2.69723023870454 (0.000177526852551982)	0	0	0	0	0	0	0
PLXDC2	0	0	0	0	0	-1.55865803802924 (0.00199911090782814)	0	-4.03085081000582 (4.90769968954362e-06)	-1.5631815365006 (0.000194425533693678)	0
PLXNA2	-2.745462745155 (3.87817828078086e-06)	0	0	0	-2.74140422813162 (0.0013156889553798)	0	0	-8.98108901383402 (6.88143740882782e-05)	-1.60086093681655 (0.00327556725771626)	-3.01651155836591 (0.00203093293337855)
PLXNA4	0	0	1.65023811557518 (0.00100506791889106)	0	0	0	0	0	1.6636007589895 (0.00506586239960475)	0
PLXNB1	0	0	0	0	0	0	0	-4.73618147686706 (0.00421626861701054)	0	0
PLXNB2	0	0	0	0	0	0	0	-2.16355697652533 (0.00329782573111604)	0	0
PLXNB3	3.05604022177566 (0.00207480584772549)	0	0	0	0	0	0	0	0	0
PLXNC1	-2.65644484799315 (6.45100390997541e-08)	0	-2.91133480109387 (1.03153043511046e-07)	0	-2.4190405912545 (0.00688287163745792)	0	0	0	0	-2.21125855114115 (0.00885043378953708)
PM20D1	0	0	0	0	0	0	0	0	0	5.77450783039849 (0.00117906369808154)
PMAIP1	-3.68050744740454 (0.00187098057503267)	0	0	0	0	0	0	0	0	0
PMEL	0	0	0	0	4.31907178245531 (0.00742260572213725)	0	0	0	0	0
PMEPA1	0	0	0	0	0	0	0	-6.53507022206756 (0.00314478756363169)	0	0
PMP22	0	0	0	0	-1.61786019886626 (0.00378471361964082)	-2.0718714061526 (0.000137011783134446)	0	-3.30527710291888 (8.04519319030952e-05)	0	0
PMVK	0	0	1.57214661257218 (0.00683519106574162)	0	0	0	0	0	0	0
PNCK	7.71135966611196 (1.75641596548941e-10)	0	0	8.50378687226566 (4.32084327343579e-08)	0	0	0	0	6.86469904684635 (3.80570327889178e-08)	0
PNKP	0	0	0	-4.32894310675918 (0.00986085681999752)	0	0	0	0	0	0
PNMA1	1.85103987483201 (0.000309287051198271)	0	0	0	0	0	0	0	0	0
PNMA3	0	0	0	0	0	5.72739478743657 (0.00499523430321242)	0	0	0	0
PNMA5	0	0	0	0	0	4.77700090555691 (0.00833956504093743)	0	0	0	0
PNMAL1	3.62567151402208 (0.000362445577676348)	0	2.94327810775481 (0.00998056721028704)	0	0	0	0	0	0	0
PNP	0	0	0	0	0	0	0	1.7841703216494 (0.0081204796842691)	0	0
PNPLA2	0	0	2.59271269214652 (3.1726446924749e-07)	0	0	0	0	0	0	0
PNPLA3	0	0	0	0	0	8.92189056144112 (2.46129105723111e-13)	0	0	0	0
PNPLA6	-1.53307296403671 (0.00392239251838186)	0	0	0	0	0	0	0	-1.64881332411706 (0.000599021094970383)	0
PNPO	0	0	0	0	0	2.71593220663909 (0.000572419216258542)	0	0	0	0
PNRC2	0	0	0	0	0	-1.54681849001661 (0.00664401304589147)	0	0	0	0
PODN	0	0	0	0	0	0	0	-4.37087259826161 (0.00134370361646468)	-2.34729008462653 (1.12723434610371e-06)	0
PODXL	-3.36057291218013 (9.90274890436289e-17)	0	0	-2.93289460249708 (1.33366231261324e-05)	-1.82638211025752 (0.00243246142296296)	-3.37923480417136 (3.80644241605325e-08)	0	-5.47660339122536 (2.29164210389433e-10)	-2.7114332996719 (7.61240777860939e-15)	-2.03721235432116 (0.00217546620723289)
PODXL2	0	0	0	0	4.28245449361695 (0.000774490046771751)	0	0	0	0	0
POF1B	0	0	0	0	0	0	0	0	2.38740316522865 (2.71690561994799e-05)	7.14245199061157 (6.31881396598808e-17)
POLD2	0	0	1.79022431903447 (0.000335605540303897)	0	0	0	0	0	0	0
POLE2	0	0	0	0	0	0	0	4.30760736069314 (0.00846921048229098)	0	0
POLQ	0	0	0	0	0	0	0	4.06885727900932 (0.000369241501045362)	0	0
POLR2A	0	0	0	0	0	-1.6729283399906 (0.000726505821585908)	0	0	0	0
POLR2B	0	0	0	0	-1.56577586134584 (0.00754596611644158)	0	0	0	0	0
POLR2H	0	3.00702852440022 (0.00820089565429221)	0	0	0	0	0	0	0	0
POLR2L	0	0	1.81041411180587 (0.000368080284893655)	0	0	0	0	0	0	0
POM121L9P	0	0	-3.2372893255761 (0.00707309287732058)	0	0	0	0	0	-4.7375194118409 (1.24794923026907e-05)	0
POMGNT1	0	0	2.34715634104876 (4.81322703497089e-05)	0	0	0	0	0	0	0
POMGNT2	0	0	0	0	0	2.46214624285556 (0.00452806097649151)	0	0	0	0
PON1	0	0	0	0	0	5.81717386455783	0	0	0	0

						(0.0072313700811003)				
PON2	0	0	-2.13466073801852 (3.08957897469788e-07)	0	-2.15827962450747 (0.00179740429812539)	0	0	0	0	-1.96413401528332 (0.00481621893214097)
PON3	0	0	0	0	0	2.597159799646 (0.00172011876597814)	0	0	-2.9020564190773 (0.00355521381063275)	0
POPDC2	5.00552763907538 (1.74029104859115e-10)	0	8.11587365247061 (6.86979120836857e-27)	5.54155690237461 (6.43566300225786e-05)	0	4.23283276899368 (0.00431323795739229)	0	0	0	0
POPDC3	4.38985213476785 (0.00580347495484278)	0	6.1796462174509 (5.47019950499905e-07)	0	0	0	0	0	0	0
POR	0	0	0	0	0	1.96990012331654 (0.00156221434866817)	0	0	0	0
POSTN	0	0	-2.41631989097794 (0.00366322852948481)	0	0	-5.0719979214866 (3.54254517668393e-05)	-1.82371226102624 (0.00426936652349802)	-9.2546281379186 (0.00336513597955671)	0	0
POTEE	0	0	0	0	0	0	0	0	2.36848691271082 (0.00117025082444946)	0
POTEF	0	0	0	0	0	0	0	0	3.57515596652337 (0.00504300541079406)	0
POU2AF1	0	0	0	0	0	0	2.90127485556419 (0.000263232543826061)	0	0	0
POU2F3	0	0	0	0	0	0	0	0	0	4.44826888827851 (0.00452714813233527)
POU3F3	0	0	0	0	8.14243811854279 (1.41386020302993e-11)	6.46029672410387 (3.4945840971523e-05)	0	0	0	0
POU5F1	-4.71885670224782 (0.00309494078436371)	0	0	0	0	0	0	0	0	0
POU6F1	0	0	0	0	0	0	0	0	-2.05583618674341 (0.0019721111948367)	0
POU6F2	0	0	0	0	5.89016098644507 (0.0034468858259432)	7.19293781088597 (1.68321734975506e-05)	0	0	0	0
PP14571	0	0	0	0	0	0	0	0	5.72605492060073 (6.86412390268833e-09)	0
PP7080	0	0	0	0	3.42856379231702 (0.00975832863400708)	0	0	0	0	0
PPAP2A	0	0	0	0	1.97862305288007 (0.00869866933428391)	0	0	0	0	0
PPAP2B	0	0	0	0	0	0	0	-3.21038398289788 (0.000528732026453956)	0	0
PPAP2C	0	0	0	0	0	0	3.13725089592837 (0.00164367027294553)	0	0	0
PPAPDC2	0	0	0	0	0	2.04134979577187 (0.00739529253245879)	0	0	0	0
PPAPDC3	0	0	5.35720560366334 (2.48979799527534e-09)	0	0	0	0	0	0	0
PPARA	0	0	2.33523286340583 (1.94005518493337e-08)	0	0	2.91413749501607 (1.05479641519847e-10)	0	0	0	0
PPARGC1A	0	0	2.07046477090976 (2.64400547412194e-05)	0	0	3.20794806353804 (0.00350303737799596)	0	0	-4.05514420664233 (2.26478914681579e-06)	0
PPARGC1B	0	0	0	0	0	2.16031797786342 (0.00868474334963479)	0	0	0	0
PPBP	0	0	0	0	0	0	2.86459809582197 (0.0060094878101056)	5.13996693862396 (0.000430026666275349)	0	0
PPFIA2	0	0	0	0	0	0	0	0	0	3.75032418033155 (0.00353165585320401)
PPFIA3	0	0	0	0	3.74916736967973 (0.00110778907214497)	4.75172678698878 (0.000231254839030239)	0	0	0	0
PPFIA4	0	0	3.44331633484056 (5.05092660601866e-05)	0	0	0	0	0	2.35151257963819 (0.000773214523458292)	0
PPFIBP1	0	0	0	0	0	0	0	-3.17021467402221 (0.000138867860929271)	0	0
PPFIBP2	0	0	0	0	0	2.46735580886966 (9.50155571665499e-05)	0	0	1.66300844642921 (4.42100999125782e-05)	0
PPIAP22	0	0	0	0	0	0	0	0	-1.86364195355408 (0.00198208447751578)	0
PPIL4	0	0	0	0	-2.31756639666033 (0.00334746190063651)	0	0	0	0	0
PPL	0	0	0	0	0	0	0	-7.93185822818202 (0.00026516537896723)	0	2.38446830348551 (0.000733962741500443)
PPM1E	0	0	0	0	0	4.16064606610248 (0.00313533691020884)	0	0	0	0
PPM1H	0	0	0	0	2.44369846763295 (0.00411809062084795)	2.26398946658353 (0.00456647379065009)	0	0	2.26956989348374 (5.93835853644137e-06)	0
PPM1K	0	0	3.41690884897607 (6.78163783399014e-17)	0	0	0	0	0	0	0
PPM1L	2.53923367134156 (2.17042173386964e-07)	0	0	0	0	0	0	0	0	0
PPOX	0	0	0	0	0	0	0	3.80587788599148 (0.000789337045222338)	0	0
PPP1R12B	3.06607513767174 (2.45720279932345e-10)	0	3.03257031673431 (3.13366894887652e-08)	3.02790809930947 (0.000127702901618976)	0	0	0	0	0	0
PPP1R12C	0	0	2.05567878636253 (6.94920764508746e-05)	0	0	0	0	0	0	0
PPP1R13B	0	0	1.5925450099282 (4.64584220690377e-05)	0	0	0	0	0	0	0
PPP1R13L	-2.22132763574435 (0.000199702375118828)	0	0	0	0	0	0	0	0	0
PPP1R14A	0	0	0	0	0	0	0	0	-1.53037540497903 (0.00494281922450519)	0
PPP1R14C	-2.44137613370592 (0.00797948958464891)	0	3.50162788608745 (5.51791137870153e-10)	0	0	0	0	0	0	3.10114621054408 (0.0011353364170387)
PPP1R15A	-2.5968372675737 (0.000129704322713979)	0	0	0	0	0	0	0	0	0
PPP1R16A	0	0	2.6001819025119 (0.0028999882878762)	0	0	3.02500098121626 (0.00451595105024666)	0	0	0	0
PPP1R1A	7.33938873115249 (1.6089739821358e-11)	0	8.66235057086634 (1.08472995320686e-15)	8.63154302494878 (1.34826464285994e-08)	8.81273965746902 (1.81870466409975e-13)	8.07646062563179 (8.19676825954065e-06)	4.9415328645231 (0.00317803400292502)	0	0	7.71610814515995 (1.79240991507195e-05)
PPP1R1B	0	0	-3.64175834987676 (0.00310330435792659)	0	0	0	0	0	-5.9013496121607 (8.68531475661616e-07)	0
PPP1R1C	0	0	3.98116854228935 (0.00732137516662276)	0	0	0	0	0	0	0
PPP1R35	0	0	0	0	3.96085259179772 (0.00137411842527894)	0	0	0	0	0
PPP1R3A	0	0	8.07236372885223 (1.01398258879799e-13)	0	0	0	0	0	0	0
PPP1R3B	0	0	0	0	0	0	0	-2.41830027378095 (0.00749589727939137)	0	0
PPP1R3F	0	0	4.43285636675833 (1.6721117068986e-09)	0	0	0	0	0	0	0
PPP1R3G	0	0	3.78664923612043 (0.00187621768733458)	0	0	4.69685244637699 (0.00434033205158643)	0	0	0	0
PPP1R9A	0	0	0	0	0	0	0	0	0	-2.90641408158763 (0.00524648695696319)
PPP2R2C	0	0	5.7831324169723 (4.60728081309915e-06)	0	7.13500379894683 (3.0976464632767e-05)	6.45245001434373 (0.00526022351803951)	0	0	0	6.26800475822342 (0.00280365280479471)
PPP2R3A	0	0	0	0	2.1059638040172 (0.000719803934504833)	0	0	-5.74781360556906 (0.00938676473375772)	0	0
PPP2R3B	0	0	3.57835202963455 (4.10831182072307e-11)	0	0	0	0	0	0	0
PPP2R4	0	0	1.57733834742017 (0.0012706982909298)	0	0	0	0	0	0	0
PPT1	0	0	-1.88508774629819 (0.000739118852614353)	0	0	-2.56266700919677 (0.00255908991929388)	0	0	0	0
PQLC1	0	0	0	0	0	1.69301874883297 (0.00836806500637937)	0	0	-1.78422484587526 (0.00281343164760609)	0
PRADC1	0	0	5.11863924417862 (2.89603873860834e-08)	0	0	0	0	0	0	0
PRAF2	0	0	0	0	0	0	0	0	-1.73690082812084 (0.000554185465727537)	0
PRAM1	-4.79440060038014 (5.03657908459604e-05)	0	0	0	0	0	0	3.64130102188613 (3.975040299403e-05)	-3.76925887270662 (5.42141995740046e-06)	0
PRAMENP	0	0	0	0	0	6.6011437087181 (0.00236873350522481)	0	0	0	0
PRAP1	0	0	0	0	0	7.66397146523196 (7.76445559392518e-10)	0	0	0	0
PRB3	0	0	0	0	5.2884415493075 (0.00914553860632148)	0	0	0	0	0
PRC1	-2.62006974042245 (8.07735626231707e-05)	0	0	0	0	0	0	4.85662153401257 (1.40349800223406e-11)	0	0
PRCD	0	0	0	0	0	4.32674153470141 (0.0013338953794454)	0	0	0	0
PRDM1	0	0	-1.84692779064262 (0.00101874714054603)	0	0	0	0	0	0	0
PRDM12	0	0	0	0	0	7.44830799101198 (0.000919267628349178)	0	0	0	0
PRDM14	0	0	0	0	0	6.5819059704344 (0.00420308949348701)	0	0	0	0
PRDM16	0	0	0	0	0	0	0	0	-2.55971432428801 (0.000746134398598461)	0
PRDM4	0	0	0	0	0	-1.81213613896886 (0.00428530710125407)	0	0	0	0
PRDM6	0	0	-3.60472871606039 (0.0049329502370106)	0	0	0	0	0	0	-6.30461788639802 (0.00938393027571382)

PRDM7	0	0	0	0	5.79712044871431 (0.000960887504490318)	0	0	0	0	0
PRDM8	2.69167194501782 (0.00373998853439962)	0	0	0	0	0	0	0	0	0
PRDM9	0	0	0	0	6.47559966567641 (0.0042482603748159)	0	0	0	0	0
PRDX2	0	0	1.80870002286054 (2.97873805569902e-05)	0	0	0	0	2.53413785460951 (0.0016017289046987)	0	0
PRDX3P1	-4.10276965291049 (0.00528851235926012)	0	0	0	0	0	0	0	0	0
PRDX4	0	0	0	0	0	2.13616595111874 (0.0041489510061723)	0	0	0	0
PRELP	1.59769130710161 (0.00206307047142217)	0	0	2.23528733377109 (0.0092493434783965)	0	0	0	-6.24968982440328 (0.000223719604432771)	0	0
PREX1	-2.23896728172921 (1.14230638170852e-06)	0	0	0	0	0	0	0	-1.90437651358511 (3.80311017926545e-07)	-2.23340883497881 (0.00235614101275688)
PRG2	0	0	0	0	0	0	0	0	10.5512696797101 (6.07643942945479e-26)	0
PRG3	0	0	0	0	0	0	0	6.97712931375118 (0.00214092686965207)	0	0
PRG4	-2.40771432701558 (0.000283675042018782)	0	-3.09846787384571 (0.00335738504974412)	-2.77809694655072 (0.00111301335218211)	-3.88448799246736 (5.05835880100655e-06)	0	0	-2.39968076201852 (0.00668120915191149)	-6.21909939251122 (1.9862784557935e-20)	0
PRICKLE2	1.84347708278479 (1.97531473791881e-05)	0	0	2.13173789281121 (0.00615994494128434)	0	0	0	0	0	0
PRIMA1	6.68738030551117 (1.28752664048486e-21)	0	0	6.56811342372065 (1.84341753385986e-09)	0	3.54528743379161 (0.00444424673092734)	0	0	2.77258212282899 (0.00699551156333755)	0
PRKAA2	3.08900588833233 (6.89676222320359e-10)	0	4.0150756738746 (2.2870407237755e-12)	3.40172026053099 (4.6521451221924e-05)	0	0	0	0	-2.05416144726057 (0.000325234028759493)	0
PRKACA	0	0	1.73672479420086 (6.72935188087369e-06)	0	0	0	0	0	0	0
PRKACB	1.6021461357404 (0.000179644879407923)	0	0	0	0	0	0	0	0	0
PRKAR2A	0	0	0	0	0	0	0	-2.98479364308667 (0.00228846025892217)	0	0
PRKAR2B	1.73041463979752 (0.000902818714765196)	0	0	0	0	0	0	2.64461687042608 (0.00243456496852956)	0	0
PRKCA	1.67198712390047 (5.91001454602796e-05)	0	0	2.08342647022339 (0.00575516611324512)	0	0	0	0	0	0
PRKCB	0	0	-2.78321565417624 (5.18829847850924e-07)	0	0	0	0	0	-2.00351010787454 (2.20879692029864e-06)	0
PRKCD	0	0	0	-2.19335801694019 (0.00799665825306328)	0	-2.54719234676077 (0.000718037387765848)	0	0	-1.56091662859884 (0.000132573727822853)	0
PRKCE	0	0	-1.59072731448575 (0.000172567740028429)	0	0	0	0	0	0	0
PRKCH	-2.05744476709955 (2.20907298129093e-05)	0	0	0	0	0	0	0	0	0
PRKCQ	0	0	0	0	0	0	0	0	-2.55153946213338 (0.00872864942358328)	0
PRKCZ	-2.84984504581907 (5.01862292510842e-06)	0	0	0	0	0	0	0	2.04614823874758 (0.00863134193912243)	0
PRKD3	0	0	0	0	0	-1.70055250746022 (0.00528703532705206)	0	0	0	0
PRKDC	0	-6.32531171434871 (0.00293164772906232)	0	0	-1.55542679135588 (0.000108253397381768)	0	0	0	0	0
PRKG1	0	0	0	0	0	0	0	-3.67999626219422 (0.00549607665398342)	0	0
PRKRIRP1	0	0	0	0	0	5.42652275839641 (0.00623406497658271)	0	0	0	0
PRKRIRP3	0	0	0	0	0	6.10460538280704 (0.00453511753877736)	0	0	0	0
PRKX	-1.57605527118424 (0.0085546536999457)	0	0	0	0	0	0	0	0	0
PRL	0	0	0	0	0	0	0	0	8.49479768669082 (6.95890694336632e-05)	0
PRLR	0	0	0	0	4.0840753157148 (0.00230147454957357)	4.37347295986962 (0.000256638742131506)	3.54876845444431 (0.00351820456078248)	0	9.58156094816643 (6.57244364159598e-48)	0
PRMT8	0	0	0	0	0	6.98874410046276 (0.00034326122856447)	0	0	0	0
PRND	0	0	0	0	6.65447770053204 (0.00254565647500638)	0	0	0	0	0
PRNP	0	0	0	0	0	0	0	-6.05797123857732 (0.000153920267232663)	0	0
PROB1	0	0	5.37594797001565 (1.03754886292242e-13)	0	0	0	0	0	0	0
PROC	0	0	0	0	0	7.90676951878266 (2.507406725836e-10)	0	0	0	0
PRODH2	0	0	0	0	0	7.18903353879792 (1.55929463677719e-07)	0	0	0	0
PROK1	0	0	0	0	0	0	0	0	6.3675447199474 (3.65852251577415e-09)	0
PROM1	0	0	0	0	0	0	0	0	3.69815018532331 (2.36362143347607e-05)	0
PROM2	0	0	0	0	2.88205837086056 (0.00159644370825212)	0	0	0	0	0
PROSER2	0	0	0	0	3.46584772827835 (5.81148197230379e-05)	2.69395343035556 (0.000699684087321323)	0	0	0	0
PROSER2-AS1	0	0	0	0	0	0	0	0	4.50465489543886 (1.57185317399922e-09)	0
PROX1	1.80145973881229 (4.04319496157593e-05)	0	2.73356046234484 (5.99348314011199e-10)	0	2.7490802732335 (5.06506524900212e-06)	5.62892054825475 (3.87455753703772e-14)	0	0	0	0
PROX1-AS1	0	0	0	0	4.59609050529168 (0.00880095582292758)	6.83107884408932 (3.72546926197659e-05)	0	0	0	0
PROX2	0	0	0	0	0	5.1395702113976 (0.000579710282883437)	0	0	0	0
PROZ	0	0	0	0	0	5.83186550604857 (2.35118863883036e-05)	0	0	0	0
PRPH	3.27522258308516 (0.00532220184237346)	0	0	0	0	0	0	0	0	0
PRR11	0	0	0	0	0	0	0	3.45829239857827 (0.0045794870987555)	0	0
PRR15	0	0	0	0	0	0	0	0	6.06102256878411 (7.13857910910976e-07)	0
PRR15L	0	0	-3.79657560080353 (0.00144110572897125)	0	0	0	0	0	-5.19973236566989 (8.8768526396846e-07)	0
PRR23B	0	0	0	0	0	6.38968800314438 (0.00475066945748178)	0	0	0	0
PRR26	2.02952105843395 (9.47841395690735e-07)	0	0	3.20875322771698 (5.85578107738657e-05)	0	0	0	0	0	0
PRR30	0	0	0	0	0	7.14469602815321 (0.00799193581954721)	0	0	0	0
PRR34-AS1	0	0	2.11344048252581 (0.00714748677691395)	0	0	0	0	0	0	0
PRR36	0	0	0	0	0	0	0	0	-4.50288320334387 (0.000559357983179774)	0
PRR5	0	0	0	0	0	2.88961084735693 (0.000929652712001496)	0	0	0	0
PRRG3	0	0	0	0	0	3.00134520306741 (0.00152747833996082)	0	0	0	0
PRRG4	-2.63741788407893 (1.20785204690702e-05)	0	-3.80048122942104 (9.00958602993956e-07)	0	0	0	0	0	0	0
PRRT1	4.06278449685776 (1.54857992903127e-06)	0	0	0	0	5.05575359915649 (0.00136959400356823)	0	0	0	0
PRRT2	0	0	-3.21553091344027 (0.000248765049969774)	0	0	0	0	0	-2.26952631302221 (0.000506575311569804)	0
PRRT4	0	0	0	0	0	0	0	5.19899009001349 (0.00818647817881995)	0	0
PRRX1	-2.24977946079574 (4.50851538785611e-05)	0	0	-2.60165322634256 (0.000923520502075636)	-1.91820685201604 (0.00483677067548544)	-2.1212043335738 (0.00613567858791527)	0	-6.45112052801653 (7.50288160851462e-05)	-2.51519125828643 (4.65049665339623e-07)	0
PRRX2	0	6.88216669998514 (1.50482790551013e-05)	0	0	0	0	0	0	0	0
PRSS1	0	0	0	0	6.01984881389889 (0.00219286599955368)	7.34187438316875 (5.83282237382239e-05)	0	0	0	0
PRSS12	0	0	-3.64419750850379 (0.000645591392636305)	0	0	0	0	0	2.54334300550453 (8.0700729499901e-08)	0
PRSS16	0	0	0	0	0	5.28314395643157 (0.000146881511038229)	0	0	0	0
PRSS23	0	0	-2.59983429380966 (1.42886417638195e-09)	0	0	0	0	-5.86327381867808 (0.000226683103814063)	0	0
PRSS27	0	0	0	0	5.24524375516253 (0.000115678657831732)	6.28617262057787 (9.79849268497065e-06)	0	0	0	0
PRSS3	0	0	0	0	0	6.16856019215855 (0.00609985852466005)	0	0	0	0
PRSS3P2	0	0	0	7.70492224287163 (9.90181358076235e-07)	0	0	0	0	0	0
PRSS42	0	0	4.67751928607143 (0.000696225179362544)	0	0	0	0	0	0	0
PRSS45	0	0	5.77123943280825 (0.000208067118257083)	0	0	0	0	0	0	0
PRSS57	0	0	0	0	0	0	0	6.80720155574357 (0.00424504638285965)	0	0
PRSS8	-5.98395057674316	0	-4.04878059810553	0	0	0	0	0	0	0

	(4.32059672884602e-06)		(0.000267491947508 89)							
PRTG	0	0	0	0	0	0	0	0	0	-6.16628522564387 (0.00847466875633612)
PRTN3	0	0	0	0	0	0	0	7.69906184234371 (0.0032633962331771)	0	0
PRUNE	0	0	1.71224329740675 (0.00312347558364477)	0	0	0	0	0	0	0
PRUNE2	4.77069733726738 (3.67012071470946e-39)	0	-1.97611590306169 (0.000861775295773615)	5.06464671718418 (2.90439495854484e-14)	0	0	0	0	0	0
PRX	-5.05473936676294 (0.000475677195065856)	0	-2.11329984642749 (0.00774951362594818)	0	0	0	0	0	-3.48820639120637 (8.75887702902994e-05)	-5.90552943274784 (0.00187869102368381)
PSAPL1	0	0	0	0	0	0	0	0	0	6.33191093972904 (0.000289149340253417)
PSD	4.54910694007148 (1.99130220668605e-08)	0	0	6.13118361222129 (8.99186207859033e-07)	0	0	0	0	0	0
PSD3	0	0	1.62579555273239 (4.41924829842294e-05)	0	0	1.98516893177876 (2.48052920257089e-05)	0	0	0	0
PSD4	-1.88163437008401 (0.000566822946999046)	0	0	-3.52225782044298 (0.00955376727928512)	0	2.29526614918206 (3.75432495126984e-06)	0	0	0	0
PSG1	0	0	0	0	0	0	0	0	10.4818766016263 (2.63145100455951e-09)	0
PSG11	0	0	0	0	0	0	0	0	10.4958279536318 (1.01812879859827e-08)	0
PSG2	0	0	0	0	0	0	0	0	8.91142740968565 (1.25641651607956e-07)	0
PSG3	0	0	0	0	0	0	0	0	11.3666600019954 (4.23780981649866e-10)	0
PSG4	0	0	0	0	0	0	0	0	11.452132852953 (1.69026854684106e-14)	0
PSG5	0	0	0	0	0	0	0	0	10.4703710077452 (5.69074635045613e-14)	0
PSG6	0	0	0	0	0	0	0	0	10.2960373813242 (3.19761014616935e-07)	0
PSG7	0	0	0	0	0	0	0	0	9.22231705032446 (2.37639563838819e-06)	0
PSG8	0	0	0	0	0	0	0	0	8.25434720774881 (0.000229575568502439)	0
PSG9	0	0	0	0	0	0	0	0	11.3379807381608 (1.54075791283095e-07)	0
PSMB10	0	0	0	0	0	0	1.91642453875772 (0.00036007992087276)	0	0	0
PSMB8	0	0	-1.66240996363902 (0.00419776224950763)	0	0	0	0	0	0	0
PSMD10P1	0	0	0	0	0	0	0	0	1.72419797894172 (0.00514774315307615)	0
PSMD9	0	0	0	0	0	2.09548900167329 (0.00928580938620484)	0	0	0	0
PSME4	0	0	0	0	-2.01490060416719 (0.00070736866877474)	0	0	0	0	0
PSORS1C2	0	0	0	0	0	0	0	0	0	5.06236954513265 (0.00315114487944353)
PSTPIP1	-3.26614370146491 (0.000223396409253692)	0	0	0	0	0	0	0	-2.67330177948987 (0.000382972353667077)	0
PSTPIP2	0	0	-1.94495899916834 (0.00590874008492949)	-3.47490116208379 (0.00946700716915285)	0	0	0	2.42159289018577 (0.00291364158661664)	0	0
PTAFR	0	0	-2.36032753331435 (0.00322050897066826)	0	0	0	0	0	-1.51139472179037 (0.00316238768782652)	0
PTBP2	2.21446589771014 (3.89206148018821e-07)	0	0	0	0	0	0	0	0	0
PTBP3	0	0	-1.81095818673877 (0.000223260074132981)	0	0	0	0	0	0	0
PTCD2	0	0	0	0	0	2.52329858183764 (0.0051707670377398)	0	0	0	0
PTCHD1	3.64945395322768 (5.15777868539876e-06)	0	0	0	0	4.43636068857104 (0.00142399911025085)	0	0	0	0
PTEN	0	0	-1.57272135583826 (0.000728865667677913)	0	0	0	0	0	0	0
PTGDS	-1.74437110240332 (0.00580452077819639)	0	2.1258323604035 (2.35005092908815e-06)	-2.76627936560554 (0.00141156302176795)	0	0	0	-2.84576360620333 (0.00450208040798991)	0	-2.50601768991952 (0.00259074567189571)
PTGER2	-2.24396463819566 (0.00213308351087687)	0	-4.28681887564816 (4.37766344235679e-06)	0	0	0	0	0	0	0
PTGER3	4.9268015220171 (5.39310197996942e-10)	0	0	0	4.26930978941214 (0.00244828271548049)	0	3.17038880325624 (0.00133337651607025)	0	2.79694125075105 (0.00825962391793439)	5.74836618586896 (3.63944613502306e-05)
PTGES	0	0	0	0	0	0	0	0	4.157414895234 (1.22574572311123e-13)	4.01019324036016 (0.000130784910599259)
PTGES2	0	0	1.92897616326632 (0.00217945224747373)	0	0	0	0	0	0	0
PTGES3	0	-5.66156068749875 (0.00775598532699839)	0	0	0	0	0	0	0	0
PTGES3L	4.92986883685807 (0.00120976394664301)	0	0	0	0	0	0	0	0	0
PTGFR	2.74149043712902 (7.34051210506361e-05)	0	0	0	0	0	0	0	0	0
PTGFRN	2.28384782238 (1.70367983840061e-09)	0	1.86742865189702 (2.19994501937061e-06)	2.18751925181036 (0.00305602650326028)	0	0	0	-4.49293527089363 (0.006455631084676)	0	0
PTGIR	0	0	0	0	0	0	0	0	-2.706706825683 (0.00142227463776725)	0
PTGIS	0	0	0	0	0	0	0	-3.96156664060405 (0.000144048636909076)	0	1.92481425919735 (0.00111036752780669)
PTGR1	0	0	0	0	0	3.35104991452384 (4.48338751593478e-11)	0	0	0	0
PTGS1	3.60263112525257 (2.38549143539227e-16)	0	0	3.21519757978224 (0.000257592033445787)	0	0	0	0	0	0
PTGS2	0	0	-5.86797898528687 (4.44884850773587e-11)	0	0	0	0	-5.37337949612051 (0.00252134188998743)	0	0
PTH1R	-3.08319347539482 (0.000281429927066178)	3.21448449391078 (0.0052313959196452)	0	0	0	0	0	0	0	0
PTH2R	0	0	0	0	0	0	0	0	3.7374983732503 (0.00722058529650809)	0
PTK2B	-1.61174760166584 (0.000521924356840182)	0	-2.56381189689028 (3.80620093660766e-07)	0	0	0	0	0	0	0
PTK7	-2.4718734411426 (0.00019433313084617)	0	-3.4414168179912 (1.84199479847792e-06)	-3.06125563971893 (0.00625356480857931)	0	0	0	-4.5898777440197 (0.00837563678500064)	0	0
PTMAP2	0	3.94766689731102 (0.00216790134074351)	0	0	0	0	0	0	0	0
PTMS	0	0	0	0	3.13432315827303 (2.98136719823409e-05)	2.94897329854717 (0.000409177307929527)	0	0	0	0
PTN	2.56258990169164 (1.98668010442991e-05)	0	2.98821695667082 (0.000259592380639071)	3.67978194072862 (0.00016973880000661)	0	0	0	0	2.18515455119389 (5.10478169262382e-06)	0
PTP4A1	0	-5.96497676947602 (0.00690247030314889)	-2.47724386238015 (5.48295789071296e-06)	0	0	0	0	-2.3206195465815 (0.00995353851642288)	0	0
PTP4A1P7	0	0	0	0	0	0	0	0	4.2249490971384 (0.00555218345737547)	0
PTP4A3	0	0	3.52160653828653 (4.80677011109215e-11)	0	0	0	0	0	-2.60981288722501 (0.00120390221612113)	0
PTPLA	0	0	2.37049290221195 (0.000444898867798065)	0	0	0	0	0	0	0
PTPLAD2	0	0	-2.3908725915073 (0.00188565471013366)	0	0	0	0	0	0	0
PTPN1	0	0	0	0	0	-1.71038868005261 (0.000889524980517036)	0	0	0	0
PTPN12	0	0	0	0	-1.74091556927278 (0.00226073009936831)	0	0	0	0	0
PTPN13	0	0	-2.09787504553768 (2.95865250146494e-05)	0	0	-2.0643807404268 (0.00339824295530826)	0	-4.31100076259961 (0.000627160531244323)	0	0
PTPN14	0	0	0	0	0	0	0	-5.4691208816347 (0.000571917158435135)	0	0
PTPN18	0	0	-1.65440208334307 (0.00505565817926033)	0	0	0	0	0	0	0
PTPN3	-1.83650155672583 (0.00173695762392211)	0	0	0	0	0	0	0	1.65188087000882 (5.69778663759087e-05)	0
PTPN5	0	0	4.51761792223137 (3.17420950591042e-05)	0	0	0	0	0	0	0
PTPN6	-2.11098037116937 (1.44920322523551e-05)	0	-2.70697311827797 (5.18768123044946e-06)	0	0	0	0	0	0	0
PTPRB	0	0	0	0	0	0	0	-4.594854107872 (0.000101080926674414)	0	0
PTPRC	-2.43181090995173 (0.000280327994720187)	0	-3.30290104643639 (3.64834628138034e-06)	-2.42047000609695 (0.00954006100108351)	-2.23516490571615 (0.00782928978384877)	0	0	0	-1.64008188616143 (0.000814632028921885)	0
PTPRF	0	0	-2.48416541930689 (9.7570813305783e-05)	0	0	0	0	-3.03220345751907 (0.00643727066028744)	0	0
PTPRG	0	0	0	0	0	0	0	-5.55450985233414 (2.66201125713606e-06)	0	0
PTPRH	0	0	0	0	0	6.36454908073801 (1.55846030335661e-07)	0	0	0	0
PTPRJ	0	0	0	0	0	0	0	0	0	-2.20137911772959 (0.00215785937485576)
PTPRK	0	0	0	0	0	0	0	-3.23170699631963 (0.00320761747804134)	0	0
PTPRM	0	0	0	0	0	0	0	-2.31759515615614 (0.00132687672668763)	0	0

PTPRN	0	0	0	0	0	5.19292866221907 (0.00219356327621867)	0	0	0	0
PTPRR	0	0	0	0	0	0	0	0	3.31660242466662 (0.00945495610548584)	0
PTPRS	0	0	0	0	0	-2.44954015053175 (0.000125470018231398)	0	-5.09572987711654 (3.06930971935281e-05)	-2.40371001032482 (2.51754640702941e-12)	0
PTPRT	0	0	0	0	0	3.7838128674933 (0.00313167649374008)	0	0	0	0
PTPRU	0	0	0	0	0	0	0	-5.88781533267213 (0.0094070682156658)	0	0
PTPRZ1	3.09913162725309 (0.000362623245894042)	0	0	0	0	0	0	0	0	0
PTRF	0	0	0	0	0	0	0	-2.88336912277893 (7.84763215839667e-05)	0	0
PTTG1	0	0	0	0	0	0	0	4.4897620328428 (0.000463940288531896)	0	0
PTX3	0	0	0	-2.42992356507927 (0.00561211730191451)	-2.28747040031549 (0.00390330259583823)	-4.6439198613191 (4.03880651254059e-07)	0	0	-2.60452151330812 (0.000404610904261405)	0
PURG	0	0	0	0	5.83005063945246 (0.00374446131635038)	0	0	0	0	0
PVR	0	0	0	0	0	0	0	-5.30954270559588 (0.00133388880847666)	0	0
PVRL3	0	0	0	0	0	1.91041026808382 (0.00906696677923237)	0	0	3.19480367912329 (1.99666351040307e-06)	0
PVRL4	-4.1717120445683 (0.00186845111755429)	0	-3.73953386464735 (0.00673312440184292)	0	0	0	0	0	3.25650108556691 (7.38618206111313e-07)	3.65488057215997 (0.00226094479912333)
PWAR1	4.22140424225757 (0.00324703861412312)	0	0	0	0	0	0	0	0	0
PWAR6	1.78453013681977 (0.00320412089171921)	0	0	0	0	0	0	0	0	0
PWRN1	0	0	0	0	0	0	0	0	4.74009748275112 (0.00416902351719798)	0
PWWP2B	-2.05353311130086 (0.00823617811155388)	0	0	0	0	0	0	0	0	0
PXDC1	0	0	0	0	0	0	0	-6.43318425627948 (0.00420515282407447)	0	0
PXDN	0	0	0	0	0	0	0	-2.60943472085972 (0.00113929776644069)	0	0
PXDNL	0	0	7.23176166071308 (1.04687770030852e-13)	0	0	0	0	0	0	0
PXMP2	0	0	2.72076717309985 (0.0039604387857078)	0	0	0	0	0	0	0
PXYLP1	0	0	1.96647765408602 (0.00634632865910841)	0	0	0	0	0	2.10814853109745 (2.1921475365957e-06)	0
PYGB	0	0	2.19515125123132 (2.97979647392427e-05)	0	0	0	0	0	0	0
PYGL	0	0	-2.11790168216125 (4.87492341960453e-05)	0	0	0	0	0	0	0
PYGM	2.93070624051754 (0.000509788684097079)	0	4.75759557964344 (8.78942690569685e-10)	0	0	0	0	0	0	0
PYGO1	1.70815914010119 (0.000416098099914695)	0	0	0	0	0	0	0	0	0
QDPR	0	0	0	0	0	1.67877898461213 (0.00679112975854449)	0	0	0	0
QPRT	0	0	0	0	0	0	0	0	2.19409892308695 (4.66212846397536e-05)	0
QRFP	0	0	0	0	0	6.52658772587952 (0.00462355117996762)	0	0	0	0
QSOX2	-1.71329799094284 (0.00496636745707927)	0	0	0	0	0	0	2.28678675504299 (0.00938886638449629)	-1.56244965900807 (0.0027640233390845)	0
R3HCC1	0	0	1.64398616106723 (0.00150384711469464)	0	0	0	0	0	0	0
RAB11FIP1	-1.55325245706128 (6.5553838597921e-05)	0	-2.81121723365384 (6.99674447417273e-10)	-2.02235297586709 (0.00588641258234493)	0	0	0	0	0	-2.30338129831591 (0.00176643036178246)
RAB11FIP2	0	0	0	0	0	-2.23643550817143 (0.000539291386422586)	0	0	0	0
RAB11FIP4	-2.8717773734846 (0.001714671013365)	0	0	0	0	0	0	0	0	0
RAB11FIP5	0	0	0	0	0	0	0	-5.92767146557535 (0.00726962450572231)	0	0
RAB12	0	0	0	0	0	0	0	-2.67016390926698 (0.00859230912817768)	0	0
RAB13	0	0	0	0	0	0	0	0	-2.26733293277084 (1.58157463348184e-10)	0
RAB15	0	0	2.39773816024012 (0.000238897903374023)	0	0	0	0	0	0	0
RAB17	0	0	0	0	2.94029230494217 (0.00464916560823324)	3.95348670160483 (2.36991840095152e-06)	0	0	0	0
RAB23	2.44337329620722 (3.4474421371405e-13)	0	0	2.85269977624221 (0.000125522356679256)	0	0	0	0	0	0
RAB25	-3.65493117893335 (0.00807820723776195)	0	0	0	0	0	0	0	0	0
RAB26	0	0	0	0	0	4.79219113477506 (0.0051644902949428)	0	0	0	0
RAB27A	0	0	-2.23170293480757 (9.64573356896614e-05)	-2.62228023468414 (0.00357563858769444)	0	0	0	0	-1.93050934747143 (1.97970437126084e-05)	0
RAB27B	-2.89779564259675 (7.43318232378163e-05)	0	-5.12794274564931 (3.75845528100953e-08)	-7.93404191598463 (0.000512109343344339)	-5.05538381614807 (0.000737737971069196)	-2.7878404175877 (0.00443122506513384)	0	0	0	0
RAB29	0	0	0	0	0	0	0	0	-1.52276572703381 (0.00490409990557149)	0
RAB31	0	0	-3.03943418622789 (2.68410856043987e-08)	0	0	0	0	0	0	0
RAB32	0	0	-2.52880141840976 (0.00510629783938236)	0	0	0	0	0	0	0
RAB37	0	0	0	0	0	2.89546387545988 (0.00824923768408371)	0	0	0	0
RAB39B	3.71128995284274 (0.00362196072836501)	0	0	0	0	0	0	0	0	0
RAB3B	4.89137637024469 (2.99647667584561e-09)	0	0	4.73125429927627 (0.0014887007719648)	5.13086434711092 (2.4375605577216e-06)	5.70082056459667 (5.54416221382333e-07)	0	0	5.75609743669549 (3.4211144825073e-06)	0
RAB3C	5.33051994080529 (2.37128415893607e-07)	0	3.48547584262593 (0.00350483033638363)	0	5.42651542417164 (0.000108861862304096)	4.06099501251939 (0.00822465526118334)	0	0	0	0
RAB3D	0	0	0	0	0	0	0	0	-1.76017252237978 (1.35830555821058e-05)	0
RAB40B	0	0	2.12434023065179 (0.0028266630949918)	0	0	0	0	0	0	0
RAB44	0	0	0	0	0	0	0	4.68200652619539 (0.000252728943549491)	0	0
RAB6B	2.88997563391899 (1.94895464493881e-05)	0	3.41263103853799 (2.07185742394081e-07)	3.39211876417255 (0.00329431934647398)	0	0	0	4.74472983733684 (3.63043629523708e-06)	0	0
RAB8B	0	0	-1.77323759799182 (0.00156604440390812)	0	0	0	0	0	0	0
RAB9B	3.41935636185471 (0.000145340431212396)	0	3.1056859590645 (0.00187232413819495)	0	0	0	0	0	0	0
RABGAP1L	0	0	0	0	0	0	0	2.01379948561229 (0.00125240135137431)	0	0
RABIF	0	0	0	0	0	0	0	0	1.78702360800376 (0.0075546048380358)	0
RAC2	0	0	0	0	0	0	0	3.22481676316269 (0.000699672496548857)	-1.72716151245787 (0.00561457836150268)	0
RACGAP1	0	0	0	0	0	0	0	2.66416644668254 (0.00326564127246536)	0	0
RAD17P1	0	0	0	0	0	0	4.6014218294231 (0.00101345059116453)	0	0	0
RAD51AP1	0	0	0	0	0	0	0	3.61100797481748 (0.00470245864317997)	0	0
RAD54L	0	0	0	0	0	0	0	4.5193019128531 (0.00831192309200048)	0	0
RAI1	0	0	0	0	0	0	0	0	-2.44901008883232 (3.36771829056899e-07)	0
RAI14	0	0	-1.77827915517887 (7.54807573544536e-05)	0	0	0	0	-3.47439318663802 (1.02445599361199e-05)	0	0
RALA	0	0	-1.99134977088906 (1.42677942936139e-05)	0	0	0	0	0	0	0
RALGPS2	2.83316222074347 (9.15231324124487e-06)	0	0	0	0	0	0	2.97698577327377 (0.00267293075500877)	1.88027911546122 (2.16072788884767e-05)	0
RAMP1	2.89902940418598 (2.39052422095748e-08)	0	3.50698800069505 (2.75607662250689e-05)	3.50757567871444 (0.00118797680832502)	0	0	0	0	0	0
RAMP2	0	0	0	0	0	0	1.54125578431798 (4.18578720170461e-05)	0	0	0
RAMP3	0	4.35196165037862 (2.1653537502812e-05)	2.17840941840074 (0.000155550129682683)	0	0	0	2.37351700918057 (2.84635104058206e-06)	0	-2.18477617817268 (0.00385634235375113)	0
RANBP10	0	0	0	0	0	0	0	3.36763990016999 (0.000250532674935416)	0	0
RANBP20P	0	0	0	0	0	7.00881329009256 (0.00886510847483269)	0	0	0	0
RAP1B	0	0	-1.58022907481898 (0.000285326321351601)	0	0	0	0	0	0	0
RAP1GAP	-2.53765729412668 (0.00961852335320212)	0	0	0	3.05518349848339 (0.000608789498725196)	3.15162310987122 (7.36542865100139e-05)	0	3.66085077798454 (0.000187948422758803)	0	0
RAP1GAP2	0	0	1.75044280651845	0	0	0	0	0	0	0

			(0.00356258668840162)							
RAP2B	-1.79369723447301 (0.000273800826795732)	0	-1.62266390219844 (0.000863603802160525)	0	0	0	0	0	0	0
RAPGEF3	0	0	2.19199233266398 (4.03183491914506e-07)	0	2.95831433766689 (4.50754440550284e-05)	0	0	0	0	0
RAPGEF4	0	0	0	0	0	0	0	0	-3.26466414291549 (9.04002027641736e-09)	0
RAPGEF5	-2.77979180482465 (2.88837654347488e-11)	0	0	-2.34135923381765 (0.00116826942796654)	0	0	0	-3.60333682519658 (0.000116197336496848)	0	0
RAPH1	0	0	0	0	-2.22732562484485 (0.0036919147660364)	0	0	-4.79030007940849 (0.00298419509614007)	0	0
RARRES1	0	0	0	0	0	0	0	-6.44382959259787 (0.00345875155487823)	-4.65216420151723 (5.01095532228538e-14)	0
RARRES2	0	0	-2.34127743173822 (0.000773779839718685)	0	0	0	0	0	-3.04452266503574 (2.62007002347578e-08)	0
RASA1	0	0	0	0	0	0	0	0	3.04996553491685 (7.55976942372075e-13)	0
RASA4CP	0	0	0	0	5.0602426192642 (0.00646359408462247)	0	0	0	0	0
RASAL1	0	0	0	0	6.97215043106458 (0.000924729043531403)	0	0	0	0	0
RASAL2	0	0	0	0	-1.60780836031012 (0.00692433586200429)	0	0	-3.67402589586113 (0.000595143423506466)	0	0
RASAL2-AS1	0	0	0	0	4.3900991012393 (0.000596471813091471)	5.41799702737979 (0.000693997919522657)	0	0	0	0
RASAL3	-2.52071500792524 (0.00364901694033424)	0	-3.11442158927294 (0.000667307746972978)	0	0	0	0	0	-3.5956842737555 (2.88587780509107e-06)	0
RASD1	0	0	0	0	0	3.0749430580427 (0.00223157057369454)	0	0	-3.00366003310525 (0.00227406343290711)	0
RASD2	3.13604010746134 (7.14143156341019e-05)	0	2.45179288702878 (0.00693563776642154)	0	0	4.48791103566211 (0.0030616170943675)	0	0	0	0
RASEF	-2.16287398790292 (0.00198122368684579)	0	-4.80852124859922 (3.54977101071755e-06)	0	0	0	0	0	0	0
RASGEF1B	-2.91055431443573 (1.04113459357715e-06)	0	-4.80447051192592 (4.36002180470298e-10)	-2.64207560523914 (0.0019910107311555)	-2.35989296417669 (0.00224559570411826)	0	0	-3.5833297240481 (0.000524975731204716)	0	0
RASGEF1C	0	0	0	0	0	6.07562527319622 (0.00800215069786628)	0	0	0	0
RASGRF1	-5.08579069351826 (0.000171587870010682)	0	-4.08252002932819 (0.00165443964403186)	0	0	0	0	0	0	0
RASGRF2	0	0	0	0	0	0	0	0	2.18376749225588 (5.47518776180044e-06)	0
RASGRP1	0	0	-2.9048925074666 (0.00951741024513222)	0	0	0	0	0	0	0
RASGRP3	0	0	2.26183343257879 (0.000142463230340868)	0	0	0	0	0	0	0
RASGRP4	-3.08518967975891 (0.000240020476274752)	0	0	0	0	0	0	0	0	0
RASIP1	0	0	0	0	0	0	0	0	0	-6.6601720339154 (0.00355623076202464)
RASL10B	0	0	4.0283018353291 (2.92437299193864e-08)	0	0	0	0	0	0	0
RASL11A	2.51207719554343 (0.00107894870689264)	0	0	0	0	0	0	0	0	0
RASL11B	0	0	0	0	0	0	0	0	3.29888056485782 (0.000274381742364231)	0
RASL12	0	0	0	0	0	0	0	-5.83619039832998 (0.0097618421974046)	0	0
RASSF2	0	0	-2.7381714310093 (9.9628488127527e-06)	0	0	0	0	0	0	0
RASSF5	-2.0261692172807 (0.000228020574453938)	0	0	-2.8398770755706 (0.0055103097543451)	0	0	0	0	0	0
RASSF6	0	0	0	0	0	0	0	0	2.91050739711423 (0.00046738095891308)	0
RASSF7	-4.43258428701244 (0.000502399768255196)	0	0	0	0	0	0	0	0	0
RASSF8	0	0	0	0	0	-1.50759730475478 (0.00178913878064288)	0	-7.90698896783937 (0.000222033887045786)	0	0
RBBP6	0	0	0	0	0	0	0	0	1.54148009144095 (1.02085577939931e-06)	0
RBBP8	-1.62037510447734 (0.000165745633109963)	0	-1.86831093987949 (0.000487327805146874)	0	0	0	0	0	0	0
RBFOX1	0	0	7.98438342571707 (1.53946775176656e-13)	0	6.82403350424883 (0.000166287887147743)	0	0	0	0	0
RBFOX2	0	0	0	0	0	0	0	-4.26641077640937 (2.81482998017067e-05)	0	0
RBFOX3	7.00252830345318 (4.96442224444125e-10)	0	0	7.80794534546782 (4.05149093657379e-07)	0	6.78050585430463 (0.000452050479494713)	5.60183672275245 (0.00260274517522135)	0	0	0
RBL1	0	0	-1.61763108991127 (0.0091415179373714)	0	0	0	0	2.30744605206418 (0.00342211210172008)	0	0
RBM12	0	0	0	0	-1.53153192991845 (0.00731099895736855)	0	0	0	0	0
RBM15	0	0	0	0	0	0	0	1.97633714752376 (0.00859768197238858)	0	0
RBM17	0	0	0	0	0	-1.60275474334118 (0.000693636104053149)	0	0	0	0
RBM20	3.19193353940105 (4.78014980102179e-05)	0	7.24821999647858 (1.41084551462776e-21)	5.22490922588943 (4.63521910005576e-05)	0	0	0	0	2.21431690997001 (0.00569281402865453)	0
RBM22P2	0	0	0	0	0	0	5.00542294455249 (0.0019535050172033)	0	0	0
RBM24	3.97403722415014 (2.54295151619997e-08)	0	5.7197570891501 (2.59602719045247e-15)	4.11810703536048 (0.00311230338312507)	0	0	0	0	0	0
RBM3	-1.87192853093786 (0.00519779359885375)	0	-1.72025127148076 (0.00307230670617377)	-3.61568882694564 (0.00015984028521623)	0	-1.82602880721719 (0.00416352655342938)	0	0	0	0
RBM38	0	0	2.26348765670856 (0.000611672349908042)	0	0	0	0	3.27073577742301 (0.00128643136430096)	-1.78637833549056 (0.00483813260003849)	0
RBM47	-2.40961746541596 (4.69616604521998e-07)	0	-3.37421130355384 (4.86085690144541e-08)	-2.93371167745678 (0.000459530865493241)	0	0	0	0	0	0
RBMS1	0	0	0	0	0	-1.52217573026555 (0.00389834482578748)	0	-1.78358222228908 (0.00631569456855292)	0	0
RBMS2	0	0	0	0	0	-2.02336261770364 (8.21050414396943e-05)	0	-3.21591576885442 (3.38956410668852e-05)	0	0
RBMS3	0	0	0	0	0	0	0	-3.93480473262677 (0.000254218436731775)	0	0
RBP1	0	0	0	0	0	3.46656386656859 (7.14321537608039e-08)	0	0	3.50920149118214 (9.10622958228293e-05)	0
RBP2	4.85130135831129 (0.00588619525605112)	0	0	0	0	0	0	0	0	0
RBP3	0	0	0	0	0	7.74911394168542 (1.73683686560124e-05)	0	0	0	0
RBP4	0	0	0	0	0	9.96392982918103 (5.38602569543458e-35)	0	0	0	0
RBP5	0	0	0	0	0	0	0	0	-2.68379046624033 (0.00815370519982827)	0
RBP7	0	0	3.76778197605025 (1.42870886466617e-06)	0	0	0	0	0	0	5.65717831336535 (1.47903561518127e-07)
RBPJ	0	0	0	0	0	-1.68184635900427 (0.00170274995537286)	0	0	0	0
RBPMS	1.87755844326406 (4.78171645823827e-11)	0	0	0	0	0	0	-2.89786100825394 (0.000746706933062455)	0	0
RBPMS2	4.29094941497147 (3.06558838537705e-18)	0	4.87532254842991 (8.37862877258279e-16)	5.13382266726799 (7.50942626701624e-10)	0	0	0	0	0	0
RCAN2	0	0	2.02048428963446 (5.95950965491117e-06)	0	0	0	0	0	-5.05535927509114 (4.37778905963731e-12)	0
RCAN3	0	0	-1.5416024708205 (0.00262301695728174)	0	0	0	0	0	0	0
RCCD1	0	0	0	0	0	0	0	3.58809666446949 (0.000354431955973023)	0	0
RCE1	-3.04047381105021 (0.00997462182083899)	0	0	0	0	0	0	0	0	0
RCL1	0	0	0	0	0	0	0	2.78446644140432 (0.00697074382933522)	0	0
RCN1	0	0	0	0	0	0	0	-3.0684332137231 (0.000913523040185203)	0	0
RCOR1	0	0	0	0	-2.13399929258497 (0.00617260112169917)	0	0	0	0	0
RD3	0	0	0	0	0	8.08791186690628 (0.00117930857036654)	0	0	0	0
RDH10	0	0	0	0	0	0	0	0	1.98267922508581 (0.000261545145787619)	0
RDH13	0	0	0	0	0	0	0	0	2.69466753424662 (1.50829168559002e-05)	0
RDH16	0	0	0	0	0	6.92184962972259 (1.46656007034255e-10)	0	0	0	0
RECK	0	0	0	0	0	0	0	-6.24401526151946 (0.00454316642394932)	0	0
REEP1	5.52496066949498 (1.76279156307679e-12)	0	0	5.07097404041603 (0.00251575433307272)	0	0	0	0	0	0
REEP2	5.59733922626693 (1.2961109769311e-06)	0	5.0563039362676 (0.00155071659287534)	0	0	0	0	0	0	0
REEP3	0	0	0	0	0	0	0	-3.30285676196789 (0.000600712655495074)	0	0

REEP6	0	0	0	0	0	5.07991830423616 (5.44502233311921e-10)	0	0	0	0
REG1A	6.95378449188564 (7.78388815216897e-09)	0	0	7.60687411822306 (4.3204660368986e-05)	0	0	0	0	0	0
REG3A	7.46200934579952 (1.65860373129379e-05)	0	0	10.2407569874013 (4.87222190307491e-16)	0	0	0	0	0	0
REG4	4.76829740771433 (0.00102314697080627)	0	0	0	0	0	0	0	0	0
REL	-1.69200310451148 (0.000227529718644779)	0	-2.24245501443002 (5.92835606594901e-07)	0	0	0	0	0	0	0
RELL2	0	0	0	0	0	0	0	0	2.9901893419988 (0.00251296010543939)	0
RELN	1.7375144147174 (0.00200292831016195)	0	0	0	3.02399248969719 (9.07672068655821e-05)	3.63427353057442 (4.46662416737016e-08)	0	0	0	0
RELT	-2.21497709386423 (0.000110590464898004)	0	-1.93406898757731 (0.00566205994774717)	0	0	0	0	0	-1.70500483107838 (0.000487704072816288)	0
REN	0	0	0	0	0	0	0	0	4.72698332873383 (0.00272775812476354)	0
RENBP	0	0	0	0	0	0	0	0	-2.20909264908834 (0.000608254454766838)	0
RERG	2.68817375724503 (1.76659803845879e-12)	0	0	2.58125382347523 (0.00121540006121012)	0	0	0	0	0	0
RERGL	3.8345810680243 (0.000317555176177772)	0	0	0	0	0	0	0	0	0
RET	3.25391772151502 (0.000738836472160243)	0	0	0	0	5.00925520963544 (0.00588720327434281)	0	0	0	0
RETN	0	0	0	0	0	0	0	7.08951669882635 (1.31947241545306e-08)	0	0
REV3L	0	0	0	0	0	0	0	0	0	1.96047730241112 (0.000348186713519245)
REXO1L1P	0	0	0	0	7.30324281679443 (0.000324803134616262)	8.26517954695529 (9.69084145098489e-05)	0	0	0	0
RFC4	0	0	0	0	0	0	0	3.30224496348295 (0.00346676458106869)	0	0
RFESD	0	0	0	0	0	0	4.22459147155191 (0.00459729011639236)	0	3.86879240228122 (0.00580963917437324)	0
RFK	0	0	0	0	0	0	0	0	1.65372154299963 (0.00106043705141603)	0
RFTN2	0	0	0	0	0	0	1.76024463472152 (0.00704871253424681)	0	0	0
RFWD2P1	0	0	0	0	0	7.3837434313229 (0.00493066199114422)	0	0	0	0
RFX2	0	3.43870066200628 (0.00247510149385038)	0	0	0	0	0	0	0	0
RFX8	0	6.9291899034286 (6.02949368241346e-06)	0	0	0	0	0	0	0	0
RGCC	-4.2264961520864 (2.90289509313562e-13)	0	-2.23009923540651 (9.65767401221687e-05)	-3.72952988698829 (9.15013607931835e-05)	-2.90689705279261 (0.000673041080377709)	-5.05916841140133 (7.50895822046175e-07)	0	-3.99083219808532 (0.000349455267769582)	0	0
RGL3	-3.03182101573548 (7.95261806114285e-07)	0	0	-3.28178402571216 (0.00488294366322593)	0	0	0	0	0	-3.1482743580718 (0.0038089641591758)
RGL4	0	0	0	0	0	0	0	5.5674616593467 (1.799305260735e-06)	0	0
RGMA	7.11933196179834 (1.64309866285645e-36)	0	3.77684764687599 (4.35285389877299e-07)	6.94928125593429 (4.77825573616759e-14)	0	5.09021590668272 (0.000854786960238334)	3.24487050387568 (0.00144745527965077)	0	0	0
RGMB-AS1	0	0	0	0	0	6.64701620369491 (0.00376485311721762)	0	0	0	0
RGN	0	4.41321712380268 (7.24379553538562e-05)	0	0	0	2.63223322601332 (0.00242915100292643)	0	0	0	0
RGPD1	0	0	0	0	0	0	0	0	4.49771247239385 (0.000725694959454345)	0
RGPD3	0	0	0	0	0	5.33805240158333 (0.00679682395495329)	0	0	0	0
RGPD8	0	4.76722450150273 (0.00152752943315572)	0	0	0	0	0	0	0	0
RGS1	0	0	-5.12489517552446 (2.1886244215904e-11)	0	0	0	0	0	0	0
RGS16	-1.95683376542916 (0.00646385556565135)	0	0	0	0	0	0	0	0	0
RGS18	-3.69905894235907 (0.00146960095902791)	0	0	0	0	0	0	0	0	0
RGS19	-1.72341232585798 (0.00843080318985394)	0	0	0	0	0	0	0	0	0
RGS2	0	0	-2.27729434823113 (0.00134169174732596)	0	0	0	0	0	0	0
RGS3	0	0	0	0	0	0	0	0	-1.69082812846962 (0.000207741965375808)	0
RGS4	4.3081481768615 (0.00153654732340359)	0	0	0	0	0	0	0	0	0
RGS5	3.20305470667073 (7.87422877058299e-08)	0	0	2.88692674421827 (0.0025313431794351)	0	0	0	-2.95922300773849 (0.00789049953583321)	0	0
RGS6	0	0	3.50273406053646 (1.60747597358318e-07)	0	0	0	0	0	0	0
RGS7BP	0	0	0	0	0	4.19671578332223 (0.00656992390268912)	0	0	0	0
RGS8	0	0	0	0	0	7.02540147424054 (0.00154659719608631)	0	0	0	0
RHAG	0	0	0	0	0	0	0	8.5229729474853 (2.64245026968546e-09)	0	0
RHBDF2	-1.59104160236702 (0.00748138530884508)	0	0	0	0	0	0	0	0	0
RHBDL2	0	0	0	0	5.48120870307519 (0.00168039895365681)	0	2.43496876172461 (0.00967042502343419)	0	0	0
RHBDL3	0	0	0	0	0	6.69160203613501 (0.000319371414143143)	0	0	0	0
RHD	0	0	0	0	0	0	0	3.87512294178617 (0.00329871276087893)	0	0
RHOB	0	0	0	0	0	0	0	-2.45143349273669 (0.00254546534791479)	0	0
RHOBTB1	0	0	0	0	0	0	0	0	3.24106967971388 (1.35367006713485e-05)	0
RHOBTB2	-2.25454171315469 (0.000100501155086446)	0	0	0	0	-2.08383000314515 (0.00588728239014041)	0	-3.0523070726852 (0.00438331387519804)	-1.5981637165114 (0.000148711375736147)	-2.90549573763541 (0.00164069087040368)
RHOBTB3	0	0	0	0	0	0	0	0	1.89816197148897 (2.13922600838098e-05)	0
RHOD	0	0	3.21639239960934 (0.000172488390528947)	0	0	0	0	0	0	0
RHOH	0	0	0	0	0	0	0	0	-2.641759299082 (0.00222981569014563)	0
RHOQ	0	0	0	0	-2.33625888297239 (0.00775743489112398)	0	0	0	0	0
RHOT1	0	0	0	0	-2.48985633768541 (0.00923652677604157)	0	0	0	0	0
RHOU	0	0	0	0	0	0	0	0	2.25195725955118 (7.92558286020166e-07)	0
RHOV	0	0	0	0	0	0	0	0	0	6.09025932543437 (3.60696498594729e-05)
RHPN1	0	0	0	0	0	0	0	0	-3.37634404503033 (0.000460591389455846)	0
RHPN2	0	0	0	0	0	2.63945790731333 (0.00642633281061293)	0	0	2.33974960255197 (0.000687824802389784)	0
RIC3	2.07788271975553 (0.000509898936206001)	0	0	0	0	0	0	0	0	0
RILP	0	0	2.7511883308709 (7.87143562451947e-05)	0	0	2.59514322587631 (0.00538957164560898)	0	0	0	0
RILPL1	0	3.18892698345536 (0.00387462165199013)	2.89458782949459 (6.87545422205485e-08)	0	0	0	0	0	0	0
RILPL2	0	0	0	0	0	0	0	0	-2.1288209722984 (3.27316404550595e-07)	0
RIMBP2	5.91333002881783 (3.13177337511944e-06)	0	0	0	6.65055932551467 (0.00282029564248256)	7.11377161682505 (1.06538742321821e-05)	5.6383572926069 (0.00962187293686001)	0	0	0
RIMKLB	0	0	0	0	0	-1.55377319137229 (0.0076058455675787)	0	0	0	0
RIMS1	4.05680657727401 (3.69626535625051e-07)	0	0	0	0	4.34867586689237 (0.00213038415895722)	2.80313980712437 (0.0096901378322475)	0	0	0
RIMS2	0	0	0	0	5.58521650767741 (0.00956961717020768)	6.17915898918056 (0.00151489551309516)	5.40621745640426 (0.00797005551679186)	0	0	0
RIN1	0	0	0	0	2.6362056975184 (0.00842210525448621)	0	0	0	0	0
RIN2	0	0	0	0	0	0	0	-7.57641687371706 (0.000396291064238299)	0	0
RINL	0	0	0	0	2.39791386211827 (0.00936453118604356)	0	0	0	0	0
RIOK1	0	2.45151591979194 (0.0090928527261931)	0	0	0	0	0	0	0	0
RIPK3	0	5.0944017739698 (6.44163582175392e-06)	0	0	0	0	0	0	0	0
RIPK4	0	4.7329785863148 (0.00124496774213793)	0	0	3.1088722752206 (0.00289339385979861)	0	0	0	0	0
RIPPLY1	0	0	0	0	0	5.38646108530797 (0.00864292530282804)	0	0	0	0
RLIM	0	0	-1.60346486971455	0	-1.95114460117986	-1.72737408725624	0	0	0	0

			(1.08843212191182e-05)		(0.000460574883533859)	(0.00020513729518794)				
RLTPR	0	0	0	0	0	4.67956913366586 (0.00850853365426161)	0	0	0	0
RMND1	0	3.89664858145567 (0.00629061671847374)	0	0	0	0	0	0	0	0
RMND5A	0	0	1.56282559081765 (0.000345625700806823)	0	0	0	0	0	0	0
RMRP	0	0	0	0	0	2.12362692445114 (0.00399954549187259)	0	0	0	0
RN7SKP237	0	0	0	0	0	6.45958379100721 (0.000270694214239416)	0	0	0	0
RN7SKP243	0	0	0	0	0	6.82028757289725 (0.00346100162704843)	0	0	0	0
RN7SKP36	0	7.66282183688538 (0.000227147361998072)	0	0	0	0	0	0	0	0
RN7SKP79	0	7.10013128763217 (6.90455663225472e-05)	0	0	0	0	0	0	0	0
RN7SKP80	0	5.00154340581535 (0.0001092765069298)	0	0	0	0	0	0	0	0
RN7SL105P	0	0	0	0	0	3.50041749172303 (0.00362284186076915)	0	0	0	0
RN7SL107P	0	0	0	0	0	5.56082193616422 (4.70074468344533e-06)	0	0	0	0
RN7SL118P	0	0	5.00077019797595 (0.000164613281494914)	0	6.10302286804462 (1.51054431658675e-05)	6.38295563347884 (5.05537827669931e-06)	0	0	0	0
RN7SL128P	0	3.48307487670244 (0.00786443114254056)	0	0	0	3.80425736670199 (5.13460876437427e-05)	0	0	0	0
RN7SL130P	0	0	0	0	0	6.31180250964108 (2.65414305390216e-05)	0	0	0	0
RN7SL144P	0	0	0	0	0	4.91569600636144 (0.00372506253161919)	0	0	0	0
RN7SL15P	0	0	0	0	3.82352963052748 (0.00970103459363597)	3.6558157506659 (0.00284378265087337)	0	0	0	0
RN7SL162P	0	0	0	0	0	5.39237492238461 (0.00726574430535586)	0	0	0	0
RN7SL183P	0	0	0	0	0	4.90866334884256 (0.00502700779306593)	0	0	0	0
RN7SL2	0	0	0	0	0	1.64054807061885 (0.000545083638093529)	0	0	0	0
RN7SL218P	0	0	0	0	0	4.11884291557061 (0.00916796400362062)	0	0	0	0
RN7SL230P	0	0	0	0	5.24110935397631 (0.00131937976883357)	5.69045241389475 (0.000197071717173335)	0	0	0	0
RN7SL242P	0	0	4.68722434994535 (0.0011059903323829)	0	0	6.53424741919944 (1.92044076645974e-07)	0	0	0	0
RN7SL263P	0	7.05769924138817 (0.000478093530014872)	4.41983112639201 (0.00386411977410615)	0	0	0	0	0	0	0
RN7SL277P	0	0	0	0	0	4.18514309054366 (6.52613928637855e-05)	0	0	0	0
RN7SL336P	0	0	0	0	5.7800642702998 (0.00591384444719865)	0	0	0	0	0
RN7SL33P	0	0	0	0	5.88428508076867 (0.0058024316167402)	8.1699330749132 (1.29273902297374e-08)	0	0	0	0
RN7SL381P	0	0	2.65364867806962 (0.00624962047178155)	0	0	3.98275228958642 (2.13087168095458e-05)	0	0	0	0
RN7SL38P	0	0	3.02365840445451 (0.00285635607059039)	0	0	0	0	0	0	0
RN7SL396P	0	2.98617963715572 (0.00857564396609726)	2.04221625587297 (0.00264015200635606)	0	0	4.25209007454515 (4.39744695326596e-07)	0	0	0	0
RN7SL413P	0	0	0	0	0	8.05714550103112 (0.00205648469268156)	0	0	0	0
RN7SL426P	0	0	3.11813464638757 (0.000911703255889892)	0	3.75742373488181 (0.00200517920052813)	5.35996048043312 (1.45481655118321e-08)	0	0	0	0
RN7SL452P	0	0	0	0	0	5.91654026677052 (0.000330217926897123)	0	0	0	0
RN7SL472P	0	0	0	0	0	7.17825716520714 (1.99183227690211e-05)	0	0	0	0
RN7SL479P	0	0	4.50263396187495 (0.0081159846739177)	0	0	7.47128579733243 (2.48201827225776e-07)	0	0	0	0
RN7SL481P	0	0	0	0	0	5.78417031375132 (8.47859270293638e-05)	0	0	0	0
RN7SL494P	0	0	0	0	0	5.28939304628412 (0.00034634083563896)	0	0	0	0
RN7SL4P	0	0	0	0	0	3.36634617895173 (4.18209934805795e-07)	0	0	0	0
RN7SL513P	0	0	0	0	0	5.30365843678441 (7.66169050513591e-05)	0	0	0	0
RN7SL555P	0	0	0	0	0	4.72899785449764 (0.000597106690859274)	0	0	0	0
RN7SL564P	0	0	0	0	0	5.95300237734648 (0.000352511290443202)	0	0	0	0
RN7SL5P	0	4.05110785769864 (6.41709054248491e-06)	0	0	0	1.67648799133164 (0.00551552589705297)	0	0	0	0
RN7SL630P	4.31747334349818 (0.00266708815929709)	0	4.83707185335995 (0.000760339983067379)	0	0	7.57001540355153 (6.95535456149117e-08)	3.1840333271146 (0.00923611757796907)	0	0	0
RN7SL665P	0	0	0	0	0	5.20727261086452 (2.17366213237833e-05)	0	0	0	0
RN7SL674P	0	0	0	0	0	2.41988173542327 (0.000792802799429231)	0	0	0	0
RN7SL677P	0	0	0	0	0	5.71221351486765 (0.00552402185830574)	0	0	0	0
RN7SL709P	0	0	0	0	0	5.15311426200766 (0.00384563724380764)	0	0	0	0
RN7SL716P	0	0	0	0	7.20697900913001 (0.00515462419084721)	0	0	0	0	0
RN7SL752P	0	4.20967904838355 (0.000517239792540822)	0	0	0	4.89821512322417 (8.21139468013145e-08)	0	0	0	0
RN7SL753P	0	0	5.59277597864743 (0.00019582529333314)	0	5.43725505292964 (0.00878816523377565)	6.25798278238416 (0.000121911964246922)	0	0	0	0
RN7SL767P	0	0	0	0	0	3.79474365764102 (0.000110676488546464)	0	0	0	0
RN7SL7P	0	0	3.1757662550524 (0.00297470161015572)	0	0	5.83955782191168 (2.47386000309527e-08)	0	0	0	0
RN7SL810P	0	0	0	0	0	4.44433646414652 (0.00267835096851383)	0	0	0	0
RN7SL838P	0	0	3.61220889440175 (0.00873808189216047)	0	0	7.35225433792501 (6.04623010361155e-10)	0	0	0	0
RN7SL8P	0	0	4.11973834941224 (0.000519803885818276)	0	4.28431797527577 (0.0017505778591716)	7.37024542939334 (1.42877102943016e-09)	0	0	0	0
RNA5-8S5	0	0	3.01445344127581 (0.000550371339018299)	0	0	5.40743596639874 (7.65715299898741e-10)	1.614846383218 (0.00110354473665288)	0	0	0
RNA5-8SP2	0	0	3.21871324834624 (0.00242931111883898)	0	0	5.25348724347386 (1.07896416244006e-05)	0	0	0	0
RNA5-8SP5	0	0	0	0	0	4.01340006877355 (0.00221177810218057)	0	0	0	0
RNA5-8SP6	0	0	0	0	0	2.87211263599567 (0.00159781881098713)	0	0	0	0
RNA5S9	0	0	0	0	0	4.17909925561812 (1.15344035988473e-06)	0	0	0	-3.14887121431958 (0.00470117591605291)
RNA5SP114	0	0	3.94359214391181 (0.00879505735921113)	0	0	5.38435617479203 (0.000703332400207162)	0	0	0	0
RNA5SP119	0	0	0	0	0	6.07841566912224 (2.50766283684407e-05)	0	0	0	0
RNA5SP122	0	0	0	0	0	4.93340941502654 (0.00024135071774961)	0	0	0	0
RNA5SP132	0	0	0	0	0	5.41741006297535 (5.01138109450402e-06)	0	0	0	0
RNA5SP145	0	0	0	0	0	0	0	0	0	-3.01123236412531 (0.00692912535042773)
RNA5SP149	0	0	0	0	0	4.13374539744558 (2.91636065930602e-07)	0	0	0	0
RNA5SP150	0	0	2.27938519987007 (0.00387352483609882)	0	2.63285096453546 (0.00499883496671054)	0	0	0	0	0
RNA5SP161	0	0	0	-3.85950598281108 (0.00719617987391088)	0	3.39165430968757 (0.00156464318784041)	0	0	0	-8.16689004649897 (0.000534584443974555)
RNA5SP19	0	0	0	0	0	4.48878342434895 (0.000122875455590222)	0	0	0	0
RNA5SP198	0	0	0	0	0	5.80578195336988 (0.000238596527092546)	0	0	0	0
RNA5SP199	0	0	0	-6.98173203296749 (0.00414453881987708)	0	0	0	0	0	0
RNA5SP202	0	0	0	0	0	4.82759515348318 (2.90320610387954e-07)	0	0	0	0
RNA5SP204	0	0	0	0	0	6.74496785463021 (0.00259720722340322)	0	0	0	0
RNA5SP207	0	0	0	0	0	3.6957879171039 (0.00835472790968853)	0	0	0	0
RNA5SP211	0	0	0	0	0	0	0	0	-2.08899611314641 (0.0093876345373656)	0
RNA5SP221	0	0	3.85939759299367 (0.00717566198658725)	0	0	6.00793346844516 (5.26353850084801e-05)	0	0	0	0

RNA5SP225	0	0	0	0	0	3.25860817149426 (0.0017112405306823)	0	0	-2.45748674495017 (0.0014366368605431)	-4.76860697718527 (0.0031408354028877)
RNA5SP242	0	0	0	-7.42927042228722 (0.00215853743919854)	0	4.55739296692897 (0.000102004659982905)	0	0	0	0
RNA5SP250	0	0	0	0	0	4.16415067787694 (0.000628374652376634)	0	0	0	0
RNA5SP252	0	0	0	0	0	5.33676280659263 (0.00683879922758768)	0	0	0	0
RNA5SP256	0	0	0	0	0	0	0	0	-3.37052562650726 (0.00789171055382876)	0
RNA5SP259	0	0	0	0	0	3.88402340442895 (0.000577031403356408)	0	0	0	0
RNA5SP260	0	0	0	0	0	3.57150184390972 (0.000338778448817445)	0	0	0	0
RNA5SP263	0	0	0	0	0	4.5861369378452 (0.00043260237240516)	0	0	0	0
RNA5SP267	0	0	3.43097161814783 (0.00823848668620107)	0	4.02115635091094 (0.000443887908280457)	5.62258140441011 (8.19361421808629e-08)	0	0	0	0
RNA5SP289	0	0	0	0	0	6.21866355776608 (0.00101293528996515)	0	0	0	0
RNA5SP290	0	0	0	0	0	5.21254280881534 (8.38702583080219e-05)	0	0	0	0
RNA5SP298	0	0	0	-4.57898443561686 (0.00238492453087845)	2.74529654180385 (0.000875084998953695)	0	0	0	0	0
RNA5SP324	0	0	0	0	0	3.91054728052274 (0.00511295349272622)	0	0	0	0
RNA5SP325	0	0	0	0	0	4.75399362718546 (5.02821275393004e-05)	0	0	0	0
RNA5SP330	0	0	0	0	7.21847437014238 (0.000749985002848081)	7.70023193681569 (0.000640481890605919)	0	0	0	0
RNA5SP336	0	0	0	0	3.78497542936741 (0.00480095424494416)	5.28089390302549 (4.37601317184186e-06)	0	0	0	0
RNA5SP338	0	0	0	0	0	3.69028391289586 (0.00138990182457478)	0	0	0	0
RNA5SP351	0	0	0	0	0	4.79367232789494 (1.08305318525597e-05)	0	0	0	0
RNA5SP353	0	0	0	0	0	5.0553456112016 (0.000955131724626531)	0	0	0	0
RNA5SP355	0	0	0	0	0	4.83235305471411 (2.59938227192776e-05)	0	0	0	0
RNA5SP358	0	0	0	0	0	4.49849206787421 (0.000954517266130454)	0	0	0	0
RNA5SP366	0	0	0	0	0	4.6768543263889 (7.08428675596125e-05)	0	0	0	0
RNA5SP370	0	0	0	0	0	3.97374528712364 (1.19064976138626e-05)	0	0	0	0
RNA5SP374	0	0	0	0	0	0	0	0	-3.26699147808533 (0.00960210047711828)	0
RNA5SP38	0	0	0	0	0	4.45239816095597 (0.000325780506363685)	0	0	0	0
RNA5SP382	0	0	0	0	0	5.14163860548385 (0.000205891837165024)	0	0	0	0
RNA5SP387	0	0	0	0	0	0	0	0	-2.85441025050082 (0.00257823523983014)	0
RNA5SP389	0	0	0	0	0	3.58133001208216 (6.1486912774709e-05)	0	0	0	0
RNA5SP395	0	0	0	0	0	6.26014107360838 (4.06620658634987e-05)	0	0	0	0
RNA5SP42	0	0	0	0	0	3.61318192108059 (0.00248113924292784)	0	0	0	0
RNA5SP425	0	0	0	0	4.6203065509548 (0.00554395359974401)	7.12511662294766 (4.73978722503233e-05)	0	0	0	0
RNA5SP429	0	0	0	0	0	3.72365564327895 (3.47278724888901e-06)	0	0	0	0
RNA5SP435	0	0	0	0	0	4.83194872130585 (8.24135094481393e-05)	0	0	0	0
RNA5SP442	0	0	0	0	0	4.58763742590041 (8.09259484126356e-06)	0	0	0	0
RNA5SP444	0	0	0	0	0	3.06561030586137 (0.0036117055192905)	0	0	-3.46853499157798 (0.000768409186906974)	0
RNA5SP449	0	0	0	0	0	5.4785838800678 (0.00173758191698863)	0	0	0	0
RNA5SP450	0	0	6.80570440495345 (5.69776378397769e-05)	0	6.4516949161868 (1.68604933109785e-06)	8.09406684696987 (4.14041352947023e-08)	0	0	0	0
RNA5SP452	0	0	0	0	0	5.7551821843009 (0.0011312674271542)	0	0	0	0
RNA5SP463	0	0	0	0	0	5.0732015814386 (0.00550906102322438)	0	0	0	0
RNA5SP473	0	0	0	0	0	3.65209692216029 (0.00119419461723892)	0	0	-3.29116359179968 (0.00524595013856872)	0
RNA5SP48	0	0	0	0	0	6.14099908644513 (1.48154853183902e-05)	0	0	0	0
RNA5SP481	0	0	0	0	0	4.17697532816613 (0.000557734441938128)	0	0	0	0
RNA5SP497	0	0	0	0	0	0	0	0	-3.93635492214692 (0.00182703811162956)	0
RNA5SP515	0	0	0	0	0	0	0	0	-1.86751424985567 (0.00869496360275835)	-6.74695240540881 (0.0042420613202434)
RNA5SP62	0	0	0	0	0	5.28596585407813 (0.000276607421085655)	0	0	0	0
RNA5SP71	0	0	0	0	0	4.49340826066586 (6.91282647543965e-05)	0	0	0	0
RNA5SP77	0	0	0	0	0	6.67105071990239 (0.0048576840122571)	0	0	0	0
RNA5SP85	0	0	0	0	0	4.61051970506748 (2.22085800855308e-05)	0	0	0	0
RNA5SP86	0	0	0	0	0	5.724237030565 (9.77678481716839e-05)	0	0	0	0
RNA5SP99	0	0	4.03605467483077 (0.00484353329009123)	0	4.41274993072767 (0.000156579257124081)	6.04359127664565 (3.40304624904265e-07)	0	0	0	0
RNASE1	0	0	0	0	0	-2.21238435070451 (0.00279361292151361)	0	-3.01654286339526 (0.00218528914778664)	0	0
RNASE2	0	0	0	0	0	0	0	4.76174399522316 (8.74832982133458e-05)	0	0
RNASET2	0	0	-2.70977293547679 (4.72725934272808e-06)	0	0	0	0	0	0	0
RND1	0	0	0	0	0	2.39336825268432 (0.00275483211479448)	0	0	-3.45418521965323 (0.000203937049719603)	0
RND2	0	0	0	0	0	4.82632359493675 (0.00221341187313596)	0	0	0	0
RND3	0	0	-1.80625202052794 (0.00117009778815835)	0	0	0	0	-8.80950057405596 (6.79005883452594e-05)	0	0
RNF115	0	0	1.71386925279738 (0.000669459074908214)	0	0	0	0	0	0	0
RNF122	0	0	1.61138789004327 (0.00501067350470288)	0	0	2.35788421107903 (0.00421918579568503)	0	0	0	0
RNF13	0	0	-1.83081102173364 (0.000541902920194413)	0	0	0	0	-2.63017399049421 (0.00746871053433875)	0	0
RNF135	0	2.92941310094021 (0.00890396127723303)	0	0	0	0	0	0	0	0
RNF144B	-2.97192794570143 (7.75823737582165e-11)	0	-1.5836228207037 (6.42843242082277e-05)	0	0	0	0	0	0	0
RNF145	0	0	-1.95573558677644 (2.41051734258435e-06)	0	-1.84434617178809 (0.00820619793678663)	-1.70957542707066 (0.00538883756095548)	0	0	0	0
RNF149	0	0	-2.01727238544716 (0.000580982994805744)	0	0	0	0	0	0	0
RNF150	4.95117329816201 (2.60089866727918e-24)	0	4.01850117799454 (6.53558014722982e-13)	5.28674059027429 (7.23036663654169e-10)	3.15858533669291 (0.00108013815763035)	3.48220205448896 (0.00547638120004191)	0	0	0	0
RNF152	-1.66468782289061 (0.00127095798879186)	0	0	-2.43945784291174 (0.00905042950785427)	0	0	0	0	0	0
RNF157	1.779373665669 (0.00478124901799288)	0	2.10255017798716 (0.00631555963528247)	0	0	3.23019370438037 (0.00145392904873564)	0	0	0	0
RNF165	0	0	0	0	3.50918635128744 (0.000790760817954289)	3.4452362322032 (0.00634670639346485)	0	0	0	0
RNF166	0	0	0	0	0	0	0	0	-1.98978259161198 (0.000287108953305638)	0
RNF17	0	0	0	0	0	0	0	0	4.42081183676217 (0.00637426274016138)	0
RNF170	0	0	0	0	0	0	0	0	1.70154952988147 (2.42409770439772e-05)	0
RNF183	0	0	0	0	0	6.62376832872746 (0.000134453013787966)	0	0	0	0
RNF207	0	0	2.22615684536661 (0.0023934993660156)	0	0	0	0	0	0	0
RNF208	0	0	3.98970304969607 (0.000447845588520725)	0	0	0	0	0	0	0
RNF213	0	-4.05014638092795 (0.00106272548482299)	0	0	0	0	0	0	0	0
RNF215	0	0	0	0	2.66303310830201 (0.00609452992769751)	2.76063413871575 (0.00583759617968008)	0	0	0	0
RNF219-AS1	0	0	0	0	0	4.68175880634273	0	0	0	0

						(0.00918416048708468)				
RNF6	0	0	0	0	0	-1.61916726415532 (0.00131191685294494)	0	0	0	0
RNU1-1	2.53591044406403 (0.00120731714059086)	0	0	0	0	0	2.12447512739766 (0.000766525027353792)	0	2.37678597306167 (0.00266737560811531)	0
RNU1-120P	0	0	0	0	0	5.25091369495173 (0.000306815644258017)	0	0	0	0
RNU1-134P	0	0	0	0	0	4.17366665572226 (0.000429451024018885)	0	0	-3.56210220359333 (0.0041035600998903)	0
RNU1-136P	0	0	5.11741191720497 (0.00177763317782585)	0	0	7.08772358916131 (1.30606219349066e-05)	3.67984088485856 (0.00851635497215346)	0	0	0
RNU1-138P	0	0	0	0	0	4.58173110184869 (0.00218119480917687)	0	0	0	0
RNU1-139P	0	0	0	0	0	2.89322679880076 (0.00941505999083809)	0	0	0	0
RNU1-13P	0	0	0	-6.37225752179895 (0.00970517262052716)	0	0	0	0	0	0
RNU1-146P	0	0	0	0	0	4.09057030710233 (0.00669284035985866)	0	0	0	0
RNU1-148P	0	0	0	0	0	2.91198762053059 (0.00442598701687459)	0	0	0	0
RNU1-16P	0	0	0	0	0	5.08646308409358 (0.000383294514688367)	0	0	0	0
RNU1-17P	0	0	0	0	0	5.25668303839483 (0.00287645154613282)	0	0	0	0
RNU1-18P	0	0	0	0	0	0	0	0	-4.51879794680663 (0.00342503553949663)	0
RNU1-20P	0	0	0	0	0	3.08419378946865 (0.00756343412330042)	0	0	0	0
RNU1-21P	0	0	0	0	0	3.23029630400959 (0.00197961116627794)	0	0	0	0
RNU1-27P	0	0	0	0	0	0	2.78500281493007 (0.000898745033815474)	0	2.95875542933572 (0.00127857162321837)	0
RNU1-28P	3.75593163176484 (4.68444262856988e-07)	0	2.79470481418994 (0.000610542399811498)	0	0	0	3.20530113087107 (5.310875339059e-07)	0	4.68654862047351 (1.90629687113701e-14)	0
RNU1-3	4.58205582528989 (0.000310218315325924)	0	4.62419713881099 (0.000511431660847065)	0	0	5.32185164734514 (0.00809718076507268)	4.48590330307727 (1.57797762928283e-05)	0	3.79741233351831 (0.00165401783448632)	0
RNU1-32P	0	0	3.34433361961908 (0.00248758144587226)	0	3.56116911785589 (0.00726962455443858)	4.9170532943053 (2.96720255077968e-05)	0	0	0	0
RNU1-4	0	0	0	0	0	0	0	0	2.41195418338922 (0.00428549291281121)	0
RNU1-65P	0	0	0	0	0	3.97364809145379 (0.00985952439452784)	0	0	0	0
RNU1-7P	0	0	0	0	0	4.49435534697782 (0.00085249511317002)	0	0	0	0
RNU1-83P	0	0	3.46217146164736 (0.00427604334196283)	0	0	4.51062126077214 (0.00136511471535739)	0	0	0	0
RNU1-89P	0	0	0	0	0	3.44883923422441 (0.000641879575308019)	0	0	0	0
RNU1-98P	0	0	4.30773490930393 (0.000191738420520755)	0	0	4.71132134061837 (0.000295862300792998)	3.21804753985478 (0.00159507267361848)	0	0	0
RNU11	0	0	2.04887329957216 (0.00294649442046219)	0	2.66609173036624 (0.00111879808163864)	3.76836978435974 (1.63344802077639e-06)	0	0	0	0
RNU4-58P	0	0	4.51293791342932 (0.0045387554378672)	0	0	0	0	0	0	0
RNU5A-1	0	0	0	-4.60640284004498 (3.83662605810005e-05)	0	0	0	0	0	0
RNU5A-8P	0	0	0	0	0	0	3.75318613575701 (0.00806892629601226)	0	0	0
RNU5B-1	0	0	0	-3.30434311161231 (0.00207054065060092)	0	0	0	0	0	-3.49654289270037 (0.00108993308256038)
RNU5B-2P	0	0	0	0	0	3.83967383766241 (0.00556861754324079)	0	0	0	0
RNU5D-2P	0	0	3.51057238029231 (0.00247346835575403)	0	0	3.74210546412834 (0.00128963054680237)	0	0	0	0
RNU5E-1	0	0	0	-5.42755268884482 (0.00307709819084135)	0	0	0	0	0	-3.70783954532859 (0.00566574977150189)
RNU6-1201P	0	0	0	0	0	5.37905236663763 (0.00252070580830627)	0	0	0	0
RNU6-2	0	0	0	0	0	5.54289502331955 (0.000298420002327179)	0	0	0	0
RNU6-702P	0	0	4.67435254638945 (0.00126500727541014)	0	0	0	0	0	0	0
RNU6-707P	0	0	4.03052913340245 (0.00128007369260632)	0	4.97432214513968 (0.000975494157584561)	5.55313578725462 (7.03176504051441e-07)	0	0	0	0
RNU6-761P	0	0	0	0	0	0	3.7588820901765 (0.00312994084909223)	0	0	0
RNU6-958P	0	0	0	0	0	3.19982221178643 (0.00872076975008063)	0	0	0	0
RNU6ATAC	0	0	2.38128707239361 (0.00676041878384796)	0	0	0	1.91713856887909 (0.00513721091158605)	0	0	0
RNVU1-10	0	0	2.89695743576303 (0.000615840734359097)	0	0	3.84534823064097 (0.000155879067714933)	2.17626575920463 (0.00231786099642203)	0	0	0
RNVU1-18	0	0	0	0	0	0	2.42339064307277 (0.000388182420215785)	0	2.54710183146307 (0.000553885852242477)	0
RNVU1-19	0	0	0	0	0	4.44235003084335 (0.000117538666875575)	0	0	0	0
RNVU1-7	1.7420411937823 (0.00722368992680463)	0	2.23795794818528 (0.00715101919947136)	0	0	0	2.25032194894991 (1.78550086580557e-05)	0	1.85205662609184 (0.000954893955665702)	0
ROBO1	0	0	0	0	0	0	0	-4.97559714360185 (0.00204270250312917)	0	0
ROBO2	-3.1149072412163 (0.000265916729396694)	0	-4.70885450390034 (1.02426823583138e-05)	0	0	0	0	0	0	0
ROBO3	0	0	0	0	0	0	0	0	-2.09204417423888 (0.00496494825346194)	0
ROCK1P1	0	0	0	0	5.17185791567869 (0.00082668642367621)	4.59117223867175 (0.00152685796092727)	0	0	0	0
ROGDI	0	0	2.7336456354881 (3.15576483694884e-07)	0	0	0	0	0	0	0
ROPN1	0	0	0	0	0	7.52336959946309 (0.00338497421599466)	0	0	0	0
ROR2	1.79021481291961 (0.00076587576973179)	0	-2.84307997196684 (0.00181509739696838)	0	0	0	0	0	0	0
RORB	0	0	0	0	0	0	0	0	4.74300893002933 (0.000937642202555522)	0
RORC	0	0	0	0	0	4.11161096598771 (4.95808476397461e-06)	0	0	0	0
ROS1	-8.89928985533789 (9.73704778078641e-15)	0	-6.79191778632232 (2.89209680659746e-14)	-8.73262122670469 (7.52198051275071e-05)	-3.55721975164253 (0.000792433608128371)	0	0	-7.67449390414829 (0.00052198579233324)	0	-9.01422231971546 (4.39283017633712e-05)
RP1	-5.28564755045743 (0.000335834013961294)	0	0	0	0	0	0	0	-2.76159102775106 (0.00764305232773292)	0
RP1-101G11.3	0	0	0	0	7.37412909408269 (0.000252528042796613)	8.32655519427756 (0.000149249241848511)	0	0	0	0
RP1-131F15.2	0	0	0	0	0	0	0	0	5.90534437884797 (9.24370415294623e-08)	0
RP1-140K8.5	-3.63074167356516 (0.00707966537844473)	0	0	0	0	0	0	0	0	0
RP1-149A16.19	0	0	0	0	0	0	0	0	-4.09118855236626 (0.00849455691579817)	0
RP1-152L7.5	0	0	0	0	0	0	0	0	3.39304618240608 (5.43255846834598e-05)	0
RP1-155D22.2	0	0	0	0	0	7.59958613495027 (0.00288964154949601)	0	0	0	0
RP1-239B22.5	0	0	0	0	0	3.01745563430086 (0.00639837583184816)	0	0	0	0
RP1-257A7.4	-3.04140117693194 (0.00308381763904551)	0	0	0	0	0	0	0	-3.11654320352907 (0.000213714303813288)	0
RP1-27K12.2	0	0	0	0	0	6.72386837267442 (7.61237980979555e-05)	0	0	0	0
RP1-29C18.10	-3.91094856617101 (0.00526356630039797)	0	0	0	0	0	0	0	0	0
RP1-29C18.8	-3.89688343253241 (0.00133164486073839)	0	0	0	0	0	0	0	0	0
RP1-29C18.9	-2.68078164930865 (0.00404921494487575)	0	0	0	0	0	0	0	0	0
RP1-301L19.1	0	0	0	0	0	5.98798819563888 (0.00416470186300144)	0	0	0	0
RP1-309I22.2	-4.16730270589843 (2.38469681760685e-14)	-6.78266889605679 (0.00231432343432402)	-2.3425751026091 (3.85174507439799e-05)	-4.37425208332261 (3.2163313044901e-06)	0	-2.14175877318895 (0.00957861408094294)	-1.84533154145498 (0.000152079636151866)	-5.44987226842568 (8.58305901757785e-07)	0	0
RP1-313I6.12	0	0	0	0	0	0	0	0	3.25760225625581 (0.00267970923096713)	0
RP1-60O19.1	0	0	0	0	0	8.99032368743066 (9.32081751291687e-13)	0	0	0	0
RP1-65J11.5	0	0	0	0	6.24434293510046 (0.0075784156376657)	7.41765384145497 (0.000641301505146358)	0	0	0	0
RP1-68D18.2	0	0	0	0	0	0	3.18452621129513 (0.00944148763662688)	0	0	0
RP1-78O14.1	0	0	0	0	0	0	0	0	5.97856136305101 (0.000746078021460784)	0
RP1-79C4.4	-2.28775658417993 (0.00231645710652011)	3.60840908264985 (0.000537530678685701)	0	0	0	0	0	0	0	0

RP11-100E13.3	0	0	0	0	0	6.94035970153732 (0.0018861097850123)	0	0	0	0
RP11-100K18.1	0	0	0	0	0	7.70041022663225 (0.00297678090088382)	0	0	0	0
RP11-1012E15.2	0	0	0	0	0	7.04063115215185 (0.00840552745288383)	0	0	0	0
RP11-1023L17.2	-2.03068428515533 (0.000852888305859187)	0	0	0	0	0	0	0	-2.02845137470489 (0.000605754079597094)	0
RP11-102K13.5	0	0	5.97840347135144 (4.71904785851401e-14)	0	0	0	0	0	0	0
RP11-103J17.2	0	0	0	0	0	0	0	0	4.07182283473454 (0.00258643907690718)	0
RP11-1069G10.1	3.55597290400585 (0.00045834738029243)	0	0	0	4.04568144963916 (0.00927186374840754)	3.97933648838725 (0.00474022188320757)	0	0	0	0
RP11-1072A3.3	0	0	0	0	0	3.32699876488496 (0.00089318887677339)	0	0	0	0
RP11-107E5.3	-1.75815900939254 (0.00100193041566904)	0	-3.67464910737615 (1.35957844597928e-05)	0	0	0	0	0	0	0
RP11-1086I4.2	0	0	0	0	0	5.71866664577082 (0.00610888751083178)	0	0	0	0
RP11-108K3.2	0	0	0	0	0	0	0	0	4.70190434703064 (0.000669495176406049)	0
RP11-108M9.3	0	0	5.97814030245736 (0.00719807252282974)	0	0	7.19604382376089 (1.3090370385242e-05)	3.93016641465347 (0.00795932809088759)	0	0	0
RP11-108O10.2	0	0	0	0	0	6.56519428926739 (0.0008335443074125)	0	0	0	0
RP11-109A6.2	0	0	0	0	0	5.20324739333798 (0.00933772649870102)	0	0	0	0
RP11-10A14.5	0	0	0	0	0	0	0	0	4.7565267360475 (0.00122295734722369)	0
RP11-10C8.2	0	0	0	0	0	6.94761621028195 (8.85699295509993e-05)	3.97741296508069 (0.00621374929825169)	0	0	0
RP11-10L12.2	-2.78141902201266 (0.005855735967354)	0	0	0	0	0	0	0	0	0
RP11-110I1.11	-4.70973481301403 (0.00304218578378204)	0	0	0	0	0	0	0	0	0
RP11-113E21.3	0	0	0	0	0	7.97896976436846 (0.00174461789794878)	0	0	0	0
RP11-113I24.1	0	0	0	0	6.47559966567641 (0.0042482603748159)	0	0	0	0	0
RP11-114G11.5	0	0	5.09770471445876 (0.00330160633536969)	0	0	0	0	0	0	0
RP11-114M5.1	0	0	-3.54223195481576 (0.00216759294792274)	0	0	0	0	0	-2.46792845578121 (0.00902925199995628)	0
RP11-1151B14.3	0	0	0	0	0	7.39824791663015 (1.63277984009593e-08)	0	0	0	0
RP11-115D19.1	0	0	0	0	0	0	0	0	3.62042249930997 (0.000236382475512164)	0
RP11-115J16.1	0	0	0	0	6.02995369297959 (0.0037021449159359)	7.00158609905914 (7.90630365570449e-05)	0	0	0	0
RP11-116D2.1	0	0	0	0	0	6.42768562482167 (0.00266331733114928)	0	0	0	0
RP11-117L5.1	0	8.24941804382365 (0.000117830374489767)	0	0	0	0	0	0	0	0
RP11-118E18.1	0	0	0	0	3.56999407704654 (0.00927235233218452)	0	0	0	0	0
RP11-120K18.3	-4.77828881539545 (0.000496936681135227)	0	0	0	0	0	0	0	-3.15934826465015 (0.00368756815560798)	0
RP11-1212A22.1	0	0	0	0	0	0	0	0	-2.11098644675077 (0.00697795638891459)	0
RP11-122A21.2	0	0	0	0	0	5.47063237031978 (0.00721408932438838)	0	0	0	0
RP11-123O10.4	0	0	0	0	0	0	0	0	4.41703937148175 (0.00205822342247181)	0
RP11-1246C19.1	0	0	0	0	4.70608989748324 (0.00268257944580698)	4.29546678719273 (0.00532055736677085)	0	0	0	0
RP11-1260E13.4	0	0	0	0	0	3.97020019590572 (0.000303877197991238)	0	0	0	0
RP11-126O22.1	0	0	0	0	7.0737635695201 (0.000527648380885118)	8.50765541015069 (7.6952613299065e-06)	0	0	4.5279503732046 (0.00182446426385003)	0
RP11-127L20.3	-3.99471124722894 (0.00254462011660628)	0	0	0	0	0	0	0	0	0
RP11-1299A16.3	0	0	0	0	7.07512549117957 (1.18550965029139e-05)	0	5.09667091026329 (0.00511153744808997)	0	0	0
RP11-12A16.3	0	0	0	0	0	0	0	0	4.54574786716669 (0.00264302133990233)	0
RP11-12A20.6	0	0	0	0	6.3357574308646 (0.000315237945466542)	0	0	0	0	0
RP11-130F10.1	0	0	0	0	0	0	0	0	3.48593701700403 (0.00511938160987337)	0
RP11-1336O20.2	4.14144506647391 (0.000149807679449907)	0	0	0	0	0	0	0	0	0
RP11-133K1.7	0	0	0	0	0	6.11516005283844 (0.00119397712963444)	0	0	0	0
RP11-134D3.1	0	0	0	0	6.47559966567641 (0.0042482603748159)	0	0	0	0	0
RP11-138B4.1	0	0	0	0	0	0	0	0	4.94871304521725 (9.66719756975013e-05)	0
RP11-139H14.5	0	0	0	0	0	7.44946195853488 (4.40366763268291e-05)	0	0	0	0
RP11-13K12.1	0	0	0	0	6.79415997022565 (0.00033123837273918)	6.01107390085474 (0.00326968591878455)	0	0	3.86200072344766 (0.00672574590685814)	0
RP11-13K12.2	0	0	0	0	6.12132713337718 (0.00201419557017153)	7.04558055332227 (0.000188446280611506)	0	0	0	0
RP11-141B14.1	0	0	0	0	0	4.30781053673035 (0.00182186837539409)	0	0	0	0
RP11-141M3.6	0	0	0	0	0	4.65127186749474 (0.00178144719705856)	0	0	5.86662748432869 (4.33535562997266e-13)	0
RP11-144I2.1	0	0	0	0	0	0	0	0	1.54376252530706 (0.00505441476359691)	0
RP11-144L1.8	0	0	7.10640594630608 (0.00170239609464387)	0	0	0	4.8603814479277 (0.00291908554290152)	0	0	0
RP11-147L13.2	0	0	0	0	4.79505878132525 (0.00832678078314052)	0	0	0	0	0
RP11-148L24.1	0	0	0	0	0	0	0	0	4.33981369977248 (0.00168310694749684)	0
RP11-14I17.1	0	0	0	0	0	5.52939196278266 (0.00147173421809754)	0	0	0	0
RP11-151A6.4	0	0	0	0	4.44210825274736 (0.00614703309105396)	4.69090844763073 (0.00107376993841506)	0	0	0	0
RP11-154J22.1	0	0	0	0	6.08631636127636 (6.09269181490974e-05)	5.42940011587143 (0.00209988952696788)	4.15874928152962 (0.00796693515024752)	0	0	0
RP11-155D18.13	0	0	0	0	0	7.01616419529879 (0.000134183751093026)	0	0	0	0
RP11-155L15.1	0	0	0	0	0	7.44488068581027 (0.000994330397208971)	0	0	0	0
RP11-156L14.1	0	0	0	0	0	7.06670291109509 (0.000126311416356477)	0	0	0	0
RP11-157G21.2	0	8.98932863502954 (9.19952661018782e-09)	0	0	0	0	0	0	5.35085572292512 (9.56598429555735e-06)	0
RP11-158H5.8	0	0	0	0	0	4.15879311202241 (0.00940824262332053)	0	0	0	0
RP11-158L12.6	0	0	0	0	0	6.25633710563463 (0.000118099084827667)	0	0	0	0
RP11-159D12.10	0	0	0	0	0	7.46102324576142 (0.000708109083945895)	0	0	0	0
RP11-159H10.3	0	0	0	0	0	7.74022813058379 (0.0021920031485895)	0	0	0	0
RP11-161M6.2	0	0	0	0	5.19532120089056 (0.000109508011837189)	0	0	0	0	0
RP11-162G10.1	0	0	0	0	0	3.8096198913009 (0.00805658766904098)	0	0	0	0
RP11-166B2.3	0	0	0	0	3.93539479449665 (0.00139443838484145)	0	0	0	0	0
RP11-166D19.1	1.99774421279152 (3.26870902367432e-06)	0	0	2.3429477438471 (0.00154503796536687)	0	0	0	0	0	0
RP11-168K9.2	0	0	0	0	7.20697900913001 (0.00515462419084721)	6.93037023825997 (0.00993412460458525)	0	0	0	0
RP11-169F17.1	0	0	0	0	0	6.68891162470414 (0.00306283178446008)	0	0	4.73227350946238 (0.000115623911444633)	0
RP11-169K17.3	0	0	0	0	0	0	0	0	3.74890837911394 (0.00914430881707623)	0
RP11-16E23.3	0	0	0	0	0	0	0	5.19583611911457 (0.0086274430580172)	0	0
RP11-171I2.1	0	0	4.74962621774767 (3.92647073113603e-08)	0	0	0	0	0	0	0
RP11-171I2.2	0	0	1.97810978500414 (0.0082004698709003)	0	0	0	0	0	0	0
RP11-171I2.3	0	0	4.54722755168337 (0.0088479216596711)	0	0	0	0	0	0	0
RP11-175O19.4	0	4.91824231110874	0	0	0	0	0	0	0	0

		(8.35963967310387e-06)								
RP11-178L8.5	0	0	0	0	0	6.8968980632387 (0.00193447755663905)	0	0	0	0
RP11-17A4.2	0	0	0	0	0	7.2732341871927 (0.000247616549737212)	0	0	0	0
RP11-17G12.2	0	0	-3.11288516984185 (0.00788004008640721)	0	0	0	0	0	0	0
RP11-17P16.1	0	0	0	0	0	5.65951094372531 (0.00559703911293668)	0	0	0	0
RP11-180M15.7	-4.01631466845325 (0.00522166929966994)	0	0	0	0	0	0	0	-3.19971428544646 (0.00640205376144666)	0
RP11-181C21.4	0	7.54641440367833 (3.59213765704542e-06)	0	0	0	0	0	0	0	0
RP11-188C12.2	0	3.50229698009626 (0.00097867187820339)	0	0	0	0	0	0	0	0
RP11-190A12.8	0	0	0	0	0	7.86260859032032 (0.0018821504921777)	0	0	0	0
RP11-193H22.2	0	0	0	0	0	5.00962947230226 (0.00510982350159659)	0	0	0	0
RP11-195F19.30	0	0	0	0	0	5.41081889563413 (0.00690114096067884)	0	0	0	0
RP11-195M16.3	0	0	0	0	0	6.16227444200091 (0.00275470600662641)	0	0	0	0
RP11-196E1.3	0	0	0	0	5.57857079249512 (0.00693709453128892)	6.68783166037613 (0.00359876711162708)	0	0	0	0
RP11-196G11.6	0	0	0	0	0	4.16374862884064 (0.00895153226554025)	0	0	0	0
RP11-196G18.22	0	0	-2.24827343836904 (0.00640913067009865)	0	0	0	0	0	0	0
RP11-1D12.2	0	0	0	0	0	0	0	6.80720155574357 (0.00424504638285965)	0	0
RP11-203I2.1	4.39888994074509 (0.00461102246614205)	0	0	0	0	0	0	0	0	0
RP11-204E4.4	0	0	0	0	0	6.1282743832105 (0.00972726320301328)	0	0	0	0
RP11-210M15.2	0	0	0	0	5.40268742539909 (0.00633417820591054)	6.11123764394419 (0.0001010025831508)	0	0	0	0
RP11-211G3.2	0	0	0	0	0	0	0	0	-2.18359658048692 (1.56992227106915e-05)	0
RP11-211N8.2	0	0	0	0	0	7.23775890656349 (0.0013154111801117)	0	0	0	0
RP11-212E4.1	-2.8774361935952 (0.00145397056408038)	0	0	0	0	0	0	0	0	0
RP11-212I21.3	-2.06801508525227 (0.00735470975741834)	0	-3.92759164413032 (0.000188830107016095)	0	0	0	-2.04392896963924 (0.000908780969577584)	0	-2.65458190184252 (0.000170373605876263)	0
RP11-212P7.2	0	0	0	0	0	3.56117453429369 (0.000831994251651822)	0	0	0	0
RP11-213H15.1	0	7.88954373723707 (5.94634687742774e-09)	0	0	0	0	0	0	0	0
RP11-214K3.21	0	0	0	0	0	0	0	0	1.94073435645263 (0.00817944179351389)	0
RP11-214O1.3	0	0	0	0	0	4.88736091411086 (0.00286327690499282)	0	0	0	0
RP11-215E13.2	0	0	0	0	5.7800642702998 (0.00591384444719865)	0	0	0	0	0
RP11-217B1.2	3.4871282291838 (0.000260859273913506)	0	6.22062318289001 (1.90858181137177e-06)	0	5.05008119014557 (9.0116914989858e-06)	6.46419317188608 (1.4672832317755e-09)	3.43754324301655 (0.000571201815990559)	0	2.95302723515899 (0.000972293585000819)	0
RP11-219B4.5	0	0	0	0	0	5.84427394053821 (0.00326408909597339)	0	0	0	0
RP11-21G15.1	0	0	0	0	0	6.18029103669083 (0.00894791953101118)	0	0	0	0
RP11-21K12.2	-3.18461807267102 (0.00121563323985078)	0	0	0	0	0	0	0	0	0
RP11-21L23.2	0	0	0	0	0	5.2114001162873 (8.28803680762531e-05)	0	0	3.79842571221798 (0.00140470589782733)	0
RP11-222K16.2	0	0	0	0	0	5.83152393176834 (0.00437029669897774)	0	0	0	0
RP11-226M10.3	0	0	0	0	0	3.04612670137936 (0.00567147781390516)	0	0	0	0
RP11-22B23.1	0	0	0	0	0	0	0	3.79506582119913 (0.00924307827988576)	0	0
RP11-231E4.2	0	0	0	0	0	7.10414613313287 (0.00135256292656179)	0	0	0	0
RP11-231E6.1	0	0	0	0	7.70543948974319 (6.99274277929523e-06)	0	0	0	0	0
RP11-231P20.2	0	0	0	0	0	6.38534498135925 (4.26287055699189e-05)	0	0	0	0
RP11-234B24.2	0	0	0	0	0	6.02008301910013 (0.000843064095466812)	0	0	0	0
RP11-234K24.3	0	0	0	0	0	3.95083376940837 (0.00937083842526962)	0	0	0	0
RP11-236P24.3	0	0	0	0	0	5.74534170193542 (0.00469603977423824)	0	0	0	0
RP11-242C19.2	0	0	0	0	0	0	0	0	-3.7612700608932 (0.00160190351211398)	0
RP11-243A14.1	0	0	0	0	0	6.56073355938923 (0.00442449246305087)	0	0	0	0
RP11-244F12.3	1.57819879062601 (0.00472815663194704)	0	1.83806127384838 (0.00477720117956053)	0	0	0	0	0	0	0
RP11-247C2.2	0	0	0	0	0	6.35947680596301 (0.00421653283516686)	0	0	0	0
RP11-248B24.1	0	0	0	0	0	6.12982525419746 (0.0018788647512987)	0	0	0	0
RP11-255B23.1	0	0	0	0	0	0	3.84676949084683 (0.008414668860105)	0	0	0
RP11-258B16.1	0	0	0	0	0	7.39204022818938 (0.00411240670568568)	0	0	0	0
RP11-259K5.2	-2.09636638082757 (0.00690586840027574)	0	-2.41134499724397 (0.00970195921392718)	0	0	0	0	0	0	0
RP11-25D10.2	0	0	0	0	0	5.85884223223625 (0.0019370928180933)	0	0	0	0
RP11-262H14.7	0	0	0	0	0	7.24475212967842 (0.00513532983523807)	0	0	0	0
RP11-263G22.1	0	0	0	0	0	6.10213009384402 (0.00755053791667713)	0	0	0	0
RP11-263K4.3	0	0	0	0	0	7.04063115215185 (0.00840552745288383)	0	0	0	0
RP11-267C16.1	0	0	0	0	0	6.6778957978631 (0.000559327717056785)	0	0	0	0
RP11-268G12.1	0	0	0	0	0	0	0	0	4.3832388953934 (0.00309078723408871)	0
RP11-274H24.1	0	0	0	0	0	0	0	0	5.18783780300963 (1.70576837012905e-05)	0
RP11-27I1.4	0	0	0	0	0	3.14150452371894 (0.00721894355061272)	0	0	0	0
RP11-280K24.4	0	0	0	0	6.95283227746436 (0.00102465732257152)	7.32624266357827 (0.00538433295435045)	0	0	0	0
RP11-283I3.6	0	0	0	0	0	2.88347967113921 (0.00857891459113206)	0	0	0	0
RP11-286N22.8	0	0	0	0	0	3.67604924828415 (0.000401534594148066)	0	0	0	0
RP11-288C17.1	0	0	0	0	0	0	0	0	4.46647493506467 (0.000485251202082093)	0
RP11-288E14.2	0	4.94349800668137 (0.00848698382760598)	0	0	0	0	0	0	0	0
RP11-288K12.1	0	0	0	0	0	0	0	0	-2.50204867936928 (0.0033374829463959)	0
RP11-289A15.1	4.07002391335022 (0.00316708063267221)	0	0	0	0	0	3.67059971339616 (0.00776212692083659)	0	0	0
RP11-290F5.1	0	0	0	0	0	4.0615012383012 (0.00290195044067408)	0	0	0	0
RP11-293M10.6	0	0	4.34622794409945 (0.00136449904405623)	0	0	6.60616704061126 (0.000928033847666575)	0	0	0	0
RP11-295D4.3	0	0	0	0	0	0	0	0	-2.24228227097053 (0.00234666514190117)	0
RP11-296E7.1	0	0	0	0	4.85162548311581 (0.000867697198451052)	4.98882072122395 (0.00262962933495006)	0	0	0	0
RP11-297L17.6	0	0	0	0	0	7.21873405049935 (0.000225500056946234)	0	0	0	0
RP11-298H24.1	0	0	0	0	7.33141360835363 (0.000333976211774335)	0	0	0	0	0
RP11-29G8.3	-3.66278297316254 (7.50190696608541e-05)	0	0	0	0	0	0	0	0	0
RP11-2A4.4	0	0	0	0	0	7.14261019565052 (0.00718963385642891)	0	0	0	0
RP11-2E11.6	0	0	0	0	0	0	0	0	2.58850994858997 (0.00165018571575845)	0
RP11-2H8.3	0	0	0	0	0	5.9930912090424 (0.000943941121590526)	0	0	0	0
RP11-2N1.2	0	0	0	0	0	0	0	0	-4.54022109816447 (0.00111556725338951)	0

RP11-2O17.2	0	0	0	0	0	5.92548431378617 (0.00951084815356596)	0	0	0	0
RP11-301G19.1	0	0	0	0	0	6.94035970153732 (0.0018861097850123)	0	0	0	0
RP11-301G23.1	0	0	0	0	0	0	0	0	3.17512585619402 (0.00880329894806737)	0
RP11-301L8.2	0	0	0	0	0	5.1965714264626 (0.00365689561403842)	0	0	2.87162811518103 (0.0089991616869)	0
RP11-305E6.4	0	0	0	0	0	4.73029751054221 (0.000440057327558534)	0	0	0	0
RP11-309L24.4	0	0	5.34354978355557 (0.00764966775836947)	0	0	0	0	0	0	0
RP11-30H9.1	0	0	0	0	0	0	0	0	3.77271319876822 (0.00504247321103256)	0
RP11-314N13.9	0	0	0	0	0	0	0	0	-3.74541376375994 (0.000467054239273172)	0
RP11-315E17.1	0	0	0	0	0	5.96297162967619 (0.00427178552237109)	0	0	0	0
RP11-318G21.4	0	0	0	0	0	0	0	0	3.83841293148795 (0.00711414327962516)	0
RP11-318K15.2	-3.22587442820902 (0.00334628656404539)	0	-3.42292285620555 (0.00381275716171964)	0	0	0	0	0	0	0
RP11-320P11.1	0	7.09455938338597 (5.97828370777017e-07)	0	0	0	0	0	0	0	0
RP11-325F22.2	0	0	0	0	0	0	0	0	-3.08247643081444 (0.000721171446254967)	0
RP11-326C3.2	0	0	0	0	0	0	0	0	-4.2430156180282 (0.00112112363768131)	0
RP11-327E2.5	0	0	0	0	0	6.07277201324841 (0.00802365726195738)	0	0	0	0
RP11-327F22.6	0	0	0	0	0	0	0	0	-4.32815984268302 (0.000695301902324412)	0
RP11-329L6.2	0	0	0	0	0	0	1.94004542573054 (0.001778351365)	0	0	0
RP11-331F4.4	0	0	0	0	0	6.61748565105267 (0.00329571580944009)	0	0	0	0
RP11-332E19.2	0	0	0	0	0	5.68992016380323 (0.00506721075968303)	0	0	0	0
RP11-332M2.1	0	0	0	0	0	0	0	0	3.11963175743641 (0.0015387442631791)	0
RP11-333A23.4	0	0	0	0	0	0	0	0	4.54369447886441 (9.31084851601802e-05)	0
RP11-334L9.1	0	8.24941804382365 (0.000117830374489767)	0	0	0	0	0	0	0	0
RP11-33E12.2	2.58930588331586 (0.000805829901386654)	0	0	0	0	0	0	0	0	0
RP11-33N14.3	0	0	0	0	7.01174960828215 (0.000223217186338613)	0	0	0	0	0
RP11-341D18.5	0	0	0	0	0	7.26986837985775 (0.00668205493536917)	0	0	0	0
RP11-345M22.2	0	0	0	0	6.65055932551467 (0.00282029564248256)	0	4.63992075909833 (0.0072477289986837)	0	0	0
RP11-346C20.3	0	0	0	0	0	0	0	0	3.69037393965583 (0.00138330292941733)	0
RP11-347C12.10	0	0	0	0	0	5.53533543574327 (0.00488620942992971)	0	0	0	0
RP11-347P5.1	-3.20123941569915 (0.00485693092660693)	0	0	0	0	0	0	0	0	0
RP11-349F21.2	0	0	0	0	0	4.58905187863487 (0.0044638553318319)	0	0	0	0
RP11-349F21.5	0	0	0	0	0	5.03030380197827 (0.000142442126751296)	0	0	0	0
RP11-350J20.9	0	0	0	0	0	6.5819059704344 (0.00420308949348701)	0	0	0	0
RP11-352M15.2	0	2.99650197086768 (0.00856615490564369)	0	0	0	0	0	0	0	0
RP11-354K4.2	0	0	0	0	0	5.92548431378617 (0.00951084815356596)	0	0	0	0
RP11-355F16.1	0	0	0	0	0	5.06861454708909 (0.00429579159437712)	0	0	0	0
RP11-356I2.4	0	0	-3.7954161163264 (0.00908679340286677)	0	0	0	0	0	0	0
RP11-358B23.7	0	0	0	0	0	0	2.67548757603819 (0.00274041936146757)	5.10695894502244 (0.00729010471731648)	3.36581073405056 (0.00118263913881277)	0
RP11-358D17.2	6.23047987806768 (3.49572550542732e-12)	0	0	5.26906060209339 (0.0042922322201194)	0	0	0	0	0	0
RP11-360A18.2	0	0	0	0	0	6.93037023825997 (0.00993412460458525)	0	0	0	0
RP11-362F19.1	0	0	0	0	0	4.93037053838418 (0.000742234394383194)	2.95819036432797 (0.00860462726181189)	0	5.91798738035745 (0.000236172613051933)	0
RP11-363E7.4	0	0	0	0	0	0	0	0	4.20748549988187 (3.23191837319911e-06)	0
RP11-365D9.1	0	0	0	0	0	6.7718725898029 (0.00239250939652811)	0	0	0	0
RP11-368J22.2	4.31468582160908 (0.00611745449087952)	0	0	0	0	0	0	0	0	0
RP11-368M16.7	0	0	0	0	0	6.61748565105267 (0.00329571580944009)	0	0	0	0
RP11-371I20.2	0	0	0	0	0	6.69472262774014 (0.00261650665231987)	0	0	0	0
RP11-378I6.1	0	0	0	0	0	6.48160421241758 (0.00574307940253469)	0	0	0	0
RP11-379K17.4	0	0	0	0	0	7.04063115215185 (0.00840552745288383)	5.48125617905839 (0.00700702075989653)	0	0	0
RP11-37B2.1	0	0	0	0	0	0	0	0	1.5029491848561 (0.000277461694625734)	0
RP11-37C7.3	0	0	0	0	0	6.99916031096431 (0.000649457772856325)	0	0	0	0
RP11-380G5.3	0	0	0	0	0	0	-1.74486201123727 (0.00931902149136946)	0	0	0
RP11-382M14.1	0	0	0	0	0	6.16980119559886 (0.00649144516590301)	0	0	0	0
RP11-384F7.2	0	0	0	0	0	0	0	0	-4.28138612212609 (0.00311832224527824)	0
RP11-384P7.7	3.37839351770781 (0.00890204188189482)	0	0	0	0	0	0	0	0	0
RP11-385H1.1	0	0	0	0	0	6.469711239845 (0.00392260125449649)	0	0	0	0
RP11-386M24.3	0	0	0	0	0	4.31778892296193 (0.00208545541753213)	0	0	0	0
RP11-390D11.1	0	0	0	0	0	0	0	0	3.79355082407682 (0.00384994017052524)	0
RP11-391A7.1	0	0	0	0	0	4.60304568659526 (0.00998947038167553)	0	0	0	0
RP11-394O4.5	3.62001409821385 (9.75647178308742e-14)	0	0	0	0	0	0	0	0	0
RP11-395G23.3	0	0	0	0	0	4.19621188421715 (0.00294001123326365)	0	0	0	0
RP11-395N21.2	0	0	0	0	0	0	0	0	-3.10941477758025 (0.000107241635032998)	0
RP11-3P17.3	0	4.62109023015095 (2.32480353567552e-05)	0	0	0	0	0	0	0	0
RP11-3P17.5	3.3655888096226 (0.00729961198625472)	6.51765424391886 (0.00154435519646877)	0	0	0	0	0	0	0	5.23020416598421 (0.00710610934258347)
RP11-400K9.2	0	0	0	0	0	7.39204022818938 (0.00411240670568568)	0	0	0	0
RP11-400L8.2	-4.10474037800696 (0.00598020940403373)	0	0	0	0	0	0	0	0	0
RP11-401L13.4	0	0	0	0	0	4.38699973478851 (0.00747203506511934)	0	0	0	0
RP11-401P9.6	0	0	0	0	0	4.77627759023307 (0.00261154713655066)	0	0	0	0
RP11-403N16.4	0	0	0	0	0	6.49092718433921 (0.000564125720018054)	0	0	0	0
RP11-409C19.2	0	0	0	0	0	5.20121098949655 (0.00431800118949873)	0	0	0	0
RP11-415C15.2	0	0	0	0	6.48222785639122 (0.00467673319314815)	6.8747978295475 (0.00948482808621846)	0	0	0	0
RP11-415J8.3	0	0	0	0	0	0	0	0	-1.85074746356712 (0.000862252284561741)	0
RP11-416I2.1	0	0	0	0	0	6.0645547329706 (0.00760120533172173)	0	0	0	0
RP11-417F21.1	-1.69753014432223 (0.00980600643209034)	0	-2.50723410643531 (0.00349766812217536)	0	0	0	0	0	0	0
RP11-41L14.1	0	0	0	0	0	6.29777569738134 (0.00633313190947706)	0	0	0	0
RP11-420A23.1	0	0	0	0	0	0	0	0	1.73389346176244 (0.0079982655901979)	0
RP11-420J11.1	0	0	0	0	0	7.35779101142259 (0.00500184185889881)	0	0	0	0
RP11-423E7.2	-2.16309315412466	0	-2.60425459692516	0	0	0	0	0	0	0

	(0.00512451821126086)		(0.00101519254304551)							
RP11-425D17.1	0	0	0	0	0	7.44279116366548 (0.00404563973443074)	0	0	0	0
RP11-428C6.2	0	0	0	0	0	6.0645547329706 (0.00760120533172173)	0	0	0	0
RP11-428P16.2	0	0	0	0	0	6.3104054400639 (0.00647666131582755)	0	0	0	0
RP11-430C1.1	0	0	0	0	0	7.3837434313229 (0.00493066199114422)	0	0	0	0
RP11-431K24.1	0	0	0	0	4.31734282129104 (0.00820889163818742)	0	0	0	0	0
RP11-433J8.1	0	0	0	0	0	6.469711239845 (0.00392260125449649)	0	0	0	0
RP11-434B12.1	0	0	4.22468941151637 (0.00847394252929553)	0	0	0	0	0	0	0
RP11-434D9.1	0	0	0	0	0	5.7626642639173 (0.00243800724357047)	0	0	0	0
RP11-434E6.2	0	0	0	0	0	7.40960786669647 (0.00472974266362044)	0	0	0	0
RP11-436I24.1	3.41908401558678 (0.00520278059880528)	0	0	0	0	0	0	0	0	0
RP11-437B10.1	-1.70637757444074 (0.000306462899830279)	0	-3.05316921396953 (1.38917762595281e-07)	0	0	0	0	0	0	0
RP11-439E19.10	2.9759233394795 (0.00551007494692477)	0	0	0	0	0	0	0	3.81942907828174 (6.72857581720396e-05)	0
RP11-439E19.9	0	0	0	0	0	6.31040539604999 (0.00647657119037574)	0	0	0	0
RP11-43D4.3	-2.92257954921245 (0.00685983749040869)	0	0	0	0	0	0	0	0	0
RP11-43N16.4	0	7.32763567695108 (5.68684896307108e-10)	0	0	0	0	0	0	0	0
RP11-441F2.5	0	0	0	0	0	7.92249751581524 (0.00165203987131941)	0	0	0	0
RP11-442O1.3	0	0	0	0	0	6.09483344452107 (0.00930799072827987)	0	0	0	0
RP11-445N18.7	0	0	0	0	0	5.19452407755394 (0.00220354594587985)	0	0	0	0
RP11-446E24.3	0	0	0	0	0	6.98841105423508 (0.00182283012072817)	0	0	0	0
RP11-446H18.5	0	0	0	0	0	0	0	0	4.9669164964391 (3.89024403244526e-07)	0
RP11-448G15.3	2.3330396064186 (0.00595567476987052)	0	0	0	0	0	0	0	0	0
RP11-449L23.3	0	0	0	0	0	6.37117209463019 (0.00442564728836765)	0	0	0	0
RP11-44F21.3	0	0	0	0	0	4.47669133313872 (0.00242798227679342)	0	0	2.43437861896819 (0.00574600121042932)	0
RP11-452I5.2	0	0	0	0	0	7.44626286508361 (0.000235329803280829)	0	0	0	0
RP11-455F5.6	0	4.83621533303824 (0.000683847871369215)	0	0	0	0	0	0	0	0
RP11-457M11.7	0	0	0	0	0	6.89568939211318 (0.0015900532405385)	0	0	0	0
RP11-45A16.4	0	0	0	0	0	0	0	0	5.00726950955334 (0.00180678711038931)	0
RP11-45A17.3	0	0	0	0	4.27346145223697 (0.00932851968293045)	0	0	0	0	0
RP11-45K10.2	0	0	0	0	0	6.28556277714431 (0.00564354943434322)	0	0	0	0
RP11-45M22.2	-2.50753843570016 (0.00797140642695624)	0	0	0	0	0	0	0	0	0
RP11-461F11.2	0	0	0	0	6.58518154506848 (0.000384285781295498)	7.12387322731736 (0.000183811993047597)	5.33195152779515 (0.00853184883913036)	0	0	0
RP11-462L8.1	0	0	0	0	0	6.68508241595046 (0.00319809764628151)	0	0	0	0
RP11-463O12.5	-2.36185132236411 (2.66441399512103e-06)	0	-2.20944516663225 (1.79213196670118e-06)	-2.36889854527857 (0.0023084832202229)	0	0	0	0	0	0
RP11-463O9.9	0	0	0	0	5.16397311622983 (0.00337117825453439)	6.81941106969351 (8.93208816859244e-06)	0	0	0	0
RP11-468E2.5	0	0	0	0	0	0	0	0	3.3765621273641 (0.00618267056557992)	0
RP11-46C24.7	0	0	0	0	0	3.94732230939338 (0.00203692200694421)	0	0	0	0
RP11-46E17.8	0	0	0	0	0	7.63242936931039 (9.78699843051384e-05)	0	0	0	0
RP11-470L19.2	0	0	0	0	6.798559966251 (0.0016583499845375)	0	0	0	0	0
RP11-471B22.3	0	0	0	0	0	6.03284904247274 (0.00999153400476506)	0	0	0	0
RP11-471M2.3	0	0	0	0	0	7.10375123425089 (0.00121982743592521)	0	0	0	0
RP11-474P2.7	0	0	0	0	0	0	0	0	2.14993460191927 (0.00397551004612629)	0
RP11-475J5.6	0	0	0	0	3.85636733739381 (0.00591597306807263)	0	0	0	0	0
RP11-475O6.1	0	0	0	0	4.73699326924491 (0.00591913113873846)	4.31186770482692 (0.00785489989196375)	0	0	0	0
RP11-476D10.1	-5.47158502446159 (0.000560874343052942)	0	0	0	0	0	0	0	-5.52182691365992 (0.000118059536707722)	0
RP11-477N12.3	0	0	0	0	6.64816874282679 (0.00347040281013931)	0	0	0	0	0
RP11-478B9.3	0	0	0	0	0	6.28446816679906 (0.00531418671143485)	0	0	0	0
RP11-478J18.2	0	0	0	0	0	5.77340500928175 (0.000443578165355569)	0	0	0	0
RP11-479O17.10	0	0	0	0	0	5.47735170402561 (0.00338120282616536)	0	0	0	0
RP11-480I12.5	0	0	0	0	0	5.8995180710935 (0.00347685775651768)	0	0	0	0
RP11-480I12.7	0	0	0	0	0	5.26461602707367 (0.00861192717171421)	0	0	0	0
RP11-481F24.3	0	0	0	0	0	0	0	0	6.08812031060771 (4.92119218904207e-08)	0
RP11-486M23.1	0	0	0	0	0	7.26986837985775 (0.00668205493536917)	0	0	0	0
RP11-486O12.2	0	0	0	0	0	3.81681300271064 (0.00856930241610198)	3.18117972976897 (0.000914905178251484)	0	0	0
RP11-488L18.4	0	0	0	0	0	-2.36320268776624 (0.00225947782755399)	0	0	0	0
RP11-488L18.8	0	0	0	0	0	0	4.07320389926518 (0.000126680771184489)	0	0	0
RP11-490G8.1	0	8.66652531635187 (1.8325534367635e-07)	0	0	0	0	0	0	0	0
RP11-491F9.3	0	0	0	0	0	7.68777182051244 (2.93074736511543e-05)	0	0	0	0
RP11-492D6.3	0	0	0	0	0	7.40324802854046 (0.000782677106135631)	0	0	0	0
RP11-493E12.2	0	0	-2.70541951116779 (0.00678873797812714)	0	0	0	0	0	0	0
RP11-494M8.4	0	0	0	0	0	0	0	0	4.34266351134366 (8.61243465162147e-05)	0
RP11-497J7.1	0	0	0	0	0	6.10213009384402 (0.00755053791667713)	0	0	0	0
RP11-4B16.4	-2.55078814784291 (0.00351011425573563)	0	-3.13323670394128 (0.000921430646492849)	0	0	0	0	0	-2.78879820397862 (0.000202424494461647)	0
RP11-502F1.1	0	0	0	0	0	6.28556277714431 (0.00564354943434322)	0	0	0	0
RP11-505K1.1	0	0	0	0	0	7.26986837985775 (0.00668205493536917)	0	0	0	0
RP11-506K19.2	0	0	5.06636929617248 (0.00456911430005041)	0	0	0	0	0	0	0
RP11-506O24.1	0	0	0	0	3.99010729892034 (0.00388563593183878)	0	0	0	0	0
RP11-506O24.2	0	0	0	0	0	4.98232120888102 (0.00585431492324909)	0	0	0	0
RP11-507B12.2	0	0	0	0	0	6.81038216101644 (0.000299334745572648)	0	0	0	0
RP11-508N22.12	0	4.09197561943108 (0.0025581042649675)	0	0	0	0	0	0	0	0
RP11-509A17.3	0	0	0	0	0	6.8747978295475 (0.00948482808621846)	0	0	0	0
RP11-510J16.5	0	0	0	0	0	0	0	0	4.34444918971329 (0.00995432727534731)	0
RP11-513N24.1	0	0	0	0	0	0	0	0	-4.39562017626817 (0.000657224323681235)	0
RP11-517M22.1	0	0	0	0	6.798559966251 (0.0016583499845375)	7.00400076500153 (0.00893319973200063)	0	0	0	0
RP11-521H3.3	0	0	0	0	0	5.92548431378617 (0.00951084815356596)	0	0	0	0
RP11-522B15.3	0	0	0	0	0	3.44782869785634 (0.00413742566852773)	0	0	0	0

RP11-528A10.1	0	0	0	0	0	6.15105400473411 (0.00203690868321723)	0	0	0	0
RP11-532N4.2	0	0	8.14311735393105 (5.15690062718114e-11)	0	0	0	0	0	0	0
RP11-534L6.3	0	0	0	0	5.7800642702998 (0.00591384444719865)	0	0	0	0	0
RP11-534P6.1	0	0	0	0	0	6.62972050443079 (0.00300730370188095)	0	0	0	0
RP11-539I5.1	0	0	0	0	0	0	0	0	-2.48641919101493 (0.00340326623879435)	0
RP11-53B2.2	-2.24116110246904 (0.00160159787147046)	0	0	0	0	0	0	0	0	0
RP11-53O19.1	3.41222738092989 (0.00129091880131296)	0	0	0	0	0	0	0	0	0
RP11-542P2.2	0	0	0	0	0	6.12759210531209 (0.00218637561515686)	0	0	0	0
RP11-545A16.1	0	0	0	0	0	7.28272757431076 (0.0048656497259573)	0	0	0	0
RP11-545A16.3	0	0	0	0	0	7.06087707931934 (0.0019802582374235)	0	0	0	0
RP11-546B8.6	0	0	0	0	6.12132713337718 (0.00201419557017153)	0	0	0	0	0
RP11-547D13.1	0	0	0	0	0	6.06240032056244 (0.00218102523153485)	4.93785957383308 (0.00121192554778034)	0	0	0
RP11-548K12.2	0	0	0	0	0	7.07308077663729 (0.00138932745134119)	0	0	0	0
RP11-54H7.4	0	0	0	0	0	0	0	0	6.52069950306173 (7.70569123398802e-07)	0
RP11-54O7.3	0	0	0	0	0	5.57089707256175 (0.000505249620405112)	0	0	0	0
RP11-550H2.2	0	0	0	0	6.48222785639122 (0.00467673319314815)	0	0	0	0	0
RP11-551L14.1	0	0	0	0	0	6.95502175084532 (0.00187089796090811)	0	0	0	0
RP11-559N14.5	0	0	0	0	6.18525407318681 (0.00135010533343173)	7.79770286481405 (6.43345031941434e-05)	0	0	0	0
RP11-561I11.3	0	0	0	0	0	6.38968800314438 (0.00475066945748178)	0	0	0	0
RP11-561O4.1	0	0	0	0	0	0	0	0	3.7954424610051 (0.00139146576882661)	0
RP11-563J2.2	0	0	-3.73238443321109 (0.00391968388398632)	0	0	0	0	0	-2.5398514572264 (0.00497231420381771)	0
RP11-563J2.3	0	0	-2.89010152910309 (0.00809268066297328)	0	0	0	0	0	-3.03666021606403 (0.000643772732540829)	0
RP11-568J23.2	0	0	0	0	0	6.28293043341668 (0.00114601734092013)	0	0	0	0
RP11-569G13.3	0	0	0	0	0	6.00813568053682 (0.00236776366027671)	0	0	0	0
RP11-571I18.5	0	4.18006617004452 (0.00467981157790746)	0	0	0	0	0	0	0	0
RP11-571O6.2	0	0	0	0	5.7800642702998 (0.00591384444719865)	7.32624266357827 (0.00538433295435045)	0	0	0	0
RP11-572M11.4	0	0	0	0	0	5.97253057444869 (0.00289993299153972)	0	0	0	0
RP11-573D15.8	0	0	0	0	0	6.87940427440784 (0.00036699593544116)	0	0	0	0
RP11-574K11.32	0	0	0	0	3.0545827301897 (0.00857102640650523)	0	0	0	0	0
RP11-582J16.4	0	0	0	0	0	0	0	0	1.89355991910856 (0.00844391504970364)	0
RP11-587D21.1	0	7.66282511003579 (0.000226746650776502)	0	0	0	0	0	0	0	0
RP11-588K22.2	2.60145061086865 (0.000301685883872176)	0	0	0	0	0	0	0	0	0
RP11-58O9.2	0	0	0	0	0	6.50776685108626 (0.0047726133768482)	0	0	5.64149085540055 (0.00315826001045313)	0
RP11-598F7.4	0	0	0	0	0	7.00881329009256 (0.00886510847483269)	0	0	0	0
RP11-59C5.3	0	0	0	0	0	0	0	0	0	-6.21851819094878 (0.00891345823511194)
RP11-600F24.7	0	0	0	0	0	4.80119066789864 (0.000416887109329294)	0	0	0	0
RP11-609N14.4	-2.30293707576456 (0.00247345740472119)	0	0	0	0	0	0	0	0	0
RP11-60L3.1	0	0	0	0	5.6726939916121 (0.00519788447491544)	0	0	0	0	0
RP11-60L3.3	0	0	0	0	0	5.83911235564155 (0.00377717823355608)	0	0	0	0
RP11-617F23.1	1.63345901720149 (0.00730455790960407)	0	2.39599173580244 (0.00186290072404372)	0	0	0	0	0	0	0
RP11-618N24.1	0	0	4.52651340169495 (0.00224282288118136)	0	0	0	0	0	0	0
RP11-61A14.4	0	0	0	0	0	6.03255908330019 (0.00959380943999706)	0	0	0	0
RP11-61L23.2	0	0	0	0	0	0	0	0	3.16684946906362 (0.00221326307317653)	0
RP11-624L4.1	0	0	0	0	4.53409685034146 (0.00957854386889111)	0	0	0	0	0
RP11-626E13.1	0	0	3.79963666745899 (0.00047636068682346)	0	0	0	0	0	0	0
RP11-627G23.1	0	0	0	0	5.69873733948859 (6.60913162355083e-05)	5.88326726599142 (7.70487265752139e-05)	0	0	0	0
RP11-629E24.2	0	0	0	0	0	7.05645578158446 (0.00152284467383944)	0	0	0	0
RP11-637N19.1	0	0	0	0	0	0	0	0	-2.43690841014863 (0.00830753642817176)	0
RP11-638L3.2	0	0	0	0	0	7.18609492789082 (0.00581362640846632)	0	0	0	0
RP11-640N11.2	0	0	0	0	0	5.83158182413771 (0.00113287562342157)	0	0	0	0
RP11-641A6.5	0	0	0	0	0	6.42311191480159 (0.0072846205182169)	0	0	0	0
RP11-642P15.1	0	0	0	0	0	0	4.22298338933743 (0.00914552810012761)	0	0	0
RP11-644F5.11	0	0	2.90129224292934 (5.97518976646545e-05)	0	0	0	0	0	0	0
RP11-649A18.4	0	0	0	0	0	0	0	0	3.5710919318852 (0.00882732340336582)	0
RP11-64B16.2	0	0	2.69363884163759 (0.000935259691086684)	0	0	0	0	0	0	0
RP11-64B16.4	-4.43121698445985 (0.00527760637000281)	0	0	0	0	0	0	0	0	0
RP11-64C12.3	0	0	0	0	6.8239605899821 (0.000267509886562154)	0	0	0	0	0
RP11-651P23.4	0	0	0	0	0	5.42652210454542 (0.00623392152737592)	0	0	0	0
RP11-65I12.1	0	0	0	0	3.47671831416206 (0.00785808808761138)	0	0	0	2.55750800070538 (0.000214238832103482)	0
RP11-65J21.3	0	0	0	0	0	0	0	0	4.004830665572 (0.00240010586147334)	0
RP11-65J3.14	0	0	0	0	0	6.50880491762822 (0.000552243498721524)	0	0	0	0
RP11-65L3.3	0	0	1.56489193590074 (0.00901227709145369)	0	0	0	0	0	0	0
RP11-65L3.4	0	0	4.24464091045619 (0.00265411574143865)	0	0	0	0	0	0	0
RP11-66B24.4	0	5.66947857423933 (8.83463531993782e-07)	0	0	2.85012559429214 (0.00975621886862173)	3.05845670322979 (0.00577952635008913)	0	0	0	0
RP11-66D17.3	0	0	0	0	0	6.96331268626672 (0.00168454536380207)	0	0	0	0
RP11-66N24.7	0	0	0	0	0	4.86057639645884 (0.00797629134339529)	0	0	0	0
RP11-66N5.2	0	0	0	0	0	7.11280790296114 (0.0064888542547563)	0	0	0	0
RP11-673E11.2	0	0	0	0	0	7.20898483477395 (0.00651838541340656)	0	0	0	0
RP11-676J12.6	0	0	0	0	0	5.25511098632939 (0.00421855639283794)	0	0	0	0
RP11-679B19.1	0	0	0	0	0	4.13261909759029 (0.00434142352094437)	0	0	0	0
RP11-680G24.5	-1.78794793774703 (0.00393026634674119)	0	0	0	0	0	0	0	-2.09503027193349 (0.000220803085903071)	0
RP11-681L4.2	0	0	0	0	6.80050039693727 (0.00176612853259011)	0	0	0	0	0
RP11-689P11.2	0	0	0	0	0	4.41225341603063 (0.00533283915557935)	0	0	0	0
RP11-690D19.3	3.35372109929989 (0.000144526968325195)	0	0	0	0	0	0	0	2.33256943502116 (0.00790999028421805)	0
RP11-693J15.5	0	0	0	0	6.58310840693429 (0.000390122548621893)	0	4.89165877017713 (0.00697105265742774)	0	0	0
RP11-696N14.1	2.01032061245631	0	0	0	0	3.59557620008427	0	0	0	0

	(0.00636115089043356)					(0.001651976903394)				
RP11-6N17.2	0	0	0	0	0	5.82560779467676 (0.00247807400370962)	0	0	0	0
RP11-6N17.4	0	0	3.37503120391794 (0.00278570636741595)	0	0	0	0	0	0	0
RP11-6O2.2	0	0	0	0	0	7.28272757431076 (0.0048656497259573)	0	0	0	0
RP11-6O2.3	4.57429369736513 (8.65252539499251e-07)	0	0	4.6365877791597 (0.00343042683788704)	0	0	0	0	0	0
RP11-6O2.4	2.77435259696955 (0.00740229971517807)	0	0	4.64955151173084 (0.00619118558119638)	0	0	0	0	0	0
RP11-701H24.4	1.84610501816452 (0.00181449913130287)	0	0	0	0	0	0	0	0	0
RP11-701P16.2	-2.91975093078548 (0.00731222254529468)	0	0	0	0	0	0	0	0	0
RP11-707P17.2	0	0	-3.88069263330056 (0.00184147867289523)	0	0	0	0	0	0	0
RP11-70C1.3	0	0	0	0	0	0	0	0	4.48446522558682 (0.000311084061545055)	0
RP11-70D24.1	0	0	0	0	0	6.76989746416059 (0.00250980415492344)	0	0	0	0
RP11-70F11.11	0	0	0	0	0	6.38968800314438 (0.00475066945748178)	0	0	0	0
RP11-70F11.8	0	0	0	0	0	6.97721416115344 (0.000648953325961982)	0	0	0	0
RP11-712B9.2	0	0	0	0	4.6716215116057 (0.00152879087236162)	4.56156424149678 (0.000791756002101872)	3.37853751116212 (0.00269695207420688)	0	0	0
RP11-713M15.2	0	0	0	0	7.21847437014238 (0.000749985002848081)	0	0	0	0	0
RP11-713P17.3	0	0	0	0	0	0	3.27252472790467 (0.00965324995711854)	0	0	0
RP11-714M23.2	0	0	0	0	0	6.93037023825997 (0.00993412460458525)	0	0	0	0
RP11-727A23.10	0	0	0	0	0	5.4081740098773 (0.00847169824293007)	0	0	0	0
RP11-728E14.3	0	0	0	0	0	6.03284916292113 (0.00999169881409401)	0	0	0	0
RP11-728K20.2	0	0	0	0	0	4.45360266606807 (0.00541971132207409)	0	0	0	0
RP11-730K11.1	0	0	0	0	0	7.17635473365475 (0.00175848211225834)	4.88744654062254 (0.00850540916740235)	0	0	0
RP11-733O18.1	0	0	0	0	0	0	0	0	4.15267972572501 (0.000227413679283459)	0
RP11-734K2.4	0	0	0	0	0	2.83160927201708 (0.0088294471601049)	0	0	0	0
RP11-744K17.2	0	0	0	0	6.47559966567641 (0.0042482603748159)	0	0	0	0	0
RP11-74D7.1	0	0	0	0	0	7.1378285905275 (0.00836124800875275)	0	0	0	0
RP11-74E22.8	0	0	0	0	0	7.35659971205863 (0.000837188281058539)	0	0	0	0
RP11-752D24.2	0	6.67683957370254 (6.41067618842315e-06)	0	0	0	0	0	0	0	0
RP11-752L20.3	-1.93555837506368 (0.000574841641869469)	0	0	0	0	0	0	0	0	0
RP11-753A21.2	0	0	0	0	0	6.43127452747204 (0.0018507180040074)	0	0	0	0
RP11-753D20.4	0	0	0	0	0	7.11254271341322 (0.00756733940646282)	0	0	0	0
RP11-753H16.3	5.53312570567889 (8.75957261166491e-05)	0	0	0	0	0	0	0	0	0
RP11-755E23.2	0	0	0	0	0	6.57967538783077 (0.00353011144244189)	0	0	0	0
RP11-756P10.2	0	0	0	0	0	5.74830536432748 (0.00478377246877675)	0	0	0	0
RP11-757O6.6	0	0	0	0	0	0	0	0	4.42513797068986 (0.00338473118519741)	0
RP11-760D2.11	0	0	0	0	0	7.37268168972322 (0.00412544184759699)	0	0	0	0
RP11-760H22.2	3.92036266593665 (0.00939047253053375)	0	0	0	0	0	0	0	0	0
RP11-764K9.2	0	0	0	0	0	0	4.61077309238408 (0.00602843059426705)	0	0	0
RP11-766F14.2	0	0	7.99264553799121 (4.90564560711072e-07)	0	0	0	0	0	0	0
RP11-766N7.3	0	0	0	0	0	7.61229752047163 (0.000418717965802401)	0	0	0	0
RP11-76C10.6	0	0	0	0	0	4.03933405781494 (0.00366685522571253)	0	0	0	0
RP11-774O3.3	2.64075079842751 (0.00408705327777921)	0	0	0	0	4.20929500432534 (0.00553886941152028)	0	0	0	0
RP11-778O17.4	0	0	0	0	0	6.52635379226284 (0.00623778701307938)	0	0	0	0
RP11-77K12.9	0	0	0	0	0	6.46591274906293 (0.00110624988633031)	4.52381444116684 (0.00764849768289529)	0	0	0
RP11-794P6.1	0	0	0	0	5.34825679501694 (0.00868755817628065)	0	0	0	0	0
RP11-796E2.4	0	0	0	0	0	0	3.70899495225131 (0.00991839848259924)	0	0	0
RP11-803D5.1	-5.14198238647496 (2.00983056297341e-06)	0	0	0	0	0	0	0	-1.7910352060873 (0.00348746984807538)	0
RP11-805I24.1	0	0	0	0	6.65447770053204 (0.00254565647500638)	8.02523049899765 (0.00016200018312921)	0	0	0	0
RP11-80I3.1	0	0	0	0	0	6.75612918803747 (0.00256172608728777)	0	0	0	0
RP11-814P5.1	0	0	0	0	0	6.50798433643379 (0.000518052762921488)	0	0	0	0
RP11-81A1.6	-1.82629280008085 (0.00385058109320283)	0	-2.92415699427969 (0.000231633911894566)	0	0	0	0	0	-1.69564437586913 (0.00435999823567886)	0
RP11-81B10.2	0	0	0	0	0	0	0	0	6.83716988936406 (2.93473669683978e-07)	0
RP11-834C11.4	4.57096744997759 (2.77635528028721e-06)	0	0	0	5.22490343971308 (0.000110832357661001)	0	0	0	0	0
RP11-83B20.3	0	0	0	0	0	0	0	0	-2.57062178527386 (0.00685291188791983)	0
RP11-844P9.2	0	0	0	0	0	6.12218764532471 (0.00139719253409669)	0	0	0	0
RP11-846F4.6	0	0	0	0	5.20427474374146 (0.00286463776503444)	0	0	0	0	0
RP11-84A19.2	0	0	0	0	0	5.82411293603098 (0.00387851086110505)	0	0	0	0
RP11-85B7.2	3.12981007155934 (0.00217489756421039)	0	0	0	0	5.71304068655683 (1.44757044538629e-06)	0	0	0	0
RP11-85G21.3	0	0	0	0	0	0	0	6.70181843905157 (0.000861619953104342)	0	0
RP11-861L17.3	-4.57489215430156 (0.00273043498346104)	0	0	0	0	0	0	0	0	0
RP11-862L9.2	0	0	0	0	0	5.09840051002355 (0.00152063492738053)	0	0	0	0
RP11-863P13.4	0	0	0	0	0	6.99788314372245 (0.00163879071541362)	0	0	0	0
RP11-87G24.2	0	0	0	0	0	6.15936048893587 (0.00674426297960733)	0	0	0	0
RP11-88E10.4	0	0	0	0	0	6.51408998396268 (0.00487467536872951)	0	0	0	0
RP11-88I18.2	4.84919805989572 (5.28931401627341e-15)	0	0	5.42936138918224 (3.72295936864611e-06)	0	0	0	0	0	0
RP11-88I18.3	5.15826336334757 (0.000395316617035292)	0	0	0	0	0	0	0	0	0
RP11-902B17.1	-3.15636037038728 (0.000313698530956644)	0	0	-6.095366290843 (0.00823536649619349)	0	0	0	0	0	0
RP11-907D1.3	0	0	0	0	0	7.44279116366548 (0.00404563973443074)	0	0	0	0
RP11-91J19.2	0	0	0	0	0	0	0	0	3.35018397144651 (0.00513581484563603)	0
RP11-91J3.1	0	0	0	0	0	0	0	0	4.62301476993937 (0.00274087087090677)	0
RP11-925D8.3	-5.08405548795608 (0.000637471703545899)	0	0	0	0	0	0	0	0	0
RP11-925D8.6	-6.28154500339173 (2.19286337911483e-05)	0	-4.15238177089942 (0.0017488992896655)	0	0	0	0	0	-4.73011493520561 (4.93029983219662e-05)	0
RP11-93B14.10	-4.71117312270541 (0.00364727643587349)	0	0	0	0	0	0	0	-4.72456904130904 (0.00136063887911581)	0
RP11-93B14.6	-3.78368790573811 (0.00416960408181372)	0	0	0	0	0	0	0	-4.06308629261862 (0.000282194017917227)	0
RP11-93B14.9	0	0	0	0	0	0	0	0	-3.39134058379137 (9.37213479830551e-05)	0
RP11-95D17.1	0	0	0	0	0	0	0	0	2.73160572208351 (0.00659822433953597)	0
RP11-95G6.1	0	0	0	0	0	0	0	0	4.80910827321531 (7.59735053005123e-05)	0

RP11-95M5.1	0	0	0	0	0	6.16856019215855 (0.00609985852466005)	0	0	0	0
RP11-960L18.1	0	0	0	0	0	6.22193051753834 (0.00858811897833788)	0	0	0	0
RP11-967K21.1	0	0	0	0	5.40218893588817 (0.00575050063442152)	0	0	0	0	0
RP11-96C21.2	0	0	0	0	0	5.97585847581896 (0.00254854874233337)	5.09577711093343 (0.00539655041374899)	0	0	0
RP11-96D1.8	0	3.41683748004152 (0.00526413748098987)	0	0	0	0	0	0	0	0
RP11-96K19.2	0	0	0	0	5.09673676539743 (0.000537662285473397)	0	0	0	0	0
RP11-981G7.6	0	0	0	0	0	4.11585911075431 (0.00325125374393998)	0	0	0	0
RP11-982M15.8	0	0	0	0	6.8079187913429 (0.000711023180093009)	7.27410109049146 (0.000760219055333255)	0	0	0	0
RP11-989E6.11	0	0	0	0	0	6.28293043341668 (0.00114601734092013)	0	0	0	0
RP13-104F24.3	-4.49015041951094 (0.00450625167731445)	0	0	0	0	0	0	0	0	0
RP13-122B23.8	0	0	0	0	0	5.20522850772392 (0.00974597258667192)	0	0	0	0
RP13-20L14.10	0	0	-3.03040789827049 (0.00394984272531568)	0	0	0	0	0	-3.00567193371734 (7.35586343729276e-05)	0
RP13-379O24.2	0	0	0	0	0	7.05013890158448 (0.00719954120809021)	0	0	0	0
RP13-39P12.2	0	0	0	0	0	0	0	0	3.49638189934064 (0.00765457631436347)	0
RP13-39P12.3	0	0	0	0	0	0	0	0	3.52890023768367 (0.00271413711327498)	0
RP13-467H17.1	0	0	0	0	0	6.38968800314438 (0.00475066945748178)	0	0	0	0
RP13-487C10.1	0	0	0	0	6.56631610201241 (0.000751259470203347)	0	0	0	0	0
RP13-516M14.2	0	0	0	0	6.47559966567641 (0.0042482603748159)	0	0	0	0	0
RP13-539F13.3	0	0	0	0	0	6.37207729201025 (0.00470337214054864)	0	0	0	0
RP13-580B18.4	0	0	0	0	0	6.9776499682496 (1.61675328669053e-05)	0	0	0	0
RP2	0	0	-2.01323694209091 (0.00097464109772418)	0	-4.48080964677851 (0.00139948264635945)	0	0	0	0	0
RP3-323A16.1	0	0	0	0	6.78391817471601 (4.46060982513049e-08)	7.74545363996337 (9.69936039240869e-09)	4.87522068336408 (0.00277527845060873)	0	0	0
RP3-323N1.2	0	0	-3.33679129999811 (0.00719614934496445)	0	0	0	0	0	-2.54514663768949 (0.00305156063805333)	0
RP3-329A5.8	0	0	0	0	3.58110203726517 (0.00732680373802334)	3.63771190679023 (0.00407781579008946)	0	0	0	0
RP3-331H24.7	0	0	0	0	0	0	0	0	2.46086504564399 (0.000972254046976664)	0
RP3-332B22.1	0	0	4.54473755807954 (0.00726751046259391)	0	0	0	0	0	0	0
RP3-333B15.4	0	0	0	0	0	7.05645578158446 (0.00152284467383944)	0	0	0	0
RP3-366N23.4	0	0	0	0	0	6.62972050443079 (0.00300730370188095)	0	0	0	0
RP3-395M20.8	0	0	0	0	0	0	0	0	-2.7743618768196 (0.00580625890214413)	0
RP3-405J10.3	-2.09288016616901 (0.0080439189731)	0	0	0	0	0	0	0	0	0
RP3-412A9.17	0	0	0	0	5.88816019370094 (0.0024041284503929)	8.15239935646577 (5.52913209630876e-05)	5.83033143714837 (0.00268165016177568)	0	0	0
RP3-416H24.1	-6.21003267531934 (3.71438123951869e-06)	0	-4.41914071676485 (0.000330560214778358)	0	0	0	0	0	0	-6.33375804652463 (0.00888848640111052)
RP3-425C14.5	0	0	0	0	0	5.29719148374998 (0.00200632575204598)	0	0	0	0
RP3-442L6.4	0	0	0	0	0	0	0	6.61455301937216 (0.00847819931613035)	0	0
RP3-467N11.1	0	7.29072813853802 (1.71540124499401e-06)	0	0	0	0	0	0	3.15987102705163 (0.00309184810985239)	0
RP3-468B3.2	0	0	0	0	0	7.46340627048653 (2.00699026482596e-05)	0	0	0	0
RP3-508I15.9	0	0	0	0	4.09190241261928 (0.00179753481908923)	0	0	0	0	0
RP3-512B11.3	0	0	4.25366266169728 (0.00523488181195977)	0	0	0	0	0	0	0
RP3-522J7.7	0	0	0	0	0	3.59369801600199 (0.00307671946768428)	0	0	0	0
RP3-523K23.2	0	0	0	0	0	0	0	0	4.1547785521049 (2.79136441620557e-06)	0
RP3-527G5.1	0	0	5.80449438394224 (0.000570359839533273)	0	0	0	0	0	0	0
RP4-530I15.9	-3.24057533799427 (1.26977337623448e-05)	0	-2.19689657458251 (0.00383637155305501)	0	0	0	0	0	-1.79852585933294 (9.1634646708221e-05)	0
RP4-535B20.1	0	0	0	0	5.41377506176406 (0.00167230267109663)	0	0	0	0	0
RP4-561L24.3	0	0	4.56242136901108 (3.84743107633155e-06)	0	0	3.90572465622236 (0.00244068205272691)	0	0	0	0
RP4-565E6.1	0	0	0	0	0	5.95892355776199 (0.00297419516070928)	0	0	0	0
RP4-568C11.4	-4.40890551189053 (0.00735759890472045)	0	0	0	0	0	0	0	0	0
RP4-610C12.4	0	0	0	0	0	7.9224813778533 (0.00210535674458453)	0	0	0	0
RP4-613B23.1	0	0	3.63756192621671 (0.00284325943126029)	0	0	0	0	0	0	0
RP4-647J21.1	-4.20887831299814 (0.00305518328046926)	0	0	0	0	0	0	0	0	0
RP4-665J23.1	0	0	0	0	0	4.79259188944414 (0.000862186565074329)	3.67202835292563 (0.00699207780401359)	0	0	0
RP4-669K10.8	0	0	0	0	0	5.29716955511337 (0.00310523698463452)	0	0	0	0
RP4-671O14.7	0	0	0	0	5.89915484312426 (0.00353673405368062)	7.38821618524173 (8.72098977042314e-05)	5.94708274814657 (0.000296815649884176)	0	0	0
RP4-695O20.1	0	0	0	0	0	4.29768052399366 (0.0028640587239043)	0	0	0	0
RP4-737E23.2	0	0	0	0	0	0	0	0	0	6.79009821217895 (0.0026785245388961)
RP4-738P15.1	0	0	0	0	0	6.93037023825997 (0.00993412460458525)	0	0	0	0
RP4-742C19.13	0	5.22353385161696 (0.000228831544368797)	0	0	0	0	0	0	0	0
RP4-742J24.2	0	0	0	0	0	0	0	0	2.33889313391406 (0.00177289238902378)	0
RP4-758J18.13	0	0	0	0	0	3.17732775533694 (0.00197440247929669)	0	0	0	0
RP4-758J18.2	0	0	0	0	0	2.61803822358061 (0.00118540572589188)	0	0	0	0
RP4-764D2.1	0	0	0	0	0	6.62972050443079 (0.00300730370188095)	0	0	0	0
RP4-791C19.1	0	0	0	0	0	6.1789715151742 (0.000433945404723294)	0	0	0	0
RP4-798P15.3	0	0	0	0	0	3.51405735264241 (0.00341362065623266)	0	0	0	0
RP5-1007F24.1	0	0	0	0	0	5.74350252804003 (0.00350070913680238)	0	0	0	0
RP5-1014D13.2	0	0	0	0	1.93088713662605 (0.00965963680790087)	2.8531190274368 (0.000592395245835049)	0	0	0	0
RP5-1028K7.2	0	0	0	0	0	0	0	0	5.46025640291583 (6.99195312321655e-06)	0
RP5-1039K5.19	0	0	0	0	2.43016518721962 (0.000439909340874392)	4.04101005000073 (9.3505347645353e-06)	2.39920235540211 (0.00762481317855066)	0	0	0
RP5-1074L1.4	0	0	0	0	0	0	0	2.52940184144934 (0.00406802563551527)	0	0
RP5-1086K13.1	0	0	0	0	0	0	3.17347460053771 (0.00835074002121085)	0	0	0
RP5-1096D14.6	0	0	6.44376333234729 (4.37162415152381e-06)	0	0	0	0	0	0	0
RP5-1103B4.3	0	0	0	0	0	5.50894165838026 (0.00441014816172659)	0	0	0	0
RP5-1112D6.4	0	0	0	0	0	0	0	0	2.4130354051682 (0.00512050694369024)	0
RP5-1112D6.7	0	0	0	0	0	0	0	0	2.05662162405025 (0.00301896547031587)	0
RP5-1119A7.11	0	0	0	0	6.65447770053204 (0.00254565647500638)	0	0	0	0	0
RP5-1121H13.1	0	0	0	0	0	7.37291534030357 (0.00497391000732472)	0	0	0	0
RP5-1125A11.7	0	0	0	0	0	5.82973500658417 (0.00107680734563539)	0	0	0	0
RP5-1139B12.2	0	0	2.75459655198393	0	0	0	0	0	0	0

			(0.00409335588778564)							
RP5-1142A6.10	0	0	0	0	0	4.50280549960002 (0.00465773376471187)	0	0	0	0
RP5-1142A6.2	0	0	0	0	0	3.99319687066938 (0.00522394799827224)	0	0	0	0
RP5-1159O4.2	0	0	0	0	0	0	0	0	3.64272191320738 (0.00500776694791063)	0
RP5-1171I10.5	0	0	0	0	0	0	0	3.77830102275893 (6.56102726967897e-06)	-2.1408479127795 (0.00931341608022177)	0
RP5-1182A14.5	0	0	0	0	0	7.07801654920983 (3.4169261630691e-05)	0	0	0	0
RP5-1198O20.4	0	0	0	0	0	0	0	0	-4.98465334647257 (4.79236752260071e-05)	0
RP5-832C2.5	0	0	0	0	0	7.39204022818938 (0.00411240670568568)	0	0	0	0
RP5-857K21.6	0	0	0	0	2.9229317413945 (0.00603388372214569)	2.62428323659337 (0.00318682950144882)	0	0	0	0
RP5-865N13.1	0	0	0	0	6.24434293510046 (0.0075784156376657)	0	0	0	0	0
RP5-882C2.2	0	0	0	0	0	0	0	0	3.67313454731015 (0.00244341813058511)	0
RP5-896L10.1	0	0	0	0	0	7.00262869924828 (0.00113604594059767)	0	0	0	0
RP5-908M14.10	0	0	0	0	0	5.72551134076583 (0.000341485179669335)	0	0	0	0
RP5-966M1.6	0	0	0	0	0	5.78088093551734 (3.64281852790756e-08)	0	0	0	0
RP5-981L23.2	0	0	0	0	0	4.78718505024292 (0.00788061640124714)	0	0	0	0
RP5-983L19.2	0	0	0	0	0	7.20898483477395 (0.00651838541340656)	0	0	0	0
RP5-991O23.1	0	0	0	0	0	7.88379747174476 (0.000329466227742762)	0	0	0	0
RP6-159A1.4	0	0	0	0	0	0	0	4.22263803627391 (7.97408727087454e-05)	0	0
RP6-91H8.5	0	0	0	0	0	6.86359550693837 (0.000780693116241573)	0	0	0	0
RPA3	0	0	2.99972090750099 (0.000552360688125001)	0	0	0	1.9115359588754 (0.000138446207279599)	0	0	0
RPGR	0	0	0	0	0	0	0	0	-2.20138105128736 (3.64821914985875e-07)	0
RPH3A	0	0	0	0	4.58681327929358 (0.00263517419251996)	5.53894192961761 (0.000610174485508617)	4.2696964690868 (0.00726852077985664)	0	0	0
RPL13AP5	0	2.55383576807744 (0.00400149512339022)	0	0	0	0	0	0	0	1.99643219571648 (0.00524941202702124)
RPL13AP6	0	0	0	0	0	5.06311520538501 (0.00474242679850936)	0	0	0	0
RPL23A	0	1.76357075643386 (0.00801242826851232)	0	0	0	0	0	0	0	0
RPL23AP23	0	0	0	0	0	0	0	0	-3.11504922757339 (0.00823593575835846)	0
RPL23AP82	2.26357041905753 (0.00153449079877304)	0	0	0	0	0	1.67052023268823 (0.00876411402085347)	0	0	0
RPL26P19	0	5.83883113912215 (5.93866736056923e-06)	0	0	0	0	0	0	0	0
RPL28	0	1.6332707810322 (0.00876911207194011)	0	0	0	0	0	0	0	0
RPL34P18	0	0	0	0	0	4.71162644548392 (0.00176603667254364)	0	0	0	0
RPL3L	0	0	9.29876955431793 (5.00274382542502e-20)	0	0	0	0	0	0	0
RPL4P1	0	0	0	0	0	5.54411384885551 (0.00735339058806829)	0	0	0	0
RPL4P4	0	4.15158150563736 (0.00129637126222791)	0	0	0	0	0	0	0	0
RPL5P4	0	3.9091503901294 (0.00366891141898489)	0	0	0	0	0	0	0	0
RPL7P7	0	0	0	0	4.39432539322049 (0.00694699022800642)	0	0	0	0	0
RPL9P16	0	0	3.43994033140878 (0.00351995827209178)	0	0	0	0	0	0	0
RPLP0P6	0	4.73673084607531 (1.61706749966124e-05)	0	0	0	0	0	0	0	0
RPRD1B	0	0	0	0	0	1.83207036610063 (0.00119277056359677)	0	0	0	0
RPS23P8	0	7.12774282209534 (1.25829545491615e-08)	0	0	0	0	0	0	0	0
RPS28	0	2.86901042303822 (0.00103124877316525)	0	0	0	0	0	0	0	0
RPS2P46	0	5.98949718996723 (0.00170445757775897)	0	0	0	0	0	0	0	0
RPS3P7	0	0	0	0	4.01488411198852 (0.0081603836173394)	0	0	0	0	0
RPS6KA1	-2.7376633605034 (3.18050579098304e-07)	0	-3.07278919224002 (4.44159226663782e-08)	0	0	0	0	0	-1.90593652010083 (2.56115394751529e-05)	0
RPS6KA2	0	0	0	0	-1.6759167935382 (5.48482550969088e-05)	-2.34984410350673 (7.14203522851434e-06)	0	-3.44172809229184 (6.13525689603375e-06)	-2.2726825841476 (4.51289354036035e-15)	0
RPS6KA5	3.14294857356475 (1.79088141350494e-07)	0	0	0	0	0	0	0	2.07138335495712 (0.000943777155585242)	0
RPS8P6	0	0	0	0	0	7.29560814722627 (0.001219361098364)	0	0	0	0
RPTN	0	0	0	0	0	6.61636308730497 (0.001289600763607)	0	0	0	0
RRAD	0	0	3.04470199223724 (3.45968326757245e-06)	0	0	0	0	0	0	0
RRAS2	0	0	0	0	-3.53784427013059 (0.00318294014331583)	0	0	0	0	0
RRBP1	0	0	0	0	0	0	0	-1.534204730381 (0.00647344597659928)	0	0
RRM1	0	0	0	0	0	0	0	2.44419695960293 (0.000525505080554924)	0	0
RRM2	-2.98501704940933 (0.00165616694935253)	0	0	-7.12531246622812 (0.00320405310179231)	0	0	0	3.09687994270921 (0.00964747558952882)	0	0
RRM2B	0	0	-2.06266388025115 (1.10960526185262e-05)	0	-2.18647365973317 (0.00435640670252871)	-1.9505514534994 (0.000260964887765555)	0	-2.75060955242465 (0.00152740355907768)	0	0
RRN3P3	0	4.22045627695079 (0.000290602284456989)	0	0	0	0	0	0	0	0
RRS1	0	0	0	0	0	0	0	0	-1.97366158595876 (0.000247345493366304)	0
RSAD2	0	0	0	-7.57647194645926 (0.00156023455937849)	0	0	0	0	0	0
RSPH1	0	0	-4.34472790185883 (0.00555710440022061)	0	0	0	0	0	-5.37374304821419 (5.7043158561619e-05)	0
RSPH6A	0	0	0	0	0	5.05755735890529 (0.0052518271302317)	0	0	0	0
RSPO1	3.53656160521482 (0.000157433832597729)	0	0	0	0	0	0	0	0	0
RSPO4	-4.8830727985805 (3.85670418991531e-05)	0	-4.63021988198341 (4.77996745425349e-05)	-6.16201242489275 (0.0087749111209434)	0	0	0	0	-4.41526810062286 (9.34233841711157e-06)	0
RTKN	0	0	0	0	0	2.91858954919907 (0.000123674582749418)	0	0	0	0
RTKN2	-3.030616256211 (0.00864741239487182)	0	0	0	0	0	2.78197687284289 (9.88784301835687e-05)	0	0	0
RTL1	0	0	0	0	0	5.28094645238944 (0.00106124970948609)	0	0	5.42922657106985 (4.87422191091234e-09)	0
RTN1	2.24491950497613 (0.00514930852529798)	0	0	0	0	0	0	0	0	0
RTN4	0	0	0	0	0	0	0	-1.72038619331466 (0.0066379647878916)	0	0
RTN4RL2	0	0	0	0	0	6.06957392991395 (0.000959283333534274)	0	0	0	0
RTP4	0	0	0	0	0	0	2.71310626954779 (0.00249611620809706)	0	0	0
RUFY3	0	0	0	0	0	0	0	-2.48914176277951 (0.00392285128588094)	0	0
RUNDC3A-AS1	0	0	0	0	5.82417227455558 (0.00528756889243704)	0	0	0	0	0
RUNDC3B	0	0	0	0	0	0	4.1393146146637 (0.00719754889117391)	0	0	0
RUNX1	0	0	-2.10958654894866 (2.60711783709032e-05)	0	0	0	0	0	0	0
RUNX1T1	0	0	0	0	0	0	0	-6.99727010737304 (0.00151966955406871)	0	0
RUNX2	-1.6871031881685 (0.00461217366523509)	0	-2.23820978578491 (0.00167067977800476)	0	0	0	0	0	0	0
RUNX3	0	0	-3.25807521203141 (0.00174837688379917)	0	0	0	0	0	0	0
RUSC1-AS1	0	0	0	-6.12246540998209 (0.00789509081177802)	0	0	0	0	0	0
RUVBL2	0	2.74768570768656 (0.00977165382309157)	0	0	0	0	0	0	0	0

RXFP1	0	0	0	0	0	4.4690050710579 (0.00582869329310032)	2.94152744333043 (0.00966939489203487)	0	5.51844987629924 (0.00126637271974683)	0
RXRA	0	0	0	0	0	1.85193653561607 (1.08220757038602e-07)	0	0	0	0
RXRG	0	0	4.41074588851232 (0.00820263557702032)	0	0	0	3.51896039091495 (0.00408126043242556)	0	0	0
RYR2	0	0	5.93341680817 (1.03126169331514e-20)	0	0	0	0	0	0	0
RYR3	3.45308918898717 (5.74778848145857e-08)	0	0	5.10810845981411 (2.01942411588108e-06)	0	0	0	3.91547715784248 (0.0047334844385173)	0	0
S	0	0	0	0	0	0	24.709358272992 (8.64750968195323e-30)	0	0	0
S100A1	0	0	6.58043112315399 (1.02758645186091e-17)	0	0	4.24245802122919 (0.00547376063395791)	0	0	0	0
S100A10	0	0	0	0	0	0	0	-2.99370963931363 (0.000469412047695799)	0	0
S100A12	0	0	0	-7.15135028963599 (0.0033928378046016)	0	0	0	0	-4.38902668068074 (2.26197706769599e-06)	0
S100A14	-4.43283174937666 (0.00204256821037789)	0	-5.27784057728633 (6.85402077342407e-06)	0	0	0	0	0	-3.47689967906371 (0.00756378848542552)	0
S100A2	0	0	0	0	0	0	4.17634383440775 (0.00171406692457533)	0	0	0
S100A7	0	0	0	0	0	0	0	0	0	10.6280615755718 (5.29616680149257e-18)
S100A8	0	0	0	0	0	0	0	3.69973757340697 (9.28047187021288e-05)	-3.37913329834084 (6.45330969428626e-10)	0
S100A9	0	0	0	0	0	0	0	3.33093329160055 (0.000209826996712318)	-2.29640084137439 (0.000110597210748919)	0
S100B	3.90402837087893 (8.14095489576251e-06)	0	0	4.33335379230409 (0.00501950580025702)	0	0	0	0	0	0
S100P	-3.9088408660269 (0.00604764571400781)	0	0	0	0	0	0	0	3.38267896617821 (1.2948452635211e-09)	0
S1PR1	-1.79289511136223 (4.44221183188778e-05)	0	0	0	-1.90495529358158 (0.00218912517718988)	-2.75571264939643 (8.83759172444443e-05)	0	-5.00870543384066 (5.73851221824029e-05)	-1.75161873138913 (0.000533354682697499)	0
S1PR2	-1.52049382091032 (0.00113456591593125)	0	0	0	0	0	0	0	0	0
S1PR3	1.68961790569379 (0.000465480740559297)	0	0	0	1.94308873920031 (0.001632486994601)	0	0	0	0	0
S1PR4	0	0	0	0	0	0	2.72623445140293 (0.00289254127409786)	0	0	0
SAA1	0	0	-3.6906550709306 (0.000153498479164886)	0	0	6.6696793137577 (2.6349984782666e-17)	0	0	-3.39265362840144 (3.13076507120238e-05)	0
SAA2	0	0	0	0	0	7.93800937038421 (3.24518169523324e-17)	0	0	-3.95748020300886 (0.00265235307633177)	0
SAA2-SAA4	0	0	0	0	0	6.58529443984317 (8.47830076726333e-08)	0	0	0	0
SAG	0	0	0	0	0	7.56725781506949 (1.62365044165595e-05)	0	0	0	0
SALL1	0	0	0	0	7.84058562903366 (1.0644447126224e-06)	7.68060289478117 (1.18322138644463e-07)	0	0	6.1604413771122 (5.36318680098426e-08)	0
SALL2	2.70740522504061 (5.80001623091519e-06)	0	0	0	0	0	0	0	0	0
SALL3	0	0	0	0	6.18525214064251 (0.00134988512834523)	0	0	0	0	0
SALL4	0	0	0	0	0	7.30792734894297 (2.01148495093208e-05)	0	0	0	0
SAMD12	0	0	0	0	0	0	0	0	-2.72461550063473 (1.94030797869831e-06)	0
SAMD4A	0	0	1.82051275015322 (1.42680858642188e-06)	1.8627424380894 (0.0035859838313169)	0	0	0	-2.87596008835158 (0.00133086006137124)	0	0
SAMD5	0	0	-3.87205978676028 (0.000143856284027222)	0	2.52001181080034 (0.00388803337937156)	0	0	0	0	0
SAMD8	0	0	0	0	0	0	0	-2.38088348065975 (0.00726487837186787)	0	0
SAMD9	0	0	0	0	0	0	0	0	2.10525361596803 (0.000295161562387318)	0
SAMD9L	0	0	0	-2.54046626728074 (0.00459717852588402)	0	0	0	0	1.74364825872149 (0.00134564432274294)	0
SAMHD1	0	-5.97374557614082 (0.00544989898817339)	0	0	0	0	0	0	0	0
SAMSN1	-2.85294859963827 (1.07215724819022e-05)	0	-3.70458170498621 (1.53391787261836e-07)	-3.55437823410772 (0.00108246639456132)	-2.94439772346722 (0.00446688399775022)	0	0	0	-1.80209740439058 (0.000872614560985059)	0
SARDH	0	0	0	0	0	3.82840576028172 (1.88635084166467e-09)	0	0	0	0
SASH1	0	0	0	0	0	-1.81467008265586 (5.76791429785489e-05)	0	-3.44677832388692 (2.63829506504324e-05)	0	0
SASH3	-2.17699050811748 (0.000322083065611657)	0	-2.18334897831413 (0.0022398111564898)	0	0	0	0	0	0	0
SASS6	0	0	0	0	0	0	0	3.1145788702946 (0.00608001045916124)	0	0
SAT1	-2.28129577034924 (1.65512592628445e-05)	-3.00369182754298 (0.000494638210061645)	-2.30412754121924 (0.000287393428618981)	-2.215979527721 (0.000272468251150337)	-1.79054601649812 (0.00261788273225239)	0	0	0	0	0
SATB1	0	0	0	0	0	0	0	0	-2.00768827366888 (1.48918509179929e-05)	0
SATB1-AS1	0	0	0	0	0	4.87561497536915 (0.00586630737010186)	0	0	0	0
SATB2	0	0	0	0	0	2.38825139499333 (0.00257143689497919)	0	0	0	0
SATL1	0	0	0	0	0	7.26986837985775 (0.00668205493536917)	0	0	0	0
SBDS	0	0	0	0	0	0	0	-2.87023071685586 (0.00186677724080688)	0	0
SBF1P1	0	0	0	0	0	6.17403800600322 (0.00788201947031849)	0	0	0	0
SBK1	0	0	0	0	0	4.28413894619818 (0.00445898818719534)	0	0	0	0
SBK3	0	0	3.75508666505053 (0.00810159068428644)	0	0	0	0	0	0	0
SBSN	0	8.83438846949353 (1.89611992709485e-06)	0	0	0	0	0	0	0	12.859433054025 (3.47073796071121e-29)
SBSPON	0	0	0	0	0	0	0	0	2.10420084206931 (0.000105728572932642)	0
SC22CB-109E1.1	0	0	0	0	5.57856130299113 (0.00693597022972821)	6.94536535564673 (0.000314778291643124)	0	0	0	0
SC5D	0	0	-1.53730251591102 (0.00797171573873393)	0	0	0	0	0	0	0
SCAF1	0	0	1.68968233371615 (0.00270673861229093)	0	2.56052746779686 (0.000396131875389 91)	0	0	0	0	0
SCAMP1	0	0	0	0	0	0	0	-6.59977756088315 (0.00237262513525655)	0	0
SCAMP5	3.31992862371782 (0.00170205918903045)	0	0	0	0	0	2.80459256410773 (0.00875915625768195)	0	3.07622779499357 (0.000320280142049149)	0
SCARA3	0	0	-2.14632472383742 (0.00315523951883375)	0	0	0	0	-6.14748781400341 (0.00601235120941556)	0	0
SCARA5	2.55056947826924 (1.73078409085077e-07)	0	0	0	0	0	0	0	0	0
SCARB1	0	0	0	0	0	0	0	0	1.87488997663729 (0.00305789131896316)	0
SCARB2	0	-6.64669898164123 (0.00199460732575468)	0	0	0	0	0	-3.33065110553803 (1.61905511419305e-05)	0	0
SCARF1	0	0	0	0	0	0	0	0	-1.82305415531884 (0.000643714368590364)	0
SCARF2	0	0	0	0	0	0	0	0	-3.05324073972978 (3.91069586573073e-05)	0
SCARNA1	0	0	0	0	0	0	0	0	1.61840937044629 (0.00522915431515879)	0
SCARNA10	0	-5.87098990785564 (0.00644217481762452)	0	0	0	0	0	0	0	0
SCARNA11	0	0	0	0	0	3.78067275871291 (0.00969280019181676)	0	0	0	0
SCARNA20	0	0	0	0	0	4.49352884700863 (0.00170594924064671)	2.6923259205096 (0.00112513146531477)	0	0	0
SCARNA21	1.53225257431733 (0.000658096566101292)	0	0	0	0	0	0	0	0	0
SCARNA6	1.75011401689273 (9.01599097995186e-07)	0	0	1.98479981193967 (0.00798721529016583)	0	0	0	0	0	0
SCART1	0	0	0	0	0	3.8785179156689 (0.000202808130888626)	0	0	0	0
SCCPDH	0	0	0	0	0	2.14608966787155 (0.0054443620447852)	0	0	1.52989669716783 (5.23329408489354e-05)	0
SCD	-2.29647700508878 (0.000484516699656472)	0	0	-3.02736604196957 (0.00362648434306615)	0	0	0	0	-1.92804213073586 (0.00197682800562103)	0
SCD5	1.78328620518851 (0.00120928013620608)	0	0	0	0	0	0	0	0	0
SCEL	-6.34753368402707 (1.33429165991243e-11)	0	-6.55233990718866 (1.45700782135091e-13)	-5.6629747361656 (0.000544419041078 59)	-5.1682785210061 (0.000102921215238504)	-4.70375928704125 (5.61528333212727e-06)	0	-7.0475861322398 (0.00130450881155542)	0	0
SCG2	2.67785000546813 (0.00247154273584114)	0	0	0	0	0	0	0	0	0
SCG3	5.24460886617188 (3.71676897320882e-05)	0	0	0	0	0	0	0	0	0
SCGB1A1	-7.66597847651586	0	-5.47514839500604	0	-5.31652906566788	-4.79684708056852	0	0	-8.43749839996886	0

	(3.57208583052884e-05)		(0.000619324763602259)		(0.00338447841078937)	(0.00291856371651739)			(1.81355130151895e-06)	
SCGB1D2	0	0	0	0	0	0	0	0	0	6.71609651231352 (0.00360897601821362)
SCGB2A2	0	0	0	0	0	0	0	0	0	8.87757818034948 (1.06524343508979e-09)
SCGB3A1	-6.52948905193237 (6.10707004406404e-07)	0	-4.7524732563043 (6.11365166507463e-05)	-6.35775406326221 (0.00754108961043537)	0	0	0	0	-6.55780971456667 (3.61132190492813e-09)	0
SCGB3A2	-7.29218922703058 (9.60200164277947e-05)	0	-5.01574861782608 (0.000206652016480122)	0	0	0	0	0	-6.51618213699608 (2.36160798848679e-05)	0
SCGN	4.28609807845952 (0.00593115171461596)	0	0	0	0	0	0	0	0	0
SCHIP1	0	0	2.31606438904628 (0.00150138747945815)	3.37983679944342 (0.0029891504876375)	0	2.8797581181301 (0.00472849234211291)	0	0	0	0
SCIN	0	0	-4.02893624135025 (0.000570594067998296)	0	0	0	0	0	2.78201953242112 (0.00399509041933375)	0
SCML2	2.89864265518607 (0.0082938699736702)	0	0	0	0	0	0	0	0	0
SCN11A	5.55828075466684 (1.4986653973461e-05)	0	0	0	5.83005063945246 (0.00374446131635038)	7.71042631094124 (1.64911680264834e-06)	0	0	3.95595807880071 (0.00179726732677155)	0
SCN1A	-2.67051982275507 (0.00561610293658235)	0	-3.80290392785469 (0.00120401822479091)	0	0	0	0	0	-4.44190746105175 (5.06337412700673e-06)	0
SCN1B	0	0	2.14479467554119 (0.00047412885834898)	0	0	0	0	0	0	0
SCN2A	0	0	0	0	4.98489605035605 (1.19978289426057e-05)	5.5079493482176 (0.000503432784075141)	0	0	0	0
SCN2B	0	0	4.13849755060586 (0.000184301669439137)	0	0	0	0	0	0	0
SCN3A	2.78786236360842 (0.00472693674247527)	0	0	0	6.55366470342981 (3.91469867515775e-13)	4.91157792500885 (0.000265795536856545)	0	0	0	0
SCN3B	2.58177416485765 (0.00380353025669461)	0	0	0	0	0	0	0	0	0
SCN4A	0	0	0	0	5.87588891730455 (3.41697724056943e-05)	5.4421827931004 (0.00813523910838199)	0	0	0	0
SCN5A	0	0	8.15626766126999 (2.23052852023204e-23)	0	0	5.19750539450815 (8.87804050807273e-05)	0	0	0	0
SCN7A	0	0	0	0	-2.55784440126383 (0.00602406591715242)	0	0	-8.04760105181222 (0.000304759965264385)	0	-4.9436012844357 (1.26696569128451e-05)
SCN8A	0	0	0	0	6.10124362972273 (0.000102142027176421)	5.95336338713915 (7.55927845601698e-07)	4.8444900567152 (0.000709826795071346)	0	0	0
SCN9A	2.1690761857439 (6.41098011530657e-05)	0	0	0	0	1.9964881978476 (0.0041128801183111)	0	0	1.98068988132124 (0.00111360390804793)	0
SCNN1A	-5.59101879240338 (4.11613149607303e-14)	0	-5.97753065191583 (2.9784653112937e-13)	-6.38899695891371 (0.000140558840963142)	0	0	0	-7.77279351499414 (0.000468906384314314)	-4.5677333357848 (6.95340519267311e-13)	0
SCNN1B	0	4.01599910443685 (0.000952490242090163)	-3.28652993076036 (0.00870853031846877)	0	0	0	0	0	0	0
SCNN1G	-4.79502920021897 (0.00141880710591272)	0	0	0	3.28022720201919 (0.00189781264052161)	0	0	0	0	0
SCPEP1	0	0	0	0	0	0	0	0	-1.62861815867367 (0.0051371797847773)	0
SCUBE1	0	0	-2.98772506013562 (0.00337984838551498)	0	0	0	0	0	0	0
SCUBE2	2.57721050642829 (0.001335675397785)	0	0	0	0	0	0	0	2.97348287732625 (2.27004167787987e-05)	0
SCUBE3	1.67264743543085 (0.00428839417372744)	0	0	0	0	0	0	0	0	0
SCYL3	0	0	0	0	0	0	0	0	1.6433381821406 (0.000158959785394502)	0
SDC1	-4.83920430949557 (1.24471448042384e-11)	0	0	-6.08438543940682 (0.000359884912503668)	0	2.96559717941957 (0.000250931752060413)	0	0	0	0
SDC2	0	2.33529257788105 (0.00617489606652642)	0	0	0	0	0	-4.82102069038502 (0.00353023926145183)	0	0
SDC4	0	0	0	0	0	0	0	-6.15648296814207 (7.96020448302425e-06)	0	0
SDCBP	0	0	-1.66285762251947 (0.00037077004511568)	0	0	0	0	0	0	0
SDCBP2	0	0	0	0	0	0	0	0	-2.37640242068057 (0.00587499474948269)	0
SDCBPP1	0	0	0	0	0	0	0	0	-4.66695976300873 (0.00180819872929)	0
SDF2L1	0	0	0	0	0	0	0	0	-3.07354566402481 (0.00340058364643699)	0
SDHA	0	0	1.82956269711106 (7.81686175383195e-07)	0	0	0	0	0	0	0
SDHB	0	0	0	0	0	1.69779053012329 (0.00285960048071472)	0	0	0	0
SDHD	0	0	0	0	0	1.83194363482024 (0.00150616327702061)	0	0	0	0
SDK2	-3.47818624983804 (7.90335893259737e-09)	0	-2.01955770541453 (0.00133067879747422)	0	0	0	0	0	-2.85246959005352 (3.10893320040864e-06)	0
SDPR	0	0	0	0	0	-1.71784729717027 (0.00104056563833176)	0	0	-1.57626932529867 (0.000131811068613572)	-2.40410672650325 (0.000233350438001707)
SDR16C5	-7.34397963748767 (7.62943039675385e-10)	0	-6.35524711578033 (2.28441452882456e-10)	-7.90310323098436 (0.000489334236090955)	-3.39580412155041 (0.00594502381090702)	0	0	-6.84515475318045 (0.00262394798580109)	-8.1046067908235 (2.58320500257146e-16)	0
SDR42E1	0	0	0	0	0	4.1619262689947 (0.000111985172785356)	2.84166158429845 (0.00384209522322828)	0	0	0
SDS	0	0	0	0	0	5.58049357454752 (5.22733160662311e-09)	0	0	0	0
SEC14L1	-1.84902502170396 (1.79814973063764e-09)	0	0	0	0	-1.76670912372811 (4.86089778006894e-06)	0	0	0	0
SEC14L2	0	0	0	0	0	3.21636791741093 (0.00011127608461451)	0	0	0	0
SEC14L4	0	0	0	0	0	4.31190508189114 (0.000314596732409678)	0	0	0	0
SEC14L5	0	0	5.11158191510021 (0.00269065456585473)	0	0	0	5.52135415764551 (0.00417316655728942)	0	0	0
SEC14L6	0	0	0	0	0	0	3.30264146167739 (0.00478960348035568)	0	0	0
SEC23A	0	0	0	0	0	0	0	-2.75203597628639 (0.00125289744128075)	0	0
SEC23B	0	0	-1.79663702223567 (5.15819701906219e-05)	0	0	0	0	0	0	0
SEC24D	0	0	0	0	-1.61494043205542 (0.00120051028947188)	0	0	0	0	0
SEL1L3	-2.20494206313499 (0.000116396278255839)	0	-2.2659649696936 (8.3265707020055e-05)	0	0	0	0	0	0	-2.38775896587561 (0.00428307914538805)
SELE	-4.54562503742695 (0.00343645469698811)	0	0	0	0	0	0	0	-4.16507017736111 (0.00164696687527451)	0
SELENBP1	0	0	0	0	0	0	0	0	-2.18960218768412 (0.000864164219738418)	0
SELK	0	0	1.55891426580594 (0.000314560808716332)	0	0	0	0	0	0	0
SELL	-2.21613122609944 (0.00582682654357624)	0	0	0	0	0	0	3.12523337141616 (0.000817244311437017)	0	0
SELM	1.89115503234515 (2.99263434738811e-05)	0	0	0	0	0	0	0	0	0
SELPLG	0	0	-2.24701879900421 (0.000184005061124479)	0	0	0	0	0	0	0
SEMA3A	2.03459008040083 (5.21542830953099e-07)	0	-3.5958536191986 (1.24952083613374e-05)	0	0	0	0	0	0	0
SEMA3B	0	0	0	0	0	0	0	0	3.16842938329279 (1.41791754167614e-05)	0
SEMA3C	1.5752719445337 (0.000127057849456502)	0	0	0	0	-3.31698828585484 (0.00018929988013908)	0	-5.20271547238321 (0.00147626528416896)	0	0
SEMA3E	4.30938500198368 (2.03788782337768e-11)	0	0	3.39025335729884 (0.00886174925566851)	0	3.10033887927896 (0.00721480684140322)	2.22106107266952 (0.0073294769703421)	0	0	0
SEMA3F	-3.47421588381647 (2.5725565914281e-13)	0	0	-2.7788803344784 (0.000737947851614354)	0	0	0	-4.72092303212032 (0.00299798034385091)	-1.52186555628398 (0.00204977682993998)	0
SEMA4C	1.51442479504778 (0.00176071482446496)	0	0	0	0	0	0	0	1.73082813709125 (2.12765979932661e-05)	0
SEMA4G	0	0	0	0	0	4.00818002876583 (0.000124526956862926)	0	0	0	0
SEMA5A	-2.15482658817228 (0.000404039464791469)	0	0	-2.94256204084241 (0.00655460044505991)	0	0	0	-6.91393628641121 (0.00208334891733124)	0	0
SEMA5B	0	0	0	0	0	4.96751079235486 (0.003239604441669)	0	0	3.39513576671689 (0.00100831951397049)	0
SEMA6A	0	0	0	0	0	0	0	0	2.07720500891457 (0.00043502901523047)	0
SEMA6B	0	0	0	0	0	0	0	0	-1.57221136939212 (0.00455766403848479)	0
SEMA6C	0	0	3.77518023630367 (4.08493255692006e-10)	0	0	0	0	0	-2.17405915057653 (0.00713635363656151)	0
SEMA6D	0	0	0	0	0	0	0	0	3.52596684046111 (4.69203744421326e-10)	0
SEMA7A	0	0	0	0	0	0	0	0	2.93998555406969 (1.80143945693674e-09)	0
SEPP1	0	0	0	0	0	0	0	-4.66963282546829 (0.000373284550648829)	0	0
SEPSECS	0	0	0	0	0	2.36617004866499 (0.00228118660224837)	0	0	0	0
SEPT1	0	0	0	0	0	0	1.69181977370969 (0.00873754673025025)	0	0	0

SEPT10	0	0	0	0	0	0	0	-3.58415592480344 (0.00127041446187941)	0	0
SEPT3	0	0	0	0	0	0	0	0	4.67193497350387 (1.23192028410968e-08)	0
SEPT4	0	0	0	0	0	1.82308312616207 (0.00415179942935416)	0	0	0	0
SEPT5	0	0	3.05017462369397 (1.0419738310819e-06)	0	0	0	0	0	0	0
SEPW1	0	0	2.44886635865398 (3.87457950221822e-09)	1.97114768223251 (0.00141627601462568)	0	0	0	-4.69850751414526 (0.00315195498714466)	0	0
SERINC2	-2.34717166327244 (0.00183574175958405)	0	0	0	0	2.91415546581996 (3.10798828876364e-05)	0	0	0	0
SERP2	0	0	3.01880972301379 (0.00567598111952534)	0	4.7769864799632 (0.00751362763840102)	0	0	0	0	0
SERPINA1	-2.64491175725851 (1.03125374059537e-05)	-6.42877164581439 (0.00307817262952218)	-3.23674854948359 (1.38050744762787e-06)	-3.40017196527195 (1.51059574549524e-05)	0	5.34893889413134 (2.08034337695077e-16)	0	0	-3.05437121903733 (1.49792603381574e-08)	-3.76738329296299 (1.82950207157675e-06)
SERPINA10	0	0	0	0	0	8.6712862846755 (1.61985816362596e-11)	0	0	0	0
SERPINA11	0	0	0	0	0	9.61815400986756 (1.11807797628362e-17)	0	0	0	0
SERPINA12	0	0	0	0	0	0	0	0	0	8.49055344589741 (4.02162820916543e-08)
SERPINA3	0	0	0	0	0	4.56472679384601 (1.38444418198246e-13)	0	-5.57994222019677 (0.000388199379604047)	0	0
SERPINA4	0	0	0	0	0	8.40242396454755 (6.84919741590348e-10)	0	0	0	0
SERPINA5	0	0	0	0	0	6.50209896021597 (1.91135062611875e-08)	0	0	0	0
SERPINA6	0	0	0	0	0	9.10933107538411 (3.60011736238952e-13)	0	0	0	0
SERPINA7	0	0	0	0	0	7.48765585953265 (9.47312290012592e-08)	0	0	0	0
SERPINA9	0	0	0	0	0	6.54401974831726 (0.00335150846722125)	0	0	0	0
SERPINB1	0	0	0	0	0	0	0	2.00424706061142 (0.00718463051135032)	-1.57206441028706 (5.53155345409646e-05)	0
SERPINB10	0	0	0	0	0	0	0	5.54276631077599 (0.00110301057694118)	0	0
SERPINB12	0	0	0	0	0	0	0	0	0	7.11203065854564 (0.000618894886710335)
SERPINB13	0	0	0	0	0	6.19087626092151 (0.00808622316855013)	4.6533163147539 (0.00592211602405414)	0	0	6.92396899541144 (0.000128599971054192)
SERPINB5	0	0	0	0	0	0	4.63002767572256 (0.00441657605391403)	0	0	8.4707312123995 (1.16514839444965e-09)
SERPINB7	0	0	0	0	0	0	0	0	4.55132560822172 (0.00345398207454152)	7.27553129623249 (0.000263701103967057)
SERPINB9	-1.60998505121585 (0.00454491610825546)	0	-1.66620000489668 (0.007362813740763)	0	0	0	0	-3.74239216825288 (0.00589270949272209)	0	-3.08028385840098 (0.00242639450187689)
SERPINC1	0	0	0	0	0	10.3871351253728 (2.65688501470811e-17)	0	0	0	0
SERPIND1	0	0	0	0	0	5.8422393205752 (1.90591967860749e-14)	0	0	0	0
SERPINE1	0	0	-1.89581653035165 (0.000504158577106217)	-2.12492757885589 (0.00115550743302971)	-2.10024725468326 (0.00203121687316754)	0	0	-5.606021997358 (7.72930280109424e-12)	0	-2.23155877960157 (0.000680676878674268)
SERPINE2	2.91117914547688 (9.10005918577763e-06)	0	0	2.97525807467891 (0.00248751066465618)	0	0	0	0	5.47815325370768 (1.45028200507257e-31)	0
SERPINF1	0	0	0	0	0	0	0	-5.67785482745799 (0.000618291993406839)	0	0
SERPINF2	0	0	0	0	0	4.73408120812885 (4.26225281426852e-06)	0	0	-4.66806594951471 (6.72632330628497e-05)	0
SERPING1	0	0	0	0	0	0	0	-3.65501482534683 (1.59265970757922e-06)	0	0
SERPINH1	0	0	0	0	0	0	0	-3.71330354263374 (9.43197847292752e-05)	0	0
SERTAD1	-2.09917380688 (0.00408146441380429)	0	0	0	0	0	0	0	0	0
SERTAD4	2.90511973780142 (0.00367230708117303)	0	0	0	0	4.65354881690222 (0.00545523045972918)	0	0	3.20613827277455 (3.852263080299e-05)	0
SERTAD4-AS1	0	0	0	7.44933129808559 (0.000113646117281295)	0	0	3.68326863431615 (0.00192515651347084)	0	0	0
SESN2	-1.85274873126663 (0.00395555723496161)	0	-2.36542139104155 (0.000800755852548728)	0	0	0	0	0	0	0
SESTD1	0	0	0	0	0	-1.9520151970902 (0.000566619103868802)	0	0	0	0
SETBP1	0	0	0	0	0	0	0	-3.20054505260147 (0.00188650104425201)	0	0
SETD7	0	0	0	0	0	0	0	-3.31247077978524 (5.30091784225503e-05)	0	0
SETP21	5.00907803105684 (0.0025152158804131)	0	0	0	6.24434293510046 (0.0075784156376657)	0	0	0	4.26787125887488 (0.00950528102727365)	0
SETSIP	0	0	4.57130970409935 (0.00857596821257909)	0	0	0	0	0	0	0
SEZ6L	4.80207704940678 (0.000909559724406427)	0	0	0	0	7.10908861711052 (0.000342957758976751)	0	0	0	0
SFN	-5.31919205308264 (1.95104025303971e-10)	0	-6.67283765520822 (1.96306918605143e-11)	-5.7920321677734 (0.000889374043013447)	-5.3236372142245 (0.000271638619794335)	0	0	-7.17645550413504 (0.00160180003266294)	0	0
SFRP1	5.89297523413006 (4.21101719862603e-20)	0	3.44602810579938 (1.00732958859304e-05)	5.20946189304261 (7.86846541275093e-07)	3.70423729595575 (0.000333590277372374)	0	0	0	5.16172770594289 (2.43870674194128e-12)	4.1834950108903 (0.000290382968172291)
SFRP2	0	0	-3.93683482525528 (0.00616483386556777)	0	0	0	0	0	-5.75846935214224 (2.91363933298763e-06)	0
SFRP4	0	0	-2.87369854346387 (0.00194029221036446)	-6.07511505301361 (0.00882340488306232)	0	0	0	0	-5.85899417011233 (6.06131111195854e-09)	0
SFRP5	4.06364051545821 (0.00744008174728445)	0	0	6.15508994147554 (0.0047077506279402)	0	0	0	0	0	0
SFTA1P	-5.20245911187824 (1.08748536587736e-06)	0	-5.23704188915436 (2.59774872391366e-07)	-6.81457482790498 (0.00267610285017289)	-3.89290984284297 (0.00814749065491002)	-3.40807876338336 (0.00610317611898528)	0	0	-7.00970177724158 (8.82930569070456e-13)	-7.09708046969553 (0.00175849260547943)
SFTA2	-7.08752824018589 (2.78812880319078e-08)	0	-5.36839933045143 (1.47314854725886e-06)	-6.91491839844203 (0.00315271137023811)	0	0	0	0	-6.71239565461458 (2.23674301646511e-10)	-7.19460757913532 (0.00211550470101405)
SFTA3	-7.44498802101729 (8.22083119471917e-09)	0	-5.72559242539035 (3.44613312046956e-07)	-7.26652097749765 (0.00202356537035581)	0	0	0	-6.21029784674114 (0.00864135090652497)	-7.48408729408316 (5.52114315629347e-12)	-7.54612284000905 (0.00134258517092)
SFTPA1	-9.8365129172141 (1.81986735101265e-17)	-6.57089118357379 (0.00386635799968316)	-11.1839016789117 (3.65136909042645e-21)	-10.0861632752068 (7.68335654802366e-07)	-7.53650598549236 (2.2335171746228e-07)	-9.11555996897449 (5.40837123511617e-15)	0	-12.4702531657239 (7.37431050252931e-06)	-13.7315166808965 (2.8565439176494e-23)	-9.36570860084844 (8.96718626546311e-07)
SFTPA2	-10.2725197305314 (1.20240434423619e-15)	-9.46591572155675 (0.000825295348133278)	-10.1300330457537 (4.27936367410391e-20)	-9.54023359812705 (6.09853861111455e-07)	-9.06815353821009 (1.98754410439546e-13)	-8.53056303331378 (2.31065630884441e-14)	0	-12.9243018289 (4.48399732789744e-06)	-14.1854520333022 (1.29589053952599e-24)	-7.81990269469493 (1.49335471631738e-05)
SFTPB	-9.18376442301129 (4.75659793244938e-41)	-9.01181881640767 (3.98953505222652e-05)	-9.13686319144555 (1.26423878422144e-38)	-7.74966930453513 (1.10952420300443e-16)	-6.76480694053003 (9.99262297358177e-13)	-6.49852603759598 (7.27886380971178e-10)	0	-10.0135089942928 (1.78375162500869e-09)	-13.7147084461016 (2.31045692636474e-50)	-9.35213095455089 (1.00159609075014e-17)
SFTPC	-8.05153175686946 (4.18172709225182e-07)	0	-9.48999331017593 (3.02179542437624e-09)	-11.1139070188874 (0.000319981427018551)	-6.16312092726344 (0.000168115584277732)	-5.47652106901507 (2.29907395944691e-05)	0	-10.0543977807158 (0.00112930021284499)	-10.9064925667721 (1.44820872514437e-13)	-11.3914265738688 (0.000223903238538084)
SFTPD	-6.43583736532199 (8.60593974529748e-08)	0	-5.03081147600901 (3.95918169835807e-07)	-6.98716082087685 (0.00219602605401281)	0	0	0	-5.93215761365674 (0.00965568384708079)	-6.38638357431581 (4.43813668484018e-11)	-4.82389887731198 (0.00611934318445646)
SFXN1	0	0	0	0	0	1.78086808717856 (0.00112446595541078)	0	0	0	0
SFXN2	0	0	0	0	0	2.56038286487163 (0.00608450029311786)	0	0	0	0
SFXN3	0	0	0	0	0	0	0	-2.43717772520741 (0.00384487657938441)	0	0
SFXN4	0	4.51714715890015 (0.000575799099597747)	0	0	0	0	0	0	0	0
SFXN5	0	0	0	0	0	2.54031404080611 (0.000156807429656546)	0	0	0	0
SGCA	0	0	2.71289352228367 (1.40040723032572e-05)	3.07012419942 (0.00938034089199579)	0	0	0	0	0	0
SGCD	0	0	1.9064146248524 (0.000269564212949835)	0	0	0	0	0	0	0
SGCG	0	0	6.79167227011613 (2.52172923926932e-11)	0	0	0	0	0	0	0
SGK1	-3.59173317018285 (4.23953295255997e-13)	0	-1.70285703694865 (1.60288139083869e-06)	-3.24090009628912 (2.46054709268949e-05)	-1.74906111307387 (0.00444296935707783)	0	0	-3.18311735101563 (0.000266032615355464)	0	0
SGK2	0	0	0	0	4.2818057904255 (0.00292694561269577)	5.69308720387449 (1.80569779477835e-08)	0	0	0	0
SGMS1-AS1	0	0	0	0	4.81700700781066 (0.00531188042168803)	5.91456778239453 (2.7498065179313e-05)	4.08171825436043 (0.00264696478107614)	0	0	0
SGMS2	0	0	0	0	0	0	0	-7.24817807558742 (0.000999495639645091)	0	0
SGOL1	0	0	0	0	4.54141315946854 (0.00748829444171019)	4.15068039878085 (0.00843772717175768)	2.6496130848309 (0.0081745389086492)	0	0	0
SGOL2	0	0	0	0	0	0	0	3.56581842452482 (0.000253534933541747)	0	0
SGPP1	0	0	0	0	0	0	0	0	2.07293795657354 (1.14055250807085e-06)	0
SGPP2	-3.72263834079776 (7.27759225810089e-05)	0	0	-6.65207912963493 (0.00450257861986244)	0	0	0	0	-3.62727765316873 (4.33869668984454e-06)	0
SGSH	0	0	-1.69164372868831 (0.000219449665370443)	0	0	0	0	0	0	0
SGSM1	0	0	2.45424239560961 (0.00743286346901016)	0	0	3.6584747853127 (0.0067739010515439)	0	0	0	0
SH2D3A	-3.32185979618699 (0.0011573471549587)	0	0	0	0	0	0	0	0	0
SH2D3C	-2.13925582726666 (0.00120684721536734)	0	0	-3.75910823470543 (0.00705701431207139)	0	0	0	0	-1.56880306570152 (0.00146570959551391)	0
SH2D4A	-2.4403999561243	0	-2.09007679789876	0	0	0	0	0	0	0

	(0.000226852975074315)		(0.00251681672919977)							
SH2D4B	0	0	0	0	0	6.41259433837147 (0.000381218227948887)	0	0	0	0
SH2D6	0	0	0	0	0	6.20391143651473 (0.00503411656664534)	0	0	0	0
SH3BGR	0	0	4.79841982333349 (1.75610661911059e-12)	2.87083965110497 (0.00221378112623672)	0	0	0	0	0	0
SH3BGRL3	0	0	-1.68178629887872 (0.00162222098551081)	0	-2.29139850661931 (0.00522895530797872)	0	0	0	-2.13756218939183 (4.48946135970228e-06)	0
SH3BP4	0	0	0	0	0	0	0	-3.47929742383543 (0.00474246562291281)	0	0
SH3D19	0	0	0	0	-1.64420826474734 (0.00500991177572115)	0	0	-8.93139525865198 (3.49768411209871e-05)	0	0
SH3D21	0	0	-2.50091085700648 (0.00696312069116005)	0	0	0	0	0	0	0
SH3GL3	0	0	0	0	4.62352692450753 (0.00685784650360415)	0	0	0	0	0
SH3GLB2	0	0	0	0	0	0	0	0	1.83773085319835 (0.00287518540554902)	0
SH3KBP1	0	0	0	0	-1.7627350455562 (0.00521051448784816)	0	0	0	0	0
SH3PXD2A	0	0	0	0	0	-1.51754376302166 (0.000549476140924029)	0	-3.01902214626832 (1.43137396876594e-05)	0	0
SH3PXD2B	0	0	-2.61014861438087 (3.65945311459974e-08)	0	-1.72126282088768 (0.00125612854252595)	-2.72679633438849 (2.52958180311491e-05)	0	-3.61353323435852 (2.16889910155028e-05)	0	0
SH3RF2	0	0	4.23397160921083 (7.04603278577141e-09)	0	0	0	0	0	0	0
SH3RF3	0	0	0	0	0	0	0	-6.40020573883489 (0.00423755241783904)	0	0
SH3TC1	-2.11678719934212 (0.000167945318290263)	0	0	0	0	0	0	0	0	0
SH3TC2	0	0	0	0	0	4.29582541088235 (1.89163059109648e-05)	0	0	5.60994681798431 (2.39175265668045e-10)	0
SH3YL1	0	0	0	-6.80684999897312 (0.00215407195170843)	0	0	0	-5.75033752275902 (0.00988371672927114)	0	0
SHANK1	0	0	0	0	0	5.21806709305221 (0.00125509351535163)	0	0	0	0
SHANK2	0	0	-4.18421453591952 (5.19999082581995e-05)	0	0	2.20764956295528 (0.000401845501536362)	0	0	2.22508404852171 (0.000308661443393606)	0
SHANK3	-1.90796108780816 (0.000512312678250951)	0	0	0	0	0	0	0	0	0
SHC4	0	0	0	0	5.66036838610677 (0.00663255844456536)	0	0	0	0	0
SHCBP1	0	0	0	0	0	0	0	4.02615221326346 (0.000327099993466288)	0	0
SHD	0	0	0	0	0	6.36201066526296 (0.00506158730931904)	0	0	0	0
SHE	0	0	0	0	0	0	0	-3.72154418676294 (0.00290682188122424)	0	0
SHF	0	0	0	0	0	3.01144241246311 (9.36698981520396e-07)	0	0	0	0
SHH	0	0	0	0	0	6.50209663228969 (6.23618388172978e-07)	3.84620359181188 (0.00441793025345332)	0	0	0
SHISA3	0	0	0	0	0	0	0	0	-4.05510647772397 (0.000996823827951769)	0
SHISA4	0	0	4.41508005293136 (2.93281881667291e-07)	0	0	0	0	0	0	0
SHISA6	0	0	0	0	4.29199482315792 (0.00282276136661386)	5.29667164103548 (0.000348989654895166)	0	0	0	0
SHISA7	0	0	0	0	0	6.72995550820238 (0.000709096996116055)	0	0	0	0
SHISA9	5.57578434103428 (4.41791624523513e-05)	0	0	0	7.37865425337025 (1.20412061759675e-06)	7.63991597507091 (4.00148477485352e-05)	6.25030841883087 (0.00399076785671122)	0	0	0
SHMT1	0	0	0	0	0	3.5886362623911 (3.00964242640347e-14)	0	0	0	0
SHMT1P1	0	0	0	0	5.09883528503147 (0.0016793418842729)	0	0	0	0	0
SHMT2	0	2.77380250503676 (0.00114859038210543)	0	0	0	0	0	0	0	0
SHOX2	0	0	0	0	0	6.31106598150167 (0.00817054750650648)	4.34061249724597 (0.00669836058486288)	0	0	6.40002109779012 (0.00168089835707986)
SHROOM1	-2.7671475589366 (0.000229471307625666)	0	0	0	0	2.68182319444098 (0.000196738633999826)	0	0	0	-6.21274271378583 (0.00650725917913086)
SHROOM2	-4.10143337256894 (1.10219064349709e-08)	0	-2.47780272289946 (3.19609638317339e-05)	-7.26388769476959 (0.000953159041202578)	0	0	0	-6.20472838697692 (0.0049468292067607)	0	-3.10260771857371 (0.00411027935479865)
SHROOM3	0	0	-1.60185470002794 (0.000320841518842594)	0	0	0	0	-4.92210847586271 (0.00234991579529319)	0	-2.80208719514904 (0.000779296402117764)
SHROOM4	-2.48533583463436 (7.19472355611621e-08)	0	0	0	-2.30225681072585 (0.00107433204637519)	0	0	0	0	-2.38021751149179 (0.00298599791283325)
SI	5.90658287609383 (2.78555395298387e-05)	0	0	0	0	0	0	0	0	0
SIAH1	0	0	0	0	0	0	0	0	1.93050956633522 (0.00182275294274006)	0
SIAH2	0	0	0	0	0	0	0	2.26803244946072 (0.00545025551988223)	0	0
SIAH3	0	0	0	0	0	7.67975599464534 (5.8349271991727e-05)	0	0	0	0
SIGLEC1	0	0	0	0	0	0	0	-4.80014446284485 (0.00730552904720312)	0	0
SIGLEC10	-3.42272490607652 (0.00768273527737287)	0	-5.63781563164319 (0.000299020302808083)	0	-4.0522878593725 (0.00542898374040549)	0	0	-5.0746083355415 (0.00806706837256502)	-3.5380644475005 (1.0442015213632e-05)	-8.86790103786024 (0.00247356172453668)
SIGLEC14	0	0	0	0	0	0	0	0	-3.55162851719022 (0.000362490277971813)	0
SIGLEC5	0	0	0	0	0	0	0	0	-3.11355495929856 (0.00199485469007908)	0
SIGLEC6	0	0	0	0	0	0	0	0	7.82299718486448 (3.87446656650293e-16)	0
SIK3-IT1	0	0	-2.68741124845519 (0.00217297884979137)	0	0	0	0	0	0	0
SIM1	0	0	0	0	7.28799440194193 (1.82186019380244e-06)	7.26085766764107 (0.00104744173405789)	0	0	0	0
SIM2	0	0	0	0	6.23052370694682 (7.45628528362707e-07)	0	0	0	0	0
SIPA1L1	0	0	-1.94158512921514 (1.4893981446302e-06)	0	0	0	0	0	0	0
SIPA1L2	0	0	0	0	0	0	0	-2.63319367011787 (0.00128540878183183)	0	0
SIPA1L3	0	0	0	0	0	0	0	0	-1.5320533575769 (0.00112030355948515)	0
SIRPB1	-3.25269074336201 (0.00020195422660056)	0	-2.32363348334863 (0.00737301623514315)	0	0	0	0	0	-2.02978295843882 (0.00171681355453497)	0
SIRPB2	0	0	-2.6259491127724 (0.00799528556747671)	0	0	0	0	0	0	0
SIRPD	0	0	0	0	0	5.25344775632081 (0.00921951071388446)	0	0	0	0
SIRT3	0	0	0	0	0	2.0567126208757 (0.00256489336425364)	0	0	0	0
SIRT5	0	0	2.832005403017 (0.000137904129043785)	0	0	3.23329384438929 (0.00562265147406118)	1.67632406770261 (0.00291412417294922)	0	0	0
SIRT7	-1.9697433199588 (0.00647467453793928)	0	0	0	0	0	0	0	0	0
SIX2	0	0	0	0	0	6.97596735093279 (0.000454226970215426)	0	0	0	0
SIX3	0	0	0	0	0	6.73335953638836 (0.00175394198084214)	0	0	0	0
SIX4	0	3.9660560593591 (0.00697443456983125)	0	0	0	0	0	0	0	0
SKAP1	0	0	0	0	0	0	1.89627954873266 (0.0045259763824698)	0	0	0
SKAP2	0	0	-2.04494580555654 (3.23333970843336e-05)	0	0	0	0	0	-1.6386102134871 (3.47123451482549e-07)	0
SKIDA1	0	0	0	0	5.2884415493075 (0.00914553860632148)	6.70890077776941 (1.42445541730259e-05)	0	0	3.36212328335753 (0.00324362682159295)	0
SKOR2	0	0	5.36018043524132 (0.00262896759670672)	0	0	7.44830799101198 (0.000919267628349178)	0	0	0	0
SKP2	0	0	0	0	0	0	0	0	3.54001072200252 (1.41818318170741e-12)	0
SLA	-1.9309005493615 (0.00531862410066269)	0	-2.94904645920958 (2.33436188395274e-05)	0	0	0	0	0	-2.40379589598588 (7.96882210094922e-05)	0
SLAMF1	0	0	0	0	0	0	2.64302684830893 (0.00630326774974609)	0	0	0
SLAMF7	-3.19635170312816 (0.00184139334758481)	0	-2.49962895245959 (0.00995976476965048)	-6.56446240084146 (0.00509835787421304)	0	0	0	0	0	-6.84348519577928 (0.00347638799862038)
SLC10A1	0	0	0	0	0	4.92766704693139 (0.000154248730935149)	0	0	0	0
SLC10A6	0	0	0	0	0	0	0	0	0	7.71610814515995 (1.79240991507195e-05)
SLC11A1	-3.5503815399675 (0.000279298075961878)	0	-4.53956937182251 (1.36108872562586e-15)	-4.35926288141583 (2.30040758323698e-07)	-2.75661315127644 (1.49278618267389e-05)	0	0	0	-3.01028000448189 (7.46190860259226e-06)	0
SLC11A2	0	0	-2.11061261625989 (9.18193882016583e-07)	0	0	0	0	0	0	0

SLC12A1	0	0	0	0	0	6.00374834948544 (0.00126697205280545)	0	0	0	0
SLC12A2	0	0	0	0	0	0	0	-2.93497938069772 (0.00635119473361201)	0	0
SLC12A3	0	0	0	0	0	6.7718725898029 (0.00239250939652811)	0	0	0	0
SLC12A6	0	2.71447381966417 (0.000281224695976012)	0	0	0	0	0	0	0	0
SLC12A7	0	0	1.80900267769769 (2.07812214198849e-05)	0	0	1.64928322768516 (0.00647550313655977)	0	0	-1.68582533759033 (4.02995398043509e-05)	0
SLC12A8	0	0	0	0	0	0	0	0	3.02593848173784 (8.69370785694411e-05)	0
SLC13A2	0	0	0	0	0	6.93187215306646 (5.09444994418538e-05)	0	0	0	0
SLC13A3	0	0	0	0	3.53621931522787 (0.00149246819190168)	3.96424715395067 (0.000364284609586605)	0	0	3.46814499446041 (1.79133590514521e-05)	0
SLC13A4	0	0	0	0	0	0	0	0	3.73186359971758 (7.75368091537509e-07)	0
SLC13A5	0	0	0	0	0	8.28324801994137 (2.54523932654913e-13)	0	0	0	0
SLC14A1	0	0	3.48808315912869 (3.71294950013527e-05)	0	0	3.26085344144742 (0.00246364892306819)	0	0	0	0
SLC14A2	0	0	0	0	6.4020842968154 (1.05191699401894e-07)	0	0	0	0	0
SLC15A1	0	0	0	0	0	5.67795370118957 (0.000143543938881333)	0	0	0	0
SLC15A2	-2.84315301556702 (0.00243812699917166)	0	0	0	0	0	0	0	0	0
SLC15A3	0	0	0	0	0	0	0	0	0	-7.15745592464877 (0.00132262831273789)
SLC16A1	1.70986938248988 (2.12238925533001e-05)	0	1.72949643039387 (0.000103147036271662)	0	0	0	0	0	2.08261817426747 (1.16485456130409e-07)	0
SLC16A10	0	0	-2.65220570341594 (0.00510475935290223)	0	0	0	0	0	0	0
SLC16A11	0	0	0	0	6.57915621825639 (9.59820202109094e-05)	4.93617509927964 (0.00626377974843173)	0	0	0	0
SLC16A13	0	0	0	0	0	4.43669435115679 (8.42670282631176e-08)	0	0	0	0
SLC16A3	-1.8944091491683 (0.00433303745798734)	0	-2.50165957196091 (5.288010636595e-05)	-2.99340641606566 (0.000439570681476368)	0	0	0	0	0	0
SLC16A4	0	3.78858667458843 (0.000115076640155157)	0	0	0	0	0	0	2.11013245755466 (9.86228443556957e-06)	0
SLC16A5	0	0	0	0	3.10353005391579 (0.000744841005832923)	0	0	0	0	0
SLC16A6	-4.29404780850862 (0.0004889605779674)	0	-6.36173585985175 (3.5482974814196e-11)	-5.83576987698839 (0.000813842580111277)	0	0	-2.36202454675196 (0.00100186046856058)	0	-3.71810706643755 (9.20945119452077e-07)	0
SLC16A7	0	0	0	0	0	0	0	0	-2.14214675509677 (7.47840490407683e-07)	0
SLC17A3	0	0	0	0	0	7.24160323056676 (1.69695085320053e-05)	0	0	0	0
SLC17A4	0	0	0	0	0	7.47684595642327 (1.05509576934883e-06)	0	0	0	0
SLC17A5	0	0	-2.48726997794447 (0.000135337982708339)	0	0	0	0	0	0	0
SLC17A7	0	0	4.09415399818058 (0.00417181612867553)	0	0	7.18126855505683 (0.000249579047840535)	0	0	0	0
SLC17A9	-2.48563221896639 (0.00912520561548001)	0	0	0	0	2.18224161219009 (0.00758895940623262)	0	0	-1.91505256491928 (0.00970061826549381)	0
SLC19A3	0	0	0	0	0	3.15625321471803 (0.000755674264680341)	2.24124240059233 (0.00162926573800602)	0	4.53977104412638 (8.27786094672923e-08)	0
SLC1A1	0	0	0	-5.98019716711017 (0.00955141260293364)	0	0	0	0	0	0
SLC1A2	0	0	3.76410162004335 (0.00158918209671685)	0	0	7.24894197236823 (3.25036117253285e-09)	0	0	5.35841594651114 (1.06717573118216e-06)	0
SLC1A3	-1.57901061808858 (0.00961965661390795)	0	0	0	0	-3.06581210417374 (0.0031670190379845)	0	0	0	0
SLC1A5	0	0	0	0	0	-3.38840241556401 (0.00193863714665215)	0	0	0	0
SLC1A7	0	0	0	0	0	4.07656855253039 (0.00887337173636841)	0	0	0	0
SLC20A1	-2.07904394862872 (4.13825726870277e-06)	0	0	0	0	0	0	0	0	0
SLC22A1	0	0	0	0	0	6.62755089553613 (8.01339737449943e-12)	0	0	0	0
SLC22A10	0	0	0	0	0	5.401882563661 (0.00672774209339887)	0	0	0	0
SLC22A11	0	0	0	0	6.24434293510046 (0.0075784156376657)	0	0	0	7.32149397105734 (0.000186886187713699)	0
SLC22A12	0	0	0	0	0	6.03442529655339 (0.00794626862131808)	0	0	0	0
SLC22A14	0	0	0	0	0	7.65903864416723 (0.000496916719364057)	0	0	0	0
SLC22A15	0	0	-1.87977610777183 (0.000366360208099829)	-3.65457223207643 (0.00420157874902658)	-3.2297510474692 (0.00869486051045994)	0	0	0	0	0
SLC22A16	0	0	0	0	0	0	4.79254961947282 (0.00637729348953755)	8.26664967537893 (8.45734802605029e-07)	0	0
SLC22A17	2.6902145857705 (9.22876143636477e-06)	0	0	2.97856964951916 (0.00650382750393523)	0	0	0	0	0	0
SLC22A2	0	0	0	0	6.44049456112654 (0.00312278320909162)	0	0	0	0	0
SLC22A20	0	0	0	0	0	0	5.02185166299294 (0.00701378195202701)	0	0	0
SLC22A25	0	0	0	0	0	5.79836869787938 (0.00166255356291409)	0	0	0	0
SLC22A3	0	0	0	0	0	0	0	0	2.55547178700523 (3.45470888332061e-07)	0
SLC22A31	-6.15696533632508 (2.08678508377771e-06)	0	-4.08414255312288 (0.000289030415841273)	0	0	0	0	0	-6.18849456640495 (1.63429924003488e-08)	-6.27232217256674 (0.00831599803406818)
SLC22A7	0	0	0	0	0	8.34319429777732 (7.75200707470786e-13)	0	0	0	0
SLC22A8	0	0	0	0	6.65055932551467 (0.00282029564248256)	7.41764670773765 (0.00467043662655644)	0	0	0	0
SLC23A1	0	0	0	0	0	4.74288847536862 (0.00210604186091758)	0	0	0	0
SLC24A2	0	0	0	0	0	6.08024614002868 (0.000708255609245075)	0	0	0	0
SLC24A3	2.00353999145652 (0.00146641386791623)	0	0	0	0	0	0	0	0	0
SLC24A4	0	0	0	0	0	3.24683194469577 (0.00513601082150916)	0	0	0	0
SLC25A10	0	0	0	0	0	4.37016709731403 (4.79017596579067e-05)	0	0	0	0
SLC25A11	0	0	1.87218068265729 (0.000193792185785621)	0	0	0	0	0	0	0
SLC25A12	2.2536476594899 (5.9735823089946e-06)	0	1.89596538583624 (2.6639436589475e-05)	0	0	0	0	0	0	0
SLC25A15	0	0	0	0	0	3.59301449129757 (0.000190391817997571)	0	0	0	0
SLC25A18	0	0	0	0	0	5.4805842647769 (4.46613888566203e-05)	0	0	0	0
SLC25A20	0	0	2.22977723592945 (2.48261601463196e-05)	0	0	2.4433671335203 (0.0073174061615303)	0	0	0	0
SLC25A23	2.08376687078048 (1.36587580688516e-07)	2.37670970670162 (0.00712509300002579)	0	2.60807709739247 (0.000157554712242304)	0	0	0	0	-1.88408612181449 (0.000204960093071694)	0
SLC25A24	0	0	-1.81382408984634 (0.00177809984431085)	0	0	0	0	0	0	0
SLC25A25	0	0	0	0	0	1.78305920086386 (0.00940736447445189)	0	0	0	0
SLC25A25-AS1	0	0	0	0	0	2.02693634377445 (0.00663532890293339)	0	0	0	0
SLC25A3	0	0	1.5859897106977 (1.30163991856388e-06)	0	0	0	0	0	0	0
SLC25A34	0	0	4.15699993237066 (0.00190006583261271)	0	0	0	0	0	0	0
SLC25A36	0	0	0	0	0	-2.10420829936389 (0.0072658780711416)	0	0	0	0
SLC25A37	-2.1733835701678 (3.0296150690245e-07)	0	-1.61213591742043 (0.000319825570126258)	-2.42651125042427 (0.00165609296402171)	0	0	0	2.62156913483909 (0.000401474018522973)	0	-2.42505276188036 (0.00154468855181721)
SLC25A39	0	0	0	0	0	0	0	2.48784586234122 (0.00321273019591473)	0	0
SLC25A4	2.00627894051151 (3.49997580018718e-06)	0	5.45680652445481 (6.49852387497615e-29)	2.40352308653156 (0.00271556938006028)	0	0	0	0	0	0
SLC25A42	0	0	0	0	0	3.31055358477273 (3.06642438116756e-05)	0	0	0	0
SLC25A44	0	0	0	0	0	0	0	0	0	-2.25913799776222 (0.00941872777079726)
SLC25A45	0	0	0	0	3.71216644635437 (0.000179543697444068)	3.71159719007306 (0.000504651518507419)	0	0	0	0
SLC25A47	0	0	0	0	0	8.04754915616024 (1.7647899025028e-10)	0	0	0	0
SLC25A5-AS1	0	0	0	0	0	4.85751883112388	0	0	0	0

						(0.00808081526626419)				
SLC25A53	1.62034874282026 (0.00596144084812842)	0	0	0	0	0	0	0	0	0
SLC26A10	0	0	3.73159074376929 (0.00795618619857143)	0	5.81508822141759 (0.000343331037352445)	0	0	0	0	0
SLC26A11	-1.59365219626545 (0.00376055083640094)	2.9908653004828 (0.00224887036767582)	-3.11321201455878 (3.75353461265016e-05)	0	0	0	0	0	0	0
SLC26A2	0	0	0	0	0	-2.19874013112359 (0.00114929869016219)	0	0	0	0
SLC26A7	0	0	0	0	5.01204010507018 (0.0068207027445484)	0	0	0	0	0
SLC26A9	-3.51685863299647 (0.000570147775257402)	0	0	0	0	0	0	0	-5.7192079756069 (1.45220217947919e-07)	0
SLC27A1	0	0	2.50102488767677 (0.000114486664050983)	0	0	0	0	0	0	0
SLC27A2	0	0	0	0	0	4.47195025341218 (8.96663936725836e-05)	0	0	0	0
SLC27A5	0	0	0	0	0	5.05171847290828 (3.51814111186908e-08)	0	0	0	0
SLC27A6	0	0	6.11567700877072 (2.07313643367661e-07)	0	0	0	0	0	6.69636126360342 (3.27739301168299e-10)	0
SLC28A1	0	0	0	0	0	7.78208490038446 (7.92740071593359e-11)	0	0	0	0
SLC29A3	0	0	0	0	0	0	0	0	3.19170513909691 (7.85920495120664e-07)	0
SLC29A4	0	0	0	0	0	5.75638087386089 (3.35054026056955e-05)	0	0	0	0
SLC2A1	0	0	0	0	0	0	0	2.96830229701006 (0.000475945783646762)	3.99252786641741 (3.72183275137119e-27)	3.02532258527536 (0.000265088299394303)
SLC2A1-AS1	0	0	0	0	3.08754028579762 (0.00775309578570078)	0	0	0	2.70694474288717 (5.44748325294313e-05)	0
SLC2A10	0	0	-2.12649686800344 (0.0009871337600846)	0	0	0	0	0	0	0
SLC2A11	0	0	0	0	0	0	0	0	2.20829438058243 (0.00343652652693609)	0
SLC2A2	0	0	0	0	0	8.757967188182 (5.48716804471136e-09)	0	0	0	0
SLC2A3	-2.76553389704781 (4.51746476111697e-07)	0	-2.79393766670229 (4.79617124768789e-09)	-2.04683135137413 (0.00963850390169083)	-1.96632460396859 (0.00372437483842789)	0	0	0	0	0
SLC2A4	4.65669291018649 (6.49721976835815e-13)	0	6.18596216563218 (8.23775795793034e-19)	4.19416209028274 (0.000742649158922153)	0	0	0	0	0	0
SLC2A5	0	0	0	0	0	0	0	4.12701295542239 (0.000332472961252285)	0	0
SLC2A6	0	0	0	0	0	0	0	0	-2.50659726989624 (0.00330899087087302)	0
SLC30A1	0	0	-1.59127414111179 (0.000783138653468519)	-3.30168572317659 (0.000749597592841512)	0	0	0	0	0	0
SLC30A10	0	0	0	0	0	8.3529329686235 (3.68021027435594e-09)	0	0	0	0
SLC30A2	0	0	4.08846903346336 (0.00681727867952484)	0	0	6.15337238156165 (0.00192191356805662)	0	0	5.51216831288693 (1.3292496001817e-06)	0
SLC30A3	0	0	5.50340046068014 (1.73782519973668e-05)	0	0	0	0	0	0	0
SLC30A7	0	0	-1.73237424966562 (1.81151254291304e-05)	0	0	0	0	0	0	0
SLC30A8	0	0	0	0	0	5.11180096061094 (0.00424901164467066)	0	0	0	0
SLC31A2	-3.05409694851341 (0.00778781630058271)	0	0	0	0	0	0	0	0	0
SLC33A1	0	0	0	-2.49167196793088 (0.00425965000397703)	0	0	0	0	0	0
SLC34A1	0	0	0	0	6.48222785639122 (0.00467673319314815)	7.19186452563482 (0.00114979349162556)	0	0	0	0
SLC34A2	-10.176838869925 (1.45042789390919e-28)	-7.89171145578369 (0.000398155067032363)	-8.74767763269509 (4.63246670154436e-32)	-8.36337161304713 (9.38993483867449e-12)	-2.80177785797059 (0.00251281495687259)	-5.23193933334228 (3.67100928129777e-13)	0	-11.3320267004795 (4.14519078203912e-07)	-6.77597627792128 (8.27560966975193e-26)	-5.27571081192352 (1.99841937699327e-08)
SLC35A1	0	0	-2.74151681101432 (0.00498193926983274)	0	0	0	0	0	0	0
SLC35C1	0	0	0	0	0	2.3376885453833 (0.00571007340739275)	0	0	0	0
SLC35E3	0	0	0	0	0	1.74261342672746 (0.00881086792166624)	0	0	0	0
SLC35E4	0	0	0	0	0	0	0	0	-4.322250583814 (0.000146985340292012)	0
SLC35F1	4.82393864438457 (1.1892330980611e-05)	0	0	0	0	5.3619999510681 (0.00823254165918177)	0	0	0	0
SLC35F3	0	0	0	0	6.0138252919796 (0.00331388791111345)	6.75142395771795 (0.00328489540138191)	0	0	0	0
SLC35G1	0	0	0	0	0	4.16936637387655 (0.000899723795854903)	0	0	0	0
SLC35G6	0	0	0	0	5.62868754405502 (0.00471651981745611)	0	0	0	0	0
SLC36A1	0	0	-2.16114463148119 (0.00233528936242794)	0	0	0	0	0	0	0
SLC36A4	0	0	-1.69331253533057 (0.00852249591082373)	0	0	0	0	0	0	0
SLC37A4	0	0	0	0	0	2.18308346599226 (0.00261312302112754)	0	0	0	0
SLC38A1	1.88819187400988 (3.99198651439486e-08)	0	0	1.80796191047662 (0.00327450204517665)	0	0	0	0	1.62073467604185 (1.28087008297801e-06)	0
SLC38A2	0	0	0	0	-1.70112371690066 (0.000905947687132836)	0	0	0	0	0
SLC38A3	0	0	0	0	0	8.79858762328123 (1.27829523647127e-06)	0	0	0	0
SLC38A4	0	0	0	0	0	8.26979241925586 (1.77574073584944e-07)	0	0	0	0
SLC38A5	-3.05687969319421 (0.000187577726934403)	0	-2.91861529263466 (0.00231164727656501)	0	0	0	0	0	-2.49194728985399 (0.000662406704356718)	0
SLC38A7	0	0	0	0	0	1.99144721031503 (0.00323568165954966)	0	0	0	0
SLC38A9	0	0	0	0	0	0	0	0	2.65097714068548 (2.231214423647e-10)	0
SLC39A11	-1.71987326103311 (0.000353734108194665)	0	-1.96328848795593 (0.00274632892420425)	0	0	0	0	0	0	0
SLC39A14	0	0	1.9050954672271 (7.1287235524522e-06)	0	0	3.6099984153396 (1.45335828214445e-06)	0	-3.45460853469557 (0.00187391595644713)	0	0
SLC39A5	0	0	0	0	0	7.4824389889536 (3.2454782340465e-07)	0	0	0	0
SLC39A8	-3.51051399102849 (2.15101367321356e-07)	0	-5.05406517618709 (3.22654308308983e-12)	-4.54455443765914 (4.88261841687951e-05)	-4.86369646109909 (2.6621210021303e-05)	0	0	0	-1.60262821041448 (0.00446642038343252)	-2.72567030924694 (0.00712907412476438)
SLC41A1	0	0	3.13657166483904 (2.34318705862037e-10)	0	0	0	0	0	0	0
SLC41A3	0	3.1631652461086 (0.00535330507355735)	0	0	0	0	0	0	0	0
SLC43A1	0	0	0	0	0	2.05156163204036 (0.003475008074377)	0	3.12251065365586 (0.00153746508017411)	0	0
SLC43A2	-2.17014837825873 (1.37654570059814e-05)	0	-1.8731229414683 (0.000406072957933844)	-2.37148317574149 (0.00954686765172216)	0	0	0	0	0	-2.49990292038921 (0.00559201723320582)
SLC43A3	0	0	0	0	0	0	0	0	-2.14783959624447 (1.45859528952269e-06)	0
SLC44A1	0	0	-1.69440683504291 (2.55130966123652e-05)	0	-2.03228610159438 (0.00658486946012233)	0	0	0	0	0
SLC44A4	-3.84918167775292 (0.000162967858593678)	0	-3.19549398797705 (0.00229756472219826)	0	0	0	0	0	-2.35622453307276 (0.000707520628837596)	-6.19972226515441 (0.00776634531451101)
SLC44A5	0	0	0	0	0	0	0	0	6.13345668903098 (3.73954436833973e-08)	0
SLC47A1	0	0	0	0	0	3.90866733774656 (3.40377199278499e-05)	0	0	0	0
SLC47A2	0	0	0	0	0	7.5665680990688 (0.000158124885767262)	4.50901168216222 (0.00760951544864832)	0	0	0
SLC48A1	0	0	0	0	1.74430747316836 (0.0070878547120428)	0	0	0	0	0
SLC4A1	-4.64758587280306 (1.43020668497778e-05)	0	0	0	0	0	0	6.5395227180784 (3.25352881514079e-11)	0	0
SLC4A10	0	0	0	0	6.87891168603988 (5.203616856777e-05)	0	0	0	0	0
SLC4A11	0	0	0	0	5.16353795176327 (0.000157189944393614)	0	0	0	0	0
SLC4A3	0	0	7.83863409780011 (5.149699509438e-28)	0	0	4.03570854317433 (0.00209010149031659)	0	0	2.34996645010207 (0.00409367260435037)	0
SLC4A4	0	0	0	0	2.18026757234142 (0.00672311648563394)	2.47824870354667 (4.5504026221425e-05)	0	0	-2.06027428854866 (0.00133189563148999)	0
SLC51A	0	0	0	0	0	3.89301562694632 (1.59057289643559e-09)	0	0	0	0
SLC52A1	0	0	0	0	6.0362426662128 (0.000265905654928788)	0	0	0	4.60815517474555 (4.4566721196667e-05)	0
SLC52A3	0	0	3.85712939780973 (0.000449708911476712)	0	0	4.66569132823587 (0.00967625186921369)	0	0	0	0
SLC5A1	4.07531066248393 (0.00649753461147553)	0	8.52326218180076 (1.02018275499754e-15)	0	0	6.87255389122489 (4.04185931242156e-06)	5.08536085950579 (0.00175014531208939)	0	0	0
SLC5A12	0	0	0	0	9.59391835529978 (1.76496916987913e-14)	0	0	0	0	0

SLC5A3	0	0	0	0	0	0	0	0	1.83875448082757 (8.79952183806889e-05)	0
SLC5A6	0	0	0	0	0	2.89253657552605 (0.000716055295728903)	0	0	2.63276517088595 (0.000302006605672192)	0
SLC5A7	5.29227312574074 (4.07806142545593e-05)	0	0	0	0	0	0	0	0	0
SLC5A8	-6.30297553039421 (0.00259705194209937)	0	-4.51477553192178 (0.00288234322637017)	0	0	0	-2.85696113705269 (0.00660861431491459)	0	-6.33889552175217 (0.000733651439414399)	0
SLC6A12	0	0	0	0	4.1548262690842 (0.000780551032427001)	4.22496106513811 (0.000927437175766547)	0	0	0	0
SLC6A13	0	0	0	0	0	7.8876288592763 (7.33336096933428e-06)	0	0	0	0
SLC6A14	-8.09180837323008 (5.45959750688907e-08)	0	-8.35984878702989 (9.20048824414969e-08)	-9.9903452402252 (0.00102354435736068)	-5.4926921056555 (0.000110870472977521)	-4.47509463987287 (0.00126546623190294)	0	-8.93091877586762 (0.0033215322352986)	-9.08397326956109 (2.45715735177697e-09)	0
SLC6A15	0	0	0	0	0	0	0	0	4.18968662065091 (0.000112692278907387)	0
SLC6A17	0	0	0	0	6.77612808267073 (0.000128192801492393)	5.65292840039313 (0.0042731565314787)	0	0	0	0
SLC6A19	0	0	0	0	0	7.07308077663729 (0.00138932745134119)	0	0	0	0
SLC6A2	0	0	0	0	0	0	0	0	7.13213730112049 (1.94020879683096e-10)	0
SLC6A4	-5.34055624239005 (2.27566857577911e-06)	0	-5.75701947996143 (9.27572678092814e-07)	-7.37912952747064 (0.00209237627064483)	0	0	0	0	0	-7.65614119682031 (0.00140300953978581)
SLC6A5	0	0	0	0	6.12132713337718 (0.00201419557017153)	0	0	0	0	0
SLC6A6	-2.59239065702791 (2.40840804081856e-07)	-5.6261087671629 (0.00870684371145687)	0	-3.51888231008338 (2.10659249186377e-06)	0	-1.94575698758425 (0.000542835825029981)	0	0	-1.67443484570727 (8.18524325677379e-09)	0
SLC6A8	1.7974945003074 (1.0862399993339e-05)	0	3.00445436284925 (2.70946077342846e-09)	2.50313420696782 (0.00163294628257621)	0	0	0	0	2.61331522484856 (2.38137669846697e-06)	0
SLC6A9	0	0	-2.8122358804583 (0.00505101086489056)	0	0	0	0	0	-2.6658508163389 (0.000336542164795756)	0
SLC7A10	0	0	0	0	6.12132713337718 (0.00201419557017153)	0	0	0	0	0
SLC7A11	-6.14243328014474 (0.000266010023745426)	0	-3.74793601878981 (0.000143183724383108)	0	0	0	-1.88271737736827 (0.00296038903840149)	0	-4.04044385091318 (2.04865958325612e-06)	0
SLC7A11-AS1	-5.2161931318908 (1.9303422775146e-05)	0	-4.75863903064407 (7.0802727982405e-05)	-6.89831415267695 (0.00467477464483363)	0	0	0	0	-3.3322478163899 (0.000362489512222798)	0
SLC7A14	6.16121260620523 (1.03294649433053e-06)	0	0	0	6.99576428898384 (1.76731941067209e-05)	7.43682223756985 (0.00053212442989317)	0	0	0	0
SLC7A2	0	0	0	0	0	3.08835616134592 (5.66638022194723e-06)	0	0	1.60392188440166 (0.0032184894491017)	0
SLC7A7	0	0	-3.46668793410561 (0.000452149480794328)	0	0	0	0	0	0	0
SLC7A8	0	0	-1.98284965718325 (0.00126133327933084)	0	0	0	0	0	0	0
SLC7A9	0	0	0	0	0	5.78719774298348 (0.000256507861733772)	0	0	0	0
SLC8A1	0	0	3.19256855514002 (2.25212966825468e-08)	0	0	0	0	0	0	0
SLC8A2	5.31683336591601 (1.57542478685412e-05)	0	0	0	0	0	0	0	0	0
SLC8A3	0	0	0	0	4.70924173529614 (0.00136716825019099)	0	4.02299855929649 (0.00426975225070819)	0	0	0
SLC9A2	0	0	0	0	6.64816874282679 (0.00347040281013931)	8.45710524368761 (0.00562260803010125)	0	0	0	0
SLC9A3	0	0	0	0	7.20756510123936 (9.90789395903898e-07)	0	0	0	0	0
SLC9A3R1	0	0	0	0	2.56719773613068 (0.00410351578725)	4.59304788731354 (1.2448897746691e-07)	0	0	0	0
SLC9A3R2	-1.86643025067255 (7.43130321649198e-07)	0	0	-1.83934972201772 (0.00948311339808674)	0	0	0	-3.75514930879861 (2.26944067479872e-05)	0	-1.99365438816215 (0.00486455929264342)
SLC9A4	0	0	0	0	0	6.61489342504846 (0.000500265812804008)	0	0	0	0
SLC9A5	-3.90689043863095 (0.0094568064121774)	0	0	0	0	0	0	0	0	0
SLC9A9	1.71090320523735 (0.000111935579390581)	0	0	0	0	0	0	0	0	0
SLC9B2	0	3.561597223169 (0.00296561126557109)	0	0	0	0	0	0	0	0
SLC9C1	0	0	4.4837397715975 (0.00107996501804455)	0	0	0	0	0	0	0
SLCO1A2	0	0	0	0	0	6.1590157979415 (0.00122147639504661)	0	0	0	0
SLCO1B1	0	0	0	0	0	8.92127880636148 (8.92355917668839e-09)	0	0	0	0
SLCO1B3	0	0	0	0	0	6.11638868273182 (0.00979003595100478)	0	0	0	0
SLCO2A1	-2.23349324738234 (5.31517328131664e-05)	0	0	0	0	-2.93258800608385 (0.000359142048367528)	0	-8.4417753466459 (9.72449007323939e-05)	0	-3.43172212330655 (1.75413134596249e-05)
SLCO2B1	0	0	-1.79212060234994 (0.00118521347211798)	0	0	0	0	-2.93744358419743 (0.0019854895519188)	0	0
SLCO3A1	0	0	0	0	0	0	0	0	-3.11836838187948 (3.74651035086905e-11)	0
SLCO4A1	-3.76485020282022 (2.42815565009629e-09)	0	-2.18302719874032 (0.000149861340713632)	0	0	-2.14524828629187 (0.00478889334705811)	0	-3.64620512705092 (0.00168228558698754)	-2.70975879244783 (6.43399757598271e-07)	0
SLCO4A1-AS1	0	0	0	0	0	0	0	0	-3.27982736179757 (0.00473935970666112)	0
SLCO4C1	-4.57399246320453 (0.0014582909596023)	0	-4.66186014733091 (2.117062177336e-05)	0	0	0	0	0	-4.48509753641012 (3.29065952053487e-06)	-5.07789834804447 (0.00804525692902709)
SLCO5A1	0	0	4.56635083694869 (1.17863838381489e-08)	0	0	4.35893376520227 (0.00264785853650355)	0	0	0	0
SLED1	-4.41317175049241 (1.68119424208405e-05)	0	-3.93167838333331 (3.72903221980495e-05)	0	0	0	0	0	-3.33826200001579 (3.87004658478051e-06)	0
SLFN11	-1.50220709993562 (0.000270269377725976)	0	0	0	0	0	0	0	-1.87975798533409 (6.49446554235894e-08)	0
SLFN13	0	0	-2.05120408315109 (0.00842681277114456)	0	0	0	0	0	-1.82459088203563 (0.00185474200914875)	0
SLFN5	0	0	0	0	0	-2.54935889751557 (6.10251578782107e-07)	0	-2.48606721523202 (0.000635778385752164)	0	0
SLFNL1	0	0	0	0	0	4.39376196081909 (0.000676128966780989)	0	0	0	0
SLFNL1-AS1	1.74313006132909 (0.00718507199694892)	0	0	0	0	2.85098461224491 (0.00317928505539131)	0	0	0	0
SLIT2	0	0	-1.57782054340271 (0.000728208630331559)	0	-2.22114209888176 (0.0028496136017558)	-1.98062304891121 (0.00149846641186925)	0	-7.89343638576626 (0.000288916745633799)	0	-2.396342412366 (0.00274012836643974)
SLIT3	0	0	-2.04941965447908 (0.00018808523119277)	0	0	0	0	-8.87812560179107 (3.39444847293826e-05)	0	0
SLITRK2	0	0	0	0	3.64255208347438 (0.00472579566082092)	0	0	0	-3.72440757208878 (0.00806270330566741)	0
SLITRK3	6.32796744153425 (6.11519368757959e-09)	0	0	0	0	0	0	0	0	0
SLITRK5	3.29440909762273 (0.00739116421608991)	0	0	0	5.06417822637345 (0.000894664670333749)	0	4.40262785441942 (0.00946587437692791)	0	0	0
SLMAP	1.75505137803959 (1.27417297341183e-10)	0	0	2.51559078759775 (7.5715700556333e-06)	0	0	0	0	0	0
SLPI	-1.67552646671763 (0.00523664819879371)	0	-4.11117825744456 (1.36141368562045e-06)	-3.07435362967984 (0.007334225782837)	0	0	0	0	-3.58763484162729 (1.93447434472415e-05)	0
SLURP1	0	0	0	0	0	0	0	0	0	8.02277179430784 (1.96236870256944e-06)
SMAD6	-2.81170732572268 (0.000303322715844797)	0	0	-4.49165354979594 (0.00052110493859148)	0	0	0	-7.87457652111159 (0.000686691117453281)	0	-4.7704883373597 (0.000229543714808897)
SMAD9	2.64557427798281 (4.54559429681501e-11)	0	0	0	0	0	0	0	0	0
SMAGP	0	0	0	0	0	0	0	0	2.00748291064223 (0.00268685437769986)	0
SMAP2	-1.86282764489573 (9.79141518336873e-05)	0	-3.00026860294087 (2.41062984255922e-09)	0	-2.40065090326902 (0.00144137209829752)	0	0	0	-1.53988666949328 (1.74135844702361e-05)	0
SMARCA1	0	0	0	0	0	0	0	-2.77436784164596 (0.00610481268312027)	0	0
SMARCB1	0	2.7546705005769 (0.00213331910744416)	0	0	0	0	0	0	0	0
SMARCE1P2	0	0	0	0	0	6.16980119559886 (0.00649144516590301)	0	0	0	0
SMC2	0	0	0	0	0	0	0	3.10406884108254 (3.21961440447193e-05)	0	0
SMC3	0	0	0	0	0	-2.27714765493688 (7.21139131846758e-05)	0	0	0	0
SMC4	0	0	0	0	-2.25580400944106 (0.00170386899966602)	0	0	2.48629541807943 (0.000134618225086712)	0	0
SMG1P1	-1.58689366194829 (0.00487062295273298)	0	0	0	0	0	0	0	0	0
SMG1P4	0	0	0	0	0	3.19167189133167 (0.00772347110810098)	0	0	2.29637776170534 (0.00727687596341322)	0
SMIM1	0	0	0	0	0	0	0	7.03130079722076 (9.91594863264378e-06)	0	0
SMIM10	0	0	0	0	2.09854314109429 (0.00230381648866987)	0	0	0	0	0
SMIM13	0	0	0	0	0	0	0	0	2.00050494462557 (9.02684220022222e-08)	0
SMIM14	0	0	0	0	0	0	0	-3.97313400856318	0	0

								(0.00207326065503713)		
SMIM24	0	0	0	0	6.19102403945923 (0.00730943111243636)	6.95458610146953 (1.91005024654223e-06)	0	0	0	0
SMIM5	0	0	0	0	3.01907294514166 (0.00481452157923108)	0	0	0	0	0
SMLR1	0	0	0	0	0	5.57078326866812 (0.00366181258334402)	0	0	0	0
SMN1	-3.04050756963146 (0.00292363571618018)	0	0	0	0	0	0	0	0	0
SMO	0	0	0	0	2.52755386188427 (0.00707711662938025)	2.83095669206153 (0.00166972704695297)	0	0	0	0
SMOC1	0	0	0	0	5.2928439506446 (0.00908567205209642)	6.64967562576156 (3.21551791824307e-07)	0	0	4.54450874198143 (3.33390054733608e-05)	0
SMOC2	2.21590429081363 (0.0001512133705042)	0	2.34417605493029 (0.000177484457717903)	0	0	0	0	0	2.02916954698402 (0.000582404130243408)	0
SMPD3	0	0	0	0	0	3.86517478292715 (0.00037596867094)	0	0	0	0
SMPDL3A	-2.41475946936801 (0.00219818318631502)	0	-2.57943225785911 (0.00631275161063564)	0	0	0	0	0	0	0
SMPDL3B	0	0	0	0	0	0	4.06187792580408 (0.000294816043088627)	0	0	0
SMPX	6.17385750202621 (4.352328731381e-06)	0	9.5763684030693 (9.25880991615874e-18)	7.95834940648001 (3.81317622483139e-06)	0	0	0	0	0	0
SMS	0	0	0	0	0	-2.42323236024848 (0.000564860331682064)	0	0	0	0
SMTN	2.42787390420459 (1.00253275191021e-09)	0	0	2.55982048457203 (0.000259947196096451)	0	0	0	0	0	0
SMTNL2	0	0	1.96502718950283 (0.00810279461670511)	0	0	0	0	0	0	0
SMYD1	9.71787270845118 (2.68288989180958e-21)	0	10.8880024471046 (6.08352675266027e-23)	9.80418578578543 (6.92470956471997e-14)	0	8.19250618406407 (0.00109421045875971)	0	0	0	0
SMYD2	0	0	2.76377734011606 (1.35913073307839e-07)	0	0	0	0	0	0	0
SNAI2	0	0	0	0	0	0	0	-6.66082022087714 (0.00286295970474407)	0	0
SNAI3-AS1	0	0	0	0	0	0	3.26773018733767 (0.0017804937799278)	0	0	0
SNAP25	3.66055873400231 (0.000931323311469074)	0	0	0	0	0	0	0	0	0
SNAP47-AS1	0	0	0	0	0	6.76296017326266 (0.00203331465568263)	0	0	0	0
SNAP91	4.91114478573142 (0.00577392231011906)	0	0	0	0	0	0	0	4.65057823884472 (0.00584337549162572)	0
SNCA	0	0	-2.841952510193 (0.00224698436426099)	0	0	0	0	0	0	0
SNCAIP	0	0	0	0	0	3.56340105917655 (0.00693047988956441)	0	0	2.6481379761942 (0.00133802741926405)	0
SNCG	2.27776602681976 (0.000426260440103719)	3.99652715924438 (0.00296360901027995)	2.01595022307119 (0.00242053867028679)	0	0	0	0	0	0	2.84387716216806 (0.00926837205228871)
SND1-IT1	0	4.22539814843593 (3.9294121124097e-06)	0	0	0	0	0	0	0	0
SNED1	0	0	0	0	0	0	0	0	-2.21642019877605 (0.000754052826324462)	0
SNHG1	0	0	0	0	0	1.63456867361675 (0.00593071727310828)	0	0	0	0
SNHG11	0	0	0	0	0	3.03420181000074 (0.000128025749505873)	0	0	0	0
SNHG14	0	0	1.52457138488324 (0.000394729222801353)	0	0	0	0	-2.77285985656575 (0.0047483761177769)	0	0
SNHG23	0	0	0	0	0	0	0	0	5.63819754733271 (6.34192268750721e-18)	0
SNHG24	0	0	0	0	0	0	0	0	5.64332741605675 (6.18607401114519e-09)	0
SNHG6	0	0	-1.59450881252777 (0.00303289815283461)	0	0	0	0	0	0	0
SNHG9	0	0	0	0	0	0	2.05657888491145 (0.00736825092101594)	0	0	0
SNN	0	0	1.83645064819661 (0.000145216053629332)	0	0	0	0	0	0	0
SNORA11	1.7867304225372 (0.00770672190609102)	0	2.20161024034332 (0.00549297531260656)	0	0	0	1.70272999227931 (0.00391138063077397)	0	2.1325514228704 (0.00102745896001601)	0
SNORA13	2.53246175921354 (0.00162962972670268)	0	2.54698011910261 (0.00910891154749817)	0	0	4.4769897952663 (3.7513279504417e-05)	3.30868212852109 (5.64302186995676e-05)	0	3.4468584099091 (2.76537503844197e-07)	0
SNORA14A	2.10501749756333 (0.00912989198477427)	0	0	0	0	4.53493596318809 (0.000529106666083519)	0	0	0	0
SNORA19	0	0	0	0	0	0	3.43426979815568 (0.00399719833943419)	0	4.41804927566724 (9.28637122904066e-05)	0
SNORA2A	0	0	4.13888929375588 (0.0039882251569921)	0	0	0	3.51947225907367 (0.000789719777362668)	0	0	0
SNORA2B	0	0	0	0	0	4.9030767733444 (0.00515689917812065)	3.2900463197668 (0.00180117461736772)	0	3.27794255163743 (0.00118490316552196)	0
SNORA31	0	0	0	0	0	0	0	0	0	-6.89403902668206 (0.00414972833716114)
SNORA33	0	0	0	0	0	0	1.67762330127588 (0.00212979883087234)	0	0	0
SNORA35	3.59795010116149 (2.03003171557571e-06)	4.56238512764451 (0.00204121931883694)	0	0	0	0	2.43288965365628 (0.000846414727631222)	0	3.29629355058925 (1.67921984824204e-07)	0
SNORA38B	0	0	5.0541434661487 (3.23082398490874e-05)	0	4.53008418366587 (0.0027813738800673)	6.12907144405486 (3.92155469604334e-07)	4.14599192577313 (2.7064128682274e-05)	0	0	0
SNORA40	2.76759658953038 (0.000363443993680334)	0	0	0	0	4.12801860541193 (0.000625870141371009)	2.2140385683178 (0.00321048421372817)	0	2.1716624258751 (0.00114652368060834)	0
SNORA45B	0	0	0	0	0	0	0	0	1.6426651683341 (0.00804371382263786)	0
SNORA47	1.9616686602532 (0.00330123320414354)	0	2.54208088203179 (0.00117616061925645)	0	0	2.93036385581359 (0.00657657115126497)	0	0	0	0
SNORA50	3.5191898346785 (6.03998639379576e-05)	0	3.46583057909368 (0.000315787540795352)	0	0	0	2.07434442427013 (0.00169041026867866)	0	0	0
SNORA53	0	0	0	0	0	0	0	0	1.51296381021047 (0.0014664596296507)	0
SNORA5A	2.21085404925573 (0.00260294965961686)	0	0	0	0	0	1.81306927463026 (0.0021413756215717)	0	2.24315616979238 (0.000492217062049652)	0
SNORA5C	0	0	3.03956093500542 (0.00104253705006752)	0	0	3.60410928623359 (0.00114707727107704)	0	0	0	0
SNORA60	0	0	2.71922479286459 (0.00581369891711175)	0	3.45999145431801 (0.00761490662198683)	3.49470357009084 (0.000464640886724951)	0	0	0	0
SNORA65	0	0	0	0	0	0	0	0	1.98729537053808 (0.00241997089599315)	0
SNORA71D	0	0	0	0	0	0	1.68328395751421 (0.00559964389733855)	0	0	0
SNORA73B	0	0	0	0	0	2.8097746166754 (0.00840818225860957)	0	0	0	0
SNORA74B	0	0	1.76581545712342 (0.00558165983176771)	0	0	0	0	0	0	0
SNORA75	0	0	0	0	0	3.12849712892669 (0.00352671025251504)	0	0	0	0
SNORA77	2.86140791594475 (0.00684699860347034)	0	3.05174624290492 (0.000455568430051208)	0	0	3.84947063302001 (0.0010377233882602)	0	0	0	0
SNORA80A	0	0	3.92016914807579 (0.00786760433285164)	0	0	4.9160833745428 (0.0032439083996495)	3.43782267087094 (0.00773858909920562)	0	0	0
SNORA80B	0	0	0	0	0	4.4842780630454 (0.00925790134970219)	3.16108516627939 (0.000923275688287385)	0	0	0
SNORD100	0	0	0	0	0	4.09985690504998 (0.000117676200189766)	0	0	0	0
SNORD104	0	0	0	0	0	4.99158676815796 (0.000111142768840932)	0	0	0	0
SNORD105	0	0	0	0	0	5.42553418641501 (0.0064338245173487)	0	0	0	0
SNORD105B	0	0	0	0	0	2.78041098605539 (0.0078538664436223)	0	0	0	0
SNORD112	0	0	0	0	0	0	0	0	5.74637129513926 (9.28814982795348e-08)	0
SNORD113-4	0	0	0	0	0	0	0	0	4.50744844343145 (0.000198527483625575)	0
SNORD113-6	0	0	0	0	0	0	0	0	6.51982859128551 (1.17533820345046e-08)	0
SNORD113-7	0	0	0	0	0	0	0	0	4.41680271276797 (0.000786806893500223)	0
SNORD113-8	0	0	0	0	0	0	0	0	6.92584740688725 (5.93893843178936e-10)	0
SNORD113-9	0	0	0	0	0	0	0	0	3.92328963315918 (0.003748728912785)	0
SNORD114-1	4.67643073160604 (0.000278633441129009)	0	5.40314459780724 (0.000215171147649729)	0	0	5.6579003478258 (0.00560532794193948)	0	0	7.00461528243781 (9.48786525808715e-12)	0
SNORD114-12	0	0	0	0	0	0	0	0	5.77573261787791 (8.19330396472095e-08)	0
SNORD114-14	4.18487385594016 (0.00223724558659146)	0	0	0	0	0	0	0	6.76489723571855 (2.24422504376076e-10)	0
SNORD114-22	0	0	0	0	0	0	0	0	5.28744605668022 (3.92545390579227e-06)	0
SNORD114-28	0	0	0	0	0	0	0	0	4.57225637971586 (0.000290773579359735)	0

SNORD114-3	0	0	0	0	0	0	0	0	6.10781744064994 (4.01950699951382e-09)	0
SNORD114-9	0	0	0	0	0	0	0	0	4.591671089189 (0.000256715096818073)	0
SNORD115-32	5.12807701000386 (0.00156120413203015)	0	0	0	0	0	0	0	0	0
SNORD115-5	5.2244316155912 (0.000406825899378034)	0	0	0	0	0	0	0	0	0
SNORD116-1	2.36381052976675 (6.76180586352146e-05)	0	2.14372316587374 (0.00173948302435357)	0	2.47639313707206 (0.00778705496604618)	0	1.53078394290745 (0.00143570488402593)	0	0	0
SNORD116-14	2.54557587557354 (0.00619764138592844)	0	0	0	0	0	0	0	0	0
SNORD116-15	2.35769710420745 (0.000146524995099352)	0	2.25097509977812 (0.00746242314803399)	0	0	0	1.56290642613625 (0.00206570161955857)	0	0	0
SNORD116-16	2.09127073218178 (0.00689258007235983)	0	0	0	0	0	0	0	0	0
SNORD116-2	3.31098066017285 (2.67366997609026e-06)	0	3.39626581078922 (4.97311202083864e-06)	0	3.21453906952685 (0.00346897772208792)	3.06623670968975 (0.00178053649748043)	2.69813527080605 (3.59418456401248e-06)	0	2.24436581099366 (0.000154559206381 72)	0
SNORD116-23	5.24705557430196 (1.97628525878452e-05)	0	0	0	0	4.86134103622259 (0.00645833539535979)	3.62829270125277 (0.00149127651916354)	0	0	0
SNORD116-25	5.14407235976857 (1.20366101373518e-05)	0	4.61173941062513 (0.00154379531578723)	0	0	0	3.75265147286799 (0.000770844940196994)	0	4.07981088138956 (0.000310564695523392)	0
SNORD116-26	3.49147139068793 (0.000179322504531838)	0	0	0	0	0	0	0	0	0
SNORD116-27	4.2632526930112 (0.000214277723999492)	0	0	0	0	0	0	0	0	0
SNORD116-29	5.03462379635614 (0.000127236315624913)	0	5.98414672384546 (0.00793053593957875)	0	6.50003006936233 (0.000274239767040683)	0	3.7106111869926 (0.00217707572137661)	0	0	0
SNORD116-6	2.61494477067919 (8.29983255154024e-06)	0	2.15793728913103 (0.00706458081021246)	0	0	2.36223563188221 (0.00534137207411553)	1.90516134519607 (0.000625062629901105)	0	1.67732201258202 (0.00408401926117341)	0
SNORD116-8	2.3923323837869 (1.53668213761569e-05)	0	2.44750244098004 (6.30736018625093e-05)	0	2.4338692559743 (0.00668341409390968)	2.45780458314797 (0.00511331812962494)	1.82472904562238 (0.000401240673396597)	0	1.70540403398446 (0.000772046567645042)	0
SNORD127	5.17983436673712 (6.53127259103494e-06)	0	0	0	0	0	4.07265480497695 (0.000113945217779594)	0	4.92237041124873 (3.45027638435829e-06)	0
SNORD14E	0	0	0	0	0	4.79405694083748 (0.000223479013744234)	2.70508527424953 (0.00341707282013286)	0	0	0
SNORD15B	0	0	0	0	0	0	0	0	1.56856698801821 (0.00133632599572136)	0
SNORD38A	0	0	0	0	0	3.27380729855166 (0.00339490256517734)	0	0	0	0
SNORD3A	0	0	0	0	0	2.34828001681233 (0.00316145671962555)	0	0	0	0
SNORD3C	0	0	0	0	0	3.13505891085555 (0.00114269756278327)	0	0	0	0
SNORD46	2.00524720299097 (0.00945824545207755)	0	0	0	0	0	1.83107440941963 (0.00662214341947908)	0	2.82609927730345 (6.89583014011633e-07)	0
SNORD53	0	0	0	0	0	4.51431000922621 (0.00345890308340881)	0	0	0	0
SNORD53_SNORD92	0	0	0	0	0	5.38752177248891 (0.00467829135624419)	0	0	0	0
SNORD59A	0	0	3.47419966726803 (0.00294622900326697)	0	0	0	0	0	0	0
SNORD63	0	0	2.76288232318648 (0.0092346046626979)	0	0	4.49039273821461 (0.000116198356071124)	0	0	0	0
SNORD66	0	0	0	0	0	3.68532624717678 (0.000324800780645378)	0	0	0	0
SNORD69	0	0	0	0	0	4.79547948503618 (0.00033902873474225)	0	0	0	0
SNORD71	0	0	0	0	0	0	0	0	2.72447522993481 (0.00131364386490751)	0
SNORD83A	0	0	0	0	0	4.52760650687645 (0.000740277096999415)	2.56120637471291 (0.00532718309748313)	0	0	0
SNORD88A	0	0	0	0	0	5.50168084960291 (0.000626668174447887)	3.38864976776508 (0.00166782454852997)	0	0	0
SNORD89	0	0	0	0	0	0	0	0	1.6929043027574 (0.000353413207803121)	0
SNORD90	0	0	0	0	0	3.88597358342024 (0.00790909423228909)	0	0	0	0
SNORD94	1.86129359816212 (0.000999300575021795)	0	0	0	0	0	0	0	0	0
SNRNP25	0	0	1.80719217299879 (0.00709941317229682)	0	0	0	0	0	0	0
SNRPCP18	0	0	0	0	0	7.28272757431076 (0.0048656497259573)	0	0	0	0
SNTA1	0	0	5.48481911668502 (5.58653549764849e-22)	0	0	0	0	0	0	0
SNTB1	0	0	-1.9109723153814 (0.000855278401767144)	0	0	0	0	0	-2.36618755858 (8.56156178721352e-07)	0
SNX10	-2.30666347087772 (0.00350041922651156)	0	-3.07784434220531 (0.000841549546722031)	0	0	0	0	0	0	0
SNX22	0	0	0	0	0	0	0	0	-2.30319473669555 (0.000329025068121216)	0
SNX27	0	0	0	0	-1.77364205705622 (0.00945904503437527)	0	0	0	0	0
SNX30	-2.23669936657345 (2.38357169862881e-08)	0	-2.32538171864303 (1.60163796572775e-08)	-2.07336750759218 (0.0059137389828999)	0	0	0	0	-1.64388431024711 (1.67955530113032e-07)	0
SNX7	0	3.2559554141176 (0.00170315612261058)	0	0	0	0	0	0	0	0
SOAT1	0	0	-1.95493637082639 (6.87565707847361e-05)	0	-2.10091658871366 (0.0064508086641987)	0	0	0	0	0
SOBP	2.04434022610581 (5.33111943311234e-06)	0	2.03702739512277 (8.69535045661395e-05)	0	0	0	0	0	0	0
SOCS3	0	0	0	0	0	0	0	-1.97017048864179 (0.00522683000932284)	0	0
SOD1	0	0	0	0	0	2.24814113779975 (5.95104164263506e-06)	0	0	0	0
SOD2	0	0	0	0	0	0	0	-2.82972626843325 (0.000525817335337381)	-2.07229348076942 (0.000170103218031614)	0
SOD3	0	0	0	0	0	0	0	-7.79584168232609 (0.000380994298021972)	-1.63140248661525 (0.000933405794294223)	0
SOGA1	0	0	0	1.85181693260382 (0.00699100945583689)	0	0	0	0	0	0
SOGA3	0	0	0	0	0	0	0	0	-2.14487880320132 (0.00904744660830596)	0
SORBS1	2.82312331005608 (9.59737168923504e-12)	-5.61693694976219 (0.00940363686632589)	2.70253006513392 (1.64094432947093e-10)	3.02888106870489 (1.74315606896549e-05)	0	0	0	-3.62531724114501 (4.95718644189043e-05)	0	0
SORBS2	1.72324192780485 (6.27991857761921e-06)	0	4.29717786227931 (1.92359105504478e-21)	1.81734954730493 (0.00465808054238344)	0	1.61158513828013 (0.00231230299883171)	0	-4.17816925425802 (0.00927079619125764)	2.34159776027753 (2.14796369311749e-18)	0
SORBS3	0	0	0	0	0	0	0	-5.60192656968189 (0.000423890046626675)	0	0
SORCS1	6.12202415916125 (4.74483667854005e-11)	0	0	6.82083694301332 (1.13440050924281e-06)	0	5.89220019606183 (8.23187981970879e-05)	0	0	0	0
SORCS3	5.9411549952192 (2.03981304585919e-07)	0	0	0	0	0	0	0	0	0
SORD	0	0	0	0	0	3.96645678284784 (1.46094423808682e-05)	0	0	0	0
SORL1	-2.24453694048248 (3.954421948036e-05)	0	-1.9169302881774 (0.000641221923079993)	0	0	0	0	0	-2.00819498988862 (5.36166090197164e-05)	-2.23834463855285 (0.00618886565791933)
SORT1	0	-5.81030994607537 (0.00663033990877644)	0	0	-1.79381436130165 (0.00061659219378326)	-2.32021919841789 (3.85060911851706e-05)	0	0	0	0
SOS2	0	0	0	0	0	-1.88450808933194 (0.00689359742687123)	0	0	0	0
SOWAHC	0	0	0	0	0	2.77330887909019 (1.37657611169386e-05)	0	0	0	0
SOX11	5.97131259480838 (4.86088805516 65e-07)	0	4.00152511743295 (0.00300461804294901)	0	0	6.00813568053682 (0.00236776366027671)	0	0	0	0
SOX13	0	0	0	0	0	0	0	-7.12763227828072 (0.00131050424676813)	0	0
SOX15	4.55234160677969 (0.00149944810742673)	0	0	0	0	0	0	0	0	0
SOX18	0	0	2.99648551674962 (5.48740792208929e-08)	0	0	0	0	0	0	0
SOX2	3.24939204412828 (0.00475444666872913)	0	0	0	0	0	0	0	0	0
SOX21	0	0	0	0	0	4.80518603413212 (0.00262223200513198)	0	0	0	0
SOX30	0	0	0	0	0	6.0652796603583 (0.00285145512050417)	0	0	0	0
SOX4	-1.74512004146114 (0.000833505485677081)	0	-2.02125475665928 (3.86913242841938e-05)	0	0	0	0	-3.52572689877405 (0.0010299342953126)	0	0
SOX6	0	0	1.51625606019041 (0.00131652110545072)	0	0	2.87867145139771 (2.30221992477855e-07)	0	4.03104030366765 (1.0551410489213e-06)	0	0
SOX7	-2.02945365427864 (0.00465341055885724)	0	0	0	0	0	0	0	0	0
SOX9	0	0	2.22799761913323 (9.72368693860783e-06)	0	2.31614968871108 (0.00362352209450946)	0	0	0	0	0
SOX9-AS1	0	0	0	0	7.04090094548521 (0.00618407582524431)	7.46208406809927 (2.22806166568336e-05)	0	0	0	0
SP140	0	0	0	0	0	0	1.62787589291541 (0.000122814346930979)	0	0	0
SP5	0	0	0	0	0	0	0	0	0	7.05313623554546

										(0.000826100766648662)
SP6	-4.18430347581255 (0.00866702037700413)	0	0	0	0	0	0	0	2.80208420683861 (0.000311864487519488)	0
SPA17	0	0	0	0	0	0	0	0	-1.8949275610816 (0.00789038892173406)	0
SPACA6P	0	0	0	0	0	0	0	-6.59955115871706 (0.00315702754227406)	0	0
SPACA6P-AS	0	0	0	0	0	5.1197758334642 (0.000544686389744683)	0	0	2.87521739187906 (0.00597200503851782)	0
SPAG1	-1.72314791707435 (0.00601848306915669)	0	0	0	0	0	0	0	-2.21713123183834 (0.000271243358824498)	0
SPAG17	-5.36645150332514 (0.000182754081488526)	0	-3.56664137301501 (0.00970022202870269)	0	0	0	0	0	-3.48864997811774 (0.000835682834165491)	0
SPAG5	-2.51125782683184 (0.00314765393811088)	0	0	0	0	0	0	4.17888521279907 (4.71046302688951e-06)	0	0
SPANXN5	0	0	0	0	0	6.54465898329173 (0.00355480838821528)	0	0	0	0
SPARC	0	0	0	0	0	-2.17052889506389 (0.00110414169677295)	0	-5.81584837991779 (1.44651222176174e-13)	0	0
SPARCL1	0	0	0	0	0	-3.45054433344294 (1.02930084050243e-05)	0	-2.9479616671526 (2.70039593837959e-05)	0	0
SPATA13	-2.38404653285009 (3.40442643789939e-05)	0	0	0	0	0	0	0	0	0
SPATA18	0	0	-2.84173190426572 (0.0024047986050795)	0	0	0	0	0	0	0
SPATA24	0	0	3.26926594300887 (0.00156555793358784)	0	0	0	0	0	0	0
SPATA31A1	0	0	0	0	0	7.50034476364749 (0.00335954893947622)	0	0	0	0
SPATA31B1P	0	0	0	0	0	7.43919716374682 (0.000722030872330539)	0	0	0	0
SPATA31C1	0	0	0	0	0	8.1700718289066 (0.000117327286402999)	0	0	0	0
SPATS2L	0	0	0	-1.8864070961134 (0.00895873994304387)	0	0	0	-2.72236964114029 (0.0018628374309139)	0	0
SPC24	0	0	0	0	0	0	0	4.96489012011356 (4.07538366426201e-06)	0	0
SPDL1	0	0	-2.3685252422152 (0.00536234318654578)	0	0	0	0	0	0	0
SPDYA	0	0	-2.44187909911742 (0.00111458384440189)	0	-3.92857066108783 (0.00838991996155355)	0	-1.53443790323665 (0.00111672323818597)	0	0	0
SPEF2	0	0	-2.51159471811619 (0.00353632878673193)	0	0	0	0	0	0	0
SPEG	2.95199585935059 (4.07031822783953e-07)	0	3.78635010328364 (4.98705662292414e-09)	3.26764758496126 (0.00135254773361103)	0	0	0	0	0	0
SPG11	0	0	0	0	-1.5182149899636 (0.00228967101102009)	0	0	0	0	0
SPHK1	-3.47190048641008 (0.000435422525518916)	0	0	0	0	0	0	0	-2.29353857030981 (0.00369664483466877)	0
SPHKAP	0	0	8.42438206752135 (2.92538031596443e-18)	0	0	0	0	0	0	0
SPI1	-2.4409301442057 (1.40640380871086e-06)	0	-2.86448325288253 (0.000215450718192927)	0	-2.29282674425882 (0.00493797578458862)	0	0	0	-2.23918639421264 (5.29674157755061e-08)	0
SPIN1	0	0	0	0	0	0	0	-2.86140037239724 (0.00877105814090702)	0	0
SPIN3	0	4.46785031323827 (0.00330108510820356)	0	0	0	0	0	0	0	0
SPINK1	0	0	0	0	0	8.40998352662277 (2.98564600480746e-08)	0	0	0	0
SPINK5	0	0	0	0	0	0	0	0	2.31183304382422 (0.000682626952616701)	8.37111543840223 (5.37998826432313e-06)
SPINT1	-3.84803133901032 (3.67149489623199e-11)	0	-4.87541716269164 (4.20076943319001e-11)	-3.18570611683882 (0.00170356241114736)	0	0	0	0	1.61548098491031 (0.000519511429318297)	0
SPINT2	-2.84825612395125 (9.06531945293967e-07)	0	0	0	0	0	0	0	0	0
SPIRE1	0	0	0	0	0	0	0	-4.50347300103383 (0.00527653918172252)	0	0
SPIRE2	0	0	0	0	0	2.35221215306038 (0.00546845638909142)	0	0	3.3108265396303 (0.000110229768932392)	0
SPN	-1.96850494098006 (0.00229725398273071)	0	0	0	0	0	0	3.86265797677778 (7.58239110860125e-08)	-3.38575579054274 (2.68807615139113e-08)	-3.50915796328844 (0.00254108382899618)
SPNS2	-1.84247156664955 (0.00590534541614065)	0	0	0	0	0	0	0	-1.89095176406234 (0.00132002266079203)	0
SPOCD1	0	0	-3.2909468394612 (0.00416856390517221)	0	0	0	0	0	0	0
SPOCK1	3.69533292456953 (6.25321023872414e-11)	3.9764233318783 (0.00196978739597693)	2.26517364625892 (0.000497475358433497)	4.97118731298099 (2.49770991499051e-07)	3.04854451953812 (0.00167550006342184)	0	0	0	4.73056297641719 (0.000327696217040587)	3.76865541677945 (0.00034603700842466)
SPOCK2	-1.88117509251848 (0.000560719156124256)	0	0	0	0	0	0	0	0	-3.98397846637026 (3.03443062991548e-05)
SPOCK3	5.18311566531934 (0.00034933677702438)	0	0	0	0	0	0	0	0	0
SPON1	0	0	-2.36603375819939 (6.56468133399355e-08)	0	0	-2.69038910812251 (0.00119089742711367)	0	-6.26115402958446 (6.77777152478673e-05)	0	-2.31376031074549 (0.000359291546250284)
SPON2	0	0	0	0	0	0	0	-3.83959762098027 (0.00563732178969194)	0	0
SPOP	0	0	0	1.70889794566032 (0.00942157264959828)	0	0	0	0	0	0
SPP1	0	0	0	0	4.92880481126037 (2.09164017415661e-07)	0	0	-4.72658273010799 (0.00544130670537274)	3.20574924375463 (1.80298130629634e-10)	0
SPPL2A	0	0	0	0	-2.18814451672496 (0.00563693103769039)	0	0	0	0	0
SPR	0	0	0	0	0	0	0	0	-4.05807272491061 (2.9211065453946e-05)	0
SPRED1	0	0	0	0	0	0	0	-3.01428810009918 (0.00220277795071919)	0	0
SPRN	0	0	0	0	0	3.4783374176854 (0.00100403126658947)	0	0	0	0
SPRR1A	0	0	0	0	0	0	0	0	0	7.89444678885094 (5.12162548990328e-06)
SPRR1B	0	0	0	0	0	0	0	0	0	8.5739717976624 (4.72505102672983e-12)
SPRR2A	0	0	0	0	0	0	0	0	0	6.2971086401216 (0.00274177900224282)
SPRR2E	0	0	0	0	0	0	0	0	0	9.83356149047454 (1.22772446428181e-15)
SPRR2G	0	0	0	0	0	0	0	0	0	10.8944550683675 (2.46866391744304e-19)
SPRR4	0	0	0	0	0	0	0	0	0	6.79009821217895 (0.0026785245388961)
SPRY1	0	0	0	0	0	0	0	-3.0193176042104 (0.00181478218636318)	0	0
SPRY3	0	0	0	0	0	3.96065819821632 (0.00179165525298724)	0	0	0	0
SPRY4	0	0	0	0	0	0	0	-8.14406637031713 (0.000138425279509794)	0	-1.99630228256078 (0.00257330163454149)
SPRYD4	0	0	0	0	0	1.97608805045006 (0.00865681233552112)	0	0	0	0
SPTA1	-3.58880156273726 (0.00190289907395496)	0	0	0	0	0	0	7.46805103438077 (3.70730062579485e-14)	-2.73784298233906 (0.000948282074386189)	0
SPTB	0	0	5.04323975082736 (5.46786099258834e-18)	0	0	0	0	6.79116368823514 (6.61481330321616e-18)	0	0
SPTBN2	0	0	0	0	3.68931757727787 (2.06088881762055e-06)	3.57953056470929 (4.32260670980487e-09)	0	0	0	3.06037535369034 (0.00116928077606157)
SPTBN4	0	0	0	0	0	3.6325000272213 (0.00495233108247664)	0	0	0	0
SPTLC3	0	0	0	0	0	2.52394609684692 (0.00861265811168484)	1.87845612927174 (0.0015560226201397)	0	5.23420478744052 (1.64853124000112e-13)	0
SPTSSB	0	0	0	0	0	0	0	0	0	7.11203065854564 (0.000618894886710335)
SPX	0	0	4.70122143956969 (0.00186558761189099)	0	0	0	0	0	4.90142002077737 (0.000278262756428552)	0
SQLE	-2.32035699515233 (0.00105807964473242)	0	0	0	0	0	0	0	0	0
SQRDL	0	0	0	0	0	0	0	0	-1.99167398131201 (0.00316823464634242)	0
SQSTM1	0	0	0	0	0	0	0	-2.23586400186996 (0.00346021942645937)	0	0
SRCIN1	0	0	0	0	4.20338431861626 (2.74005074857337e-05)	3.30523300855129 (0.00179282267823219)	0	0	0	0
SRD5A1	0	0	0	0	0	2.4899547258386 (0.00410908734652332)	0	0	0	0
SRD5A3-AS1	0	0	0	0	0	4.4186267844504 (0.00559246788192291)	0	0	0	0
SREBF1	-1.5057770066333 (9.05393367792752e-05)	0	0	0	0	1.65123641037534 (0.000644153097275083)	0	0	0	0
SRF	0	0	1.93645949490526 (6.39827185763378e-05)	0	0	0	0	0	0	0
SRGAP1	0	0	0	0	0	0	0	-4.98733938639101 (0.00159710953689039)	0	0
SRGAP3	0	0	0	0	3.12662305542595 (1.0550427456814e-05)	0	0	0	0	0

SRGN	-1.64272846228762 (0.00685404307146576)	-6.34473063129545 (0.00419991568759844)	-1.78207512122909 (0.00201221728501436)	0	-2.23538172262792 (0.00683319586605722)	0	0	0	-2.086868467415 (6.77858310886188e-05)	0
SRL	0	0	10.1259667427792 (3.46021985554009e-34)	0	0	0	0	0	0	0
SRM	0	0	0	0	0	0	0	0	-1.93012261425802 (7.03016758006613e-05)	0
SRP19	0	0	0	0	0	0	1.56980801061585 (0.00387955890839274)	0	0	0
SRP9	0	0	-1.5228061801947 (0.00388517907424131)	0	0	0	0	0	0	0
SRPK3	0	0	6.0931185756817 (1.56306148936289e-07)	0	0	0	0	0	0	0
SRPRB	0	0	0	0	0	0	0	0	0	-3.75928327944231 (0.00535674839893718)
SRRM3	0	0	0	0	4.46936345284741 (0.00341898007228681)	0	0	0	0	0
SRRM4	0	0	0	0	6.1919269522775 (0.00241557134618783)	6.97014476884208 (9.96975360899311e-05)	0	0	0	0
SRRM5	0	0	0	0	0	4.03216340947635 (0.00396125335109937)	0	0	0	0
SRSF10P1	0	0	0	0	0	6.03255896650603 (0.00959365730383889)	0	0	0	0
SSC5D	1.84434226409149 (0.000250651876731919)	0	0	2.96021017704153 (0.000221271161966906)	0	0	0	-7.15884212614489 (0.00126561073499954)	0	0
SSH2	-1.84194483814816 (2.15864346595286e-06)	0	0	0	0	0	0	0	0	0
SSPN	2.05091790561881 (0.000533770373814543)	0	0	0	0	0	0	0	0	0
SSPO	0	0	0	0	3.12132800189079 (0.00165801259592385)	3.80928884978609 (0.0023287972099973)	0	0	0	0
SSR1	0	-5.58080073896683 (0.00937243694479468)	0	0	0	0	0	0	0	0
SSR3	0	0	0	0	-1.75872403657138 (0.00252354124218507)	0	0	0	0	0
SSSCA1-AS1	0	0	0	0	0	4.53529874538245 (0.00412961353093536)	0	0	0	0
SST	5.96897498391665 (3.83551518114986e-06)	0	0	0	0	0	0	0	0	0
SSTR1	0	4.20395245435546 (0.00864787554679088)	0	0	0	0	0	0	0	0
SSTR2	0	0	0	0	5.28300916004504 (2.22209215519659e-05)	5.80701216360444 (5.95480672335263e-06)	4.03494430913532 (0.00148250596112066)	0	0	0
SSTR3	0	0	0	0	6.30139135490905 (0.0080350818762734)	7.91563076759273 (0.000319740889687041)	0	0	0	0
SSTR5-AS1	0	0	6.27031470709689 (4.35210148489933e-06)	0	0	6.53439883271265 (0.00326655861226161)	4.81245669481063 (0.000920290600599386)	0	0	0
SSUH2	0	0	0	0	0	6.66736988147187 (0.00263111335541718)	0	0	0	0
SSX2IP	0	0	0	0	0	0	0	2.43256386770328 (0.00291248110942216)	0	0
ST13	0	0	0	1.81554086519926 (0.00289173913532316)	0	0	0	0	0	0
ST14	-2.07428146093106 (0.00177392814508593)	0	-4.02031275073403 (2.65221958256171e-06)	-3.35668283136051 (0.00767990459563203)	0	0	0	-6.32455227461056 (0.0051790460578648)	0	0
ST18	0	0	0	0	4.16222436179294 (0.00798688042953847)	5.31690335256424 (0.000108729673718992)	0	0	0	0
ST3GAL4	0	0	0	0	0	0	0	-5.85422940698287 (0.00878936329583685)	0	0
ST3GAL5	0	0	-2.43474634202124 (0.00150157794149641)	0	0	0	0	-6.06214917374429 (0.00829018330049467)	0	0
ST3GAL6	0	0	2.69434953022662 (1.84838146673596e-05)	0	0	0	0	0	3.1117435024029 (1.81352166477568e-09)	0
ST6GAL1	0	0	0	0	0	3.65695592345951 (6.83659769360577e-07)	0	0	0	0
ST6GAL2	3.26316323104118 (0.00640162481968161)	0	0	0	0	4.89127341436543 (0.000829336776131004)	0	0	4.15484681244539 (3.15026086857268e-06)	0
ST6GALNAC3	0	0	0	0	0	0	0	0	-2.09468794224122 (0.00117905408343364)	0
ST6GALNAC4	0	0	0	0	0	0	0	0	-2.72181470700622 (8.64996349260916e-06)	0
ST6GALNAC5	0	0	-2.50757601342821 (0.00843261610640559)	0	0	0	0	0	0	0
ST6GALNAC6	0	0	0	0	0	0	0	0	-1.86156536410764 (0.000498242227784015)	0
ST8SIA1	2.47554075504487 (0.00421450320715517)	0	0	0	0	0	0	0	0	0
ST8SIA2	0	0	4.89892226484177 (0.0042251374992457)	0	0	7.23749721837271 (0.00619146838029926)	0	0	0	0
ST8SIA3	5.20727034221058 (9.05502418074632e-05)	0	0	0	0	7.87595553598681 (0.000128262053601168)	0	0	0	0
ST8SIA5	0	0	0	0	5.89834063866756 (4.74807544427369e-05)	6.55902630224349 (7.26142773134218e-06)	0	0	0	0
STAB2	0	0	0	0	0	4.17093771332184 (2.89964379539091e-05)	0	0	0	0
STAC	0	0	0	0	0	0	0	0	-4.26172331797596 (0.000291775825944159)	0
STAC2	0	0	0	0	5.16344286501947 (0.000735792427316459)	0	0	0	0	0
STAG3	0	0	0	0	2.88245495790234 (0.00118840667516913)	3.00351359371693 (0.000978698370584893)	0	0	0	0
STARD10	0	0	1.7600648088104 (0.00186685341800137)	0	0	3.26127953745806 (2.45596495891547e-09)	0	0	0	0
STARD13	1.60317893308416 (2.34342959936134e-06)	0	0	0	0	0	0	0	0	0
STARD4	-2.36913088921376 (1.67210483077204e-05)	0	-1.95738145172735 (0.000673431201936858)	0	0	0	0	0	0	0
STAT1	0	0	-2.11676442740448 (4.58350007164621e-06)	0	0	0	0	0	0	0
STAT2	0	0	0	-1.92515647803535 (0.00457818408832261)	0	0	0	0	0	0
STEAP1	0	0	-4.42927983952674 (1.38718466402493e-06)	0	0	0	0	-5.95170112412779 (0.00756513396025226)	-6.79688344454854 (1.18014944502738e-13)	0
STEAP2	0	0	-1.86756112354108 (0.000717279049017289)	0	-1.71312937022867 (0.00457893246988118)	0	0	-7.90907778464389 (0.000211661991111768)	-5.52087673948083 (4.57907082199949e-21)	0
STEAP3-AS1	0	0	0	0	0	5.34846881497691 (0.00114520249458928)	0	0	0	0
STEAP4	0	0	0	0	0	0	0	-3.13157982081323 (0.000782423462992457)	0	0
STIL	0	0	0	0	0	0	0	4.21900930190623 (0.000221733684558507)	0	0
STIP1	0	0	1.5169203206913 (0.00394305113833928)	0	0	0	1.58300731004787 (0.00791442916165902)	0	0	0
STIP1P3	0	0	0	0	0	6.67639306088117 (0.00313654878531337)	0	0	0	0
STK11	0	0	0	0	0	0	0	2.10822450345046 (0.00126764380378139)	0	0
STK17B	-2.08719651615804 (0.000143130821386535)	0	-2.81618363797463 (7.25878408250461e-07)	0	-2.08170023188666 (0.0037911939736365)	-1.90522383409792 (0.001882790276629)	0	0	0	0
STK26	0	0	-2.10571161688068 (0.00418803152984441)	0	0	0	0	2.70783858531415 (0.00135112064727747)	1.76488627507605 (1.1216952460502e-05)	0
STK32A	0	0	-3.7193425959932 (0.000932531348530505)	0	0	0	0	0	-4.96486500152863 (1.40005800249311e-06)	0
STK38L	0	0	0	2.47782322287874 (0.000312090798249197)	0	0	0	0	0	0
STK39	0	0	0	0	0	0	0	0	-1.61548932733458 (0.00194430579953967)	0
STK4	0	0	-2.42653733654143 (4.1732909209334e-08)	0	0	0	0	0	0	0
STMN1	0	0	0	0	0	0	0	2.49733110341034 (0.00100396672738044)	0	0
STMN2	6.62609852007805 (4.18032565812593e-10)	0	0	7.07811860246089 (5.93844653626063e-06)	0	5.45219528058666 (0.00042327518844502)	0	0	0	0
STMN3	0	3.06436772670215 (0.00864340504300772)	0	0	0	0	0	0	0	0
STON1	0	0	-1.91760298295699 (0.0028479979115883)	0	0	0	0	0	0	0
STOX2	0	0	2.00477870931977 (8.08039414441117e-05)	0	0	0	0	0	0	0
STRA6	0	0	0	0	6.00573681175893 (0.000446537105455914)	0	4.79457100694586 (0.00499143350464887)	0	8.31586119602808 (8.46443497156823e-15)	0
STS	-1.76185496267517 (0.00816830216370097)	0	0	0	0	0	0	0	2.19907928863202 (0.00109726475618158)	0
STUB1	0	0	2.29681072415955 (9.98817916724579e-06)	0	0	0	0	0	-1.52895440930688 (0.00319291941430043)	0
STX11	-3.19783428562263 (3.47339400514587e-05)	0	-2.31626221737396 (0.00109038987334469)	0	0	0	0	0	-2.04608471333232 (0.000365162262457215)	0
STX12	0	0	0	0	0	0	0	-2.54385708097378 (0.00738353466927497)	0	0
STX1B	3.17961319719827 (0.00176684614781383)	0	0	0	5.09124190312263 (0.00138884583919301)	0	3.40012684399729 (0.00959652607437774)	0	0	0
STX3	-1.98503711624389	0	0	-2.60695255810714	0	0	0	0	0	0

	(4.49255420251713e-07)			(0.00194277273255374)						
STX6	0	0	0	0	-2.2819672037702 (0.00717649031149239)	0	0	0	0	0
STXBP1	0	0	0	0	0	0	0	-3.09122526494198 (0.00369188023861932)	0	0
STXBP2	-3.20609354773746 (2.56548710904176e-06)	0	-3.35081549269861 (1.54761223492453e-07)	-3.04322315340882 (0.0033585580559235)	0	0	0	0	0	0
STXBP4	0	0	-1.83002899641792 (0.000282258288779749)	0	0	0	0	0	0	0
STXBP5-AS1	0	6.27694243403626 (0.00010164762003878)	0	0	0	0	0	0	0	0
STYXL1	0	0	0	0	0	0	1.61711108810291 (0.00438714640498107)	0	0	0
SUCLA2	0	0	1.60101585992718 (0.00120446802686772)	0	0	0	0	0	0	0
SUCLG1	0	0	2.49246548780597 (3.14690764134699e-08)	0	0	2.55016932262782 (5.14333319310387e-05)	0	0	0	0
SUCO	0	0	0	0	0	0	0	1.9485158122263 (0.00996797039799485)	0	0
SULF1	1.5646446884932 (6.38345748331926e-05)	0	0	0	0	0	0	0	0	0
SULF2	0	2.57739062121095 (0.000428566829748061)	0	0	0	0	0	0	0	0
SULT1B1	0	0	0	0	0	0	0	0	-2.78157837213186 (7.7689500336158e-05)	0
SULT2A1	0	0	0	0	0	11.6886842909325 (3.18772033041525e-06)	0	0	0	0
SULT2B1	0	0	0	0	3.72367330168653 (0.00840030062744029)	0	0	0	0	4.71632164846761 (0.00161771417416011)
SULT4A1	4.08195224883881 (0.00253391542686557)	0	0	0	0	0	0	0	0	0
SUN5	0	0	0	0	0	7.08201150050981 (0.00710054012903161)	0	0	0	0
SUPT3H	0	0	0	0	0	0	0	0	2.04748827662413 (0.000477134373753216)	0
SUSD1	-1.64784680547308 (0.000454987665230001)	0	0	0	0	0	0	0	0	0
SUSD2	-2.64565735701474 (8.46171315829074e-07)	0	-2.5897137953655 (1.7578366255046e-05)	-4.62511240331028 (0.00024060598635739)	0	0	0	-7.01042112790372 (0.00129756393718569)	-3.03501666417162 (4.77065100753657e-06)	-2.4459811819863 (0.00419501828072425)
SV2A	1.76594440957934 (0.00153646306442902)	0	0	0	0	0	0	0	0	0
SV2B	4.43179648725158 (3.83903810636836e-05)	0	3.98339330679699 (0.00368223266602294)	0	5.67894470482175 (0.000382572318135047)	5.65192010084473 (0.00174498154363092)	4.25450956576981 (0.0028123148503214)	0	3.41589619503061 (0.0018781901721083)	0
SV2C	5.29507423099355 (2.54604376327191e-05)	0	0	0	5.7704815129679 (0.00180325995651682)	7.41486033472136 (3.08232940483079e-06)	0	0	0	0
SVEP1	0	0	-1.65594358480335 (0.000727095449697377)	0	0	0	0	-4.57019125886894 (2.31773764879577e-05)	0	0
SVIL	3.45946546578267 (3.86982533715516e-24)	0	2.70732327206637 (8.07210546663204e-11)	4.18173912464822 (2.64903764886944e-11)	0	0	0	0	0	0
SVILP1	3.93726986825219 (1.10989416378072e-08)	0	4.36969224091441 (8.80137581313415e-06)	4.78240378170005 (0.000244192583586056)	0	0	0	0	0	0
SVOP	0	0	0	0	0	7.11545408144817 (0.000237876156173884)	0	0	0	0
SVOPL	0	0	0	0	0	7.75728083832033 (0.00225511272880956)	0	0	0	0
SYBU	0	0	0	0	0	0	0	0	-2.35669900326056 (0.000769054639937699)	0
SYCP3	2.55898807203555 (0.00446043522939389)	0	0	0	0	3.35029563317693 (0.00517659150011214)	0	0	0	0
SYDE1	0	0	0	0	0	0	0	0	1.86268248510347 (0.000355005349989494)	0
SYDE2	2.43722952405994 (2.76701220396086e-05)	0	0	0	0	0	0	0	0	0
SYK	0	0	-2.81206721727594 (6.84325288239994e-06)	-2.7568957851054 (0.00470180642350771)	0	0	0	2.26522818779835 (0.00423783129123481)	0	0
SYN2	2.68113750223694 (0.000181693979505578)	0	0	3.55703609383186 (0.00753260549148324)	0	0	0	0	0	0
SYNC	2.04391210655484 (0.00532960270579519)	0	0	0	0	0	0	0	0	0
SYNDIG1	0	0	5.04104747470484 (8.37672376637726e-08)	0	0	0	0	0	0	0
SYNE1	0	0	0	0	0	0	0	0	-1.78641417778936 (1.83422414216751e-05)	0
SYNE3	0	0	0	0	0	0	0	0	0	1.97306464683229 (0.00660808127874052)
SYNGR1	2.11294974888163 (0.00452947393221851)	0	2.10794718593941 (0.00222401815450699)	0	2.74635330877178 (0.00235337366737737)	2.542411699034 (0.00483292845202179)	0	0	0	0
SYNJ2	0	0	1.75446055877041 (0.000299052348157337)	0	0	1.5460429127426 (0.00827515197619301)	0	0	0	0
SYNJ2BP	0	0	0	0	0	0	0	-3.16127694747788 (0.00394765692683238)	0	0
SYNM	4.6351350564405 (7.62410874600039e-24)	0	2.87807296312689 (6.7024790903044e-11)	5.55924207989925 (5.89709580058035e-13)	0	0	0	0	0	0
SYNPO	0	0	2.49844647390156 (1.03323926120981e-08)	0	0	0	0	-3.90975576630481 (3.22817887798575e-05)	0	0
SYNPO2	4.36394156162603 (1.19074763284417e-25)	0	1.59310504386254 (3.2255182156844e-07)	4.9802837695761 (6.62144465666878e-15)	0	0	0	-4.74117173135198 (1.06512292026134e-05)	1.8112052102159 (5.43458823351022e-05)	1.73844438745343 (0.00818221674245902)
SYNPO2L	0	0	9.44106048850193 (3.20847386119458e-16)	0	0	0	0	0	0	0
SYP	2.66572777624453 (0.00149476401422022)	0	0	0	0	0	0	0	0	0
SYPL2	2.84558785862587 (0.00842180199819715)	0	4.0520073345588 (4.45042289955313e-06)	0	0	4.82788154688865 (0.00024182174419863)	0	0	0	0
SYT1	7.32321453032997 (2.06389485839056e-10)	0	0	7.35228074738455 (1.21664021102872e-05)	0	5.94337273545499 (0.000219166565607236)	4.09015305305588 (0.0013933250204831)	0	6.32636188746766 (2.61248710689458e-09)	0
SYT12	0	0	0	0	0	6.52785761756674 (2.98746634197935e-08)	4.72680851211584 (0.00767151982682165)	0	3.94561180302198 (0.00139675808048203)	0
SYT13	4.48837334914862 (0.00412362238091154)	0	0	0	0	0	0	0	0	0
SYT14	0	0	0	0	6.95283227746436 (0.00102465732257152)	0	0	0	0	0
SYT16	0	0	0	0	5.36127900956991 (0.00377464716521872)	6.35438856224129 (0.000128613729650291)	0	0	5.20897093149007 (1.12281725307821e-07)	0
SYT17	0	0	0	0	0	2.47060581446683 (0.00740430738121438)	0	0	0	0
SYT2	3.36457075899183 (0.00126097528078983)	0	0	0	0	5.45375224435041 (0.000228475741665436)	3.40862536569452 (0.00238715295397653)	0	2.70028771237554 (0.00734454164884193)	0
SYT3	0	0	0	0	0	7.0048238026879 (0.00773068985945733)	0	0	0	0
SYT4	5.29437731471769 (8.68599661128322e-05)	0	0	0	0	0	0	0	0	0
SYT5	4.20998587840809 (0.00985239964998221)	0	0	0	0	0	0	0	0	0
SYT7	0	0	2.71173963070558 (0.00173914863577329)	0	0	4.1459246852487 (0.000576444442246346)	0	0	3.8448930381784 (0.00406937134530115)	0
SYT8	0	0	0	0	0	0	0	0	3.35553910618971 (2.99801833228794e-05)	0
SYT9	0	0	0	0	0	6.99831452312107 (1.73020725435787e-06)	0	0	0	0
SYTL1	-3.0604111192923 (6.93685654372926e-05)	0	0	0	0	0	0	2.58681378410078 (0.00274345786772463)	0	0
SYTL2	0	0	-2.0476608656672 (0.000460394584663103)	0	0	0	0	0	0	0
SYTL5	0	0	0	0	0	0	0	0	2.85225323311062 (5.04652220651959e-05)	0
T	0	0	0	0	0	7.61084972965719 (0.00271354931107362)	0	0	0	0
TAC3	6.90661012523122 (3.13932618352821e-08)	0	0	0	0	0	0	0	8.41090679990255 (5.51012607804686e-14)	0
TACC1	0	0	0	0	0	-1.61155220799825 (0.00111855821499634)	0	0	0	0
TACC2	0	0	1.84646317660533 (7.98877584469111e-07)	0	0	0	0	-5.3021959504177 (1.24896280214347e-05)	0	0
TACC3	-2.80177581247661 (4.66888677641656e-06)	0	0	0	0	0	0	2.87111434505634 (4.98041764229232e-05)	-1.78002460200589 (0.000493310774786115)	0
TACR2	8.99536282734819 (4.36195831633358e-18)	0	0	9.52683523758567 (3.5259044647731e-14)	0	0	0	0	0	0
TACR3	0	0	0	0	0	6.10213009384402 (0.00755053791667713)	0	0	0	0
TACSTD2	-5.53319227623324 (7.27753233910152e-13)	0	-5.92510414138259 (1.28790327321503e-12)	-8.37142660144102 (0.000141793001824426)	0	-2.2379468427094 (0.00534976993686962)	0	-4.87060066863245 (0.00347318848872612)	0	0
TAF1L	0	0	0	0	0	0	4.94612814228629 (0.00876435635423229)	0	0	0
TAF4B	0	0	0	0	0	0	0	3.47032844542383 (0.00144952379761579)	0	0
TAF9B	0	0	0	0	0	-2.93984489291702 (0.00403990164133809)	0	0	0	0
TAGLN	2.25809317689244 (3.65742129817734e-07)	0	0	2.07719994503962 (0.00682583080155043)	0	0	0	-6.62056947295191 (5.82244443016631e-05)	0	0
TAGLN3	5.14703837614813 (0.00179167218375895)	0	0	0	0	0	0	0	0	0

TAL1	-2.20624157873547 (0.00318585939801743)	0	0	0	0	0	0	3.91107180100419 (1.57899429358112e-06)	0	0
TALDO1	0	0	0	0	0	0	0	2.10288479935119 (0.00278958882060383)	0	0
TANC1	0	0	0	0	0	0	0	-2.50285977997504 (0.000833201901658063)	0	0
TANC2	0	0	-2.0367447466191 (2.60203597947654e-08)	-1.97620445598653 (0.00272808493451441)	0	0	0	0	0	0
TAOK1	0	-6.14199751222971 (0.00369044375230383)	0	0	0	0	0	0	0	0
TAP2	-1.70307836121384 (0.00615888271492255)	0	0	0	0	0	0	0	0	0
TAPBP	0	-5.79785475580371 (0.0070052509424168)	0	0	0	0	0	0	0	0
TAPT1-AS1	0	0	0	0	0	5.52377413135426 (0.000391819567044809)	3.72338418535262 (0.00300952691413569)	0	3.36621415337811 (0.00251184834409753)	0
TARM1	0	0	0	0	0	0	0	6.97712931375118 (0.00214092686965207)	0	0
TARSL2	0	0	1.82950337236985 (0.00100113424812171)	0	0	0	0	0	0	0
TAS1R1	0	0	0	0	0	6.57263684295884 (0.00102204111871329)	0	0	0	0
TAT	0	0	0	0	0	10.2451382902386 (2.90539127220495e-21)	0	0	0	0
TATDN2P2	-2.70562945945262 (0.00148490441477958)	0	-2.49179488740135 (0.00816772912018301)	0	0	0	0	0	-2.17782045828664 (0.000676340309685459)	0
TAZ	-1.6417064653644 (0.00766809351269842)	0	0	0	0	0	0	0	-1.67669976895288 (0.00041485917041876)	0
TBC1D10B	0	2.36039548411332 (0.00734429281745136)	0	0	0	0	0	0	0	0
TBC1D10C	0	0	0	0	0	3.32836648875578 (0.00124137562996251)	0	0	0	0
TBC1D13	0	0	1.59790160927054 (0.00911512755791727)	0	0	0	0	0	0	0
TBC1D16	0	0	0	0	0	0	0	0	-1.85875664257157 (2.8293533201295e-05)	0
TBC1D2	-2.63330177079119 (1.78749132319861e-08)	0	-2.55133944735685 (2.70061242636831e-06)	-4.12766578301096 (0.000181329111696998)	0	0	0	0	-2.44683299874046 (7.10679258996857e-06)	0
TBC1D24	0	0	0	0	0	0	0	0	-1.57541906708425 (0.000636038233776155)	0
TBC1D29	0	0	0	0	0	6.16856019215855 (0.00609985852466005)	0	0	0	0
TBC1D32	0	0	0	0	0	0	0	0	1.52182026728642 (0.00931869094590833)	0
TBC1D3P5	0	0	0	0	0	6.0645547329706 (0.00760120533172173)	0	0	0	0
TBC1D4	0	0	2.35001460999087 (3.02749174014017e-07)	0	0	0	0	0	0	0
TBC1D8	-1.78838647497252 (6.15161002988139e-05)	0	0	0	0	0	0	0	0	0
TBC1D8B	0	0	0	0	0	1.86128546042833 (0.00758911834722205)	0	0	0	0
TBKBP1	0	0	0	0	0	0	0	0	-1.5246605911407 (0.00756273579908605)	0
TBL1XR1	0	-5.71300340198674 (0.00723429050583029)	0	0	0	0	0	0	0	0
TBR1	0	0	0	0	0	7.5389106497497 (0.00386277983424322)	0	0	0	0
TBX15	0	0	4.32940407216112 (0.0035910075301782)	0	0	5.50504962815865 (0.00489925804038256)	0	0	0	6.60431822788938 (0.000836386786641224)
TBX18	0	0	4.42208485139152 (0.000272382222662115)	0	0	0	0	0	4.74300661510157 (1.02855952758839e-06)	0
TBX2	-2.94150929960082 (9.52238633052831e-10)	0	-1.95629286340957 (6.77145593764491e-05)	-4.1888401221471 (5.59222540479297e-06)	0	-2.32530056171698 (0.00171731654562875)	0	-8.38011335420051 (0.000120654481991122)	0	-4.27660053130956 (2.18694153228758e-06)
TBX20	0	0	8.77897891683278 (1.69657423246326e-17)	0	0	0	0	0	5.61761231811007 (6.59438670302663e-05)	0
TBX3	-1.7719088130586 (2.18872152510651e-05)	0	0	0	0	0	0	-5.31830056072293 (0.000920797804661312)	0	0
TBX4	-7.67050712995552 (4.18968248458548e-12)	0	-6.65315074068324 (2.30331000476943e-14)	-8.21310121715769 (0.000161025530361546)	-3.1297981844754 (0.00156915901463322)	0	0	-7.15304872254016 (0.00105141335997304)	-2.22557247589009 (0.000479888766399171)	-8.4926278264175 (9.52307925568944e-05)
TBX5	-7.3939515002023 (1.51945509739143e-11)	0	0	-7.9438339031555 (0.000249331474599715)	-2.4997324838061 (0.00748863797666074)	0	0	-6.88554574138402 (0.00155573626670428)	0	-2.78164447385002 (0.00170530968460632)
TBX5-AS1	-5.36105781482243 (4.53118192155415e-05)	0	0	0	0	0	0	0	0	0
TBXA2R	0	0	0	0	0	3.52602930552713 (0.00226753977386639)	0	0	0	0
TBXAS1	-1.75640303008915 (0.000307453761546568)	0	-2.86410680249964 (5.86883083614878e-05)	0	0	0	0	0	-1.9139442268539 (3.34339901168809e-06)	0
TC2N	0	0	-2.0874355086613 (0.000273572457164387)	0	0	0	0	0	0	0
TCAF1	0	0	0	0	-3.58463150788493 (0.00340081956546466)	0	0	0	0	0
TCAM1P	0	0	0	0	0	7.14261019565052 (0.00718963385642891)	0	0	5.69812350294823 (8.98038397174987e-05)	0
TCAP	0	0	10.4645065511572 (7.16824622305374e-53)	0	0	0	0	0	0	0
TCEA3	0	0	2.4244203675374 (1.00827124144238e-06)	0	0	1.99002520985008 (0.00304924104087739)	0	0	0	0
TCEAL2	2.65556557821203 (5.2415912531145e-06)	0	0	4.53798701300888 (2.08676589406961e-06)	0	0	0	0	0	0
TCEAL3	0	0	0	0	0	-2.0834183496354 (0.00163538952288239)	0	0	0	0
TCEAL4	0	0	0	0	0	-1.60220260715435 (0.00173261997307308)	0	-1.94030993684925 (0.00350048437695794)	0	0
TCEAL5	4.80819744089563 (0.000140499179946733)	0	0	0	0	0	0	0	0	0
TCEAL7	2.62825146625949 (7.67772903176533e-05)	0	0	0	0	0	0	0	0	0
TCEANC	0	0	0	0	0	0	0	0	1.9886139019773 (0.00396202679842418)	0
TCF15	0	0	6.2920690346515 (2.74348040304604e-07)	0	0	0	0	0	0	0
TCF21	-1.96585183221 (9.32386219767772e-05)	0	-2.80310714632907 (1.273371896343e-05)	0	-1.89965189427446 (0.00574749924073949)	-2.55759329428512 (0.00343780702210173)	0	-7.65303529959413 (0.000418628042941552)	0	-4.54967373688183 (7.29100196774333e-06)
TCF23	0	0	0	0	0	6.35231280093571 (0.000377132572749979)	0	0	0	0
TCF4	0	0	0	0	0	0	0	-2.07255598832318 (0.00169402351787746)	0	0
TCF7L2	0	0	0	0	0	0	0	0	1.87678029857395 (0.000156511148717863)	0
TCHH	0	0	0	0	0	4.34067302320937 (0.00134888547954392)	0	0	5.28567577898394 (3.08408252087996e-06)	5.75677728814151 (2.53918887356565e-08)
TCHHL1	0	0	0	0	0	5.70316710991758 (0.000835033546943035)	0	0	0	0
TCIRG1	-1.55325283037891 (0.000288162766662388)	0	0	-2.10157293484306 (0.00850473511331761)	0	0	0	0	0	0
TCL6	0	0	0	0	7.4022293925345 (0.000231644832239065)	7.98148299358198 (2.20364418001713e-05)	6.29234221007186 (0.00383380240609246)	0	8.61749453029419 (4.22420353778039e-07)	0
TCN1	0	0	0	0	0	0	0	7.437750778539 (3.36531112570896e-09)	0	0
TCN2	0	0	0	0	0	0	0	0	-1.75870411502213 (0.00222939717378379)	0
TDG	-1.53041723040181 (0.00655261082459169)	0	0	0	0	0	0	0	0	0
TDGF1	0	0	0	0	7.11396461597731 (0.000122685775547753)	0	0	0	0	0
TDO2	0	0	0	0	0	4.89979753947782 (1.8228650937474e-05)	0	0	-2.79763708323712 (0.00548308856670806)	0
TDP1	0	0	0	0	0	0	0	3.13270503423481 (0.00574613106887045)	0	0
TDP2	0	0	-2.26880539311295 (0.000204318398410701)	0	0	0	0	0	0	0
TDRD1	0	0	0	0	0	6.5990620737568 (0.00240741962572908)	0	0	0	0
TDRD12	0	0	0	0	0	5.68992041452135 (0.00506706388705067)	0	0	0	0
TDRP	0	0	0	0	0	0	0	0	2.62183934819472 (1.59997788729862e-08)	0
TEAD1	0	0	0	0	0	0	0	-4.91376293872964 (4.47812938869411e-05)	0	0
TEAD3	1.70102582045903 (7.07065186920155e-05)	0	0	0	0	0	0	-6.20136152129674 (0.00569221169694369)	0	0
TEAD4	0	0	0	0	0	0	0	0	-2.69261119639765 (1.82150223345345e-05)	0
TEC	0	0	0	0	0	0	0	2.89524083571739 (0.00312757017304523)	0	0
TECRL	0	0	10.2274892140379 (1.38460917454079e-21)	0	0	0	0	0	0	0
TEF	2.40136180283189 (0.000110776974328356)	0	0	0	0	0	0	0	0	0
TEK	-1.94013414107238	0	0	-2.89805597294481	0	0	0	-4.29598624901601	0	0

	(0.000213457696587385)			(0.00206713132194664)				(0.00121677835243637)		
TEKT4P2	0	0	0	0	0	5.5201769506133 (0.000143146309059941)	0	0	0	0
TENM1	0	0	0	0	0	0	0	0	-4.27110904541498 (3.70957120130935e-05)	0
TENM2	0	0	9.01902611321384 (7.77162338895843e-22)	0	5.35177296119855 (0.00147398426996991)	5.88872927153531 (0.000259449288237811)	4.19287682636674 (0.00254699125587383)	0	0	0
TENM3	1.50242871081877 (0.00440393219665435)	0	0	0	0	0	0	0	3.6036354385907 (5.13266450460789e-18)	0
TENM4	0	0	0	0	0	3.18052534306081 (0.00821776624449613)	0	0	0	0
TEPP	0	0	0	0	0	6.83616064796014 (0.00210048989075515)	0	0	0	0
TES	2.29244827282056 (1.39374910667231e-10)	0	0	0	0	0	0	0	0	0
TESC	0	0	3.02763071762856 (0.000375106453847486)	0	0	3.31076249609 (0.00123121622194516)	0	0	0	0
TESPA1	0	0	0	0	0	0	0	3.25312406662559 (0.00867921529052674)	0	0
TET3	-1.59826873362335 (9.17157418561308e-05)	0	-1.91642035776685 (1.92102415364154e-05)	-2.58506800439048 (0.000312424731736119)	0	0	0	1.62023533067096 (0.00817857206797296)	0	0
TEX101	0	0	0	0	0	5.17594239922016 (0.000215873468928819)	0	0	0	0
TEX15	0	0	0	0	6.54579188954221 (0.000256005979761898)	6.28446816679906 (0.00531418671143485)	5.60146432205035 (0.00936689468382057)	0	0	0
TEX26-AS1	0	0	0	0	0	5.16328963994565 (0.00527401554394988)	0	0	0	0
TEX41	0	6.53752645738439 (5.37430371185955e-05)	0	0	0	0	0	0	0	0
TF	3.34533506083031 (0.00656216344800974)	0	0	0	5.61483947657735 (0.000989258517019549)	11.2634067541334 (4.78464505618866e-28)	5.0589581084634 (0.00658228986154607)	0	0	0
TFAP2A	0	0	0	0	4.42550352287615 (0.00389632704504871)	4.50424471061458 (0.0037427517230183)	0	0	8.06860731094138 (1.31647684861164e-30)	7.37665372206289 (5.0037610480572e-11)
TFAP2B	0	0	0	0	0	6.469711239845 (0.00392260125449649)	0	0	0	0
TFAP2C	0	0	0	0	0	0	0	0	4.32178964813601 (1.14160946822704e-13)	4.08530037976975 (0.00185740143472856)
TFCP2L1	-4.61104207060202 (3.78775083857606e-07)	0	-5.25411230530484 (1.45774794942221e-07)	-7.75764383181057 (0.000898916757439086)	0	0	0	-6.69815746880078 (0.00429806116970376)	0	0
TFDP1	0	0	0	0	0	0	0	2.7213686617573 (8.35825231106184e-05)	0	0
TFDP2	0	0	0	0	1.60924373303721 (0.00410800607867966)	0	0	0	0	0
TFEC	-2.75577233629406 (1.03996658616512e-05)	0	-3.65376862080111 (9.49707114190614e-07)	0	0	0	0	0	0	0
TFPI	0	0	-2.14190783865007 (2.52207435463439e-05)	-1.83620273981272 (0.0074724090419628)	0	0	0	-3.12391754140888 (3.0529750309996e-05)	0	0
TFPI2	0	0	0	0	0	0	0	0	6.56391133760873 (4.15758365354181e-07)	0
TFR2	0	0	0	0	0	8.0285401383012 (1.53196793183287e-21)	0	6.3256784314093 (1.08383165668895e-07)	0	0
TFRC	-2.44308187966619 (1.61884259628361e-05)	0	-1.94354176777637 (0.000224718501761229)	0	-2.74716707542136 (0.000317378836775998)	-1.64512807755951 (0.00743865975961795)	0	2.77615002113884 (0.0004445126118107)	0	0
TG	0	5.17053212719753 (0.000185462095687957)	0	0	0	0	0	0	0	0
TGFA	-3.29262861842192 (0.00235951314455544)	0	-3.16263754588086 (0.00468687689019072)	0	0	0	0	0	0	0
TGFB1I1	1.65315312798511 (0.00258207205056202)	0	0	0	0	0	0	0	0	0
TGFB2	0	0	0	0	0	0	0	0	2.0543157902613 (0.000107799339771156)	0
TGFB3	0	0	0	0	0	0	0	0	1.8014772759399 (0.000585665023795598)	0
TGFBI	0	0	0	0	0	0	0	0	1.5754051753543 (3.16071010753026e-06)	0
TGFBR1	0	0	0	0	0	0	0	0	1.7874933082259 (6.96029252818348e-07)	0
TGFBR2	0	0	0	0	0	-1.97607234373574 (5.36056776956959e-05)	0	-2.31525126032274 (0.000124708924896617)	0	0
TGFBR3	0	0	0	0	0	0	0	-3.19417583573807 (0.00188533626020052)	0	0
TGM2	-1.61719559760151 (2.81984980456953e-07)	-4.50005308592618 (0.000150913207848068)	0	-1.78897776387552 (0.00406944842496386)	0	0	0	-2.85130206446619 (1.30225088672654e-05)	0	-3.17993981433896 (4.58992675328095e-07)
TGM3	0	0	0	0	0	0	0	0	0	6.71609651231352 (0.00360897601821362)
TH	0	0	0	0	6.67803863300218 (0.000476355490538224)	0	0	0	0	0
THAP2	3.11954459468057 (7.5772474504422e-05)	0	0	0	0	0	2.29764460658891 (0.00181802233953021)	0	0	0
THAP4	0	0	1.555116689484 (0.00311152248983875)	0	0	0	0	0	0	0
THAP9-AS1	0	0	-2.28053445048852 (0.0043070338138239)	0	0	0	0	0	0	0
THBD	-1.88831423661537 (0.000104394421819838)	0	0	0	-1.86288477984683 (0.00959159728876507)	0	0	-7.95380818417361 (0.00026200549386506)	0	0
THBS1	0	-3.00124104737157 (1.76909634069057e-05)	-1.75917279653737 (0.00298405833825759)	0	-2.03172493371872 (3.99437326178418e-05)	0	0	-3.02397185747785 (3.42105618992087e-06)	0	-2.21118092501319 (0.000475418417490308)
THBS2	0	0	0	0	0	0	0	-3.36516919551918 (3.17843430556409e-05)	-1.73140674648141 (0.00501597291849591)	1.73062573651434 (0.00416855916820013)
THBS4	4.11649167248504 (1.13762692034475e-08)	0	6.44758281736654 (4.41303650224638e-18)	4.93604691666629 (0.000148606041277591)	0	0	0	0	0	0
THEMIS2	-2.49165251934911 (3.46688343516712e-06)	0	-3.2293971412628 (1.2638845033117e-07)	0	0	0	0	0	-1.78883829667011 (0.000291490722872845)	-2.25528434795419 (0.00821529875391778)
THNSL1	0	0	0	0	0	3.4984177824396 (4.43067075665997e-05)	0	0	0	0
THRAP3P1	0	0	0	0	0	4.77923498056216 (0.00502094001908614)	0	0	0	0
THRB	1.84348528390546 (1.75168639690161e-05)	0	0	0	0	1.92221364288107 (0.0050644632450309)	0	0	0	0
THRB-AS1	0	0	0	0	0	5.32661039294077 (0.00321690815660543)	0	0	0	0
THRB-IT1	0	0	4.16017385935996 (0.00616673190694433)	0	0	4.84395535438121 (2.97494310860095e-05)	0	0	0	0
THRSP	0	0	0	0	0	6.50335813769539 (0.0021197772825343)	0	0	0	0
THSD1	-1.91870843558176 (0.0052981993453997)	0	0	0	0	0	0	0	0	0
THSD4	0	0	0	0	0	0	0	-7.20791136031227 (0.000859524636890157)	0	0
THSD7A	0	3.73930712354802 (0.00215494642783995)	0	0	3.49554304311345 (2.52328833597022e-07)	0	0	0	4.94598038018569 (3.63255167956118e-19)	0
THSD7B	4.43003255783065 (4.96456584084537e-05)	0	0	5.87473749305186 (0.000363893524370232)	4.91224883140448 (0.00329807771994563)	0	0	0	0	0
THUMPD1	0	0	0	0	-1.57319319895293 (0.00751783640403595)	0	0	0	0	0
THY1	2.39541646511664 (0.000302338878408143)	0	0	0	0	0	0	0	1.71554498333985 (0.00887554751162695)	0
THYN1	2.13698281535772 (6.808602301025e-05)	0	1.90406599407689 (0.00424021352058443)	0	0	0	0	0	0	0
TIAM1	0	0	0	0	0	0	0	0	1.62897994466673 (0.00139353521591229)	0
TICRR	0	0	0	0	0	0	0	5.80285069072749 (5.05200607565231e-07)	0	0
TIFA	0	0	0	0	0	0	1.51143349192898 (0.00942159244153935)	0	0	0
TIGD1	0	0	0	0	0	3.475440113943 (0.00314252740210245)	0	0	0	0
TIGD2	0	0	0	0	0	3.11321829429665 (1.81815439487661e-05)	0	0	2.47730903280132 (1.20916300246904e-05)	0
TIGIT	0	0	0	0	5.07769757950036 (0.00221983849089542)	0	0	0	0	0
TIMD4	0	0	0	0	0	5.44233372459262 (0.00144012907900347)	0	0	4.65482655082732 (0.000146132545160594)	0
TIMELESS	0	0	0	0	0	0	0	2.68388332280845 (0.00601409357684272)	0	0
TIMM10	0	0	1.72134220624596 (0.00742458002582623)	0	0	0	0	0	0	0
TIMM17B	0	0	0	0	0	1.88695170278325 (0.0036332090278829)	0	0	0	0
TIMM21	0	0	1.58850037580091 (0.00455196048309682)	0	0	0	0	0	0	0
TIMM8B	0	0	1.54267799542625 (0.00131847823958792)	0	0	0	0	0	0	0
TIMMDC1	0	0	0	0	0	-2.80147420587935 (0.00178544318851194)	0	0	0	0
TIMP1	0	0	0	-2.19392359062406 (0.0026739345774623)	0	0	0	-4.76139425194582 (1.82045546280163e-08)	-1.53993282979357 (0.00124489235686327)	0
TIMP2	0	0	0	0	0	0	0	-2.19872226737076 (0.00179882230771317)	0	0
TIMP3	0	0	0	0	0	0	0	-6.667291090672 (0.00272133859746164)	1.68194896016708 (0.00204056215774924)	0

TIMP4	0	4.2066504771499 (0.00517952714286944)	0	0	0	0	0	0	0	0
TINAGL1	0	0	1.90351425835577 (3.38171178795031e-08)	0	0	0	0	0	1.67381232615268 (1.46197881333684e-05)	0
TINCR	0	0	0	0	0	0	0	0	4.76610345391415 (8.43690121899681e-12)	0
TIPARP	0	0	-1.66494950370236 (0.00102020455183367)	0	0	0	0	0	0	0
TJP1	0	0	0	0	0	0	0	-4.28222304583039 (2.12524850309126e-07)	0	0
TJP3	-3.89817208325342 (0.00191880098276839)	0	-3.36540796606375 (0.00808998906904751)	0	0	0	0	0	0	0
TKT	0	0	0	0	0	0	0	0	-2.06082978216756 (2.57260359236902e-10)	0
TLCD2	0	0	0	0	0	0	0	-3.08675138374272 (0.00355879128356624)	-2.19901105675892 (8.01908375901213e-08)	0
TLDC2	0	0	-2.09884414342656 (0.00529804681743316)	0	0	0	0	0	0	0
TLE1	0	0	0	0	0	2.02420974607436 (0.00985295355782346)	0	0	0	0
TLE6	0	0	0	0	0	0	0	0	4.36519201784765 (6.72415906633886e-05)	0
TLL2	0	0	5.47909367391878 (0.000221989477190883)	0	0	7.40376750331562 (0.000102707983999801)	5.29652156392103 (0.00658675901261765)	0	0	0
TLR1	0	0	-3.67610941447358 (0.000275632261850695)	0	0	0	0	0	0	0
TLR2	-2.93681905559094 (1.98247929967937e-08)	0	-3.69751092617506 (1.89960611482199e-08)	-3.69267765087856 (0.000117577742334747)	-3.89426972831868 (0.000167790127959184)	0	0	0	-1.74109581910611 (0.000311215290740864)	0
TLR3	0	0	-2.12180057133773 (0.00892359326858437)	0	0	0	0	0	0	0
TLR7	0	0	0	0	0	0	0	0	3.63472580654651 (6.43173028370543e-07)	0
TLX1	0	0	0	0	0	7.00881329009256 (0.00886510847483269)	0	0	0	0
TM4SF1	0	0	0	0	0	-1.95963973369176 (0.00118241637907173)	0	-4.1599504429275 (0.000125333179696082)	0	0
TM4SF4	0	0	0	0	0	8.21764061334088 (2.94975118828959e-10)	0	0	0	0
TM4SF5	0	0	0	0	0	6.96835631227594 (3.60541039474263e-05)	0	0	0	0
TM7SF2	0	0	2.78147501292503 (5.58257501390949e-05)	0	0	0	0	0	0	0
TMC1	0	0	0	0	0	0	0	0	5.84830370710436 (4.6297424794961e-08)	0
TMC2	0	0	0	0	0	4.94784574577754 (0.00554722108118848)	0	0	0	0
TMC3	0	0	0	0	0	6.65032359453391 (0.000392457591696049)	0	0	0	0
TMC4	-3.5710538005443 (0.000148819858587361)	0	0	0	0	0	0	0	0	0
TMC5	-3.44816044946291 (2.98204333801137e-09)	0	-4.96203746772429 (3.27954540057051e-07)	-7.87772871898961 (0.000322358592273061)	-3.28813574279653 (0.00281754181402369)	0	0	-4.37417503539779 (0.00815342028605537)	-1.85950753813948 (0.00327871151209378)	-2.54528322881497 (0.00569331214791963)
TMC6	-2.49336879804594 (1.01626441131629e-05)	0	0	-4.54715289454824 (0.000498573553152779)	0	0	0	0	-1.58966916366259 (0.000378375745102914)	0
TMC8	0	0	0	0	0	0	0	3.141569092473 (0.0034092701966104)	0	0
TMCC2	0	0	0	0	0	0	0	3.55965528416464 (5.46352166502816e-05)	-2.15091975609287 (0.000196859262456424)	0
TMCO5A	0	0	0	0	0	6.94035970153732 (0.0018861097850123)	0	0	0	0
TMED1	0	0	4.31866130425879 (0.000304422035593741)	0	0	0	0	0	0	0
TMED7	0	0	0	0	0	0	0	-3.43220332461215 (0.00178429429277198)	0	0
TMED8	0	0	-1.84405879321341 (0.000268863045303936)	0	0	0	0	0	0	0
TMEM100	0	0	-4.19967998501363 (4.22772239821615e-06)	0	0	0	0	-7.91683122969328 (0.00753266064215847)	0	0
TMEM116	0	0	0	0	2.35993476542766 (0.00505286672411853)	0	0	0	0	0
TMEM117	0	0	0	0	0	0	0	0	2.20473301292947 (0.00810265376214965)	0
TMEM119	-2.90419357837013 (9.56940421743568e-06)	0	-5.19618167494904 (2.0787119815176e-09)	-7.90749682733307 (0.000419613295502706)	-3.24611722599638 (0.00699848831541651)	0	0	0	-1.83061068201419 (0.00512784218637779)	0
TMEM120A	0	0	1.89342339521259 (0.00589170690341534)	0	0	0	0	0	0	0
TMEM125	-3.80840782580044 (0.00341055076590384)	0	0	0	0	0	0	0	0	0
TMEM126A	0	0	3.34514266328246 (0.00111093669276725)	4.60548786865412 (0.00571016409205443)	0	0	2.29523996520161 (0.00234865012334485)	0	0	0
TMEM130	0	0	0	0	5.02223786820477 (1.5445745903999e-05)	0	0	0	0	0
TMEM132B	4.61198160004863 (0.000523692379778087)	0	0	0	6.46352991553233 (0.000572179705787466)	8.37540970079775 (8.02652224753798e-07)	6.31675751810603 (0.00199005180319921)	0	4.73149108797786 (0.00315062106129175)	0
TMEM132C	0	0	0	0	0	0	0	0	4.23910490469107 (1.05839878859952e-05)	0
TMEM132D	0	0	0	0	6.18218515066015 (0.000987935163834932)	6.88479574321606 (7.31985025055082e-06)	0	0	0	0
TMEM132E	0	0	0	0	0	6.41019867787669 (0.000564956101458213)	0	0	0	0
TMEM134	0	0	2.59621819966774 (0.00336825407308316)	0	0	0	0	0	0	0
TMEM139	0	0	0	0	0	0	0	0	2.1412822351872 (0.00604627706487003)	0
TMEM143	0	0	3.4094405434993 (2.40515081497592e-05)	0	0	0	0	0	0	0
TMEM150C	2.34036793452683 (9.70566396501712e-06)	0	2.47932154764185 (0.000419772671981499)	0	0	0	0	0	0	0
TMEM151A	0	0	0	0	0	7.50034476364749 (0.00335954893947622)	0	0	0	0
TMEM151B	0	0	0	0	0	4.442921736729 (0.00734940665570454)	0	0	0	0
TMEM154	-2.46770874363863 (0.0010026398782931)	0	0	0	0	0	0	0	0	0
TMEM156	0	0	0	0	0	4.75208412917202 (0.00348872772093563)	3.87213095175966 (0.000169599577675626)	0	0	0
TMEM159	0	0	2.32442654543845 (0.000422507975712938)	0	0	0	0	0	0	0
TMEM161A	0	0	0	0	0	0	0	0	-3.00113241704534 (0.00515370250213876)	0
TMEM163	-4.5757716284939 (0.00072113366352667)	0	0	0	0	0	0	0	-2.28196621090049 (0.00862358234750307)	0
TMEM164	-1.73220273597963 (0.00278903150244096)	0	0	0	0	0	0	0	-1.51188958678615 (0.00611389883188179)	0
TMEM165	0	0	0	0	0	-1.78514279238166 (0.00127851265735538)	0	0	0	0
TMEM167A	0	0	0	0	-1.94991715124118 (0.00307568889299106)	0	0	0	0	0
TMEM169	0	0	0	0	0	4.55295869255214 (0.00423034637240975)	0	0	0	0
TMEM17	0	0	0	0	0	5.06080345592914 (0.00329353433554954)	0	0	0	0
TMEM170B	0	0	-2.6387704006932 (0.00284375703057637)	0	0	0	0	0	0	0
TMEM173	0	0	0	-2.51829750420497 (0.00732487261446774)	0	0	0	0	0	0
TMEM176A	0	0	0	0	0	3.13743907273101 (1.2329345122665e-07)	0	-5.86853541137683 (0.00900041526637169)	0	0
TMEM176B	0	0	0	0	0	1.92869709669868 (0.0081779765104022)	0	-4.61685816952261 (0.00580587848833481)	0	0
TMEM178B	2.7465979491822 (0.00915399063323699)	0	5.41287776252856 (2.06350019798137e-09)	0	0	4.7495050433634 (0.00329231599870689)	0	0	0	0
TMEM179	0	0	0	0	0	6.54044405584213 (0.000943839666215635)	0	0	0	0
TMEM18	0	0	0	0	0	1.94629593124298 (0.00790180724149834)	0	0	0	0
TMEM182	0	0	6.21028926625015 (8.4405038702432e-14)	0	0	0	0	0	0	0
TMEM184A	-2.47633080356334 (0.00420302542807872)	0	-3.26083731324237 (0.00202434690991842)	0	0	0	0	0	0	0
TMEM185A	0	0	1.85988010971024 (0.00898184441745417)	0	0	3.2173577611638 (0.000758848509012356)	0	0	0	0
TMEM19	0	0	1.71549746009769 (0.000563611411479236)	0	0	0	1.69420493113648 (0.000146630449369936)	0	0	0
TMEM192	0	0	0	0	0	1.69506213918126 (0.00977855238694858)	0	0	0	0
TMEM196	0	0	0	0	0	8.15318136493862 (0.000231837061293165)	0	0	0	0
TMEM2	-1.67517626141258 (0.000101141910459519)	0	-1.85252683594014 (1.48031834424966e-05)	0	0	0	0	0	0	0
TMEM200A	-2.55499662867925	0	-4.27468347043006	0	0	0	0	0	0	0

	(0.000466427724903939)		(1.01911125532466e-05)							
TMEM200C	0	0	0	0	0	5.04207894146126 (0.000606603981670862)	0	0	0	0
TMEM204	-2.18958040996241 (0.000200403791714853)	0	0	-4.75952890173138 (0.00486978493666596)	0	0	0	-6.1404832638623 (0.00602596836694633)	-2.16303960811326 (0.000542774350266684)	0
TMEM205	0	0	1.75629862850299 (0.00522532400963341)	0	0	0	0	0	0	0
TMEM212	0	0	0	0	5.89964397029407 (0.000980480353925928)	0	0	0	0	0
TMEM213	0	0	0	0	5.73763540622618 (3.17338417834961e-05)	0	0	0	0	0
TMEM243	-1.96694524808663 (0.00535177518356104)	0	0	-5.85726820549533 (0.0098804515122084)	0	0	0	0	0	0
TMEM246	1.92022919403222 (0.00542916402470767)	0	2.36825973643857 (0.00246823713766783)	0	0	0	0	0	0	0
TMEM247	0	0	0	0	0	0	0	0	4.19137607542318 (0.00469257380148457)	0
TMEM25	0	0	0	0	0	2.54921437520769 (0.000748742511459844)	0	0	0	0
TMEM252	3.86699958813184 (0.00780282930588906)	0	0	0	0	0	0	0	0	0
TMEM254	0	0	0	0	0	0	0	0	1.63293217215322 (0.00848239396027947)	0
TMEM255A	4.23307355985906 (0.00136232670260853)	0	0	0	0	0	0	0	0	0
TMEM255B	0	0	0	0	0	0	0	0	-2.15372675087837 (0.0015707739882268)	0
TMEM30A	0	-6.0587672537639 (0.00466695327278368)	0	0	0	0	0	-2.31813322810434 (0.00157421639213934)	0	0
TMEM30B	-2.54506840080829 (0.000228409324887095)	0	-3.22592166052042 (0.00106272847086028)	0	0	0	0	0	0	0
TMEM33	0	0	0	0	-1.97613607816518 (0.00732924318224839)	0	0	0	0	0
TMEM35	6.93896363388096 (1.29113710782199e-10)	0	0	6.58645012042001 (0.000118439698367557)	0	0	0	0	0	0
TMEM39B	0	0	0	0	0	2.9433906347579 (0.00651671973609114)	0	0	0	0
TMEM40	0	0	3.68471208737377 (0.00504610468460763)	0	0	5.66787849250059 (0.00597181433113187)	3.99901580631135 (0.000866952740957193)	0	5.95632457083328 (1.7127455439537e-09)	5.76868422707899 (0.00346344062540423)
TMEM41B	0	0	-1.74440263534366 (0.000730048248478758)	0	0	0	0	0	0	0
TMEM43	0	0	0	0	0	-1.8585004540808 (0.000440893710833976)	0	-4.17063723805934 (0.000516293863463832)	0	0
TMEM44	-2.27289511654188 (0.00450719121417938)	0	0	0	0	0	0	0	0	0
TMEM45A	0	0	0	0	0	3.06711674577849 (1.10710052827218e-05)	0	0	2.9029955140081 (0.000181495493102675)	4.59684007720195 (6.92622010231636e-08)
TMEM47	0	0	0	0	0	0	0	-6.49526438835704 (0.00319008920905141)	0	0
TMEM51-AS1	0	0	0	0	0	4.04022800936757 (0.00551326713353044)	0	0	0	0
TMEM53	0	0	0	0	0	2.80361817144615 (0.00418950197974391)	0	0	0	0
TMEM56	0	0	2.15027786637081 (0.000256191904486791)	0	0	3.9046842454937 (1.32888656831976e-05)	0	3.44118696792861 (0.000744566386412289)	0	0
TMEM63C	0	0	0	0	0	5.31815061607835 (0.00303138288690528)	0	0	0	0
TMEM82	0	0	0	0	0	6.14277459061269 (0.0056177535123712)	0	0	0	0
TMEM86A	0	4.11014457765686 (0.00121838940967145)	0	0	0	0	0	0	0	0
TMEM86B	0	0	0	0	0	4.64010976049128 (0.0093025259984)	0	0	0	0
TMEM87B	0	0	-1.75195064058175 (0.00051522248207068)	0	0	0	0	-2.67644561567728 (0.00528427962196969)	0	0
TMEM88	0	0	3.96844515381213 (1.04126455102812e-05)	0	0	0	0	0	0	0
TMEM8B	1.81081239608841 (0.000101583439627332)	0	0	0	0	0	0	0	0	0
TMF1	0	0	0	0	-1.79418121969212 (0.00018156811128823)	0	0	0	0	0
TMOD1	0	0	3.16417465398254 (1.20811575222459e-10)	0	0	0	0	0	-2.01306769131676 (0.000555266573335901)	0
TMOD2	2.12911136682499 (2.23116192271479e-07)	0	0	2.26668424280561 (0.00258140306793948)	0	0	0	0	0	0
TMPO	0	0	0	0	0	0	0	2.09183243821249 (0.00294163191369735)	0	0
TMPRSS11E	0	0	0	0	0	6.66766415170456 (0.00442862993524995)	0	0	0	0
TMPRSS12	0	0	0	0	5.83004520490158 (0.00374383828643843)	0	0	0	0	0
TMPRSS2	-3.29833468564638 (1.07175231466832e-06)	0	-4.54561406294709 (2.21644099840857e-06)	-3.51829998453397 (0.00787188204487685)	0	0	0	-5.90307984292418 (0.0078915502058229)	-6.32941782492537 (1.25830694302938e-12)	-7.24231156358695 (0.00105775142942478)
TMPRSS3	0	0	0	0	0	3.45682949401097 (0.00859357464339799)	0	0	0	0
TMPRSS4	0	0	0	0	3.29732547306894 (0.000778917234924395)	0	0	0	0	4.06793223002497 (0.000361125883674653)
TMPRSS6	0	0	0	0	0	6.75739337599158 (0.000536925292794979)	0	0	0	0
TMSB4XP8	0	5.59843331686811 (0.00120981017355229)	0	0	0	0	0	0	0	0
TMTC1	1.67551001526739 (1.39867522197567e-05)	0	1.8184493482588 (3.12578420827953e-05)	2.23880036298897 (0.00256848666464656)	0	0	0	0	0	0
TMTC3	0	0	0	0	0	0	0	-3.70324500904185 (0.00263989385427198)	0	0
TMX3	0	0	0	0	-1.89463023982098 (0.00316484965108799)	-1.52773977630038 (0.00272736929397449)	0	0	0	0
TNC	1.68874059152617 (0.00443314766883021)	0	0	0	0	-2.99507543429833 (0.000214165100983033)	0	-4.22972774254152 (2.47849524516828e-05)	0	0
TNFAIP3	-2.03146337213879 (0.000827185342379513)	0	-3.26696834495818 (2.90343453779725e-17)	-2.19113437854419 (0.000602950543732198)	-1.56831104776785 (0.00821994278078679)	0	0	-2.92287963678774 (6.98880338824554e-05)	0	0
TNFAIP8	0	0	-2.39496983153682 (0.000855177479287323)	0	0	0	0	0	0	0
TNFAIP8L1	1.88346541495246 (0.000462020956760445)	0	0	2.68255705052203 (0.00530111879926384)	0	2.64866686144195 (7.51401383329446e-06)	0	0	0	0
TNFAIP8L3	0	0	-2.34845235218401 (0.00121653744906777)	0	0	0	0	0	-3.82762536204824 (1.26265758118089e-08)	0
TNFRSF10B	0	0	0	0	0	0	0	-4.09000123165501 (0.000925350850586509)	-1.59183743700962 (0.000302767504991051)	0
TNFRSF10C	-2.88313392789794 (0.00443786216343778)	0	-3.57565119437955 (0.00153160857313964)	0	0	0	0	0	0	0
TNFRSF10D	0	0	0	0	0	0	0	-5.91668027023526 (0.00819178346755267)	-1.79769342401829 (0.000388769141387427)	0
TNFRSF11A	0	0	0	0	0	3.49614546399655 (0.000617650055128716)	0	0	0	0
TNFRSF11B	-4.82569088432633 (3.3601017013728e-07)	0	0	-6.56578275435219 (0.00300150463211157)	0	0	0	0	0	-6.84602709481637 (0.00195867417594082)
TNFRSF12A	-4.20287133587949 (2.97101244971735e-08)	0	0	0	0	0	0	-6.69672917265611 (0.00302660251310414)	0	0
TNFRSF13B	0	0	0	0	0	0	2.68830185897348 (0.00937727259759947)	4.64940026757609 (0.00829114873195035)	0	0
TNFRSF13C	0	0	0	0	0	3.63067978072396 (0.00517893818535174)	0	0	0	0
TNFRSF14	0	0	-1.54609085722361 (0.00189371596304246)	-3.00486615507081 (0.00208571799151626)	0	0	0	0	-1.81754120018139 (2.85310043869389e-05)	0
TNFRSF19	-3.37283450239564 (1.28572021018206e-05)	0	0	0	0	0	0	0	0	0
TNFRSF21	0	0	-1.74831503466911 (0.00327321661710465)	0	0	0	0	0	0	-2.99920118564912 (0.00491008089727422)
TNFRSF8	0	0	0	0	0	7.23087429744765 (0.00111582692063281)	0	0	0	0
TNFSF10	0	0	0	0	0	0	0	0	3.19091565568744 (1.40049972610707e-08)	0
TNFSF12	0	0	0	0	0	0	0	0	-1.97414268973085 (0.000451982403047764)	0
TNFSF13B	0	0	-3.29829762996927 (6.47182046715956e-05)	-4.63227455480371 (0.00708263496599723)	0	0	0	0	0	-4.91184158031145 (0.00427089053604215)
TNFSF14	0	0	0	0	0	0	0	0	-3.6140782444646 (0.000380248631815952)	0
TNFSF15	0	0	-3.09719320671454 (0.00160628565192084)	0	0	0	0	0	0	0
TNIK	0	0	0	0	0	0	0	0	-2.20153822560951 (2.16003531535229e-08)	-3.27772212206671 (9.12017025936663e-05)
TNIP3	0	0	0	0	0	0	2.51657206226677 (0.00960679659121326)	0	0	0
TNKS1BP1	0	0	0	0	0	0	0	-2.01002474587533 (0.00225736716565692)	0	0
TNKS2	0	0	-1.50244120886954 (1.25341825202881e-05)	0	0	0	0	0	0	0
TNMD	0	0	0	0	0	0	0	0	6.14037586658378 (2.79455733862364e-06)	0

TNNC1	0	0	8.37910196136631 (8.39795065975029e-43)	0	0	0	0	0	0	0
TNNI1	0	0	5.69629351894479 (7.32851969866598e-07)	0	0	5.32189892101479 (0.00132175736984473)	0	0	0	0
TNNI3	0	0	12.0085895590803 (3.13128078188913e-27)	0	0	0	0	0	0	0
TNNI3K	0	0	4.90522984957063 (0.00104502542349257)	0	0	0	0	0	0	0
TNNT1	0	0	6.75639358380693 (6.51968989610029e-10)	0	0	0	0	0	3.35762723533484 (0.00419206449797292)	0
TNNT2	0	0	11.276207639379 (1.37812576476218e-87)	0	0	0	2.24709305766731 (0.0026337785901924)	5.77666516432851 (1.01934891849403e-07)	2.5694104327309 (0.00121599301927029)	0
TNNT3	0	0	7.66716081751104 (4.6324926281176e-14)	0	0	0	0	0	7.22350923038222 (1.6452511377435e-12)	0
TNS1	0	0	0	1.87473265270753 (0.00901509350906171)	0	0	0	0	0	0
TNS3	-1.60756798084724 (0.00014841860069315)	0	0	0	0	0	0	0	0	0
TNS4	0	0	0	0	0	0	0	0	6.15981385734682 (9.91432973497356e-22)	6.73931095013569 (0.00254350696659338)
TOB1	0	0	0	0	0	2.01256128347956 (0.00291224548179772)	0	0	0	0
TOM1L1	0	2.97766663641087 (0.00881607122308641)	0	0	0	0	0	0	-2.06977240181237 (0.000671105359967463)	0
TOM1L2	0	0	3.06779331372392 (1.92047638995661e-10)	0	0	0	0	0	0	0
TOMM22	0	0	1.615025865811 (0.00249194665643504)	0	0	0	0	0	0	0
TOMM5	0	0	1.73202457353811 (0.00742402239371937)	0	0	0	0	0	0	0
TONSL	0	0	0	0	0	0	0	3.85071513024708 (0.00187917146147281)	0	0
TOP1	0	-5.99829137692766 (0.00489553432103528)	0	0	0	0	0	0	0	0
TOP2A	-3.60553668577303 (2.07267259178289e-05)	0	0	-4.12734201521204 (0.00822516495126242)	0	0	0	3.82656761407908 (0.000504191120537153)	0	0
TOP2B	0	0	0	0	0	-1.68835929350762 (0.00155445524436949)	0	0	0	0
TOPBP1	0	0	0	0	0	0	0	1.92992686749454 (0.00665020350116342)	0	0
TOR4A	-1.96440601683744 (0.00145894144350282)	0	0	0	0	0	0	0	-3.19655982356727 (1.01859669050067e-07)	-3.767632397074 (0.0057618911447156)
TOX	2.36201320733746 (0.000496451156388784)	0	0	0	0	0	0	0	0	0
TOX2	-2.79238970617803 (4.07258906137189e-06)	0	-2.88388193461156 (4.71589507360063e-05)	0	0	0	0	-6.08032326094695 (0.00656955196088912)	-3.33668238253734 (1.54205664899228e-07)	-2.97895619511516 (0.0086839008343909)
TOX3	0	0	0	0	0	2.59201153356015 (0.00738301249608314)	0	0	-4.00196403183929 (0.0051043584002531)	0
TP53I3	-2.12881868686358 (0.00388121240419628)	0	0	0	0	0	0	0	0	0
TP53INP1	0	0	0	0	-2.02892036017014 (0.00504443263768831)	0	0	0	0	0
TP53INP2	0	0	1.75650898726127 (0.000339385678381984)	0	0	0	0	-6.54375807241245 (0.00363909375804655)	0	0
TP63	0	0	0	0	0	0	2.78984060720578 (0.00631085857915231)	0	4.34944600273141 (3.10624372336338e-15)	7.12436606318315 (1.98886757086667e-15)
TP73	0	0	0	0	0	0	0	0	-3.76908713149967 (0.00348497389388164)	0
TP73-AS1	0	0	0	0	0	0	0	2.42584893052165 (0.00121904706262704)	0	0
TPBG	0	0	0	0	0	0	0	0	1.8877310864185 (0.00112376337620101)	0
TPD52L1	0	0	1.94028335384494 (0.0025392001300225)	0	2.7776112996822 (0.00583996281086136)	2.26171465450398 (0.00761832829060931)	0	0	0	0
TPH1	0	0	0	0	4.29526466373694 (0.00568451909902245)	0	0	0	0	0
TPM1	3.25020446987285 (4.0270952457132e-19)	0	4.43064485103374 (1.74143161358433e-30)	3.5990604018856 (6.52068908745549e-09)	0	0	0	0	1.65075496712636 (1.48706807179855e-06)	0
TPM2	2.52200589552184 (7.25494574976797e-11)	0	0	3.2505728964209 (6.14688478261793e-06)	0	0	0	-2.23910387441493 (0.00520845565901385)	0	0
TPM3P1	0	0	0	0	0	0	0	0	3.48567250613039 (0.00356699057354824)	0
TPM4	0	0	-1.62062815136462 (0.000148764133014383)	0	0	0	0	-1.68185656090385 (0.0044560860279656)	0	0
TPO	0	0	5.4414161691107 (2.13535876580014e-09)	0	0	0	0	0	0	0
TPP1	0	-6.140855495745 (0.00563586164208658)	-1.55972593706323 (0.00216727951080024)	0	-2.22003059420647 (0.00528211177055506)	0	0	0	0	0
TPRG1	0	0	0	0	0	0	0	0	0	3.06427042257169 (0.00421950596604542)
TPRXL	0	0	0	0	0	6.1033135872854 (0.000377327690589974)	0	0	5.89970121524803 (8.36895265205189e-07)	0
TPSAB1	0	0	0	0	0	0	0	0	-4.10644588246845 (0.000562830311631746)	0
TPST1	0	0	-1.58014660131644 (0.00464690330280548)	0	0	0	0	0	0	0
TPST2	-1.75322054141247 (0.000163883473685088)	0	0	0	0	0	0	0	0	0
TPX2	-2.45929427962223 (0.00990015841637056)	0	0	0	0	0	0	4.1198014515949 (7.73686775835994e-05)	0	0
TRABD2B	0	0	0	0	3.8689640302361 (0.00126849916704963)	4.69269432537331 (5.90521913737551e-05)	0	0	0	0
TRAF3IP2	0	0	0	0	0	0	0	0	0	2.06473867241361 (0.00539901552691227)
TRAF3IP3	0	0	0	0	0	0	0	0	-1.91215738273282 (0.00762674916290102)	0
TRAF4	-3.07055883005649 (0.000478263244607716)	0	0	0	0	0	0	0	0	0
TRAK1	0	0	1.61550922702507 (8.50723557572072e-05)	0	0	0	0	0	0	0
TRAM1	0	0	-1.64233129979126 (2.65286001801241e-05)	0	-1.82658321139244 (0.00485065907009601)	0	0	0	0	0
TRAM2-AS1	0	0	0	0	3.680805293148 (0.00127404968567262)	0	0	0	0	0
TRAPPC5	0	0	0	0	0	3.28406219089398 (0.00681991250706874)	0	0	0	0
TRBC2	0	0	-4.00429059927021 (0.00897950154584215)	0	0	0	0	0	0	0
TRDN	0	0	9.82209153366708 (2.70759104668226e-21)	0	0	0	0	0	0	0
TREH	0	0	0	0	0	4.93614034414593 (0.00646483086838058)	0	0	0	0
TREM1	0	0	-3.27979259313494 (0.000610527637001071)	0	0	0	0	0	0	0
TREML2	0	0	0	0	0	3.69552617058007 (0.00656933047196971)	0	0	0	0
TREML4	-4.57142500651606 (0.00851438547164149)	0	0	0	0	0	0	0	0	0
TRERF1	0	0	0	0	0	0	0	2.3498114277898 (0.00802859344270406)	0	0
TRIB1	-2.33874896087585 (0.000316430776448396)	0	0	0	0	0	0	0	0	0
TRIB3	-3.29433378399304 (0.00207716299893076)	0	0	0	0	0	0	0	-2.69191155691191 (0.00197804875139662)	0
TRIM14	0	0	-2.38898556883942 (0.000355329946397087)	0	0	0	0	0	0	0
TRIM15	0	0	0	0	0	5.89411801530288 (0.00119118719273806)	0	0	0	0
TRIM2	0	0	0	0	0	0	0	-6.30679098557103 (0.003868593211716)	0	0
TRIM22	0	0	-1.64861865500456 (6.31049559950431e-05)	0	0	0	0	0	0	-1.78912988664911 (0.00466445777511569)
TRIM25	0	0	0	-2.05948111680351 (0.00547919329675438)	0	0	0	0	0	0
TRIM27	0	0	0	0	0	0	0	0	0	-1.89008456583076 (0.00901539424728494)
TRIM29	0	0	0	0	0	3.73074701446604 (0.00909530094257861)	2.86686795567701 (0.00374577606506072)	0	5.4046980886847 (4.36730256593637e-21)	7.1350544112655 (9.66155568290486e-17)
TRIM38	0	0	-1.99507714863981 (3.94080520374131e-06)	0	0	0	0	0	0	0
TRIM5	0	0	0	0	0	0	0	0	0	-2.27600566783183 (0.00538107858295686)
TRIM54	0	0	8.30504746505668 (6.30069533008632e-18)	0	0	0	0	0	0	0
TRIM55	0	0	7.11047081584329 (2.05666051340829e-14)	0	0	5.64788605791049 (0.000181843705922124)	0	0	0	0
TRIM58	-2.8530671993544 (0.000222183815871021)	0	0	0	0	0	0	4.47778075079872 (8.88659159295119e-09)	0	0
TRIM63	0	0	10.0778343485992 (1.85244222080912e-18)	0	0	0	0	6.97712931375118 (0.00214092686965207)	0	0
TRIM67	0	0	0	0	7.91277690880897	7.15253914850507	0	0	0	0

					(0.00564017437078207)	(0.00223099795110157)				
TRIM72	0	0	4.20105528320006 (0.00108379966368447)	0	5.17515025496614 (0.000518146535375769)	0	0	0	0	0
TRIM9	0	0	0	0	4.31180472560042 (0.00430072645613084)	3.81451381561872 (0.00337477120209559)	0	0	0	0
TRIML2	0	0	0	0	0	0	0	0	6.0556290381867 (9.97932432930020e-11)	0
TRPC1	1.88088670641194 (0.000247170239139441)	0	0	0	0	0	0	0	0	0
TRPC3	2.45912242521025 (0.00077798812486241)	0	0	0	0	0	0	0	0	0
TRPC5	0	0	0	0	0	5.58441598700003 (0.00488718105191222)	0	0	0	0
TRPC6	0	0	-3.03648986913554 (0.00098358072120056)	0	0	0	0	0	0	0
TRPM3	0	0	0	0	4.6304498496878 (0.000206148953942567)	6.37563960556716 (6.94093322751721e-06)	4.07636812102287 (0.00977903794871721)	0	0	0
TRPM4	-1.50224768607302 (0.000300606643625328)	0	0	0	0	0	0	0	0	0
TRPM6	0	0	0	0	0	5.72838506946248 (0.000318940071971569)	3.87780484497182 (0.00330095157359529)	0	0	0
TRPM8	0	0	0	0	0	8.22637439450015 (1.08602362571512e-07)	0	0	0	0
TRPT1	0	0	0	0	0	2.5562037802047 (9.0858572910244e-05)	0	0	0	0
TRPV1	0	0	0	0	0	3.08639095417574 (0.00713254711837865)	0	0	0	0
TRPV2	-2.42630279327233 (0.000900518253411367)	0	0	0	0	0	0	0	0	0
TRPV3	0	0	0	0	0	5.08120721207893 (0.000234763426461115)	3.83833690524544 (0.00514935582131089)	0	2.6973041634727 (0.00363016641799849)	4.4247548675751 (0.00792175278669872)
TRPV6	0	0	0	0	3.69363653962756 (0.00377815385340654)	4.55278707902017 (0.000123676909099208)	0	0	6.68092260850753 (1.94061637566407e-08)	0
TSEN2	0	4.75974321217456 (6.00170806051661e-06)	0	0	0	0	0	0	0	0
TSEN54	0	3.97823922398238 (0.00517088463036686)	0	0	0	0	0	0	0	0
TSHR	0	0	0	0	0	4.8321099741348 (0.00561499129417)	3.79686196134292 (0.000960083270039929)	0	0	0
TSHZ2	1.93405973352537 (3.5949764475848e-05)	0	0	0	0	2.73626938865332 (9.49441278512208e-08)	0	0	0	2.25390456308835 (0.00791350273213999)
TSKU	0	0	0	0	0	3.91639428224429 (2.39000167711936e-08)	0	0	2.89868476533587 (0.000118841968239666)	0
TSPAN1	-2.48811050832741 (0.005783673855688)	0	-3.5665280965168 (0.00152518108540142)	0	0	0	0	0	0	0
TSPAN11	2.7248325716416 (1.2143399771423e-05)	0	0	2.90748987617145 (0.00663065912508993)	0	0	0	0	0	0
TSPAN13	-2.42208813104064 (1.3845872630646e-06)	0	0	0	0	0	0	0	0	0
TSPAN14	-1.68756198088465 (1.4799733239852e-07)	0	0	-1.73371402766535 (0.00707017595073796)	0	0	0	0	0	0
TSPAN18	2.24054883849348 (3.41033513946278e-07)	0	2.82406631203036 (2.21219978765713e-09)	2.82440586651698 (0.000348911617970434)	0	0	0	0	0	0
TSPAN19	-4.50652854051433 (0.00495187598048468)	0	0	0	0	0	0	0	-4.54048846727822 (0.00153716750356534)	0
TSPAN2	3.25536336221497 (4.33942604863973e-10)	0	0	0	0	0	0	0	0	0
TSPAN3	0	0	-1.75847899946602 (2.1192053892462e-05)	0	0	-1.63326072987639 (0.00542710431789352)	0	-2.88639603202072 (0.00110371343780137)	-1.6089094217945 (1.17891291127989e-05)	-2.52475303285802 (0.0016259119527224)
TSPAN31	0	0	0	0	2.07073423299339 (0.000606861554179239)	0	0	0	0	0
TSPAN32	0	0	0	0	0	0	0	4.65765772276102 (0.00118692805883649)	0	0
TSPAN5	0	0	0	0	0	0	0	0	2.00421572958046 (0.0024212535350179)	0
TSPAN9	0	0	2.06597985312629 (1.37975041483843e-06)	0	0	0	0	0	0	0
TSPEAR-AS2	0	0	0	0	0	4.41065562923594 (0.00479300644477015)	0	0	0	0
TSPYL4	0	0	1.99336355677344 (9.43467472605878e-05)	0	0	0	0	0	0	0
TST	0	0	2.21680779626928 (0.00128722862171131)	0	0	3.93951411551162 (1.20535401418916e-06)	0	0	0	0
TSTA3	0	0	0	0	0	1.97768752144139 (0.00326245203245953)	0	0	0	0
TSTD1	0	0	0	0	0	0	1.81997643602117 (0.00652742426182783)	0	0	0
TTBK1	0	0	0	0	6.5708819469102 (0.000626804841320625)	6.44160776320759 (0.00136047489253677)	0	0	0	0
TTC22	0	0	0	0	0	4.21934323219883 (0.0033613205267304)	0	0	0	0
TTC28	0	0	0	0	0	0	0	-3.25347040572612 (0.00655903116962324)	0	0
TTC30A	2.10649577441575 (0.00694378501496233)	0	0	0	0	0	0	0	0	0
TTC31	0	0	0	0	0	1.79300565751939 (0.00633532412560922)	0	0	0	0
TTC38	0	0	0	0	0	2.65788318904674 (6.49440534446429e-05)	0	0	0	0
TTC39A	-2.69472860875629 (0.00489849486067961)	0	0	0	0	0	0	0	-3.66391760555326 (0.000124448016751982)	0
TTC39B	0	0	-1.97174883745232 (0.00124330791818591)	0	0	0	0	0	0	0
TTC39C	0	0	0	0	0	2.62219696295003 (2.53679368517595e-06)	0	0	0	0
TTC3P1	0	0	0	0	0	2.20368326750638 (0.00940180639656862)	0	0	0	0
TTC6	0	0	0	0	0	3.73134894266425 (0.000730516661193059)	0	0	0	0
TTC7A	-1.70710982706844 (0.00182224894107893)	0	0	0	0	0	0	0	0	0
TTC7B	0	0	0	0	1.68549595159523 (0.00154850580479765)	1.71567768149234 (0.00582949492002677)	0	0	0	0
TTK	0	0	0	0	0	0	0	4.48000684431222 (0.0018406178527742)	0	0
TTLL2	0	0	0	0	0	4.83700968477121 (0.00842366242309067)	0	0	0	0
TTLL3	0	0	-1.76514767972208 (0.000198750362461347)	0	0	0	0	0	0	0
TTLL7	2.3588029946119 (1.4524908860143e-05)	0	0	0	0	0	0	0	0	0
TTN	0	-6.32210335911291 (0.00435027413645249)	7.30117943344806 (6.54563175282914e-43)	0	0	0	0	0	0	0
TTN-AS1	0	0	2.60833618455599 (2.19773585295606e-11)	0	0	0	0	0	0	0
TTPA	0	0	0	0	0	6.52203688020878 (6.83310639627133e-05)	0	0	0	0
TTR	0	0	0	0	0	10.548067738947 (4.48324348287049e-22)	0	0	0	0
TUB	1.62422413632255 (0.00350027697709516)	0	1.76484244004996 (0.00230369038034229)	0	0	0	0	0	0	0
TUBA1A	1.55005499373618 (0.000139896313884848)	0	0	2.10234064028076 (0.00335505829970661)	0	-1.77499660615214 (0.00829943502833342)	0	0	0	0
TUBA3D	0	0	6.36942270476097 (9.64385525289145e-12)	0	0	0	0	0	0	0
TUBA3E	0	0	7.52341182963669 (6.93484012673299e-08)	0	0	0	0	0	0	0
TUBA4A	0	0	2.11150551596117 (0.00153640832090408)	0	0	0	0	0	-2.34894186263994 (0.00129053323572177)	0
TUBB1	-4.06364348474731 (0.00231902648924104)	0	0	0	0	0	0	0	0	0
TUBB2A	1.90379903051002 (0.00264112798914013)	0	0	0	0	0	0	0	0	0
TUBB2B	6.50378625910625 (7.44989844679471e-09)	0	0	6.91881011234931 (0.00173281350374006)	0	0	0	0	0	0
TUBB4A	4.78804599470581 (2.56925980451239e-05)	0	0	0	0	4.9812596927174 (0.00553554549771808)	0	0	0	0
TUBB4B	0	0	1.58148875096351 (2.41862436132746e-05)	0	0	0	0	0	0	0
TUBB6	0	0	0	0	0	0	0	-3.47171193020417 (0.00571492722112784)	0	0
TUBBP1	0	0	0	0	0	0	3.93139302100913 (0.00457050909826635)	0	0	0
TUBG1	0	0	0	0	0	0	0	3.75377575345034 (4.60823355778053e-06)	0	0
TUBG1P	0	0	0	0	0	5.97140023153538 (0.00334569674645343)	0	0	0	0
TUBG2	0	0	0	0	0	0	0	3.62720629886709 (4.55716918665875e-05)	0	0
TUBGCP3	0	0	0	0	0	0	0	2.04610179373791 (0.00939430224311005)	0	0

TUFT1	0	0	-1.93836772527902 (0.00413977833120077)	0	0	0	0	0	0	2.18910925591875 (0.00789952100055418)
TUSC3	0	0	0	0	0	0	0	0	1.7417855240695 (0.00366037108421169)	0
TUSC5	0	0	5.66634966435744 (0.000383230896125424)	0	0	6.16856019215855 (0.00609985852466005)	0	0	0	8.02277179430784 (1.96236870256944e-06)
TVP23A	0	0	0	0	3.78705208843662 (0.00701053354988588)	4.0166709471534 (0.00428876089431117)	0	0	0	0
TWF1	0	0	-1.62927740128734 (0.00237644417957074)	0	0	0	0	0	0	0
TWF2	0	0	1.91069168897653 (0.00238927540711809)	0	0	0	0	0	0	0
TWIST1	0	0	0	0	0	0	0	0	6.18271921290178 (4.69087128455664e-13)	6.45337610625967 (1.53549254893103e-05)
TXLNB	0	0	4.87548548861819 (9.20778065353731e-14)	0	0	0	0	0	0	0
TXNDC12	0	0	0	0	0	-2.50087378690055 (0.00782999636546818)	0	0	0	0
TXNDC17	0	4.21027727612753 (0.000214524046247205)	0	0	0	0	0	0	0	0
TXNDC2	0	0	0	0	0	5.98192862589376 (0.000819118761540727)	0	0	0	0
TXNRD1	-1.60090629978717 (0.000589508073734091)	0	0	0	-1.8181871064297 (0.00368388392816461)	-2.45783026013773 (3.059111587463e-06)	0	-2.26562519397744 (0.00720330607118533)	-2.09284097914042 (8.38513981420766e-08)	-2.22086507230794 (0.00628470680743633)
TXNRD2	-1.55590345762401 (0.00951830145922821)	0	0	0	0	0	0	0	0	0
TXNRD3	2.11109092940403 (0.00541775467345077)	0	0	0	0	0	0	0	0	0
TYK2	-1.50950461233333 (0.00561118695486554)	0	0	0	0	0	0	0	0	0
TYMP	-2.19076607473417 (1.37825873489779e-05)	0	-1.67948184250298 (0.00032653618839406)	-2.64570149748457 (0.0014653081538468)	-2.26332256666918 (0.00808717119754968)	0	0	0	0	0
TYMS	0	0	0	0	0	0	0	3.57758006235098 (0.00190628212727836)	0	0
TYROBP	0	0	-2.98765829335494 (7.50654477370585e-05)	0	0	0	0	0	0	0
TYRP1	0	0	0	0	0	0	0	0	0	4.90333558512121 (0.000488860575494601)
U1	0	0	0	-3.15237109330303 (0.0058941210797544)	0	0	0	0	0	0
U3	0	0	0	0	0	2.94770137231132 (0.00196943280891505)	0	0	0	0
U47924.6	0	0	0	0	0	0	0	0	-3.13872278436923 (0.00902906527826021)	0
U91328.19	2.0133312087337 (0.00779963238848111)	0	0	0	0	2.92227042025589 (0.00778677539593596)	0	0	0	0
UACA	0	0	0	0	0	0	0	-3.01366814398106 (0.000239897325862953)	0	0
UAP1	0	0	0	0	-2.28737932534593 (0.0082455099154492)	0	0	0	0	0
UAP1L1	-3.02171857826831 (4.47882307641832e-05)	0	0	0	0	0	0	0	-1.75705754837291 (0.000417305127580269)	0
UBAC1	0	0	0	0	0	0	0	3.65987607978568 (6.450331230971e-07)	0	0
UBASH3B	-1.73127470063124 (0.00268482858680077)	0	-2.32301569585021 (0.00133176318902652)	0	0	0	0	0	1.94449750420724 (0.000207437321695971)	0
UBB	0	0	0	0	0	1.66201528865108 (0.00331716503476167)	0	0	0	0
UBBP4	0	0	1.90612471021728 (0.0071557299906304)	0	0	0	1.96864954557448 (0.00540805495072311)	0	0	0
UBE2B	0	0	0	0	0	-2.00397492854501 (0.00380025278984496)	0	0	0	0
UBE2D1	0	0	0	0	0	-2.97767349664354 (0.00772709095015494)	0	0	0	0
UBE2G2	0	0	0	0	0	-1.52695057889965 (1.21867598838785e-05)	0	0	0	0
UBE2L2	0	0	0	0	0	0	0	0	4.59153847210885 (0.00369662831151038)	0
UBE2M	0	0	1.57598963356765 (0.000555976638365795)	0	0	0	0	0	0	0
UBE2O	0	0	0	0	0	0	0	2.19796403131794 (0.00740290158384869)	0	0
UBE2QL1	4.58840598275659 (0.000605545731879789)	0	7.35743229438025 (1.96277641916357e-09)	0	0	5.59489895445448 (0.00179322827678931)	0	0	0	0
UBE4A	0	0	0	0	-1.90299782722676 (0.00334493630051495)	0	0	0	0	0
UBIAD1	0	3.97070223137627 (0.00234524126080862)	0	0	0	0	0	0	0	0
UBL4A	0	0	1.62261739359143 (0.00276140666819509)	0	0	0	0	0	0	0
UBLCP1	0	0	0	-3.2337507306036 (0.00657557988456307)	0	0	0	0	0	0
UBR1	0	0	0	0	-1.93772714729943 (0.00121750863506965)	0	0	0	0	0
UBR4	0	-2.71947902813198 (0.00946865121522704)	0	0	0	0	0	0	0	0
UBTD1	0	0	0	0	0	0	0	0	-2.12832859011782 (0.00270480062736113)	0
UBXN11	-2.32694424893001 (0.000479637733018104)	0	0	0	0	0	0	0	-2.2434467256157 (0.0011064825990142)	0
UBXN2B	0	0	-3.02025705964996 (4.66323170156745e-06)	0	0	0	0	0	0	0
UCA1	0	0	0	0	0	0	0	0	6.66513534314016 (1.19625614104682e-09)	0
UCHL1	0	0	0	0	0	0	0	0	-2.746323732725 (0.00667779222929578)	0
UCP3	0	0	0	0	0	4.70686003807078 (0.00192037120968472)	0	0	0	0
UG0898H09	4.3627531041978 (0.00816018425604207)	0	0	0	0	0	0	0	0	0
UGDH	0	0	0	0	0	0	0	-2.98521225158635 (0.0024719358697911)	0	0
UGT1A1	0	0	0	0	0	8.57247821619764 (8.76815169736384e-10)	0	0	0	0
UGT1A4	0	0	0	0	0	6.64654445011188 (0.000328760357262524)	0	0	0	0
UGT1A6	0	0	0	0	0	5.87006862359187 (2.97415607900322e-06)	0	0	0	0
UGT1A9	0	0	0	0	7.15610975261289 (0.000184366331289355)	6.06485551226283 (0.00597842306547675)	0	0	0	0
UGT2A3	0	0	0	0	6.8778274631684 (0.000424573737859399)	7.57688036310577 (9.72058020789714e-06)	0	0	0	0
UGT2B10	0	0	0	0	0	6.55624934414228 (0.00143128437145902)	0	0	0	0
UGT2B15	0	0	0	0	0	8.01098655831655 (7.30722837813973e-07)	0	0	0	0
UGT2B4	0	0	3.77961192033376 (0.00186846020654001)	0	0	0	0	0	0	0
UGT2B7	0	0	0	0	6.92649065266769 (0.00067276942236021)	6.42332869160652 (0.00193968207361189)	0	0	0	0
UGT3A1	0	0	0	0	0	8.65766984645879 (4.00575318085737e-10)	0	0	0	0
UHMK1	0	-5.74293887444808 (0.00814350015189564)	-1.56293367215173 (9.46604352090032e-05)	0	-1.95790284173324 (0.00244850749459227)	0	0	0	0	0
UHRF2P1	0	0	0	0	0	0	0	0	1.82474008035971 (0.00492422523223246)	0
ULBP1	-3.89489659673923 (0.00384543813381523)	0	0	0	0	0	0	0	0	0
UNC13A	0	0	0	0	4.08065415832443 (0.00191921782796448)	5.6636117089218 (0.000509294255164186)	0	0	0	0
UNC13B	0	0	0	0	0	0	0	-2.48843972286196 (0.0034534550802621)	-2.38873693764415 (2.01722240505382e-14)	0
UNC13C	4.16607018548826 (0.00230599146454089)	0	0	5.9944267178639 (0.00891930605663301)	5.83456925213832 (0.00309706562413725)	0	0	0	3.79208572600728 (0.00926568006149939)	0
UNC13D	-3.21324547387425 (5.38971591234122e-08)	0	-2.75814928297584 (4.01133555917762e-06)	0	0	0	0	0	0	0
UNC45B	0	0	7.28112104172793 (1.04931484226832e-11)	0	0	0	0	0	0	0
UNC5B	-2.6128742235346 (6.34271818057894e-06)	0	0	0	0	0	0	-4.25088978121797 (0.00870373577536045)	-2.40042398211549 (3.03112323829602e-06)	-2.78238785717818 (0.00193816070584908)
UNC5C	0	0	0	0	0	0	0	-6.0572148142351 (0.00695420557605743)	0	0
UNC5CL	0	0	0	0	0	3.10196907297624 (0.00204588001698886)	0	0	0	0
UNC5D	0	0	0	0	5.70675754203492 (0.00421749965692642)	6.87255806373517 (0.000551256826074327)	0	0	0	0
UNC79	0	0	0	0	0	5.15950089071667 (0.00444091445758914)	0	0	0	0
UNC80	4.50916564328426 (0.0031695285211302)	0	0	0	6.94195591991278 (0.000902314700617742)	7.43707984036751 (0.000759745408992322)	6.29121564900398 (0.005715619299583)	0	0	0
UNC93B1	0	2.98345191918995	-2.20466914443252	0	0	0	0	0	0	0

		(0.00507358959468207)	(0.00331873401940519)							
UNGP3	0	0	0	0	0	7.14261019565052 (0.00718963385642891)	0	0	0	0
UPB1	0	0	0	0	3.33927572330287 (0.00519755342122285)	4.78249128041543 (3.69859748487517e-09)	0	0	0	0
UPK1B	0	0	0	0	0	5.29469309462522 (0.00613221366740856)	0	0	7.65694719028916 (1.26982481347372e-07)	0
UPP1	-3.04623645099496 (1.6153436066493e-08)	0	-1.71187780734065 (0.00101573053448226)	-3.02398593257218 (0.000509642629244824)	0	0	0	0	0	0
UQCC2	0	0	2.14611704412434 (2.84771011390858e-06)	0	0	0	0	0	0	0
UQCR10	0	0	1.75836311382975 (0.000189363526117533)	0	0	0	0	0	0	0
UQCR11	0	0	2.51189185180558 (8.07805584860586e-09)	0	0	0	0	0	0	0
UQCRC1	0	0	2.35499849369893 (3.50830474063985e-12)	0	0	0	0	0	0	0
UQCRH	0	0	1.99804725986715 (2.34676240608162e-06)	0	0	0	0	0	0	0
UQCRHL	0	0	2.63299277653867 (0.0019851978392406)	0	0	0	0	0	0	0
UQCRQ	0	0	2.16977181996433 (9.2073452183542e-12)	0	0	2.01998141877955 (0.00708614647709562)	0	0	0	0
URAHP	0	0	0	0	0	5.95341613343703 (0.00617839854868693)	0	0	0	0
UROC1	0	0	0	0	0	7.58074101949551 (4.2834530911118e-08)	0	0	0	0
UROD	0	0	0	0	0	0	0	2.84381760458137 (0.000293185873489337)	0	0
USB1	0	0	0	0	0	0	0	0	-1.72120330262143 (0.000233992424271053)	0
USH1C	3.42949948950955 (0.00894592553874223)	0	0	0	0	0	0	0	0	0
USH1G	0	0	0	0	6.47559966567641 (0.0042482603748159)	0	0	0	0	0
USH2A	0	0	4.87242786788645 (0.00412529810595462)	0	6.65447770053204 (0.00254565647500638)	0	6.69743281063623 (0.0010594334848069)	0	0	0
USHBP1	0	0	2.20621701807257 (0.00131535837776864)	0	0	0	0	0	0	0
USP11	0	0	0	2.03640707136198 (0.0053320140281178)	0	0	0	0	0	0
USP13	0	0	2.81969958557667 (7.85525819049636e-08)	0	0	0	0	0	0	0
USP15	0	0	0	0	0	0	0	1.80135850665387 (0.00765237167026245)	0	0
USP2	0	0	3.50096939565939 (1.38873681883934e-07)	0	0	0	0	0	0	0
USP27X	0	0	0	0	0	0	0	0	2.43912267969148 (0.000514551636344939)	0
USP28	0	0	1.83101198919261 (9.34736726155413e-05)	0	0	0	0	0	0	0
USP32P3	0	0	0	0	0	0	0	0	4.04706004677072 (0.00176370517231042)	0
USP33	0	0	0	0	-1.5363322006107 (0.00474018509739795)	0	0	0	0	0
USP35	0	0	0	0	0	2.74919396481072 (0.000849920266120469)	0	0	0	0
USP43	0	0	0	0	0	0	0	0	3.7186960613985 (3.46624584295201e-09)	0
USP46	0	0	1.68040169687232 (0.000742292686275665)	0	0	0	0	0	0	0
USP48	0	0	0	0	0	0	0	1.81894342004997 (0.00907370563611358)	0	0
USP49	0	0	-1.83005261499233 (0.00519372403319494)	0	0	0	0	0	0	0
USP53	-1.60091306258771 (0.000133033234454981)	0	-2.53486276825844 (2.0660885734459e-07)	0	0	-2.73077638183728 (1.2223157982122e-06)	0	-4.00416813383342 (5.55360493804334e-06)	0	0
USP54	0	0	0	0	0	0	0	0	-2.2855806703804 (8.82335456663667e-09)	0
VAMP8	-2.50769076881453 (9.59068977690008e-05)	0	-1.94605869241681 (0.00518587859439409)	-3.47386527779884 (0.00402175215796591)	0	0	0	0	0	0
VASH2	0	0	0	0	0	3.37465214914224 (0.00118417047068841)	0	0	0	0
VASN	-1.98298929146876 (1.2638657490237e-05)	0	0	-2.7602761499214 (0.00156836147233031)	0	0	0	-6.95326036108255 (0.00129259525839201)	0	0
VAT1L	5.23691271876847 (1.8397862609564e-07)	7.7587847397069 (7.80207108727854e-07)	5.24214026395434 (2.00734624725301e-05)	0	4.92024845942125 (0.00799730271179513)	5.42257624543309 (0.000133210068948207)	0	0	0	0
VAV1	0	0	-3.27187679478064 (0.00019462763353945)	0	0	0	0	0	0	0
VAV2	-1.59414507485055 (0.000378083714714219)	0	0	0	0	1.50800338435225 (0.0077783621239066)	0	0	0	0
VAV3	0	0	0	0	0	0	0	0	2.01014450078175 (7.04019479572206e-05)	0
VCAN	-1.85589415703356 (0.000452776557512661)	0	-2.20179976737725 (1.66818141171235e-05)	-1.86701831433928 (0.00548027933265011)	-1.92739082480368 (0.00390920688911623)	0	0	0	0	0
VCL	0	0	0	0	0	-1.60192298726592 (4.09605475091287e-05)	0	0	0	0
VDAC2	0	0	1.55746580607721 (0.000167051385071998)	0	0	0	0	0	0	0
VDAC3	0	2.62694162567793 (0.00549956047631787)	0	0	0	0	0	0	0	0
VDR	-2.35068722605314 (3.52033164836628e-05)	0	-3.23794972421871 (1.11164560536905e-05)	0	0	0	0	0	-2.16384112404702 (1.17921409346735e-05)	0
VEPH1	-6.48724238171661 (1.37615684301374e-07)	0	-5.45591811968763 (3.44323448283546e-07)	0	0	0	0	0	-2.94329869134694 (0.000958877867080034)	-7.3074198577729 (0.00157315578146731)
VGLL1	0	0	0	0	0	0	0	0	7.90376556380226 (2.38201515716598e-15)	0
VGLL3	-1.80611873505074 (9.2962170710761e-05)	0	-3.11810591587236 (3.9299296356302e-13)	0	-2.49751962159545 (0.000181062694015564)	-2.766246084516 (4.32566963017609e-06)	0	-5.86146787469836 (2.91686897656202e-06)	2.18960679376406 (4.16947346101929e-07)	0
VIL1	5.86351875169014 (7.00259404026152e-06)	0	0	0	0	5.89227382385663 (0.00237639932425537)	0	0	0	0
VIP	6.42499197909379 (5.72719823636687e-12)	0	0	5.82807895878162 (0.00102489326465037)	0	0	0	0	0	0
VIPR1	-5.57507357218939 (1.53705086985197e-10)	0	-5.65391315208959 (7.28974595505703e-10)	-6.30801697183038 (0.000426918478224313)	0	0	0	-7.68725580710448 (0.000879559193635001)	-6.03692655938264 (1.32612169310839e-14)	-4.58443905925744 (0.000345267874789883)
VIPR1-AS1	-5.6973635891542 (3.12086521567624e-05)	0	-4.24072718207 (0.000764224865195668)	0	0	0	0	0	-4.44054001378983 (0.000118483608395705)	0
VIPR2	5.93052934427977 (2.85147864492348e-06)	0	6.23156749453899 (6.5684409208053e-08)	0	0	0	0	0	0	0
VIT	0	0	4.49109158412739 (5.25863752543778e-07)	0	0	3.81671677933853 (0.00712608302547651)	0	0	2.94010055066313 (0.00441574234906263)	4.39658223892966 (0.00819269057568707)
VLDLR	0	0	2.26758760728257 (1.59667761461795e-07)	0	0	0	0	0	0	0
VMP1	-1.55465928247901 (0.000841465019080885)	-2.4797830375395 (0.0037496274792176)	-2.3333578396285 (1.94713434212869e-07)	0	-1.50210155435354 (0.00182766748885236)	0	0	-2.32133827379228 (0.00055415808354554)	0	0
VN1R31P	0	0	0	0	0	7.20461390755274 (0.00723935324462451)	0	0	0	0
VN2R3P	0	0	0	0	6.47559966567641 (0.0042482603748159)	0	0	0	0	0
VNN1	-4.04356655825137 (4.37246997683413e-05)	0	-2.74640476238294 (0.00321102707605298)	0	0	0	0	0	-3.13657708061339 (2.76246796686403e-05)	0
VNN2	-4.46124869916441 (0.000163321745163868)	0	0	0	0	0	0	0	-2.85620782220006 (0.00188442982464979)	0
VNN3	-4.11973139067992 (0.00962326624411315)	0	0	0	0	0	0	0	0	0
VPS13A	0	0	0	0	0	0	0	1.85490020126971 (0.00196951263043762)	0	0
VPS13C	0	-3.9836885305767 (0.00998055503520511)	0	0	0	0	0	0	0	0
VPS18	0	0	0	0	0	0	0	0	-1.50603752641515 (0.00073748840496621)	0
VPS37B	-1.92504077112172 (0.000283005960498271)	0	0	0	0	0	0	0	0	0
VPS72	0	3.50571587186063 (0.000267192900122607)	0	0	0	0	0	0	0	0
VRK1	0	0	0	0	0	0	0	3.62237889681124 (0.00242846073077767)	0	0
VSIG10	0	0	0	0	0	2.19801046425489 (0.00670567614612947)	0	0	0	0
VSIG4	0	0	0	0	-2.72328707461003 (0.00770817377277814)	0	0	-3.34560829118599 (0.00434039774468416)	0	0
VSTM1	0	0	0	0	0	0	0	6.80250431028387 (0.000486899312846794)	0	0
VSTM2L	-4.91847751071671 (3.30593245264837e-05)	0	0	-6.11529922030297 (0.00934943566852246)	0	0	0	0	-4.68541491931844 (1.51126174108432e-06)	0
VTN	0	0	4.22314744706647 (6.04242810839304e-05)	0	0	10.6759074937557 (5.82936548471872e-25)	0	0	3.54607877495812 (0.000153403800811233)	0
VTRNA1-1	0	0	0	0	3.57901776511734 (0.00102971339009347)	2.64204511608374 (0.0037093326327183)	0	0	0	-8.09637384049281 (0.00647676250428303)
VTRNA1-2	0	0	0	0	6.56572221988705 (2.41281874276212e-06)	0	0	0	4.28244387053706 (2.01227406369732e-05)	0

VTRNA1-3	0	0	0	0	5.03321109023217 (0.000305802897726587)	0	0	0	0	0
VWA1	1.71551971165333 (0.000153867759942727)	0	0	0	0	1.83765303432499 (0.00150085830481306)	0	-5.78251416678928 (0.0095329027289632)	0	0
VWA3A	-4.23255373995679 (0.00142701496754815)	0	-3.59718118944005 (0.00553270723432263)	0	0	0	0	0	-3.84978080017139 (0.000500909784838573)	0
VWA5A	0	0	-2.8948638429365 (0.000102145262061071)	0	0	0	0	0	0	0
VWA5B1	0	0	0	0	4.5820529829233 (0.000785967216496618)	4.61516028486413 (0.00354275078021173)	0	0	0	0
VWA7	0	0	3.22539885521942 (0.00955090963616698)	0	0	0	0	0	0	0
VWA8-AS1	0	0	0	0	0	6.10213009384402 (0.00755053791667713)	0	0	0	0
VWC2	4.38814583967895 (0.00209213378113973)	0	6.67552422991754 (3.65007437097863e-08)	0	0	6.14240207511957 (0.00915586857540382)	0	0	0	0
VWCE	0	0	0	0	0	4.7599934148918 (1.76929849431736e-05)	0	0	0	0
VWDE	0	0	0	0	0	5.41203020299231 (0.00669580707093687)	3.74756684471218 (0.00547722194128835)	0	0	0
VWF	-1.52478790052627 (0.000103609353776843)	0	0	0	0	-3.76048901927937 (1.87047525430937e-07)	0	-3.93964134546876 (2.00087118060439e-07)	-1.68208743537402 (2.14182984939161e-05)	0
WARS	0	-6.05284968644189 (0.00507361314030917)	0	-1.96110251231249 (0.00694114938301123)	0	-2.02502893234479 (7.75087637310027e-05)	0	-2.40888296607758 (0.00192242346271344)	0	-2.20101875367923 (0.0024801865092748)
WAS	-2.03918645240865 (0.00202273871234537)	0	0	0	0	0	0	0	0	0
WASF1	1.65135600961653 (0.00866142385248105)	0	0	0	0	0	0	0	0	0
WASF3	0	0	0	0	0	0	0	0	-2.54863241360269 (4.12721280543545e-05)	0
WASF3-AS1	0	0	0	0	0	5.70705223914787 (0.00395358567104524)	0	0	0	0
WBP5	0	0	0	0	0	0	0	-2.60731460282211 (0.00156912035392176)	0	0
WBSCR16	0	0	1.52393348749738 (0.000511540944401821)	0	0	0	0	0	0	0
WBSCR17	0	0	2.50388925793449 (0.000181659565090427)	0	0	0	0	0	0	0
WDFY1	0	0	0	-2.02130127433691 (0.00599659282085249)	0	0	0	0	0	0
WDHD1	0	0	0	0	0	0	0	2.90925979559161 (0.00246802448590154)	0	0
WDR11	0	0	-1.72892841907673 (1.22882520071769e-06)	0	0	0	0	0	0	0
WDR11-AS1	0	0	0	0	0	5.66417836507212 (0.00246586349691744)	0	0	0	0
WDR31	0	0	2.17504608035803 (0.00310906841019401)	0	0	0	0	0	0	0
WDR49	0	0	0	0	0	0	0	0	-3.49357419827138 (0.00736023668751097)	0
WDR60	0	2.10963512208701 (0.00918615262879914)	0	0	0	0	0	0	0	0
WDR62	0	0	2.5439818125142 (0.0026986762461817)	0	0	0	0	4.71617734876999 (1.96714815392246e-05)	0	0
WDR66	-2.42148778410769 (0.00421963951192683)	0	-2.93098465129619 (0.00413346617904114)	-6.18770438591518 (0.00939651541868739)	0	0	0	0	0	0
WDR72	0	0	0	0	6.7995615968217 (2.83972380424656e-07)	6.59191304635841 (1.46292121090747e-07)	0	0	0	0
WDR76	0	3.46573116750779 (0.00296491344945345)	0	0	0	0	0	2.90668693526243 (0.00335576204971582)	0	0
WDR86	0	0	0	0	0	0	0	0	2.29660283823689 (0.00986312102578741)	0
WDR87	0	0	0	0	7.51593757119648 (1.45446539806265e-05)	8.14611295305517 (0.00144837432832693)	0	0	0	0
WDTC1	0	0	1.73914193541143 (2.01989043970291e-05)	0	0	0	0	0	0	0
WEE1	0	0	0	0	0	0	0	0	2.84012556056959 (2.76124267761944e-12)	0
WFDC1	0	0	0	0	0	0	0	0	2.20182676279928 (0.000144233766588733)	0
WFDC2	0	0	-4.76250748781184 (6.30702529265974e-06)	0	0	0	0	0	-4.69445661421751 (2.729552503848e-07)	0
WFDC5	0	0	0	0	0	0	0	0	0	8.05314564677364 (1.55286088811466e-06)
WFIKKN2	0	0	0	0	0	7.02540147424054 (0.00154659719608631)	0	0	0	0
WHSC1	0	0	0	0	0	0	0	2.53964921917511 (4.33480276568076e-05)	0	0
WI2-1896O14.1	0	0	4.03600576497951 (9.33260797020577e-07)	0	0	0	0	0	0	0
WIF1	0	0	-3.92123250227532 (0.00671405963611002)	0	0	0	0	0	-5.74529116735338 (8.25478394593644e-06)	0
WIPF1	0	0	-1.50737249976975 (0.000614285312017936)	0	0	0	0	0	0	0
WIPF3	3.66686462707487 (0.000101046371687457)	0	3.5208804094911 (0.000883031517962261)	0	3.85150131823277 (0.00495125071443447)	4.32151393656481 (0.000266079515891457)	0	0	3.17028596601869 (0.00285206274110605)	0
WISP1	-1.89060613342357 (0.00608507005210029)	0	-4.31975149785345 (1.32740765699485e-06)	-3.03962572997476 (0.00900915958458611)	0	0	0	-6.7446518631432 (0.00300296666792927)	-2.13538579481264 (0.000776635715665967)	0
WISP2	-2.95997222019017 (2.2939298858679e-07)	0	-2.00966277545229 (0.000114599770739775)	0	0	0	0	-6.68462697622776 (0.0024765188528303)	-7.53417980563698 (1.0155214436664e-16)	0
WLS	1.97973956046471 (1.48303464769347e-06)	0	0	0	0	0	0	0	0	0
WNK2	5.08074519831287 (2.74413593379717e-21)	0	6.39408295620972 (2.96022467909414e-27)	6.0767084805478 (9.98057684220294e-12)	0	3.62876422157915 (7.94532338993678e-05)	0	0	2.72106441461531 (5.8912270172437e-05)	0
WNK3	0	0	0	0	0	2.55442650564199 (0.00383100179852476)	0	3.12516631648688 (0.00613524908916149)	0	0
WNK4	0	0	0	0	5.45773218349723 (0.000145161882420335)	0	0	0	0	0
WNT10B	0	0	0	0	0	0	0	0	4.55724879382121 (0.00390597011184956)	0
WNT2	-4.53246315713311 (0.0021812518755221)	0	0	0	0	0	0	0	0	0
WNT2B	0	0	-2.2449123677524 (0.00194344327885586)	0	0	0	0	0	0	0
WNT3	0	0	0	0	0	0	0	0	0	4.22426184262237 (0.00311429245264384)
WNT4	0	0	0	0	0	0	0	0	3.37294461714073 (0.0016954639884626)	0
WNT5A	0	0	0	0	0	0	0	0	4.09522959751231 (1.87970174758542e-05)	0
WNT7A	0	0	0	0	0	0	0	0	3.22271697362599 (6.82438752800358e-05)	0
WSB1	0	0	-1.65961491989703 (4.43030635346317e-05)	0	0	-1.60329464308786 (0.00781919049241971)	0	0	0	0
WT1	0	0	5.64692750270185 (1.85380543035394e-06)	0	5.70120859955342 (0.00934464482525645)	0	0	0	0	0
WT1-AS	0	0	5.77985572362888 (5.03021593407882e-05)	0	0	6.0645547329706 (0.00760120533172173)	0	0	0	0
WWC1	-3.83250926568268 (9.06975061398297e-08)	0	-4.00477884439076 (4.51181587946326e-07)	-8.00100444258479 (0.000416824142151227)	0	0	0	-6.9435534928405 (0.00228503597293525)	0	0
WWC2	0	0	0	0	0	0	0	-4.3634526744401 (0.000425075388432139)	0	0
WWC3	0	0	0	0	0	-1.71845924393292 (0.00369461258948561)	0	0	0	0
WWTR1	0	0	0	0	0	-1.50277893397777 (0.00653355494864606)	0	-6.16819376697275 (0.000125408821746718)	0	0
XAF1	0	0	0	-4.29146474208472 (0.0018305009575748)	0	0	0	0	0	0
XAGE2B	0	0	0	0	0	0	0	0	7.04925914483128 (1.6775031407448e-17)	0
XAGE3	0	0	0	0	0	0	0	0	7.79112473941914 (2.17750813840326e-15)	0
XCR1	0	0	0	0	0	6.42936134767581 (0.00118438233517729)	0	0	5.33027024076638 (9.74062603195145e-07)	0
XDH	0	0	0	0	0	4.93843804764283 (0.000103687380424526)	0	0	0	0
XG	0	0	0	0	0	0	0	0	0	6.46186531540019 (0.00125897299487892)
XIRP1	0	0	11.5367566735214 (1.10601630640123e-44)	0	0	4.93822860407438 (0.00174700295126239)	0	6.00276570674932 (7.50194752546891e-07)	0	0
XIRP2	0	0	9.92362463623573 (5.31021678495237e-19)	0	0	4.95165164538599 (0.000435722961636332)	0	0	0	0
XIST	0	0	0	-24.2171284918971 (7.24548480675849e-06)	0	0	0	-22.8705946308579 (2.26632872071669e-05)	0	0
XK	1.8969672466518 (0.00499451090324124)	0	0	0	0	0	0	4.20883150386116 (1.62220826955742e-05)	0	0
XKR4	5.61728009776239 (6.19658026256722e-06)	0	0	0	6.47455491145071 (0.000575384878180951)	8.38000171108927 (8.19598158746937e-08)	6.28212445441956 (0.00162602162056732)	0	0	0
XKR5	0	0	0	0	7.03170776224579 (0.000276967628449891)	7.06958521774959 (0.00112781423995707)	0	0	0	0
XKR7	0	0	0	0	7.15386498749009	7.73114527663028	0	0	0	0

					(0.0046297852324467)	(0.000445673833418136)				
XKRX	-4.71338135681846 (0.000899195002403717)	0	0	0	0	0	0	0	0	0
XPO7	0	0	0	0	0	0	0	2.22016233053934 (0.00108296318882247)	0	0
XPOT	0	0	0	0	-2.20390068504235 (0.000777377531092145)	0	0	0	0	0
XPOTP1	0	0	0	0	0	4.25515471543188 (0.00757613831134697)	0	0	0	0
XRCC2	0	0	0	0	0	0	0	4.23290715118189 (0.000192937335286035)	0	0
XYLB	0	0	0	0	0	2.30509825586269 (0.00271359869864634)	0	0	0	0
YAP1	0	-5.47299984081264 (0.00981328792688339)	0	0	0	-1.74399741119325 (0.00019309794430414)	0	-6.47692981788391 (3.2561345034605e-05)	0	0
YBX1P10	0	0	0	0	0	0	0	0	-2.15657491899408 (0.00117925873893444)	0
YBX1P2	0	6.93748639102443 (2.04821214062385e-06)	0	0	0	0	0	0	0	0
YBX1P6	0	0	0	0	0	5.50894165838026 (0.00441014816172659)	0	0	4.6313588785357 (0.000659711898864783)	0
YBX2	0	0	0	0	0	7.48597982059694 (0.000496492294207373)	0	0	0	0
YBX3	0	0	0	0	0	-1.82702136270225 (0.000386333443160273)	0	0	0	0
YES1	0	0	0	0	0	0	0	-3.27618478023432 (0.000464191825131754)	0	0
YIPF3	0	0	1.58226297167947 (0.000154869701066519)	0	0	0	0	0	0	0
YJEFN3	0	0	-1.68610531911779 (0.00811755068939384)	0	0	0	0	0	0	-3.39508948738779 (0.00505289672220318)
YOD1	-1.72412225384396 (0.000359567391194172)	0	0	-2.69021286274652 (0.0094749751164268)	0	0	0	2.06927764107875 (0.00867535220275908)	0	0
YPEL4	0	0	0	0	0	0	0	3.6136637290317 (0.000148209657451922)	0	0
Y_RNA	0	0	2.20203914706461 (0.00288521044212611)	0	0	0	0	0	0	0
ZAN	0	0	0	0	6.81201551833002 (0.00158736055535963)	6.63090287815528 (0.00154716252638909)	0	0	0	0
ZAR1L	0	0	0	0	6.24434293510046 (0.0075784156376657)	0	0	0	0	0
ZBED2	0	0	0	0	0	0	2.61706284022287 (0.0035882227419679)	0	0	0
ZBED6CL	0	0	0	0	3.58088087557217 (0.00633587294319245)	0	0	0	0	0
ZBP1	0	0	0	0	0	0	2.53374950028984 (0.00134491273835333)	0	0	0
ZBTB16	0	-5.9410959746759 (0.00667622867623668)	0	0	0	0	0	0	-1.60972185622764 (0.00578820248107716)	0
ZBTB17	0	0	0	0	0	0	0	0	-1.51641883566197 (0.00911903928717003)	0
ZBTB26	1.86147567350818 (0.00293307125620164)	0	0	0	0	0	0	0	0	0
ZBTB38	0	0	0	0	0	0	0	-2.25425463594073 (0.000533795690892115)	0	0
ZBTB42	0	0	0	0	0	0	0	0	-2.40880796390601 (0.0099344712682136)	0
ZBTB45	0	0	0	0	0	2.93089459355425 (0.00772154010368426)	0	0	0	0
ZBTB46	0	0	1.6547243908266 (0.00361695827176558)	0	0	0	0	0	0	0
ZBTB47	0	0	3.23921496490294 (9.48021362207257e-09)	0	0	0	0	0	0	0
ZBTB7C	0	4.66291032752921 (2.16049113744768e-05)	0	0	0	0	0	0	0	0
ZBTB8B	0	0	0	0	0	5.59959554753236 (0.00480245449603446)	0	0	0	0
ZC3H12A	-1.90061404376602 (0.00544000467645684)	0	-2.46115857609821 (0.00126992128268591)	0	0	0	0	0	0	0
ZC3H8	0	0	0	0	0	2.34036024057795 (0.00648082441480922)	0	0	0	0
ZCCHC18	0	0	0	0	0	5.50049987851234 (0.00794487596213647)	0	0	0	0
ZCCHC24	1.5736704521239 (1.38613559881384e-05)	0	0	0	0	0	0	0	0	0
ZDBF2	0	0	0	0	0	0	0	0	2.47603528670081 (7.10907420546298e-07)	0
ZDHHC11B	0	0	0	0	0	0	0	0	-2.0661990708955 (0.00654324068306615)	0
ZDHHC19	0	0	0	0	0	4.82705313981018 (0.00240864860638159)	0	0	0	0
ZDHHC20	0	0	-2.05392847575381 (0.000119202864880751)	0	0	-1.79446708301983 (0.00990876840451545)	0	0	0	0
ZDHHC22	0	0	0	0	0	6.89844137680823 (0.00146210936952516)	0	0	0	0
ZDHHC23	0	0	0	0	0	3.44317160107068 (0.000118496237285185)	0	0	0	0
ZDHHC7	0	0	0	0	-1.79029873462403 (0.00741402241604781)	0	0	0	0	0
ZDHHC8P1	0	0	0	0	0	0	0	0	5.38599330388922 (6.75986313316372e-05)	0
ZER1	0	0	1.69576177707755 (0.000549968581411203)	0	0	0	0	0	0	0
ZFAND4	0	0	0	0	0	0	0	2.57192952829577 (0.00708298790020667)	0	0
ZFAS1	0	0	-2.53011352084724 (0.000318392163628281)	0	0	0	0	0	0	0
ZFAT	0	0	0	0	0	0	0	0	3.70600095059689 (1.79005345682621e-10)	0
ZFHX2	0	0	0	0	0	3.30650245369058 (0.000105784948855413)	0	0	0	0
ZFHX3	0	-5.55067087229989 (0.00903022342534597)	0	0	0	0	0	-2.74023264176571 (0.000129054630048383)	0	0
ZFHX4	4.70045611018533 (2.14746394545607e-11)	0	0	4.38093711637537 (0.00235706077161039)	0	5.25062931566815 (1.94453006504824e-09)	3.07188895088789 (0.00273132400626246)	0	0	0
ZFP36	-1.63648723253396 (3.89023242146462e-05)	-2.79000357410541 (0.00494253588152665)	-1.92730822823766 (3.39341571986467e-08)	0	0	0	0	-2.65731072207222 (0.000359570190915608)	0	0
ZFP36L1	0	0	-1.59349965141801 (0.000380208510495929)	0	0	0	0	-2.18575050608689 (0.00161194244683421)	0	0
ZFP41	0	0	0	0	0	0	0	0	-1.78936686350641 (0.00481898257428612)	0
ZFP57	0	0	4.47028094289953 (0.000176841948199037)	0	0	0	0	0	0	0
ZFP64	0	0	0	0	0	2.81966371340897 (0.00277874682431159)	0	0	0	0
ZFPM1	0	0	0	0	0	2.66263326271783 (0.000180186708513357)	0	0	0	0
ZFR2	0	0	0	0	5.44567642195117 (0.00113503061551906)	0	0	0	0	0
ZFYVE16	0	0	0	0	-2.2129653321034 (0.00285217352291528)	0	0	0	0	0
ZG16	0	0	0	0	0	7.44279116366548 (0.00404563973443074)	0	0	0	0
ZGRF1	0	0	0	0	0	0	0	2.94379017045719 (0.00123673588276011)	0	0
ZIC1	0	0	0	0	0	6.06191771491402 (0.000191730490335566)	0	0	0	0
ZIC4	0	0	0	0	0	7.07308077663729 (0.00138932745134119)	0	0	0	0
ZIC5	0	0	0	0	0	6.7484978562393 (0.000163816983004119)	0	0	0	0
ZIM2	0	0	0	0	0	0	0	0	3.83385963277196 (0.00144434083755894)	0
ZMAT3	0	0	-1.68947923232415 (0.000260475925944338)	0	0	0	0	-3.34040067735267 (0.000902226846233002)	0	0
ZMPSTE24	0	0	0	0	0	0	0	-2.88805086127626 (0.00968107076367951)	0	0
ZMYND11	0	0	0	0	0	0	0	-2.89395008459666 (0.00218505899843198)	0	0
ZMYND15	-3.97027330690852 (0.00243931930950911)	0	0	0	0	0	0	0	0	0
ZMYND8	0	0	0	0	0	0	0	1.96001243089882 (0.00964335824799145)	0	0
ZNF10	0	0	0	0	0	2.9650263125172 (0.00483134018512619)	0	0	0	0
ZNF100	0	4.23604872784171 (3.46803471750421e-07)	0	0	0	0	0	0	0	0
ZNF106	0	-5.87418342168075 (0.00573020906892207)	2.66012368197427 (2.70958606005843e-10)	0	0	0	0	0	0	0
ZNF114	0	0	0	0	0	0	0	0	4.22891126125351 (3.49502187671405e-05)	0

ZNF117	0	0	0	0	0	0	0	0	2.3982717145318 (0.000209260835923496)	0
ZNF137P	0	0	0	0	0	0	0	0	2.03999430055928 (0.00497674155132088)	0
ZNF14	0	0	0	0	0	0	0	0	1.57322265231195 (0.00443186940622784)	0
ZNF165	0	0	0	0	0	0	0	0	2.38907585746505 (5.6592521628222e-05)	0
ZNF185	0	0	0	0	0	0	0	0	2.03753063156562 (4.45893462796749e-06)	0
ZNF19	0	0	0	0	0	3.58847627310695 (0.0050718535856889)	0	0	0	0
ZNF205-AS1	0	0	0	0	0	5.6642697251291 (0.00149584174945958)	0	0	0	0
ZNF208	1.75262979258108 (0.00759952109947852)	0	0	0	0	0	0	0	0	0
ZNF213	0	0	0	0	2.54859767442748 (0.00652086289081727)	0	0	0	0	0
ZNF229	0	0	0	0	0	4.04973372844988 (0.00861819313237757)	0	0	0	0
ZNF257	0	0	0	0	0	0	0	0	2.48873119692264 (0.00082950180446007)	0
ZNF267	0	0	-1.88813618495613 (0.0027333870636879)	0	0	0	0	0	0	0
ZNF277	0	2.74906065386022 (0.00411678759671628)	0	0	0	0	0	0	0	0
ZNF28	0	0	0	0	0	0	0	0	1.54900250185114 (0.000523889672172891)	0
ZNF280B	0	0	0	0	0	4.86060191064723 (0.00134768282917417)	0	0	0	0
ZNF295-AS1	0	0	0	0	0	7.0361055957444 (0.000175602624459593)	0	0	0	0
ZNF311	0	0	0	0	0	3.68513959405262 (0.00368896349469487)	0	0	0	0
ZNF316	0	2.88259371155775 (0.000301116953615074)	0	0	0	0	0	0	0	0
ZNF32	1.62160796257977 (0.0057287724442925)	0	0	0	0	0	0	0	0	0
ZNF331	0	0	-2.68878678337813 (1.82590635189892e-08)	0	0	0	0	0	0	0
ZNF354B	0	0	0	0	0	0	0	0	2.09172016922172 (0.000108534010849744)	0
ZNF354C	1.70174824156097 (0.00106573387002242)	0	0	0	0	0	0	0	0	0
ZNF358	0	0	1.85785248702408 (0.00193579932884479)	0	0	0	0	0	-1.67406893996354 (0.0038439589330244)	0
ZNF382	1.99370872237939 (0.00711548221504467)	0	2.30127006569705 (0.000232198636180863)	0	2.91658011244175 (0.00335439190080804)	3.51619265972807 (0.0012475715094576)	1.67082271777986 (0.00940188713978698)	0	2.47659566564849 (7.35110402476707e-06)	0
ZNF385B	-4.05474912026545 (1.95171532068709e-05)	0	-4.73797025384763 (8.95946778221963e-06)	-6.27949615659789 (0.00628337708962056)	0	0	0	0	-3.68653208249988 (2.11269460813649e-05)	0
ZNF385C	0	0	0	0	0	4.82552347350585 (0.00677991782064727)	0	0	0	0
ZNF415	1.8218701925207 (0.00402117787632039)	0	0	0	0	0	0	0	0	0
ZNF423	2.00365970950564 (0.00614795039366046)	0	1.7004055562973 (0.00737662886665521)	0	0	0	0	0	0	0
ZNF438	0	0	0	0	0	-2.40560923865097 (0.0059830000279108)	0	0	0	0
ZNF460	0	0	0	0	-2.02650879211675 (0.00363332800724668)	0	0	0	0	0
ZNF468	0	0	0	0	0	0	0	0	2.47290846122973 (1.34275819676879e-07)	0
ZNF469	-2.78918400700969 (0.00846875698265295)	0	0	0	0	0	0	0	0	0
ZNF474	0	0	0	0	0	0	0	0	-4.27898645815452 (0.00581015202472425)	0
ZNF483	2.2227254750393 (0.000708192325577838)	0	0	0	0	0	0	0	0	0
ZNF486	0	0	0	0	0	0	0	0	1.6289531515146 (8.19109500402495e-05)	0
ZNF488	0	0	0	0	0	0	0	0	4.88614001561367 (0.000172518582026239)	0
ZNF503-AS1	0	0	0	0	0	4.24873090427683 (0.00409731554522186)	0	0	0	0
ZNF503-AS2	0	0	0	0	4.99367394734222 (0.00260004173319772)	4.89704936728423 (0.00315472791371674)	0	0	0	0
ZNF512B	0	0	1.62349992921966 (0.00714022231195333)	0	0	0	0	0	0	0
ZNF519	2.13533324742067 (0.003906973765943)	0	0	0	2.95442442425908 (0.00852045309421113)	0	0	0	0	0
ZNF527	0	0	2.20118074034421 (0.00619097029077563)	0	0	3.40924720654753 (0.00113912232070723)	0	0	0	0
ZNF532	0	0	0	0	0	0	0	-3.87420263243836 (0.00110552349550961)	0	0
ZNF536	3.35953944964477 (0.00499547833673561)	0	0	0	0	0	0	0	0	0
ZNF547	0	0	0	0	0	3.9082625638841 (0.00352030007544964)	0	0	0	0
ZNF552	0	0	0	0	2.57794341170301 (0.00720764445096923)	0	0	0	0	0
ZNF556	0	0	0	0	0	4.85423398120624 (0.000541539804763806)	0	0	0	0
ZNF57	0	0	0	0	0	0	0	0	1.96335182926402 (0.00850899583789978)	0
ZNF576	0	0	0	0	0	2.48777429043341 (0.00880159660510852)	0	0	0	0
ZNF578	0	0	0	0	0	0	0	0	2.4340064616471 (0.00316309897279091)	0
ZNF579	0	5.40491295048605 (1.72240523465553e-06)	0	0	0	2.86487375729459 (0.00878228777414074)	0	0	0	0
ZNF598	0	0	0	0	0	0	0	0	-1.50932314921645 (0.00664455686444033)	0
ZNF599	0	0	0	0	0	3.38267637948599 (0.00819504608888972)	0	0	0	0
ZNF608	0	0	-1.62181496445766 (0.00455385812017553)	0	0	0	0	0	-1.90570216871411 (0.000236793870231318)	0
ZNF611	0	0	0	0	0	0	0	0	1.64538187730757 (8.6507869090459e-06)	0
ZNF614	0	0	0	0	0	0	0	0	1.84174632879213 (0.000260474940046848)	0
ZNF619	0	3.79908065212118 (0.000338151142221294)	0	0	0	0	0	0	0	0
ZNF622	0	0	1.57720728551696 (0.00296592204024211)	0	0	0	0	0	0	0
ZNF638-IT1	0	0	-2.96149320812364 (2.08896049074942e-06)	0	-2.69524299563171 (0.0079522789904828)	-2.56124220057212 (0.00549009759349742)	0	0	0	0
ZNF648	0	0	0	0	0	7.42274282412889 (0.00391418928524199)	0	0	0	0
ZNF662	0	0	0	0	0	0	0	0	2.37503182449249 (1.9257943601491e-07)	0
ZNF674	0	0	0	0	0	2.81267223582279 (0.00255339738183295)	0	0	0	0
ZNF683	0	0	0	0	0	5.83036139806018 (0.00200034770997088)	4.71560264501103 (0.00572867663086585)	0	0	0
ZNF691	0	0	0	0	0	0	1.80523698551299 (0.00875114750051518)	0	0	0
ZNF697	0	0	0	0	0	0	0	-6.49142494291475 (0.00347584365526329)	0	0
ZNF7	0	0	0	0	0	2.80521479139074 (0.000319895020988881)	0	0	0	0
ZNF701	0	4.67513608936998 (5.89359139021505e-07)	0	0	0	0	0	0	0	0
ZNF702P	0	0	0	0	0	0	0	0	3.44847709641464 (1.4409298873615e-07)	0
ZNF703	2.0775276253216 (3.26665258065645e-06)	0	0	0	0	0	0	0	1.88838716699681 (5.22322541841944e-05)	0
ZNF750	0	0	0	0	0	0	0	0	0	4.80711361513886 (4.03845897031821e-06)
ZNF761	0	0	0	0	0	0	0	0	1.63943513447362 (9.1214129981215e-06)	0
ZNF764	0	0	0	0	0	3.00698004354207 (0.00500688892910378)	0	0	0	0
ZNF768	0	0	1.57469554519814 (0.00186036403852779)	0	0	0	0	0	0	0
ZNF771	0	0	0	0	0	4.63315513263204 (0.000282706650059508)	0	0	0	0
ZNF775	0	0	0	0	0	3.54613475358135 (0.00203142077838829)	0	0	0	0
ZNF786	0	0	0	0	0	4.47935750620335 (0.00363363143575034)	0	0	0	0
ZNF813	0	0	0	0	0	0	0	0	2.40412009815452	0

									(5.55381169789555e-08)	
ZNF827	0	0	0	0	1.60917736856061 (0.00866432520760152)	0	0	0	0	0
ZNF83	0	0	0	0	0	0	0	0	1.50757693267883 (2.22655398843189e-07)	0
ZNF830	0	0	0	0	0	0	0	2.69021886763157 (0.00503620721333977)	0	0
ZNF831	0	0	0	0	0	3.26748742981068 (0.00612805832215288)	0	0	0	0
ZNF850	0	0	0	0	0	0	0	0	1.52478821999168 (0.00298906094025361)	0
ZNF853	2.393403306096 (0.000444633123491494)	0	2.19367321908253 (0.000709465944000868)	0	0	0	0	0	0	0
ZNF865	0	0	0	0	0	0	0	0	-1.7172544964365 (0.00359356826719035)	0
ZNF90	0	0	0	0	0	0	0	0	1.84019967695893 (0.00519375896942667)	0
ZNF91	0	0	0	0	0	0	0	0	2.25530548575148 (2.49575162390519e-09)	0
ZNF99	0	0	0	0	0	0	0	0	3.09925118692405 (0.00903286357663931)	0
ZNHIT2	0	0	2.89057435174534 (0.00916068917987862)	0	0	0	0	0	0	0
ZNRF1	0	0	1.54350292996622 (0.00280781988035861)	0	0	2.1881543728523 (0.00331615154288328)	0	0	0	0
ZSCAN16	0	0	0	0	0	0	0	0	-2.26328209770677 (0.00375681718895386)	0
ZSCAN29	0	0	0	0	0	0	0	0	0	-2.32699185655214 (0.00718607432014769)
ZSCAN4	0	0	0	0	0	0	0	0	4.94218462804936 (0.00020772144240972)	0
ZSWIM6	0	0	-1.55719707433164 (6.8524882354431e-05)	0	0	0	0	0	0	0
ZXDB	0	0	0	0	0	2.2739300639868 (0.000312122441039755)	0	0	0	0
ZYX	0	0	0	0	0	-1.55320783018879 (0.00709908841513104)	0	0	0	0
chr22-38_28785274-29006793.1	0	0	0	0	1.67627154207928 (0.00300597588807069)	2.63490474663432 (0.000921555750496419)	0	0	0	0
pk	2.73816453611341 (4.01868622840892e-33)	0	1.91546539840045 (7.94575670145795e-12)	2.81177863117258 (1.3216164251361e-07)	0	0	0	0	0	0
snoU2-30	0	0	0	0	0	4.15659010973271 (0.00650742148677147)	0	0	0	0
