# Supplementary material for: A protein interactions map of multiple organ systems associated with COVID-19 disease
Source: Genomics Inform. 2021 Jun 30;19(2):e14. doi: 10.5808/gi.20078 (PMC8261268; doi:10.5808/gi.20078)
Supplement: Supplementary Table. 2. — Functional annotations and pathways of protein modules. [file gi-20078-suppl2.pdf]

Analysis Type: PANTHER Overrepresentation Test (Released 20200728)

Annotation Version and Release Date: PANTHER version 16.0 Released 2020-12-01

Reference List: Homo sapiens (all genes in database)
Test Type: FISHER
Correction: BONFERRONI

**Cluster 1**						
Genes List:	ABCA1,AURKA,AURKB,CDCA8,CENPU,DMD,INCENP,KIF11,NCAPH,PLK1,PLK2,PTEN,SPAG5,TPX2,SMC2,PRC1,USP13					
Biological Process						
Bonferroni count:	1810					
PANTHER GO-Slim Biological Process	Homo sapiens - REFLIST (20595)	BC_1.txt (17)	BC_1.txt (expected)	BC_1.txt (over/under)	BC_1.txt (fold Enrichment)	BC_1.txt (P-value)
regulation of cytokinesis (GO:0032465)	18	4	0.01	+	> 100	4.15E-06
regulation of cell division (GO:0051302)	21	4	0.02	+	> 100	7.17E-06
cytokinesis (GO:0000910)	50	5	0.04	+	> 100	1.23E-06
membrane fission (GO:0090148)	58	5	0.05	+	> 100	2.47E-06
cell division (GO:0051301)	61	5	0.05	+	99.3	3.14E-06
mitotic spindle organization (GO:0007052)	39	3	0.03	+	93.19	0.00949
microtubule cytoskeleton organization involved in mitosis (GO:1902850)	47	3	0.04	+	77.33	0.0161
mitotic sister chromatid segregation (GO:0000070)	61	3	0.05	+	59.58	0.0341
regulation of cell cycle process (GO:0010564)	110	4	0.09	+	44.05	0.00362
mitotic cell cycle (GO:0000278)	267	7	0.22	+	31.76	2.13E-06
mitotic cell cycle process (GO:1903047)	267	7	0.22	+	31.76	2.13E-06
mitotic nuclear division (GO:0140014)	267	7	0.22	+	31.76	2.13E-06
nuclear division (GO:0000280)	319	7	0.26	+	26.58	7.13E-06
organelle fission (GO:0048285)	340	7	0.28	+	24.94	1.1E-05
regulation of cell cycle (GO:0051726)	207	4	0.17	+	23.41	0.0414
cell cycle process	434	7	0.36	+	19.54	5.72E-05

(GO:0022402)						
cell cycle (GO:0007049)	473	7	0.39	+	17.93	0.000102
Unclassified (UNCLASSIFIED)	9793	7	8.08	-	0.87	0
Molecular Function						
Bonferroni count:	510					
PANTHER GO-Slim Molecular Function	Homo sapiens - REFLIST (20595)	BC_1.txt (17)	BC_1.txt (expected)	BC_1.txt (over/under)	BC_1.txt (fold Enrichment)	BC_1.txt (P-value)
Unclassified (UNCLASSIFIED)	10792	8	8.91	-	0.9	0
Cellular Component						
Bonferroni count:	438					
PANTHER GO-Slim Cellular Component	Homo sapiens - REFLIST (20595)	BC_1.txt (17)	BC_1.txt (expected)	BC_1.txt (over/under)	BC_1.txt (fold Enrichment)	BC_1.txt (P-value)
spindle midzone (GO:0051233)	15	4	0.01	+	> 100	5.33E-07
spindle pole (GO:0000922)	28	4	0.02	+	> 100	4.92E-06
spindle (GO:0005819)	77	6	0.06	+	94.4	1.86E-08
condensed nuclear chromosome (GO:0000794)	48	3	0.04	+	75.72	0.00415
chromosome, centromeric region (GO:0000775)	55	3	0.05	+	66.08	0.00612
condensed chromosome (GO:0000793)	69	3	0.06	+	52.67	0.0118
chromosomal region (GO:0098687)	83	3	0.07	+	43.79	0.02
centrosome (GO:0005813)	134	4	0.11	+	36.16	0.00187
centriole (GO:0005814)	149	4	0.12	+	32.52	0.00282
microtubule organizing center (GO:0005815)	194	4	0.16	+	24.98	0.00781
microtubule cytoskeleton (GO:0015630)	432	6	0.36	+	16.83	0.000395
cytoskeleton (GO:0005856)	667	6	0.55	+	10.9	0.00472
Unclassified (UNCLASSIFIED)	9302	6	7.68	-	0.78	0
Pathway						
Bonferroni count:	156					
PANTHER Pathways	Homo sapiens - REFLIST (20595)	BC_1.txt (17)	BC_1.txt (expected)	BC_1.txt (over/under)	BC_1.txt (fold Enrichment)	BC_1.txt (P-value)
Unclassified (UNCLASSIFIED)	17977	16	14.84	+	1.08	0
**Cluster 2**						
Genes List:	ABL1,ABL2,CAV1,CAV2,HBA1,HBB,HSPA1A,MYRIP,NOS3,PRKAR2A,PRKAR2B,RAB27B,R					

	IN1,PTGS2,SH2D3C,TRPC1,VAV2,ERN1,AKAP13,MAP2,PRKACA					
Biological Process						
Bonferroni count:	1810					
PANTHER GO-Slim Biological Process	Homo sapiens - REFLIST (20595)	BC_2.txt (24)	BC_2.txt (expected)	BC_2.txt (over/under)	BC_2.txt (fold Enrichment)	BC_2.txt (P-value)
intracellular signal transduction (GO:0035556)	786	8	0.92	+	8.73	0.00359
Unclassified (UNCLASSIFIED)	9793	5	11.41	-	0.44	0
Molecular Function						
Bonferroni count:	510					
PANTHER GO-Slim Molecular Function	Homo sapiens - REFLIST (20595)	BC_2.txt (24)	BC_2.txt (expected)	BC_2.txt (over/under)	BC_2.txt (fold Enrichment)	BC_2.txt (P-value)
kinase binding (GO:0019900)	196	5	0.23	+	21.89	0.00156
protein kinase binding (GO:0019901)	176	4	0.21	+	19.5	0.0266
small molecule binding (GO:0036094)	397	8	0.46	+	17.29	5.89E-06
nucleoside phosphate binding (GO:1901265)	254	5	0.3	+	16.89	0.00536
nucleotide binding (GO:0000166)	254	5	0.3	+	16.89	0.00536
carbohydrate derivative binding (GO:0097367)	272	5	0.32	+	15.77	0.00742
protein serine/threonine kinase activity (GO:0004674)	349	5	0.41	+	12.29	0.024
anion binding (GO:0043168)	500	6	0.58	+	10.3	0.00999
enzyme binding (GO:0019899)	703	7	0.82	+	8.54	0.00589
protein binding (GO:0005515)	2640	12	3.08	+	3.9	0.00615
Unclassified (UNCLASSIFIED)	10792	8	12.58	-	0.64	0
Cellular Component						
Bonferroni count:	438					
PANTHER GO-Slim Cellular Component	Homo sapiens - REFLIST (20595)	BC_2.txt (24)	BC_2.txt (expected)	BC_2.txt (over/under)	BC_2.txt (fold Enrichment)	BC_2.txt (P-value)
perinuclear region of cytoplasm (GO:0048471)	45	3	0.05	+	57.21	0.0101
Unclassified (UNCLASSIFIED)	9302	8	10.84	-	0.74	0
Pathway						
Bonferroni count:	156					
PANTHER Pathways	Homo sapiens - REFLIST (20595)	BC_2.txt (24)	BC_2.txt (expected)	BC_2.txt (over/under)	BC_2.txt (fold Enrichment)	BC_2.txt (P-value)
Histamine H2	25	3	0.03	+	> 100	0.000695

receptor mediated signaling pathway (P04386)						
Enkephalin release (P05913)	35	3	0.04	+	73.55	0.00178
GABA-B receptor II signaling (P05731)	36	3	0.04	+	71.51	0.00192
Endothelin signaling pathway (P00019)	85	6	0.1	+	60.57	1.23E-07
Beta2 adrenergic receptor signaling pathway (P04378)	47	3	0.05	+	54.77	0.00409
Beta1 adrenergic receptor signaling pathway (P04377)	47	3	0.05	+	54.77	0.00409
5HT1 type receptor mediated signaling pathway (P04373)	48	3	0.06	+	53.63	0.00434
Metabotropic glutamate receptor group II pathway (P00040)	49	3	0.06	+	52.54	0.0046
Dopamine receptor mediated signaling pathway (P05912)	59	3	0.07	+	43.63	0.00781
Muscarinic acetylcholine receptor 2 and 4 signaling pathway (P00043)	61	3	0.07	+	42.2	0.0086
Metabotropic glutamate receptor group III pathway (P00039)	70	3	0.08	+	36.78	0.0127
Unclassified (UNCLASSIFIED)	17977	12	20.95	-	0.57	0
**Cluster 3**						
Genes List:	ACTA1,BAIAP2,GSN,HIP1R,MTSS1,SPIRE1,GC,VCL					
Biological Process						
Bonferroni count:	1810					
PANTHER GO-Slim Biological Process	Homo sapiens - REFLIST (20595)	BC_3.txt (9)	BC_3.txt (expected)	BC_3.txt (over/under)	BC_3.txt (fold Enrichment)	BC_3.txt (P-value)
positive regulation of cellular component organization (GO:0051130)	139	3	0.06	+	49.39	0.0473
Unclassified (UNCLASSIFIED)	9793	4	4.28	-	0.93	0
Molecular Function						
Bonferroni count:	510					
PANTHER GO-Slim Molecular Function	Homo sapiens - REFLIST (20595)	BC_3.txt (9)	BC_3.txt (expected)	BC_3.txt (over/under)	BC_3.txt (fold Enrichment)	BC_3.txt (P-value)
Unclassified (UNCLASSIFIED)	10792	6	4.72	+	1.27	0
Cellular Component						
Bonferroni count:	438					

PANTHER GO-Slim Cellular Component	Homo sapiens - REFLIST (20595)	BC_3.txt (9)	BC_3.txt (expected)	BC_3.txt (over/under)	BC_3.txt (fold Enrichment)	BC_3.txt (P-value)
Unclassified (UNCLASSIFIED)	9302	4	4.06	-	0.98	0
Pathway						
Bonferroni count:	156					
PANTHER Pathways	Homo sapiens - REFLIST (20595)	BC_3.txt (9)	BC_3.txt (expected)	BC_3.txt (over/under)	BC_3.txt (fold Enrichment)	BC_3.txt (P-value)
Unclassified (UNCLASSIFIED)	17977	4	7.86	-	0.51	0
**Cluster 4**						
Genes List:	AGER,BTC,EGF,EGFR,ERBB3,ERRFI1,HGFAC,LINGO1,MET,NKD2,NRG1,SPINT1,TGFA,AREG,GPNMB,HGF					
Biological Process						
Bonferroni count:	1810					
PANTHER GO-Slim Biological Process	Homo sapiens - REFLIST (20595)	BC_4.txt (16)	BC_4.txt (expected)	BC_4.txt (over/under)	BC_4.txt (fold Enrichment)	BC_4.txt (P-value)
positive regulation of mitotic nuclear division (GO:0045840)	12	3	0.01	+	> 100	0.000314
regulation of epidermal growth factor receptor signaling pathway (GO:0042058)	17	4	0.01	+	> 100	2.6E-06
epidermal growth factor receptor signaling pathway (GO:0007173)	21	4	0.02	+	> 100	5.49E-06
ERBB signaling pathway (GO:0038127)	23	4	0.02	+	> 100	7.61E-06
positive regulation of mitotic cell cycle (GO:0045931)	21	3	0.02	+	> 100	0.00139
positive regulation of peptidyl-tyrosine phosphorylation (GO:0050731)	25	3	0.02	+	> 100	0.00225
regulation of peptidyl-tyrosine phosphorylation (GO:0050730)	30	3	0.02	+	> 100	0.00374
regulation of signaling receptor activity (GO:0010469)	32	3	0.02	+	> 100	0.00448
positive regulation of cell cycle process (GO:0090068)	34	3	0.03	+	> 100	0.00531
regulation of mitotic nuclear division (GO:0007088)	35	3	0.03	+	> 100	0.00576
positive regulation of cell cycle (GO:0045787)	37	3	0.03	+	> 100	0.00674

regulation of nuclear division (GO:0051783)	40	3	0.03	+	96.54	0.00841
peptidyl-tyrosine phosphorylation (GO:0018108)	51	3	0.04	+	75.72	0.0168
peptidyl-tyrosine modification (GO:0018212)	53	3	0.04	+	72.86	0.0188
positive regulation of cell population proliferation (GO:0008284)	90	5	0.07	+	71.51	1.42E-05
positive regulation of protein kinase activity (GO:0045860)	72	4	0.06	+	71.51	0.000544
positive regulation of kinase activity (GO:0033674)	128	7	0.1	+	70.39	8.71E-09
positive regulation of transferase activity (GO:0051347)	136	7	0.11	+	66.25	1.31E-08
transmembrane receptor protein tyrosine kinase signaling pathway (GO:0007169)	212	8	0.16	+	48.57	3.21E-09
regulation of kinase activity (GO:0043549)	206	7	0.16	+	43.74	2.18E-07
regulation of transferase activity (GO:0051338)	215	7	0.17	+	41.91	2.92E-07
regulation of cell population proliferation (GO:0042127)	167	5	0.13	+	38.54	0.00028
regulation of protein kinase activity (GO:0045859)	144	4	0.11	+	35.76	0.00786
cell population proliferation (GO:0008283)	190	5	0.15	+	33.87	0.000523
enzyme linked receptor protein signaling pathway (GO:0007167)	317	8	0.25	+	32.48	7.32E-08
positive regulation of phosphorylation (GO:0042327)	293	7	0.23	+	30.75	2.39E-06
positive regulation of phosphorus metabolic process (GO:0010562)	300	7	0.23	+	30.03	2.8E-06
positive regulation of phosphate metabolic process (GO:0045937)	300	7	0.23	+	30.03	2.8E-06
positive regulation of catalytic activity (GO:0043085)	323	7	0.25	+	27.9	4.63E-06
regulation of phosphorylation (GO:0042325)	393	7	0.31	+	22.93	1.75E-05
regulation of phosphorus metabolic process	419	7	0.33	+	21.5	2.7E-05

(GO:0051174)						
regulation of phosphate metabolic process (GO:0019220)	419	7	0.33	+	21.5	2.7E-05
positive regulation of molecular function (GO:0044093)	421	7	0.33	+	21.4	2.79E-05
regulation of catalytic activity (GO:0050790)	646	7	0.5	+	13.95	0.000501
phosphorylation (GO:0016310)	704	7	0.55	+	12.8	0.000891
positive regulation of cellular metabolic process (GO:0031325)	768	7	0.6	+	11.73	0.00159
cell surface receptor signaling pathway (GO:0007166)	1003	9	0.78	+	11.55	2.43E-05
regulation of molecular function (GO:0065009)	806	7	0.63	+	11.18	0.00219
positive regulation of metabolic process (GO:0009893)	841	7	0.65	+	10.71	0.00291
phosphate-containing compound metabolic process (GO:0006796)	1090	7	0.85	+	8.27	0.0161
phosphorus metabolic process (GO:0006793)	1103	7	0.86	+	8.17	0.0174
positive regulation of cellular process (GO:0048522)	1295	7	1.01	+	6.96	0.0493
signal transduction (GO:0007165)	2062	9	1.6	+	5.62	0.011
signaling (GO:0023052)	2218	9	1.72	+	5.22	0.0201
cell communication (GO:0007154)	2230	9	1.73	+	5.19	0.021
response to stimulus (GO:0050896)	3027	10	2.35	+	4.25	0.0293
Unclassified (UNCLASSIFIED)	9793	5	7.61	-	0.66	0
Molecular Function						
Bonferroni count:	510					
PANTHER GO-Slim Molecular Function	Homo sapiens - REFLIST (20595)	BC_4.txt (16)	BC_4.txt (expected)	BC_4.txt (over/under)	BC_4.txt (fold Enrichment)	BC_4.txt (P-value)
transmembrane receptor protein tyrosine kinase activity (GO:0004714)	54	3	0.04	+	71.51	0.00558
protein tyrosine kinase activity (GO:0004713)	83	4	0.06	+	62.03	0.000264
transmembrane receptor protein kinase activity (GO:0019199)	64	3	0.05	+	60.34	0.0091
Unclassified	10792	9	8.38	+	1.07	0

(UNCLASSIFIED)						
Cellular Component						
Bonferroni count:	438					
PANTHER GO-Slim Cellular Component	Homo sapiens - REFLIST (20595)	BC_4.txt (16)	BC_4.txt (expected)	BC_4.txt (over/under)	BC_4.txt (fold Enrichment)	BC_4.txt (P-value)
Unclassified (UNCLASSIFIED)	9302	6	7.23	-	0.83	0
Pathway						
Bonferroni count:	156					
PANTHER Pathways	Homo sapiens - REFLIST (20595)	BC_4.txt (16)	BC_4.txt (expected)	BC_4.txt (over/under)	BC_4.txt (fold Enrichment)	BC_4.txt (P-value)
EGF receptor signaling pathway (P00018)	141	7	0.11	+	63.9	1.44E-09
Unclassified (UNCLASSIFIED)	17977	8	13.97	-	0.57	0
**Cluster 5**						
Genes List:	AHR,BAIAP2L1,DDB1,EPS8,SHANK1,SHANK2,SHANK3,WDTC1,DCAF4,FBXW5					
Biological Process						
Bonferroni count:	1810					
PANTHER GO-Slim Biological Process	Homo sapiens - REFLIST (20595)	BC_5.txt (10)	BC_5.txt (expected)	BC_5.txt (over/under)	BC_5.txt (fold Enrichment)	BC_5.txt (P-value)
dendritic spine organization (GO:0097061)	11	3	0.01	+	> 100	5.41E-05
regulation of dendritic spine development (GO:0060998)	13	3	0.01	+	> 100	8.32E-05
regulation of AMPA receptor activity (GO:2000311)	15	3	0.01	+	> 100	0.000121
neuromuscular junction development (GO:0007528)	15	3	0.01	+	> 100	0.000121
chemical synaptic transmission, postsynaptic (GO:0099565)	15	3	0.01	+	> 100	0.000121
ionotropic glutamate receptor signaling pathway (GO:0035235)	17	3	0.01	+	> 100	0.000169
regulation of neurotransmitter receptor activity (GO:0099601)	20	3	0.01	+	> 100	0.000263
regulation of synaptic transmission, glutamatergic (GO:0051966)	20	3	0.01	+	> 100	0.000263
dendrite morphogenesis (GO:0048813)	21	3	0.01	+	> 100	0.0003

synapse assembly (GO:0007416)	22	3	0.01	+	> 100	0.000341
regulation of dendrite development (GO:0050773)	22	3	0.01	+	> 100	0.000341
positive regulation of synaptic transmission (GO:0050806)	26	3	0.01	+	> 100	0.000541
regulation of synaptic plasticity (GO:0048167)	26	3	0.01	+	> 100	0.000541
positive regulation of cell projection organization (GO:0031346)	38	4	0.02	+	> 100	5.62E-06
glutamate receptor signaling pathway (GO:0007215)	29	3	0.01	+	> 100	0.000734
positive regulation of neuron projection development (GO:0010976)	30	3	0.01	+	> 100	0.000807
regulation of signaling receptor activity (GO:0010469)	32	3	0.02	+	> 100	0.000967
positive regulation of neuron differentiation (GO:0045666)	33	3	0.02	+	> 100	0.00105
dendrite development (GO:0016358)	35	3	0.02	+	> 100	0.00125
synaptic transmission, glutamatergic (GO:0035249)	36	3	0.02	+	> 100	0.00135
positive regulation of neurogenesis (GO:0050769)	39	3	0.02	+	> 100	0.00169
regulation of cation channel activity (GO:2001257)	40	3	0.02	+	> 100	0.00182
positive regulation of nervous system development (GO:0051962)	43	3	0.02	+	> 100	0.00224
developmental growth (GO:0048589)	44	3	0.02	+	> 100	0.00239
positive regulation of cell development (GO:0010720)	46	3	0.02	+	> 100	0.00271
head development (GO:0060322)	48	3	0.02	+	> 100	0.00306
brain development (GO:0007420)	48	3	0.02	+	> 100	0.00306
regulation of system process (GO:0044057)	48	3	0.02	+	> 100	0.00306
growth (GO:0040007)	54	3	0.03	+	> 100	0.0043
regulation of transmembrane transporter activity (GO:0022898)	57	3	0.03	+	> 100	0.00502
regulation of ion	57	3	0.03	+	> 100	0.00502

transmembrane transporter activity (GO:0032412)						
regulation of transporter activity (GO:0032409)	57	3	0.03	+	> 100	0.00502
regulation of cation transmembrane transport (GO:1904062)	62	3	0.03	+	99.65	0.00641
synapse organization (GO:0050808)	63	3	0.03	+	98.07	0.00671
regulation of plasma membrane bounded cell projection organization (GO:0120035)	89	4	0.04	+	92.56	0.000145
positive regulation of cell differentiation (GO:0045597)	67	3	0.03	+	92.22	0.00802
regulation of cell projection organization (GO:0031344)	91	4	0.04	+	90.53	0.000158
regulation of neuron projection development (GO:0010975)	74	3	0.04	+	83.49	0.0107
central nervous system development (GO:0007417)	79	3	0.04	+	78.21	0.0129
cell junction assembly (GO:0034329)	81	3	0.04	+	76.28	0.0139
regulation of ion transmembrane transport (GO:0034765)	82	3	0.04	+	75.35	0.0144
regulation of trans-synaptic signaling (GO:0099177)	83	3	0.04	+	74.44	0.0149
modulation of chemical synaptic transmission (GO:0050804)	83	3	0.04	+	74.44	0.0149
positive regulation of cellular component organization (GO:0051130)	139	5	0.07	+	74.08	6.89E-06
regulation of neuron differentiation (GO:0045664)	85	3	0.04	+	72.69	0.016
regulation of transmembrane transport (GO:0034762)	87	3	0.04	+	71.02	0.0171
positive regulation of developmental process (GO:0051094)	92	3	0.04	+	67.16	0.0201
regulation of neurogenesis (GO:0050767)	108	3	0.05	+	57.21	0.0321
regulation of ion transport (GO:0043269)	110	3	0.05	+	56.17	0.0339

regulation of nervous system development (GO:0051960)	118	3	0.06	+	52.36	0.0416
positive regulation of multicellular organismal process (GO:0051240)	118	3	0.06	+	52.36	0.0416
regulation of cell development (GO:0060284)	121	3	0.06	+	51.06	0.0448
animal organ morphogenesis (GO:0009887)	121	3	0.06	+	51.06	0.0448
regulation of cellular component organization (GO:0051128)	410	5	0.2	+	25.12	0.00136
plasma membrane bounded cell projection organization (GO:0120036)	395	4	0.19	+	20.86	0.048
Molecular Function						
Bonferroni count:	510					
PANTHER GO-Slim Molecular Function	Homo sapiens - REFLIST (20595)	BC_5.txt (10)	BC_5.txt (expected)	BC_5.txt (over/under)	BC_5.txt (fold Enrichment)	BC_5.txt (P-value)
signaling adaptor activity (GO:0035591)	11	3	0.01	+	> 100	1.52E-05
protein-macromolecule adaptor activity (GO:0030674)	84	3	0.04	+	73.55	0.00436
molecular adaptor activity (GO:0060090)	119	3	0.06	+	51.92	0.012
Unclassified (UNCLASSIFIED)	10792	4	5.24	-	0.76	0
Cellular Component						
Bonferroni count:	438					
PANTHER GO-Slim Cellular Component	Homo sapiens - REFLIST (20595)	BC_5.txt (10)	BC_5.txt (expected)	BC_5.txt (over/under)	BC_5.txt (fold Enrichment)	BC_5.txt (P-value)
dendritic spine (GO:0043197)	30	3	0.01	+	> 100	0.000195
neuron spine (GO:0044309)	30	3	0.01	+	> 100	0.000195
ionotropic glutamate receptor complex (GO:0008328)	35	3	0.02	+	> 100	0.000301
postsynaptic membrane (GO:0045211)	58	3	0.03	+	> 100	0.00128
neuron to neuron synapse (GO:0098984)	66	3	0.03	+	93.61	0.00186
asymmetric synapse (GO:0032279)	66	3	0.03	+	93.61	0.00186
postsynaptic density (GO:0014069)	66	3	0.03	+	93.61	0.00186
postsynaptic specialization (GO:0099572)	70	3	0.03	+	88.26	0.0022

synaptic membrane (GO:0097060)	78	3	0.04	+	79.21	0.00302
dendritic tree (GO:0097447)	108	3	0.05	+	57.21	0.00778
dendrite (GO:0030425)	108	3	0.05	+	57.21	0.00778
postsynapse (GO:0098794)	126	3	0.06	+	49.04	0.0122
ion channel complex (GO:0034702)	131	3	0.06	+	47.16	0.0137
somatodendritic compartment (GO:0036477)	134	3	0.07	+	46.11	0.0146
transmembrane transporter complex (GO:1902495)	136	3	0.07	+	45.43	0.0153
transporter complex (GO:1990351)	141	3	0.07	+	43.82	0.0169
receptor complex (GO:0043235)	213	4	0.1	+	38.68	0.00105
Pathway						
Bonferroni count:	156					
PANTHER Pathways	Homo sapiens - REFLIST (20595)	BC_5.txt (10)	BC_5.txt (expected)	BC_5.txt (over/under)	BC_5.txt (fold Enrichment)	BC_5.txt (P-value)
Ionotropic glutamate receptor pathway (P00037)	50	3	0.02	+	> 100	0.000297
Unclassified (UNCLASSIFIED)	17977	7	8.73	-	0.8	0
**Cluster 6**						
Genes List:	AMOT,FOS,FOSL1,JUN,MBD3,MECOM,PTPN14,RUNX2,SOX2,SOX9,TEAD1,TEAD3,TLE1,YAP1,ATF3,KLF4,POU5F1,FOSB,JUNB,AMOTL2,TEAD4					
Biological Process						
Bonferroni count:	1810					
PANTHER GO-Slim Biological Process	Homo sapiens - REFLIST (20595)	BC_6.txt (21)	BC_6.txt (expected)	BC_6.txt (over/under)	BC_6.txt (fold Enrichment)	BC_6.txt (P-value)
hippo signaling (GO:0035329)	17	6	0.02	+	> 100	9.21E-11
embryonic organ development (GO:0048568)	39	3	0.04	+	75.44	0.0184
vasculature development (GO:0001944)	55	3	0.06	+	53.49	0.0491
circulatory system development (GO:0072359)	110	4	0.11	+	35.66	0.00893
system development (GO:0048731)	804	8	0.82	+	9.76	0.00131
regulation of transcription by RNA polymerase II (GO:0006357)	1591	15	1.62	+	9.25	1.39E-09
transcription by	1635	15	1.67	+	9	2.06E-09

RNA polymerase II (GO:0006366)						
multicellular organism development (GO:0007275)	906	8	0.92	+	8.66	0.00318
regulation of transcription, DNA-templated (GO:0006355)	1984	16	2.02	+	7.91	1.34E-09
regulation of nucleic acid-templated transcription (GO:1903506)	1984	16	2.02	+	7.91	1.34E-09
regulation of RNA biosynthetic process (GO:2001141)	1984	16	2.02	+	7.91	1.34E-09
anatomical structure development (GO:0048856)	1126	9	1.15	+	7.84	0.00131
nucleic acid-templated transcription (GO:0097659)	2047	16	2.09	+	7.67	2.16E-09
transcription, DNA-templated (GO:0006351)	2047	16	2.09	+	7.67	2.16E-09
RNA biosynthetic process (GO:0032774)	2054	16	2.09	+	7.64	2.28E-09
regulation of cellular macromolecule biosynthetic process (GO:2000112)	2112	16	2.15	+	7.43	3.5E-09
regulation of RNA metabolic process (GO:0051252)	2113	16	2.15	+	7.43	3.53E-09
regulation of macromolecule biosynthetic process (GO:0010556)	2120	16	2.16	+	7.4	3.71E-09
regulation of cellular biosynthetic process (GO:0031326)	2134	16	2.18	+	7.35	4.11E-09
regulation of biosynthetic process (GO:0009889)	2141	16	2.18	+	7.33	4.32E-09
regulation of nucleobase-containing compound metabolic process (GO:0019219)	2161	16	2.2	+	7.26	4.98E-09
developmental process (GO:0032502)	1271	9	1.3	+	6.94	0.00359
nucleobase-containing compound biosynthetic process (GO:0034654)	2297	16	2.34	+	6.83	1.27E-08
heterocycle biosynthetic process (GO:0018130)	2322	16	2.37	+	6.76	1.5E-08
aromatic compound biosynthetic process (GO:0019438)	2323	16	2.37	+	6.75	1.51E-08

organic cyclic compound biosynthetic process (GO:1901362)	2350	16	2.4	+	6.68	1.81E-08
regulation of gene expression (GO:0010468)	2410	16	2.46	+	6.51	2.66E-08
cellular macromolecule biosynthetic process (GO:0034645)	2580	16	2.63	+	6.08	7.55E-08
macromolecule biosynthetic process (GO:0009059)	2590	16	2.64	+	6.06	8.01E-08
cellular nitrogen compound biosynthetic process (GO:0044271)	2597	16	2.65	+	6.04	8.35E-08
RNA metabolic process (GO:0016070)	2659	16	2.71	+	5.9	1.2E-07
regulation of nitrogen compound metabolic process (GO:0051171)	2715	16	2.77	+	5.78	1.65E-07
regulation of primary metabolic process (GO:0080090)	2743	16	2.8	+	5.72	1.92E-07
regulation of cellular metabolic process (GO:0031323)	2835	16	2.89	+	5.53	3.18E-07
nucleic acid metabolic process (GO:0090304)	2918	16	2.98	+	5.38	4.93E-07
regulation of macromolecule metabolic process (GO:0060255)	2931	16	2.99	+	5.35	5.27E-07
cellular biosynthetic process (GO:0044249)	2966	16	3.02	+	5.29	6.31E-07
organic substance biosynthetic process (GO:1901576)	3004	16	3.06	+	5.22	7.66E-07
biosynthetic process (GO:0009058)	3011	16	3.07	+	5.21	7.93E-07
gene expression (GO:0010467)	3048	16	3.11	+	5.15	9.55E-07
regulation of metabolic process (GO:0019222)	3093	16	3.15	+	5.07	1.19E-06
nucleobase-containing compound metabolic process (GO:0006139)	3150	16	3.21	+	4.98	1.57E-06
heterocycle metabolic process (GO:0046483)	3198	16	3.26	+	4.91	1.97E-06
cellular aromatic compound metabolic process (GO:0006725)	3214	16	3.28	+	4.88	2.13E-06
organic cyclic compound metabolic process (GO:1901360)	3262	16	3.33	+	4.81	2.66E-06

cellular nitrogen compound metabolic process (GO:0034641)	3483	16	3.55	+	4.51	7.14E-06
cellular macromolecule metabolic process (GO:0044260)	4233	16	4.32	+	3.71	0.000131
regulation of cellular process (GO:0050794)	5092	19	5.19	+	3.66	6.73E-07
regulation of biological process (GO:0050789)	5324	19	5.43	+	3.5	1.52E-06
biological regulation (GO:0065007)	5797	19	5.91	+	3.21	7.22E-06
macromolecule metabolic process (GO:0043170)	5051	16	5.15	+	3.11	0.00173
nitrogen compound metabolic process (GO:0006807)	5350	16	5.46	+	2.93	0.00397
primary metabolic process (GO:0044238)	5603	16	5.71	+	2.8	0.0077
cellular metabolic process (GO:0044237)	5684	16	5.8	+	2.76	0.00945
organic substance metabolic process (GO:0071704)	5910	16	6.03	+	2.66	0.0164
metabolic process (GO:0008152)	6178	16	6.3	+	2.54	0.0307
Molecular Function						
Bonferroni count:	510					
PANTHER GO-Slim Molecular Function	Homo sapiens - REFLIST (20595)	BC_6.txt (21)	BC_6.txt (expected)	BC_6.txt (over/under)	BC_6.txt (fold Enrichment)	BC_6.txt (P-value)
RNA polymerase II cis-regulatory region sequence-specific DNA binding (GO:0000978)	1054	15	1.07	+	13.96	9.89E-13
cis-regulatory region sequence-specific DNA binding (GO:0000987)	1057	15	1.08	+	13.92	1.03E-12
DNA-binding transcription factor activity, RNA polymerase II-specific (GO:0000981)	1226	15	1.25	+	12	8.94E-12
DNA-binding transcription factor activity (GO:0003700)	1313	15	1.34	+	11.2	2.42E-11
RNA polymerase II transcription regulatory region sequence-specific DNA binding (GO:0000977)	1337	15	1.36	+	11	3.15E-11
transcription regulator activity (GO:0140110)	1559	17	1.59	+	10.69	2.18E-13

transcription regulatory region sequence-specific DNA binding (GO:0000976)	1377	15	1.4	+	10.68	4.84E-11
regulatory region nucleic acid binding (GO:0001067)	1377	15	1.4	+	10.68	4.84E-11
sequence-specific double-stranded DNA binding (GO:1990837)	1403	15	1.43	+	10.49	6.34E-11
sequence-specific DNA binding (GO:0043565)	1436	15	1.46	+	10.24	8.89E-11
double-stranded DNA binding (GO:0003690)	1451	15	1.48	+	10.14	1.03E-10
DNA binding (GO:0003677)	1648	15	1.68	+	8.93	6.52E-10
molecular function regulator (GO:0098772)	2358	17	2.4	+	7.07	2.04E-10
nucleic acid binding (GO:0003676)	2248	15	2.29	+	6.54	5.63E-08
heterocyclic compound binding (GO:1901363)	2595	15	2.65	+	5.67	4.33E-07
organic cyclic compound binding (GO:0097159)	2632	15	2.68	+	5.59	5.29E-07
binding (GO:0005488)	5963	16	6.08	+	2.63	0.00525
molecular_function (GO:0003674)	9803	19	10	+	1.9	0.0371
Cellular Component						
Bonferroni count:	438					
PANTHER GO-Slim Cellular Component	Homo sapiens - REFLIST (20595)	BC_6.txt (21)	BC_6.txt (expected)	BC_6.txt (over/under)	BC_6.txt (fold Enrichment)	BC_6.txt (P-value)
transcription regulator complex (GO:0005667)	200	6	0.2	+	29.42	1.94E-05
nuclear chromatin (GO:0000790)	704	9	0.72	+	12.54	5.97E-06
chromatin (GO:0000785)	738	9	0.75	+	11.96	8.93E-06
nuclear chromosome (GO:0000228)	787	9	0.8	+	11.22	1.55E-05
chromosome (GO:0005694)	898	9	0.92	+	9.83	4.74E-05
nuclear lumen (GO:0031981)	1398	9	1.43	+	6.31	0.0019
membrane-enclosed lumen (GO:0031974)	1494	9	1.52	+	5.91	0.00327
intracellular organelle lumen (GO:0070013)	1494	9	1.52	+	5.91	0.00327
organelle lumen (GO:0043233)	1494	9	1.52	+	5.91	0.00327
intracellular non-membrane-bounded organelle (GO:0043232)	1959	9	2	+	4.51	0.0287

non-membrane-bounded organelle (GO:0043228)	1959	9	2	+	4.51	0.0287
nucleus (GO:0005634)	3956	17	4.03	+	4.21	7.99E-07
membrane-bounded organelle (GO:0043227)	5999	19	6.12	+	3.11	3.26E-06
intracellular membrane-bounded organelle (GO:0043231)	5903	17	6.02	+	2.82	0.000449
intracellular organelle (GO:0043229)	6633	19	6.76	+	2.81	2.02E-05
organelle (GO:0043226)	6781	19	6.91	+	2.75	3.01E-05
intracellular (GO:0005622)	8253	19	8.42	+	2.26	0.00102
Pathway						
Bonferroni count:	156					
PANTHER Pathways	Homo sapiens - REFLIST (20595)	BC_6.txt (21)	BC_6.txt (expected)	BC_6.txt (over/under)	BC_6.txt (fold Enrichment)	BC_6.txt (P-value)
Apoptosis signaling pathway (P00006)	118	4	0.12	+	33.24	0.00101
TGF-beta signaling pathway (P00052)	102	3	0.1	+	28.84	0.0249
Gonadotropin-releasing hormone receptor pathway (P06664)	231	5	0.24	+	21.23	0.000515
Unclassified (UNCLASSIFIED)	17977	13	18.33	-	0.71	0
**Cluster 7**						
Genes List:	AP4B1,BUB1B,CDC20,CDC27,PTTG1,BUB1					
Biological Process						
Bonferroni count:	1810					
PANTHER GO-Slim Biological Process	Homo sapiens - REFLIST (20595)	BC_7.txt (6)	BC_7.txt (expected)	BC_7.txt (over/under)	BC_7.txt (fold Enrichment)	BC_7.txt (P-value)
negative regulation of mitotic sister chromatid segregation (GO:0033048)	14	3	0	+	> 100	1.69E-05
negative regulation of sister chromatid segregation (GO:0033046)	14	3	0	+	> 100	1.69E-05
negative regulation of mitotic sister chromatid separation (GO:2000816)	14	3	0	+	> 100	1.69E-05
negative regulation of mitotic nuclear division (GO:0045839)	14	3	0	+	> 100	1.69E-05
negative regulation of chromosome separation	15	3	0	+	> 100	2.02E-05

(GO:1905819)						
negative regulation of chromosome segregation (GO:0051985)	15	3	0	+	> 100	2.02E-05
negative regulation of nuclear division (GO:0051784)	18	3	0.01	+	> 100	3.3E-05
regulation of mitotic sister chromatid separation (GO:0010965)	19	3	0.01	+	> 100	3.82E-05
mitotic sister chromatid separation (GO:0051306)	20	3	0.01	+	> 100	4.39E-05
regulation of chromosome separation (GO:1905818)	21	3	0.01	+	> 100	5.02E-05
regulation of mitotic sister chromatid segregation (GO:0033047)	21	3	0.01	+	> 100	5.02E-05
regulation of mitotic metaphase/anaphase transition (GO:0030071)	15	2	0	+	> 100	0.0174
regulation of sister chromatid segregation (GO:0033045)	24	3	0.01	+	> 100	7.25E-05
regulation of chromosome segregation (GO:0051983)	26	3	0.01	+	> 100	9.05E-05
regulation of mitotic nuclear division (GO:0007088)	35	3	0.01	+	> 100	0.000209
negative regulation of chromosome organization (GO:2001251)	37	3	0.01	+	> 100	0.000244
negative regulation of cell cycle process (GO:0010948)	39	3	0.01	+	> 100	0.000284
regulation of nuclear division (GO:0051783)	40	3	0.01	+	> 100	0.000305
chromosome separation (GO:0051304)	41	3	0.01	+	> 100	0.000328
meiotic chromosome segregation (GO:0045132)	43	3	0.01	+	> 100	0.000375
negative regulation of mitotic cell cycle (GO:0045930)	58	3	0.02	+	> 100	0.000889
mitotic sister chromatid segregation (GO:0000070)	61	3	0.02	+	> 100	0.00103
regulation of chromosome organization (GO:0033044)	68	3	0.02	+	> 100	0.00141
sister chromatid segregation	79	3	0.02	+	> 100	0.00218

(GO:0000819)						
negative regulation of organelle organization (GO:0010639)	85	3	0.02	+	> 100	0.0027
meiotic cell cycle process (GO:1903046)	91	3	0.03	+	> 100	0.0033
meiotic nuclear division (GO:0140013)	91	3	0.03	+	> 100	0.0033
meiotic cell cycle (GO:0051321)	91	3	0.03	+	> 100	0.0033
negative regulation of cell cycle (GO:0045786)	93	3	0.03	+	> 100	0.00351
regulation of mitotic cell cycle (GO:0007346)	103	3	0.03	+	99.98	0.00474
nuclear chromosome segregation (GO:0098813)	109	3	0.03	+	94.47	0.0056
regulation of cell cycle process (GO:0010564)	110	3	0.03	+	93.61	0.00575
negative regulation of cellular component organization (GO:0051129)	114	3	0.03	+	90.33	0.00638
chromosome segregation (GO:0007059)	125	3	0.04	+	82.38	0.00837
reproductive process (GO:0022414)	179	3	0.05	+	57.53	0.0241
reproduction (GO:0000003)	179	3	0.05	+	57.53	0.0241
regulation of cell cycle (GO:0051726)	207	3	0.06	+	49.75	0.037
Molecular Function						
Bonferroni count:	510					
PANTHER GO-Slim Molecular Function	Homo sapiens - REFLIST (20595)	BC_7.txt (6)	BC_7.txt (expected)	BC_7.txt (over/under)	BC_7.txt (fold Enrichment)	BC_7.txt (P-value)
Unclassified (UNCLASSIFIED)	10792	3	3.14	-	0.95	0
Cellular Component						
Bonferroni count:	438					
PANTHER GO-Slim Cellular Component	Homo sapiens - REFLIST (20595)	BC_7.txt (6)	BC_7.txt (expected)	BC_7.txt (over/under)	BC_7.txt (fold Enrichment)	BC_7.txt (P-value)
Pathway						
Bonferroni count:	156					
PANTHER Pathways	Homo sapiens - REFLIST (20595)	BC_7.txt (6)	BC_7.txt (expected)	BC_7.txt (over/under)	BC_7.txt (fold Enrichment)	BC_7.txt (P-value)
Unclassified (UNCLASSIFIED)	17977	6	5.24	+	1.15	0
**Cluster 8**						
Genes List:	AR,BAG1,CDK7,POLR2A,PRKCB,PR					

	KCD,RIPK4,RPRD1B,TGFB1I1,FOXA1,TMF1,CCNK,SMN1,PIK3CG					
Biological Process						
Bonferroni count:	1810					
PANTHER GO-Slim Biological Process	Homo sapiens - REFLIST (20595)	BC_8.txt (15)	BC_8.txt (expected)	BC_8.txt (over/under)	BC_8.txt (fold Enrichment)	BC_8.txt (P-value)
Unclassified (UNCLASSIFIED)	9793	5	7.13	-	0.7	0
Molecular Function						
Bonferroni count:	510					
PANTHER GO-Slim Molecular Function	Homo sapiens - REFLIST (20595)	BC_8.txt (15)	BC_8.txt (expected)	BC_8.txt (over/under)	BC_8.txt (fold Enrichment)	BC_8.txt (P-value)
phosphotransferase activity, alcohol group as acceptor (GO:0016773)	555	5	0.4	+	12.37	0.0178
transferase activity, transferring phosphorus-containing groups (GO:0016772)	714	6	0.52	+	11.54	0.00347
kinase activity (GO:0016301)	613	5	0.45	+	11.2	0.0285
Unclassified (UNCLASSIFIED)	10792	3	7.86	-	0.38	0
Cellular Component						
Bonferroni count:	438					
PANTHER GO-Slim Cellular Component	Homo sapiens - REFLIST (20595)	BC_8.txt (15)	BC_8.txt (expected)	BC_8.txt (over/under)	BC_8.txt (fold Enrichment)	BC_8.txt (P-value)
RNA polymerase II, holoenzyme (GO:0016591)	63	3	0.05	+	65.38	0.00608
nuclear DNA-directed RNA polymerase complex (GO:0055029)	82	3	0.06	+	50.23	0.013
DNA-directed RNA polymerase complex (GO:0000428)	83	3	0.06	+	49.63	0.0135
RNA polymerase complex (GO:0030880)	84	3	0.06	+	49.04	0.014
transferase complex, transferring phosphorus-containing groups (GO:0061695)	188	5	0.14	+	36.52	8.33E-05
transferase complex (GO:1990234)	482	5	0.35	+	14.24	0.0078
nucleoplasm (GO:0005654)	504	5	0.37	+	13.62	0.00965
nuclear lumen (GO:0031981)	1398	7	1.02	+	6.87	0.0117
membrane-enclosed lumen (GO:0031974)	1494	7	1.09	+	6.43	0.0179
intracellular organelle lumen (GO:0070013)	1494	7	1.09	+	6.43	0.0179

organelle lumen (GO:0043233)	1494	7	1.09	+	6.43	0.0179
Unclassified (UNCLASSIFIED)	9302	3	6.77	-	0.44	0
Pathway						
Bonferroni count:	156					
PANTHER Pathways	Homo sapiens - REFLIST (20595)	BC_8.txt (15)	BC_8.txt (expected)	BC_8.txt (over/under)	BC_8.txt (fold Enrichment)	BC_8.txt (P-value)
VEGF signaling pathway (P00056)	68	4	0.05	+	80.76	2.83E-05
B cell activation (P00010)	70	3	0.05	+	58.84	0.00293
Endothelin signaling pathway (P00019)	85	3	0.06	+	48.46	0.00514
Apoptosis signaling pathway (P00006)	118	4	0.09	+	46.54	0.000237
FGF signaling pathway (P00021)	123	3	0.09	+	33.49	0.015
Angiogenesis (P00005)	175	4	0.13	+	31.38	0.00109
EGF receptor signaling pathway (P00018)	141	3	0.1	+	29.21	0.0223
Unclassified (UNCLASSIFIED)	17977	8	13.09	-	0.61	0
**Cluster 9**						
Genes List:	ARRB2,CNR1,GRM2,HTR2A,INSR,IRS1,MAP1A,NDUFB10,NTF3,NTRK3,PTPN1,PTPN3,SORT1,ADRB1,EDNRA,ANKS1B,GLUL,PON2,GRB14,BDNF					
Biological Process						
Bonferroni count:	1810					
PANTHER GO-Slim Biological Process	Homo sapiens - REFLIST (20595)	BC_9.txt (20)	BC_9.txt (expected)	BC_9.txt (over/under)	BC_9.txt (fold Enrichment)	BC_9.txt (P-value)
transmembrane receptor protein tyrosine kinase signaling pathway (GO:0007169)	212	6	0.21	+	29.14	8.09E-05
cellular response to endogenous stimulus (GO:0071495)	244	5	0.24	+	21.1	0.00598
response to endogenous stimulus (GO:0009719)	259	5	0.25	+	19.88	0.00795
enzyme linked receptor protein signaling pathway (GO:0007167)	317	6	0.31	+	19.49	0.000823
positive regulation of phosphorylation (GO:0042327)	293	5	0.28	+	17.57	0.0143
positive regulation of phosphorus metabolic process	300	5	0.29	+	17.16	0.016

(GO:0010562)						
positive regulation of phosphate metabolic process (GO:0045937)	300	5	0.29	+	17.16	0.016
regulation of cell communication (GO:0010646)	813	7	0.79	+	8.87	0.0137
regulation of signaling (GO:0023051)	813	7	0.79	+	8.87	0.0137
signal transduction (GO:0007165)	2062	11	2	+	5.49	0.00133
signaling (GO:0023052)	2218	11	2.15	+	5.11	0.00277
cell communication (GO:0007154)	2230	11	2.17	+	5.08	0.00292
cellular response to stimulus (GO:0051716)	2522	11	2.45	+	4.49	0.00989
Unclassified (UNCLASSIFIED)	9793	6	9.51	-	0.63	0
Molecular Function						
Bonferroni count:	510					
PANTHER GO-Slim Molecular Function	Homo sapiens - REFLIST (20595)	BC_9.txt (20)	BC_9.txt (expected)	BC_9.txt (over/under)	BC_9.txt (fold Enrichment)	BC_9.txt (P-value)
transmembrane signaling receptor activity (GO:0004888)	669	6	0.65	+	9.24	0.0161
molecular transducer activity (GO:0060089)	783	6	0.76	+	7.89	0.0385
signaling receptor activity (GO:0038023)	783	6	0.76	+	7.89	0.0385
Unclassified (UNCLASSIFIED)	10792	7	10.48	-	0.67	0
Cellular Component						
Bonferroni count:	438					
PANTHER GO-Slim Cellular Component	Homo sapiens - REFLIST (20595)	BC_9.txt (20)	BC_9.txt (expected)	BC_9.txt (over/under)	BC_9.txt (fold Enrichment)	BC_9.txt (P-value)
Unclassified (UNCLASSIFIED)	9302	5	9.03	-	0.55	0
Pathway						
Bonferroni count:	156					
PANTHER Pathways	Homo sapiens - REFLIST (20595)	BC_9.txt (20)	BC_9.txt (expected)	BC_9.txt (over/under)	BC_9.txt (fold Enrichment)	BC_9.txt (P-value)
Heterotrimeric G-protein signaling pathway-Gi alpha and Gs alpha mediated pathway (P00026)	166	4	0.16	+	24.81	0.00304
Unclassified (UNCLASSIFIED)	17977	8	17.46	-	0.46	0
**Cluster 10**						
Genes List:	ATAD2,CSF2RB,ESR1,PBXIP1,PGR,					

	PIK3CA,PIK3R1,THRB,ZBTB17,NR2F1,GFI1					
Biological Process						
Bonferroni count:	1810					
PANTHER GO-Slim Biological Process	Homo sapiens - REFLIST (20595)	BC_10.txt (12)	BC_10.txt (expected)	BC_10.txt (over/under)	BC_10.txt (fold Enrichment)	BC_10.txt (P-value)
regulation of transcription by RNA polymerase II (GO:0006357)	1591	7	0.93	+	7.55	0.0168
transcription by RNA polymerase II (GO:0006366)	1635	7	0.95	+	7.35	0.0202
Molecular Function						
Bonferroni count:	510					
PANTHER GO-Slim Molecular Function	Homo sapiens - REFLIST (20595)	BC_10.txt (12)	BC_10.txt (expected)	BC_10.txt (over/under)	BC_10.txt (fold Enrichment)	BC_10.txt (P-value)
RNA polymerase II cis-regulatory region sequence-specific DNA binding (GO:0000978)	1054	6	0.61	+	9.77	0.00659
cis-regulatory region sequence-specific DNA binding (GO:0000987)	1057	6	0.62	+	9.74	0.00669
DNA-binding transcription factor activity (GO:0003700)	1313	6	0.77	+	7.84	0.0229
RNA polymerase II transcription regulatory region sequence-specific DNA binding (GO:0000977)	1337	6	0.78	+	7.7	0.0254
transcription regulatory region sequence-specific DNA binding (GO:0000976)	1377	6	0.8	+	7.48	0.03
regulatory region nucleic acid binding (GO:0001067)	1377	6	0.8	+	7.48	0.03
sequence-specific double-stranded DNA binding (GO:1990837)	1403	6	0.82	+	7.34	0.0333
sequence-specific DNA binding (GO:0043565)	1436	6	0.84	+	7.17	0.0379
double-stranded DNA binding (GO:0003690)	1451	6	0.85	+	7.1	0.0402
Cellular Component						
Bonferroni count:	438					
PANTHER GO-Slim Cellular Component	Homo sapiens - REFLIST (20595)	BC_10.txt (12)	BC_10.txt (expected)	BC_10.txt (over/under)	BC_10.txt (fold Enrichment)	BC_10.txt (P-value)
Unclassified	9302	3	5.42	-	0.55	0

(UNCLASSIFIED)						
Pathway						
Bonferroni count:	156					
PANTHER Pathways	Homo sapiens - REFLIST (20595)	BC_10.txt (12)	BC_10.txt (expected)	BC_10.txt (over/under)	BC_10.txt (fold Enrichment)	BC_10.txt (P-value)
Unclassified (UNCLASSIFIED)	17977	7	10.47	-	0.67	0
**Cluster 11**						
Genes List:	BTK,CDCP1,GP6,HCK,IL6,IL6R,IL6ST,LIFR,LYN,OSM,PLAUR,SYK,TYK2,YES1,FAS,LIF,FGR,PLAU,RPS6KA2					
Biological Process						
Bonferroni count:	1810					
PANTHER GO-Slim Biological Process	Homo sapiens - REFLIST (20595)	BC_11.txt (21)	BC_11.txt (expected)	BC_11.txt (over/under)	BC_11.txt (fold Enrichment)	BC_11.txt (P-value)
protein autophosphorylation (GO:0046777)	40	4	0.04	+	98.07	0.00019
peptidyl-tyrosine phosphorylation (GO:0018108)	51	5	0.05	+	96.15	4.39E-06
peptidyl-tyrosine modification (GO:0018212)	53	5	0.05	+	92.52	5.26E-06
regulation of cell population proliferation (GO:0042127)	167	6	0.17	+	35.24	2.83E-05
cell population proliferation (GO:0008283)	190	6	0.19	+	30.97	5.97E-05
protein phosphorylation (GO:0006468)	541	7	0.55	+	12.69	0.00137
phosphorylation (GO:0016310)	704	7	0.72	+	9.75	0.00777
cell surface receptor signaling pathway (GO:0007166)	1003	9	1.02	+	8.8	0.000498
signal transduction (GO:0007165)	2062	10	2.1	+	4.76	0.0232
signaling (GO:0023052)	2218	10	2.26	+	4.42	0.0441
cell communication (GO:0007154)	2230	10	2.27	+	4.4	0.0463
Unclassified (UNCLASSIFIED)	9793	9	9.99	-	0.9	0
Molecular Function						
Bonferroni count:	510					
PANTHER GO-Slim Molecular Function	Homo sapiens - REFLIST (20595)	BC_11.txt (21)	BC_11.txt (expected)	BC_11.txt (over/under)	BC_11.txt (fold Enrichment)	BC_11.txt (P-value)
cytokine binding (GO:0019955)	65	4	0.07	+	60.35	0.000336
cytokine receptor activity	50	3	0.05	+	58.84	0.0105

(GO:0004896)						
protein tyrosine kinase activity (GO:0004713)	83	4	0.08	+	47.26	0.000855
protein kinase activity (GO:0004672)	467	5	0.48	+	10.5	0.0472
Unclassified (UNCLASSIFIED)	10792	10	11	-	0.91	0
Cellular Component						
Bonferroni count:	438					
PANTHER GO-Slim Cellular Component	Homo sapiens - REFLIST (20595)	BC_11.txt (21)	BC_11.txt (expected)	BC_11.txt (over/under)	BC_11.txt (fold Enrichment)	BC_11.txt (P-value)
extrinsic component of cytoplasmic side of plasma membrane (GO:0031234)	56	4	0.06	+	70.05	0.000164
cytoplasmic side of plasma membrane (GO:0009898)	69	4	0.07	+	56.85	0.000362
cytoplasmic side of membrane (GO:0098562)	75	4	0.08	+	52.3	0.000498
extrinsic component of plasma membrane (GO:0019897)	87	4	0.09	+	45.09	0.000879
extrinsic component of membrane (GO:0019898)	134	4	0.14	+	29.28	0.00461
side of membrane (GO:0098552)	292	8	0.3	+	26.87	1.39E-07
leaflet of membrane bilayer (GO:0097478)	292	8	0.3	+	26.87	1.39E-07
external side of plasma membrane (GO:0009897)	217	4	0.22	+	18.08	0.0292
Unclassified (UNCLASSIFIED)	9302	9	9.48	-	0.95	0
Pathway						
Bonferroni count:	156					
PANTHER Pathways	Homo sapiens - REFLIST (20595)	BC_11.txt (21)	BC_11.txt (expected)	BC_11.txt (over/under)	BC_11.txt (fold Enrichment)	BC_11.txt (P-value)
Interleukin signaling pathway (P00036)	86	4	0.09	+	45.61	0.0003
B cell activation (P00010)	70	3	0.07	+	42.03	0.00844
Parkinson disease (P00049)	97	4	0.1	+	40.44	0.000475
Unclassified (UNCLASSIFIED)	17977	7	18.33	-	0.38	0
**Cluster 12**						
Genes List:	CCNA2,CCNB1,CCND1,CCNE1,CCP110,CDK1,CDK6,CDKN1A,CDKN1B,CDKN3,DDB2,GADD45G,MXD1,RPS6KA1,SCML2,AK					

	T3,FBXO31,CCNF,GADD45A,CDKN2D					
Biological Process						
Bonferroni count:	1810					
PANTHER GO-Slim Biological Process	Homo sapiens - REFLIST (20595)	BC_12.txt (21)	BC_12.txt (expected)	BC_12.txt (over/under)	BC_12.txt (fold Enrichment)	BC_12.txt (P-value)
regulation of cyclin-dependent protein kinase activity (GO:1904029)	28	5	0.03	+	> 100	2.77E-07
regulation of cyclin-dependent protein serine/threonine kinase activity (GO:0000079)	28	5	0.03	+	> 100	2.77E-07
G1/S transition of mitotic cell cycle (GO:0000082)	31	4	0.03	+	> 100	7.39E-05
cell cycle G1/S phase transition (GO:0044843)	33	4	0.03	+	> 100	9.3E-05
mitotic cell cycle phase transition (GO:0044772)	84	8	0.09	+	93.4	4.05E-11
cell cycle phase transition (GO:0044770)	90	8	0.09	+	87.17	6.82E-11
regulation of protein serine/threonine kinase activity (GO:0071900)	89	7	0.09	+	77.13	7.58E-09
regulation of mitotic cell cycle phase transition (GO:1901990)	53	3	0.05	+	55.51	0.0441
regulation of cell cycle (GO:0051726)	207	11	0.21	+	52.12	8.35E-14
regulation of protein kinase activity (GO:0045859)	144	7	0.15	+	47.67	1.9E-07
regulation of kinase activity (GO:0043549)	206	7	0.21	+	33.33	2.12E-06
regulation of transferase activity (GO:0051338)	215	7	0.22	+	31.93	2.83E-06
mitotic cell cycle (GO:0000278)	267	8	0.27	+	29.38	2.87E-07
mitotic cell cycle process (GO:1903047)	267	8	0.27	+	29.38	2.87E-07
mitotic nuclear division (GO:0140014)	267	8	0.27	+	29.38	2.87E-07
nuclear division (GO:0000280)	319	8	0.33	+	24.59	1.13E-06
organelle fission (GO:0048285)	340	8	0.35	+	23.08	1.85E-06
cell cycle (GO:0007049)	473	11	0.48	+	22.81	5.51E-10
protein phosphorylation (GO:0006468)	541	12	0.55	+	21.75	5.27E-11
regulation of protein phosphorylation	327	7	0.33	+	20.99	4.76E-05

(GO:0001932)						
regulation of protein modification process (GO:0031399)	378	7	0.39	+	18.16	0.000126
cell cycle process (GO:0022402)	434	8	0.44	+	18.08	1.21E-05
regulation of phosphorylation (GO:0042325)	393	7	0.4	+	17.47	0.000164
phosphorylation (GO:0016310)	704	12	0.72	+	16.72	1.12E-09
regulation of phosphorus metabolic process (GO:0051174)	419	7	0.43	+	16.38	0.000251
regulation of phosphate metabolic process (GO:0019220)	419	7	0.43	+	16.38	0.000251
regulation of cellular protein metabolic process (GO:0032268)	571	7	0.58	+	12.02	0.00196
regulation of protein metabolic process (GO:0051246)	608	7	0.62	+	11.29	0.00297
phosphate-containing compound metabolic process (GO:0006796)	1090	12	1.11	+	10.8	1.74E-07
phosphorus metabolic process (GO:0006793)	1103	12	1.12	+	10.67	2E-07
regulation of catalytic activity (GO:0050790)	646	7	0.66	+	10.63	0.00443
regulation of molecular function (GO:0065009)	806	7	0.82	+	8.52	0.0187
cellular protein modification process (GO:0006464)	1383	12	1.41	+	8.51	2.64E-06
protein modification process (GO:0036211)	1383	12	1.41	+	8.51	2.64E-06
macromolecule modification (GO:0043412)	1481	12	1.51	+	7.95	5.73E-06
cellular protein metabolic process (GO:0044267)	1906	13	1.94	+	6.69	6.82E-06
protein metabolic process (GO:0019538)	2236	13	2.28	+	5.7	4.74E-05
organonitrogen compound metabolic process (GO:1901564)	2699	13	2.75	+	4.72	0.000451
cellular macromolecule metabolic process (GO:0044260)	4233	16	4.32	+	3.71	0.000131
macromolecule metabolic process (GO:0043170)	5051	16	5.15	+	3.11	0.00173
nitrogen compound	5350	16	5.46	+	2.93	0.00397

metabolic process (GO:0006807)						
regulation of cellular process (GO:0050794)	5092	15	5.19	+	2.89	0.0162
primary metabolic process (GO:0044238)	5603	16	5.71	+	2.8	0.0077
regulation of biological process (GO:0050789)	5324	15	5.43	+	2.76	0.0291
cellular metabolic process (GO:0044237)	5684	16	5.8	+	2.76	0.00945
organic substance metabolic process (GO:0071704)	5910	16	6.03	+	2.66	0.0164
metabolic process (GO:0008152)	6178	16	6.3	+	2.54	0.0307
Unclassified (UNCLASSIFIED)	9793	4	9.99	-	0.4	0
Molecular Function						
Bonferroni count:	510					
PANTHER GO-Slim Molecular Function	Homo sapiens - REFLIST (20595)	BC_12.txt (21)	BC_12.txt (expected)	BC_12.txt (over/under)	BC_12.txt (fold Enrichment)	BC_12.txt (P-value)
cyclin-dependent protein serine/threonine kinase regulator activity (GO:0016538)	36	6	0.04	+	> 100	1.33E-09
cyclin-dependent protein kinase activity (GO:0097472)	63	9	0.06	+	> 100	6.67E-15
cyclin-dependent protein serine/threonine kinase activity (GO:0004693)	63	9	0.06	+	> 100	6.67E-15
cyclin binding (GO:0030332)	65	9	0.07	+	> 100	8.65E-15
protein kinase regulator activity (GO:0019887)	71	6	0.07	+	82.88	5.89E-08
kinase regulator activity (GO:0019207)	89	6	0.09	+	66.12	2.14E-07
protein kinase binding (GO:0019901)	176	6	0.18	+	33.43	1.08E-05
protein serine/threonine kinase activity (GO:0004674)	349	11	0.36	+	30.91	6.09E-12
kinase binding (GO:0019900)	196	6	0.2	+	30.02	2.01E-05
protein kinase activity (GO:0004672)	467	11	0.48	+	23.1	1.36E-10
phosphotransferase activity, alcohol group as acceptor (GO:0016773)	555	11	0.57	+	19.44	8.51E-10
kinase activity (GO:0016301)	613	11	0.63	+	17.6	2.45E-09

transferase activity, transferring phosphorus-containing groups (GO:0016772)	714	11	0.73	+	15.11	1.23E-08
enzyme regulator activity (GO:0030234)	402	6	0.41	+	14.64	0.00125
enzyme binding (GO:0019899)	703	6	0.72	+	8.37	0.0288
catalytic activity, acting on a protein (GO:0140096)	1551	11	1.58	+	6.96	4.03E-05
transferase activity (GO:0016740)	1572	11	1.6	+	6.86	4.62E-05
Unclassified (UNCLASSIFIED)	10792	7	11	-	0.64	0
Cellular Component						
Bonferroni count:	438					
PANTHER GO-Slim Cellular Component	Homo sapiens - REFLIST (20595)	BC_12.txt (21)	BC_12.txt (expected)	BC_12.txt (over/under)	BC_12.txt (fold Enrichment)	BC_12.txt (P-value)
cyclin-dependent protein kinase holoenzyme complex (GO:0000307)	29	6	0.03	+	> 100	3.56E-10
serine/threonine protein kinase complex (GO:1902554)	52	6	0.05	+	> 100	8.74E-09
protein kinase complex (GO:1902911)	59	6	0.06	+	99.73	1.78E-08
centrosome (GO:0005813)	134	5	0.14	+	36.59	0.000106
centriole (GO:0005814)	149	5	0.15	+	32.91	0.000176
transferase complex, transferring phosphorus-containing groups (GO:0061695)	188	6	0.19	+	31.3	1.36E-05
microtubule organizing center (GO:0005815)	194	5	0.2	+	25.28	0.000626
transferase complex (GO:1990234)	482	7	0.49	+	14.24	0.000155
microtubule cytoskeleton (GO:0015630)	432	5	0.44	+	11.35	0.0282
catalytic complex (GO:1902494)	858	7	0.87	+	8	0.00679
nucleus (GO:0005634)	3956	15	4.03	+	3.72	0.000132
intracellular membrane-bounded organelle (GO:0043231)	5903	15	6.02	+	2.49	0.0268
membrane-bounded organelle (GO:0043227)	5999	15	6.12	+	2.45	0.033
Unclassified (UNCLASSIFIED)	9302	5	9.48	-	0.53	0
Pathway						

Bonferroni count:	156					
PANTHER Pathways	Homo sapiens - REFLIST (20595)	BC_12.txt (21)	BC_12.txt (expected)	BC_12.txt (over/under)	BC_12.txt (fold Enrichment)	BC_12.txt (P-value)
Cell cycle (P00013)	22	3	0.02	+	> 100	0.000322
p53 pathway (P00059)	89	9	0.09	+	99.17	3.72E-14
p53 pathway feedback loops 2 (P04398)	51	4	0.05	+	76.92	4.09E-05
PI3 kinase pathway (P00048)	57	3	0.06	+	51.62	0.00468
Interleukin signaling pathway (P00036)	86	4	0.09	+	45.61	0.0003
Unclassified (UNCLASSIFIED)	17977	8	18.33	-	0.44	0
**Cluster 13**						
Genes List:	CDH1,CDK5RAP2,CEP135,CEP152,CTNNA1,ECT2,KCNQ1,LIMA1,PLK4,RACGAP1,WDR62,PTPRM,AKAP9,CEP170,TUBG1,KIF23					
Biological Process						
Bonferroni count:	1810					
PANTHER GO-Slim Biological Process	Homo sapiens - REFLIST (20595)	BC_13.txt (17)	BC_13.txt (expected)	BC_13.txt (over/under)	BC_13.txt (fold Enrichment)	BC_13.txt (P-value)
microtubule cytoskeleton organization involved in mitosis (GO:1902850)	47	3	0.04	+	77.33	0.0161
cell cycle process (GO:0022402)	434	5	0.36	+	13.96	0.0388
Unclassified (UNCLASSIFIED)	9793	6	8.08	-	0.74	0
Molecular Function						
Bonferroni count:	510					
PANTHER GO-Slim Molecular Function	Homo sapiens - REFLIST (20595)	BC_13.txt (17)	BC_13.txt (expected)	BC_13.txt (over/under)	BC_13.txt (fold Enrichment)	BC_13.txt (P-value)
Unclassified (UNCLASSIFIED)	10792	7	8.91	-	0.79	0
Cellular Component						
Bonferroni count:	438					
PANTHER GO-Slim Cellular Component	Homo sapiens - REFLIST (20595)	BC_13.txt (17)	BC_13.txt (expected)	BC_13.txt (over/under)	BC_13.txt (fold Enrichment)	BC_13.txt (P-value)
spindle (GO:0005819)	77	3	0.06	+	47.2	0.0161
microtubule cytoskeleton (GO:0015630)	432	5	0.36	+	14.02	0.00919
cytoskeleton (GO:0005856)	667	6	0.55	+	10.9	0.00472
Unclassified (UNCLASSIFIED)	9302	7	7.68	-	0.91	0

Pathway						
Bonferroni count:	156					
PANTHER Pathways	Homo sapiens - REFLIST (20595)	BC_13.txt (17)	BC_13.txt (expected)	BC_13.txt (over/under)	BC_13.txt (fold Enrichment)	BC_13.txt (P-value)
Unclassified (UNCLASSIFIED)	17977	15	14.84	+	1.01	0
**Cluster 14**						
Genes List:	CDH2,CTNNA2,CTNNB1,EFNB3,EPHA2,EPHA3,EPHA4,ID2,JUP,PDLIM5,PPARGC1A,PRKAA2,TCF4,TCF7L2,IQGAP1,FHIT,NR5A2,SATB1,EFNA1,EFNA5,ID1					
Biological Process						
Bonferroni count:	1810					
PANTHER GO-Slim Biological Process	Homo sapiens - REFLIST (20595)	BC_14.txt (21)	BC_14.txt (expected)	BC_14.txt (over/under)	BC_14.txt (fold Enrichment)	BC_14.txt (P-value)
axon guidance (GO:0007411)	104	6	0.11	+	56.58	1.85E-06
neuron projection guidance (GO:0097485)	104	6	0.11	+	56.58	1.85E-06
axonogenesis (GO:0007409)	140	7	0.14	+	49.04	1.57E-07
axon development (GO:0061564)	143	7	0.15	+	48.01	1.81E-07
cell morphogenesis involved in neuron differentiation (GO:0048667)	158	7	0.16	+	43.45	3.55E-07
neuron projection morphogenesis (GO:0048812)	177	7	0.18	+	38.79	7.62E-07
cell projection morphogenesis (GO:0048858)	178	7	0.18	+	38.57	7.91E-07
plasma membrane bounded cell projection morphogenesis (GO:0120039)	178	7	0.18	+	38.57	7.91E-07
cell morphogenesis involved in differentiation (GO:0000904)	178	7	0.18	+	38.57	7.91E-07
cell part morphogenesis (GO:0032990)	180	7	0.18	+	38.14	8.53E-07
cellular component morphogenesis (GO:0032989)	204	7	0.21	+	33.65	1.98E-06
taxis (GO:0042330)	176	6	0.18	+	33.43	3.83E-05
chemotaxis (GO:0006935)	176	6	0.18	+	33.43	3.83E-05
neuron projection development (GO:0031175)	216	7	0.22	+	31.78	2.92E-06
neuron development (GO:0048666)	235	7	0.24	+	29.21	5.15E-06
cell morphogenesis	241	7	0.25	+	28.49	6.11E-06

(GO:0000902)						
transmembrane receptor protein tyrosine kinase signaling pathway (GO:0007169)	212	6	0.22	+	27.76	0.000112
neuron differentiation (GO:0030182)	320	7	0.33	+	21.45	4.12E-05
generation of neurons (GO:0048699)	348	7	0.35	+	19.73	7.24E-05
neurogenesis (GO:0022008)	366	7	0.37	+	18.76	0.000102
enzyme linked receptor protein signaling pathway (GO:0007167)	317	6	0.32	+	18.56	0.00114
cell development (GO:0048468)	382	7	0.39	+	17.97	0.000135
plasma membrane bounded cell projection organization (GO:0120036)	395	7	0.4	+	17.38	0.000169
cell projection organization (GO:0030030)	401	7	0.41	+	17.12	0.000187
locomotion (GO:0040011)	384	6	0.39	+	15.32	0.00341
nervous system development (GO:0007399)	479	7	0.49	+	14.33	0.000613
anatomical structure morphogenesis (GO:0009653)	492	7	0.5	+	13.95	0.000732
response to external stimulus (GO:0009605)	611	7	0.62	+	11.24	0.00307
cell differentiation (GO:0030154)	802	9	0.82	+	11.01	7.5E-05
cellular developmental process (GO:0048869)	806	9	0.82	+	10.95	7.83E-05
movement of cell or subcellular component (GO:0006928)	595	6	0.61	+	9.89	0.0405
system development (GO:0048731)	804	8	0.82	+	9.76	0.00131
multicellular organism development (GO:0007275)	906	8	0.92	+	8.66	0.00318
developmental process (GO:0032502)	1271	11	1.3	+	8.49	1.84E-05
response to chemical (GO:0042221)	822	7	0.84	+	8.35	0.0213
anatomical structure development (GO:0048856)	1126	9	1.15	+	7.84	0.00131
multicellular organismal process (GO:0032501)	1385	10	1.41	+	7.08	0.000624
regulation of transcription by	1591	9	1.62	+	5.55	0.0225

RNA polymerase II (GO:0006357)						
transcription by RNA polymerase II (GO:0006366)	1635	9	1.67	+	5.4	0.0281
signaling (GO:0023052)	2218	11	2.26	+	4.86	0.00524
cell communication (GO:0007154)	2230	11	2.27	+	4.84	0.00552
signal transduction (GO:0007165)	2062	10	2.1	+	4.76	0.0232
regulation of cellular metabolic process (GO:0031323)	2835	13	2.89	+	4.5	0.000807
regulation of metabolic process (GO:0019222)	3093	13	3.15	+	4.12	0.00224
regulation of cellular process (GO:0050794)	5092	19	5.19	+	3.66	6.73E-07
regulation of biological process (GO:0050789)	5324	19	5.43	+	3.5	1.52E-06
biological regulation (GO:0065007)	5797	19	5.91	+	3.21	7.22E-06
cellular process (GO:0009987)	9951	20	10.15	+	1.97	0.0116
Molecular Function						
Bonferroni count:	510					
PANTHER GO-Slim Molecular Function	Homo sapiens - REFLIST (20595)	BC_14.txt (21)	BC_14.txt (expected)	BC_14.txt (over/under)	BC_14.txt (fold Enrichment)	BC_14.txt (P-value)
cadherin binding (GO:0045296)	50	3	0.05	+	58.84	0.0105
transmembrane receptor protein tyrosine kinase activity (GO:0004714)	54	3	0.06	+	54.48	0.0131
transmembrane receptor protein kinase activity (GO:0019199)	64	3	0.07	+	45.97	0.0213
transcription coactivator activity (GO:0003713)	78	3	0.08	+	37.72	0.0377
protein tyrosine kinase activity (GO:0004713)	83	3	0.08	+	35.45	0.045
molecular_function (GO:0003674)	9803	20	10	+	2	0.00274
Cellular Component						
Bonferroni count:	438					
PANTHER GO-Slim Cellular Component	Homo sapiens - REFLIST (20595)	BC_14.txt (21)	BC_14.txt (expected)	BC_14.txt (over/under)	BC_14.txt (fold Enrichment)	BC_14.txt (P-value)
adherens junction (GO:0005912)	68	4	0.07	+	57.69	0.000343
extrinsic component of plasma membrane (GO:0019897)	87	3	0.09	+	33.82	0.0443
cell-cell junction (GO:0005911)	129	4	0.13	+	30.41	0.00399

anchoring junction (GO:0070161)	168	4	0.17	+	23.35	0.011
intrinsic component of plasma membrane (GO:0031226)	798	6	0.81	+	7.37	0.0496
cellular anatomical entity (GO:0110165)	11122	20	11.34	+	1.76	0.0312
cellular_component (GO:0005575)	11293	20	11.52	+	1.74	0.0348
Pathway						
Bonferroni count:	156					
PANTHER Pathways	Homo sapiens - REFLIST (20595)	BC_14.txt (21)	BC_14.txt (expected)	BC_14.txt (over/under)	BC_14.txt (fold Enrichment)	BC_14.txt (P-value)
Alzheimer disease-presenilin pathway (P00004)	127	4	0.13	+	30.89	0.00134
Cadherin signaling pathway (P00012)	164	4	0.17	+	23.92	0.00356
Wnt signaling pathway (P00057)	317	4	0.32	+	12.37	0.0437
Unclassified (UNCLASSIFIED)	17977	12	18.33	-	0.65	0
**Cluster 15**						
Genes List:	COL1A1,FN1,IL1R1,IRAK1,KDR,KRT19,PDGFA,PDGFB,PDGFRA,PDGFRB,PELI1,PELI2,RASA1,TGM2,MBP,PELI3,PDGFC					
Biological Process						
Bonferroni count:	1810					
PANTHER GO-Slim Biological Process	Homo sapiens - REFLIST (20595)	BC_15.txt (18)	BC_15.txt (expected)	BC_15.txt (over/under)	BC_15.txt (fold Enrichment)	BC_15.txt (P-value)
regulation of phosphatidylinositol 3-kinase signaling (GO:0014066)	9	3	0.01	+	> 100	0.000222
phosphatidylinositol 3-kinase signaling (GO:0014065)	16	3	0.01	+	> 100	0.000972
positive regulation of MAP kinase activity (GO:0043406)	34	3	0.03	+	> 100	0.00772
inositol lipid-mediated signaling (GO:0048017)	35	3	0.03	+	98.07	0.00838
phosphatidylinositol-mediated signaling (GO:0048015)	35	3	0.03	+	98.07	0.00838
positive regulation of protein serine/threonine kinase activity (GO:0071902)	39	3	0.03	+	88.01	0.0114
protein autophosphorylation (GO:0046777)	40	3	0.03	+	85.81	0.0122
positive regulation	56	4	0.05	+	81.73	0.000348

of cell migration (GO:0030335)						
positive regulation of ERK1 and ERK2 cascade (GO:0070374)	45	3	0.04	+	76.28	0.0171
positive regulation of cell motility (GO:2000147)	60	4	0.05	+	76.28	0.000453
positive regulation of cellular component movement (GO:0051272)	61	4	0.05	+	75.03	0.000482
positive regulation of locomotion (GO:0040017)	61	4	0.05	+	75.03	0.000482
regulation of ERK1 and ERK2 cascade (GO:0070372)	48	3	0.04	+	71.51	0.0205
ERK1 and ERK2 cascade (GO:0070371)	48	3	0.04	+	71.51	0.0205
regulation of MAP kinase activity (GO:0043405)	56	3	0.05	+	61.29	0.0319
positive regulation of kinase activity (GO:0033674)	128	6	0.11	+	53.63	2.12E-06
positive regulation of transferase activity (GO:0051347)	136	6	0.12	+	50.48	3.02E-06
regulation of cell migration (GO:0030334)	111	4	0.1	+	41.23	0.00479
regulation of cell motility (GO:2000145)	118	4	0.1	+	38.79	0.00607
regulation of locomotion (GO:0040012)	123	4	0.11	+	37.21	0.00712
regulation of cellular component movement (GO:0051270)	125	4	0.11	+	36.61	0.00758
regulation of kinase activity (GO:0043549)	206	6	0.18	+	33.33	3.34E-05
transmembrane receptor protein tyrosine kinase signaling pathway (GO:0007169)	212	6	0.19	+	32.38	3.95E-05
regulation of transferase activity (GO:0051338)	215	6	0.19	+	31.93	4.28E-05
positive regulation of phosphorylation (GO:0042327)	293	6	0.26	+	23.43	0.000257
positive regulation of phosphorus metabolic process (GO:0010562)	300	6	0.26	+	22.88	0.000295
positive regulation of phosphate metabolic process (GO:0045937)	300	6	0.26	+	22.88	0.000295
cell migration	263	5	0.23	+	21.75	0.00483

(GO:0016477)						
enzyme linked receptor protein signaling pathway (GO:0007167)	317	6	0.28	+	21.66	0.000405
positive regulation of catalytic activity (GO:0043085)	323	6	0.28	+	21.25	0.000452
positive regulation of molecular function (GO:0044093)	421	7	0.37	+	19.02	7.49E-05
localization of cell (GO:0051674)	304	5	0.27	+	18.82	0.00968
cell motility (GO:0048870)	304	5	0.27	+	18.82	0.00968
regulation of phosphorylation (GO:0042325)	393	6	0.34	+	17.47	0.0014
regulation of phosphorus metabolic process (GO:0051174)	419	6	0.37	+	16.38	0.00202
regulation of phosphate metabolic process (GO:0019220)	419	6	0.37	+	16.38	0.00202
locomotion (GO:0040011)	384	5	0.34	+	14.9	0.0295
cell surface receptor signaling pathway (GO:0007166)	1003	10	0.88	+	11.41	4.34E-06
regulation of catalytic activity (GO:0050790)	646	6	0.56	+	10.63	0.0238
regulation of molecular function (GO:0065009)	806	7	0.7	+	9.94	0.00569
phosphorylation (GO:0016310)	704	6	0.62	+	9.75	0.0385
signal transduction (GO:0007165)	2062	10	1.8	+	5.55	0.00382
signaling (GO:0023052)	2218	10	1.94	+	5.16	0.00745
cell communication (GO:0007154)	2230	10	1.95	+	5.13	0.00782
cellular response to stimulus (GO:0051716)	2522	10	2.2	+	4.54	0.0238
response to stimulus (GO:0050896)	3027	11	2.65	+	4.16	0.0147
Unclassified (UNCLASSIFIED)	9793	4	8.56	-	0.47	0
Molecular Function						
Bonferroni count:	510					
PANTHER GO-Slim Molecular Function	Homo sapiens - REFLIST (20595)	BC_15.txt (18)	BC_15.txt (expected)	BC_15.txt (over/under)	BC_15.txt (fold Enrichment)	BC_15.txt (P-value)
growth factor receptor binding (GO:0070851)	39	3	0.03	+	88.01	0.0032
transmembrane receptor protein tyrosine kinase activity (GO:0004714)	54	3	0.05	+	63.56	0.0081

transmembrane receptor protein kinase activity (GO:0019199)	64	3	0.06	+	53.63	0.0132
protein tyrosine kinase activity (GO:0004713)	83	3	0.07	+	41.36	0.0279
catalytic activity, acting on a protein (GO:0140096)	1551	8	1.36	+	5.9	0.0118
transferase activity (GO:0016740)	1572	8	1.37	+	5.82	0.013
Unclassified (UNCLASSIFIED)	10792	5	9.43	-	0.53	0
Cellular Component						
Bonferroni count:	438					
PANTHER GO-Slim Cellular Component	Homo sapiens - REFLIST (20595)	BC_15.txt (18)	BC_15.txt (expected)	BC_15.txt (over/under)	BC_15.txt (fold Enrichment)	BC_15.txt (P-value)
Unclassified (UNCLASSIFIED)	9302	9	8.13	+	1.11	0
Pathway						
Bonferroni count:	156					
PANTHER Pathways	Homo sapiens - REFLIST (20595)	BC_15.txt (18)	BC_15.txt (expected)	BC_15.txt (over/under)	BC_15.txt (fold Enrichment)	BC_15.txt (P-value)
Angiogenesis (P00005)	175	7	0.15	+	45.77	1.7E-08
PDGF signaling pathway (P00047)	147	5	0.13	+	38.92	2.52E-05
Unclassified (UNCLASSIFIED)	17977	7	15.71	-	0.45	0
**Cluster 16**						
Genes List:	EGLN1,EIF4A1,EIF4G1,EPAS1,FOXO3,HIF1A,MUC1,PABPC1,PDCD4,FBP1,ESRRA					
Biological Process						
Bonferroni count:	1810					
PANTHER GO-Slim Biological Process	Homo sapiens - REFLIST (20595)	BC_16.txt (11)	BC_16.txt (expected)	BC_16.txt (over/under)	BC_16.txt (fold Enrichment)	BC_16.txt (P-value)
cellular response to hypoxia (GO:0071456)	8	3	0	+	> 100	3.37E-05
response to hypoxia (GO:0001666)	22	3	0.01	+	> 100	0.000468
response to oxygen levels (GO:0070482)	22	3	0.01	+	> 100	0.000468
response to decreased oxygen levels (GO:0036293)	22	3	0.01	+	> 100	0.000468
response to abiotic stimulus (GO:0009628)	97	3	0.05	+	57.91	0.0322
Unclassified (UNCLASSIFIED)	9793	4	5.23	-	0.76	0

Molecular Function						
Bonferroni count:	510					
PANTHER GO-Slim Molecular Function	Homo sapiens - REFLIST (20595)	BC_16.txt (11)	BC_16.txt (expected)	BC_16.txt (over/under)	BC_16.txt (fold Enrichment)	BC_16.txt (P-value)
nucleic acid binding (GO:0003676)	2248	7	1.2	+	5.83	0.021
Cellular Component						
Bonferroni count:	438					
PANTHER GO-Slim Cellular Component	Homo sapiens - REFLIST (20595)	BC_16.txt (11)	BC_16.txt (expected)	BC_16.txt (over/under)	BC_16.txt (fold Enrichment)	BC_16.txt (P-value)
Pathway						
Bonferroni count:	156					
PANTHER Pathways	Homo sapiens - REFLIST (20595)	BC_16.txt (11)	BC_16.txt (expected)	BC_16.txt (over/under)	BC_16.txt (fold Enrichment)	BC_16.txt (P-value)
Unclassified (UNCLASSIFIED)	17977	7	9.6	-	0.73	0
**Cluster 17**						
Genes List:	GRK5,MCM4,MCM5,MCM7,NFKBIA,REL,MAP3K8					
Biological Process						
Bonferroni count:	1810					
PANTHER GO-Slim Biological Process	Homo sapiens - REFLIST (20595)	BC_17.txt (7)	BC_17.txt (expected)	BC_17.txt (over/under)	BC_17.txt (fold Enrichment)	BC_17.txt (P-value)
pre-replicative complex assembly involved in nuclear cell cycle DNA replication (GO:0006267)	7	3	0	+	> 100	5.21E-06
double-strand break repair via break-induced replication (GO:0000727)	11	3	0	+	> 100	1.58E-05
cell cycle DNA replication (GO:0044786)	15	3	0.01	+	> 100	3.54E-05
nuclear DNA replication (GO:0033260)	15	3	0.01	+	> 100	3.54E-05
DNA replication initiation (GO:0006270)	17	3	0.01	+	> 100	4.94E-05
DNA-dependent DNA replication (GO:0006261)	60	3	0.02	+	> 100	0.00171
double-strand break repair via homologous recombination (GO:0000724)	64	3	0.02	+	> 100	0.00206
recombinational repair (GO:0000725)	66	3	0.02	+	> 100	0.00226
DNA replication (GO:0006260)	77	3	0.03	+	> 100	0.00353
protein-DNA complex assembly	91	3	0.03	+	96.99	0.00575

(GO:0065004)						
double-strand break repair (GO:0006302)	99	3	0.03	+	89.16	0.00736
DNA biosynthetic process (GO:0071897)	101	3	0.03	+	87.39	0.0078
DNA recombination (GO:0006310)	109	3	0.04	+	80.98	0.00975
protein-DNA complex subunit organization (GO:0071824)	119	3	0.04	+	74.17	0.0126
cellular response to stress (GO:0033554)	500	4	0.17	+	23.54	0.0211
Unclassified (UNCLASSIFIED)	9793	3	3.33	-	0.9	0
Molecular Function						
Bonferroni count:	510					
PANTHER GO-Slim Molecular Function	Homo sapiens - REFLIST (20595)	BC_17.txt (7)	BC_17.txt (expected)	BC_17.txt (over/under)	BC_17.txt (fold Enrichment)	BC_17.txt (P-value)
DNA replication origin binding (GO:0003688)	18	3	0.01	+	> 100	1.62E-05
single-stranded DNA binding (GO:0003697)	39	3	0.01	+	> 100	0.00014
Unclassified (UNCLASSIFIED)	10792	3	3.67	-	0.82	0
Cellular Component						
Bonferroni count:	438					
PANTHER GO-Slim Cellular Component	Homo sapiens - REFLIST (20595)	BC_17.txt (7)	BC_17.txt (expected)	BC_17.txt (over/under)	BC_17.txt (fold Enrichment)	BC_17.txt (P-value)
MCM core complex (GO:0097373)	30	3	0.01	+	> 100	5.71E-05
MCM complex (GO:0042555)	30	3	0.01	+	> 100	5.71E-05
Unclassified (UNCLASSIFIED)	9302	3	3.16	-	0.95	0
Pathway						
Bonferroni count:	156					
PANTHER Pathways	Homo sapiens - REFLIST (20595)	BC_17.txt (7)	BC_17.txt (expected)	BC_17.txt (over/under)	BC_17.txt (fold Enrichment)	BC_17.txt (P-value)
Toll receptor signaling pathway (P00054)	58	3	0.02	+	> 100	0.000134
Apoptosis signaling pathway (P00006)	118	3	0.04	+	74.8	0.00106
**Cluster 18**						
Genes List:	MDM2,RBBP6,RNF144B,RRM1,RRM2B,TP63,TP73,RRM2,PPP1R13L,SIAH1					
Biological Process						
Bonferroni count:	1810					

PANTHER GO-Slim Biological Process	Homo sapiens - REFLIST (20595)	BC_18.txt (10)	BC_18.txt (expected)	BC_18.txt (over/under)	BC_18.txt (fold Enrichment)	BC_18.txt (P-value)
nucleotide biosynthetic process (GO:0009165)	117	3	0.06	+	52.81	0.0406
nucleoside phosphate biosynthetic process (GO:1901293)	119	3	0.06	+	51.92	0.0427
nucleobase-containing compound biosynthetic process (GO:0034654)	2297	7	1.12	+	6.28	0.0346
heterocycle biosynthetic process (GO:0018130)	2322	7	1.13	+	6.21	0.0372
aromatic compound biosynthetic process (GO:0019438)	2323	7	1.13	+	6.21	0.0373
organic cyclic compound biosynthetic process (GO:1901362)	2350	7	1.14	+	6.13	0.0403
Molecular Function						
Bonferroni count:	510					
PANTHER GO-Slim Molecular Function	Homo sapiens - REFLIST (20595)	BC_18.txt (10)	BC_18.txt (expected)	BC_18.txt (over/under)	BC_18.txt (fold Enrichment)	BC_18.txt (P-value)
ubiquitin protein ligase activity (GO:0061630)	191	3	0.09	+	32.35	0.0479
Cellular Component						
Bonferroni count:	438					
PANTHER GO-Slim Cellular Component	Homo sapiens - REFLIST (20595)	BC_18.txt (10)	BC_18.txt (expected)	BC_18.txt (over/under)	BC_18.txt (fold Enrichment)	BC_18.txt (P-value)
Pathway						
Bonferroni count:	156					
PANTHER Pathways	Homo sapiens - REFLIST (20595)	BC_18.txt (10)	BC_18.txt (expected)	BC_18.txt (over/under)	BC_18.txt (fold Enrichment)	BC_18.txt (P-value)
P53 pathway feedback loops 1 (P04392)	7	3	0	+	> 100	1.54E-06
De novo pyrimidine deoxyribonucleotide biosynthesis (P02739)	15	3	0.01	+	> 100	1.04E-05
De novo purine biosynthesis (P02738)	30	3	0.01	+	> 100	6.95E-05
p53 pathway feedback loops 2 (P04398)	51	4	0.02	+	> 100	1.47E-06
p53 pathway (P00059)	89	5	0.04	+	> 100	6.85E-08
Unclassified (UNCLASSIFIED)	17977	3	8.73	-	0.34	0
**Other than Core**						
Genes List:	ABCC8,ABCG5,A					

	BCG8,ACE2,ACKR1,ACSS1,ACTN2,ACVR2A,ACVRL1,ADAMTS13,ADCYAP1,ADCYAP1R1,ADRA2A,AGR2,AGT,ALK,ALOX5,ANGPT2,ANGPTL2,ANK2,ANK3,APOE,ARHGDIB,ARPIN,ASF1B,ATP6V1B2,BAX,BCL2L1,BMP1,BMP2,BMP4,BMP7,BMPR1A,BMPR1B,BMPR2,BRCA2,C3,C8A,C9,CABP1,CACNA1C,CACNA1D,CALCRL,CAMK2A,CAMK2B,CAMK2D,CAMK2G,CASK,CASKIN1,CASP6,CASP7,CBFA2T3,CBFB,CCL11,CCL14,CCL16,CCL2,CCL4,CCL5,CCL8,CCNC,CCR1,CCR5,CD28,CD4,CD44,CD86,CD9,CDH23,CDK8,CDT1,CEACAM1,CEACAM5,CEBPA,CFB,CFH,CFTR,CHD3,CHD4,CHRD,CHRM3,CLDN18,CLDN19,CLEC1B,CLEC7A,CNTN1,CNTNAP4,COL3A1,COPS5,COTL1,CRYAB,CTSS,CXCL1,CXCL5,CXCL8,CXCR1,CXCR4,CYFIP1,DAB2,DCC,DCUN1D2,DDR1,DISC1,DLAT,DLC1,DNMT1,DOCK2,DPP4,DSP,E2F2,ELN,ENG,ETFA,ETFB,EXO1,EZH2,EZR,F13A1,F2,F3,F7,F8,F9,FANCD2,FANCI,FBLN5,FCGR2B,FGF10,FGF12,FGF13,FGF2,FGF5,FGF7,FGFR2,FGFR3,FGFR4,FHL2,FKBP1A,FLT1,FOXM1,FOXO1,FSCN1,FYCO1,GATA1,GATA2,GATA3,GDNF,GFRA1,GINS3,GPC3,GRIA2,GRIA3,GRIN2A,GTF2H1,GTF2H5,HAVCR2,HBEGF,HDAC5,HJURP,HLA-C,HLA-DMA,HLA-DMB,HLA-DOA,HLA-G,HMGA1,HMGB1,HSPB1,HTRA1,ICAM1,IFIT3,IFNGR1,IGF2,IGFBP1,IGFBP3,IL16,IL1B,IL1RAP,IL2RA,IL2RB,IL2RG,IL32,IM					

	MT,INHBA,ITGA2,ITGA3,ITGA4,ITGA5,ITGA7,ITGAL,ITGAV,ITGAX,ITGB2,ITGB6,ITK,JAK3,JARID2,KANK1,KCNJ11,KCNMA1,KCNN2,KIF15,KIF21A,KIF5B,KLF1,KRT14,KRT18,KRT5,KRT6A,KRT8,LCN2,LCP2,LDB1,LDB2,LGALS3,LHX4,LILRB2,LMNB1,LMO3,LRG1,LRP1,LRRK2,LYL1,MAP1LC3A,MAPK14,MAPT,MASP1,MBL2,MCAM,MCL1,MCM2,MCM6,MED25,MFN2,MKI67,MLPH,MMP14,MMP9,MRPL12,MRPS27,MST1,MST1R,MTA3,MUC2,MYB,MYO10,MYO6,NCF1,NCF2,NCF4,NCKAP1,NDUFA9,NEDD4L,NFIB,NFIC,NOD2,NOS1,NOTUM,NR2F2,NR4A1,NRP1,NTN1,NTN4,NUF2,OBSCN,OBSL1,OCLN,OGDH,OGDHL,OLFM4,ORAI2,ORC1,PARD3,PDE5A,PDK3,PDPN,PGF,PINK1,PLXNA2,PLXNB1,PODXL,PPARA,PPBP,PRDM14,PRKCH,PRKG1,PRL,PRLR,PRTN3,PTGES3,PTN,PTPN13,PTPRC,PTPRR,PTPRZ1,RAB27A,RAC2,RAMP1,RAPH1,RASIP1,RASSF5,RBBP8,RBL1,RCOR1,REN,RET,RGMA,RGS4,RILP,RND1,RNF165,ROBO1,RUNX1,RUVBL2,RXRA,RYR2,S,S100A8,S100A9,SCN1A,SCN5A,SDC1,SDC2,SDC4,SELE,SEMA3A,SERPINA5,SERPINC1,SETD7,SH3GL3,SH3RF3,SIAH2,SIRT3,SLC11A2,SLC12A2,SLC15A3,SLC25A4,SLC38A9,SLC8A1,SLC9A3R1,SLIT2,SMAD6,SNCA,SNCAIP,SNTA1,SPARC,SPC24,SPI1,SPTA1,SPTB,SPTLC3,SPTSSB,SQSTM1,SREBF1,STAT1,STIP1,STK11,STK26,STK4,STUB1,TAL1,TBXA2R,TCAP,TEK,TF,TFDP1,TFDP2,					

	TFRC,TGFB2,TGFB3,TGFBR1,TGFBR2,TGFBR3,TIAM1,TLR2,TNFRSF10D,TNFRSF13B,TNFSF10,TNFSF13B,TNIK,TNKS2,TNS1,TOM1L2,TOP2A,TRIB1,TRPC3,TTK,TTN,UBB,UBE2B,UBE2M,UBR1,UNC5B,UNC5C,USH1C,USH1G,USP11,USP2,USP33,VDR,VLDLR,VWF,WAS,WASF1,WIPF1,WNT7A,ZFPM1,ZNF827					
Biological Process						
Bonferroni count:	1810					
PANTHER GO-Slim Biological Process	Homo sapiens - REFLIST (20595)	3_Otherthan_Core_Genes.txt (418)	3_Otherthan_Core_Genes.txt (expected)	3_Otherthan_Core_Genes.txt (over/under)	3_Otherthan_Core_Genes.txt (fold Enrichment)	3_Otherthan_Core_Genes.txt (P-value)
positive chemotaxis (GO:0050918)	9	5	0.18	+	27.37	0.0095
neutrophil migration (GO:1990266)	49	13	0.99	+	13.07	3.94E-07
granulocyte chemotaxis (GO:0071621)	49	13	0.99	+	13.07	3.94E-07
leukocyte chemotaxis (GO:0030595)	58	15	1.18	+	12.74	2.17E-08
positive regulation of ERK1 and ERK2 cascade (GO:0070374)	45	11	0.91	+	12.04	2.04E-05
granulocyte migration (GO:0097530)	55	13	1.12	+	11.65	1.34E-06
cell chemotaxis (GO:0060326)	64	15	1.3	+	11.55	7.2E-08
myeloid leukocyte migration (GO:0097529)	61	14	1.24	+	11.31	4.12E-07
regulation of ERK1 and ERK2 cascade (GO:0070372)	48	11	0.97	+	11.29	3.64E-05
ERK1 and ERK2 cascade (GO:0070371)	48	11	0.97	+	11.29	3.64E-05
leukocyte migration (GO:0050900)	71	15	1.44	+	10.41	2.56E-07
response to interleukin-1 (GO:0070555)	35	7	0.71	+	9.85	0.0314
lymphocyte migration (GO:0072676)	36	7	0.73	+	9.58	0.0368
inflammatory response (GO:0006954)	128	20	2.6	+	7.7	2.87E-08
transmembrane receptor protein serine/threonine kinase signaling pathway (GO:0007178)	97	15	1.97	+	7.62	1.15E-05

BMP signaling pathway (GO:0030509)	52	8	1.06	+	7.58	0.043
cellular response to BMP stimulus (GO:0071773)	53	8	1.08	+	7.44	0.0486
response to BMP (GO:0071772)	53	8	1.08	+	7.44	0.0486
cell migration (GO:0016477)	263	39	5.34	+	7.31	2.42E-17
response to growth factor (GO:0070848)	131	19	2.66	+	7.15	2.91E-07
cellular response to growth factor stimulus (GO:0071363)	131	19	2.66	+	7.15	2.91E-07
taxis (GO:0042330)	176	25	3.57	+	7	5.44E-10
chemotaxis (GO:0006935)	176	25	3.57	+	7	5.44E-10
positive regulation of MAPK cascade (GO:0043410)	103	14	2.09	+	6.7	0.000158
localization of cell (GO:0051674)	304	39	6.17	+	6.32	2.29E-15
cell motility (GO:0048870)	304	39	6.17	+	6.32	2.29E-15
cytokine-mediated signaling pathway (GO:0019221)	139	17	2.82	+	6.03	2.73E-05
positive regulation of cell population proliferation (GO:0008284)	90	11	1.83	+	6.02	0.00978
positive regulation of protein phosphorylation (GO:0001934)	241	29	4.89	+	5.93	3.05E-10
positive regulation of phosphorus metabolic process (GO:0010562)	300	36	6.09	+	5.91	3.49E-13
positive regulation of phosphate metabolic process (GO:0045937)	300	36	6.09	+	5.91	3.49E-13
positive regulation of phosphorylation (GO:0042327)	293	35	5.95	+	5.89	1.05E-12
MAPK cascade (GO:0000165)	146	17	2.96	+	5.74	5.29E-05
positive regulation of protein modification process (GO:0031401)	261	30	5.3	+	5.66	3.4E-10
cellular response to cytokine stimulus (GO:0071345)	148	17	3	+	5.66	6.34E-05
locomotion (GO:0040011)	384	44	7.79	+	5.65	6.46E-16
enzyme linked receptor protein signaling pathway (GO:0007167)	317	36	6.43	+	5.6	1.67E-12
regulation of MAPK cascade (GO:0043408)	133	15	2.7	+	5.56	0.000504

signal transduction by protein phosphorylation (GO:0023014)	152	17	3.09	+	5.51	9.05E-05
response to cytokine (GO:0034097)	155	17	3.15	+	5.4	0.000117
regulation of cell migration (GO:0030334)	111	12	2.25	+	5.33	0.0117
regulation of locomotion (GO:0040012)	123	13	2.5	+	5.21	0.00607
positive regulation of transferase activity (GO:0051347)	136	14	2.76	+	5.07	0.0034
regulation of cell motility (GO:2000145)	118	12	2.39	+	5.01	0.0207
positive regulation of multicellular organismal process (GO:0051240)	118	12	2.39	+	5.01	0.0207
positive regulation of kinase activity (GO:0033674)	128	13	2.6	+	5	0.00907
animal organ morphogenesis (GO:0009887)	121	12	2.46	+	4.89	0.0261
transmembrane receptor protein tyrosine kinase signaling pathway (GO:0007169)	212	21	4.3	+	4.88	1.73E-05
regulation of cellular component movement (GO:0051270)	125	12	2.54	+	4.73	0.0352
positive regulation of cellular protein metabolic process (GO:0032270)	340	32	6.9	+	4.64	7.38E-09
positive regulation of protein metabolic process (GO:0051247)	366	34	7.43	+	4.58	2.14E-09
regulation of protein phosphorylation (GO:0001932)	327	30	6.64	+	4.52	6.33E-08
regulation of phosphorylation (GO:0042325)	393	36	7.98	+	4.51	6.62E-10
regulation of cell population proliferation (GO:0042127)	167	15	3.39	+	4.43	0.00698
cellular response to organic substance (GO:0071310)	468	42	9.5	+	4.42	1.23E-11
response to endogenous stimulus (GO:0009719)	259	23	5.26	+	4.38	2.28E-05
regulation of phosphorus metabolic process (GO:0051174)	419	37	8.5	+	4.35	8.55E-10
regulation of phosphate metabolic	419	37	8.5	+	4.35	8.55E-10

process (GO:0019220)						
cellular response to endogenous stimulus (GO:0071495)	244	21	4.95	+	4.24	0.000162
movement of cell or subcellular component (GO:0006928)	595	51	12.08	+	4.22	7.2E-14
response to organic substance (GO:0010033)	551	47	11.18	+	4.2	1.62E-12
positive regulation of signal transduction (GO:0009967)	294	25	5.97	+	4.19	1.18E-05
protein phosphorylation (GO:0006468)	541	46	10.98	+	4.19	3.75E-12
cell population proliferation (GO:0008283)	190	16	3.86	+	4.15	0.00721
cell part morphogenesis (GO:0032990)	180	15	3.65	+	4.11	0.0162
regulation of protein modification process (GO:0031399)	378	31	7.67	+	4.04	3.97E-07
positive regulation of intracellular signal transduction (GO:1902533)	185	15	3.75	+	3.99	0.0219
positive regulation of cell communication (GO:0010647)	321	26	6.52	+	3.99	1.49E-05
positive regulation of signaling (GO:0023056)	321	26	6.52	+	3.99	1.49E-05
neuron projection development (GO:0031175)	216	17	4.38	+	3.88	0.00843
cellular component morphogenesis (GO:0032989)	204	16	4.14	+	3.86	0.0167
cellular response to chemical stimulus (GO:0070887)	590	46	11.97	+	3.84	7.2E-11
positive regulation of catalytic activity (GO:0043085)	323	25	6.56	+	3.81	6.67E-05
neuron development (GO:0048666)	235	18	4.77	+	3.77	0.00633
cell surface receptor signaling pathway (GO:0007166)	1003	75	20.36	+	3.68	1.96E-18
positive regulation of cellular metabolic process (GO:0031325)	768	57	15.59	+	3.66	3.64E-13
phosphorylation (GO:0016310)	704	52	14.29	+	3.64	1.04E-11
positive regulation of metabolic process (GO:0009893)	841	60	17.07	+	3.52	2.96E-13
positive regulation	707	50	14.35	+	3.48	1.74E-10

of nitrogen compound metabolic process (GO:0051173)						
response to chemical (GO:0042221)	822	58	16.68	+	3.48	1.59E-12
anatomical structure morphogenesis (GO:0009653)	492	34	9.99	+	3.4	3.43E-06
neuron differentiation (GO:0030182)	320	22	6.49	+	3.39	0.00285
positive regulation of macromolecule metabolic process (GO:0010604)	753	50	15.28	+	3.27	1.61E-09
multicellular organism development (GO:0007275)	906	60	18.39	+	3.26	7.09E-12
positive regulation of response to stimulus (GO:0048584)	504	33	10.23	+	3.23	2.07E-05
regulation of cellular protein metabolic process (GO:0032268)	571	37	11.59	+	3.19	3.35E-06
regulation of protein metabolic process (GO:0051246)	608	39	12.34	+	3.16	1.56E-06
positive regulation of molecular function (GO:0044093)	421	27	8.54	+	3.16	0.000676
response to external stimulus (GO:0009605)	611	39	12.4	+	3.14	1.78E-06
system development (GO:0048731)	804	51	16.32	+	3.13	4.68E-09
generation of neurons (GO:0048699)	348	22	7.06	+	3.11	0.0102
neurogenesis (GO:0022008)	366	23	7.43	+	3.1	0.00687
nervous system development (GO:0007399)	479	30	9.72	+	3.09	0.000244
animal organ development (GO:0048513)	385	24	7.81	+	3.07	0.00482
regulation of multicellular organismal process (GO:0051239)	329	20	6.68	+	3	0.0461
defense response (GO:0006952)	395	24	8.02	+	2.99	0.00729
anatomical structure development (GO:0048856)	1126	68	22.85	+	2.98	6.09E-12
positive regulation of biological process (GO:0048518)	1455	87	29.53	+	2.95	6.31E-16
positive regulation of cellular process (GO:0048522)	1295	76	26.28	+	2.89	4.65E-13
cell development	382	22	7.75	+	2.84	0.0402

(GO:0048468)						
developmental process (GO:0032502)	1271	73	25.8	+	2.83	5.83E-12
regulation of catalytic activity (GO:0050790)	646	37	13.11	+	2.82	7.19E-05
cellular developmental process (GO:0048869)	806	46	16.36	+	2.81	1.59E-06
cell differentiation (GO:0030154)	802	45	16.28	+	2.76	4.05E-06
regulation of signal transduction (GO:0009966)	728	40	14.78	+	2.71	5.77E-05
regulation of intracellular signal transduction (GO:1902531)	441	24	8.95	+	2.68	0.0411
multicellular organismal process (GO:0032501)	1385	74	28.11	+	2.63	1.24E-10
regulation of cell communication (GO:0010646)	813	41	16.5	+	2.48	0.000395
regulation of signaling (GO:0023051)	813	41	16.5	+	2.48	0.000395
regulation of response to stimulus (GO:0048583)	1004	50	20.38	+	2.45	2.15E-05
regulation of molecular function (GO:0065009)	806	40	16.36	+	2.45	0.00079
phosphate-containing compound metabolic process (GO:0006796)	1090	54	22.12	+	2.44	8.53E-06
phosphorus metabolic process (GO:0006793)	1103	54	22.39	+	2.41	1.08E-05
response to stress (GO:0006950)	967	47	19.63	+	2.39	0.000155
signal transduction (GO:0007165)	2062	99	41.85	+	2.37	2.46E-12
immune system process (GO:0002376)	676	32	13.72	+	2.33	0.0425
cell communication (GO:0007154)	2230	103	45.26	+	2.28	6.99E-12
signaling (GO:0023052)	2218	102	45.02	+	2.27	1.21E-11
cellular response to stimulus (GO:0051716)	2522	114	51.19	+	2.23	4.7E-13
cellular protein modification process (GO:0006464)	1383	62	28.07	+	2.21	2.14E-05
protein modification process (GO:0036211)	1383	62	28.07	+	2.21	2.14E-05
response to stimulus (GO:0050896)	3027	134	61.44	+	2.18	2.07E-15
negative regulation of cellular process	1045	45	21.21	+	2.12	0.00625

(GO:0048523)						
macromolecule modification (GO:0043412)	1481	62	30.06	+	2.06	0.000202
cellular protein metabolic process (GO:0044267)	1906	69	38.68	+	1.78	0.00688
regulation of cellular process (GO:0050794)	5092	174	103.35	+	1.68	1.62E-10
protein metabolic process (GO:0019538)	2236	75	45.38	+	1.65	0.0321
regulation of biological process (GO:0050789)	5324	177	108.06	+	1.64	9.13E-10
regulation of cellular metabolic process (GO:0031323)	2835	94	57.54	+	1.63	0.00372
biological regulation (GO:0065007)	5797	189	117.66	+	1.61	4.72E-10
regulation of nitrogen compound metabolic process (GO:0051171)	2715	87	55.1	+	1.58	0.0395
regulation of metabolic process (GO:0019222)	3093	99	62.78	+	1.58	0.00856
cellular process (GO:0009987)	9951	255	201.97	+	1.26	0.000567
biological_process (GO:0008150)	10802	269	219.24	+	1.23	0.0026
Unclassified (UNCLASSIFIED)	9793	149	198.76	-	0.75	0
Molecular Function						
Bonferroni count:	510					
PANTHER GO-Slim Molecular Function	Homo sapiens - REFLIST (20595)	3_Otherthan_Core_Genes.txt (418)	3_Otherthan_Core_Genes.txt (expected)	3_Otherthan_Core_Genes.txt (over/under)	3_Otherthan_Core_Genes.txt (fold Enrichment)	3_Otherthan_Core_Genes.txt (P-value)
transmembrane receptor protein serine/threonine kinase activity (GO:0004675)	10	6	0.2	+	29.56	0.000206
fibroblast growth factor receptor binding (GO:0005104)	24	6	0.49	+	12.32	0.0121
transmembrane receptor protein kinase activity (GO:0019199)	64	15	1.3	+	11.55	2.03E-08
growth factor activity (GO:0008083)	39	9	0.79	+	11.37	0.000193
chemokine receptor binding (GO:0042379)	51	11	1.04	+	10.63	1.76E-05
growth factor receptor binding (GO:0070851)	39	8	0.79	+	10.11	0.00186
growth factor binding (GO:0019838)	35	7	0.71	+	9.85	0.00884
transmembrane receptor protein	54	9	1.1	+	8.21	0.00209

tyrosine kinase activity (GO:0004714)						
cytokine receptor binding (GO:0005126)	100	16	2.03	+	7.88	6.52E-07
cytokine activity (GO:0005125)	129	19	2.62	+	7.26	6.47E-08
receptor ligand activity (GO:0048018)	245	31	4.97	+	6.23	3.54E-12
signaling receptor activator activity (GO:0030546)	247	31	5.01	+	6.18	4.33E-12
protein tyrosine kinase activity (GO:0004713)	83	10	1.68	+	5.94	0.00825
receptor regulator activity (GO:0030545)	276	31	5.6	+	5.53	6.57E-11
G protein-coupled receptor binding (GO:0001664)	139	12	2.82	+	4.25	0.026
protein kinase activity (GO:0004672)	467	32	9.48	+	3.38	3.56E-06
signaling receptor binding (GO:0005102)	680	43	13.8	+	3.12	8.63E-08
protein serine/threonine kinase activity (GO:0004674)	349	22	7.08	+	3.11	0.003
phosphotransferase activity, alcohol group as acceptor (GO:0016773)	555	32	11.26	+	2.84	0.000151
kinase activity (GO:0016301)	613	32	12.44	+	2.57	0.00116
transmembrane signaling receptor activity (GO:0004888)	669	32	13.58	+	2.36	0.00669
molecular transducer activity (GO:0060089)	783	37	15.89	+	2.33	0.00218
signaling receptor activity (GO:0038023)	783	37	15.89	+	2.33	0.00218
transferase activity, transferring phosphorus-containing groups (GO:0016772)	714	32	14.49	+	2.21	0.0235
protein binding (GO:0005515)	2640	112	53.58	+	2.09	2.09E-11
catalytic activity, acting on a protein (GO:0140096)	1551	57	31.48	+	1.81	0.0118
binding (GO:0005488)	5963	166	121.03	+	1.37	0.00177
molecular_function (GO:0003674)	9803	242	198.96	+	1.22	0.016
Unclassified (UNCLASSIFIED)	10792	176	219.04	-	0.8	0
Cellular Component						
Bonferroni count:	438					

PANTHER GO-Slim Cellular Component	Homo sapiens - REFLIST (20595)	3_Otherthan_Core_Genes.txt (418)	3_Otherthan_Core_Genes.txt (expected)	3_Otherthan_Core_Genes.txt (over/under)	3_Otherthan_Core_Genes.txt (fold Enrichment)	3_Otherthan_Core_Genes.txt (P-value)
receptor complex (GO:0043235)	213	28	4.32	+	6.48	2.73E-11
transcription regulator complex (GO:0005667)	200	16	4.06	+	3.94	0.0032
cell surface (GO:0009986)	325	19	6.6	+	2.88	0.029
extracellular space (GO:0005615)	1051	58	21.33	+	2.72	6.72E-09
neuron projection (GO:0043005)	384	21	7.79	+	2.69	0.0301
extracellular region (GO:0005576)	1203	63	24.42	+	2.58	8.34E-09
intrinsic component of plasma membrane (GO:0031226)	798	34	16.2	+	2.1	0.0461
cellular anatomical entity (GO:0110165)	11122	271	225.73	+	1.2	0.00422
cellular_component (GO:0005575)	11293	272	229.2	+	1.19	0.0126
Unclassified (UNCLASSIFIED)	9302	146	188.8	-	0.77	0
Pathway						
Bonferroni count:	156					
PANTHER Pathways	Homo sapiens - REFLIST (20595)	3_Otherthan_Core_Genes.txt (418)	3_Otherthan_Core_Genes.txt (expected)	3_Otherthan_Core_Genes.txt (over/under)	3_Otherthan_Core_Genes.txt (fold Enrichment)	3_Otherthan_Core_Genes.txt (P-value)
Axon guidance mediated by Slit/Robo (P00008)	25	7	0.51	+	13.8	0.0004
TGF-beta signaling pathway (P00052)	102	18	2.07	+	8.69	4.38E-09
Blood coagulation (P00011)	48	8	0.97	+	8.21	0.0022
Axon guidance mediated by netrin (P00009)	36	6	0.73	+	8.21	0.0267
Ionotropic glutamate receptor pathway (P00037)	50	7	1.01	+	6.9	0.0205
Gonadotropin-releasing hormone receptor pathway (P06664)	231	25	4.69	+	5.33	1.03E-08
Inflammation mediated by chemokine and cytokine signaling pathway (P00031)	255	25	5.18	+	4.83	6.94E-08
FGF signaling pathway (P00021)	123	12	2.5	+	4.81	0.00261
CCKR signaling map (P06959)	172	16	3.49	+	4.58	0.000188
Integrin signalling pathway (P00034)	193	15	3.92	+	3.83	0.00301
Unclassified (UNCLASSIFIED)	17977	255	364.86	-	0.7	0
