# Supplementary material for: A protein interactions map of multiple organ systems associated with COVID-19 disease
Source: Genomics Inform. 2021 Jun 30;19(2):e14. doi: 10.5808/gi.20078 (PMC8261268; doi:10.5808/gi.20078)
Supplement: Supplementary Table. 3. — List of significant proteins and the drug candidates. [file gi-20078-suppl3.pdf]

Target	Organ_System	Degree	Core	Drug	Matador_Score	Interaction
ABCA1	Multi-Organ	2	Yes	reserpine	169	DIRECT
ABCA1	Multi-Organ	2	Yes	verapamil	169	DIRECT
ABCC8	Placenta	2	No	amlexanox	364	DIRECT
ABCC8	Placenta	2	No	diazoxide	950	DIRECT
ABCC8	Placenta	2	No	glibenclamide	950	DIRECT
ABCC8	Placenta	2	No	gliclazide	950	DIRECT
ABCC8	Placenta	2	No	glipizide	950	DIRECT_INDIRECT
ABCC8	Placenta	2	No	pinacidil	950	DIRECT
ABCC8	Placenta	2	No	repaglinide	950	DIRECT
ABCC8	Placenta	2	No	tolbutamide	950	DIRECT
ABCG5	Liver	1	No	19-nortestosterone	240	INDIRECT
ABCG5	Liver	1	No	acarbose	240	INDIRECT
ABCG5	Liver	1	No	bezafibrate	240	INDIRECT
ABCG5	Liver	1	No	ciprofibrate	240	INDIRECT
ABCG5	Liver	1	No	clofibrate	240	DIRECT
ABCG5	Liver	1	No	desogestrel	240	INDIRECT
ABCG5	Liver	1	No	doxazosin	240	INDIRECT
ABCG5	Liver	1	No	estrogen	240	INDIRECT
ABCG5	Liver	1	No	fluvastatin	240	INDIRECT
ABCG5	Liver	1	No	gemfibrozil	240	INDIRECT
ABCG5	Liver	1	No	glibenclamide	240	INDIRECT
ABCG5	Liver	1	No	lovastatin	240	INDIRECT
ABCG5	Liver	1	No	pioglitazone	240	INDIRECT
ABCG5	Liver	1	No	probucol	240	DIRECT
ABCG5	Liver	1	No	Ronicol	240	INDIRECT
ABCG5	Liver	1	No	rosiglitazone	240	INDIRECT
ABCG5	Liver	1	No	rosuvastatin	240	INDIRECT
ABCG5	Liver	1	No	streptozotocin	240	INDIRECT
ABCG5	Liver	1	No	tibolone	240	INDIRECT
ABCG5	Liver	1	No	Triton WR-1339	240	INDIRECT
ABCG8	Liver	1	No	reserpine	169	DIRECT
ABCG8	Liver	1	No	verapamil	169	DIRECT
ABL1	Multi-Organ	1	Yes	cytosine arabinoside	950	INDIRECT
ABL1	Multi-Organ	1	Yes	imatinib	950	DIRECT
ACE2	Heart	1	No	benazepril	891	DIRECT
ACE2	Heart	1	No	captopril	891	DIRECT
ACE2	Heart	1	No	diisopropylfluorophosphate	154	DIRECT_INDIRECT
ACE2	Heart	1	No	losartan	950	INDIRECT
ACVRL1	Multi-Organ	5	No	imatinib	159	DIRECT
ACVRL1	Multi-Organ	5	No	rapamycin	159	INDIRECT
ACVRL1	Multi-Organ	5	No	tamoxifen	159	INDIRECT
ADAMTS13	Liver	2	No	captopril	86	DIRECT
ADAMTS13	Liver	2	No	losartan	86	DIRECT_INDIRECT
ADAMTS13	Liver	2	No	ticlopidine	950	INDIRECT
ADCYAP1	Multi-Organ	1	No	riluzole	306	INDIRECT
ADRA2A	Fat	1	No	alprenolol	585	DIRECT
ADRA2A	Fat	1	No	amphetamine	908	DIRECT
ADRA2A	Fat	1	No	atropine	585	DIRECT
ADRA2A	Fat	1	No	brimonidine	908	DIRECT
ADRA2A	Fat	1	No	carvedilol	585	DIRECT
ADRA2A	Fat	1	No	chlorpromazine	908	DIRECT
ADRA2A	Fat	1	No	clonidine	908	DIRECT
ADRA2A	Fat	1	No	clozapine	694	DIRECT
ADRA2A	Fat	1	No	cocaine	908	INDIRECT
ADRA2A	Fat	1	No	desipramine	908	DIRECT

ADRA2A	Fat	1	No	dihydroergotamine	908	DIRECT
ADRA2A	Fat	1	No	droperidol	694	DIRECT
ADRA2A	Fat	1	No	ephedrine	694	DIRECT
ADRA2A	Fat	1	No	epinastine	694	DIRECT
ADRA2A	Fat	1	No	ergometrine	908	DIRECT
ADRA2A	Fat	1	No	ergotamine	694	DIRECT
ADRA2A	Fat	1	No	guanfacine	908	DIRECT
ADRA2A	Fat	1	No	haloperidol	908	DIRECT
ADRA2A	Fat	1	No	ibopamine	585	DIRECT
ADRA2A	Fat	1	No	imipramine	585	INDIRECT
ADRA2A	Fat	1	No	labetalol	694	DIRECT
ADRA2A	Fat	1	No	lofexidine	908	DIRECT
ADRA2A	Fat	1	No	metaraminol	694	DIRECT
ADRA2A	Fat	1	No	midodrine	694	DIRECT
ADRA2A	Fat	1	No	mirtazapine	908	DIRECT
ADRA2A	Fat	1	No	moxonidine	908	DIRECT
ADRA2A	Fat	1	No	naphazoline	908	DIRECT
ADRA2A	Fat	1	No	nicergoline	694	DIRECT
ADRA2A	Fat	1	No	nifedipine	585	DIRECT
ADRA2A	Fat	1	No	olanzapine	585	DIRECT
ADRA2A	Fat	1	No	oxymetazoline	908	DIRECT
ADRA2A	Fat	1	No	phenoxybenzamine	908	DIRECT
ADRA2A	Fat	1	No	phentolamine	908	DIRECT
ADRA2A	Fat	1	No	phenylephrine	694	DIRECT
ADRA2A	Fat	1	No	pindolol	585	DIRECT
ADRA2A	Fat	1	No	prazosin	908	DIRECT
ADRA2A	Fat	1	No	rilmenidine	908	DIRECT
ADRA2A	Fat	1	No	risperidone	908	DIRECT
ADRA2A	Fat	1	No	terbutaline	694	INDIRECT
ADRA2A	Fat	1	No	thioridazine	694	DIRECT
ADRA2A	Fat	1	No	tizanidine	908	DIRECT
ADRA2A	Fat	1	No	tolazoline	694	DIRECT
ADRA2A	Fat	1	No	tramazoline	908	DIRECT
ADRA2A	Fat	1	No	trazodone	694	DIRECT
ADRA2A	Fat	1	No	urapidil	908	DIRECT_IRRELEVANT
ADRA2A	Fat	1	No	verapamil	694	DIRECT
ADRA2A	Fat	1	No	yohimbine	908	DIRECT
ADRB1	Multi-Organ	1	Yes	acebutolol	809	DIRECT
ADRB1	Multi-Organ	1	Yes	alprenolol	809	DIRECT
ADRB1	Multi-Organ	1	Yes	amiodarone	809	INDIRECT
ADRB1	Multi-Organ	1	Yes	atenolol	950	DIRECT
ADRB1	Multi-Organ	1	Yes	atropine	585	DIRECT
ADRB1	Multi-Organ	1	Yes	betaxolol	950	DIRECT
ADRB1	Multi-Organ	1	Yes	bisoprolol	950	DIRECT
ADRB1	Multi-Organ	1	Yes	carteolol	809	DIRECT
ADRB1	Multi-Organ	1	Yes	carvedilol	950	DIRECT
ADRB1	Multi-Organ	1	Yes	clenbuterol	950	DIRECT
ADRB1	Multi-Organ	1	Yes	cocaine	585	INDIRECT
ADRB1	Multi-Organ	1	Yes	desipramine	809	INDIRECT
ADRB1	Multi-Organ	1	Yes	dobutamine	950	DIRECT
ADRB1	Multi-Organ	1	Yes	ephedrine	809	DIRECT
ADRB1	Multi-Organ	1	Yes	fenoterol	809	DIRECT
ADRB1	Multi-Organ	1	Yes	ibopamine	809	DIRECT
ADRB1	Multi-Organ	1	Yes	imipramine	809	INDIRECT
ADRB1	Multi-Organ	1	Yes	isoproterenol	950	DIRECT
ADRB1	Multi-Organ	1	Yes	labetalol	809	DIRECT
ADRB1	Multi-Organ	1	Yes	lofexidine	585	DIRECT

ADRB1	Multi-Organ	1	Yes	metipranolol	809	DIRECT
ADRB1	Multi-Organ	1	Yes	metoprolol	950	DIRECT
ADRB1	Multi-Organ	1	Yes	mianserin	809	DIRECT_INDIRECT
ADRB1	Multi-Organ	1	Yes	nadolol	809	DIRECT
ADRB1	Multi-Organ	1	Yes	naphazoline	585	DIRECT
ADRB1	Multi-Organ	1	Yes	nebivolol	950	DIRECT
ADRB1	Multi-Organ	1	Yes	nifedipine	585	DIRECT
ADRB1	Multi-Organ	1	Yes	olanzapine	585	DIRECT
ADRB1	Multi-Organ	1	Yes	phentolamine	585	DIRECT
ADRB1	Multi-Organ	1	Yes	phenylephrine	809	DIRECT
ADRB1	Multi-Organ	1	Yes	pindolol	809	DIRECT
ADRB1	Multi-Organ	1	Yes	pirbuterol	809	DIRECT
ADRB1	Multi-Organ	1	Yes	practolol	950	DIRECT
ADRB1	Multi-Organ	1	Yes	prazosin	809	DIRECT
ADRB1	Multi-Organ	1	Yes	prenalterol	950	DIRECT
ADRB1	Multi-Organ	1	Yes	procaterol	809	DIRECT
ADRB1	Multi-Organ	1	Yes	propranolol	950	DIRECT
ADRB1	Multi-Organ	1	Yes	reserpine	809	INDIRECT
ADRB1	Multi-Organ	1	Yes	ritodrine	809	DIRECT
ADRB1	Multi-Organ	1	Yes	salbutamol	809	DIRECT
ADRB1	Multi-Organ	1	Yes	sotalol	809	DIRECT
ADRB1	Multi-Organ	1	Yes	streptozotocin	809	INDIRECT
ADRB1	Multi-Organ	1	Yes	talinolol	950	DIRECT
ADRB1	Multi-Organ	1	Yes	terbutaline	809	DIRECT
ADRB1	Multi-Organ	1	Yes	timolol	809	DIRECT
AGT	Multi-Organ	1	No	candesartan	950	DIRECT
AGT	Multi-Organ	1	No	eprosartan	950	DIRECT
AGT	Multi-Organ	1	No	irbesartan	950	DIRECT
AGT	Multi-Organ	1	No	losartan	737	DIRECT
AGT	Multi-Organ	1	No	losartan	950	DIRECT
AGT	Multi-Organ	1	No	streptozotocin	950	INDIRECT
AGT	Multi-Organ	1	No	telmisartan	950	DIRECT
AGT	Multi-Organ	1	No	valsartan	950	DIRECT
ALK	Bowel	1	No	imatinib	94	DIRECT
ALOX5	Multi-Organ	1	No	1,8-dihydroxy-9-anthrone	866	DIRECT
ALOX5	Multi-Organ	1	No	5-aminosalicylic acid	950	DIRECT
ALOX5	Multi-Organ	1	No	auranofin	950	INDIRECT
ALOX5	Multi-Organ	1	No	azelastine	866	INDIRECT
ALOX5	Multi-Organ	1	No	benoxaprofen	866	DIRECT
ALOX5	Multi-Organ	1	No	dibutyryl cyclic AMP	866	INDIRECT
ALOX5	Multi-Organ	1	No	dithranol	866	DIRECT
ALOX5	Multi-Organ	1	No	gamma-linolenic acid	866	DIRECT
ALOX5	Multi-Organ	1	No	Hae	866	DIRECT
ALOX5	Multi-Organ	1	No	hydroxyurea	866	DIRECT
ALOX5	Multi-Organ	1	No	ibuprofen	950	DIRECT
ALOX5	Multi-Organ	1	No	iloprost	866	INDIRECT
ALOX5	Multi-Organ	1	No	indomethacin	610	DIRECT
ALOX5	Multi-Organ	1	No	ketoprofen	610	DIRECT
ALOX5	Multi-Organ	1	No	ketotifen	610	DIRECT
ALOX5	Multi-Organ	1	No	meclofenamate	866	INDIRECT
ALOX5	Multi-Organ	1	No	mizolastine	950	DIRECT
ALOX5	Multi-Organ	1	No	montelukast	950	DIRECT
ALOX5	Multi-Organ	1	No	naproxen	610	DIRECT
ALOX5	Multi-Organ	1	No	nimesulide	610	INDIRECT

ALOX5	Multi-Organ	1	No	nordihydroguaiaretic acid	950	DIRECT
ALOX5	Multi-Organ	1	No	phenylbutazone	610	DIRECT
ALOX5	Multi-Organ	1	No	phenylephrine	610	INDIRECT
ALOX5	Multi-Organ	1	No	salicylate	610	DIRECT
ALOX5	Multi-Organ	1	No	silymarin	950	DIRECT_INDIRECT
ALOX5	Multi-Organ	1	No	sulfasalazine	950	DIRECT
ALOX5	Multi-Organ	1	No	tenidap	950	DIRECT
ALOX5	Multi-Organ	1	No	Tris buffer	866	DIRECT
ALOX5	Multi-Organ	1	No	verapamil	866	INDIRECT
AMOT	Multi-Organ	1	Yes	famotidine	765	DIRECT
AMOT	Multi-Organ	1	Yes	famotidine	950	DIRECT
AMOT	Multi-Organ	1	Yes	famotidine	950	INDIRECT
AMOT	Multi-Organ	1	Yes	lamotrigine	233	DIRECT
AMOT	Multi-Organ	1	Yes	lamotrigine	318	DIRECT
AMOT	Multi-Organ	1	Yes	xamoterol	511	INDIRECT
ANGPT2	Fat	1	No	thalidomide	463	INDIRECT
APOE	Multi-Organ	1	No	19-nortestosterone	240	INDIRECT
APOE	Multi-Organ	1	No	acarbose	240	INDIRECT
APOE	Multi-Organ	1	No	acipimox	325	INDIRECT
APOE	Multi-Organ	1	No	atorvastatin	950	INDIRECT
APOE	Multi-Organ	1	No	bezafibrate	950	INDIRECT
APOE	Multi-Organ	1	No	ciprofibrate	325	INDIRECT
APOE	Multi-Organ	1	No	clofibrate	325	INDIRECT
APOE	Multi-Organ	1	No	desogestrel	240	INDIRECT
APOE	Multi-Organ	1	No	doxazosin	240	INDIRECT
APOE	Multi-Organ	1	No	estrogen	950	INDIRECT
APOE	Multi-Organ	1	No	fenofibrate	950	INDIRECT
APOE	Multi-Organ	1	No	fluvastatin	950	INDIRECT
APOE	Multi-Organ	1	No	gemfibrozil	950	INDIRECT
APOE	Multi-Organ	1	No	glibenclamide	240	INDIRECT
APOE	Multi-Organ	1	No	lovastatin	325	INDIRECT
APOE	Multi-Organ	1	No	pantethine	325	INDIRECT
APOE	Multi-Organ	1	No	pioglitazone	240	INDIRECT
APOE	Multi-Organ	1	No	probucol	950	INDIRECT
APOE	Multi-Organ	1	No	Ronicol	240	INDIRECT
APOE	Multi-Organ	1	No	rosiglitazone	240	INDIRECT
APOE	Multi-Organ	1	No	rosuvastatin	325	INDIRECT
APOE	Multi-Organ	1	No	simvastatin	950	INDIRECT
APOE	Multi-Organ	1	No	streptozotocin	240	INDIRECT
APOE	Multi-Organ	1	No	tibolone	325	INDIRECT
APOE	Multi-Organ	1	No	Triton WR-1339	240	INDIRECT
AR	Multi-Organ	12	Yes	5-methylpyrazinecarboxylic acid 4-oxide	950	INDIRECT
AR	Multi-Organ	12	Yes	acarbose	240	INDIRECT
AR	Multi-Organ	12	Yes	acarbose	778	DIRECT
AR	Multi-Organ	12	Yes	acarbose	950	DIRECT
AR	Multi-Organ	12	Yes	acarbose	950	INDIRECT
AR	Multi-Organ	12	Yes	acenocoumarol	197	INDIRECT
AR	Multi-Organ	12	Yes	acenocoumarol	950	DIRECT
AR	Multi-Organ	12	Yes	acenocoumarol	950	INDIRECT
AR	Multi-Organ	12	Yes	acetylcarnitine	213	DIRECT
AR	Multi-Organ	12	Yes	acetylcarnitine	950	DIRECT
AR	Multi-Organ	12	Yes	acetylcarnitine	950	INDIRECT
AR	Multi-Organ	12	Yes	aclarubicin	620	DIRECT
AR	Multi-Organ	12	Yes	aclarubicin	920	DIRECT
AR	Multi-Organ	12	Yes	aclarubicin	950	DIRECT

AR	Multi-Organ	12	Yes	adapalene	950	DIRECT
AR	Multi-Organ	12	Yes	amiodarone	692	DIRECT
AR	Multi-Organ	12	Yes	amiodarone	809	INDIRECT
AR	Multi-Organ	12	Yes	amiodarone	914	DIRECT
AR	Multi-Organ	12	Yes	amiodarone	950	DIRECT
AR	Multi-Organ	12	Yes	amiodarone	950	INDIRECT
AR	Multi-Organ	12	Yes	argatroban	671	INDIRECT
AR	Multi-Organ	12	Yes	argatroban	705	INDIRECT
AR	Multi-Organ	12	Yes	argatroban	950	DIRECT
AR	Multi-Organ	12	Yes	argatroban	950	INDIRECT
AR	Multi-Organ	12	Yes	aripiprazole	800	DIRECT
AR	Multi-Organ	12	Yes	aripiprazole	950	DIRECT
AR	Multi-Organ	12	Yes	benzbromarone	950	DIRECT_INDIRECT
AR	Multi-Organ	12	Yes	bezafibrate	950	DIRECT
AR	Multi-Organ	12	Yes	candesartan	950	DIRECT
AR	Multi-Organ	12	Yes	candesartan	950	INDIRECT
AR	Multi-Organ	12	Yes	carbachol	233	INDIRECT
AR	Multi-Organ	12	Yes	carbachol	374	INDIRECT
AR	Multi-Organ	12	Yes	carbachol	407	INDIRECT
AR	Multi-Organ	12	Yes	carbachol	511	DIRECT_INDIRECT
AR	Multi-Organ	12	Yes	carbachol	556	DIRECT
AR	Multi-Organ	12	Yes	carbachol	619	INDIRECT
AR	Multi-Organ	12	Yes	carbachol	753	INDIRECT
AR	Multi-Organ	12	Yes	carbachol	903	DIRECT
AR	Multi-Organ	12	Yes	carbachol	907	INDIRECT
AR	Multi-Organ	12	Yes	carbachol	950	DIRECT_INDIRECT
AR	Multi-Organ	12	Yes	carbachol	950	INDIRECT
AR	Multi-Organ	12	Yes	carbamazepine	318	DIRECT
AR	Multi-Organ	12	Yes	carbamazepine	950	DIRECT
AR	Multi-Organ	12	Yes	carbamazepine	950	DIRECT_INDIRECT
AR	Multi-Organ	12	Yes	carbamazepine	950	INDIRECT
AR	Multi-Organ	12	Yes	carbenoxolone	950	DIRECT_IRRELEVANT
AR	Multi-Organ	12	Yes	carbetocin	950	DIRECT
AR	Multi-Organ	12	Yes	carbetocin	950	DIRECT_IRRELEVANT
AR	Multi-Organ	12	Yes	carboquone	950	DIRECT
AR	Multi-Organ	12	Yes	Cardiovasc	233	DIRECT
AR	Multi-Organ	12	Yes	Cardiovasc	609	DIRECT
AR	Multi-Organ	12	Yes	carteolol	163	DIRECT
AR	Multi-Organ	12	Yes	carteolol	809	DIRECT
AR	Multi-Organ	12	Yes	carteolol	950	DIRECT
AR	Multi-Organ	12	Yes	carvedilol	585	DIRECT
AR	Multi-Organ	12	Yes	carvedilol	809	DIRECT
AR	Multi-Organ	12	Yes	carvedilol	950	DIRECT
AR	Multi-Organ	12	Yes	carvedilol	950	INDIRECT
AR	Multi-Organ	12	Yes	cinnarizine	233	DIRECT
AR	Multi-Organ	12	Yes	cinnarizine	696	DIRECT
AR	Multi-Organ	12	Yes	cinnarizine	800	DIRECT
AR	Multi-Organ	12	Yes	ciprofibrate	950	DIRECT
AR	Multi-Organ	12	Yes	clofibrate	80	INDIRECT
AR	Multi-Organ	12	Yes	clofibrate	950	DIRECT
AR	Multi-Organ	12	Yes	cocaine	950	INDIRECT
AR	Multi-Organ	12	Yes	cytosine arabinoside	276	DIRECT
AR	Multi-Organ	12	Yes	cytosine arabinoside	950	DIRECT

AR	Multi-Organ	12	Yes	cytosine arabinoside	950	INDIRECT
AR	Multi-Organ	12	Yes	d-tubocurarine	389	DIRECT
AR	Multi-Organ	12	Yes	d-tubocurarine	556	DIRECT
AR	Multi-Organ	12	Yes	d-tubocurarine	906	DIRECT
AR	Multi-Organ	12	Yes	d-tubocurarine	950	DIRECT
AR	Multi-Organ	12	Yes	d-tubocurarine	950	INDIRECT
AR	Multi-Organ	12	Yes	dacarbazine	950	DIRECT
AR	Multi-Organ	12	Yes	DCF	672	DIRECT
AR	Multi-Organ	12	Yes	diisopropylfluorophosphate	154	DIRECT_INDIRECT
AR	Multi-Organ	12	Yes	eprosartan	950	DIRECT
AR	Multi-Organ	12	Yes	eprosartan	950	INDIRECT
AR	Multi-Organ	12	Yes	fasudil	582	DIRECT
AR	Multi-Organ	12	Yes	fenofibrate	950	DIRECT
AR	Multi-Organ	12	Yes	ferrous fumarate	845	INDIRECT
AR	Multi-Organ	12	Yes	ferrous fumarate	950	INDIRECT
AR	Multi-Organ	12	Yes	fludarabine	766	DIRECT
AR	Multi-Organ	12	Yes	fludarabine	784	DIRECT
AR	Multi-Organ	12	Yes	fludarabine	920	INDIRECT
AR	Multi-Organ	12	Yes	fludarabine	950	DIRECT
AR	Multi-Organ	12	Yes	fludarabine	950	INDIRECT
AR	Multi-Organ	12	Yes	flunarizine	609	DIRECT
AR	Multi-Organ	12	Yes	flunarizine	828	DIRECT
AR	Multi-Organ	12	Yes	flunarizine	924	DIRECT
AR	Multi-Organ	12	Yes	fondaparinux sodium	950	DIRECT
AR	Multi-Organ	12	Yes	fondaparinux sodium	950	INDIRECT
AR	Multi-Organ	12	Yes	fumarate	950	DIRECT
AR	Multi-Organ	12	Yes	gemfibrozil	950	DIRECT
AR	Multi-Organ	12	Yes	hexobarbital	163	DIRECT
AR	Multi-Organ	12	Yes	idarubicin	914	DIRECT
AR	Multi-Organ	12	Yes	idarubicin	920	DIRECT
AR	Multi-Organ	12	Yes	Implanon	263	DIRECT
AR	Multi-Organ	12	Yes	irbesartan	177	INDIRECT
AR	Multi-Organ	12	Yes	irbesartan	950	DIRECT
AR	Multi-Organ	12	Yes	irbesartan	950	INDIRECT
AR	Multi-Organ	12	Yes	isocarboxazid	749	DIRECT
AR	Multi-Organ	12	Yes	isocarboxazid	950	DIRECT
AR	Multi-Organ	12	Yes	losartan	86	DIRECT_INDIRECT
AR	Multi-Organ	12	Yes	losartan	228	INDIRECT
AR	Multi-Organ	12	Yes	losartan	308	INDIRECT
AR	Multi-Organ	12	Yes	losartan	737	DIRECT
AR	Multi-Organ	12	Yes	losartan	826	INDIRECT
AR	Multi-Organ	12	Yes	losartan	950	DIRECT
AR	Multi-Organ	12	Yes	losartan	950	INDIRECT
AR	Multi-Organ	12	Yes	lovastatin	499	INDIRECT
AR	Multi-Organ	12	Yes	metaraminol	694	DIRECT
AR	Multi-Organ	12	Yes	mifepristone	263	DIRECT
AR	Multi-Organ	12	Yes	morphine	950	INDIRECT
AR	Multi-Organ	12	Yes	naratriptan	950	DIRECT
AR	Multi-Organ	12	Yes	nordihydroguaiaretic acid	171	DIRECT
AR	Multi-Organ	12	Yes	nordihydroguaiaretic acid	610	DIRECT
AR	Multi-Organ	12	Yes	nordihydroguaiaretic acid	635	DIRECT
AR	Multi-Organ	12	Yes	nordihydroguaiaretic acid	753	INDIRECT

AR	Multi-Organ	12	Yes	nordihydroguaiaretic acid	866	DIRECT
AR	Multi-Organ	12	Yes	nordihydroguaiaretic acid	898	DIRECT
AR	Multi-Organ	12	Yes	nordihydroguaiaretic acid	950	DIRECT
AR	Multi-Organ	12	Yes	nordihydroguaiaretic acid	950	INDIRECT
AR	Multi-Organ	12	Yes	oxcarbazepine	950	DIRECT
AR	Multi-Organ	12	Yes	paraoxon	138	DIRECT_INDIRECT
AR	Multi-Organ	12	Yes	paraoxon	163	DIRECT
AR	Multi-Organ	12	Yes	paraoxon	749	DIRECT
AR	Multi-Organ	12	Yes	paraoxon	903	INDIRECT
AR	Multi-Organ	12	Yes	paraoxon	906	DIRECT
AR	Multi-Organ	12	Yes	paraoxon	922	DIRECT
AR	Multi-Organ	12	Yes	paraoxon	950	DIRECT
AR	Multi-Organ	12	Yes	pargyline	950	DIRECT
AR	Multi-Organ	12	Yes	pargyline	950	INDIRECT
AR	Multi-Organ	12	Yes	paroxetine	950	DIRECT
AR	Multi-Organ	12	Yes	pemetrexed	950	DIRECT
AR	Multi-Organ	12	Yes	pentobarbital	489	DIRECT
AR	Multi-Organ	12	Yes	pentobarbital	556	DIRECT
AR	Multi-Organ	12	Yes	pentobarbital	950	INDIRECT
AR	Multi-Organ	12	Yes	phenobarbital	138	INDIRECT
AR	Multi-Organ	12	Yes	phenobarbital	372	INDIRECT
AR	Multi-Organ	12	Yes	phenobarbital	414	DIRECT
AR	Multi-Organ	12	Yes	phenobarbital	489	DIRECT
AR	Multi-Organ	12	Yes	phenobarbital	582	DIRECT_INDIRECT
AR	Multi-Organ	12	Yes	phenobarbital	749	INDIRECT
AR	Multi-Organ	12	Yes	phenobarbital	772	INDIRECT
AR	Multi-Organ	12	Yes	phenobarbital	903	INDIRECT
AR	Multi-Organ	12	Yes	phenobarbital	918	INDIRECT
AR	Multi-Organ	12	Yes	phenobarbital	950	DIRECT
AR	Multi-Organ	12	Yes	phenobarbital	950	DIRECT_INDIRECT
AR	Multi-Organ	12	Yes	phenobarbital	950	INDIRECT
AR	Multi-Organ	12	Yes	pilocarpine	903	DIRECT
AR	Multi-Organ	12	Yes	pilocarpine	950	INDIRECT
AR	Multi-Organ	12	Yes	pioglitazone	950	DIRECT
AR	Multi-Organ	12	Yes	pirarubicin	920	INDIRECT
AR	Multi-Organ	12	Yes	pirarubicin	950	DIRECT
AR	Multi-Organ	12	Yes	probucol	283	INDIRECT
AR	Multi-Organ	12	Yes	probucol	892	INDIRECT
AR	Multi-Organ	12	Yes	proglumide	906	DIRECT
AR	Multi-Organ	12	Yes	raloxifene	263	DIRECT
AR	Multi-Organ	12	Yes	rosiglitazone	499	INDIRECT
AR	Multi-Organ	12	Yes	rosiglitazone	950	DIRECT
AR	Multi-Organ	12	Yes	rosiglitazone	950	INDIRECT
AR	Multi-Organ	12	Yes	secobarbital	489	DIRECT
AR	Multi-Organ	12	Yes	silymarin	228	INDIRECT
AR	Multi-Organ	12	Yes	silymarin	320	INDIRECT
AR	Multi-Organ	12	Yes	silymarin	366	INDIRECT
AR	Multi-Organ	12	Yes	silymarin	655	INDIRECT
AR	Multi-Organ	12	Yes	silymarin	772	INDIRECT
AR	Multi-Organ	12	Yes	silymarin	906	INDIRECT
AR	Multi-Organ	12	Yes	silymarin	923	INDIRECT
AR	Multi-Organ	12	Yes	silymarin	950	DIRECT_INDIRECT

AR	Multi-Organ	12	Yes	silymarin	950	INDIRECT
AR	Multi-Organ	12	Yes	sparteine	950	DIRECT
AR	Multi-Organ	12	Yes	spironolactone	263	DIRECT
AR	Multi-Organ	12	Yes	sulindac	950	DIRECT_INDIRECT
AR	Multi-Organ	12	Yes	tamoxifen	263	DIRECT
AR	Multi-Organ	12	Yes	tazarotene	511	DIRECT
AR	Multi-Organ	12	Yes	tazarotene	950	DIRECT
AR	Multi-Organ	12	Yes	tazarotene	950	INDIRECT
AR	Multi-Organ	12	Yes	telmisartan	950	DIRECT
AR	Multi-Organ	12	Yes	troglitazone	499	INDIRECT
AR	Multi-Organ	12	Yes	troglitazone	950	DIRECT
AR	Multi-Organ	12	Yes	troglitazone	950	INDIRECT
AR	Multi-Organ	12	Yes	valsartan	950	DIRECT
AR	Multi-Organ	12	Yes	valsartan	950	INDIRECT
AR	Multi-Organ	12	Yes	warfarin	98	INDIRECT
AR	Multi-Organ	12	Yes	warfarin	197	INDIRECT
AR	Multi-Organ	12	Yes	warfarin	713	DIRECT
AR	Multi-Organ	12	Yes	warfarin	950	DIRECT
AR	Multi-Organ	12	Yes	warfarin	950	INDIRECT
ARPIN	Marrow	1	No	pilocarpine	903	DIRECT
ARPIN	Marrow	1	No	pilocarpine	950	INDIRECT
ARRB2	Multi-Organ	4	Yes	morphine	950	INDIRECT
BAX	Placenta	2	No	5-fluorouracil	950	INDIRECT
BAX	Placenta	2	No	bortezomib	950	INDIRECT
BAX	Placenta	2	No	etoposide	950	INDIRECT
BAX	Placenta	2	No	fludarabine	950	INDIRECT
BAX	Placenta	2	No	Ras	950	INDIRECT
BCL2L1	Bowel	1	No	bortezomib	950	INDIRECT
BCL2L1	Bowel	1	No	etoposide	950	INDIRECT
BCL2L1	Bowel	1	No	imatinib	950	INDIRECT
BDNF	Heart	2	Yes	imatinib	160	DIRECT
BDNF	Heart	2	Yes	rapamycin	159	INDIRECT
BDNF	Heart	2	Yes	Ras	950	INDIRECT
BDNF	Heart	2	Yes	riluzole	950	INDIRECT
BDNF	Heart	2	Yes	tamoxifen	159	INDIRECT
BMPR1A	Multi-Organ	2	No	imatinib	159	DIRECT
BMPR1A	Multi-Organ	2	No	rapamycin	159	INDIRECT
BMPR1A	Multi-Organ	2	No	tamoxifen	159	INDIRECT
BMPR1B	Kidney	1	No	imatinib	159	DIRECT
BMPR1B	Kidney	1	No	rapamycin	159	INDIRECT
BMPR1B	Kidney	1	No	tamoxifen	159	INDIRECT
BRCA2	Marrow	1	No	mitomycin C	950	INDIRECT
BTK	Multi-Organ	1	Yes	imatinib	94	DIRECT
C3	Multi-Organ	2	No	2-chlorodeoxyadenosine	496	DIRECT
C3	Multi-Organ	2	No	acetylcarnitine	213	DIRECT
C3	Multi-Organ	2	No	amiloride	197	DIRECT
C3	Multi-Organ	2	No	bortezomib	214	DIRECT
C3	Multi-Organ	2	No	captopril	86	DIRECT
C3	Multi-Organ	2	No	cimetidine	182	DIRECT
C3	Multi-Organ	2	No	cimetidine	812	DIRECT
C3	Multi-Organ	2	No	clofibrate	80	INDIRECT
C3	Multi-Organ	2	No	desferrioxamine	135	DIRECT
C3	Multi-Organ	2	No	diazoxide	112	DIRECT
C3	Multi-Organ	2	No	etoposide	950	DIRECT
C3	Multi-Organ	2	No	fenofibrate	207	INDIRECT
C3	Multi-Organ	2	No	glucuronic acid	950	DIRECT

C3	Multi-Organ	2	No	gold sodium thiomalate	207	INDIRECT
C3	Multi-Organ	2	No	ibuprofen	207	INDIRECT
C3	Multi-Organ	2	No	isoproterenol	329	INDIRECT
C3	Multi-Organ	2	No	losartan	86	DIRECT_INDIRECT
C3	Multi-Organ	2	No	melphalan	196	DIRECT
C3	Multi-Organ	2	No	melphalan	616	DIRECT
C3	Multi-Organ	2	No	mepyramine	812	INDIRECT
C3	Multi-Organ	2	No	methotrexate	950	DIRECT
C3	Multi-Organ	2	No	methylprednisolone	207	INDIRECT
C3	Multi-Organ	2	No	MGB	812	DIRECT
C3	Multi-Organ	2	No	minoxidil	112	DIRECT_INDIRECT
C3	Multi-Organ	2	No	ouabain	560	INDIRECT
C3	Multi-Organ	2	No	pentoxifylline	496	DIRECT
C3	Multi-Organ	2	No	piroxicam	207	INDIRECT
C3	Multi-Organ	2	No	probenecid	613	DIRECT
C3	Multi-Organ	2	No	repaglinide	112	DIRECT
C3	Multi-Organ	2	No	reserpine	169	DIRECT
C3	Multi-Organ	2	No	rosiglitazone	207	INDIRECT
C3	Multi-Organ	2	No	spironolactone	97	INDIRECT
C3	Multi-Organ	2	No	sulfinpyrazone	182	DIRECT
C3	Multi-Organ	2	No	sulfobromophthalein	613	DIRECT
C3	Multi-Organ	2	No	teniposide	950	DIRECT_INDIRECT
C3	Multi-Organ	2	No	valproic acid	412	DIRECT
C3	Multi-Organ	2	No	verapamil	112	DIRECT
C3	Multi-Organ	2	No	verapamil	613	DIRECT
C8A	Liver	1	No	amiloride	197	DIRECT
C8A	Liver	1	No	ouabain	560	INDIRECT
C9	Liver	1	No	8-methoxypsoralen	163	DIRECT
C9	Liver	1	No	acenocoumarol	950	DIRECT
C9	Liver	1	No	alprazolam	163	DIRECT
C9	Liver	1	No	amiloride	591	DIRECT
C9	Liver	1	No	amiloride	950	DIRECT
C9	Liver	1	No	aminoglutethimide	163	DIRECT
C9	Liver	1	No	aminopyrine	163	DIRECT
C9	Liver	1	No	aprepitant	950	DIRECT
C9	Liver	1	No	caffeine	163	DIRECT
C9	Liver	1	No	carteolol	163	DIRECT
C9	Liver	1	No	celecoxib	950	DIRECT
C9	Liver	1	No	chloral hydrate	163	DIRECT
C9	Liver	1	No	clofibrate	80	INDIRECT
C9	Liver	1	No	clofibrate	163	INDIRECT
C9	Liver	1	No	desferrioxamine	135	DIRECT
C9	Liver	1	No	diclofenac	950	DIRECT
C9	Liver	1	No	diosmetin	163	DIRECT
C9	Liver	1	No	ET-743	950	DIRECT
C9	Liver	1	No	ethylmorphine	163	DIRECT
C9	Liver	1	No	felodipine	163	DIRECT
C9	Liver	1	No	fluoxetine	950	DIRECT
C9	Liver	1	No	flurbiprofen	950	DIRECT
C9	Liver	1	No	fluvastatin	950	DIRECT
C9	Liver	1	No	fluvoxamine	950	DIRECT
C9	Liver	1	No	gemfibrozil	950	DIRECT
C9	Liver	1	No	glimepiride	950	DIRECT
C9	Liver	1	No	halothane	163	DIRECT

C9	Liver	1	No	hexobarbital	163	DIRECT
C9	Liver	1	No	ibuprofen	950	DIRECT
C9	Liver	1	No	lansoprazole	950	DIRECT
C9	Liver	1	No	lornoxicam	950	DIRECT
C9	Liver	1	No	losartan	950	DIRECT
C9	Liver	1	No	metyrapone	163	DIRECT
C9	Liver	1	No	mifepristone	163	DIRECT
C9	Liver	1	No	nifedipine	950	DIRECT_INDIRECT
C9	Liver	1	No	pantoprazole	950	DIRECT
C9	Liver	1	No	paraoxon	163	DIRECT
C9	Liver	1	No	phenobarbital	950	INDIRECT
C9	Liver	1	No	phenprocoumon	950	DIRECT
C9	Liver	1	No	phenytoin	950	DIRECT
C9	Liver	1	No	propoxyphene	163	DIRECT
C9	Liver	1	No	reserpine	169	DIRECT
C9	Liver	1	No	rosiglitazone	950	DIRECT_IRRELEVANT
C9	Liver	1	No	tienilic acid	163	DIRECT
C9	Liver	1	No	tolbutamide	950	DIRECT
C9	Liver	1	No	torasemide	950	DIRECT
C9	Liver	1	No	toremifene	163	DIRECT
C9	Liver	1	No	tPA	950	DIRECT
C9	Liver	1	No	trimipramine	163	DIRECT
C9	Liver	1	No	valproic acid	412	DIRECT
C9	Liver	1	No	verapamil	169	DIRECT
C9	Liver	1	No	warfarin	950	DIRECT
CACNA1C	Multi-Organ	1	No	amiloride	233	DIRECT
CACNA1C	Multi-Organ	1	No	amlodipine	609	DIRECT
CACNA1C	Multi-Organ	1	No	benidipine	609	DIRECT
CACNA1C	Multi-Organ	1	No	bepridil	233	DIRECT
CACNA1C	Multi-Organ	1	No	caffeine	233	INDIRECT
CACNA1C	Multi-Organ	1	No	carbachol	233	INDIRECT
CACNA1C	Multi-Organ	1	No	Cardiovasc	609	DIRECT
CACNA1C	Multi-Organ	1	No	cilnidipine	609	DIRECT
CACNA1C	Multi-Organ	1	No	cinnarizine	233	DIRECT
CACNA1C	Multi-Organ	1	No	D-600	609	DIRECT
CACNA1C	Multi-Organ	1	No	dantrolene	233	DIRECT
CACNA1C	Multi-Organ	1	No	diltiazem	609	DIRECT
CACNA1C	Multi-Organ	1	No	felodipine	609	DIRECT
CACNA1C	Multi-Organ	1	No	flunarizine	609	DIRECT
CACNA1C	Multi-Organ	1	No	gabapentin	609	DIRECT
CACNA1C	Multi-Organ	1	No	halothane	233	DIRECT
CACNA1C	Multi-Organ	1	No	isoproterenol	609	INDIRECT
CACNA1C	Multi-Organ	1	No	isradipine	609	DIRECT
CACNA1C	Multi-Organ	1	No	lacidipine	609	DIRECT
CACNA1C	Multi-Organ	1	No	lamotrigine	233	DIRECT
CACNA1C	Multi-Organ	1	No	loperamide	233	DIRECT
CACNA1C	Multi-Organ	1	No	manidipine	233	DIRECT
CACNA1C	Multi-Organ	1	No	mibefradil	609	DIRECT
CACNA1C	Multi-Organ	1	No	nifedipine	609	DIRECT
CACNA1C	Multi-Organ	1	No	nilvadipine	609	DIRECT
CACNA1C	Multi-Organ	1	No	nimodipine	609	DIRECT
CACNA1C	Multi-Organ	1	No	nisoldipine	609	DIRECT
CACNA1C	Multi-Organ	1	No	nitrendipine	609	DIRECT
CACNA1C	Multi-Organ	1	No	phenytoin	233	DIRECT
CACNA1C	Multi-Organ	1	No	pimozide	233	DIRECT
CACNA1C	Multi-Organ	1	No	pinaverium bromide	609	DIRECT

CACNA1C	Multi-Organ	1	No	procaine	233	DIRECT_IRRELEVANT
CACNA1C	Multi-Organ	1	No	verapamil	609	DIRECT
CACNA1D	Multi-Organ	1	No	amiloride	233	DIRECT
CACNA1D	Multi-Organ	1	No	amlodipine	609	DIRECT
CACNA1D	Multi-Organ	1	No	benidipine	609	DIRECT
CACNA1D	Multi-Organ	1	No	bepridil	233	DIRECT
CACNA1D	Multi-Organ	1	No	caffeine	233	INDIRECT
CACNA1D	Multi-Organ	1	No	carbachol	233	INDIRECT
CACNA1D	Multi-Organ	1	No	Cardiovasc	609	DIRECT
CACNA1D	Multi-Organ	1	No	cilnidipine	609	DIRECT
CACNA1D	Multi-Organ	1	No	cinnarizine	233	DIRECT
CACNA1D	Multi-Organ	1	No	D-600	609	DIRECT
CACNA1D	Multi-Organ	1	No	dantrolene	233	DIRECT
CACNA1D	Multi-Organ	1	No	diltiazem	609	DIRECT
CACNA1D	Multi-Organ	1	No	felodipine	609	DIRECT
CACNA1D	Multi-Organ	1	No	flunarizine	609	DIRECT
CACNA1D	Multi-Organ	1	No	gabapentin	609	DIRECT
CACNA1D	Multi-Organ	1	No	halothane	233	DIRECT
CACNA1D	Multi-Organ	1	No	isoproterenol	609	INDIRECT
CACNA1D	Multi-Organ	1	No	isradipine	609	DIRECT
CACNA1D	Multi-Organ	1	No	lacidipine	609	DIRECT
CACNA1D	Multi-Organ	1	No	lamotrigine	233	DIRECT
CACNA1D	Multi-Organ	1	No	loperamide	233	DIRECT
CACNA1D	Multi-Organ	1	No	manidipine	233	DIRECT
CACNA1D	Multi-Organ	1	No	mibefradil	609	DIRECT
CACNA1D	Multi-Organ	1	No	nifedipine	609	DIRECT
CACNA1D	Multi-Organ	1	No	nilvadipine	609	DIRECT
CACNA1D	Multi-Organ	1	No	nimodipine	609	DIRECT
CACNA1D	Multi-Organ	1	No	nisoldipine	609	DIRECT
CACNA1D	Multi-Organ	1	No	nitrendipine	609	DIRECT
CACNA1D	Multi-Organ	1	No	phenytoin	233	DIRECT
CACNA1D	Multi-Organ	1	No	pimozide	233	DIRECT
CACNA1D	Multi-Organ	1	No	pinaverium bromide	609	DIRECT
CACNA1D	Multi-Organ	1	No	procaine	233	DIRECT_IRRELEVANT
CACNA1D	Multi-Organ	1	No	verapamil	609	DIRECT
CASP6	Bowel	1	No	etoposide	950	INDIRECT
CASP7	Heart	3	No	etoposide	950	INDIRECT
CCL11	Multi-Organ	1	No	budesonide	950	INDIRECT
CCL11	Multi-Organ	1	No	fluticasone propionate	950	INDIRECT
CCL11	Multi-Organ	1	No	irbesartan	177	INDIRECT
CCL11	Multi-Organ	1	No	montelukast	950	INDIRECT
CCL16	Liver	1	No	fluticasone propionate	177	INDIRECT
CCL16	Liver	1	No	irbesartan	177	INDIRECT
CCL2	Marrow	2	No	atorvastatin	950	INDIRECT
CCL2	Marrow	2	No	cerivastatin	950	INDIRECT
CCL2	Marrow	2	No	fenofibrate	950	INDIRECT
CCL2	Marrow	2	No	fluticasone propionate	177	INDIRECT
CCL2	Marrow	2	No	irbesartan	177	INDIRECT
CCL2	Marrow	2	No	irbesartan	950	INDIRECT
CCL2	Marrow	2	No	pioglitazone	950	INDIRECT
CCL2	Marrow	2	No	pravastatin	950	INDIRECT
CCL2	Marrow	2	No	troglitazone	950	INDIRECT
CCL2	Marrow	2	No	valsartan	950	INDIRECT
CCL4	Bowel	2	No	fluticasone	177	INDIRECT

				propionate		
CCL4	Bowel	2	No	irbesartan	177	INDIRECT
CCL5	Heart	2	No	budesonide	950	INDIRECT
CCL5	Heart	2	No	fluticasone propionate	950	INDIRECT
CCL5	Heart	2	No	irbesartan	177	INDIRECT
CCL8	Multi-Organ	2	No	fluticasone propionate	177	INDIRECT
CCL8	Multi-Organ	2	No	irbesartan	177	INDIRECT
CCNA2	Marrow	5	Yes	bortezomib	320	INDIRECT
CCNA2	Marrow	5	Yes	rapamycin	903	INDIRECT
CCNA2	Marrow	5	Yes	silymarin	320	INDIRECT
CCNA2	Marrow	5	Yes	troglitazone	320	INDIRECT
CCNB1	Multi-Organ	1	Yes	bortezomib	320	INDIRECT
CCNB1	Multi-Organ	1	Yes	rapamycin	320	INDIRECT
CCNB1	Multi-Organ	1	Yes	silymarin	950	INDIRECT
CCNB1	Multi-Organ	1	Yes	troglitazone	320	INDIRECT
CCNC	Heart	2	No	bortezomib	320	INDIRECT
CCNC	Heart	2	No	rapamycin	320	INDIRECT
CCNC	Heart	2	No	silymarin	320	INDIRECT
CCNC	Heart	2	No	troglitazone	320	INDIRECT
CCND1	Multi-Organ	7	Yes	bortezomib	320	INDIRECT
CCND1	Multi-Organ	7	Yes	fulvestrant	950	INDIRECT
CCND1	Multi-Organ	7	Yes	rapamycin	950	INDIRECT
CCND1	Multi-Organ	7	Yes	silymarin	950	INDIRECT
CCND1	Multi-Organ	7	Yes	sulindac	950	INDIRECT
CCND1	Multi-Organ	7	Yes	tamoxifen	950	INDIRECT
CCND1	Multi-Organ	7	Yes	troglitazone	950	INDIRECT
CCNF	Marrow	1	Yes	bortezomib	320	INDIRECT
CCNF	Marrow	1	Yes	rapamycin	320	INDIRECT
CCNF	Marrow	1	Yes	silymarin	320	INDIRECT
CCNF	Marrow	1	Yes	troglitazone	320	INDIRECT
CD28	Placenta	1	No	rapamycin	950	INDIRECT
CD4	Multi-Organ	1	Yes	caffeine	134	DIRECT
CD4	Multi-Organ	1	Yes	carbachol	407	INDIRECT
CD4	Multi-Organ	1	Yes	diisopropylfluorophosphate	171	INDIRECT
CD4	Multi-Organ	1	Yes	fenofibrate	950	INDIRECT
CD4	Multi-Organ	1	Yes	glycerophosphocholine	171	INDIRECT
CD4	Multi-Organ	1	Yes	milrinone	134	DIRECT
CD4	Multi-Organ	1	Yes	nordihydroguaiaretic acid	171	DIRECT
CD4	Multi-Organ	1	Yes	papaverine	134	DIRECT
CD4	Multi-Organ	1	Yes	pentoxifylline	134	DIRECT
CD4	Multi-Organ	1	Yes	reserpine	169	DIRECT
CD4	Multi-Organ	1	Yes	verapamil	169	DIRECT
CDH1	Multi-Organ	7	Yes	procainamide	514	DIRECT_INDIRECT
CDKN1A	Multi-Organ	5	Yes	etoposide	950	INDIRECT
CDKN1B	Marrow	5	Yes	rapamycin	950	INDIRECT
CDKN1B	Marrow	5	Yes	silymarin	950	INDIRECT
CEACAM5	Multi-Organ	1	No	5-fluorouracil	950	INDIRECT
CEACAM5	Multi-Organ	1	No	tegafur	950	INDIRECT
CFH	Marrow	1	No	19-nortestosterone	240	INDIRECT
CFH	Marrow	1	No	acarbose	240	INDIRECT
CFH	Marrow	1	No	acipimox	325	INDIRECT
CFH	Marrow	1	No	bezafibrate	325	INDIRECT
CFH	Marrow	1	No	ciprofibrate	325	INDIRECT

CFH	Marrow	1	No	clofibrate	325	INDIRECT
CFH	Marrow	1	No	desogestrel	240	INDIRECT
CFH	Marrow	1	No	doxazosin	240	INDIRECT
CFH	Marrow	1	No	estrogen	240	INDIRECT
CFH	Marrow	1	No	fluvastatin	325	INDIRECT
CFH	Marrow	1	No	gemfibrozil	325	INDIRECT
CFH	Marrow	1	No	glibenclamide	240	INDIRECT
CFH	Marrow	1	No	lovastatin	325	INDIRECT
CFH	Marrow	1	No	pantethine	325	INDIRECT
CFH	Marrow	1	No	pioglitazone	240	INDIRECT
CFH	Marrow	1	No	probucol	325	INDIRECT
CFH	Marrow	1	No	Ronicol	240	INDIRECT
CFH	Marrow	1	No	rosiglitazone	240	INDIRECT
CFH	Marrow	1	No	rosuvastatin	325	INDIRECT
CFH	Marrow	1	No	streptozotocin	240	INDIRECT
CFH	Marrow	1	No	tibolone	325	INDIRECT
CFH	Marrow	1	No	Triton WR-1339	240	INDIRECT
CFTR	Liver	2	No	bumetanide	950	INDIRECT
CFTR	Liver	2	No	glibenclamide	950	DIRECT
CFTR	Liver	2	No	isoproterenol	329	INDIRECT
CNR1	Multi-Organ	1	Yes	delta 9-tetrahydrocannabinol	950	DIRECT
CNR1	Multi-Organ	1	Yes	morphine	950	INDIRECT
CNR1	Multi-Organ	1	Yes	nabilone	900	DIRECT
COL1A1	Multi-Organ	1	Yes	colchicine	228	INDIRECT
COL1A1	Multi-Organ	1	Yes	losartan	228	INDIRECT
COL1A1	Multi-Organ	1	Yes	silymarin	228	INDIRECT
COL1A1	Multi-Organ	1	Yes	spironolactone	228	INDIRECT
COL3A1	Multi-Organ	2	No	colchicine	228	INDIRECT
COL3A1	Multi-Organ	2	No	losartan	228	INDIRECT
COL3A1	Multi-Organ	2	No	silymarin	228	INDIRECT
COL3A1	Multi-Organ	2	No	spironolactone	228	INDIRECT
CSF2RB	Placenta	1	Yes	imatinib	159	DIRECT
CSF2RB	Placenta	1	Yes	rapamycin	159	INDIRECT
CSF2RB	Placenta	1	Yes	tamoxifen	159	INDIRECT
CTNNB1	Fat	13	Yes	sulindac	950	INDIRECT
CXCL1	Multi-Organ	2	No	fluticasone propionate	177	INDIRECT
CXCL1	Multi-Organ	2	No	irbesartan	177	INDIRECT
CXCL5	Multi-Organ	1	No	fluticasone propionate	177	INDIRECT
CXCL5	Multi-Organ	1	No	irbesartan	177	INDIRECT
DDR1	Multi-Organ	1	No	imatinib	160	DIRECT
DLAT	Heart	1	No	acetylcarnitine	213	DIRECT
DLAT	Heart	1	No	clofibrate	80	INDIRECT
DPP4	Multi-Organ	2	No	diisopropylfluorophosphate	771	DIRECT
DSP	Multi-Organ	4	No	spironolactone	97	INDIRECT
EGF	Heart	1	Yes	bortezomib	950	INDIRECT
EGF	Heart	1	Yes	celecoxib	950	INDIRECT
EGF	Heart	1	Yes	docetaxel	950	INDIRECT
EGF	Heart	1	Yes	gefitinib	950	DIRECT
EGF	Heart	1	Yes	gefitinib	950	INDIRECT
EGF	Heart	1	Yes	gemcitabine	950	INDIRECT
EGF	Heart	1	Yes	imatinib	160	DIRECT
EGF	Heart	1	Yes	rapamycin	159	INDIRECT
EGF	Heart	1	Yes	rapamycin	950	INDIRECT
EGF	Heart	1	Yes	tamoxifen	159	INDIRECT

EGF	Heart	1	Yes	thalidomide	463	INDIRECT
EGF	Heart	1	Yes	thalidomide	950	INDIRECT
EGFR	Multi-Organ	11	Yes	docetaxel	950	INDIRECT
EGFR	Multi-Organ	11	Yes	gefitinib	950	DIRECT
EGFR	Multi-Organ	11	Yes	gemcitabine	950	INDIRECT
EGFR	Multi-Organ	11	Yes	imatinib	160	DIRECT
EGFR	Multi-Organ	11	Yes	rapamycin	159	INDIRECT
EGFR	Multi-Organ	11	Yes	tamoxifen	159	INDIRECT
EGLN1	Multi-Organ	1	Yes	D-penicillamine	702	INDIRECT
EIF4A1	Jejunum	4	Yes	rapamycin	621	INDIRECT
EIF4G1	Fat	2	Yes	rapamycin	950	INDIRECT
ELN	Multi-Organ	1	No	D-penicillamine	950	INDIRECT
ELN	Multi-Organ	1	No	spironolactone	97	INDIRECT
ENG	Marrow	3	No	rose bengal	708	DIRECT
EPHA2	Marrow	3	Yes	imatinib	160	DIRECT
EPHA3	Multi-Organ	1	Yes	imatinib	160	DIRECT
EPHA4	Bowel	3	Yes	imatinib	160	DIRECT
ERBB3	Heart	4	Yes	gefitinib	950	INDIRECT
ERBB3	Heart	4	Yes	imatinib	160	DIRECT
ERBB3	Heart	4	Yes	rapamycin	159	INDIRECT
ERBB3	Heart	4	Yes	tamoxifen	159	INDIRECT
ESR1	Liver	6	Yes	clomiphene citrate	950	DIRECT
ESR1	Liver	6	Yes	danazol	950	INDIRECT
ESR1	Liver	6	Yes	diethylstilbestrol	950	DIRECT
ESR1	Liver	6	Yes	estrogen	950	DIRECT
ESR1	Liver	6	Yes	flutamide	950	INDIRECT
ESR1	Liver	6	Yes	fulvestrant	950	DIRECT
ESR1	Liver	6	Yes	Implanon	263	DIRECT
ESR1	Liver	6	Yes	mifepristone	950	DIRECT_IRRELEVANT
ESR1	Liver	6	Yes	moxestrol	950	DIRECT
ESR1	Liver	6	Yes	octyl methoxycinnamate	950	DIRECT_IRRELEVANT
ESR1	Liver	6	Yes	phenol red	950	DIRECT
ESR1	Liver	6	Yes	raloxifene	950	DIRECT
ESR1	Liver	6	Yes	spironolactone	263	DIRECT
ESR1	Liver	6	Yes	tamoxifen	950	DIRECT
ESR1	Liver	6	Yes	tibolone	950	INDIRECT
ESR1	Liver	6	Yes	toremifene	950	DIRECT
ESRRA	Heart	1	Yes	diethylstilbestrol	950	DIRECT
ESRRA	Heart	1	Yes	Implanon	263	DIRECT
ESRRA	Heart	1	Yes	mifepristone	263	DIRECT
ESRRA	Heart	1	Yes	raloxifene	573	DIRECT
ESRRA	Heart	1	Yes	spironolactone	263	DIRECT
ESRRA	Heart	1	Yes	tamoxifen	573	DIRECT
F13A1	Multi-Organ	1	No	clofibrate	80	INDIRECT
F2	Liver	2	Yes	25-hydroxyvitamin D	274	DIRECT
F2	Liver	2	Yes	4-methylumbelliferone	187	DIRECT
F2	Liver	2	Yes	8-methoxypsoralen	163	DIRECT
F2	Liver	2	Yes	ACA	98	DIRECT
F2	Liver	2	Yes	alprazolam	163	DIRECT
F2	Liver	2	Yes	aminoglutethimide	163	DIRECT
F2	Liver	2	Yes	aminopyrine	163	DIRECT
F2	Liver	2	Yes	bicalutamide	867	INDIRECT
F2	Liver	2	Yes	budesonide	950	INDIRECT
F2	Liver	2	Yes	caffeine	163	DIRECT
F2	Liver	2	Yes	camostat mesilate	98	DIRECT

F2	Liver	2	Yes	captopril	427	INDIRECT
F2	Liver	2	Yes	carteolol	163	DIRECT
F2	Liver	2	Yes	chloral hydrate	163	DIRECT
F2	Liver	2	Yes	clenbuterol	467	INDIRECT
F2	Liver	2	Yes	clofibrate	163	INDIRECT
F2	Liver	2	Yes	desferrioxamine	135	DIRECT
F2	Liver	2	Yes	diclofenac	163	DIRECT
F2	Liver	2	Yes	diisopropylfluorophosphate	427	DIRECT
F2	Liver	2	Yes	diosmetin	163	DIRECT
F2	Liver	2	Yes	docetaxel	867	INDIRECT
F2	Liver	2	Yes	estramustine	867	INDIRECT
F2	Liver	2	Yes	ethylmorphine	163	DIRECT
F2	Liver	2	Yes	felodipine	163	DIRECT
F2	Liver	2	Yes	fenofibrate	950	INDIRECT
F2	Liver	2	Yes	finasteride	867	INDIRECT
F2	Liver	2	Yes	fluticasone propionate	950	INDIRECT
F2	Liver	2	Yes	halothane	163	DIRECT
F2	Liver	2	Yes	hexobarbital	163	DIRECT
F2	Liver	2	Yes	imatinib	159	DIRECT
F2	Liver	2	Yes	Implanon	263	DIRECT
F2	Liver	2	Yes	lansoprazole	163	DIRECT
F2	Liver	2	Yes	lovastatin	499	INDIRECT
F2	Liver	2	Yes	metyrapone	163	DIRECT
F2	Liver	2	Yes	mifepristone	163	DIRECT
F2	Liver	2	Yes	mifepristone	263	DIRECT
F2	Liver	2	Yes	nifedipine	163	DIRECT
F2	Liver	2	Yes	nilutamide	867	INDIRECT
F2	Liver	2	Yes	paraoxon	163	DIRECT
F2	Liver	2	Yes	probucol	283	INDIRECT
F2	Liver	2	Yes	propoxyphene	163	DIRECT
F2	Liver	2	Yes	raloxifene	263	DIRECT
F2	Liver	2	Yes	rapamycin	159	INDIRECT
F2	Liver	2	Yes	rapamycin	169	INDIRECT
F2	Liver	2	Yes	rapamycin	950	INDIRECT
F2	Liver	2	Yes	reserpine	169	DIRECT
F2	Liver	2	Yes	rosiglitazone	499	INDIRECT
F2	Liver	2	Yes	spironolactone	263	DIRECT
F2	Liver	2	Yes	streptozotocin	950	INDIRECT
F2	Liver	2	Yes	tamoxifen	159	INDIRECT
F2	Liver	2	Yes	tamoxifen	263	DIRECT
F2	Liver	2	Yes	tienilic acid	163	DIRECT
F2	Liver	2	Yes	tolbutamide	163	DIRECT
F2	Liver	2	Yes	toremifene	163	DIRECT
F2	Liver	2	Yes	trimipramine	163	DIRECT
F2	Liver	2	Yes	troglitazone	499	INDIRECT
F2	Liver	2	Yes	valproic acid	412	DIRECT
F2	Liver	2	Yes	verapamil	169	DIRECT
F2	Liver	2	Yes	warfarin	98	INDIRECT
F3	Multi-Organ	1	Yes	8-methoxypsoralen	163	DIRECT
F3	Multi-Organ	1	Yes	alprazolam	163	DIRECT
F3	Multi-Organ	1	Yes	aminoglutethimide	163	DIRECT
F3	Multi-Organ	1	Yes	aminopyrine	163	DIRECT
F3	Multi-Organ	1	Yes	caffeine	163	DIRECT
F3	Multi-Organ	1	Yes	carteolol	163	DIRECT
F3	Multi-Organ	1	Yes	chloral hydrate	163	DIRECT
F3	Multi-Organ	1	Yes	clofibrate	163	INDIRECT

F3	Multi-Organ	1	Yes	desferrioxamine	135	DIRECT
F3	Multi-Organ	1	Yes	diclofenac	163	DIRECT
F3	Multi-Organ	1	Yes	diosmetin	163	DIRECT
F3	Multi-Organ	1	Yes	ebastine	950	DIRECT
F3	Multi-Organ	1	Yes	ethylmorphine	163	DIRECT
F3	Multi-Organ	1	Yes	etoposide	604	INDIRECT
F3	Multi-Organ	1	Yes	felodipine	163	DIRECT
F3	Multi-Organ	1	Yes	halothane	163	DIRECT
F3	Multi-Organ	1	Yes	hexobarbital	163	DIRECT
F3	Multi-Organ	1	Yes	imatinib	159	DIRECT
F3	Multi-Organ	1	Yes	lansoprazole	163	DIRECT
F3	Multi-Organ	1	Yes	metyrapone	163	DIRECT
F3	Multi-Organ	1	Yes	mifepristone	163	DIRECT
F3	Multi-Organ	1	Yes	nifedipine	163	DIRECT
F3	Multi-Organ	1	Yes	paraoxon	163	DIRECT
F3	Multi-Organ	1	Yes	propoxyphene	163	DIRECT
F3	Multi-Organ	1	Yes	propylthiouracil	184	INDIRECT
F3	Multi-Organ	1	Yes	rapamycin	159	INDIRECT
F3	Multi-Organ	1	Yes	rapamycin	169	INDIRECT
F3	Multi-Organ	1	Yes	riluzole	306	INDIRECT
F3	Multi-Organ	1	Yes	tamoxifen	159	INDIRECT
F3	Multi-Organ	1	Yes	tienilic acid	163	DIRECT
F3	Multi-Organ	1	Yes	tolbutamide	163	DIRECT
F3	Multi-Organ	1	Yes	toremifene	163	DIRECT
F3	Multi-Organ	1	Yes	trimipramine	163	DIRECT
F7	Liver	1	Yes	ACA	98	DIRECT
F7	Liver	1	Yes	acenocoumarol	197	INDIRECT
F7	Liver	1	Yes	bezafibrate	197	INDIRECT
F7	Liver	1	Yes	camostat mesilate	98	DIRECT
F7	Liver	1	Yes	desogestrel	950	INDIRECT
F7	Liver	1	Yes	diisopropylfluorophosphate	950	DIRECT
F7	Liver	1	Yes	fluticasone propionate	163	DIRECT_INDIRECT
F7	Liver	1	Yes	fondaparinux sodium	950	INDIRECT
F7	Liver	1	Yes	imiquimod	163	INDIRECT
F7	Liver	1	Yes	Linomide	163	INDIRECT
F7	Liver	1	Yes	polyinosinic-polycytidylic acid	163	INDIRECT
F7	Liver	1	Yes	rapamycin	163	INDIRECT
F7	Liver	1	Yes	sulfasalazine	163	INDIRECT
F7	Liver	1	Yes	thalidomide	163	INDIRECT
F7	Liver	1	Yes	troglitazone	163	INDIRECT
F7	Liver	1	Yes	warfarin	950	INDIRECT
F8	Multi-Organ	3	No	8-methoxypsoralen	163	DIRECT
F8	Multi-Organ	3	No	acenocoumarol	197	INDIRECT
F8	Multi-Organ	3	No	alprazolam	163	DIRECT
F8	Multi-Organ	3	No	aminoglutethimide	163	DIRECT
F8	Multi-Organ	3	No	aminopyrine	163	DIRECT
F8	Multi-Organ	3	No	bezafibrate	197	INDIRECT
F8	Multi-Organ	3	No	caffeine	163	DIRECT
F8	Multi-Organ	3	No	carteolol	163	DIRECT
F8	Multi-Organ	3	No	chloral hydrate	163	DIRECT
F8	Multi-Organ	3	No	clofibrate	163	INDIRECT
F8	Multi-Organ	3	No	desferrioxamine	135	DIRECT
F8	Multi-Organ	3	No	desogestrel	197	INDIRECT
F8	Multi-Organ	3	No	diclofenac	163	DIRECT
F8	Multi-Organ	3	No	diosmetin	163	DIRECT

F8	Multi-Organ	3	No	ethylmorphine	163	DIRECT
F8	Multi-Organ	3	No	felodipine	163	DIRECT
F8	Multi-Organ	3	No	fluticasone propionate	163	DIRECT_INDIRECT
F8	Multi-Organ	3	No	fluticasone propionate	177	INDIRECT
F8	Multi-Organ	3	No	halothane	163	DIRECT
F8	Multi-Organ	3	No	hexobarbital	163	DIRECT
F8	Multi-Organ	3	No	imiquimod	163	INDIRECT
F8	Multi-Organ	3	No	irbesartan	177	INDIRECT
F8	Multi-Organ	3	No	lansoprazole	163	DIRECT
F8	Multi-Organ	3	No	Linomide	163	INDIRECT
F8	Multi-Organ	3	No	metyrapone	163	DIRECT
F8	Multi-Organ	3	No	mifepristone	163	DIRECT
F8	Multi-Organ	3	No	nifedipine	163	DIRECT
F8	Multi-Organ	3	No	paraoxon	163	DIRECT
F8	Multi-Organ	3	No	phenobarbital	372	INDIRECT
F8	Multi-Organ	3	No	polyinosinic-polycytidylic acid	163	INDIRECT
F8	Multi-Organ	3	No	propoxyphene	163	DIRECT
F8	Multi-Organ	3	No	rapamycin	163	INDIRECT
F8	Multi-Organ	3	No	sulfasalazine	163	INDIRECT
F8	Multi-Organ	3	No	thalidomide	163	INDIRECT
F8	Multi-Organ	3	No	tienilic acid	163	DIRECT
F8	Multi-Organ	3	No	tolbutamide	163	DIRECT
F8	Multi-Organ	3	No	toremifene	163	DIRECT
F8	Multi-Organ	3	No	trimipramine	163	DIRECT
F8	Multi-Organ	3	No	troglitazone	163	INDIRECT
F8	Multi-Organ	3	No	warfarin	197	INDIRECT
F9	Liver	1	No	ACA	98	DIRECT
F9	Liver	1	No	acenocoumarol	197	INDIRECT
F9	Liver	1	No	bezafibrate	197	INDIRECT
F9	Liver	1	No	camostat mesilate	98	DIRECT
F9	Liver	1	No	desogestrel	197	INDIRECT
F9	Liver	1	No	diisopropylfluorophosphate	98	DIRECT
F9	Liver	1	No	fluticasone propionate	163	DIRECT_INDIRECT
F9	Liver	1	No	imiquimod	163	INDIRECT
F9	Liver	1	No	Linomide	163	INDIRECT
F9	Liver	1	No	polyinosinic-polycytidylic acid	163	INDIRECT
F9	Liver	1	No	propylthiouracil	184	INDIRECT
F9	Liver	1	No	rapamycin	163	INDIRECT
F9	Liver	1	No	sulfasalazine	163	INDIRECT
F9	Liver	1	No	thalidomide	163	INDIRECT
F9	Liver	1	No	troglitazone	163	INDIRECT
F9	Liver	1	No	warfarin	197	INDIRECT
FAS	Multi-Organ	2	Yes	clofibrate	80	INDIRECT
FAS	Multi-Organ	2	Yes	etoposide	950	INDIRECT
FAS	Multi-Organ	2	Yes	fasudil	582	DIRECT
FAS	Multi-Organ	2	Yes	fasudil	619	INDIRECT
FAS	Multi-Organ	2	Yes	fasudil	909	DIRECT
FAS	Multi-Organ	2	Yes	fasudil	950	DIRECT
FAS	Multi-Organ	2	Yes	riluzole	306	INDIRECT
FAS	Multi-Organ	2	Yes	sulfasalazine	163	INDIRECT
FAS	Multi-Organ	2	Yes	sulfasalazine	610	DIRECT
FAS	Multi-Organ	2	Yes	sulfasalazine	691	DIRECT
FAS	Multi-Organ	2	Yes	sulfasalazine	866	DIRECT

FAS	Multi-Organ	2	Yes	sulfasalazine	950	DIRECT
FAS	Multi-Organ	2	Yes	sulfasalazine	950	INDIRECT
FBLN5	Marrow	1	No	spironolactone	97	INDIRECT
FBP1	Multi-Organ	2	Yes	metformin	950	INDIRECT
FBP1	Multi-Organ	2	Yes	mifepristone	950	INDIRECT
FBP1	Multi-Organ	2	Yes	streptozotocin	950	INDIRECT
FGFR2	Multi-Organ	5	No	imatinib	160	DIRECT
FGFR2	Multi-Organ	5	No	rapamycin	159	INDIRECT
FGFR2	Multi-Organ	5	No	tamoxifen	159	INDIRECT
FGFR3	Liver	4	No	imatinib	160	DIRECT
FGFR3	Liver	4	No	rapamycin	159	INDIRECT
FGFR3	Liver	4	No	tamoxifen	159	INDIRECT
FGFR4	Multi-Organ	2	No	imatinib	160	DIRECT
FGFR4	Multi-Organ	2	No	rapamycin	159	INDIRECT
FGFR4	Multi-Organ	2	No	tamoxifen	159	INDIRECT
FKBP1A	Multi-Organ	1	No	rapamycin	950	DIRECT
FKBP1A	Multi-Organ	1	No	tacrolimus	950	DIRECT
FLT1	Multi-Organ	1	No	imatinib	160	DIRECT
FLT1	Multi-Organ	1	No	rapamycin	159	INDIRECT
FLT1	Multi-Organ	1	No	tamoxifen	159	INDIRECT
FN1	Multi-Organ	3	Yes	spironolactone	97	INDIRECT
FOS	Multi-Organ	2	Yes	amphetamine	950	INDIRECT
FOS	Multi-Organ	2	Yes	capsaicin	950	INDIRECT
FOS	Multi-Organ	2	Yes	clozapine	950	INDIRECT
FOS	Multi-Organ	2	Yes	cocaine	950	INDIRECT
FOS	Multi-Organ	2	Yes	dibutyryl cyclic AMP	950	INDIRECT
FOS	Multi-Organ	2	Yes	fosinopril	950	DIRECT
FOS	Multi-Organ	2	Yes	fosinopril	950	INDIRECT
FOS	Multi-Organ	2	Yes	haloperidol	950	INDIRECT
FOS	Multi-Organ	2	Yes	ifosfamide	871	INDIRECT
FOS	Multi-Organ	2	Yes	ifosfamide	950	DIRECT
FOS	Multi-Organ	2	Yes	ifosfamide	950	DIRECT_INDIRECT
FOS	Multi-Organ	2	Yes	levodopa	950	INDIRECT
FOS	Multi-Organ	2	Yes	losartan	950	INDIRECT
FOS	Multi-Organ	2	Yes	methamphetamine	950	INDIRECT
FOS	Multi-Organ	2	Yes	miltefosine	753	DIRECT
FOS	Multi-Organ	2	Yes	morphine	950	INDIRECT
FOS	Multi-Organ	2	Yes	nicotine	950	INDIRECT
FOS	Multi-Organ	2	Yes	pentylenetetrazol	950	INDIRECT
FOSB	Multi-Organ	1	Yes	levodopa	950	INDIRECT
GC	Liver	1	Yes	3-morpholinosydnonimine	950	INDIRECT
GC	Liver	1	Yes	acetylcarnitine	213	DIRECT
GC	Liver	1	Yes	atorvastatin	950	DIRECT
GC	Liver	1	Yes	cerivastatin	950	DIRECT
GC	Liver	1	Yes	clofibrate	80	INDIRECT
GC	Liver	1	Yes	fluvastatin	950	DIRECT
GC	Liver	1	Yes	lovastatin	950	DIRECT
GC	Liver	1	Yes	pravastatin	950	DIRECT
GC	Liver	1	Yes	rosiglitazone	950	INDIRECT
GC	Liver	1	Yes	SCMC	950	INDIRECT
GC	Liver	1	Yes	simvastatin	950	DIRECT
GC	Liver	1	Yes	spironolactone	97	INDIRECT
GC	Liver	1	Yes	troglitazone	950	INDIRECT
GDNF	Multi-Organ	1	No	Ras	950	INDIRECT
GDNF	Multi-Organ	1	No	riluzole	950	INDIRECT

GFRA1	Multi-Organ	2	No	imatinib	159	DIRECT
GFRA1	Multi-Organ	2	No	rapamycin	159	INDIRECT
GFRA1	Multi-Organ	2	No	tamoxifen	159	INDIRECT
GLUL	Multi-Organ	1	Yes	dibutyryl cyclic AMP	875	INDIRECT
GRIA2	Liver	1	No	amphetamine	844	INDIRECT
GRIA2	Liver	1	No	cocaine	316	INDIRECT
GRIA2	Liver	1	No	cyclothiazide	950	DIRECT
GRIA2	Liver	1	No	diazoxide	844	DIRECT
GRIA2	Liver	1	No	felbamate	203	INDIRECT
GRIA2	Liver	1	No	haloperidol	316	INDIRECT
GRIA2	Liver	1	No	isoflurane	844	DIRECT_IRRELEVANT
GRIA2	Liver	1	No	nicotine	316	INDIRECT
GRIA2	Liver	1	No	reset	950	DIRECT
GRIA2	Liver	1	No	riluzole	316	DIRECT
GRIA3	Multi-Organ	1	No	amphetamine	844	INDIRECT
GRIA3	Multi-Organ	1	No	cocaine	316	INDIRECT
GRIA3	Multi-Organ	1	No	cyclothiazide	950	DIRECT_INDIRECT
GRIA3	Multi-Organ	1	No	diazoxide	844	DIRECT
GRIA3	Multi-Organ	1	No	felbamate	203	INDIRECT
GRIA3	Multi-Organ	1	No	haloperidol	316	INDIRECT
GRIA3	Multi-Organ	1	No	isoflurane	844	DIRECT_IRRELEVANT
GRIA3	Multi-Organ	1	No	nicotine	316	INDIRECT
GRIA3	Multi-Organ	1	No	reset	844	DIRECT
GRIA3	Multi-Organ	1	No	riluzole	316	DIRECT
GRIN2A	Multi-Organ	2	No	amantadine	624	DIRECT
GRIN2A	Multi-Organ	2	No	cocaine	316	INDIRECT
GRIN2A	Multi-Organ	2	No	cyclothiazide	203	DIRECT_INDIRECT
GRIN2A	Multi-Organ	2	No	dextromethorphan	624	DIRECT
GRIN2A	Multi-Organ	2	No	felbamate	203	INDIRECT
GRIN2A	Multi-Organ	2	No	haloperidol	624	DIRECT
GRIN2A	Multi-Organ	2	No	ifenprodil	624	DIRECT
GRIN2A	Multi-Organ	2	No	ketamine	624	DIRECT
GRIN2A	Multi-Organ	2	No	memantine	950	DIRECT
GRIN2A	Multi-Organ	2	No	morphine	624	INDIRECT
GRIN2A	Multi-Organ	2	No	nicotine	316	INDIRECT
GRIN2A	Multi-Organ	2	No	pentylenetetrazol	624	INDIRECT
GRIN2A	Multi-Organ	2	No	reset	316	DIRECT
GRIN2A	Multi-Organ	2	No	riluzole	624	INDIRECT
GRM2	Multi-Organ	1	Yes	amphetamine	645	INDIRECT
GRM2	Multi-Organ	1	Yes	cocaine	316	INDIRECT
GRM2	Multi-Organ	1	Yes	cyclothiazide	645	INDIRECT
GRM2	Multi-Organ	1	Yes	felbamate	203	INDIRECT
GRM2	Multi-Organ	1	Yes	haloperidol	316	INDIRECT
GRM2	Multi-Organ	1	Yes	nicotine	316	INDIRECT
GRM2	Multi-Organ	1	Yes	reset	316	DIRECT
GRM2	Multi-Organ	1	Yes	riluzole	316	DIRECT
HBA1	Multi-Organ	2	Yes	acarbose	950	INDIRECT
HBA1	Multi-Organ	2	Yes	bezafibrate	684	DIRECT
HBA1	Multi-Organ	2	Yes	desferrioxamine	684	INDIRECT
HBA1	Multi-Organ	2	Yes	metformin	950	INDIRECT
HBA1	Multi-Organ	2	Yes	pioglitazone	684	INDIRECT
HBA1	Multi-Organ	2	Yes	troglitazone	684	INDIRECT
HBB	Multi-Organ	1	Yes	bezafibrate	684	DIRECT
HBB	Multi-Organ	1	Yes	desferrioxamine	684	INDIRECT

HBB	Multi-Organ	1	Yes	pioglitazone	684	INDIRECT
HBB	Multi-Organ	1	Yes	troglitazone	684	INDIRECT
HCK	Multi-Organ	2	Yes	imatinib	94	DIRECT
HGF	Multi-Organ	2	Yes	ACA	98	DIRECT
HGF	Multi-Organ	2	Yes	camostat mesilate	98	DIRECT
HGF	Multi-Organ	2	Yes	diisopropylfluorophosphate	98	DIRECT
HGF	Multi-Organ	2	Yes	warfarin	98	INDIRECT
HGFAC	Liver	3	Yes	ACA	98	DIRECT
HGFAC	Liver	3	Yes	camostat mesilate	98	DIRECT
HGFAC	Liver	3	Yes	diisopropylfluorophosphate	98	DIRECT
HGFAC	Liver	3	Yes	warfarin	98	INDIRECT
HIF1A	Multi-Organ	6	Yes	desferrioxamine	950	INDIRECT
HIF1A	Multi-Organ	6	Yes	rapamycin	950	INDIRECT
HTR2A	Bowel	9	Yes	aripiprazole	950	DIRECT
HTR2A	Bowel	9	Yes	clonidine	381	DIRECT_IRRELEVANT
HTR2A	Bowel	9	Yes	clozapine	950	DIRECT
HTR2A	Bowel	9	Yes	cocaine	950	INDIRECT
HTR2A	Bowel	9	Yes	cyproheptadine	381	DIRECT
HTR2A	Bowel	9	Yes	fluoxetine	950	INDIRECT
HTR2A	Bowel	9	Yes	fluvoxamine	950	INDIRECT
HTR2A	Bowel	9	Yes	imipramine	950	INDIRECT
HTR2A	Bowel	9	Yes	ketanserin	950	DIRECT
HTR2A	Bowel	9	Yes	loxapine	950	INDIRECT
HTR2A	Bowel	9	Yes	metergoline	950	DIRECT
HTR2A	Bowel	9	Yes	methysergide	381	DIRECT
HTR2A	Bowel	9	Yes	metoclopramide	381	DIRECT
HTR2A	Bowel	9	Yes	mianserin	950	DIRECT
HTR2A	Bowel	9	Yes	mirtazapine	950	DIRECT
HTR2A	Bowel	9	Yes	mosapramine	950	DIRECT
HTR2A	Bowel	9	Yes	nefazodone	950	DIRECT
HTR2A	Bowel	9	Yes	nicotine	381	INDIRECT
HTR2A	Bowel	9	Yes	olanzapine	950	DIRECT
HTR2A	Bowel	9	Yes	phentolamine	235	DIRECT
HTR2A	Bowel	9	Yes	pindolol	381	DIRECT
HTR2A	Bowel	9	Yes	pizotifen	381	DIRECT
HTR2A	Bowel	9	Yes	propranolol	381	DIRECT_IRRELEVANT
HTR2A	Bowel	9	Yes	quetiapine	950	DIRECT
HTR2A	Bowel	9	Yes	risperidone	950	DIRECT
HTR2A	Bowel	9	Yes	sertindole	950	DIRECT
HTR2A	Bowel	9	Yes	sumatriptan	381	DIRECT
HTR2A	Bowel	9	Yes	trazodone	950	DIRECT
HTR2A	Bowel	9	Yes	ziprasidone	950	DIRECT
HTR2A	Bowel	9	Yes	zotepine	950	DIRECT
ICAM1	Multi-Organ	1	No	azelastine	950	INDIRECT
ICAM1	Multi-Organ	1	No	budesonide	950	INDIRECT
ICAM1	Multi-Organ	1	No	cerivastatin	950	INDIRECT
ICAM1	Multi-Organ	1	No	fluvastatin	950	INDIRECT
ICAM1	Multi-Organ	1	No	loratadine	950	INDIRECT
ICAM1	Multi-Organ	1	No	olopatadine	950	INDIRECT
ICAM1	Multi-Organ	1	No	pentoxifylline	950	INDIRECT
ICAM1	Multi-Organ	1	No	rosiglitazone	950	INDIRECT
ID2	Liver	2	Yes	beta-acetyldigoxin	950	INDIRECT
ID2	Liver	2	Yes	cocaine	316	INDIRECT
ID2	Liver	2	Yes	cyclothiazide	203	DIRECT_INDIRECT

ID2	Liver	2	Yes	felbamate	203	INDIRECT
ID2	Liver	2	Yes	haloperidol	316	INDIRECT
ID2	Liver	2	Yes	isoproterenol	950	INDIRECT
ID2	Liver	2	Yes	nicotine	316	INDIRECT
ID2	Liver	2	Yes	reset	316	DIRECT
ID2	Liver	2	Yes	riluzole	316	DIRECT
IFIT3	Multi-Organ	1	No	polyinosinic-polycytidylic acid	563	INDIRECT
IGF2	Multi-Organ	2	No	imatinib	159	DIRECT
IGF2	Multi-Organ	2	No	rapamycin	159	INDIRECT
IGF2	Multi-Organ	2	No	streptozotocin	950	INDIRECT
IGF2	Multi-Organ	2	No	tamoxifen	159	INDIRECT
IGFBP1	Multi-Organ	1	No	metformin	950	INDIRECT
IGFBP1	Multi-Organ	1	No	mifepristone	950	INDIRECT
IGFBP1	Multi-Organ	1	No	streptozotocin	950	INDIRECT
IGFBP3	Multi-Organ	1	No	mifepristone	576	INDIRECT
IL16	Multi-Organ	1	No	fluticasone propionate	163	DIRECT_INDIRECT
IL16	Multi-Organ	1	No	imiquimod	163	INDIRECT
IL16	Multi-Organ	1	No	Linomide	163	INDIRECT
IL16	Multi-Organ	1	No	polyinosinic-polycytidylic acid	163	INDIRECT
IL16	Multi-Organ	1	No	rapamycin	163	INDIRECT
IL16	Multi-Organ	1	No	sulfasalazine	163	INDIRECT
IL16	Multi-Organ	1	No	thalidomide	163	INDIRECT
IL16	Multi-Organ	1	No	troglitazone	163	INDIRECT
IL1B	Multi-Organ	2	No	fluticasone propionate	163	DIRECT_INDIRECT
IL1B	Multi-Organ	2	No	imiquimod	163	INDIRECT
IL1B	Multi-Organ	2	No	Linomide	163	INDIRECT
IL1B	Multi-Organ	2	No	polyinosinic-polycytidylic acid	163	INDIRECT
IL1B	Multi-Organ	2	No	rapamycin	163	INDIRECT
IL1B	Multi-Organ	2	No	sulfasalazine	163	INDIRECT
IL1B	Multi-Organ	2	No	thalidomide	163	INDIRECT
IL1B	Multi-Organ	2	No	troglitazone	163	INDIRECT
IL2RA	Heart	1	No	rapamycin	950	INDIRECT
IL2RA	Heart	1	No	tacrolimus	950	INDIRECT
IL32	Multi-Organ	1	No	fluticasone propionate	163	DIRECT_INDIRECT
IL32	Multi-Organ	1	No	imiquimod	163	INDIRECT
IL32	Multi-Organ	1	No	Linomide	163	INDIRECT
IL32	Multi-Organ	1	No	polyinosinic-polycytidylic acid	163	INDIRECT
IL32	Multi-Organ	1	No	rapamycin	163	INDIRECT
IL32	Multi-Organ	1	No	sulfasalazine	163	INDIRECT
IL32	Multi-Organ	1	No	thalidomide	163	INDIRECT
IL32	Multi-Organ	1	No	troglitazone	163	INDIRECT
IL6	Multi-Organ	4	Yes	atorvastatin	950	INDIRECT
IL6	Multi-Organ	4	Yes	bortezomib	950	INDIRECT
IL6	Multi-Organ	4	Yes	fluticasone propionate	163	DIRECT_INDIRECT
IL6	Multi-Organ	4	Yes	imatinib	159	DIRECT
IL6	Multi-Organ	4	Yes	imiquimod	950	INDIRECT
IL6	Multi-Organ	4	Yes	indomethacin	950	INDIRECT
IL6	Multi-Organ	4	Yes	Linomide	163	INDIRECT
IL6	Multi-Organ	4	Yes	methylprednisolone	950	INDIRECT
IL6	Multi-Organ	4	Yes	pentoxifylline	950	INDIRECT
IL6	Multi-Organ	4	Yes	polyinosinic-polycytidylic acid	950	INDIRECT

IL6	Multi-Organ	4	Yes	rapamycin	159	INDIRECT
IL6	Multi-Organ	4	Yes	rapamycin	163	INDIRECT
IL6	Multi-Organ	4	Yes	rapamycin	291	DIRECT
IL6	Multi-Organ	4	Yes	sulfasalazine	163	INDIRECT
IL6	Multi-Organ	4	Yes	tacrolimus	254	DIRECT
IL6	Multi-Organ	4	Yes	tamoxifen	159	INDIRECT
IL6	Multi-Organ	4	Yes	thalidomide	163	INDIRECT
IL6	Multi-Organ	4	Yes	troglitazone	163	INDIRECT
IL6ST	Multi-Organ	3	Yes	imatinib	159	DIRECT
IL6ST	Multi-Organ	3	Yes	rapamycin	159	INDIRECT
IL6ST	Multi-Organ	3	Yes	tamoxifen	159	INDIRECT
INSR	Multi-Organ	6	Yes	glibenclamide	911	INDIRECT
INSR	Multi-Organ	6	Yes	gold thioglucose	911	INDIRECT
INSR	Multi-Organ	6	Yes	gold thioglucose	950	INDIRECT
INSR	Multi-Organ	6	Yes	imatinib	160	DIRECT
INSR	Multi-Organ	6	Yes	metformin	911	DIRECT
INSR	Multi-Organ	6	Yes	metformin	950	DIRECT
INSR	Multi-Organ	6	Yes	pioglitazone	911	INDIRECT
INSR	Multi-Organ	6	Yes	pioglitazone	950	INDIRECT
INSR	Multi-Organ	6	Yes	rosiglitazone	911	INDIRECT
INSR	Multi-Organ	6	Yes	rosiglitazone	950	INDIRECT
IRS1	Jejunum	2	Yes	metformin	950	INDIRECT
IRS1	Jejunum	2	Yes	pioglitazone	950	INDIRECT
IRS1	Jejunum	2	Yes	rapamycin	950	INDIRECT
IRS1	Jejunum	2	Yes	rosiglitazone	950	INDIRECT
IRS1	Jejunum	2	Yes	troglitazone	950	INDIRECT
ITGA2	Multi-Organ	1	No	eptifibatide	950	DIRECT
ITGA2	Multi-Organ	1	No	ticlopidine	950	DIRECT_INDIRECT
ITGA2	Multi-Organ	1	No	tirofiban	950	DIRECT
ITK	Heart	1	No	imatinib	94	DIRECT
JAK3	Multi-Organ	1	No	imatinib	94	DIRECT
JUN	Multi-Organ	6	Yes	bortezomib	950	INDIRECT
JUN	Multi-Organ	6	Yes	mifepristone	950	INDIRECT
JUN	Multi-Organ	6	Yes	nordihydroguaiaretic acid	950	DIRECT
JUN	Multi-Organ	6	Yes	pioglitazone	950	INDIRECT
JUN	Multi-Organ	6	Yes	raloxifene	950	INDIRECT
JUN	Multi-Organ	6	Yes	rapamycin	950	INDIRECT
JUN	Multi-Organ	6	Yes	tamoxifen	950	INDIRECT
JUN	Multi-Organ	6	Yes	troglitazone	950	INDIRECT
KCNMA1	Multi-Organ	1	No	diazoxide	112	DIRECT
KCNMA1	Multi-Organ	1	No	minoxidil	112	DIRECT_INDIRECT
KCNMA1	Multi-Organ	1	No	repaglinide	112	DIRECT
KCNMA1	Multi-Organ	1	No	verapamil	112	DIRECT
KCNN2	Placenta	1	No	d-tubocurarine	950	INDIRECT
KCNN2	Placenta	1	No	diazoxide	112	DIRECT
KCNN2	Placenta	1	No	minoxidil	112	DIRECT_INDIRECT
KCNN2	Placenta	1	No	repaglinide	112	DIRECT
KCNN2	Placenta	1	No	verapamil	112	DIRECT
KCNQ1	Heart	1	Yes	diazoxide	112	DIRECT
KCNQ1	Heart	1	Yes	minoxidil	112	DIRECT_INDIRECT
KCNQ1	Heart	1	Yes	repaglinide	112	DIRECT
KCNQ1	Heart	1	Yes	verapamil	112	DIRECT
KDR	Multi-Organ	1	Yes	imatinib	160	DIRECT
KDR	Multi-Organ	1	Yes	rapamycin	159	INDIRECT

KDR	Multi-Organ	1	Yes	tamoxifen	159	INDIRECT
LCN2	Multi-Organ	1	No	fenofibrate	207	INDIRECT
LCN2	Multi-Organ	1	No	gold sodium thiomalate	207	INDIRECT
LCN2	Multi-Organ	1	No	ibuprofen	207	INDIRECT
LCN2	Multi-Organ	1	No	isoproterenol	329	INDIRECT
LCN2	Multi-Organ	1	No	methylprednisolone	207	INDIRECT
LCN2	Multi-Organ	1	No	piroxicam	207	INDIRECT
LCN2	Multi-Organ	1	No	rosiglitazone	207	INDIRECT
LIF	Multi-Organ	1	Yes	4-methylumbelliferone	187	DIRECT
LIF	Multi-Organ	1	Yes	4-methylumbelliferone	470	DIRECT
LIF	Multi-Organ	1	Yes	4-methylumbelliferone	950	DIRECT
LIF	Multi-Organ	1	Yes	imatinib	159	DIRECT
LIF	Multi-Organ	1	Yes	rapamycin	159	INDIRECT
LIF	Multi-Organ	1	Yes	tamoxifen	159	INDIRECT
LIFR	Marrow	2	Yes	imatinib	159	DIRECT
LIFR	Marrow	2	Yes	rapamycin	159	INDIRECT
LIFR	Marrow	2	Yes	tamoxifen	159	INDIRECT
LMNB1	Multi-Organ	1	No	etoposide	950	INDIRECT
LMNB1	Multi-Organ	1	No	lovastatin	916	INDIRECT
LRP1	Marrow	1	No	lovastatin	283	INDIRECT
LRP1	Marrow	1	No	probucol	283	INDIRECT
LRP1	Marrow	1	No	troglitazone	283	INDIRECT
LYN	Multi-Organ	3	Yes	imatinib	94	DIRECT
LYN	Multi-Organ	3	Yes	lynestrenol	950	INDIRECT
MAP2	Multi-Organ	2	Yes	estramustine	950	DIRECT
MAP2	Multi-Organ	2	Yes	imatinib	94	DIRECT
MAP2	Multi-Organ	2	Yes	riluzole	950	INDIRECT
MAPK14	Marrow	2	No	dibutyryl cyclic AMP	826	INDIRECT
MAPK14	Marrow	2	No	losartan	826	INDIRECT
MAPK14	Marrow	2	No	salicylate	826	INDIRECT
MASP1	Multi-Organ	1	No	ACA	98	DIRECT
MASP1	Multi-Organ	1	No	camostat mesilate	98	DIRECT
MASP1	Multi-Organ	1	No	diisopropylfluorophosphate	98	DIRECT
MASP1	Multi-Organ	1	No	warfarin	98	INDIRECT
MBP	Placenta	1	Yes	25-hydroxyvitamin D	274	DIRECT
MCL1	Fat	1	No	etoposide	950	INDIRECT
MCL1	Fat	1	No	fludarabine	950	INDIRECT
MCL1	Fat	1	No	imatinib	950	INDIRECT
MET	Marrow	4	Yes	4-methylumbelliferone	187	DIRECT
MET	Marrow	4	Yes	4-methylumbelliferone	470	DIRECT
MET	Marrow	4	Yes	4-methylumbelliferone	950	DIRECT
MET	Marrow	4	Yes	5-methylpyrazinecarboxylic acid 4-oxide	950	INDIRECT
MET	Marrow	4	Yes	8-methoxypsoralen	163	DIRECT
MET	Marrow	4	Yes	8-methoxypsoralen	950	DIRECT
MET	Marrow	4	Yes	8-methoxypsoralen	950	INDIRECT
MET	Marrow	4	Yes	alpha-methyl-p-tyrosine	950	DIRECT
MET	Marrow	4	Yes	beclomethasone	950	INDIRECT
MET	Marrow	4	Yes	bumetanide	718	DIRECT

MET	Marrow	4	Yes	bumetanide	950	DIRECT
MET	Marrow	4	Yes	bumetanide	950	INDIRECT
MET	Marrow	4	Yes	chlormethiazole	950	INDIRECT
MET	Marrow	4	Yes	cimetidine	182	DIRECT
MET	Marrow	4	Yes	cimetidine	511	INDIRECT
MET	Marrow	4	Yes	cimetidine	812	DIRECT
MET	Marrow	4	Yes	cimetidine	914	DIRECT
MET	Marrow	4	Yes	cimetidine	950	DIRECT
MET	Marrow	4	Yes	cimetidine	950	INDIRECT
MET	Marrow	4	Yes	dextromethorphan	624	DIRECT
MET	Marrow	4	Yes	dextromethorphan	950	DIRECT
MET	Marrow	4	Yes	diisopropylfluorophosphate	154	DIRECT_INDIRECT
MET	Marrow	4	Yes	dimethindene	950	DIRECT
MET	Marrow	4	Yes	diosmetin	163	DIRECT
MET	Marrow	4	Yes	diosmetin	950	DIRECT_INDIRECT
MET	Marrow	4	Yes	ergometrine	908	DIRECT
MET	Marrow	4	Yes	imatinib	160	DIRECT
MET	Marrow	4	Yes	indomethacin	610	DIRECT
MET	Marrow	4	Yes	indomethacin	713	DIRECT
MET	Marrow	4	Yes	indomethacin	868	INDIRECT
MET	Marrow	4	Yes	indomethacin	950	DIRECT
MET	Marrow	4	Yes	indomethacin	950	INDIRECT
MET	Marrow	4	Yes	metaproterenol	511	INDIRECT
MET	Marrow	4	Yes	metaraminol	694	DIRECT
MET	Marrow	4	Yes	metergoline	381	DIRECT
MET	Marrow	4	Yes	metergoline	511	INDIRECT
MET	Marrow	4	Yes	metergoline	696	DIRECT
MET	Marrow	4	Yes	metergoline	950	DIRECT
MET	Marrow	4	Yes	metergoline	950	DIRECT_IRRELEVANT
MET	Marrow	4	Yes	metformin	671	INDIRECT
MET	Marrow	4	Yes	metformin	865	INDIRECT
MET	Marrow	4	Yes	metformin	911	DIRECT
MET	Marrow	4	Yes	metformin	928	INDIRECT
MET	Marrow	4	Yes	metformin	950	DIRECT
MET	Marrow	4	Yes	metformin	950	INDIRECT
MET	Marrow	4	Yes	methadol	617	DIRECT
MET	Marrow	4	Yes	methadone	950	DIRECT
MET	Marrow	4	Yes	methadone	950	DIRECT_INDIRECT
MET	Marrow	4	Yes	methamphetamine	696	DIRECT_INDIRECT
MET	Marrow	4	Yes	methamphetamine	800	INDIRECT
MET	Marrow	4	Yes	methamphetamine	912	DIRECT_INDIRECT
MET	Marrow	4	Yes	methamphetamine	920	INDIRECT
MET	Marrow	4	Yes	methamphetamine	923	INDIRECT
MET	Marrow	4	Yes	methamphetamine	950	DIRECT
MET	Marrow	4	Yes	methamphetamine	950	DIRECT_INDIRECT
MET	Marrow	4	Yes	methamphetamine	950	INDIRECT
MET	Marrow	4	Yes	methazolamide	950	DIRECT
MET	Marrow	4	Yes	methimazole	692	DIRECT
MET	Marrow	4	Yes	methimazole	950	DIRECT
MET	Marrow	4	Yes	methimazole	950	INDIRECT
MET	Marrow	4	Yes	methohexital	489	DIRECT_INDIRECT
MET	Marrow	4	Yes	methotrexate	182	DIRECT_INDIREC

						T
MET	Marrow	4	Yes	methotrexate	868	INDIRECT
MET	Marrow	4	Yes	methotrexate	914	DIRECT
MET	Marrow	4	Yes	methotrexate	950	DIRECT
MET	Marrow	4	Yes	methotrexate	950	INDIRECT
MET	Marrow	4	Yes	methoxamine	808	DIRECT
MET	Marrow	4	Yes	methoxyflurane	950	DIRECT
MET	Marrow	4	Yes	methyldopa	934	DIRECT
MET	Marrow	4	Yes	methyldopa	950	DIRECT
MET	Marrow	4	Yes	methyldopa	950	INDIRECT
MET	Marrow	4	Yes	methylphenidate	696	INDIRECT
MET	Marrow	4	Yes	methylphenidate	950	DIRECT
MET	Marrow	4	Yes	methylphenidate	950	INDIRECT
MET	Marrow	4	Yes	methylprednisolone	207	INDIRECT
MET	Marrow	4	Yes	methylprednisolone	736	INDIRECT
MET	Marrow	4	Yes	methylprednisolone	950	INDIRECT
MET	Marrow	4	Yes	methylscopolamine	389	DIRECT
MET	Marrow	4	Yes	methylscopolamine	903	DIRECT
MET	Marrow	4	Yes	methyltestosterone	950	DIRECT
MET	Marrow	4	Yes	methysergide	381	DIRECT
MET	Marrow	4	Yes	methysergide	511	INDIRECT
MET	Marrow	4	Yes	methysergide	950	DIRECT
MET	Marrow	4	Yes	methysergide	950	INDIRECT
MET	Marrow	4	Yes	metipranolol	809	DIRECT
MET	Marrow	4	Yes	metoclopramide	381	DIRECT
MET	Marrow	4	Yes	metoclopramide	389	DIRECT
MET	Marrow	4	Yes	metoclopramide	800	DIRECT
MET	Marrow	4	Yes	metoclopramide	903	INDIRECT
MET	Marrow	4	Yes	metoclopramide	950	DIRECT
MET	Marrow	4	Yes	metoclopramide	950	INDIRECT
MET	Marrow	4	Yes	metoprolol	950	DIRECT
MET	Marrow	4	Yes	metyrapone	163	DIRECT
MET	Marrow	4	Yes	metyrapone	950	DIRECT
MET	Marrow	4	Yes	metyrapone	950	INDIRECT
MET	Marrow	4	Yes	mometasone	950	DIRECT_INDIREC T
MET	Marrow	4	Yes	nabumetone	950	DIRECT
MET	Marrow	4	Yes	octyl methoxycinnamate	950	DIRECT_IRRELE VANT
MET	Marrow	4	Yes	oxymetazoline	808	DIRECT
MET	Marrow	4	Yes	oxymetazoline	908	DIRECT
MET	Marrow	4	Yes	oxymetazoline	950	DIRECT
MET	Marrow	4	Yes	pemetrexed	950	DIRECT
MET	Marrow	4	Yes	pemetrexed	950	DIRECT_IRRELE VANT
MET	Marrow	4	Yes	promethazine	765	DIRECT
MET	Marrow	4	Yes	promethazine	903	DIRECT
MET	Marrow	4	Yes	promethazine	924	DIRECT
MET	Marrow	4	Yes	promethazine	950	DIRECT
MET	Marrow	4	Yes	promethazine	950	INDIRECT
MET	Marrow	4	Yes	rapamycin	159	INDIRECT
MET	Marrow	4	Yes	salmeterol	950	DIRECT
MET	Marrow	4	Yes	salmeterol	950	INDIRECT
MET	Marrow	4	Yes	tamoxifen	159	INDIRECT
MET	Marrow	4	Yes	tolmetin	476	DIRECT
MET	Marrow	4	Yes	tolmetin	713	DIRECT
MET	Marrow	4	Yes	tolmetin	950	DIRECT
MET	Marrow	4	Yes	trimetazidine	778	DIRECT
MET	Marrow	4	Yes	trimethaphan	556	DIRECT

MET	Marrow	4	Yes	trimetoquinol	950	DIRECT_INDIRECT
MKI67	Multi-Organ	1	No	procainamide	514	DIRECT_INDIRECT
MMP14	Multi-Organ	2	No	captopril	86	DIRECT
MMP14	Multi-Organ	2	No	diacerein	308	INDIRECT
MMP14	Multi-Organ	2	No	dibutyryl cyclic AMP	308	INDIRECT
MMP14	Multi-Organ	2	No	latanoprost	308	INDIRECT
MMP14	Multi-Organ	2	No	losartan	308	INDIRECT
MMP14	Multi-Organ	2	No	mifepristone	308	INDIRECT
MMP9	Placenta	1	No	captopril	86	DIRECT
MMP9	Placenta	1	No	diacerein	631	INDIRECT
MMP9	Placenta	1	No	dibutyryl cyclic AMP	905	INDIRECT
MMP9	Placenta	1	No	gold thioglucose	631	INDIRECT
MMP9	Placenta	1	No	indomethacin	950	INDIRECT
MMP9	Placenta	1	No	latanoprost	308	INDIRECT
MMP9	Placenta	1	No	losartan	308	INDIRECT
MMP9	Placenta	1	No	mifepristone	308	INDIRECT
MMP9	Placenta	1	No	rosiglitazone	950	INDIRECT
MST1	Liver	2	No	imatinib	160	DIRECT
MST1R	Bowel	1	No	imatinib	160	DIRECT
MYO10	Multi-Organ	1	No	clenbuterol	211	INDIRECT
MYO10	Multi-Organ	1	No	isoproterenol	211	INDIRECT
MYO10	Multi-Organ	1	No	phenylephrine	211	INDIRECT
MYO10	Multi-Organ	1	No	trifluoperazine	211	INDIRECT
MYO6	Marrow	2	No	clenbuterol	397	INDIRECT
MYO6	Marrow	2	No	isoproterenol	211	INDIRECT
MYO6	Marrow	2	No	phenylephrine	211	INDIRECT
MYO6	Marrow	2	No	trifluoperazine	211	INDIRECT
NFKBIA	Marrow	5	Yes	bortezomib	950	DIRECT_INDIRECT
NFKBIA	Marrow	5	Yes	sulfasalazine	950	INDIRECT
NOS1	Placenta	1	No	nitroglycerin	674	INDIRECT
NOS3	Heart	3	Yes	nitroglycerin	674	INDIRECT
NR2F1	Multi-Organ	1	Yes	Implanon	263	DIRECT
NR2F1	Multi-Organ	1	Yes	mifepristone	263	DIRECT
NR2F1	Multi-Organ	1	Yes	raloxifene	263	DIRECT
NR2F1	Multi-Organ	1	Yes	spironolactone	263	DIRECT
NR2F1	Multi-Organ	1	Yes	tamoxifen	263	DIRECT
NR2F2	Marrow	1	No	Implanon	263	DIRECT
NR2F2	Marrow	1	No	mifepristone	263	DIRECT
NR2F2	Marrow	1	No	raloxifene	263	DIRECT
NR2F2	Marrow	1	No	spironolactone	263	DIRECT
NR2F2	Marrow	1	No	tamoxifen	263	DIRECT
NRG1	Multi-Organ	1	Yes	riluzole	306	INDIRECT
NTF3	Placenta	2	Yes	riluzole	306	INDIRECT
NTN1	Multi-Organ	3	No	riluzole	306	INDIRECT
NTN4	Marrow	1	No	riluzole	306	INDIRECT
NTRK3	Kidney	2	Yes	imatinib	160	DIRECT
NTRK3	Kidney	2	Yes	rapamycin	159	INDIRECT
NTRK3	Kidney	2	Yes	tamoxifen	159	INDIRECT
OSM	Bowel	3	Yes	diosmetin	163	DIRECT
OSM	Bowel	3	Yes	diosmetin	950	DIRECT_INDIRECT
PDE5A	Multi-Organ	1	No	caffeine	134	DIRECT
PDE5A	Multi-Organ	1	No	glycerophosphocholine	134	DIRECT
PDE5A	Multi-Organ	1	No	milrinone	134	DIRECT

PDE5A	Multi-Organ	1	No	papaverine	134	DIRECT
PDE5A	Multi-Organ	1	No	pentoxifylline	134	DIRECT
PDE5A	Multi-Organ	1	No	sildenafil	950	DIRECT
PDE5A	Multi-Organ	1	No	tadalafil	950	DIRECT
PDGFRA	Multi-Organ	3	Yes	imatinib	950	DIRECT
PDGFRA	Multi-Organ	3	Yes	rapamycin	159	INDIRECT
PDGFRA	Multi-Organ	3	Yes	tamoxifen	159	INDIRECT
PDGFRB	Multi-Organ	6	Yes	imatinib	950	DIRECT
PDGFRB	Multi-Organ	6	Yes	rapamycin	159	INDIRECT
PDGFRB	Multi-Organ	6	Yes	tamoxifen	159	INDIRECT
PGF	Placenta	1	No	indomethacin	950	INDIRECT
PGR	Bowel	1	Yes	19-nortestosterone	950	DIRECT
PGR	Bowel	1	Yes	danazol	950	INDIRECT
PGR	Bowel	1	Yes	desogestrel	870	INDIRECT
PGR	Bowel	1	Yes	diethylstilbestrol	950	INDIRECT
PGR	Bowel	1	Yes	dydrogesterone	870	DIRECT
PGR	Bowel	1	Yes	estrogen	950	INDIRECT
PGR	Bowel	1	Yes	fulvestrant	950	INDIRECT
PGR	Bowel	1	Yes	gestrinone	950	DIRECT_IRRELEVANT
PGR	Bowel	1	Yes	Implanon	950	DIRECT
PGR	Bowel	1	Yes	letrozole	950	INDIRECT
PGR	Bowel	1	Yes	mifepristone	950	DIRECT
PGR	Bowel	1	Yes	nomegestrol	870	DIRECT
PGR	Bowel	1	Yes	norethisterone	950	DIRECT
PGR	Bowel	1	Yes	promegestone	950	DIRECT
PGR	Bowel	1	Yes	raloxifene	950	INDIRECT
PGR	Bowel	1	Yes	spironolactone	870	DIRECT_IRRELEVANT
PGR	Bowel	1	Yes	tamoxifen	950	INDIRECT
PGR	Bowel	1	Yes	tibolone	870	DIRECT
PGR	Bowel	1	Yes	toremifene	950	INDIRECT
PINK1	Heart	1	No	fenofibrate	207	INDIRECT
PINK1	Heart	1	No	gold sodium thiomalate	207	INDIRECT
PINK1	Heart	1	No	ibuprofen	207	INDIRECT
PINK1	Heart	1	No	methylprednisolone	207	INDIRECT
PINK1	Heart	1	No	piroxicam	207	INDIRECT
PINK1	Heart	1	No	rosiglitazone	207	INDIRECT
PLAU	Bowel	1	Yes	ACA	98	DIRECT
PLAU	Bowel	1	Yes	acenocoumarol	197	INDIRECT
PLAU	Bowel	1	Yes	amiloride	950	DIRECT
PLAU	Bowel	1	Yes	argatroban	671	INDIRECT
PLAU	Bowel	1	Yes	bezafibrate	197	INDIRECT
PLAU	Bowel	1	Yes	camostat mesilate	866	INDIRECT
PLAU	Bowel	1	Yes	desogestrel	197	INDIRECT
PLAU	Bowel	1	Yes	diisopropylfluorophosphate	98	DIRECT
PLAU	Bowel	1	Yes	gemfibrozil	671	INDIRECT
PLAU	Bowel	1	Yes	metformin	671	INDIRECT
PLAU	Bowel	1	Yes	stanozolol	671	INDIRECT
PLAU	Bowel	1	Yes	tranexamic acid	671	DIRECT
PLAU	Bowel	1	Yes	warfarin	197	INDIRECT
PON2	Multi-Organ	1	Yes	diisopropylfluorophosphate	922	DIRECT
PON2	Multi-Organ	1	Yes	paraoxon	922	DIRECT
PPARA	Multi-Organ	1	No	bezafibrate	950	DIRECT
PPARA	Multi-Organ	1	No	ciprofibrate	950	DIRECT
PPARA	Multi-Organ	1	No	clofibrate	950	DIRECT

PPARA	Multi-Organ	1	No	fenofibrate	950	DIRECT
PPARA	Multi-Organ	1	No	gemfibrozil	950	DIRECT
PPARA	Multi-Organ	1	No	troglitazone	950	DIRECT
PPARGC1A	Multi-Organ	2	Yes	rosiglitazone	950	INDIRECT
PPARGC1A	Multi-Organ	2	Yes	troglitazone	950	INDIRECT
PPBP	Multi-Organ	1	No	acenocoumarol	197	INDIRECT
PPBP	Multi-Organ	1	No	bezafibrate	197	INDIRECT
PPBP	Multi-Organ	1	No	cloricromene	950	INDIRECT
PPBP	Multi-Organ	1	No	desogestrel	197	INDIRECT
PPBP	Multi-Organ	1	No	fluticasone propionate	177	INDIRECT
PPBP	Multi-Organ	1	No	iloprost	950	INDIRECT
PPBP	Multi-Organ	1	No	indobufen	950	INDIRECT
PPBP	Multi-Organ	1	No	irbesartan	177	INDIRECT
PPBP	Multi-Organ	1	No	picotamide	950	INDIRECT
PPBP	Multi-Organ	1	No	ticlopidine	950	INDIRECT
PPBP	Multi-Organ	1	No	warfarin	197	INDIRECT
PRKAA2	Multi-Organ	1	Yes	acadesine	950	DIRECT_INDIRECT
PRKAA2	Multi-Organ	1	Yes	metformin	950	INDIRECT
PRKAR2A	Marrow	3	Yes	fasudil	582	DIRECT
PRKAR2B	Multi-Organ	3	Yes	fasudil	582	DIRECT
PRKCB	Multi-Organ	3	Yes	streptozotocin	950	INDIRECT
PRKG1	Marrow	1	No	fasudil	909	DIRECT
PRL	Placenta	2	No	alfentanil	617	DIRECT
PRL	Placenta	2	No	bromocriptine	950	INDIRECT
PRL	Placenta	2	No	buprenorphine	950	DIRECT
PRL	Placenta	2	No	butorphanol	617	DIRECT
PRL	Placenta	2	No	cocaine	617	DIRECT_INDIRECT
PRL	Placenta	2	No	codeine	617	DIRECT
PRL	Placenta	2	No	haloperidol	617	INDIRECT
PRL	Placenta	2	No	hydrocodone	617	DIRECT
PRL	Placenta	2	No	ketamine	617	DIRECT
PRL	Placenta	2	No	loperamide	617	DIRECT
PRL	Placenta	2	No	meperidine	617	DIRECT
PRL	Placenta	2	No	methadol	617	DIRECT
PRL	Placenta	2	No	naltrexone	617	DIRECT
PRL	Placenta	2	No	propoxyphene	617	DIRECT
PRL	Placenta	2	No	remifentanil	617	DIRECT
PRL	Placenta	2	No	tramadol	617	DIRECT_IRRELEVANT
PRL	Placenta	2	No	trimebutine	617	DIRECT
PRLR	Multi-Organ	1	No	bromocriptine	950	INDIRECT
PRTN3	Marrow	1	No	ACA	98	DIRECT
PRTN3	Marrow	1	No	camostat mesilate	98	DIRECT
PRTN3	Marrow	1	No	diisopropylfluorophosphate	98	DIRECT
PRTN3	Marrow	1	No	warfarin	98	INDIRECT
PTGS2	Multi-Organ	1	Yes	5-aminosalicylic acid	950	DIRECT_INDIRECT
PTGS2	Multi-Organ	1	Yes	acetaminophen	950	DIRECT
PTGS2	Multi-Organ	1	Yes	aminopyrine	950	DIRECT
PTGS2	Multi-Organ	1	Yes	aspirin	950	DIRECT
PTGS2	Multi-Organ	1	Yes	celecoxib	950	DIRECT
PTGS2	Multi-Organ	1	Yes	dibutyryl cyclic AMP	950	INDIRECT
PTGS2	Multi-Organ	1	Yes	diclofenac	950	DIRECT
PTGS2	Multi-Organ	1	Yes	diflunisal	950	DIRECT
PTGS2	Multi-Organ	1	Yes	etodolac	950	DIRECT

PTGS2	Multi-Organ	1	Yes	etoricoxib	950	DIRECT
PTGS2	Multi-Organ	1	Yes	fenoprofen	950	DIRECT
PTGS2	Multi-Organ	1	Yes	flufenamic acid	950	DIRECT
PTGS2	Multi-Organ	1	Yes	flurbiprofen	950	DIRECT
PTGS2	Multi-Organ	1	Yes	ibuprofen	950	DIRECT
PTGS2	Multi-Organ	1	Yes	indobufen	950	DIRECT
PTGS2	Multi-Organ	1	Yes	indomethacin	950	DIRECT
PTGS2	Multi-Organ	1	Yes	indoprofen	950	DIRECT
PTGS2	Multi-Organ	1	Yes	ketoprofen	950	DIRECT
PTGS2	Multi-Organ	1	Yes	ketorolac	950	DIRECT
PTGS2	Multi-Organ	1	Yes	lumiracoxib	950	DIRECT
PTGS2	Multi-Organ	1	Yes	meclofenamate	950	DIRECT
PTGS2	Multi-Organ	1	Yes	mefenamic acid	950	DIRECT
PTGS2	Multi-Organ	1	Yes	meloxicam	950	DIRECT
PTGS2	Multi-Organ	1	Yes	nabumetone	950	DIRECT
PTGS2	Multi-Organ	1	Yes	naproxen	950	DIRECT
PTGS2	Multi-Organ	1	Yes	niflumic acid	950	DIRECT
PTGS2	Multi-Organ	1	Yes	nimesulide	950	DIRECT
PTGS2	Multi-Organ	1	Yes	nordihydroguaiaretic acid	950	DIRECT
PTGS2	Multi-Organ	1	Yes	oxaprozin	950	DIRECT
PTGS2	Multi-Organ	1	Yes	oxyphenbutazone	950	DIRECT
PTGS2	Multi-Organ	1	Yes	phenylbutazone	950	DIRECT
PTGS2	Multi-Organ	1	Yes	pioglitazone	950	INDIRECT
PTGS2	Multi-Organ	1	Yes	piroxicam	950	DIRECT_IRRELEVANT
PTGS2	Multi-Organ	1	Yes	rofecoxib	950	DIRECT
PTGS2	Multi-Organ	1	Yes	salicylate	950	INDIRECT
PTGS2	Multi-Organ	1	Yes	streptozotocin	950	INDIRECT
PTGS2	Multi-Organ	1	Yes	sulfinpyrazone	950	DIRECT
PTGS2	Multi-Organ	1	Yes	sulindac	950	DIRECT
PTGS2	Multi-Organ	1	Yes	suprofen	950	DIRECT
PTGS2	Multi-Organ	1	Yes	tenoxicam	950	DIRECT
PTGS2	Multi-Organ	1	Yes	tiaprofenic acid	950	DIRECT
PTGS2	Multi-Organ	1	Yes	tolfenamic acid	950	DIRECT
PTGS2	Multi-Organ	1	Yes	tolmetin	950	DIRECT
PTGS2	Multi-Organ	1	Yes	triflusal	950	DIRECT
PTGS2	Multi-Organ	1	Yes	valdecoxib	950	DIRECT
PTGS2	Multi-Organ	1	Yes	zomepirac	950	DIRECT
PTN	Multi-Organ	1	No	amlexanox	364	DIRECT
REL	Multi-Organ	2	Yes	acetaminophen	950	DIRECT_IRRELEVANT
REL	Multi-Organ	2	Yes	bendrofluazide	474	DIRECT_IRRELEVANT
REL	Multi-Organ	2	Yes	benzydamine	950	DIRECT_IRRELEVANT
REL	Multi-Organ	2	Yes	bepridil	318	DIRECT_IRRELEVANT
REL	Multi-Organ	2	Yes	caffeine	950	DIRECT_IRRELEVANT
REL	Multi-Organ	2	Yes	carbenoxolone	950	DIRECT_IRRELEVANT
REL	Multi-Organ	2	Yes	carbetocin	950	DIRECT_IRRELEVANT
REL	Multi-Organ	2	Yes	celecoxib	950	DIRECT_IRRELEVANT
REL	Multi-Organ	2	Yes	clonidine	381	DIRECT_IRRELEVANT
REL	Multi-Organ	2	Yes	clopidogrel	511	INDIRECT
REL	Multi-Organ	2	Yes	clopidogrel	705	INDIRECT
REL	Multi-Organ	2	Yes	clopidogrel	950	DIRECT

REL	Multi-Organ	2	Yes	clopidogrel	950	INDIRECT
REL	Multi-Organ	2	Yes	clozapine	950	DIRECT_IRRELEVANT
REL	Multi-Organ	2	Yes	desogestrel	197	INDIRECT
REL	Multi-Organ	2	Yes	desogestrel	240	INDIRECT
REL	Multi-Organ	2	Yes	desogestrel	870	INDIRECT
REL	Multi-Organ	2	Yes	desogestrel	950	INDIRECT
REL	Multi-Organ	2	Yes	diethylstilbestrol	950	DIRECT_IRRELEVANT
REL	Multi-Organ	2	Yes	donepezil	950	DIRECT_IRRELEVANT
REL	Multi-Organ	2	Yes	ethopropazine	950	DIRECT_IRRELEVANT
REL	Multi-Organ	2	Yes	exemestane	950	DIRECT_IRRELEVANT
REL	Multi-Organ	2	Yes	fentanyl	950	DIRECT_IRRELEVANT
REL	Multi-Organ	2	Yes	gestrinone	950	DIRECT_IRRELEVANT
REL	Multi-Organ	2	Yes	granisetron	950	DIRECT_IRRELEVANT
REL	Multi-Organ	2	Yes	ibuprofen	713	DIRECT_IRRELEVANT
REL	Multi-Organ	2	Yes	isoflurane	844	DIRECT_IRRELEVANT
REL	Multi-Organ	2	Yes	ketanserin	950	DIRECT_IRRELEVANT
REL	Multi-Organ	2	Yes	lanreotide	950	DIRECT_IRRELEVANT
REL	Multi-Organ	2	Yes	melperone	800	DIRECT_IRRELEVANT
REL	Multi-Organ	2	Yes	metergoline	950	DIRECT_IRRELEVANT
REL	Multi-Organ	2	Yes	mianserin	950	DIRECT_IRRELEVANT
REL	Multi-Organ	2	Yes	mifepristone	950	DIRECT_IRRELEVANT
REL	Multi-Organ	2	Yes	modafinil	950	DIRECT_IRRELEVANT
REL	Multi-Organ	2	Yes	nifedipine	903	DIRECT_IRRELEVANT
REL	Multi-Organ	2	Yes	nimesulide	950	DIRECT_IRRELEVANT
REL	Multi-Organ	2	Yes	nortriptyline	950	DIRECT_IRRELEVANT
REL	Multi-Organ	2	Yes	octyl methoxycinnamate	950	DIRECT_IRRELEVANT
REL	Multi-Organ	2	Yes	pemetrexed	950	DIRECT_IRRELEVANT
REL	Multi-Organ	2	Yes	piroxicam	950	DIRECT_IRRELEVANT
REL	Multi-Organ	2	Yes	procaine	233	DIRECT_IRRELEVANT
REL	Multi-Organ	2	Yes	procaine	950	DIRECT_IRRELEVANT
REL	Multi-Organ	2	Yes	propofol	556	DIRECT_IRRELEVANT
REL	Multi-Organ	2	Yes	propranolol	381	DIRECT_IRRELEVANT
REL	Multi-Organ	2	Yes	propylthiouracil	184	INDIRECT
REL	Multi-Organ	2	Yes	rabeprazole	950	DIRECT_IRRELEVANT
REL	Multi-Organ	2	Yes	rosiglitazone	950	DIRECT_IRRELEVANT
REL	Multi-Organ	2	Yes	sertindole	800	DIRECT_IRRELE

						VANT
REL	Multi-Organ	2	Yes	sertraline	950	DIRECT_IRRELEVANT
REL	Multi-Organ	2	Yes	spironolactone	870	DIRECT_IRRELEVANT
REL	Multi-Organ	2	Yes	tolfenamic acid	950	DIRECT_IRRELEVANT
REL	Multi-Organ	2	Yes	tramadol	617	DIRECT_IRRELEVANT
REL	Multi-Organ	2	Yes	tramadol	950	DIRECT_IRRELEVANT
REL	Multi-Organ	2	Yes	urapidil	908	DIRECT_IRRELEVANT
REL	Multi-Organ	2	Yes	valdecoxib	950	DIRECT_IRRELEVANT
REL	Multi-Organ	2	Yes	vinorelbine	581	DIRECT
REL	Multi-Organ	2	Yes	yohimbine	696	DIRECT_IRRELEVANT
REN	Placenta	1	No	alprenolol	511	INDIRECT
REN	Placenta	1	No	alprenolol	585	DIRECT
REN	Placenta	1	No	alprenolol	809	DIRECT
REN	Placenta	1	No	alprenolol	950	DIRECT
REN	Placenta	1	No	alprenolol	950	INDIRECT
REN	Placenta	1	No	buprenorphine	950	DIRECT
REN	Placenta	1	No	canrenone	950	DIRECT
REN	Placenta	1	No	eplerenone	950	DIRECT
REN	Placenta	1	No	eplerenone	950	INDIRECT
REN	Placenta	1	No	hexoprenaline	950	INDIRECT
REN	Placenta	1	No	isoproterenol	211	INDIRECT
REN	Placenta	1	No	isoproterenol	317	INDIRECT
REN	Placenta	1	No	isoproterenol	329	INDIRECT
REN	Placenta	1	No	isoproterenol	511	INDIRECT
REN	Placenta	1	No	isoproterenol	609	INDIRECT
REN	Placenta	1	No	isoproterenol	619	INDIRECT
REN	Placenta	1	No	isoproterenol	692	INDIRECT
REN	Placenta	1	No	isoproterenol	711	INDIRECT
REN	Placenta	1	No	isoproterenol	795	INDIRECT
REN	Placenta	1	No	isoproterenol	809	DIRECT
REN	Placenta	1	No	isoproterenol	848	INDIRECT
REN	Placenta	1	No	isoproterenol	851	INDIRECT
REN	Placenta	1	No	isoproterenol	865	INDIRECT
REN	Placenta	1	No	isoproterenol	903	INDIRECT
REN	Placenta	1	No	isoproterenol	926	INDIRECT
REN	Placenta	1	No	isoproterenol	950	DIRECT
REN	Placenta	1	No	isoproterenol	950	INDIRECT
REN	Placenta	1	No	lynestrenol	950	INDIRECT
REN	Placenta	1	No	metaproterenol	511	INDIRECT
REN	Placenta	1	No	nitrendipine	233	DIRECT
REN	Placenta	1	No	nitrendipine	609	DIRECT
REN	Placenta	1	No	nitrendipine	950	INDIRECT
REN	Placenta	1	No	pirenzepine	511	INDIRECT
REN	Placenta	1	No	pirenzepine	950	INDIRECT
REN	Placenta	1	No	prenalterol	511	INDIRECT
REN	Placenta	1	No	prenalterol	809	DIRECT
REN	Placenta	1	No	prenalterol	950	DIRECT
REN	Placenta	1	No	prenalterol	950	INDIRECT
REN	Placenta	1	No	prenylamine	924	DIRECT
REN	Placenta	1	No	prenylamine	950	DIRECT
REN	Placenta	1	No	remikiren	950	DIRECT
RET	Multi-Organ	1	No	acitretin	950	DIRECT

RET	Multi-Organ	1	No	etretinate	950	DIRECT
RET	Multi-Organ	1	No	etretinate	950	INDIRECT
RET	Multi-Organ	1	No	imatinib	160	DIRECT
RET	Multi-Organ	1	No	metformin	950	INDIRECT
RET	Multi-Organ	1	No	nordihydroguaiaretic acid	171	DIRECT
RET	Multi-Organ	1	No	nordihydroguaiaretic acid	610	DIRECT
RET	Multi-Organ	1	No	nordihydroguaiaretic acid	635	DIRECT
RET	Multi-Organ	1	No	nordihydroguaiaretic acid	753	INDIRECT
RET	Multi-Organ	1	No	nordihydroguaiaretic acid	866	DIRECT
RET	Multi-Organ	1	No	nordihydroguaiaretic acid	898	DIRECT
RET	Multi-Organ	1	No	nordihydroguaiaretic acid	950	DIRECT
RET	Multi-Organ	1	No	nordihydroguaiaretic acid	950	INDIRECT
RET	Multi-Organ	1	No	norethisterone	950	DIRECT
RET	Multi-Organ	1	No	norethisterone	950	INDIRECT
RET	Multi-Organ	1	No	pioglitazone	950	INDIRECT
RET	Multi-Organ	1	No	rosiglitazone	950	INDIRECT
RIN1	Kidney	1	Yes	cocaine	950	INDIRECT
RIN1	Kidney	1	Yes	haloperidol	950	DIRECT
RIN1	Kidney	1	Yes	ifenprodil	950	DIRECT
RIN1	Kidney	1	Yes	memantine	950	DIRECT
RPS6KA1	Multi-Organ	2	Yes	rapamycin	950	INDIRECT
RPS6KA2	Multi-Organ	1	Yes	rapamycin	585	DIRECT
RRM1	Marrow	2	Yes	2-chlorodeoxyadenosine	766	DIRECT
RRM1	Marrow	2	Yes	5-fluorouracil	766	INDIRECT
RRM1	Marrow	2	Yes	DCF	766	INDIRECT
RRM1	Marrow	2	Yes	desferrioxamine	766	DIRECT
RRM1	Marrow	2	Yes	fludarabine	766	DIRECT
RRM1	Marrow	2	Yes	fotemustine	766	DIRECT
RRM1	Marrow	2	Yes	gemcitabine	766	INDIRECT
RRM1	Marrow	2	Yes	hydroxyurea	766	DIRECT
RRM2	Multi-Organ	1	Yes	2-chlorodeoxyadenosine	766	DIRECT
RRM2	Multi-Organ	1	Yes	5-fluorouracil	766	INDIRECT
RRM2	Multi-Organ	1	Yes	DCF	766	INDIRECT
RRM2	Multi-Organ	1	Yes	desferrioxamine	766	DIRECT
RRM2	Multi-Organ	1	Yes	fludarabine	766	DIRECT
RRM2	Multi-Organ	1	Yes	fotemustine	766	DIRECT
RRM2	Multi-Organ	1	Yes	gemcitabine	766	INDIRECT
RRM2	Multi-Organ	1	Yes	hydroxyurea	766	DIRECT
RRM2B	Multi-Organ	2	Yes	2-chlorodeoxyadenosine	766	DIRECT
RRM2B	Multi-Organ	2	Yes	5-fluorouracil	766	INDIRECT
RRM2B	Multi-Organ	2	Yes	DCF	766	INDIRECT
RRM2B	Multi-Organ	2	Yes	desferrioxamine	766	DIRECT
RRM2B	Multi-Organ	2	Yes	fludarabine	766	DIRECT
RRM2B	Multi-Organ	2	Yes	fotemustine	766	DIRECT
RRM2B	Multi-Organ	2	Yes	gemcitabine	766	INDIRECT
RRM2B	Multi-Organ	2	Yes	hydroxyurea	766	DIRECT
RXRA	Liver	2	No	tazarotene	511	DIRECT

RYR2	Heart	1	No	amiloride	233	DIRECT
RYR2	Heart	1	No	bepridil	233	DIRECT
RYR2	Heart	1	No	caffeine	865	DIRECT
RYR2	Heart	1	No	carbachol	233	INDIRECT
RYR2	Heart	1	No	Cardiovasc	233	DIRECT
RYR2	Heart	1	No	cilnidipine	233	DIRECT
RYR2	Heart	1	No	cinnarizine	233	DIRECT
RYR2	Heart	1	No	D-600	233	DIRECT
RYR2	Heart	1	No	dantrolene	865	DIRECT
RYR2	Heart	1	No	diltiazem	233	DIRECT
RYR2	Heart	1	No	felodipine	233	DIRECT
RYR2	Heart	1	No	gabapentin	233	DIRECT
RYR2	Heart	1	No	halothane	865	DIRECT_INDIRECT
RYR2	Heart	1	No	isoproterenol	950	INDIRECT
RYR2	Heart	1	No	lacidipine	233	DIRECT
RYR2	Heart	1	No	lamotrigine	233	DIRECT
RYR2	Heart	1	No	loperamide	233	DIRECT
RYR2	Heart	1	No	manidipine	233	DIRECT
RYR2	Heart	1	No	nifedipine	233	DIRECT
RYR2	Heart	1	No	nilvadipine	233	DIRECT
RYR2	Heart	1	No	nisoldipine	233	DIRECT
RYR2	Heart	1	No	nitrendipine	233	DIRECT
RYR2	Heart	1	No	phenytoin	233	DIRECT
RYR2	Heart	1	No	pimozide	233	DIRECT
RYR2	Heart	1	No	procaine	865	DIRECT
RYR2	Heart	1	No	rapamycin	865	DIRECT
RYR2	Heart	1	No	tetracaine	865	DIRECT
RYR2	Heart	1	No	verapamil	233	DIRECT
S	Lung	2	Yes	19-nortestosterone	240	INDIRECT
S	Lung	2	Yes	19-nortestosterone	950	DIRECT
S	Lung	2	Yes	19-nortestosterone	950	INDIRECT
S	Lung	2	Yes	2-chlorodeoxyadenosine	496	DIRECT
S	Lung	2	Yes	2-chlorodeoxyadenosine	766	DIRECT
S	Lung	2	Yes	2-chlorodeoxyadenosine	784	DIRECT
S	Lung	2	Yes	2-chlorodeoxyadenosine	950	DIRECT
S	Lung	2	Yes	2-chlorodeoxyadenosine	950	INDIRECT
S	Lung	2	Yes	25-hydroxyvitamin D	274	DIRECT
S	Lung	2	Yes	25-hydroxyvitamin D	950	DIRECT
S	Lung	2	Yes	3-morpholinosydnonimine	950	INDIRECT
S	Lung	2	Yes	4-hydroxyandrostenedione	950	DIRECT
S	Lung	2	Yes	4-hydroxyandrostenedione	950	INDIRECT
S	Lung	2	Yes	4-hydroxyanisole	950	DIRECT
S	Lung	2	Yes	4-methylumbelliferone	187	DIRECT

S	Lung	2	Yes	4-methylumbelliferone	470	DIRECT
S	Lung	2	Yes	5-aminosalicylic acid	866	DIRECT_INDIRECT
S	Lung	2	Yes	5-aminosalicylic acid	950	DIRECT
S	Lung	2	Yes	5-aminosalicylic acid	950	DIRECT_INDIRECT
S	Lung	2	Yes	5-fluorouracil	950	DIRECT
S	Lung	2	Yes	5-fluorouracil	950	INDIRECT
S	Lung	2	Yes	8-methoxypsoralen	163	DIRECT
S	Lung	2	Yes	8-methoxypsoralen	950	DIRECT
S	Lung	2	Yes	8-methoxypsoralen	950	INDIRECT
S	Lung	2	Yes	ACA	98	DIRECT
S	Lung	2	Yes	ACA	875	DIRECT
S	Lung	2	Yes	ACA	950	DIRECT_INDIRECT
S	Lung	2	Yes	acadesine	950	DIRECT
S	Lung	2	Yes	acadesine	950	DIRECT_INDIRECT
S	Lung	2	Yes	acadesine	950	INDIRECT
S	Lung	2	Yes	acamprosate	414	DIRECT
S	Lung	2	Yes	acarbose	240	INDIRECT
S	Lung	2	Yes	acarbose	778	DIRECT
S	Lung	2	Yes	acarbose	950	DIRECT
S	Lung	2	Yes	acarbose	950	INDIRECT
S	Lung	2	Yes	acebutolol	950	INDIRECT
S	Lung	2	Yes	acenocoumarol	197	INDIRECT
S	Lung	2	Yes	acenocoumarol	950	INDIRECT
S	Lung	2	Yes	acetaminophen	303	DIRECT
S	Lung	2	Yes	acetaminophen	366	INDIRECT
S	Lung	2	Yes	acetaminophen	655	INDIRECT
S	Lung	2	Yes	acetaminophen	923	INDIRECT
S	Lung	2	Yes	acetaminophen	934	DIRECT
S	Lung	2	Yes	acetaminophen	950	DIRECT
S	Lung	2	Yes	acetaminophen	950	DIRECT_IRRELEVANT
S	Lung	2	Yes	acetaminophen	950	INDIRECT
S	Lung	2	Yes	acetylcarnitine	213	DIRECT
S	Lung	2	Yes	acetylcarnitine	950	INDIRECT
S	Lung	2	Yes	acipimox	325	INDIRECT
S	Lung	2	Yes	acipimox	688	INDIRECT
S	Lung	2	Yes	acipimox	950	INDIRECT
S	Lung	2	Yes	aclarubicin	920	DIRECT
S	Lung	2	Yes	acrivastine	950	DIRECT
S	Lung	2	Yes	adenosylcobalamin	950	DIRECT
S	Lung	2	Yes	adenosylcobalamin	950	INDIRECT
S	Lung	2	Yes	alfentanil	617	DIRECT
S	Lung	2	Yes	alfuzosin	950	DIRECT
S	Lung	2	Yes	allopurinol	950	DIRECT
S	Lung	2	Yes	alosetron	950	DIRECT
S	Lung	2	Yes	alpha-methyl-p-tyrosine	950	DIRECT
S	Lung	2	Yes	alprazolam	163	DIRECT
S	Lung	2	Yes	alprenolol	950	INDIRECT
S	Lung	2	Yes	amantadine	800	INDIRECT
S	Lung	2	Yes	amantadine	923	INDIRECT
S	Lung	2	Yes	amiloride	197	DIRECT
S	Lung	2	Yes	amiloride	233	DIRECT
S	Lung	2	Yes	amiloride	318	DIRECT

S	Lung	2	Yes	amiloride	591	DIRECT
S	Lung	2	Yes	amiloride	866	DIRECT
S	Lung	2	Yes	amiloride	950	DIRECT
S	Lung	2	Yes	amiloride	950	INDIRECT
S	Lung	2	Yes	aminoglutethimide	163	DIRECT
S	Lung	2	Yes	aminoglutethimide	911	INDIRECT
S	Lung	2	Yes	aminopyrine	163	DIRECT
S	Lung	2	Yes	aminopyrine	303	DIRECT
S	Lung	2	Yes	aminopyrine	923	INDIRECT
S	Lung	2	Yes	aminopyrine	950	DIRECT
S	Lung	2	Yes	aminopyrine	950	INDIRECT
S	Lung	2	Yes	amiodarone	692	DIRECT
S	Lung	2	Yes	amisulpride	800	DIRECT
S	Lung	2	Yes	amlexanox	364	DIRECT
S	Lung	2	Yes	amlexanox	950	DIRECT
S	Lung	2	Yes	amlodipine	950	INDIRECT
S	Lung	2	Yes	amphetamine	696	INDIRECT
S	Lung	2	Yes	amphetamine	730	INDIRECT
S	Lung	2	Yes	amphetamine	920	INDIRECT
S	Lung	2	Yes	amphetamine	950	DIRECT
S	Lung	2	Yes	amphetamine	950	INDIRECT
S	Lung	2	Yes	amsacrine	920	DIRECT
S	Lung	2	Yes	amsacrine	950	DIRECT
S	Lung	2	Yes	anastrozole	950	DIRECT
S	Lung	2	Yes	antazoline	950	DIRECT
S	Lung	2	Yes	apomorphine	950	DIRECT
S	Lung	2	Yes	apomorphine	950	INDIRECT
S	Lung	2	Yes	argatroban	671	INDIRECT
S	Lung	2	Yes	argatroban	950	DIRECT
S	Lung	2	Yes	aripiprazole	800	DIRECT
S	Lung	2	Yes	aspirin	705	DIRECT_INDIRECT
S	Lung	2	Yes	aspirin	950	DIRECT
S	Lung	2	Yes	aspirin	950	INDIRECT
S	Lung	2	Yes	astemizole	765	DIRECT
S	Lung	2	Yes	astemizole	950	DIRECT
S	Lung	2	Yes	atenolol	950	INDIRECT
S	Lung	2	Yes	atomoxetine	950	DIRECT
S	Lung	2	Yes	atorvastatin	688	INDIRECT
S	Lung	2	Yes	atorvastatin	705	INDIRECT
S	Lung	2	Yes	atorvastatin	851	INDIRECT
S	Lung	2	Yes	atorvastatin	950	DIRECT
S	Lung	2	Yes	atorvastatin	950	INDIRECT
S	Lung	2	Yes	atosiban	950	DIRECT
S	Lung	2	Yes	atropine	389	DIRECT
S	Lung	2	Yes	atropine	950	DIRECT_INDIRECT
S	Lung	2	Yes	atropine	950	INDIRECT
S	Lung	2	Yes	azapropazone	476	DIRECT
S	Lung	2	Yes	azathioprine	366	INDIRECT
S	Lung	2	Yes	azathioprine	923	INDIRECT
S	Lung	2	Yes	azelastine	610	DIRECT
S	Lung	2	Yes	azelastine	866	INDIRECT
S	Lung	2	Yes	azelastine	950	DIRECT
S	Lung	2	Yes	azelastine	950	INDIRECT
S	Lung	2	Yes	baclofen	950	INDIRECT
S	Lung	2	Yes	BCNU	655	INDIRECT
S	Lung	2	Yes	BCNU	950	DIRECT

S	Lung	2	Yes	beclomethasone	950	INDIRECT
S	Lung	2	Yes	benazepril	950	INDIRECT
S	Lung	2	Yes	bendrofluazide	950	INDIRECT
S	Lung	2	Yes	benidipine	950	INDIRECT
S	Lung	2	Yes	benzbromarone	950	DIRECT_INDIRECT
S	Lung	2	Yes	benzocaine	318	DIRECT
S	Lung	2	Yes	benzoyl peroxide	950	INDIRECT
S	Lung	2	Yes	benztropine	950	DIRECT
S	Lung	2	Yes	bepridil	233	DIRECT
S	Lung	2	Yes	bepridil	318	DIRECT_IRRELEVANT
S	Lung	2	Yes	bepridil	924	DIRECT
S	Lung	2	Yes	bepridil	950	INDIRECT
S	Lung	2	Yes	beraprost sodium	511	INDIRECT
S	Lung	2	Yes	beraprost sodium	950	INDIRECT
S	Lung	2	Yes	betahistine	765	DIRECT
S	Lung	2	Yes	betahistine	900	DIRECT
S	Lung	2	Yes	betahistine	950	DIRECT
S	Lung	2	Yes	betaine	950	DIRECT
S	Lung	2	Yes	bezafibrate	197	INDIRECT
S	Lung	2	Yes	bezafibrate	240	INDIRECT
S	Lung	2	Yes	bezafibrate	302	INDIRECT
S	Lung	2	Yes	bezafibrate	325	INDIRECT
S	Lung	2	Yes	bezafibrate	618	INDIRECT
S	Lung	2	Yes	bezafibrate	684	DIRECT
S	Lung	2	Yes	bezafibrate	950	INDIRECT
S	Lung	2	Yes	bicalutamide	867	INDIRECT
S	Lung	2	Yes	bismuth subcitrate	950	INDIRECT
S	Lung	2	Yes	bisoprolol	950	DIRECT
S	Lung	2	Yes	bisoprolol	950	INDIRECT
S	Lung	2	Yes	bopindolol	950	INDIRECT
S	Lung	2	Yes	bortezomib	214	DIRECT
S	Lung	2	Yes	bortezomib	320	INDIRECT
S	Lung	2	Yes	bortezomib	872	INDIRECT
S	Lung	2	Yes	bortezomib	950	INDIRECT
S	Lung	2	Yes	bosentan	950	DIRECT
S	Lung	2	Yes	brimonidine	950	INDIRECT
S	Lung	2	Yes	bromocriptine	800	DIRECT
S	Lung	2	Yes	bromocriptine	950	INDIRECT
S	Lung	2	Yes	brompheniramine	950	DIRECT
S	Lung	2	Yes	budesonide	950	DIRECT
S	Lung	2	Yes	budesonide	950	INDIRECT
S	Lung	2	Yes	bumetanide	718	DIRECT
S	Lung	2	Yes	bumetanide	950	DIRECT
S	Lung	2	Yes	bumetanide	950	INDIRECT
S	Lung	2	Yes	bupropion	950	DIRECT
S	Lung	2	Yes	buspirone	511	INDIRECT
S	Lung	2	Yes	buspirone	696	DIRECT
S	Lung	2	Yes	buspirone	800	DIRECT
S	Lung	2	Yes	buspirone	950	DIRECT
S	Lung	2	Yes	busulfan	950	DIRECT
S	Lung	2	Yes	butorphanol	617	DIRECT
S	Lung	2	Yes	cabergoline	800	DIRECT
S	Lung	2	Yes	caffeine	134	DIRECT
S	Lung	2	Yes	caffeine	163	DIRECT
S	Lung	2	Yes	caffeine	233	INDIRECT
S	Lung	2	Yes	caffeine	317	DIRECT

S	Lung	2	Yes	caffeine	389	INDIRECT
S	Lung	2	Yes	caffeine	800	INDIRECT
S	Lung	2	Yes	caffeine	920	INDIRECT
S	Lung	2	Yes	calcipotriol	950	DIRECT
S	Lung	2	Yes	camostat mesilate	98	DIRECT
S	Lung	2	Yes	camostat mesilate	866	INDIRECT
S	Lung	2	Yes	camostat mesilate	938	DIRECT
S	Lung	2	Yes	camostat mesilate	950	INDIRECT
S	Lung	2	Yes	candesartan	950	DIRECT
S	Lung	2	Yes	candesartan	950	INDIRECT
S	Lung	2	Yes	capecitabine	138	DIRECT
S	Lung	2	Yes	capecitabine	749	DIRECT
S	Lung	2	Yes	capecitabine	950	DIRECT
S	Lung	2	Yes	capsaicin	676	DIRECT
S	Lung	2	Yes	capsaicin	950	DIRECT
S	Lung	2	Yes	capsaicin	950	INDIRECT
S	Lung	2	Yes	captopril	86	DIRECT
S	Lung	2	Yes	captopril	427	INDIRECT
S	Lung	2	Yes	carbachol	233	INDIRECT
S	Lung	2	Yes	carbachol	374	INDIRECT
S	Lung	2	Yes	carbachol	619	INDIRECT
S	Lung	2	Yes	carbachol	950	DIRECT_INDIRECT
S	Lung	2	Yes	carbachol	950	INDIRECT
S	Lung	2	Yes	carbamazepine	318	DIRECT
S	Lung	2	Yes	carbamazepine	950	INDIRECT
S	Lung	2	Yes	Cardiovasc	233	DIRECT
S	Lung	2	Yes	Cardiovasc	609	DIRECT
S	Lung	2	Yes	carteolol	163	DIRECT
S	Lung	2	Yes	carvedilol	950	INDIRECT
S	Lung	2	Yes	celecoxib	950	DIRECT
S	Lung	2	Yes	celecoxib	950	DIRECT_IRRELEVANT
S	Lung	2	Yes	celecoxib	950	INDIRECT
S	Lung	2	Yes	cerivastatin	950	DIRECT
S	Lung	2	Yes	cerivastatin	950	INDIRECT
S	Lung	2	Yes	cetirizine	950	DIRECT
S	Lung	2	Yes	chemical_name	matador_score	matador_annotation
S	Lung	2	Yes	chloral hydrate	163	DIRECT
S	Lung	2	Yes	chloroform	923	INDIRECT
S	Lung	2	Yes	chloropyramine	950	DIRECT
S	Lung	2	Yes	chlorothiazide	950	INDIRECT
S	Lung	2	Yes	chlorpheniramine	950	DIRECT
S	Lung	2	Yes	chlorpromazine	800	DIRECT
S	Lung	2	Yes	chlorpromazine	924	DIRECT
S	Lung	2	Yes	cibenzoline	950	DIRECT
S	Lung	2	Yes	ciclesonide	950	DIRECT
S	Lung	2	Yes	cicletanine	950	DIRECT
S	Lung	2	Yes	cilnidipine	233	DIRECT
S	Lung	2	Yes	cimetidine	182	DIRECT
S	Lung	2	Yes	cimetidine	950	DIRECT
S	Lung	2	Yes	cimetidine	950	INDIRECT
S	Lung	2	Yes	cinnarizine	233	DIRECT
S	Lung	2	Yes	cinnarizine	800	DIRECT
S	Lung	2	Yes	ciprofibrate	240	INDIRECT
S	Lung	2	Yes	ciprofibrate	302	INDIRECT
S	Lung	2	Yes	ciprofibrate	325	INDIRECT
S	Lung	2	Yes	ciprofibrate	655	INDIRECT

S	Lung	2	Yes	ciprofibrate	688	INDIRECT
S	Lung	2	Yes	ciprofibrate	950	INDIRECT
S	Lung	2	Yes	cisapride	903	DIRECT
S	Lung	2	Yes	cisapride	950	DIRECT
S	Lung	2	Yes	clebopride	800	DIRECT
S	Lung	2	Yes	clemastine	950	DIRECT
S	Lung	2	Yes	clenbuterol	211	INDIRECT
S	Lung	2	Yes	clofibrate	80	INDIRECT
S	Lung	2	Yes	clofibrate	163	INDIRECT
S	Lung	2	Yes	clofibrate	240	DIRECT
S	Lung	2	Yes	clofibrate	302	INDIRECT
S	Lung	2	Yes	clofibrate	325	INDIRECT
S	Lung	2	Yes	clofibrate	585	INDIRECT
S	Lung	2	Yes	clofibrate	688	INDIRECT
S	Lung	2	Yes	clofibrate	754	INDIRECT
S	Lung	2	Yes	clofibrate	950	INDIRECT
S	Lung	2	Yes	clomiphene citrate	950	DIRECT
S	Lung	2	Yes	clomiphene citrate	950	INDIRECT
S	Lung	2	Yes	clomipramine	950	DIRECT
S	Lung	2	Yes	clonidine	381	DIRECT_IRRELEVANT
S	Lung	2	Yes	clonidine	950	INDIRECT
S	Lung	2	Yes	clopidogrel	950	INDIRECT
S	Lung	2	Yes	clozapine	381	DIRECT
S	Lung	2	Yes	clozapine	950	DIRECT
S	Lung	2	Yes	clozapine	950	INDIRECT
S	Lung	2	Yes	cocaine	318	DIRECT
S	Lung	2	Yes	cocaine	617	DIRECT_INDIRECT
S	Lung	2	Yes	cocaine	800	INDIRECT
S	Lung	2	Yes	cocaine	920	INDIRECT
S	Lung	2	Yes	cocaine	950	DIRECT
S	Lung	2	Yes	cocaine	950	INDIRECT
S	Lung	2	Yes	codeine	617	DIRECT
S	Lung	2	Yes	colchicine	228	INDIRECT
S	Lung	2	Yes	colchicine	581	DIRECT
S	Lung	2	Yes	colchicine	923	INDIRECT
S	Lung	2	Yes	colchicine	950	INDIRECT
S	Lung	2	Yes	colesevelam hydrochloride	950	INDIRECT
S	Lung	2	Yes	cyproheptadine	381	DIRECT
S	Lung	2	Yes	cyproheptadine	950	DIRECT
S	Lung	2	Yes	cysteamine	734	INDIRECT
S	Lung	2	Yes	cytosine arabinoside	276	DIRECT
S	Lung	2	Yes	cytosine arabinoside	950	DIRECT
S	Lung	2	Yes	cytosine arabinoside	950	INDIRECT
S	Lung	2	Yes	D-600	233	DIRECT
S	Lung	2	Yes	d-tubocurarine	389	DIRECT
S	Lung	2	Yes	danazol	943	INDIRECT
S	Lung	2	Yes	danazol	950	DIRECT
S	Lung	2	Yes	danazol	950	INDIRECT
S	Lung	2	Yes	dantrolene	233	DIRECT
S	Lung	2	Yes	dantrolene	851	INDIRECT
S	Lung	2	Yes	daunorubicin	276	DIRECT
S	Lung	2	Yes	daunorubicin	920	DIRECT
S	Lung	2	Yes	DCF	741	INDIRECT
S	Lung	2	Yes	DDAVP	950	INDIRECT
S	Lung	2	Yes	debrisoquine	950	DIRECT
S	Lung	2	Yes	deferiprone	845	INDIRECT

S	Lung	2	Yes	delapril	950	INDIRECT
S	Lung	2	Yes	delta 9-tetrahydrocannabinol	950	DIRECT
S	Lung	2	Yes	deoxyspergualin	950	DIRECT
S	Lung	2	Yes	desferrioxamine	135	DIRECT
S	Lung	2	Yes	desferrioxamine	303	DIRECT
S	Lung	2	Yes	desferrioxamine	655	INDIRECT
S	Lung	2	Yes	desferrioxamine	684	INDIRECT
S	Lung	2	Yes	desferrioxamine	766	DIRECT
S	Lung	2	Yes	desferrioxamine	781	INDIRECT
S	Lung	2	Yes	desferrioxamine	845	INDIRECT
S	Lung	2	Yes	desferrioxamine	901	INDIRECT
S	Lung	2	Yes	desferrioxamine	918	INDIRECT
S	Lung	2	Yes	desferrioxamine	950	DIRECT
S	Lung	2	Yes	desferrioxamine	950	INDIRECT
S	Lung	2	Yes	desipramine	809	INDIRECT
S	Lung	2	Yes	desipramine	903	DIRECT
S	Lung	2	Yes	desipramine	908	DIRECT
S	Lung	2	Yes	desipramine	950	DIRECT
S	Lung	2	Yes	desipramine	950	INDIRECT
S	Lung	2	Yes	desloratadine	950	DIRECT
S	Lung	2	Yes	desloratadine	950	INDIRECT
S	Lung	2	Yes	desogestrel	197	INDIRECT
S	Lung	2	Yes	desogestrel	240	INDIRECT
S	Lung	2	Yes	desogestrel	870	INDIRECT
S	Lung	2	Yes	desogestrel	950	INDIRECT
S	Lung	2	Yes	dexetimide	389	DIRECT
S	Lung	2	Yes	dexrazoxane	920	DIRECT
S	Lung	2	Yes	dexrazoxane	950	DIRECT
S	Lung	2	Yes	diacerein	308	INDIRECT
S	Lung	2	Yes	diacerein	631	INDIRECT
S	Lung	2	Yes	diazepam	476	DIRECT
S	Lung	2	Yes	diazoxide	112	DIRECT
S	Lung	2	Yes	dibucaine	924	DIRECT
S	Lung	2	Yes	dibucaine	950	DIRECT
S	Lung	2	Yes	dibutyryl cyclic AMP	308	INDIRECT
S	Lung	2	Yes	dibutyryl cyclic AMP	480	INDIRECT
S	Lung	2	Yes	dibutyryl cyclic AMP	692	INDIRECT
S	Lung	2	Yes	dibutyryl cyclic AMP	711	INDIRECT
S	Lung	2	Yes	dibutyryl cyclic AMP	868	INDIRECT
S	Lung	2	Yes	dibutyryl cyclic AMP	924	INDIRECT
S	Lung	2	Yes	dibutyryl cyclic AMP	950	INDIRECT
S	Lung	2	Yes	diclofenac	163	DIRECT
S	Lung	2	Yes	diclofenac	950	DIRECT
S	Lung	2	Yes	diethylstilbestrol	303	DIRECT
S	Lung	2	Yes	diethylstilbestrol	573	DIRECT
S	Lung	2	Yes	diethylstilbestrol	713	DIRECT
S	Lung	2	Yes	diethylstilbestrol	950	DIRECT
S	Lung	2	Yes	diethylstilbestrol	950	DIRECT_IRRELEVANT
S	Lung	2	Yes	diethylstilbestrol	950	INDIRECT
S	Lung	2	Yes	diflunisal	713	DIRECT

S	Lung	2	Yes	diflunisal	950	DIRECT
S	Lung	2	Yes	digoxin	950	DIRECT
S	Lung	2	Yes	digoxin	950	INDIRECT
S	Lung	2	Yes	diiodotyrosine	692	DIRECT
S	Lung	2	Yes	diiodotyrosine	950	DIRECT
S	Lung	2	Yes	diisopropylfluorophosphate	98	DIRECT
S	Lung	2	Yes	diisopropylfluorophosphate	154	DIRECT_INDIRECT
S	Lung	2	Yes	diisopropylfluorophosphate	171	INDIRECT
S	Lung	2	Yes	diisopropylfluorophosphate	427	DIRECT
S	Lung	2	Yes	diisopropylfluorophosphate	706	INDIRECT
S	Lung	2	Yes	diisopropylfluorophosphate	749	DIRECT
S	Lung	2	Yes	diisopropylfluorophosphate	771	DIRECT
S	Lung	2	Yes	diisopropylfluorophosphate	893	INDIRECT
S	Lung	2	Yes	diisopropylfluorophosphate	901	DIRECT_INDIRECT
S	Lung	2	Yes	diisopropylfluorophosphate	903	INDIRECT
S	Lung	2	Yes	diisopropylfluorophosphate	906	DIRECT
S	Lung	2	Yes	diisopropylfluorophosphate	922	DIRECT
S	Lung	2	Yes	diisopropylfluorophosphate	950	DIRECT
S	Lung	2	Yes	diisopropylfluorophosphate	950	DIRECT_INDIRECT
S	Lung	2	Yes	dilazep	784	DIRECT
S	Lung	2	Yes	diltiazem	233	DIRECT
S	Lung	2	Yes	diltiazem	851	INDIRECT
S	Lung	2	Yes	diltiazem	950	INDIRECT
S	Lung	2	Yes	dimethindene	950	DIRECT
S	Lung	2	Yes	diosmetin	163	DIRECT
S	Lung	2	Yes	diosmetin	950	DIRECT_INDIRECT
S	Lung	2	Yes	dipalmitoylphosphatidylcholine	753	DIRECT
S	Lung	2	Yes	dipalmitoylphosphatidylcholine	945	DIRECT
S	Lung	2	Yes	dipalmitoylphosphatidylcholine	950	DIRECT
S	Lung	2	Yes	diphenhydramine	950	DIRECT
S	Lung	2	Yes	dipyridamole	784	DIRECT
S	Lung	2	Yes	dipyridamole	950	DIRECT
S	Lung	2	Yes	disopyramide	318	DIRECT
S	Lung	2	Yes	disopyramide	950	DIRECT
S	Lung	2	Yes	distigmine bromide	906	DIRECT
S	Lung	2	Yes	distigmine bromide	950	DIRECT
S	Lung	2	Yes	distigmine bromide	950	INDIRECT
S	Lung	2	Yes	disulfiram	582	DIRECT
S	Lung	2	Yes	disulfiram	950	DIRECT
S	Lung	2	Yes	dithranol	950	INDIRECT
S	Lung	2	Yes	dobutamine	950	INDIRECT
S	Lung	2	Yes	docetaxel	581	DIRECT
S	Lung	2	Yes	docetaxel	867	INDIRECT
S	Lung	2	Yes	docetaxel	950	DIRECT

S	Lung	2	Yes	docetaxel	950	INDIRECT
S	Lung	2	Yes	dolasetron	950	DIRECT
S	Lung	2	Yes	domperidone	800	DIRECT
S	Lung	2	Yes	doxazosin	240	INDIRECT
S	Lung	2	Yes	doxazosin	808	DIRECT
S	Lung	2	Yes	doxazosin	950	DIRECT
S	Lung	2	Yes	doxazosin	950	INDIRECT
S	Lung	2	Yes	doxepin	950	DIRECT
S	Lung	2	Yes	dydrogesterone	870	DIRECT
S	Lung	2	Yes	dydrogesterone	950	INDIRECT
S	Lung	2	Yes	ebastine	950	DIRECT
S	Lung	2	Yes	ebastine	950	INDIRECT
S	Lung	2	Yes	echothiophate	389	DIRECT
S	Lung	2	Yes	echothiophate	749	DIRECT_INDIRECT
S	Lung	2	Yes	encainide	318	DIRECT
S	Lung	2	Yes	enprostil	950	DIRECT
S	Lung	2	Yes	enprostil	950	INDIRECT
S	Lung	2	Yes	epinastine	694	DIRECT
S	Lung	2	Yes	epinastine	950	DIRECT
S	Lung	2	Yes	eplerenone	950	INDIRECT
S	Lung	2	Yes	eprosartan	950	DIRECT
S	Lung	2	Yes	eprosartan	950	INDIRECT
S	Lung	2	Yes	erdosteine	655	INDIRECT
S	Lung	2	Yes	erdosteine	950	INDIRECT
S	Lung	2	Yes	ergotamine	696	DIRECT
S	Lung	2	Yes	estramustine	867	INDIRECT
S	Lung	2	Yes	estramustine	950	DIRECT
S	Lung	2	Yes	estrogen	240	INDIRECT
S	Lung	2	Yes	estrogen	688	INDIRECT
S	Lung	2	Yes	estrogen	772	INDIRECT
S	Lung	2	Yes	estrogen	870	INDIRECT
S	Lung	2	Yes	estrogen	950	DIRECT
S	Lung	2	Yes	estrogen	950	INDIRECT
S	Lung	2	Yes	ethacrynic acid	950	DIRECT
S	Lung	2	Yes	ethosuximide	828	DIRECT
S	Lung	2	Yes	ethylmorphine	163	DIRECT
S	Lung	2	Yes	etodolac	950	DIRECT
S	Lung	2	Yes	etoposide	466	INDIRECT
S	Lung	2	Yes	etoposide	604	INDIRECT
S	Lung	2	Yes	etoposide	914	DIRECT
S	Lung	2	Yes	etoposide	916	INDIRECT
S	Lung	2	Yes	etoposide	920	DIRECT
S	Lung	2	Yes	etoposide	950	DIRECT
S	Lung	2	Yes	etoposide	950	DIRECT_INDIRECT
S	Lung	2	Yes	etoposide	950	INDIRECT
S	Lung	2	Yes	etoricoxib	950	DIRECT
S	Lung	2	Yes	etretinate	950	INDIRECT
S	Lung	2	Yes	Ets	950	DIRECT
S	Lung	2	Yes	everolimus	950	DIRECT
S	Lung	2	Yes	exemestane	950	DIRECT
S	Lung	2	Yes	exemestane	950	DIRECT_IRRELEVANT
S	Lung	2	Yes	ezetimibe	736	INDIRECT
S	Lung	2	Yes	famotidine	765	DIRECT
S	Lung	2	Yes	famotidine	950	DIRECT
S	Lung	2	Yes	fasudil	582	DIRECT
S	Lung	2	Yes	fasudil	619	INDIRECT

S	Lung	2	Yes	fasudil	909	DIRECT
S	Lung	2	Yes	fasudil	950	DIRECT
S	Lung	2	Yes	felodipine	163	DIRECT
S	Lung	2	Yes	felodipine	233	DIRECT
S	Lung	2	Yes	felodipine	924	DIRECT
S	Lung	2	Yes	felodipine	950	INDIRECT
S	Lung	2	Yes	fendiline	924	DIRECT
S	Lung	2	Yes	fenofibrate	207	INDIRECT
S	Lung	2	Yes	fenofibrate	688	INDIRECT
S	Lung	2	Yes	fenofibrate	726	INDIRECT
S	Lung	2	Yes	fenofibrate	950	INDIRECT
S	Lung	2	Yes	fenoldopam	696	DIRECT
S	Lung	2	Yes	fenoprofen	950	DIRECT
S	Lung	2	Yes	fenspiride	950	INDIRECT
S	Lung	2	Yes	ferrous fumarate	845	INDIRECT
S	Lung	2	Yes	ferrous fumarate	950	INDIRECT
S	Lung	2	Yes	fexofenadine	950	DIRECT
S	Lung	2	Yes	finasteride	867	INDIRECT
S	Lung	2	Yes	finasteride	904	DIRECT
S	Lung	2	Yes	finasteride	950	DIRECT
S	Lung	2	Yes	finasteride	950	INDIRECT
S	Lung	2	Yes	flecainide	318	DIRECT
S	Lung	2	Yes	flecainide	950	DIRECT_INDIRECT
S	Lung	2	Yes	fludarabine	784	DIRECT
S	Lung	2	Yes	fludarabine	920	INDIRECT
S	Lung	2	Yes	fludarabine	950	DIRECT
S	Lung	2	Yes	fludrocortisone	950	DIRECT
S	Lung	2	Yes	flufenamic acid	950	DIRECT
S	Lung	2	Yes	flunarizine	924	DIRECT
S	Lung	2	Yes	flunisolide	950	DIRECT
S	Lung	2	Yes	fluocinolone acetonide	950	INDIRECT
S	Lung	2	Yes	fluoxetine	950	DIRECT
S	Lung	2	Yes	fluoxymesterone	950	INDIRECT
S	Lung	2	Yes	flupenthixol	800	DIRECT
S	Lung	2	Yes	fluphenazine	800	DIRECT
S	Lung	2	Yes	fluphenazine	924	DIRECT
S	Lung	2	Yes	fluphenazine	950	INDIRECT
S	Lung	2	Yes	flurbiprofen	950	DIRECT
S	Lung	2	Yes	fluspirilene	696	DIRECT
S	Lung	2	Yes	fluspirilene	800	DIRECT
S	Lung	2	Yes	flutamide	950	INDIRECT
S	Lung	2	Yes	fluticasone propionate	163	DIRECT_INDIRECT
S	Lung	2	Yes	fluticasone propionate	177	INDIRECT
S	Lung	2	Yes	fluticasone propionate	950	DIRECT
S	Lung	2	Yes	fluticasone propionate	950	DIRECT_INDIRECT
S	Lung	2	Yes	fluticasone propionate	950	INDIRECT
S	Lung	2	Yes	fluvastatin	240	INDIRECT
S	Lung	2	Yes	fluvastatin	325	INDIRECT
S	Lung	2	Yes	fluvastatin	366	INDIRECT
S	Lung	2	Yes	fluvastatin	688	INDIRECT
S	Lung	2	Yes	fluvastatin	923	INDIRECT
S	Lung	2	Yes	fluvastatin	950	DIRECT
S	Lung	2	Yes	fluvastatin	950	INDIRECT

S	Lung	2	Yes	fondaparinux sodium	950	DIRECT
S	Lung	2	Yes	fondaparinux sodium	950	INDIRECT
S	Lung	2	Yes	fosinopril	950	DIRECT
S	Lung	2	Yes	fosinopril	950	INDIRECT
S	Lung	2	Yes	fotemustine	766	DIRECT
S	Lung	2	Yes	fotemustine	950	DIRECT
S	Lung	2	Yes	FP-CIT	950	DIRECT
S	Lung	2	Yes	fulvestrant	950	DIRECT
S	Lung	2	Yes	fulvestrant	950	DIRECT_INDIRECT
S	Lung	2	Yes	fulvestrant	950	INDIRECT
S	Lung	2	Yes	furosemide	713	DIRECT
S	Lung	2	Yes	furosemide	718	DIRECT
S	Lung	2	Yes	furosemide	784	INDIRECT
S	Lung	2	Yes	furosemide	950	DIRECT
S	Lung	2	Yes	furosemide	950	INDIRECT
S	Lung	2	Yes	gabapentin	233	DIRECT
S	Lung	2	Yes	gabapentin	318	DIRECT
S	Lung	2	Yes	gabapentin	366	DIRECT
S	Lung	2	Yes	gallamine	389	DIRECT
S	Lung	2	Yes	gamma-linolenic acid	950	DIRECT
S	Lung	2	Yes	gefitinib	683	INDIRECT
S	Lung	2	Yes	gemcitabine	784	DIRECT
S	Lung	2	Yes	gemcitabine	950	DIRECT_INDIRECT
S	Lung	2	Yes	gemcitabine	950	INDIRECT
S	Lung	2	Yes	gemfibrozil	240	INDIRECT
S	Lung	2	Yes	gemfibrozil	302	INDIRECT
S	Lung	2	Yes	gemfibrozil	671	INDIRECT
S	Lung	2	Yes	gemfibrozil	688	INDIRECT
S	Lung	2	Yes	gemfibrozil	726	INDIRECT
S	Lung	2	Yes	gemfibrozil	950	INDIRECT
S	Lung	2	Yes	gestrinone	950	DIRECT_IRRELEVANT
S	Lung	2	Yes	glibenclamide	240	INDIRECT
S	Lung	2	Yes	glibenclamide	911	INDIRECT
S	Lung	2	Yes	glibenclamide	950	INDIRECT
S	Lung	2	Yes	gliclazide	950	DIRECT
S	Lung	2	Yes	glimepiride	950	DIRECT
S	Lung	2	Yes	glimepiride	950	INDIRECT
S	Lung	2	Yes	glucuronic acid	531	DIRECT
S	Lung	2	Yes	glucuronic acid	674	DIRECT
S	Lung	2	Yes	glucuronic acid	950	DIRECT
S	Lung	2	Yes	glycerophosphocholine	134	DIRECT
S	Lung	2	Yes	glycerophosphocholine	171	INDIRECT
S	Lung	2	Yes	gold sodium thiomalate	207	INDIRECT
S	Lung	2	Yes	gold sodium thiomalate	736	INDIRECT
S	Lung	2	Yes	gold sodium thiomalate	950	INDIRECT
S	Lung	2	Yes	gold thioglucose	631	INDIRECT
S	Lung	2	Yes	gold thioglucose	655	DIRECT
S	Lung	2	Yes	gold thioglucose	911	INDIRECT
S	Lung	2	Yes	gold thioglucose	950	INDIRECT
S	Lung	2	Yes	granisetron	950	DIRECT

S	Lung	2	Yes	granisetron	950	DIRECT_IRRELEVANT
S	Lung	2	Yes	guanfacine	950	INDIRECT
S	Lung	2	Yes	haloperidol	617	INDIRECT
S	Lung	2	Yes	haloperidol	800	DIRECT
S	Lung	2	Yes	haloperidol	950	INDIRECT
S	Lung	2	Yes	halothane	163	DIRECT
S	Lung	2	Yes	halothane	233	DIRECT
S	Lung	2	Yes	halothane	923	INDIRECT
S	Lung	2	Yes	hexobarbital	163	DIRECT
S	Lung	2	Yes	hydralazine	950	INDIRECT
S	Lung	2	Yes	hydrochlorothiazide	950	INDIRECT
S	Lung	2	Yes	hydrocodone	617	DIRECT
S	Lung	2	Yes	hydroxyurea	950	INDIRECT
S	Lung	2	Yes	hydroxyzine	950	DIRECT
S	Lung	2	Yes	ibopamine	950	INDIRECT
S	Lung	2	Yes	ibuprofen	207	INDIRECT
S	Lung	2	Yes	ibuprofen	476	DIRECT
S	Lung	2	Yes	ibuprofen	950	DIRECT
S	Lung	2	Yes	idarubicin	920	DIRECT
S	Lung	2	Yes	ifosfamide	871	INDIRECT
S	Lung	2	Yes	ifosfamide	950	DIRECT
S	Lung	2	Yes	ifosfamide	950	DIRECT_INDIRECT
S	Lung	2	Yes	iloprost	511	DIRECT_INDIRECT
S	Lung	2	Yes	iloprost	866	INDIRECT
S	Lung	2	Yes	iloprost	936	INDIRECT
S	Lung	2	Yes	iloprost	950	DIRECT
S	Lung	2	Yes	iloprost	950	INDIRECT
S	Lung	2	Yes	imatinib	94	DIRECT
S	Lung	2	Yes	imatinib	159	DIRECT
S	Lung	2	Yes	imatinib	160	DIRECT
S	Lung	2	Yes	imatinib	950	INDIRECT
S	Lung	2	Yes	imidapril	950	INDIRECT
S	Lung	2	Yes	imipramine	800	INDIRECT
S	Lung	2	Yes	imipramine	924	DIRECT
S	Lung	2	Yes	imipramine	950	DIRECT
S	Lung	2	Yes	imiquimod	163	INDIRECT
S	Lung	2	Yes	Implanon	263	DIRECT
S	Lung	2	Yes	indapamide	950	INDIRECT
S	Lung	2	Yes	indobufen	950	DIRECT
S	Lung	2	Yes	indomethacin	868	INDIRECT
S	Lung	2	Yes	indomethacin	950	DIRECT
S	Lung	2	Yes	indomethacin	950	INDIRECT
S	Lung	2	Yes	indoprofen	950	DIRECT
S	Lung	2	Yes	iopanoic acid	692	DIRECT
S	Lung	2	Yes	ipratropium bromide	389	DIRECT
S	Lung	2	Yes	irbesartan	177	INDIRECT
S	Lung	2	Yes	irbesartan	950	DIRECT
S	Lung	2	Yes	irbesartan	950	INDIRECT
S	Lung	2	Yes	irinotecan	950	DIRECT
S	Lung	2	Yes	isocarboxazid	749	DIRECT
S	Lung	2	Yes	isocarboxazid	950	DIRECT
S	Lung	2	Yes	isoflurane	489	DIRECT
S	Lung	2	Yes	isoflurane	834	DIRECT_INDIRECT
S	Lung	2	Yes	isoflurane	844	DIRECT_IRRELE

						VANT
S	Lung	2	Yes	isoflurane	950	INDIRECT
S	Lung	2	Yes	isoproterenol	211	INDIRECT
S	Lung	2	Yes	isoproterenol	317	INDIRECT
S	Lung	2	Yes	isoproterenol	329	INDIRECT
S	Lung	2	Yes	isoproterenol	511	INDIRECT
S	Lung	2	Yes	isoproterenol	609	INDIRECT
S	Lung	2	Yes	isoproterenol	619	INDIRECT
S	Lung	2	Yes	isoproterenol	692	INDIRECT
S	Lung	2	Yes	isoproterenol	711	INDIRECT
S	Lung	2	Yes	isoproterenol	795	INDIRECT
S	Lung	2	Yes	isoproterenol	809	DIRECT
S	Lung	2	Yes	isoproterenol	848	INDIRECT
S	Lung	2	Yes	isoproterenol	851	INDIRECT
S	Lung	2	Yes	isoproterenol	865	INDIRECT
S	Lung	2	Yes	isoproterenol	903	INDIRECT
S	Lung	2	Yes	isoproterenol	926	INDIRECT
S	Lung	2	Yes	isoproterenol	950	DIRECT
S	Lung	2	Yes	isoproterenol	950	INDIRECT
S	Lung	2	Yes	isradipine	609	DIRECT
S	Lung	2	Yes	isradipine	828	DIRECT
S	Lung	2	Yes	ketamine	617	DIRECT
S	Lung	2	Yes	ketanserin	511	INDIRECT
S	Lung	2	Yes	ketanserin	808	DIRECT
S	Lung	2	Yes	ketanserin	920	DIRECT
S	Lung	2	Yes	ketanserin	950	DIRECT
S	Lung	2	Yes	ketanserin	950	DIRECT_IRRELE VANT
S	Lung	2	Yes	ketoprofen	476	DIRECT
S	Lung	2	Yes	ketoprofen	950	DIRECT
S	Lung	2	Yes	ketorolac	950	DIRECT
S	Lung	2	Yes	ketotifen	950	DIRECT
S	Lung	2	Yes	labetalol	950	INDIRECT
S	Lung	2	Yes	lacidipine	233	DIRECT
S	Lung	2	Yes	lafutidine	950	DIRECT
S	Lung	2	Yes	lamotrigine	233	DIRECT
S	Lung	2	Yes	lamotrigine	318	DIRECT
S	Lung	2	Yes	lanreotide	950	DIRECT
S	Lung	2	Yes	lanreotide	950	DIRECT_IRRELE VANT
S	Lung	2	Yes	lansoprazole	163	DIRECT
S	Lung	2	Yes	lansoprazole	950	DIRECT
S	Lung	2	Yes	lansoprazole	950	INDIRECT
S	Lung	2	Yes	latanoprost	308	INDIRECT
S	Lung	2	Yes	latanoprost	950	DIRECT
S	Lung	2	Yes	latanoprost	950	INDIRECT
S	Lung	2	Yes	leucovorin	950	DIRECT
S	Lung	2	Yes	levocabastine	730	DIRECT
S	Lung	2	Yes	levocabastine	950	DIRECT
S	Lung	2	Yes	levodopa	696	INDIRECT
S	Lung	2	Yes	levodopa	950	INDIRECT
S	Lung	2	Yes	levosimendan	617	DIRECT
S	Lung	2	Yes	levosimendan	848	INDIRECT
S	Lung	2	Yes	levosimendan	950	INDIRECT
S	Lung	2	Yes	lidocaine	318	DIRECT
S	Lung	2	Yes	lidocaine	950	DIRECT
S	Lung	2	Yes	Linomide	163	INDIRECT
S	Lung	2	Yes	lisuride	696	DIRECT

S	Lung	2	Yes	lisuride	800	DIRECT
S	Lung	2	Yes	lisuride	950	INDIRECT
S	Lung	2	Yes	looser	950	DIRECT
S	Lung	2	Yes	loperamide	233	DIRECT
S	Lung	2	Yes	loperamide	617	DIRECT
S	Lung	2	Yes	loratadine	950	DIRECT
S	Lung	2	Yes	losartan	86	DIRECT_INDIRECT
S	Lung	2	Yes	losartan	228	INDIRECT
S	Lung	2	Yes	losartan	308	INDIRECT
S	Lung	2	Yes	losartan	737	DIRECT
S	Lung	2	Yes	losartan	826	INDIRECT
S	Lung	2	Yes	losartan	950	DIRECT
S	Lung	2	Yes	losartan	950	INDIRECT
S	Lung	2	Yes	lovastatin	240	INDIRECT
S	Lung	2	Yes	lovastatin	265	INDIRECT
S	Lung	2	Yes	lovastatin	283	INDIRECT
S	Lung	2	Yes	lovastatin	325	INDIRECT
S	Lung	2	Yes	lovastatin	499	INDIRECT
S	Lung	2	Yes	lovastatin	505	INDIRECT
S	Lung	2	Yes	lovastatin	851	INDIRECT
S	Lung	2	Yes	lovastatin	916	INDIRECT
S	Lung	2	Yes	lovastatin	923	INDIRECT
S	Lung	2	Yes	lovastatin	950	DIRECT
S	Lung	2	Yes	lovastatin	950	INDIRECT
S	Lung	2	Yes	loxapine	950	DIRECT
S	Lung	2	Yes	lumiracoxib	950	DIRECT
S	Lung	2	Yes	lynestrenol	950	INDIRECT
S	Lung	2	Yes	manidipine	233	DIRECT
S	Lung	2	Yes	maprotiline	950	DIRECT
S	Lung	2	Yes	mazindol	950	DIRECT
S	Lung	2	Yes	meclofenamate	950	DIRECT
S	Lung	2	Yes	medroxyprogesterone	950	INDIRECT
S	Lung	2	Yes	mefenamic acid	950	DIRECT
S	Lung	2	Yes	melagatran	950	DIRECT
S	Lung	2	Yes	meloxicam	950	DIRECT
S	Lung	2	Yes	melperone	950	DIRECT
S	Lung	2	Yes	melphalan	196	DIRECT
S	Lung	2	Yes	melphalan	616	DIRECT
S	Lung	2	Yes	meperidine	617	DIRECT
S	Lung	2	Yes	mepyramine	950	DIRECT
S	Lung	2	Yes	mequitazine	950	DIRECT
S	Lung	2	Yes	mersalyl	762	INDIRECT
S	Lung	2	Yes	mersalyl	950	DIRECT_INDIRECT
S	Lung	2	Yes	mesoridazine	696	DIRECT
S	Lung	2	Yes	mesoridazine	950	DIRECT
S	Lung	2	Yes	metergoline	381	DIRECT
S	Lung	2	Yes	metergoline	696	DIRECT
S	Lung	2	Yes	metformin	671	INDIRECT
S	Lung	2	Yes	metformin	911	DIRECT
S	Lung	2	Yes	metformin	950	DIRECT
S	Lung	2	Yes	metformin	950	INDIRECT
S	Lung	2	Yes	methadol	617	DIRECT
S	Lung	2	Yes	methamphetamine	800	INDIRECT
S	Lung	2	Yes	methamphetamine	920	INDIRECT
S	Lung	2	Yes	methamphetamine	950	DIRECT
S	Lung	2	Yes	methamphetamine	950	DIRECT_INDIREC

						T
S	Lung	2	Yes	methamphetamine	950	INDIRECT
S	Lung	2	Yes	methimazole	950	DIRECT
S	Lung	2	Yes	methotrexate	182	DIRECT_INDIRECT
S	Lung	2	Yes	methotrexate	950	DIRECT
S	Lung	2	Yes	methotrexate	950	INDIRECT
S	Lung	2	Yes	methyldopa	934	DIRECT
S	Lung	2	Yes	methylphenidate	950	DIRECT
S	Lung	2	Yes	methylphenidate	950	INDIRECT
S	Lung	2	Yes	methylprednisolone	207	INDIRECT
S	Lung	2	Yes	methylprednisolone	736	INDIRECT
S	Lung	2	Yes	methylprednisolone	950	INDIRECT
S	Lung	2	Yes	methylscopolamine	389	DIRECT
S	Lung	2	Yes	methylscopolamine	903	DIRECT
S	Lung	2	Yes	methyltestosterone	950	DIRECT
S	Lung	2	Yes	methysergide	381	DIRECT
S	Lung	2	Yes	methysergide	511	INDIRECT
S	Lung	2	Yes	methysergide	950	DIRECT
S	Lung	2	Yes	methysergide	950	INDIRECT
S	Lung	2	Yes	metoclopramide	381	DIRECT
S	Lung	2	Yes	metoclopramide	389	DIRECT
S	Lung	2	Yes	metoclopramide	800	DIRECT
S	Lung	2	Yes	metoclopramide	950	INDIRECT
S	Lung	2	Yes	metyrapone	163	DIRECT
S	Lung	2	Yes	mexiletine	318	DIRECT
S	Lung	2	Yes	MGB	950	INDIRECT
S	Lung	2	Yes	mianserin	511	INDIRECT
S	Lung	2	Yes	mianserin	800	INDIRECT
S	Lung	2	Yes	mianserin	809	DIRECT_INDIRECT
S	Lung	2	Yes	mianserin	950	DIRECT
S	Lung	2	Yes	mianserin	950	DIRECT_IRRELEVANT
S	Lung	2	Yes	mianserin	950	INDIRECT
S	Lung	2	Yes	mifepristone	163	DIRECT
S	Lung	2	Yes	mifepristone	263	DIRECT
S	Lung	2	Yes	mifepristone	308	INDIRECT
S	Lung	2	Yes	mifepristone	461	INDIRECT
S	Lung	2	Yes	mifepristone	576	INDIRECT
S	Lung	2	Yes	mifepristone	950	DIRECT
S	Lung	2	Yes	mifepristone	950	DIRECT_IRRELEVANT
S	Lung	2	Yes	mifepristone	950	INDIRECT
S	Lung	2	Yes	miglitol	470	DIRECT
S	Lung	2	Yes	miglitol	950	DIRECT
S	Lung	2	Yes	milnacipran	950	DIRECT
S	Lung	2	Yes	milrinone	134	DIRECT
S	Lung	2	Yes	miltefosine	753	DIRECT
S	Lung	2	Yes	minoxidil	112	DIRECT_INDIRECT
S	Lung	2	Yes	minoxidil	934	DIRECT
S	Lung	2	Yes	minoxidil	950	INDIRECT
S	Lung	2	Yes	mirtazapine	950	DIRECT
S	Lung	2	Yes	misoprostol	584	DIRECT
S	Lung	2	Yes	misoprostol	705	DIRECT
S	Lung	2	Yes	mithramycin	950	DIRECT
S	Lung	2	Yes	mithramycin	950	INDIRECT
S	Lung	2	Yes	mitoxantrone	920	DIRECT

S	Lung	2	Yes	mitoxantrone	950	INDIRECT
S	Lung	2	Yes	mizolastine	950	DIRECT
S	Lung	2	Yes	modafinil	950	DIRECT_IRRELEVANT
S	Lung	2	Yes	mometasone	950	DIRECT_INDIRECT
S	Lung	2	Yes	monamine	950	DIRECT
S	Lung	2	Yes	montelukast	675	DIRECT
S	Lung	2	Yes	montelukast	950	DIRECT
S	Lung	2	Yes	montelukast	950	INDIRECT
S	Lung	2	Yes	morphine	950	INDIRECT
S	Lung	2	Yes	mosapramine	950	DIRECT
S	Lung	2	Yes	moxestrol	950	DIRECT
S	Lung	2	Yes	moxonidine	950	INDIRECT
S	Lung	2	Yes	N-butyldeoxynojirimycin	470	DIRECT
S	Lung	2	Yes	nabilone	900	DIRECT
S	Lung	2	Yes	nabumetone	950	DIRECT
S	Lung	2	Yes	naltrexone	617	DIRECT
S	Lung	2	Yes	naproxen	950	DIRECT
S	Lung	2	Yes	nebivolol	950	INDIRECT
S	Lung	2	Yes	nedocromil sodium	950	INDIRECT
S	Lung	2	Yes	nefazodone	950	DIRECT
S	Lung	2	Yes	neostigmine	389	INDIRECT
S	Lung	2	Yes	neostigmine	903	INDIRECT
S	Lung	2	Yes	neostigmine	950	DIRECT
S	Lung	2	Yes	niaprazine	920	DIRECT
S	Lung	2	Yes	nicorandil	950	DIRECT
S	Lung	2	Yes	nicotine	381	INDIRECT
S	Lung	2	Yes	nicotine	696	INDIRECT
S	Lung	2	Yes	nicotine	950	INDIRECT
S	Lung	2	Yes	nifedipine	163	DIRECT
S	Lung	2	Yes	nifedipine	233	DIRECT
S	Lung	2	Yes	nifedipine	619	INDIRECT
S	Lung	2	Yes	nifedipine	906	INDIRECT
S	Lung	2	Yes	nifedipine	924	INDIRECT
S	Lung	2	Yes	nifedipine	950	INDIRECT
S	Lung	2	Yes	niflumic acid	950	DIRECT
S	Lung	2	Yes	nilutamide	867	INDIRECT
S	Lung	2	Yes	nilvadipine	233	DIRECT
S	Lung	2	Yes	nimesulide	610	INDIRECT
S	Lung	2	Yes	nimesulide	950	DIRECT
S	Lung	2	Yes	nimesulide	950	DIRECT_IRRELEVANT
S	Lung	2	Yes	nisoldipine	233	DIRECT
S	Lung	2	Yes	nisoldipine	609	DIRECT
S	Lung	2	Yes	nitisinone	950	DIRECT
S	Lung	2	Yes	nitrendipine	233	DIRECT
S	Lung	2	Yes	nitrendipine	950	INDIRECT
S	Lung	2	Yes	nitroglycerin	674	INDIRECT
S	Lung	2	Yes	nitroglycerin	851	INDIRECT
S	Lung	2	Yes	nitroglycerin	950	INDIRECT
S	Lung	2	Yes	nizatidine	950	DIRECT
S	Lung	2	Yes	nomegestrol	870	DIRECT
S	Lung	2	Yes	nomifensine	950	DIRECT
S	Lung	2	Yes	nordihydroguaiaretic acid	171	DIRECT
S	Lung	2	Yes	nordihydroguaiaretic acid	950	DIRECT

S	Lung	2	Yes	nordihydroguaiaretic acid	950	INDIRECT
S	Lung	2	Yes	norethisterone	950	DIRECT
S	Lung	2	Yes	norethisterone	950	INDIRECT
S	Lung	2	Yes	nortriptyline	950	DIRECT
S	Lung	2	Yes	octopamine	950	DIRECT_INDIRECT
S	Lung	2	Yes	octyl methoxycinnamate	950	DIRECT_IRRELEVANT
S	Lung	2	Yes	olanzapine	800	DIRECT
S	Lung	2	Yes	olanzapine	950	DIRECT
S	Lung	2	Yes	olopatadine	950	DIRECT
S	Lung	2	Yes	ondansetron	950	DIRECT
S	Lung	2	Yes	orlistat	950	DIRECT
S	Lung	2	Yes	orlistat	950	INDIRECT
S	Lung	2	Yes	ouabain	560	INDIRECT
S	Lung	2	Yes	ouabain	950	INDIRECT
S	Lung	2	Yes	oxaprozin	950	DIRECT
S	Lung	2	Yes	oxyphenbutazone	950	DIRECT
S	Lung	2	Yes	pantethine	325	INDIRECT
S	Lung	2	Yes	pantethine	688	INDIRECT
S	Lung	2	Yes	papaverine	134	DIRECT
S	Lung	2	Yes	paraoxon	138	DIRECT_INDIRECT
S	Lung	2	Yes	paraoxon	163	DIRECT
S	Lung	2	Yes	paraoxon	749	DIRECT
S	Lung	2	Yes	paroxetine	950	DIRECT
S	Lung	2	Yes	pemetrexed	950	DIRECT
S	Lung	2	Yes	penfluridol	924	DIRECT
S	Lung	2	Yes	pentobarbital	950	INDIRECT
S	Lung	2	Yes	pentoxifylline	134	DIRECT
S	Lung	2	Yes	pentoxifylline	950	INDIRECT
S	Lung	2	Yes	pentylenetetrazol	950	INDIRECT
S	Lung	2	Yes	pergolide	800	DIRECT
S	Lung	2	Yes	perindopril	950	INDIRECT
S	Lung	2	Yes	perphenazine	800	DIRECT
S	Lung	2	Yes	phenindamine	950	DIRECT
S	Lung	2	Yes	pheniramine	950	DIRECT
S	Lung	2	Yes	phenobarbital	138	INDIRECT
S	Lung	2	Yes	phenobarbital	582	DIRECT_INDIRECT
S	Lung	2	Yes	phenobarbital	749	INDIRECT
S	Lung	2	Yes	phenobarbital	918	INDIRECT
S	Lung	2	Yes	phenobarbital	950	INDIRECT
S	Lung	2	Yes	phenol red	950	DIRECT
S	Lung	2	Yes	phenoxybenzamine	950	DIRECT
S	Lung	2	Yes	phenprocoumon	476	DIRECT
S	Lung	2	Yes	phentolamine	235	DIRECT
S	Lung	2	Yes	phentolamine	950	INDIRECT
S	Lung	2	Yes	phenylbutazone	476	DIRECT
S	Lung	2	Yes	phenylbutazone	950	DIRECT
S	Lung	2	Yes	phenylephrine	211	INDIRECT
S	Lung	2	Yes	phenylephrine	619	INDIRECT
S	Lung	2	Yes	phenylephrine	683	INDIRECT
S	Lung	2	Yes	phenylephrine	950	INDIRECT
S	Lung	2	Yes	phenytoin	233	DIRECT
S	Lung	2	Yes	phenytoin	318	DIRECT
S	Lung	2	Yes	phenytoin	950	INDIRECT
S	Lung	2	Yes	phosphocreatine	707	INDIRECT

S	Lung	2	Yes	phosphocreatine	851	DIRECT
S	Lung	2	Yes	phosphocreatine	950	INDIRECT
S	Lung	2	Yes	physostigmine	389	DIRECT
S	Lung	2	Yes	physostigmine	556	DIRECT
S	Lung	2	Yes	physostigmine	749	DIRECT
S	Lung	2	Yes	physostigmine	903	DIRECT
S	Lung	2	Yes	physostigmine	906	DIRECT
S	Lung	2	Yes	physostigmine	950	DIRECT
S	Lung	2	Yes	picotamide	950	DIRECT
S	Lung	2	Yes	pimozide	233	DIRECT
S	Lung	2	Yes	pimozide	800	DIRECT
S	Lung	2	Yes	pimozide	924	DIRECT
S	Lung	2	Yes	pinacidil	950	DIRECT
S	Lung	2	Yes	pindolol	381	DIRECT
S	Lung	2	Yes	pindolol	950	INDIRECT
S	Lung	2	Yes	pioglitazone	240	INDIRECT
S	Lung	2	Yes	pioglitazone	366	INDIRECT
S	Lung	2	Yes	pioglitazone	684	INDIRECT
S	Lung	2	Yes	pioglitazone	911	INDIRECT
S	Lung	2	Yes	pioglitazone	923	INDIRECT
S	Lung	2	Yes	pioglitazone	950	INDIRECT
S	Lung	2	Yes	pirarubicin	920	INDIRECT
S	Lung	2	Yes	pirenzepine	950	INDIRECT
S	Lung	2	Yes	piroxicam	207	INDIRECT
S	Lung	2	Yes	piroxicam	950	DIRECT
S	Lung	2	Yes	piroxicam	950	DIRECT_IRRELEVANT
S	Lung	2	Yes	pizotifen	381	DIRECT
S	Lung	2	Yes	polyinosinic-polycytidylic acid	163	INDIRECT
S	Lung	2	Yes	polyinosinic-polycytidylic acid	563	INDIRECT
S	Lung	2	Yes	polyinosinic-polycytidylic acid	681	INDIRECT
S	Lung	2	Yes	polyinosinic-polycytidylic acid	866	INDIRECT
S	Lung	2	Yes	polyinosinic-polycytidylic acid	898	INDIRECT
S	Lung	2	Yes	polyinosinic-polycytidylic acid	950	DIRECT
S	Lung	2	Yes	polyinosinic-polycytidylic acid	950	INDIRECT
S	Lung	2	Yes	pramipexole	800	DIRECT
S	Lung	2	Yes	pranlukast	675	DIRECT
S	Lung	2	Yes	pranlukast	950	DIRECT
S	Lung	2	Yes	pranlukast	950	INDIRECT
S	Lung	2	Yes	pravastatin	688	INDIRECT
S	Lung	2	Yes	pravastatin	736	INDIRECT
S	Lung	2	Yes	pravastatin	950	DIRECT
S	Lung	2	Yes	pravastatin	950	INDIRECT
S	Lung	2	Yes	prazosin	585	DIRECT
S	Lung	2	Yes	prazosin	694	DIRECT
S	Lung	2	Yes	prazosin	808	DIRECT
S	Lung	2	Yes	prazosin	809	DIRECT
S	Lung	2	Yes	prazosin	908	DIRECT
S	Lung	2	Yes	prazosin	915	DIRECT
S	Lung	2	Yes	prazosin	950	DIRECT
S	Lung	2	Yes	prazosin	950	INDIRECT
S	Lung	2	Yes	prednisolone	923	INDIRECT
S	Lung	2	Yes	prednisolone	950	DIRECT

S	Lung	2	Yes	prednisolone	950	INDIRECT
S	Lung	2	Yes	prednisone	950	DIRECT
S	Lung	2	Yes	prenalterol	950	INDIRECT
S	Lung	2	Yes	prenylamine	924	DIRECT
S	Lung	2	Yes	prenylamine	950	DIRECT
S	Lung	2	Yes	probenecid	182	DIRECT
S	Lung	2	Yes	probenecid	950	DIRECT
S	Lung	2	Yes	probucol	240	DIRECT
S	Lung	2	Yes	probucol	283	INDIRECT
S	Lung	2	Yes	probucol	302	INDIRECT
S	Lung	2	Yes	probucol	325	INDIRECT
S	Lung	2	Yes	probucol	655	INDIRECT
S	Lung	2	Yes	probucol	688	INDIRECT
S	Lung	2	Yes	probucol	892	INDIRECT
S	Lung	2	Yes	probucol	950	INDIRECT
S	Lung	2	Yes	procainamide	318	DIRECT
S	Lung	2	Yes	procainamide	514	DIRECT_INDIRECT
S	Lung	2	Yes	procaine	233	DIRECT_IRRELEVANT
S	Lung	2	Yes	procaine	318	DIRECT
S	Lung	2	Yes	procaine	749	DIRECT
S	Lung	2	Yes	procaine	950	DIRECT_IRRELEVANT
S	Lung	2	Yes	prochlorperazine	800	DIRECT
S	Lung	2	Yes	procyclidine	389	DIRECT_INDIRECT
S	Lung	2	Yes	proglumide	938	INDIRECT
S	Lung	2	Yes	promazine	696	DIRECT
S	Lung	2	Yes	promegestone	870	DIRECT
S	Lung	2	Yes	promegestone	950	DIRECT
S	Lung	2	Yes	promethazine	924	DIRECT
S	Lung	2	Yes	promethazine	950	DIRECT
S	Lung	2	Yes	propanidid	138	DIRECT
S	Lung	2	Yes	propanidid	749	DIRECT
S	Lung	2	Yes	propoxyphene	163	DIRECT
S	Lung	2	Yes	propoxyphene	617	DIRECT
S	Lung	2	Yes	propranolol	381	DIRECT_IRRELEVANT
S	Lung	2	Yes	propylthiouracil	184	INDIRECT
S	Lung	2	Yes	propylthiouracil	655	INDIRECT
S	Lung	2	Yes	propylthiouracil	950	DIRECT
S	Lung	2	Yes	pyridostigmine	906	DIRECT
S	Lung	2	Yes	pyridostigmine	950	DIRECT
S	Lung	2	Yes	pyridostigmine	950	INDIRECT
S	Lung	2	Yes	quetiapine	800	DIRECT
S	Lung	2	Yes	quetiapine	950	DIRECT
S	Lung	2	Yes	quinagolide	800	DIRECT
S	Lung	2	Yes	quinapril	950	INDIRECT
S	Lung	2	Yes	raloxifene	263	DIRECT
S	Lung	2	Yes	raloxifene	573	DIRECT
S	Lung	2	Yes	raloxifene	950	DIRECT
S	Lung	2	Yes	raloxifene	950	INDIRECT
S	Lung	2	Yes	ramipril	950	INDIRECT
S	Lung	2	Yes	ranitidine	950	DIRECT
S	Lung	2	Yes	rapamycin	159	INDIRECT
S	Lung	2	Yes	rapamycin	163	INDIRECT
S	Lung	2	Yes	rapamycin	169	INDIRECT
S	Lung	2	Yes	rapamycin	320	INDIRECT

S	Lung	2	Yes	rapamycin	585	DIRECT
S	Lung	2	Yes	rapamycin	892	DIRECT
S	Lung	2	Yes	rapamycin	926	INDIRECT
S	Lung	2	Yes	rapamycin	929	INDIRECT
S	Lung	2	Yes	rapamycin	950	INDIRECT
S	Lung	2	Yes	Ras	950	DIRECT
S	Lung	2	Yes	Ras	950	INDIRECT
S	Lung	2	Yes	reboxetine	950	DIRECT
S	Lung	2	Yes	remifentanil	617	DIRECT
S	Lung	2	Yes	remikiren	950	DIRECT
S	Lung	2	Yes	remoxipride	800	DIRECT
S	Lung	2	Yes	repaglinide	112	DIRECT
S	Lung	2	Yes	repaglinide	950	DIRECT
S	Lung	2	Yes	reserpine	169	DIRECT
S	Lung	2	Yes	reserpine	511	INDIRECT
S	Lung	2	Yes	reserpine	804	DIRECT
S	Lung	2	Yes	reserpine	808	INDIRECT
S	Lung	2	Yes	reserpine	809	INDIRECT
S	Lung	2	Yes	reserpine	903	INDIRECT
S	Lung	2	Yes	reserpine	914	DIRECT
S	Lung	2	Yes	reserpine	920	DIRECT
S	Lung	2	Yes	reserpine	923	INDIRECT
S	Lung	2	Yes	reserpine	950	DIRECT
S	Lung	2	Yes	reserpine	950	INDIRECT
S	Lung	2	Yes	reset	316	DIRECT
S	Lung	2	Yes	reset	818	DIRECT
S	Lung	2	Yes	reset	844	DIRECT
S	Lung	2	Yes	reset	950	DIRECT
S	Lung	2	Yes	reset	950	DIRECT_INDIRECT
S	Lung	2	Yes	riluzole	306	INDIRECT
S	Lung	2	Yes	riluzole	318	DIRECT
S	Lung	2	Yes	risedronate	950	DIRECT
S	Lung	2	Yes	risedronate	950	INDIRECT
S	Lung	2	Yes	risperidone	694	DIRECT
S	Lung	2	Yes	risperidone	800	DIRECT
S	Lung	2	Yes	risperidone	808	DIRECT
S	Lung	2	Yes	risperidone	908	DIRECT
S	Lung	2	Yes	risperidone	912	DIRECT
S	Lung	2	Yes	risperidone	950	DIRECT
S	Lung	2	Yes	rivastigmine	906	DIRECT
S	Lung	2	Yes	rivastigmine	950	DIRECT
S	Lung	2	Yes	rofecoxib	950	DIRECT
S	Lung	2	Yes	Ronicol	240	INDIRECT
S	Lung	2	Yes	ropinirole	800	DIRECT
S	Lung	2	Yes	ropivacaine	318	DIRECT
S	Lung	2	Yes	rose bengal	708	DIRECT
S	Lung	2	Yes	rosiglitazone	207	INDIRECT
S	Lung	2	Yes	rosiglitazone	240	INDIRECT
S	Lung	2	Yes	rosiglitazone	499	INDIRECT
S	Lung	2	Yes	rosiglitazone	736	INDIRECT
S	Lung	2	Yes	rosiglitazone	911	INDIRECT
S	Lung	2	Yes	rosiglitazone	950	DIRECT
S	Lung	2	Yes	rosiglitazone	950	DIRECT_IRRELEVANT
S	Lung	2	Yes	rosiglitazone	950	INDIRECT
S	Lung	2	Yes	rosuvastatin	240	INDIRECT
S	Lung	2	Yes	rosuvastatin	325	INDIRECT

S	Lung	2	Yes	rosuvastatin	688	INDIRECT
S	Lung	2	Yes	rosuvastatin	736	INDIRECT
S	Lung	2	Yes	rosuvastatin	950	DIRECT
S	Lung	2	Yes	rosuvastatin	950	INDIRECT
S	Lung	2	Yes	roxatidine	950	DIRECT
S	Lung	2	Yes	salbutamol	511	INDIRECT
S	Lung	2	Yes	salbutamol	809	DIRECT
S	Lung	2	Yes	salbutamol	950	DIRECT
S	Lung	2	Yes	salbutamol	950	INDIRECT
S	Lung	2	Yes	salicylate	480	INDIRECT
S	Lung	2	Yes	salicylate	610	DIRECT
S	Lung	2	Yes	salicylate	674	INDIRECT
S	Lung	2	Yes	salicylate	713	DIRECT
S	Lung	2	Yes	salicylate	734	DIRECT
S	Lung	2	Yes	salicylate	826	INDIRECT
S	Lung	2	Yes	salicylate	909	INDIRECT
S	Lung	2	Yes	salicylate	950	DIRECT
S	Lung	2	Yes	salicylate	950	INDIRECT
S	Lung	2	Yes	salmeterol	950	DIRECT
S	Lung	2	Yes	salmeterol	950	INDIRECT
S	Lung	2	Yes	SCMC	950	INDIRECT
S	Lung	2	Yes	scopolamine	556	DIRECT
S	Lung	2	Yes	scopolamine	696	DIRECT
S	Lung	2	Yes	scopolamine	903	DIRECT
S	Lung	2	Yes	scopolamine	950	INDIRECT
S	Lung	2	Yes	secobarbital	489	DIRECT
S	Lung	2	Yes	sedormid	950	INDIRECT
S	Lung	2	Yes	selegiline	950	DIRECT
S	Lung	2	Yes	selegiline	950	INDIRECT
S	Lung	2	Yes	sertindole	800	DIRECT_IRRELEVANT
S	Lung	2	Yes	sertindole	950	DIRECT
S	Lung	2	Yes	sertraline	950	DIRECT
S	Lung	2	Yes	sertraline	950	DIRECT_IRRELEVANT
S	Lung	2	Yes	sevoflurane	489	DIRECT
S	Lung	2	Yes	sevoflurane	834	DIRECT
S	Lung	2	Yes	sevoflurane	923	INDIRECT
S	Lung	2	Yes	sevoflurane	950	DIRECT
S	Lung	2	Yes	sibutramine	950	DIRECT
S	Lung	2	Yes	sildenafil	950	DIRECT
S	Lung	2	Yes	silymarin	228	INDIRECT
S	Lung	2	Yes	silymarin	320	INDIRECT
S	Lung	2	Yes	silymarin	366	INDIRECT
S	Lung	2	Yes	silymarin	655	INDIRECT
S	Lung	2	Yes	silymarin	772	INDIRECT
S	Lung	2	Yes	silymarin	906	INDIRECT
S	Lung	2	Yes	silymarin	923	INDIRECT
S	Lung	2	Yes	silymarin	950	DIRECT_INDIRECT
S	Lung	2	Yes	silymarin	950	INDIRECT
S	Lung	2	Yes	simvastatin	950	DIRECT
S	Lung	2	Yes	simvastatin	950	INDIRECT
S	Lung	2	Yes	sotalol	809	DIRECT
S	Lung	2	Yes	sparteine	950	DIRECT
S	Lung	2	Yes	spirapril	950	DIRECT
S	Lung	2	Yes	spirapril	950	INDIRECT
S	Lung	2	Yes	spironolactone	97	INDIRECT
S	Lung	2	Yes	spironolactone	228	INDIRECT

S	Lung	2	Yes	spironolactone	263	DIRECT
S	Lung	2	Yes	spironolactone	870	DIRECT_IRRELEVANT
S	Lung	2	Yes	spironolactone	950	DIRECT
S	Lung	2	Yes	spironolactone	950	DIRECT_INDIRECT
S	Lung	2	Yes	spironolactone	950	INDIRECT
S	Lung	2	Yes	SR 57746A	950	INDIRECT
S	Lung	2	Yes	stanozolol	671	INDIRECT
S	Lung	2	Yes	stanozolol	865	INDIRECT
S	Lung	2	Yes	stanozolol	950	INDIRECT
S	Lung	2	Yes	streptozotocin	240	INDIRECT
S	Lung	2	Yes	streptozotocin	511	INDIRECT
S	Lung	2	Yes	streptozotocin	800	INDIRECT
S	Lung	2	Yes	streptozotocin	809	INDIRECT
S	Lung	2	Yes	streptozotocin	926	INDIRECT
S	Lung	2	Yes	streptozotocin	950	DIRECT
S	Lung	2	Yes	streptozotocin	950	INDIRECT
S	Lung	2	Yes	succinylcholine	556	DIRECT_INDIRECT
S	Lung	2	Yes	succinylcholine	851	INDIRECT
S	Lung	2	Yes	succinylcholine	903	DIRECT
S	Lung	2	Yes	succinylcholine	950	DIRECT
S	Lung	2	Yes	succinylcholine	950	INDIRECT
S	Lung	2	Yes	sufentanil	950	DIRECT
S	Lung	2	Yes	sulfasalazine	163	INDIRECT
S	Lung	2	Yes	sulfasalazine	610	DIRECT
S	Lung	2	Yes	sulfasalazine	691	DIRECT
S	Lung	2	Yes	sulfasalazine	866	DIRECT
S	Lung	2	Yes	sulfasalazine	950	DIRECT
S	Lung	2	Yes	sulfasalazine	950	INDIRECT
S	Lung	2	Yes	sulfinpyrazone	182	DIRECT
S	Lung	2	Yes	sulfinpyrazone	950	DIRECT
S	Lung	2	Yes	sulfobromophthalein	613	DIRECT
S	Lung	2	Yes	sulfobromophthalein	950	DIRECT
S	Lung	2	Yes	sulindac	950	DIRECT
S	Lung	2	Yes	sulindac	950	DIRECT_INDIRECT
S	Lung	2	Yes	sulindac	950	INDIRECT
S	Lung	2	Yes	sulpiride	696	DIRECT
S	Lung	2	Yes	sulpiride	800	DIRECT
S	Lung	2	Yes	sulprostone	511	INDIRECT
S	Lung	2	Yes	sulprostone	950	DIRECT
S	Lung	2	Yes	sulprostone	950	INDIRECT
S	Lung	2	Yes	sumatriptan	381	DIRECT
S	Lung	2	Yes	sumatriptan	950	DIRECT
S	Lung	2	Yes	sumatriptan	950	INDIRECT
S	Lung	2	Yes	suprofen	950	DIRECT
S	Lung	2	Yes	synephrine	950	DIRECT
S	Lung	2	Yes	tacrolimus	254	DIRECT
S	Lung	2	Yes	tacrolimus	449	DIRECT
S	Lung	2	Yes	tacrolimus	868	DIRECT
S	Lung	2	Yes	tacrolimus	914	DIRECT
S	Lung	2	Yes	tacrolimus	950	DIRECT
S	Lung	2	Yes	tacrolimus	950	INDIRECT
S	Lung	2	Yes	tamoxifen	159	INDIRECT
S	Lung	2	Yes	tamoxifen	263	DIRECT

S	Lung	2	Yes	tamoxifen	573	DIRECT
S	Lung	2	Yes	tamoxifen	924	DIRECT
S	Lung	2	Yes	tamoxifen	950	DIRECT
S	Lung	2	Yes	tamoxifen	950	INDIRECT
S	Lung	2	Yes	tamsulosin	808	DIRECT
S	Lung	2	Yes	tazarotene	511	DIRECT
S	Lung	2	Yes	tazarotene	950	INDIRECT
S	Lung	2	Yes	tegafur	950	INDIRECT
S	Lung	2	Yes	tegaserod	950	DIRECT
S	Lung	2	Yes	telmisartan	950	DIRECT
S	Lung	2	Yes	teniposide	920	DIRECT
S	Lung	2	Yes	teniposide	950	DIRECT_INDIRECT
S	Lung	2	Yes	tenoxicam	950	DIRECT
S	Lung	2	Yes	terazosin	808	DIRECT
S	Lung	2	Yes	terbutaline	950	INDIRECT
S	Lung	2	Yes	terfenadine	950	DIRECT
S	Lung	2	Yes	terlipressin	950	INDIRECT
S	Lung	2	Yes	tetrabenazine	696	DIRECT
S	Lung	2	Yes	tetrabenazine	804	DIRECT
S	Lung	2	Yes	tetrabenazine	920	DIRECT
S	Lung	2	Yes	tetrabenazine	923	DIRECT
S	Lung	2	Yes	tetrabenazine	950	DIRECT
S	Lung	2	Yes	thalidomide	163	INDIRECT
S	Lung	2	Yes	thioguanine	276	DIRECT
S	Lung	2	Yes	thioridazine	696	DIRECT
S	Lung	2	Yes	thioridazine	924	DIRECT
S	Lung	2	Yes	thiothixene	696	DIRECT
S	Lung	2	Yes	tiadenol	739	INDIRECT
S	Lung	2	Yes	tiadenol	950	INDIRECT
S	Lung	2	Yes	tiagabine	950	DIRECT
S	Lung	2	Yes	tiapride	800	DIRECT
S	Lung	2	Yes	tiaprofenic acid	950	DIRECT
S	Lung	2	Yes	tiazofurin	950	INDIRECT
S	Lung	2	Yes	tibolone	240	INDIRECT
S	Lung	2	Yes	tibolone	325	INDIRECT
S	Lung	2	Yes	tibolone	950	INDIRECT
S	Lung	2	Yes	ticlopidine	950	INDIRECT
S	Lung	2	Yes	tienilic acid	163	DIRECT
S	Lung	2	Yes	tirofiban	950	INDIRECT
S	Lung	2	Yes	tolbutamide	163	DIRECT
S	Lung	2	Yes	tolbutamide	950	DIRECT
S	Lung	2	Yes	tolfenamic acid	950	DIRECT
S	Lung	2	Yes	tolfenamic acid	950	DIRECT_IRRELEVANT
S	Lung	2	Yes	tolmetin	476	DIRECT
S	Lung	2	Yes	tolmetin	950	DIRECT
S	Lung	2	Yes	topiramate	318	DIRECT
S	Lung	2	Yes	torasemide	950	DIRECT
S	Lung	2	Yes	torasemide	950	INDIRECT
S	Lung	2	Yes	toremifene	163	DIRECT
S	Lung	2	Yes	toremifene	950	DIRECT
S	Lung	2	Yes	tramadol	617	DIRECT_IRRELEVANT
S	Lung	2	Yes	tramadol	950	DIRECT
S	Lung	2	Yes	trandolapril	950	INDIRECT
S	Lung	2	Yes	tranexamic acid	671	DIRECT
S	Lung	2	Yes	trapidil	950	DIRECT
S	Lung	2	Yes	trifluoperazine	211	INDIRECT

S	Lung	2	Yes	trifluoperazine	461	DIRECT_INDIRECT
S	Lung	2	Yes	trifluoperazine	619	INDIRECT
S	Lung	2	Yes	trifluoperazine	800	DIRECT
S	Lung	2	Yes	trifluoperazine	950	DIRECT
S	Lung	2	Yes	trifluoperazine	950	INDIRECT
S	Lung	2	Yes	triflupromazine	696	DIRECT
S	Lung	2	Yes	triflupromazine	924	DIRECT
S	Lung	2	Yes	triflusal	950	DIRECT
S	Lung	2	Yes	trimebutine	617	DIRECT
S	Lung	2	Yes	trimeprazine	950	DIRECT
S	Lung	2	Yes	trimipramine	163	DIRECT
S	Lung	2	Yes	tripelennamine	950	DIRECT
S	Lung	2	Yes	triprolidine	950	DIRECT
S	Lung	2	Yes	Tris buffer	866	DIRECT
S	Lung	2	Yes	Triton WR-1339	240	INDIRECT
S	Lung	2	Yes	tritoqualine	950	DIRECT
S	Lung	2	Yes	troglitazone	163	INDIRECT
S	Lung	2	Yes	troglitazone	283	INDIRECT
S	Lung	2	Yes	troglitazone	320	INDIRECT
S	Lung	2	Yes	troglitazone	366	INDIRECT
S	Lung	2	Yes	troglitazone	499	INDIRECT
S	Lung	2	Yes	troglitazone	684	INDIRECT
S	Lung	2	Yes	troglitazone	950	INDIRECT
S	Lung	2	Yes	tropicamide	389	INDIRECT
S	Lung	2	Yes	tropisetron	950	DIRECT
S	Lung	2	Yes	trospium chloride	903	DIRECT
S	Lung	2	Yes	unoprostone	950	DIRECT
S	Lung	2	Yes	valdecoxib	950	DIRECT
S	Lung	2	Yes	valdecoxib	950	DIRECT_IRRELEVANT
S	Lung	2	Yes	valproic acid	412	DIRECT
S	Lung	2	Yes	valproic acid	929	DIRECT
S	Lung	2	Yes	valsartan	950	DIRECT
S	Lung	2	Yes	valsartan	950	INDIRECT
S	Lung	2	Yes	Vena	950	DIRECT
S	Lung	2	Yes	venlafaxine	950	DIRECT
S	Lung	2	Yes	verapamil	112	DIRECT
S	Lung	2	Yes	verapamil	169	DIRECT
S	Lung	2	Yes	verapamil	233	DIRECT
S	Lung	2	Yes	verapamil	318	DIRECT
S	Lung	2	Yes	verapamil	389	INDIRECT
S	Lung	2	Yes	verapamil	950	DIRECT_INDIRECT
S	Lung	2	Yes	verapamil	950	INDIRECT
S	Lung	2	Yes	vigabatrin	950	DIRECT_INDIRECT
S	Lung	2	Yes	vinblastine	581	DIRECT
S	Lung	2	Yes	vinblastine	950	DIRECT
S	Lung	2	Yes	vincristine	581	DIRECT
S	Lung	2	Yes	vinorelbine	581	DIRECT
S	Lung	2	Yes	voglibose	950	DIRECT
S	Lung	2	Yes	voglibose	950	INDIRECT
S	Lung	2	Yes	warfarin	98	INDIRECT
S	Lung	2	Yes	warfarin	197	INDIRECT
S	Lung	2	Yes	warfarin	950	DIRECT
S	Lung	2	Yes	warfarin	950	INDIRECT
S	Lung	2	Yes	ximelagatran	923	INDIRECT
S	Lung	2	Yes	ximelagatran	950	DIRECT

S	Lung	2	Yes	yohimbine	696	DIRECT_IRRELEVANT
S	Lung	2	Yes	yohimbine	950	INDIRECT
S	Lung	2	Yes	zafirlukast	675	DIRECT
S	Lung	2	Yes	zafirlukast	950	DIRECT
S	Lung	2	Yes	ziprasidone	381	DIRECT
S	Lung	2	Yes	ziprasidone	696	DIRECT
S	Lung	2	Yes	ziprasidone	800	DIRECT
S	Lung	2	Yes	ziprasidone	950	DIRECT
S	Lung	2	Yes	zoledronic acid	950	INDIRECT
S	Lung	2	Yes	zomepirac	950	DIRECT
S	Lung	2	Yes	zonisamide	318	DIRECT
S	Lung	2	Yes	zonisamide	474	DIRECT
S	Lung	2	Yes	zonisamide	828	DIRECT
S100A8	Multi-Organ	1	No	amlexanox	364	DIRECT
S100A9	Multi-Organ	1	No	amlexanox	950	DIRECT
SCN5A	Multi-Organ	4	No	flecainide	950	DIRECT_INDIRECT
SCN5A	Multi-Organ	4	No	lidocaine	950	DIRECT
SELE	Multi-Organ	4	No	probucol	950	INDIRECT
SELE	Multi-Organ	4	No	selegiline	950	DIRECT
SELE	Multi-Organ	4	No	selegiline	950	INDIRECT
SERPINC1	Liver	1	No	25-hydroxyvitamin D	274	DIRECT
SERPINC1	Liver	1	No	aspirin	950	DIRECT
SERPINC1	Liver	1	No	danazol	950	INDIRECT
SERPINC1	Liver	1	No	fondaparinux sodium	950	DIRECT
SERPINC1	Liver	1	No	ticlopidine	950	INDIRECT
SLC11A2	Heart	1	No	desferrioxamine	950	INDIRECT
SLC12A2	Marrow	1	No	bumetanide	950	DIRECT
SLC12A2	Marrow	1	No	furosemide	950	DIRECT
SLC25A4	Multi-Organ	1	No	amiloride	197	DIRECT
SLC8A1	Heart	1	No	amiloride	197	DIRECT
SLC8A1	Heart	1	No	ouabain	560	INDIRECT
SMC2	Marrow	1	Yes	bortezomib	214	DIRECT
SORT1	Multi-Organ	1	Yes	amphetamine	730	INDIRECT
SORT1	Multi-Organ	1	Yes	levocabastine	730	DIRECT
TBXA2R	Liver	1	No	AA-2414	950	DIRECT
TBXA2R	Liver	1	No	glibenclamide	950	DIRECT
TBXA2R	Liver	1	No	losartan	950	DIRECT
TBXA2R	Liver	1	No	misoprostol	584	DIRECT
TBXA2R	Liver	1	No	picotamide	950	DIRECT
TBXA2R	Liver	1	No	torasemide	950	DIRECT
TBXA2R	Liver	1	No	trimetoquinol	950	DIRECT_INDIRECT
TEK	Multi-Organ	1	No	imatinib	160	DIRECT
TF	Multi-Organ	1	Yes	19-nortestosterone	240	INDIRECT
TF	Multi-Organ	1	Yes	acarbose	240	INDIRECT
TF	Multi-Organ	1	Yes	bezafibrate	240	INDIRECT
TF	Multi-Organ	1	Yes	ciprofibrate	240	INDIRECT
TF	Multi-Organ	1	Yes	clofibrate	240	DIRECT
TF	Multi-Organ	1	Yes	desferrioxamine	901	INDIRECT
TF	Multi-Organ	1	Yes	desferrioxamine	950	INDIRECT
TF	Multi-Organ	1	Yes	desogestrel	240	INDIRECT
TF	Multi-Organ	1	Yes	diethylstilbestrol	950	INDIRECT
TF	Multi-Organ	1	Yes	doxazosin	240	INDIRECT
TF	Multi-Organ	1	Yes	estrogen	240	INDIRECT
TF	Multi-Organ	1	Yes	fluvastatin	240	INDIRECT

TF	Multi-Organ	1	Yes	gemfibrozil	240	INDIRECT
TF	Multi-Organ	1	Yes	glibenclamide	240	INDIRECT
TF	Multi-Organ	1	Yes	imatinib	159	DIRECT
TF	Multi-Organ	1	Yes	lovastatin	240	INDIRECT
TF	Multi-Organ	1	Yes	metformin	671	INDIRECT
TF	Multi-Organ	1	Yes	metformin	865	INDIRECT
TF	Multi-Organ	1	Yes	metformin	911	DIRECT
TF	Multi-Organ	1	Yes	metformin	928	INDIRECT
TF	Multi-Organ	1	Yes	metformin	950	DIRECT
TF	Multi-Organ	1	Yes	metformin	950	INDIRECT
TF	Multi-Organ	1	Yes	pioglitazone	240	INDIRECT
TF	Multi-Organ	1	Yes	probucol	240	DIRECT
TF	Multi-Organ	1	Yes	rapamycin	159	INDIRECT
TF	Multi-Organ	1	Yes	riluzole	306	INDIRECT
TF	Multi-Organ	1	Yes	Ronicol	240	INDIRECT
TF	Multi-Organ	1	Yes	rose bengal	708	DIRECT
TF	Multi-Organ	1	Yes	rosiglitazone	240	INDIRECT
TF	Multi-Organ	1	Yes	rosuvastatin	240	INDIRECT
TF	Multi-Organ	1	Yes	streptozotocin	240	INDIRECT
TF	Multi-Organ	1	Yes	tamoxifen	159	INDIRECT
TF	Multi-Organ	1	Yes	tibolone	240	INDIRECT
TF	Multi-Organ	1	Yes	Triton WR-1339	240	INDIRECT
TFRC	Multi-Organ	1	No	desferrioxamine	950	INDIRECT
TGFA	Multi-Organ	2	Yes	tamoxifen	950	INDIRECT
TGFBR1	Placenta	6	No	imatinib	159	DIRECT
TGFBR1	Placenta	6	No	rapamycin	159	INDIRECT
TGFBR1	Placenta	6	No	tamoxifen	159	INDIRECT
TGM2	Multi-Organ	2	Yes	clofibrate	80	INDIRECT
TUBG1	Marrow	2	Yes	colchicine	581	DIRECT
TUBG1	Marrow	2	Yes	docetaxel	581	DIRECT
TUBG1	Marrow	2	Yes	vinblastine	581	DIRECT
TUBG1	Marrow	2	Yes	vincristine	581	DIRECT
TUBG1	Marrow	2	Yes	vinorelbine	581	DIRECT
TYK2	Bowel	2	Yes	imatinib	94	DIRECT
UBB	Liver	1	No	colchicine	581	DIRECT
UBB	Liver	1	No	docetaxel	581	DIRECT
UBB	Liver	1	No	vinblastine	581	DIRECT
UBB	Liver	1	No	vincristine	581	DIRECT
UBB	Liver	1	No	vinorelbine	581	DIRECT
USP2	Heart	1	No	clopidogrel	950	INDIRECT
VCL	Liver	2	Yes	colcemid	950	INDIRECT
VCL	Liver	2	Yes	colchicine	950	INDIRECT
VDR	Multi-Organ	1	No	25-hydroxyvitamin D	950	INDIRECT
VDR	Multi-Organ	1	No	calcipotriol	950	DIRECT
VLDLR	Heart	1	No	lovastatin	283	INDIRECT
VLDLR	Heart	1	No	probucol	283	INDIRECT
VLDLR	Heart	1	No	troglitazone	283	INDIRECT
VWF	Multi-Organ	2	No	acenocoumarol	197	INDIRECT
VWF	Multi-Organ	2	No	bezafibrate	197	INDIRECT
VWF	Multi-Organ	2	No	DDAVP	950	INDIRECT
VWF	Multi-Organ	2	No	desogestrel	197	INDIRECT
VWF	Multi-Organ	2	No	ticlopidine	950	INDIRECT
VWF	Multi-Organ	2	No	warfarin	197	INDIRECT
YAP1	Multi-Organ	9	Yes	riluzole	306	INDIRECT
YES1	Marrow	3	Yes	imatinib	94	DIRECT
