# Supplementary figures and images for: A protein interactions map of multiple organ systems associated with COVID-19 disease
Source: Genomics Inform. 2021 Jun 30;19(2):e14. doi: 10.5808/gi.20078 (PMC8261268; doi:10.5808/gi.20078)

**Bowel**

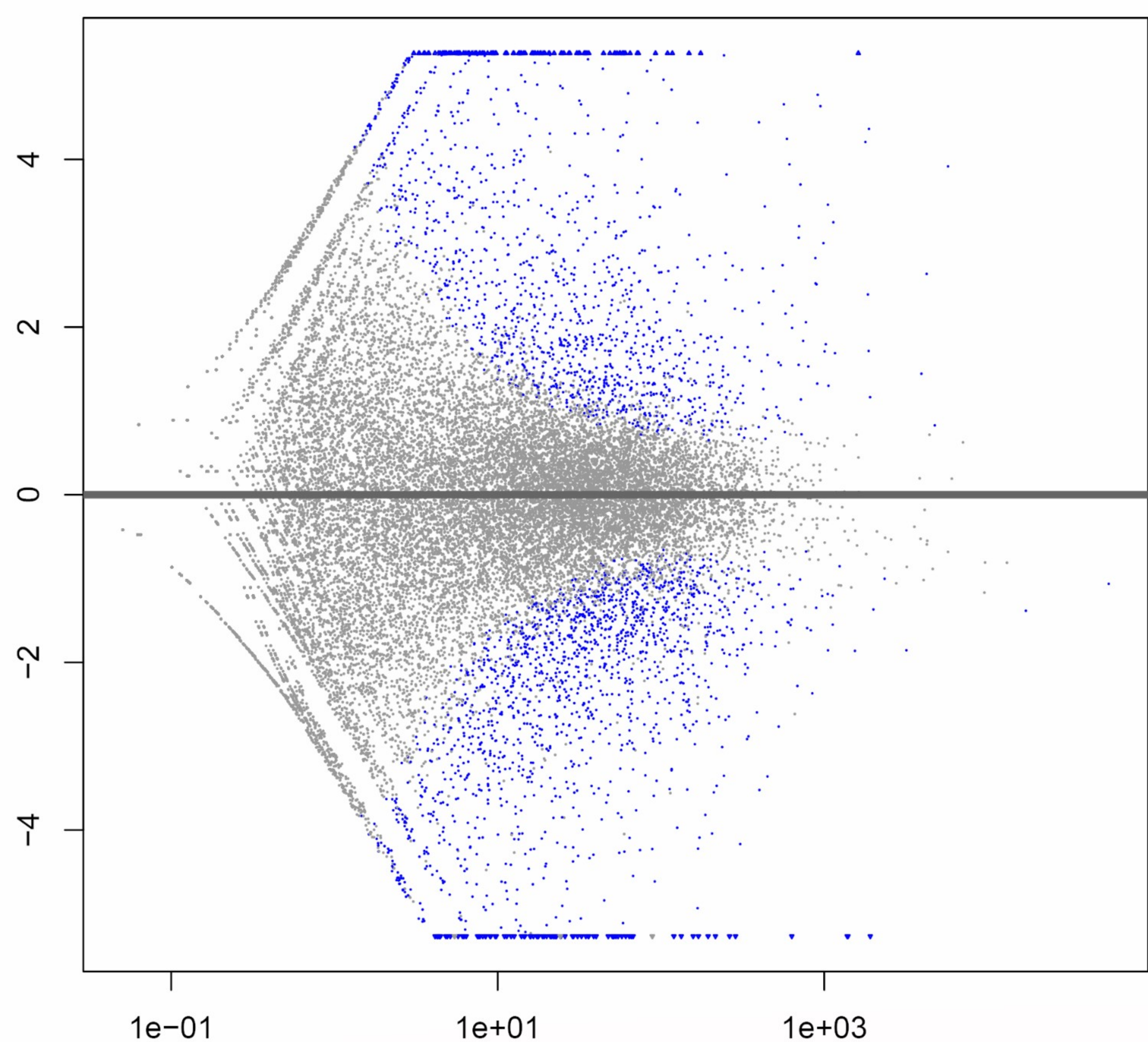

**Fat**

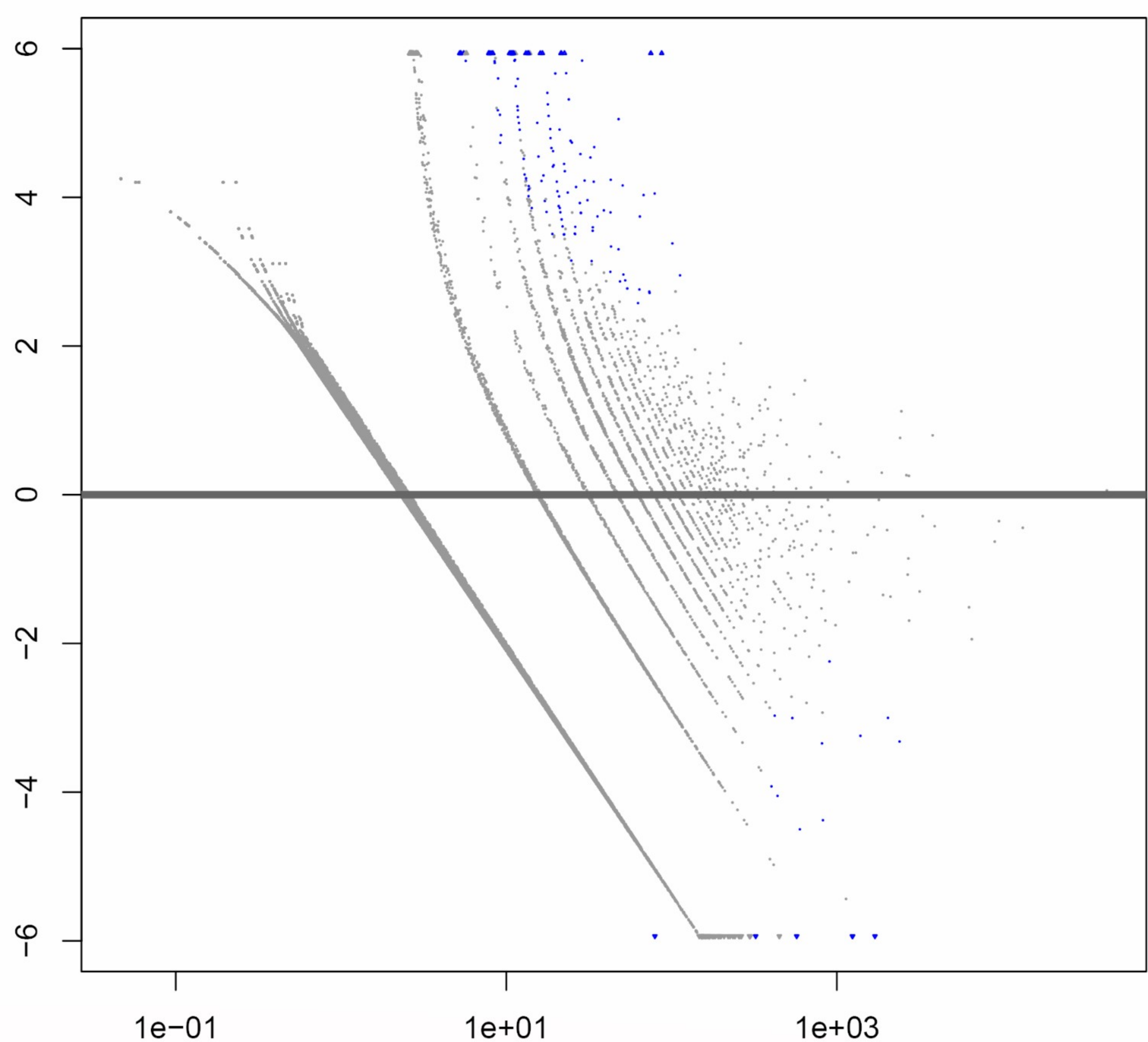

**Heart**

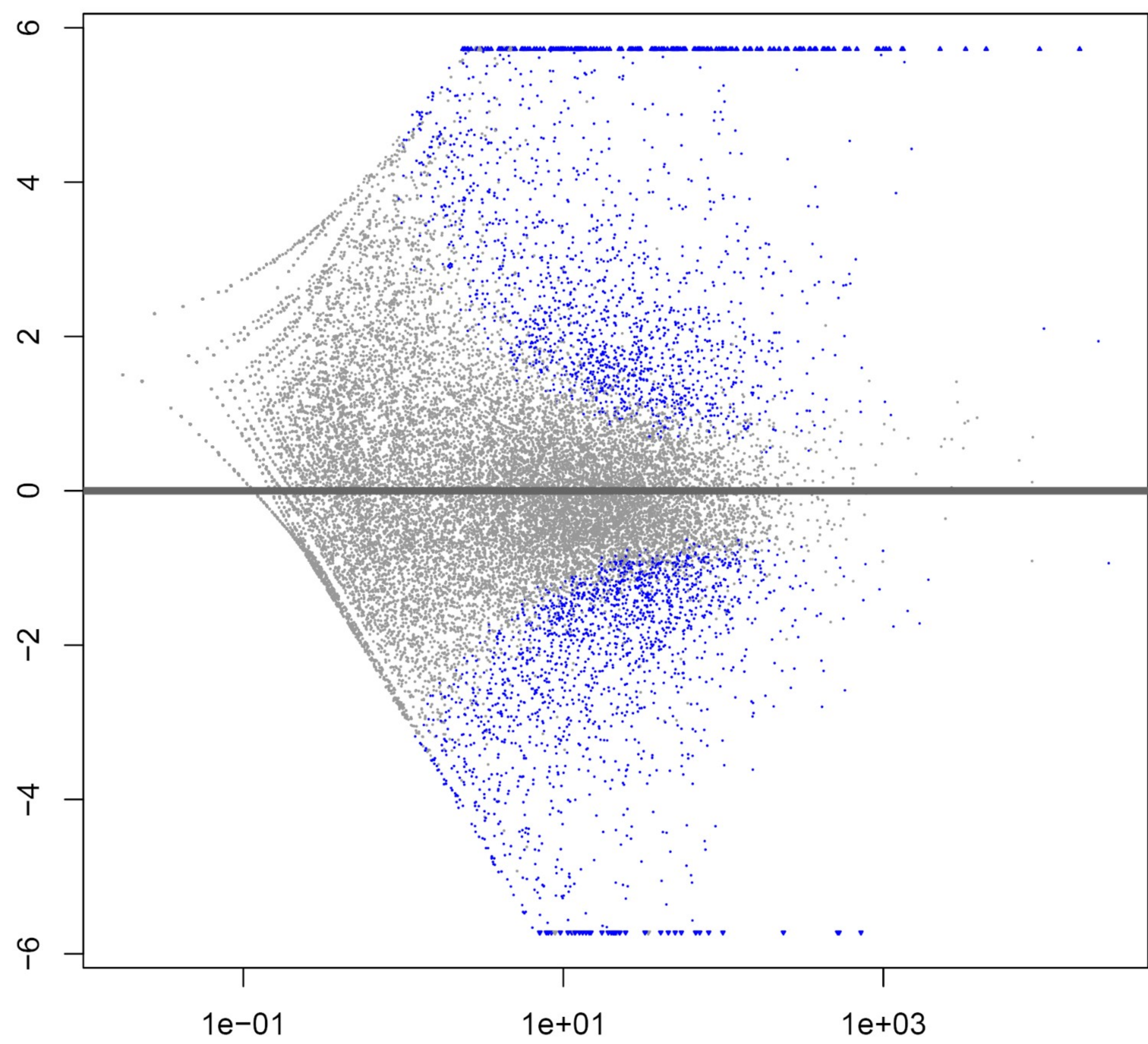

**Jejunum**

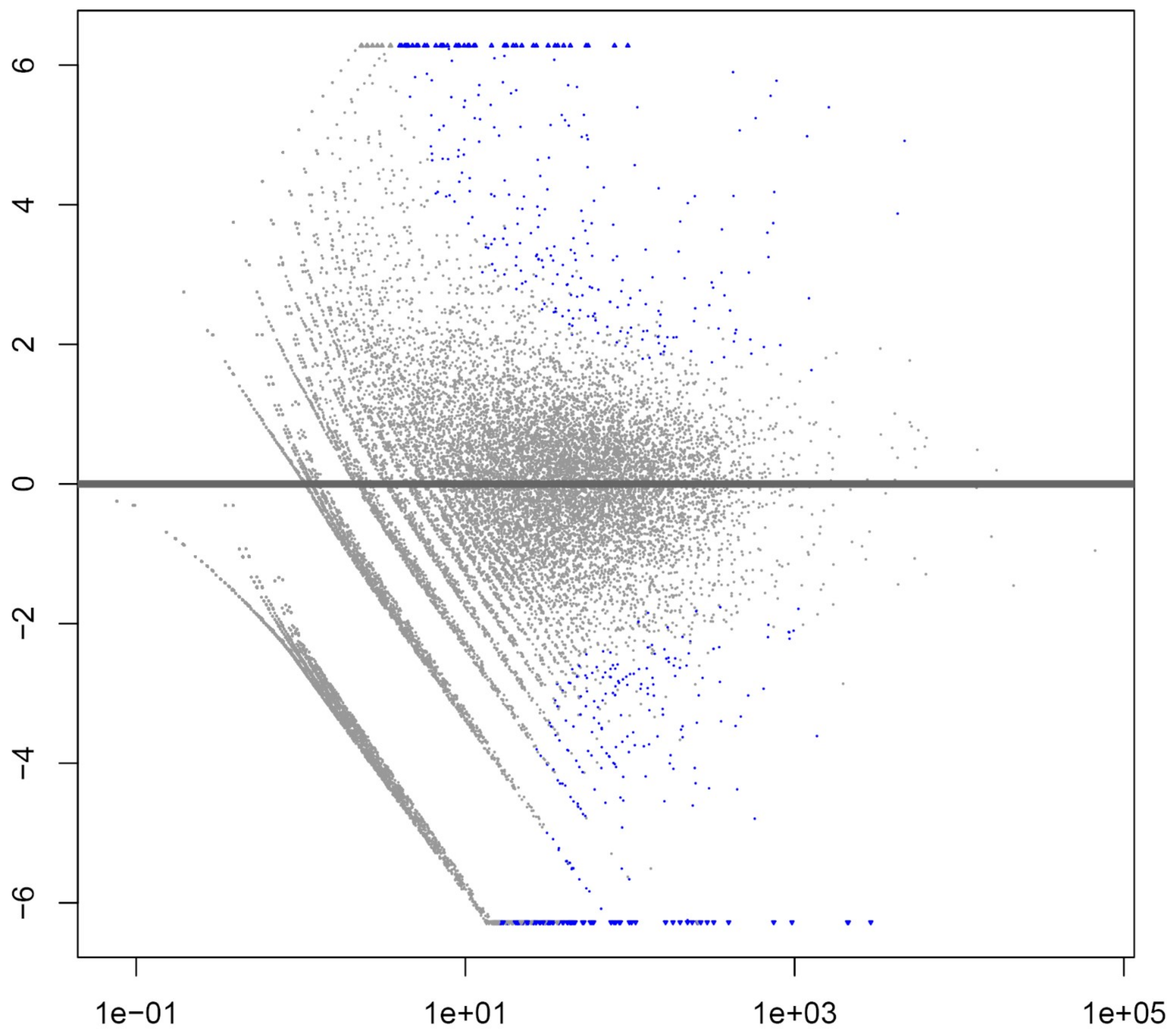

**Kidney**

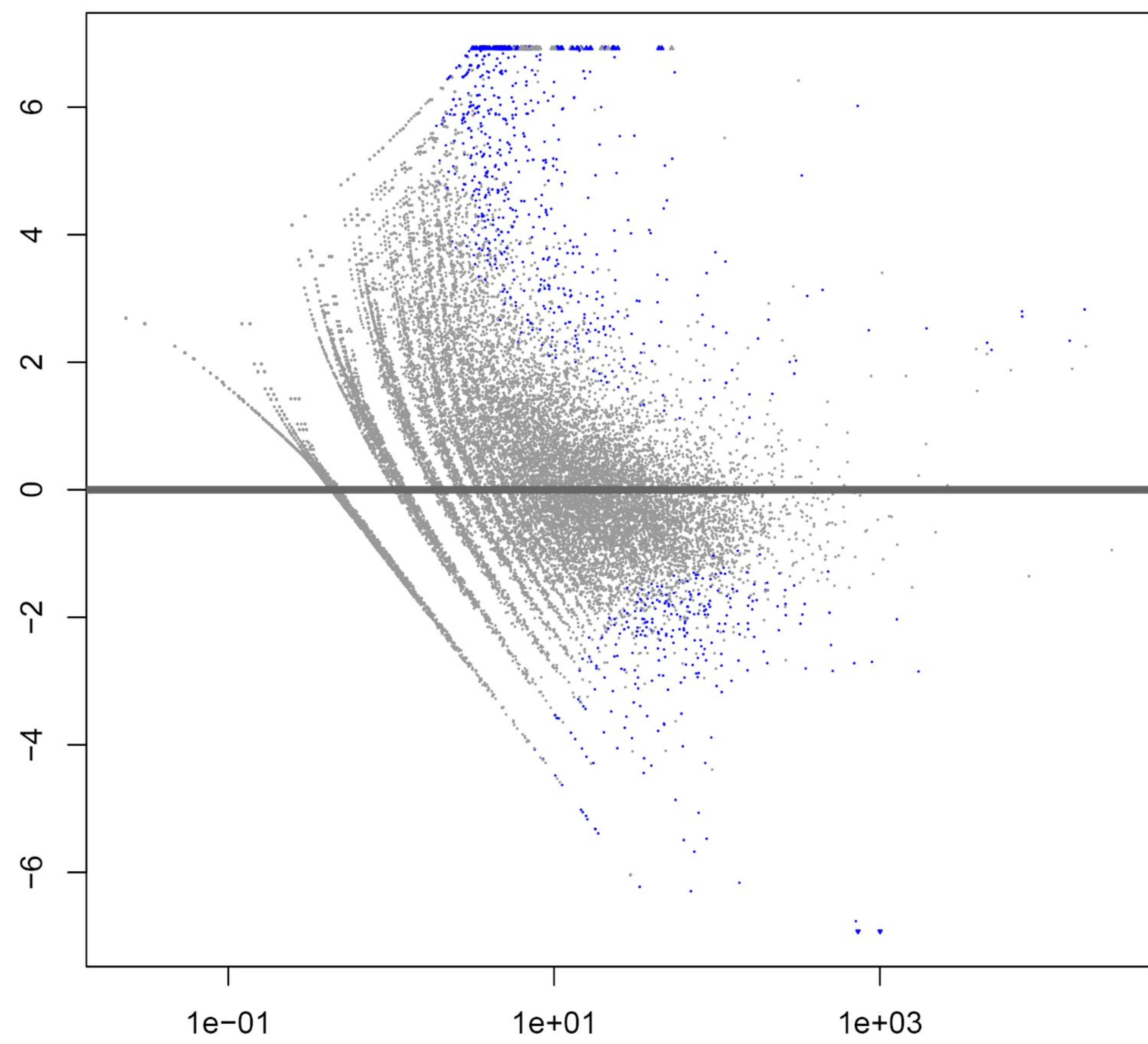

**Liver**

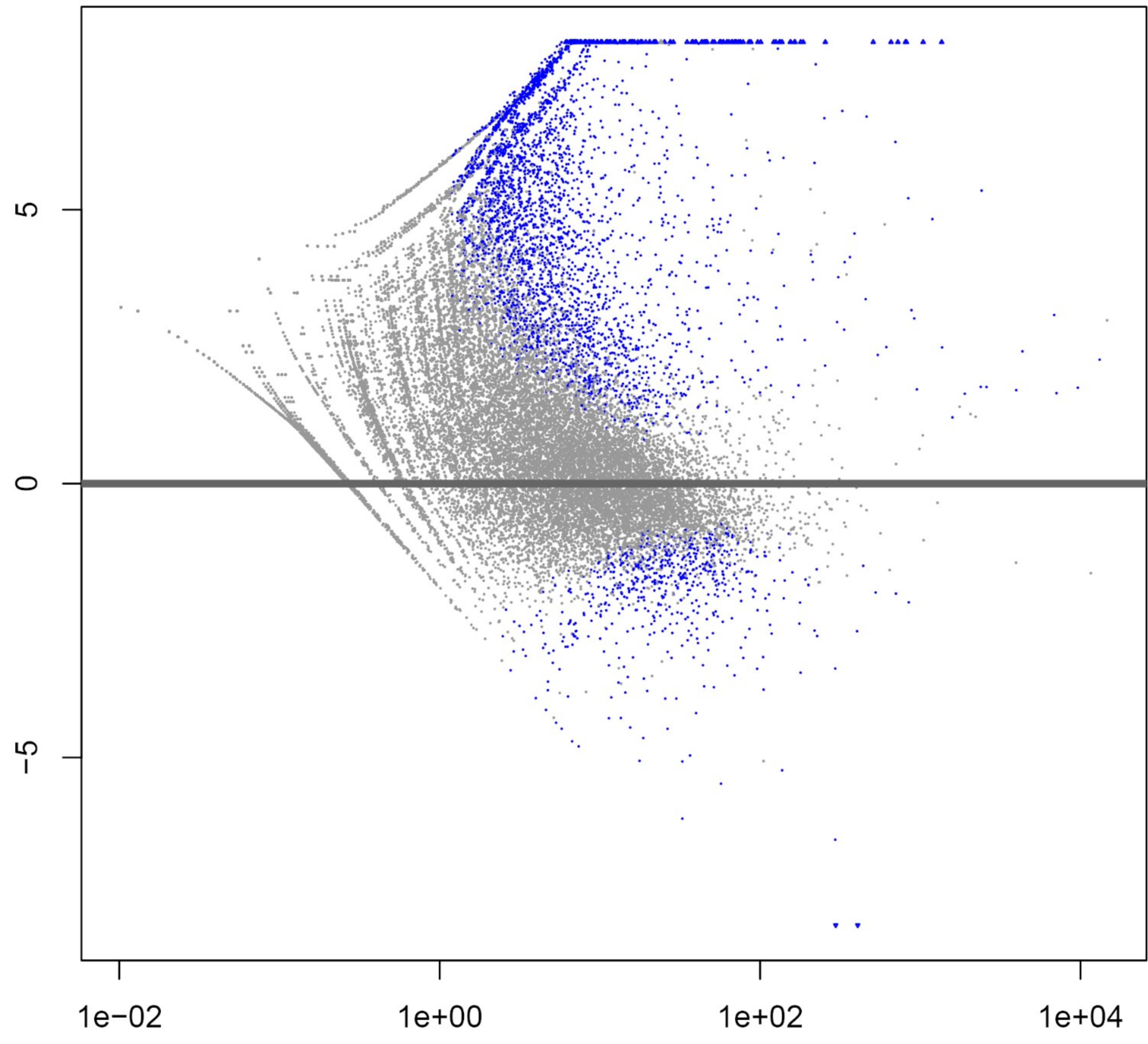

**Lung**

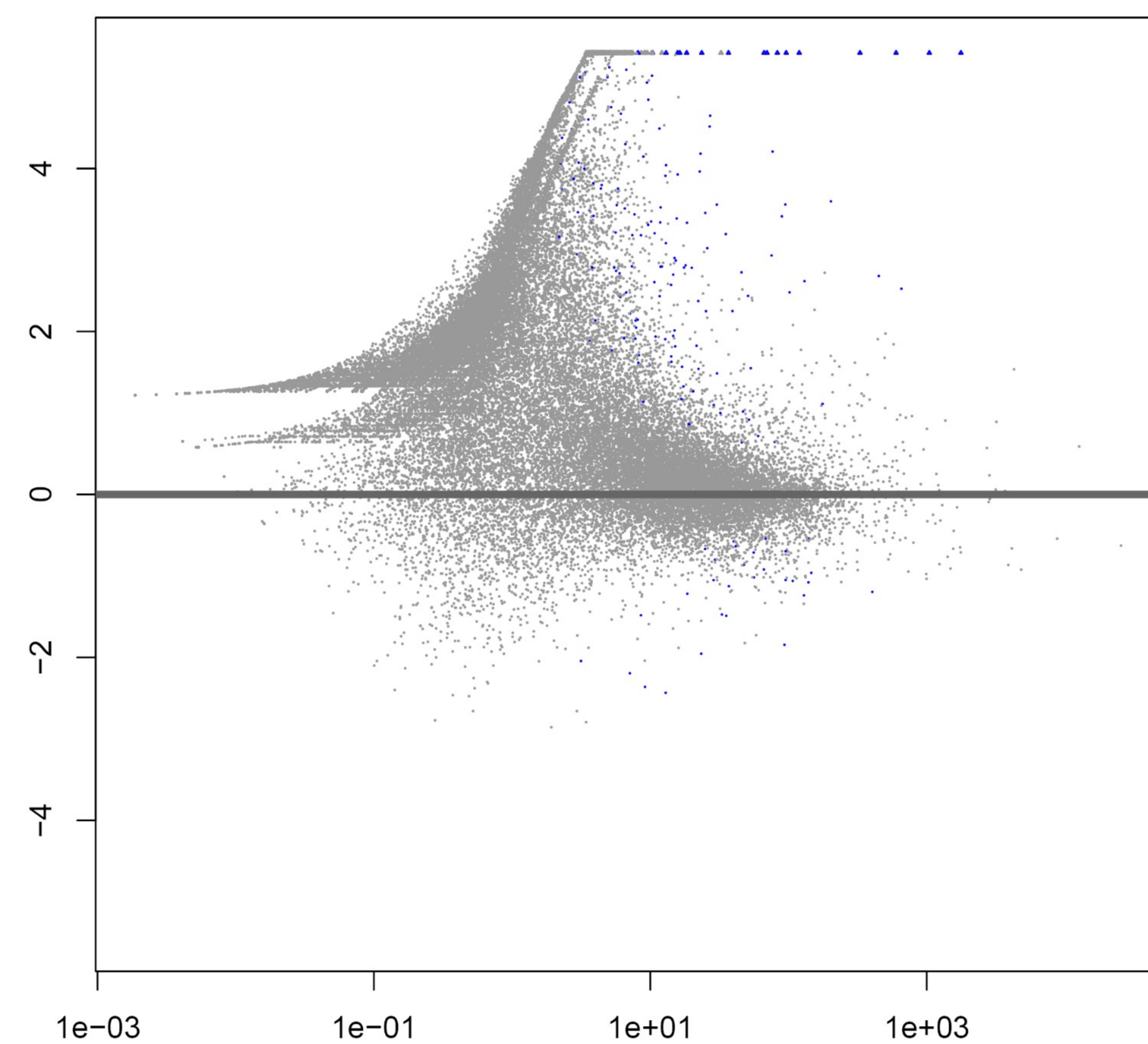

**Marrow**

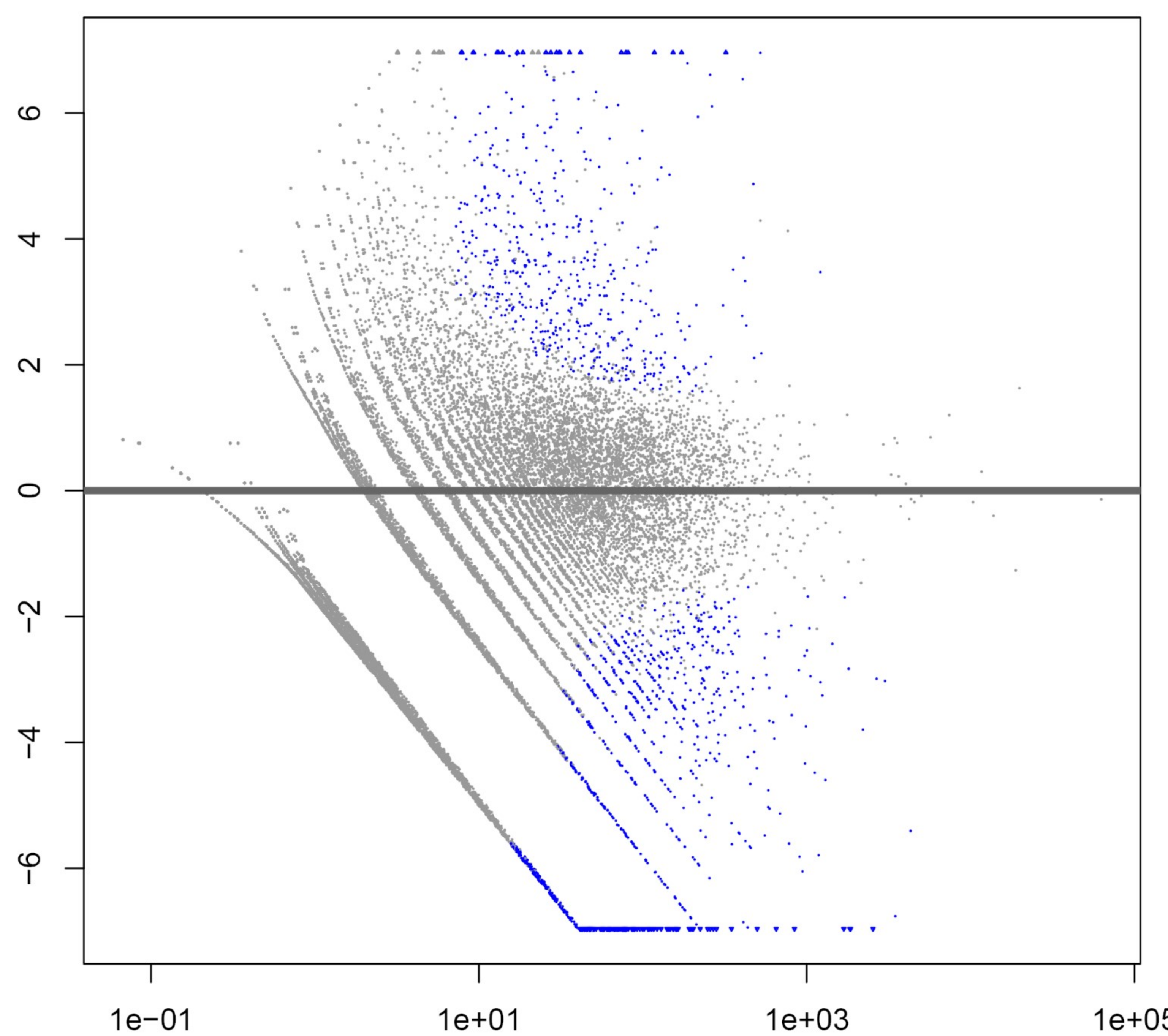

**Placenta**

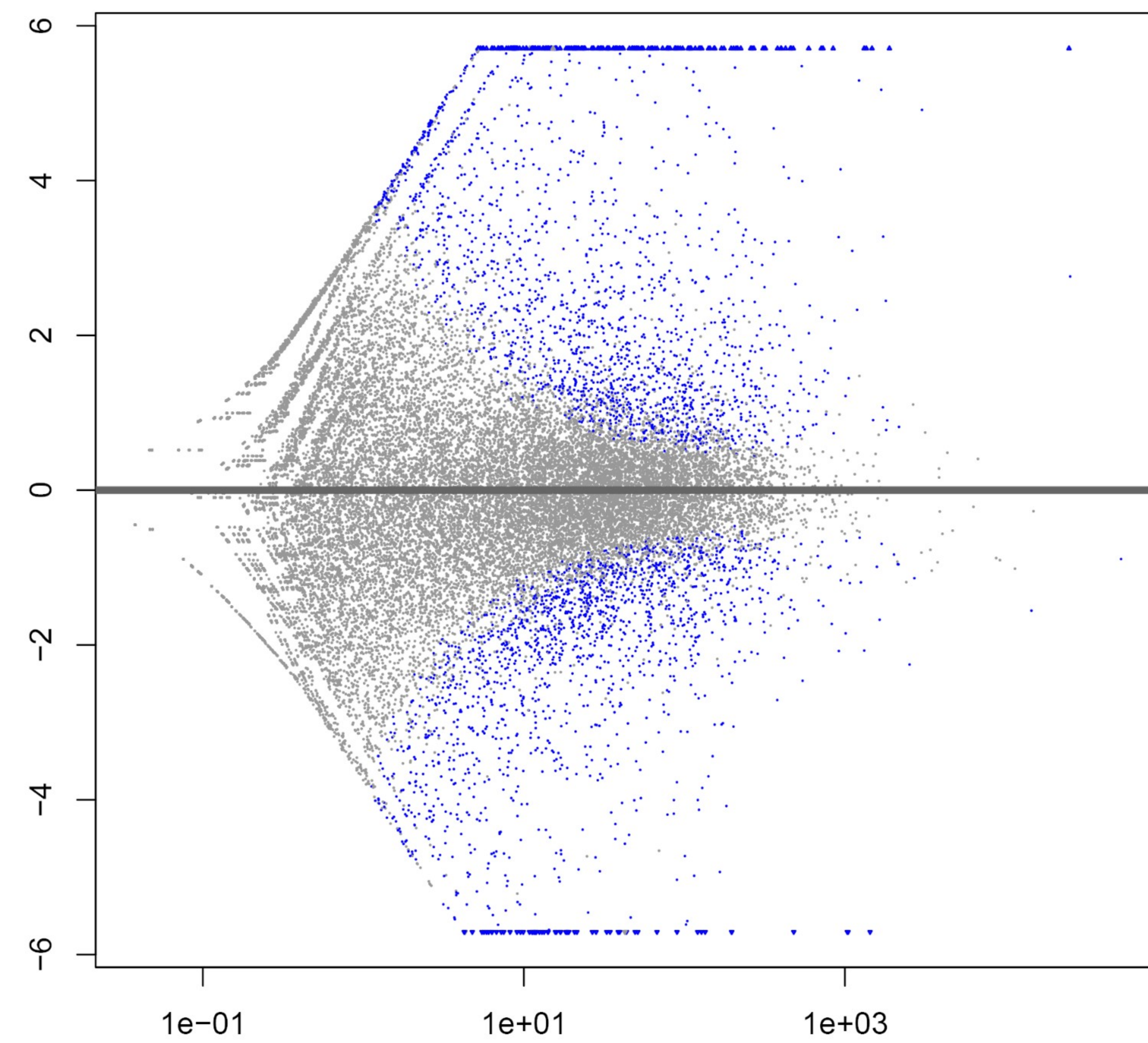

**Skin**

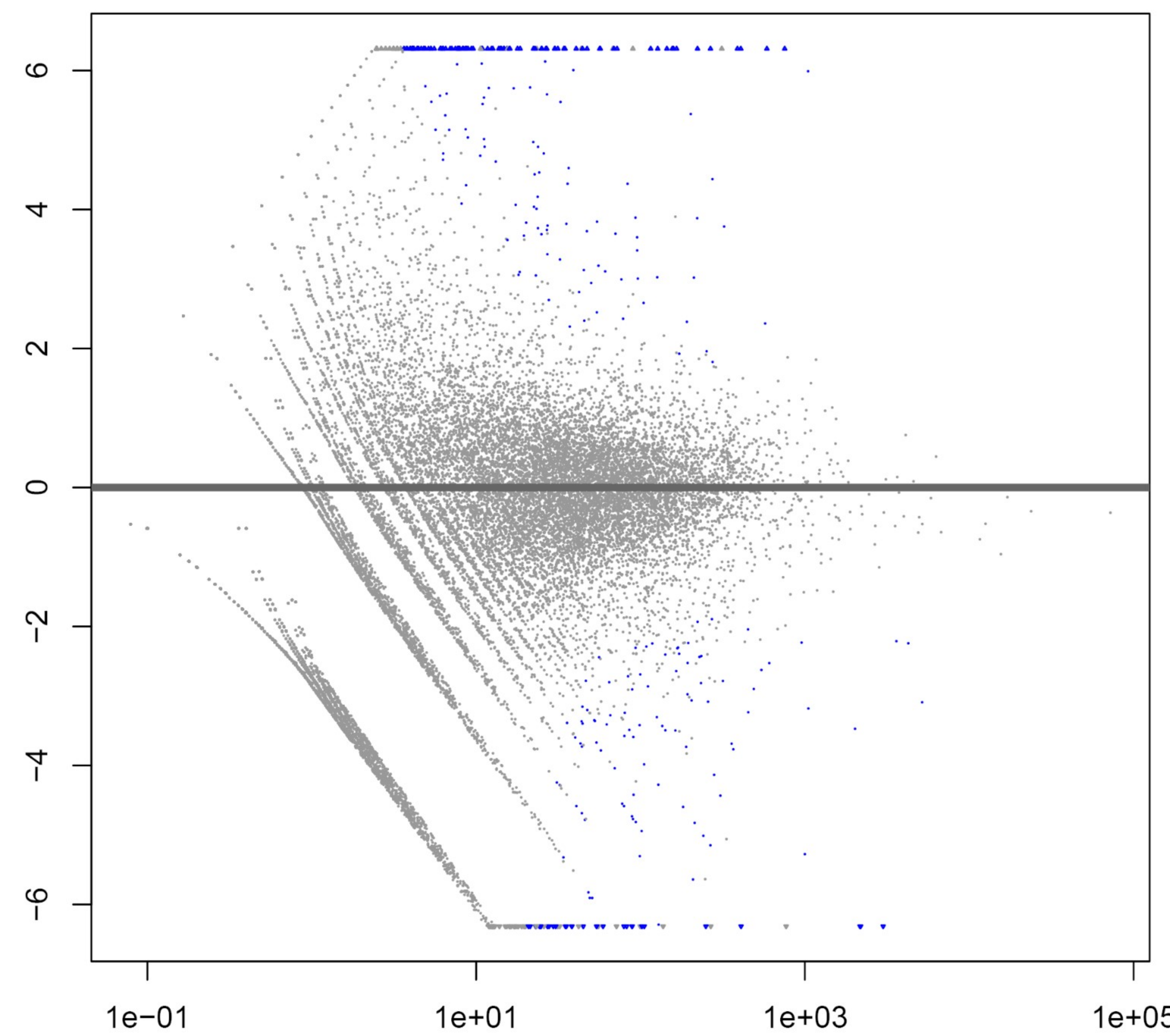

Supplement: Supplementary Fig. 1. — MA-plot of the DESeq2 processed data. The mean of normalized counts is represented on the X-axis while the log fold change values on the y-axis. [file gi-20078-suppl4.pdf]

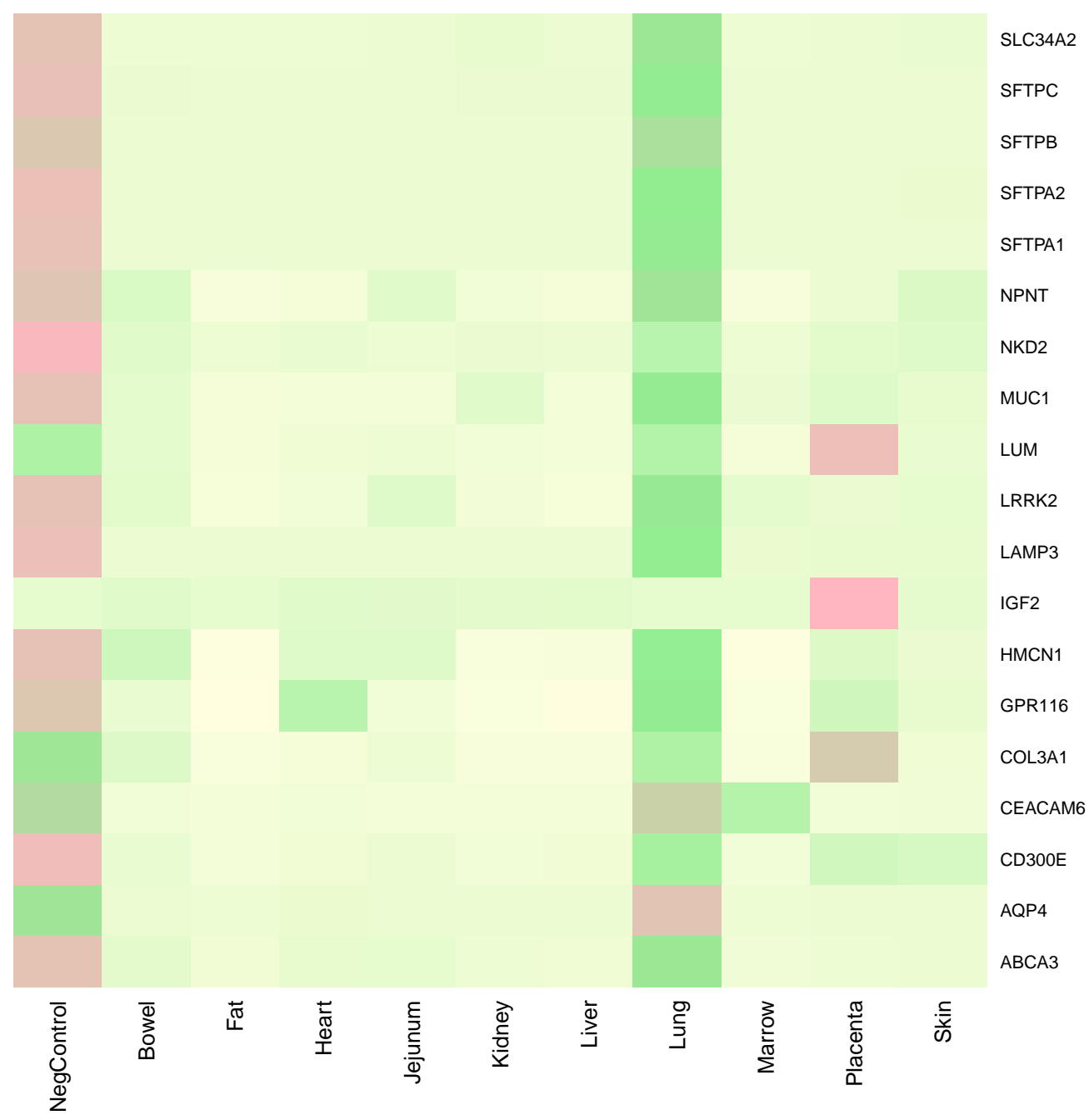

Supplement: Supplementary Fig. 2. — A heatmap of organ-wise averaged read counts of genes differentially regulated in more than seven organ systems. Light yellow color represents the maximum value, light yellow the mini-mum and light-green color in between maximum and minimum values. [file gi-20078-suppl5.pdf]

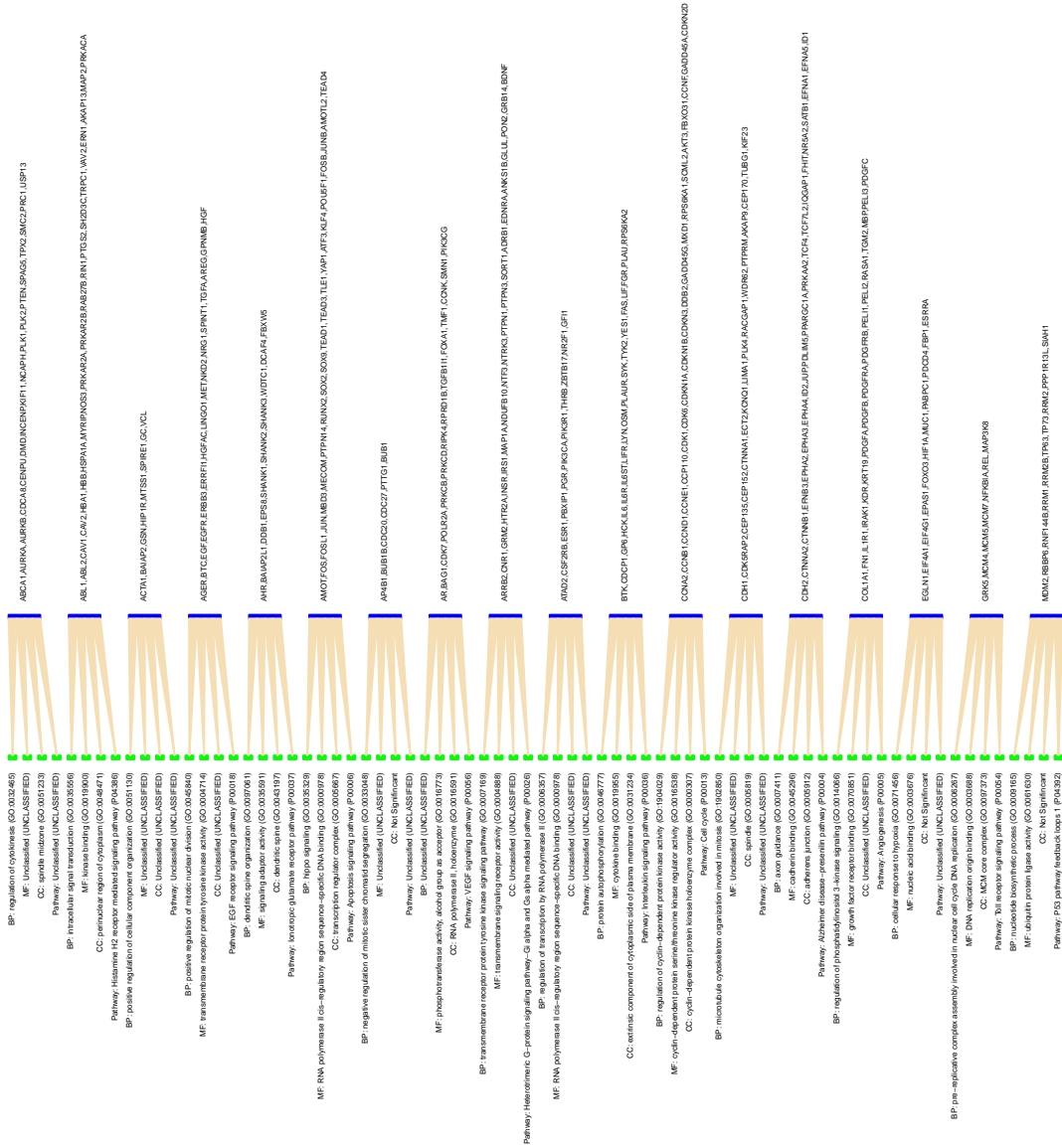

BP: one-rod complex assembly involved

[illegible]

Supplement: Supplementary Fig. 4. — A graph of top functional annotations and pathways of genes of different modules. [file gi-20078-suppl7.pdf]

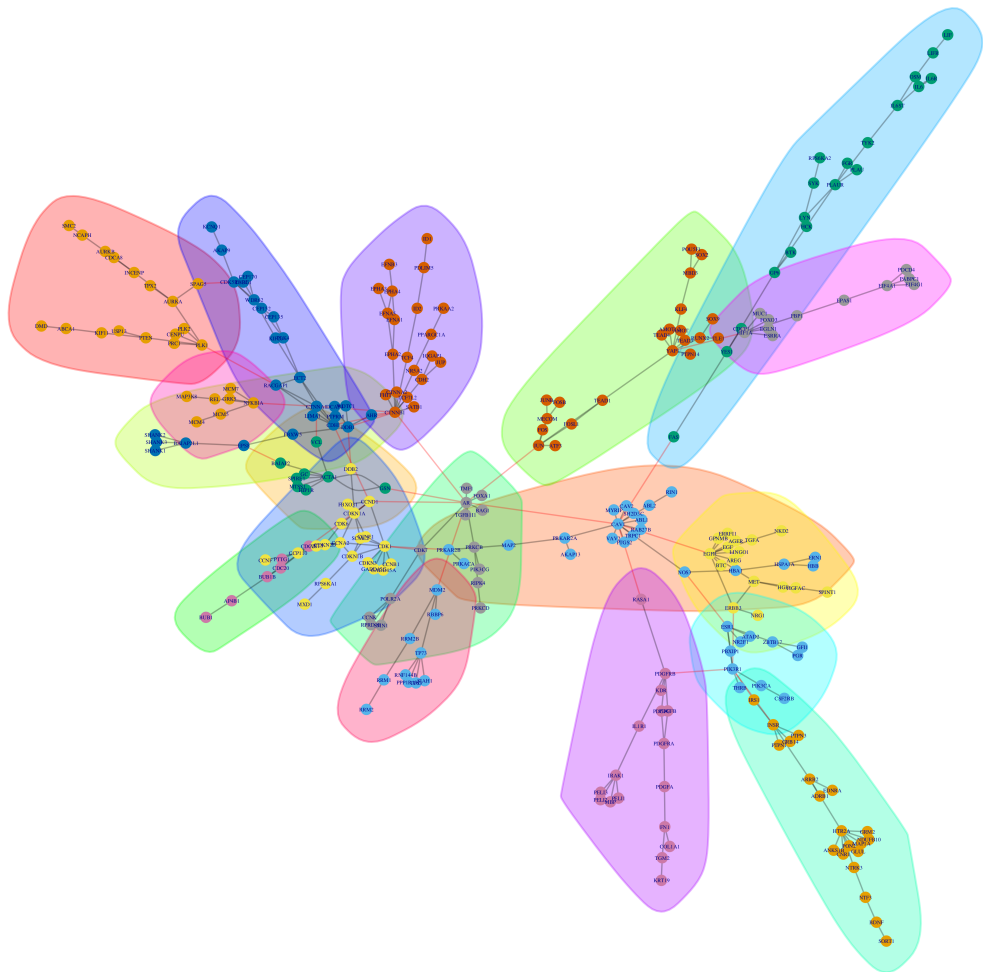

Supplement: Supplementary Fig. 5. — A map of modules in the core interactions sub-network. [file gi-20078-suppl8.pdf]
