# Supplementary material for: A protein interactions map of multiple organ systems associated with COVID-19 disease
Source: Genomics Inform. 2021 Jun 30;19(2):e14. doi: 10.5808/gi.20078 (PMC8261268; doi:10.5808/gi.20078)
Supplement: Supplementary Fig. 3. — A map of core interactions sub-network. [file gi-20078-suppl6.pdf]

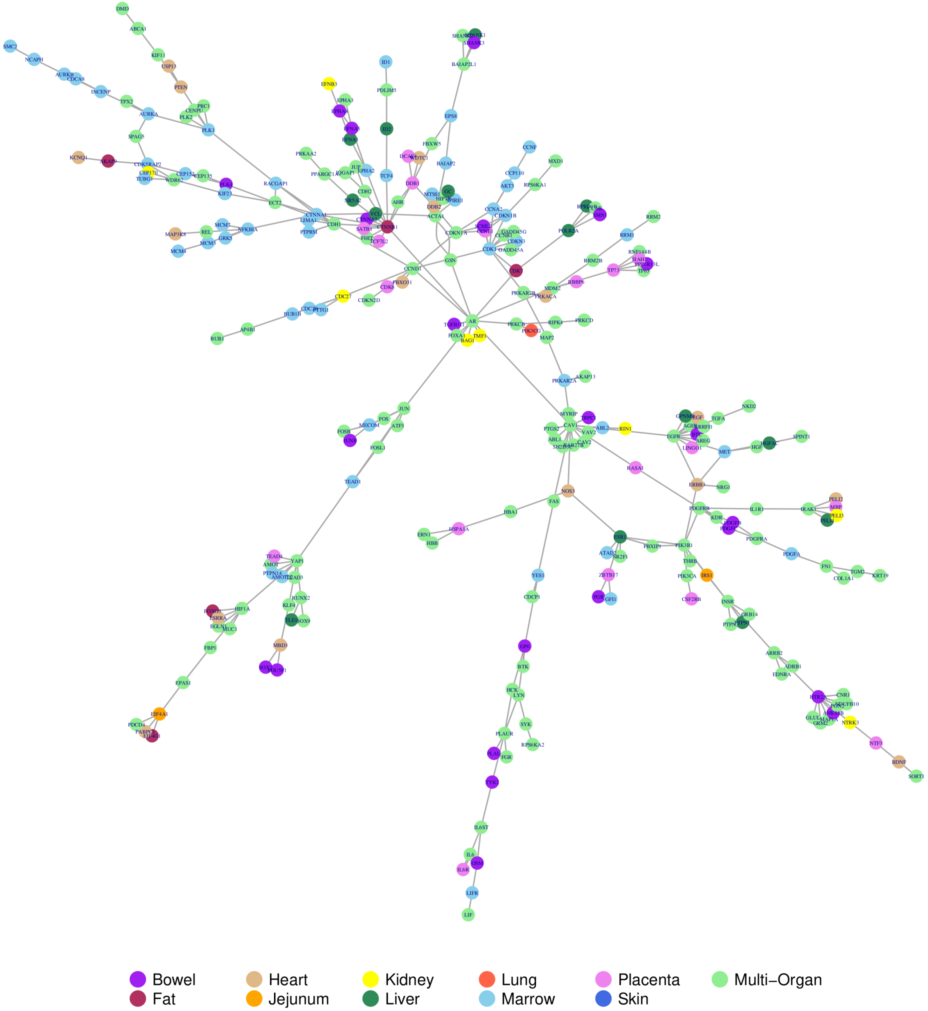
Bowel
Heart
Kidney
Lung
Placenta
Multi−Organ
Fat
Jejunum
Liver
Marrow
Skin
